# Gold-catalyzed (4 + 2)-annulations between α-alkyl alkenylgold carbenes and benzisoxazoles with reactive alkyl groups†
†Electronic supplementary information (ESI) available. CCDC 1819135–1819138. For ESI and crystallographic data in CIF or other electronic format see DOI: 10.1039/c8sc00986d


**DOI:** 10.1039/c8sc00986d

**Published:** 2018-04-23

**Authors:** Bhanudas Dattatray Mokar, Prakash D. Jadhav, Y. B. Pandit, Rai-Shung Liu

**Affiliations:** a Frontier Research Centers for Materials Science and Technology , Department of Chemistry , National Tsing-Hua University , Hsinchu , Taiwan , Republic of China . Email: rsliu@mx.nthu.edu.tw

## Abstract

This work reports new (4 + 2)-annulations of α-alkyl vinylgold carbenes with benzisoxazoles to afford 3,4-dihydroquinoline derivatives with high *anti*-stereoselectivity. The annulations are operable with carbenes in both acyclic and cyclic forms. This reaction sequence involves an initial formation of imines from α-alkylgold carbenes and benzisoxazoles, followed by a novel carbonyl-enamine reaction to yield 3,4-dihydroquinoline derivatives. This system presents the first alkyl C–H reactivity of α-alkyl gold carbenes with an external substrate.

## Abstract 2

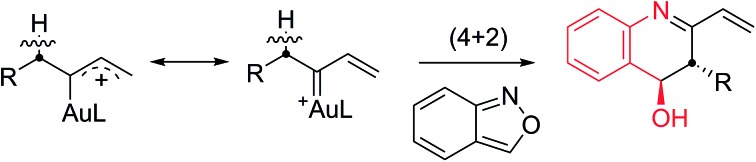
This work reports new (4 + 2)-annulations of α-alkyl vinylgold carbenes with benzisoxazoles to afford 3,4-dihydroquinoline derivatives with high *anti*-stereoselectivity.

## Introduction

Metal carbenes are versatile intermediates to implement a vast number of useful reactions including cyclopropanation, X–H insertion (X = C, N and O), skeletal rearrangement and annulation reactions (eqn (1)).^1^ Despite their widespread applications, applicable metal carbenes, derived from diazo precursors, are mainly restricted to donor/acceptor (**D**/**A**) types **I** (R = H, aryl and alkenyl; EWG = CN, ketones and esters) whereas highly desirable α-alkyl metal carbenes **II** are less efficient because of a competitive 1,2-hydrogen shift to form olefins (eqn (2)).^1^ This side reaction is particularly serious for gold carbenes because their LAu = C^+^ carbons are highly cationic.^2^ Few intermolecular reactions involving Ar–Pd(ii) catalysts focused on α-alkyl metal carbenes of **D**/**A** types.^3^ The limited utility of α-alkyl carbenoids features an unsolved and challenging task in metal carbene chemistry. We seek new α-alkyl carbenoids beyond commonly used **D**/**A** carbenes **II**, aiming at two objectives: (i) suppression of a 1,2-H shift and (ii) an alkyl C–H reaction with an external substrate.
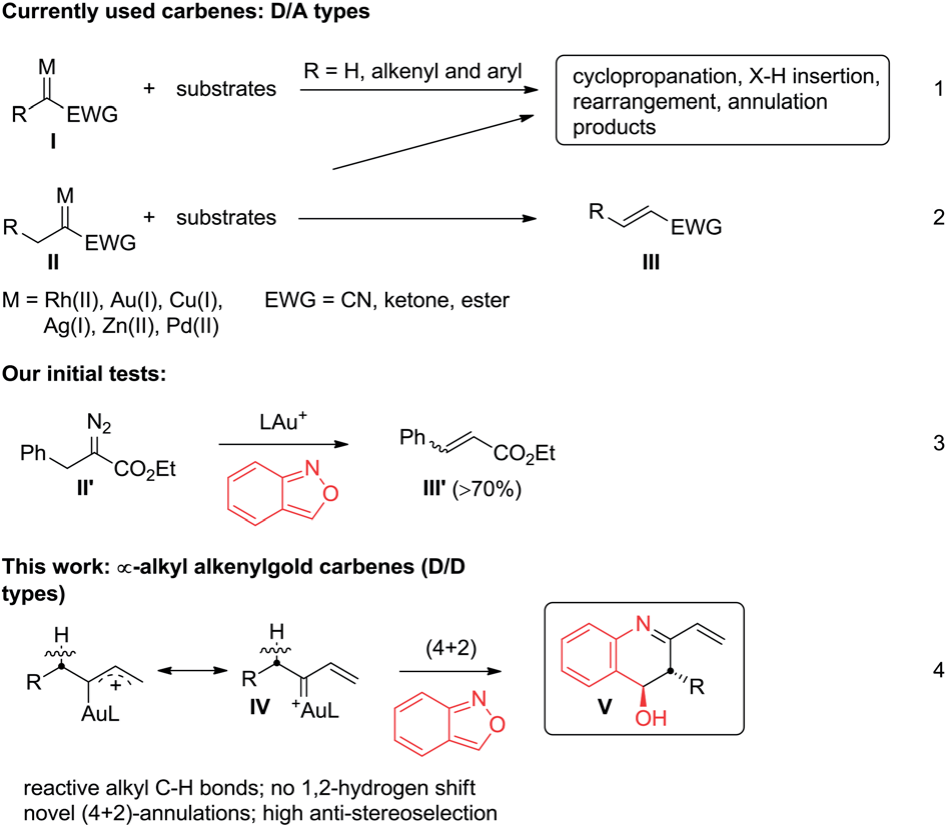


Interest in the reactions of benzisoxazoles is rapidly growing in gold catalysis because of their various annulation modes with gold π-alkynes.^4^–^6^ To explore the reactivity of benzisoxazoles toward gold carbenes,^7^ we first tested the reactions with **D**/**A**-type benzyl α-oxogold carbene **II′** (R = Ph and EWG = CO_2_Et), yielding an olefin product **III′** efficiently (eqn (3)). We envisage that **D**/**D** type carbenes such as α-alkyl alkenylgold carbenes **IV** might be viable species to achieve new annulations with benzisoxazoles because their gold-stabilized allyl cation character **IV** is unfavorable for a 1,2-H shift. According to this hypothesis, this work reports novel intermolecular (4 + 2)-annulations between α-alkyl vinylgold carbenes and benzisoxazoles, thus manifesting an unprecedented C–H reactivity of α-alkyl metal carbenes.

## Results and discussion

As shown in eqn (5), we further tested the reaction of acyclic alkylgold carbenes **A** that were generated *in situ* from cyclopropene derivatives **1a–1b** and gold catalysts.^8^ With IPrAuCl/AgSbF_6_, quinoline derivatives **3a** and **3b** were isolated in satisfactory yields (72–75%), together with enones **1a-O** and **1b-O** in minor proportions (17–19%). A 1,2-hydrogen shift was effectively suppressed with vinylcarbenes **A**, supporting our hypothetic role of gold-stabilized allyl cations **A**.
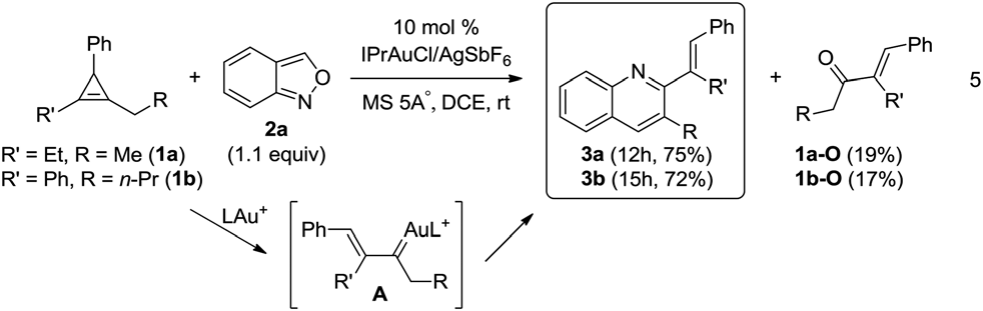


**Fig. 1:**
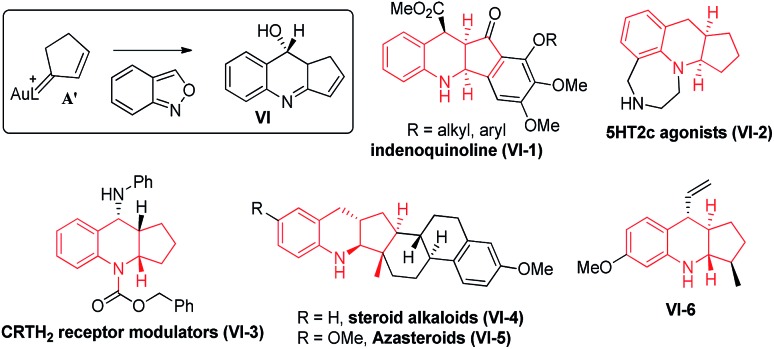
Suitable alkylgold carbenes to access bioactive molecules.

Our primary interest is to construct complicated frameworks *via* cascade reactions. Fig. 1 depicts several bioactive compounds (**VI-1**)–(**VI-6**) bearing a common tricyclic framework **VI**, which can be easily constructed from cyclopentenylgold carbene **A′** and benzisoxazole. Indenoquinoline (**VI-1**) showed antiproliferative activities against breast (MCF-7) and lung epithelial (A-549) cells.9*a* Species **VI-2** and **VI-3** served as 5HT2c agonists and CRTH_2_ receptor modulators, respectively.9b,c Compounds **VI-4** and **VI-5** were N-containing steroids found in higher plants.9d,e Species **VI-6** is a key intermediate for the total synthesis of naturally occurring (–)-isoschizogaline9*f* and (–)-isoschizozygamine.9*g*

In this new task, we optimized the annulation cascades between vinylallene **4a** and benzisoxazole **2a** in dichloromethane (DCM) using various gold catalysts; species **4a** serves as a precursor for cyclopentenylgold carbene **A′** (Table 1).^10^

**Table 1:** Catalytic reactions with various gold catalysts

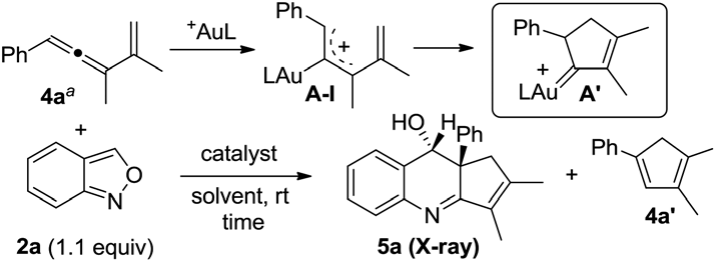						
Entry	Catalyst [mol%]	Solvent	*t* [h]	Yield^*b*^ [%]		
				**4a**	**5a**	**4a′**
1	IPrAuCl/AgSbF_6_ (5)	DCM	12	8	62	25
2	IPrAuCl/AgSbF_6_ (10)	DCM	3	—	85	12
3	(PhO)_3_PAuCl/AgSbF_6_ (10)	DCM	3	—	82	16
4	Ph_3_PAuCl/AgSbF_6_ (10)	DCM	4	—	55	36
5	LAuCl/AgSbF_6_ (10)^*c*^	DCM	3	—	40	52
6	IPrAuCl/AgOTf (10)	DCM	4	—	65	26
7	IPrAuCl/AgNTf_2_ (10)	DCM	4	—	71	20
8	AgSbF_6_ (10)	DCM	24	95	—	—
9	IPrAuCl/AgSbF_6_ (10)	DCE	5	—	70	24
10	IPrAuCl/AgSbF_6_ (10)	MeCN	12	—	20	65
11	IPrAuCl/AgSbF_6_ (10)	Dioxane	10	—	—	90

^*a*^[**4a**] = 0.05 M.

^*b*^Product yields are reported after purification from a silica column.

^*c*^L = P(*t*-Bu)_2_(*o*-biphenyl). IPr = 1,3-bis(diisopropylphenyl)imidazole-2-ylidene, DCE = 1,2-dichloroethane.

An initial test of IPrAuCl/AgSbF_6_ at a 5 mol% loading afforded a new azacyclic product **5a** and cyclopentadiene **4a′** in 62% and 25% yields, respectively (entry 1); the latter was derived from a 1,2-H shift of gold carbenes **A′** that was generated from cyclizations of gold-stabilized pentadienyl cation **A-I**. Notably, an increased gold loading (10 mol%) enhanced the yield of desired **5a** up to 85%. Other gold catalysts LAuCl/AgSbF_6_ (L = P(OPh)_3_, PPh_3_ and P(*t*-Bu)_2_(*o*-biphenyl)) gave **5a** in 40–82% yields with L = P(OPh)_3_ being the most effective (entries 3–5). For various silver salts as in IPrAuCl/AgX (X = OTf and NTf_2_), resulting **5a** was obtained in 65% and 71% yields, respectively (entries 6–7). AgNTf_2_ was entirely inactive (entry 8). IPrAuCl/AgSbF_6_ in various solvents gave **5a** in the following yields: DCE 70%, MeCN 20% and 1,4-dioxane 0 (entries 9–11). The molecular structure of compound **5a** was characterized with X-ray diffraction,^11^ showing an *anti*-configuration between the alcohol and phenyl groups.

Table 2 assesses the generality of these gold-catalyzed reactions using various vinylallenes **4b–4t** catalyzed with IPrAuCl/AgSbF_6_ (10 mol%) in DCM. All resulting products **5b–5t** assumed *anti*-configurations with the alcohol and R^1^ groups being mutually *trans*. We tested the reaction of trisubstituted vinylallenes **4b–4f** bearing R^1^ = 4-MePh, 4-OMePh, 4-ClPh, 4-CF_3_Ph and *n*-Bu, yielding desired **5b–5f** in 78–88% yields (entries 1–5). For species **4g** and **4h** bearing 3-phenyl substituents (X = OMe and Cl), their corresponding products **5g** and **5h** were obtained in 84% and 87% yields, respectively (entries 6 and 7). The reactions were extensible to other vinylallenes **4i–4k** bearing 2-naphthyl, 2-furan and 2-thiophene, further delivering desired products **5i–5k** in 82–84% yields (entries 8–10). We tested the reaction on vinylallene **4l** bearing distinct R^1^ = Me and R^2^ = Ph, which yielded compound **5l** with an anti-configuration in which the hydroxy and methyl groups are mutually *trans* (entry 11); this configuration was established by the ^1^H NOE effect. Additional alkyl-substituted vinylallenes **4m–4p** yielded desired **5m–5p** in satisfactory yields (80–85%, entries 12–15). Variations of the R^2^ group with an *n*-butyl group as in species **4q** gave expected product **5q** in 86% yield (entry 16). We prepared species **4r** bearing varied R^2^ = Ph and R^3^ = *n*-butyl, producing compound **5r** in 80% yield (entry 17). For 1,3-disubstituted vinylallenes **4s** and **4t** (R^3^ = H), their resulting compounds **5s** and **5t** were obtained in 82–83% yields (entries 18 and 19).

**Table 2:** Catalytic annulations with various alkenylallenes

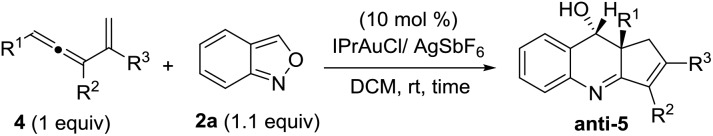
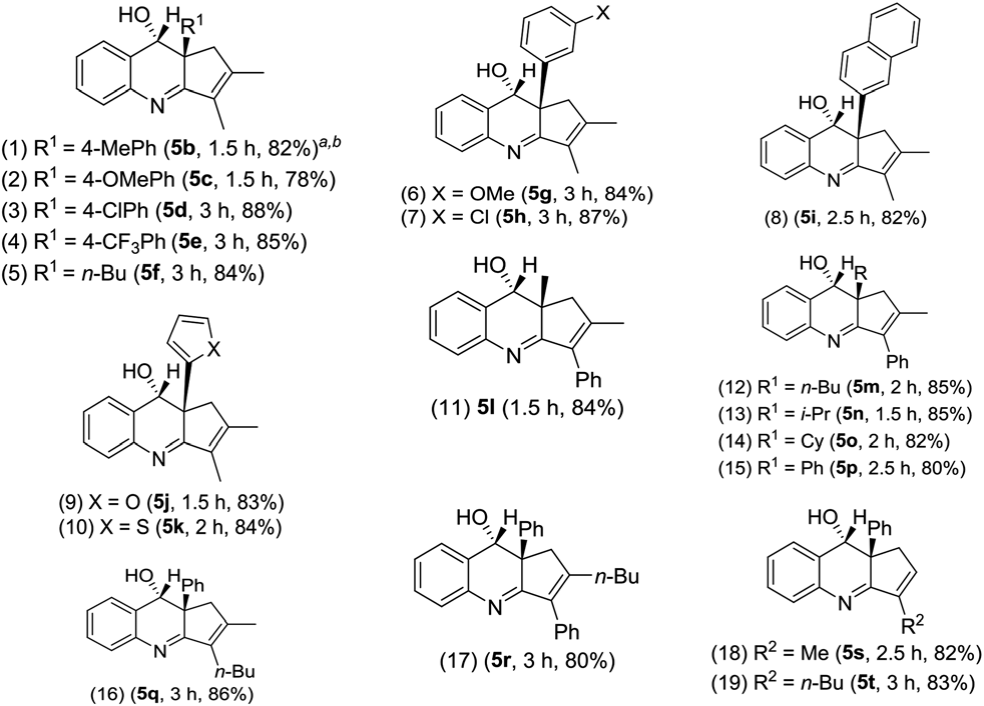

^*a*^[**4**] = 0.05 M.

^*b*^Product yields are reported after purification from a silica column.

We tested these new annulations on distinct substrates such as enynyl acetates **6a–6g**, bearing varied phenyl (R = 4-XC_6_H_4_, X = H, Cl, Br, Me, and OMe), 2-thienyl and isopropyl substituents; these enyne acetates can be catalyzed with the same gold catalyst to yield distinct α-alkylgold carbenes **A′** (see Table 3).^12^ To our pleasure, new alkylgold carbenes **A′**, generated from these enynyl acetates, were trapped efficiently with benzisoxazole **2a** to afford the desired (4 + 2)-annulation products **7a–7g** in satisfactory yields (61–74%), further manifesting the reaction generality (entries 1–7). For unsubstituted propargyl acetate **6h** (R = H), its reaction led to a 68% recovery of initial **6h** (entry 8). Even if the reaction is successful, a dehydration of compound **7h** would occur to give quinoline products. The molecular structure of compound **7a** (R = Ph) was confirmed with X-ray diffraction analysis that revealed an *anti*-configuration (Table 3).^11^

**Table 3:** Annulation reactions with enynyl acetates

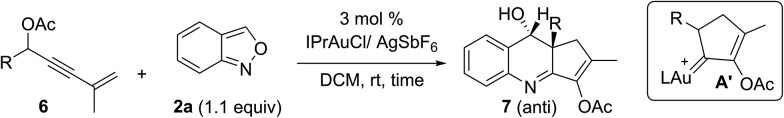
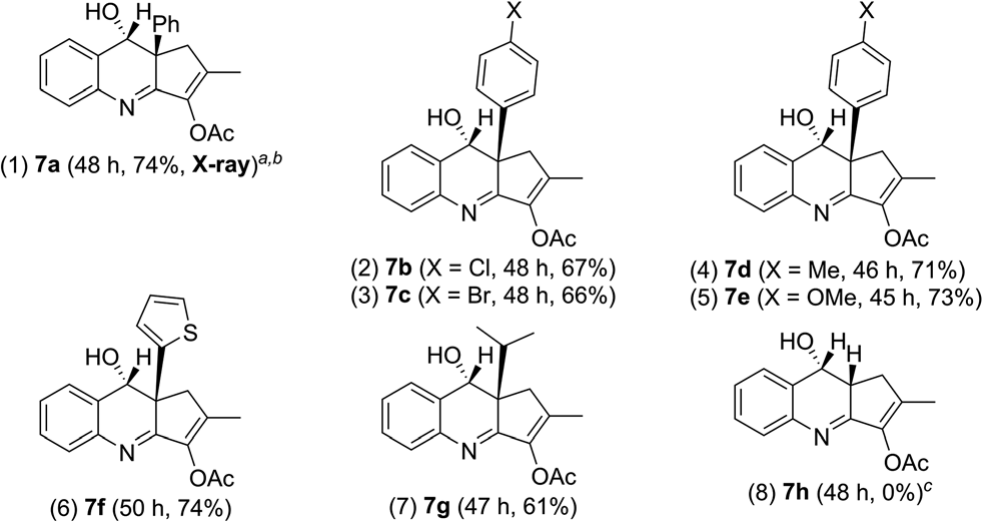

^*a*^ **6** = 0.05 M.

^*b*^Product yields are reported after purification from a silica column.

^*c*^A 68% recovery of initial **6h** is found in entry 8.

The scope of these catalytic reactions is further expanded with various applicable benzisoxazoles **2b–2j** substituted with the C(3), C(5) and C(6) carbons. Other C(5)-substituted benzisoxazoles **2b–2f** (R^1^ = Me, OMe, Br, Cl, and –OCO_2_Et) maintained high efficiencies to deliver *anti*-configured products **8b–8f** in 80–90% yields (entries 1–5). High reaction efficiencies were maintained also for C(6)-substituted benzisoxazoles **2g–2i** that furnished products **8g–8i** in 86–92% yields (entries 6–8). A final applicable reaction with a C(3)-substituted benzisoxazole **2j** enabled the production of a tertiary alcohol **8j**, reflecting the reaction feasibility (entry 9). ^1^H NOE spectra were recorded to verify the stereochemistry of compound **8j** (Table 4).

**Table 4:** Catalytic annulations with various benzisoxazoles

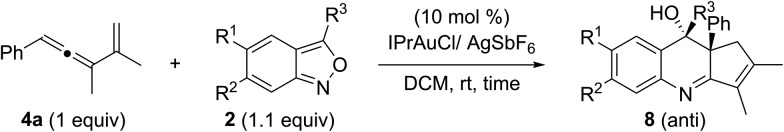
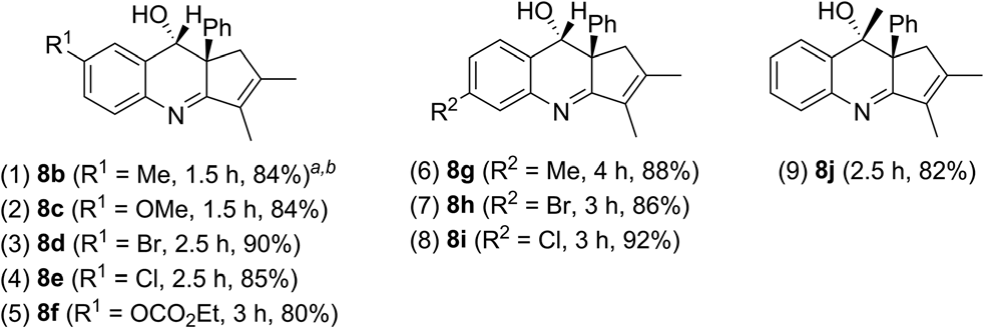

^*a*^ **4a** = 0.05.

^*b*^Product yields are reported after purification from a silica column.

Gold-catalyzed reactions of 3,5-dimethylisoxazole **2a′** with vinylallenes **4a** and **4u** delivered 2-aminocyclopentadienes **9a** and **9b** in 72% and 64% yields, respectively (eqn (6)).5a,^13^,^14^ The molecular structure of compound **9b** was characterized with X-ray diffraction.^11^ Cyclizations of compounds **9a** and **9b** with a gold catalyst were unsuccessful because of the two different forms of the enol imines (eqn (6)). To rationalize the origin of the stereoselectivity, compound **5a** was treated with Zn(OTf)_2_ (20 mol%) in refluxing DCE to examine the hydroxyl epimerization that turned out to be slow. An equilibrium, *anti*/*syn* = 4 : 1, was attained for species **5a** after reflux in DCE for 48 h (eqn (7)).
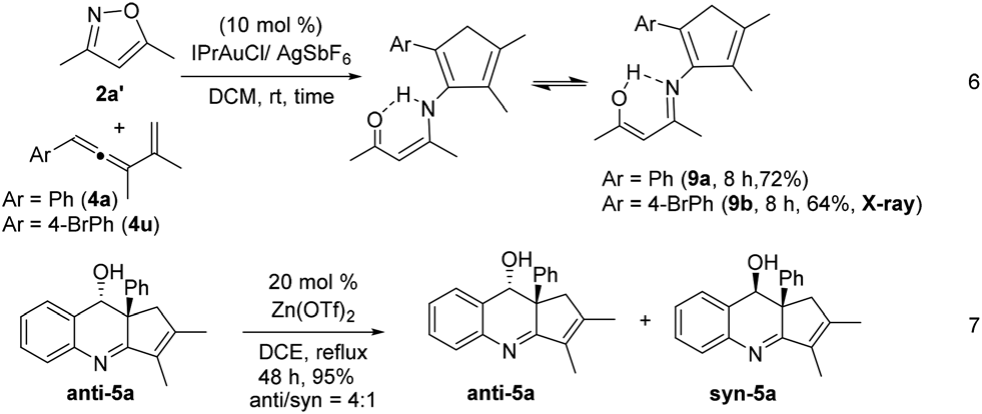


**Scheme 1:**
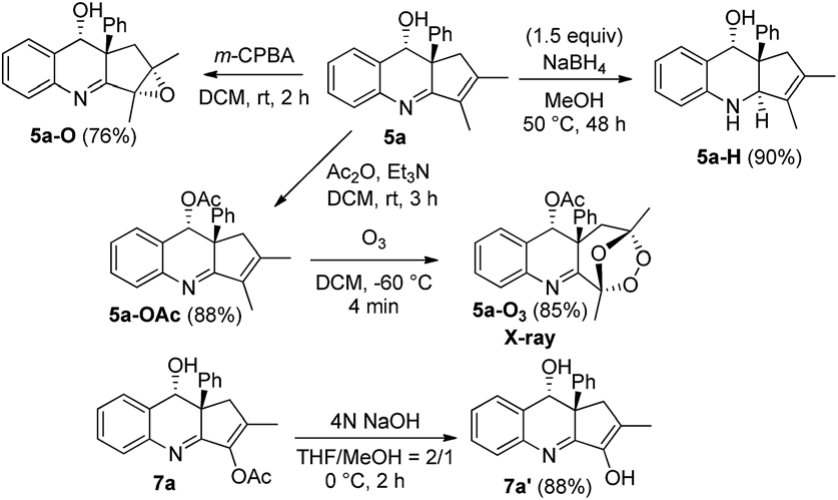
Chemical functionalizations.

Scheme 1 shows the stereoselective functionalizations of *anti*-**5a***via* NaBH_4_ reductions and *m*-CPBA oxidations, respectively yielding compounds **5a-H** and **5a-O** as single diastereomeric products. The stereochemistries of compounds **5a-H** and **5a-O** were established with ^1^H NOE spectra. Likewise, the acetate species **7a** was readily removed under basic conditions, yielding the enol form **7a′** as shown by its NMR in CD_3_COCD_3_ and CDCl_3_. We also studied an O_3_-induced oxidative cleavage of the acetate derivative **5a-OAc** to cleave the olefin group, yielding the peroxide **5a-O_3_** in 85% yield. The molecular structure of species **5a-O_3_** has been characterized by X-ray diffraction.^11^

**Scheme 2:**
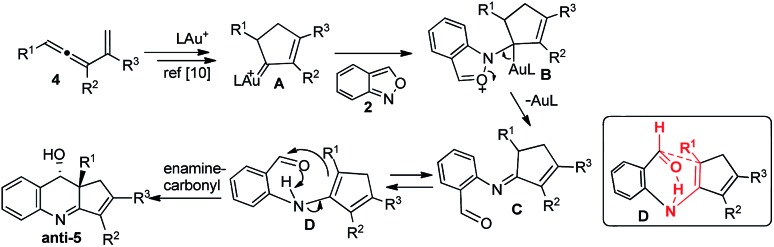
A plausible reaction mechanism.

As depicted in Scheme 2, we postulate an initial formation of imines between alkylgold carbene **A** and benzisoxazole, yielding 2-iminoyl benzaldehyde **C**. This hypothesis is supported by our observation of 3,5-dimethylisoxazole, depicted in eqn (6). A tautomerization of imine species **C** is expected to form enamines **D** bearing an NH···O=C hydrogen bond. We believe that this enamine form, unlike other enamine-carbonyl couplings,^15^ is stabilized with the NH···O=C bond to enable a concerted process, analogous to the well-known carbonyl–ene reactions. A boat-like conformation **D** is favorable to yield *anti*-**5** stereoselectively.

## Conclusions

This work reports novel gold-catalyzed (4 + 2)-annulations between alkylgold carbenes and benzisoxazoles **2** to form 3,4-dihydroquinoline derivatives. Gold carbenes in cyclic and acyclic forms are both applicable. In this reaction sequence, the gold complex catalyzes an initial formation of imines between alkylgold carbenes^13^,^14^ and benzisoxazoles; the resulting intermediates bear an enamine moiety that is bound to an aldehyde *via* a hydrogen bond to induce a carbonyl-enamine reaction. Control experiments with 3,5-dimethylisoxazoles supported this postulated mechanism. This new synthetic design involving α-alkyl metal carbenes of **D**/**D** types will attract growing interest because of its distinct utility.

## Conflicts of interest

There are no conflicts to declare.

## Supplementary Material

Supplementary information

Crystal structure data
