# Supplementary material for: Gold-catalyzed (4 + 2)-annulations between α-alkyl alkenylgold carbenes and benzisoxazoles with reactive alkyl groups
Source: Chem Sci. 2018 Apr 23;9(19):4488–92. doi: 10.1039/c8sc00986d (PMC6049024; doi:10.1039/c8sc00986d)

Electronic Supplementary Material (ESI) for Chemical Science.
This journal is © The Royal Society of Chemistry 2018

Supporting Information

Gold-catalyzed (4+2)-Annulations between α-Alkyl alkenylgold Carbenes and Benzisoxazoles with Reactive Alkyl Groups

Bhanudas Dattatray Mokar,‡ Prakash D. Jadhav,‡ Y. B. Pandit and Rai-Shung Liu*

Frontier Research Centers for Materials Science and Technology and Department of Chemistry, National Tsing-Hua University, Hsinchu, Taiwan, ROC
---E-mail: rsliu@mx.nthu.edu.tw

Contents:

(1) Representative synthetic procedures --- S2
(2) Standard procedures for catalytic operation --- S5
(3) Synthetic procedures for chemical functionalizations ---------------------------------- S6
(4) Spectral data of key compounds -- S8
(5) X-ray crystallographic data of compounds (5a, 5a-O_3, 7a and 9b) ------------------ S43
(6) ^{1}H, ^{13}C and ^{1}H NOE of key compounds -- S87

(1) Representative synthetic procedures

(A) General experimental:

Unless otherwise noted, all reactions were carried out under a N_2 atmosphere in reaction tube. Tetrahydrofuran was dried with sodium benzophenone and distilled before use. Dichloromethane were dried over CaH_2 and distilled before use. The triethylamine (Et_3N) were stored over 4 Å molecular sieves prior to use. Reagents were purchased from commercial sources and used without purification, unless otherwise stated. Reactions were magnetically stirred and monitored by thin layer chromatography carried out on 0.25 mm E. Merck silica gel plate (60f- 254) using UV light as visualizing agents and/or potassium permanganate ($KMnO_4$). ^{1}H NMR and ^{13}C NMR spectra were recorded on a Bruker 400, Varian 400, 500, and 600 MHz spectrometers using chloroform-*d* ($CDCl_3$) and *d*-acetone $(CD_3)_2CO$ as the internal standard. Chemical shifts are reported in parts per million (ppm). Multiplicities are indicated by s (singlet), d (doublet), t (triplet), q (quartet), and m (multiplet). Coupling constants *J* are reported in Hertz (Hz). The substrates **1a** and **1b** were prepared by using literature procedure.[S1] The vinylallene substrates[S2-S4] **4a-4t** and benzisoxazole substrates[S5] **2a-2j** were prepared according to literature procedures.

(S1) Z. Liu, Q. Li, P. Liao and X. Bi, *Chem. Eur. J.*, 2017, **23**, 4756.

(S2) S. Bhunia and R.-S. Liu, *J. Am. Chem. Soc.*, 2008, **130**, 16488.

(S3) T. M. Macdonald and D. R. Reagan, *J. Org. Chem.*, 1980, **45**, 4740.

(S4) R. Chaudhari, H.-Y. Liao and R.-S. Liu, *Chem. Eur. J.*, 2009, **15**, 8895.

(S5) (a) J. Chauhan and S. Fletcher, *Tetrahedron Lett*., 2012, **53**, 4951; (b) H. Jin, L. Huang, J. Xie, M. Rudolph, F. Rominger and A. S. K. Hashmi, *Angew. Chem. Int. Ed*., 2016, **55**, 794; (c) H. Jin, B. Tian, X. Song, J. Xie, M. Rudolph, F. Rominger and A. S. K. Hashmi, *Angew. Chem. Int. Ed*., 2016, **55**, 12688; (d) R. L. Sahani and R.-S. Liu, *Angew. Chem. Int. Ed*., 2017, **56**, 12736.

(B) Synthesis of the substrate (3,4-dimethylpenta-1,2,4-trien-1-yl)benzene (4a):

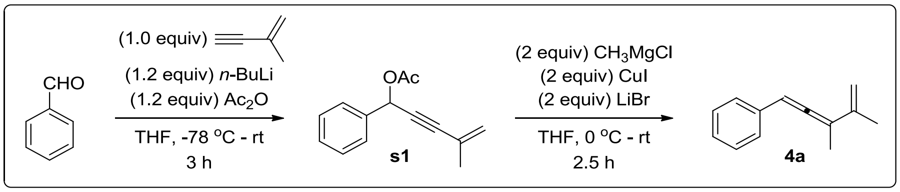

(a) Synthesis of 4-methyl-1-phenylpent-4-en-2-yn-1-yl acetate (s1):

To a THF (10 mL) solution of 2-methylbut-1-en-3-yne (1.0 g, 15.13 mmol) was added *n*-BuLi (2.5 M in hexane, 7.3 mL, 18.15 mmol) at -78 ^{o}C and stirred at this temperature for 0.5 h. The THF (10 mL) solution of PhCHO (1.61 g, 15.13 mmol) was added and the reaction mixture was stirred at rt for additional 1.5 h. The resulting mixture was quenched by acetic anhydride (1.86 g, 18.15 mmol) and stirred for next 1 h. After completion of reaction, saturated NH_4Cl was added and extracted with diethyl ether (2 x 50 mL). The combined organic layer were dried over $MgSO_4$ and concentrated under reduced pressure. The cude reaction mass was purified by silica column eluting with hexane/ethyl acetate (5:1) to afford 4-methyl-1-phenylpent-4-en-2-yn-1-ol **s1** (3.1 g, 14.48 mmol, 96%) as pale yellow oil.

(b) Synthesis of (3,4-dimethylpenta-1,2,4-trien-1-yl)benzene (4a):

To a dry 250-mL flask were added LiBr (0.81 g, 9.33 mmol) and CuI (1.78 g, 9.33 mmol), evacuated and backfilled with N_2 balloon before dried THF (25 mL) was added. The reaction mixture was cooled to 0 ^{o}C, CH_3MgCl (3.0 M in THF, 2.1 mL, 9.33 mmol) was added. The resulting solution was stirred reaction mixture at 0 ^{o}C for additional 0.5 h. To this solution was added propargylic acetate (**s1**) (1 g, 4.67 mmol), and the mixture was stirred for 2 h. The resulting mixture was quenched by a saturated NH_4Cl, filtrated, and extracted with diethyl ether (2 x 50 mL). The extract was dried over $MgSO_4$, concentrated in vacuo and purified by a silica column to afford (3,4-dimethylpenta-1,2,4-trien-1-yl)benzene **4a** (0.600 g, 3.52 mmol, 76%) as colorless oil.

Substrates **4b**-**4k** and **4q** were synthesized using same as above procedure.

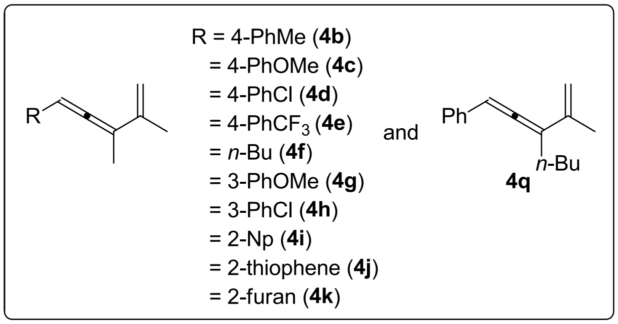

(C) Synthesis of the substrate (4-methylpenta-1,2,4-triene-1,3-diyl)dibenzene (4p):

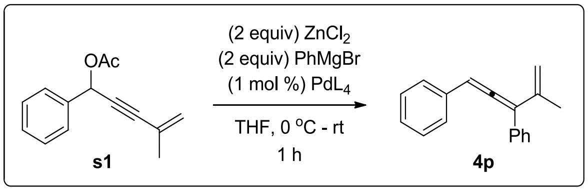

Synthesis of (4-methylpenta-1,2,4-triene-1,3-diyl)dibenzene (4p):

To a dry 250-mL flask was added $ZnCl_2$ (1.27 g, 9.93 mmol), dried in vacuo and refilled with N_2 balloon. To this flask were added dry THF (25 mL) and then PhMgBr (1.69 g, 9.93 mmol); the mixture was stirred at 25 ^{o}C for 0.5 h. The [Pd(PPh_3)$_4$] (54.0 mg, 0.047 mmol) and compound **s1** (1 g, 4.67 mmol) were added to this reaction mixture; the resulting solution was stirred for 1 h before quenching with water. The organic layer was extracted with diethyl ether (2 x 25 mL), dried over $MgSO_4$ and concentrated under reduced pressure. The residue was eluted through a silica column to afford (4-methylpenta-1,2,4-triene-1,3-diyl)dibenzene **4p** (0.800 g, 3.44 mmol, 74%) as colorless liquid.

Substrates **4l-4o** and **4r** were synthesized using same as above procedure.

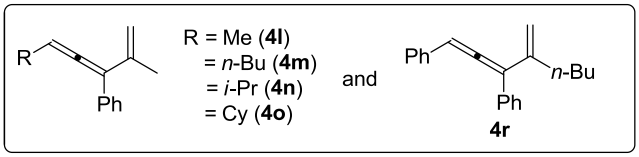

(D) Synthesis of the substrate (3-methylpenta-1,2,4-trien-1-yl)benzene (4s):

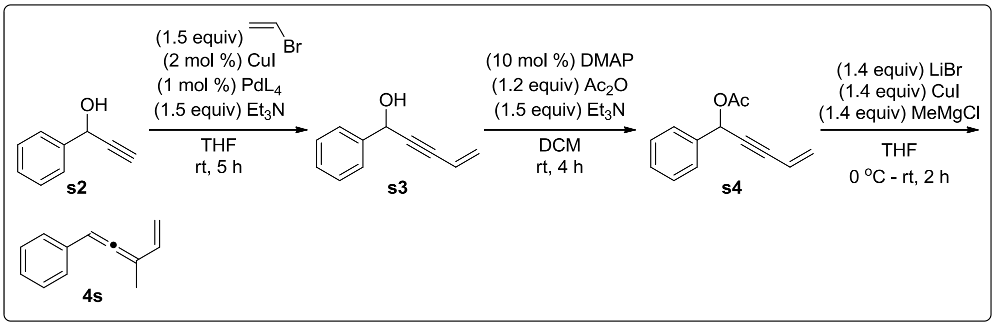

(a) Synthesis of 1-phenylpent-4-en-2-yn-1-ol (s3):

Copper (I) iodide (0.029 g, 0.15 mmol) and tetrakis(triphenylphosphin)palladium (0.087 g, 0.076 mmol) were dissolved in anhydrous and degassed Et_3N (3.2 mL, 22.7 mmol), and the mixture was cooled to 0 °C. To this solution was added alkyne **s2** (1 g, 7.57 mmol) and

vinyl bromide (1 M in THF, 1.21 g, 11.35 mmol), and the mixture was stirred at room temperature until a complete consumption of starting material. The reaction solution was filtered through a celite pad, concentrated and eluted through a silica column to give 1-phenylpent-4-en-2-yn-1-ol **s3** (0.550 g, 2.75 mmol, 46%) as colorless oil.

(b) Synthesis of 1-phenylpent-4-en-2-yn-1-yl acetate (s4):

To a DCM solution of 1-phenylpent-4-en-2-yn-1-ol (1.0 g, 6.33 mmol) were added DMAP (77 mg, 0.63 mmol), Et_3N (0.96 g, 9.49 mmol) and the mixtures were stirred at 0 ^oC for 15 min. To this solution was added Ac_2O (0.78 g, 7.59 mmol), and the mixture was slowly warmed to room temperature for a stirring of 4 h. The solution was quenched by water and extracted with DCM (2 x 25 mL). The combined organic layer was dried over $MgSO_4$, concentrated in vacuo, and further purified by a silica column to afford 1-phenylpent-4-en-2-yn-1-yl acetate **s4** (1.19 g, 7.52 mmol, 94%) as pale yellow oil.

(c) Synthesis of (3-methylpenta-1,2,4-trien-1-yl)benzene (4s):

The substarte **1s** was synthesized using similar synthetic procedure of substrate **4a** from **s1**.

Substrate **4t** was prepared using same as above procedure of **4s**.

(2) Standard procedures for catalytic operation.

(a) Standard procedure for the synthesis of (9*S*,9a*S*)-2,3-dimethyl-9a-phenyl-9,9a-dihydro-1*H*-cyclopenta[*b*]quinolin-9-ol (5a):

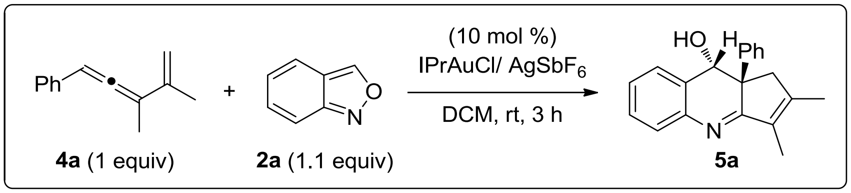

A catalytic tube was charged with IPrAuCl (0.036 g, 0.059 mmol) and $AgNTf_2$ (0.020 g, 0.059 mmol), and to this mixture was added dry DCM (0.5 mL). The resulting mixture was stirred at room temperature for 10 min. To this mixture was added a dry DCM solution (0.5 mL) of benzisoxazole (**2a**) (0.077 g, 0.646 mmol) followed by dropwise addition of (3,4-dimethylpenta-1,2,4-trien-1-yl)benzene (**4a**) (0.100 g, 0.587 mmol) in 1 mL DCM. After stirring at room temperature for 3 h, the reaction mixture was filtered over a short celite bed, concentrated, and eluted through a silica column (EA/hexane = 30/70) to give the desired

(9*S*,9a*S*)-2,3-dimethyl-9a-phenyl-9,9a-dihydro-1*H*-cyclopenta[*b*]quinolin-9-ol (**5a**) (0.144 g, 0.498 mmol, 85%) as white solid.

The compounds **3**, **7**, **8**, and **9** were synthesized using same as above catalytic procedure.

(b) Standard procedure for the synthesis of (9*R*,9a*S*)-2,3-dimethyl-9a-phenyl-9,9a-dihydro-1*H*-cyclopenta[*b*]quinolin-9-ol (syn-5a):

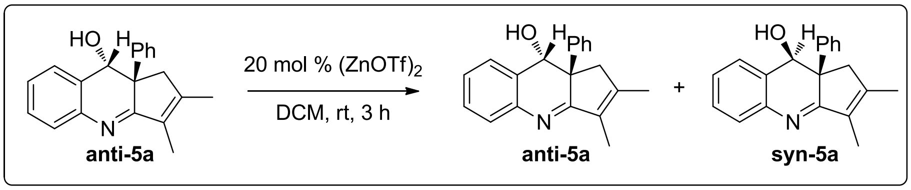

To a DCM (4 mL) solution of (9*S*,9a*S*)-2,3-dimethyl-9a-phenyl-9,9a-dihydro-1*H*-cyclopenta[*b*]quinolin-9-ol (**5a**) (0.200 g, 0.69 mmol) was added $Zn(OTf)_2$ (0.050 g, 0.13 mmol), and refluxed for 48 hours. The reaction mixture was then filtered over a short celite bed, concentrated and eluted through a silica column (EA/hexane = 2/5) to give isomers dr ratio 4:1 the (**syn-5a**, 0.048 g, 0.13 mmol, 19%) and (**anti-5a,** 0.152 g, 0.52 mmol, 76%).

(3) Synthetic procedures for chemical functionalizations:

(i) Synthesis of (3a*R*,9*R*,9a*S*)-2,3-dimethyl-9a-phenyl-3a,4,9,9a-tetrahydro-1*H*-cyclopenta[*b*]quinolin-9-ol (5a-H):

To a CH_3OH (7 mL) solution of corresponding imine (9*S*,9a*S*)-2,3-dimethyl-9a-phenyl-9,9a-dihydro-1*H*-cyclopenta[*b*]quinolin-9-ol (**5a**) (0.100 g, 0.34 mmol) was added $NaBH_4$ (0.019 g, 0.50 mmol) and the resulting mixture was stirred at the 50 °C for 48 h. After completion, the reaction was quenched with saturated ammonium chloride solution and extracted with dichloromethane (3 x 10 mL). The combined organic layers were washed with brine, dried over anhydrous $MgSO_4$, and concentrated. The residue was purified via column chromatography (30% EtOAc/hexanes) to give **5a-H** (0.090 g, 0.30 mmol, 90%) as colorless liquid.

(ii) Synthesis of (1a*R*,7*S*,7a*S*,8a*S*)-1a,8a-dimethyl-7a-phenyl-7,7a,8,8a-tetrahydro-1a*H*-oxireno[2',3':4,5]cyclopenta[1,2-*b*]quinolin-7-ol (5a-O):

To a DCM (5 mL) solution of the (9*S*,9a*S*)-2,3-dimethyl-9a-phenyl-9,9a-dihydro-1*H*-

cyclopenta[*b*]quinolin-9-ol (**5a**) (0.200 g, 0.69 mmol) was added *m*-CPBA (0.119 g, 0.69 mmol) at 0 °C. The reaction mixture was stirred at room temperature for 24 hours. The reaction mixture was then quenched with saturated $NaHCO_3$ solution and extracted with DCM (2 x 10 mL). The combined organic layer were washed with brine, dried over $MgSO_4$ and concentrated under reduced pressure. The residue was purified via column chromatography (25% EtOAc/Hexanes) to afford compound **5a-O** (0.160 g, 0.52 mmol, 76%) as Colorless liquid.

(iii) Synthesis of (1*R*,4*S*,5a*S*,6*S*)-1,4-dimethyl-5a-phenyl-4,5,5a,6-tetrahydro-1*H*-1,4-epoxy[1,2]dioxepino[4,5-*b*]quinolin-6-yl acetate (5a-O$_3$):

(a) Synthesis of (9*S*,9a*S*)-2,3-dimethyl-9a-phenyl-9,9a-dihydro-1*H*-cyclopenta[*b*]quinolin-9-yl acetate (5a-OAc):

To a DCM (10 mL) solution of (9S,9a*S*)-2,3-dimethyl-9a-phenyl-9,9a-dihydro-1*H*-cyclopenta[*b*]quinolin-9-ol (**5a**) (0.500 g, 1.72 mmol) was added Et_3N (0.364 mL, 2.59 mmol) and acetic anhydride (0.196 mL, 2.06 mmol) at 0 °C. The reaction mixture was stirred at room temperature for 3 h. The saturated $NaHCO_3$ solution was added in to the reaction mixture and extracted with DCM (2 x 20 mL). The combined organic layer was washed with brine, dried over $MgSO_4$, and concentrated under reduced pressure. The residue was purified by column chromatography (20% EtOAc/hexanes) to give **5a-OAc** (0.503 g, 1.52 mmol, 80%) as white solid.

(b) Synthesis of (1*R*,4*S*,5a*S*,6*S*)-1,4-dimethyl-5a-phenyl-4,5,5a,6-tetrahydro-1*H*-1,4-epoxy[1,2]dioxepino[4,5-*b*]quinolin-6-yl acetate (5a-O$_3$):

To a DCM (10 mL) solution of (9*S*,9a*S*)-2,3-dimethyl-9a-phenyl-9,9a-dihydro-1*H*-cyclopenta[*b*]quinolin-9-yl acetate **(5a-OAc)** (0.333 g, 1 mmol) at -60 °C, a stream of O_3/O_2 (~ 1 mmol/min of O_3) was introduced through a disposable pipet for a period with the amount of alkene 4 min. Once complete, the reaction was sparged with O_2 and then N_2. The crude reaction mixture was concentrated and purified by flash chromatography with (25% EtOAc/Hexanes) to furnish the **5a-O$_3$** (0.323 g, 0.85 mmol, 85%) as white solid.

(iv) Synthesis of (9*S*,9a*S*)-2-methyl-9a-phenyl-9,9a-dihydro-1*H*-cyclopenta[*b*]quinoline-3,9-diol (7a'):

To a THF (10 mL) and MeOH (5 mL) solution of (9*S*,9a*S*)-9-hydroxy-2-methyl-9a-phenyl-

9,9a-dihydro-1*H*-cyclopenta[*b*]quinolin-3-yl acetate (**7a**) (0.170 g, 0.50 mmol) was added 4M aqueous NaOH (4 mL) solution at 0 °C. The reaction mixture was stirred at this temperature for 2 h. The solution was concentrated, then diluted with DCM and quenched with 1N HCl. The aqueous layer was extracted with DCM (2 x 10 mL). The combined organic layers were washed with saturated $NaHCO_3$, dried over $MgSO_4$, and concentrated. The residue was purified by column chromatography (25% EtOAc/hexanes) to give compound **7a'** (0.113 g, 0.38 mmol, 80%) as brown solid.

(4) Spectral data of key compounds

Spectral data for (2,3-diethylcycloprop-2-en-1-yl)benzene (1a):

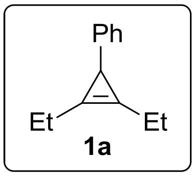

Colorless oil (0.079 g, 0.459 mmol, 70%); ^{1}H NMR (400 MHz, $CDCl_3$): δ 7.26 ~ 7.22 (m, 2H), 7.12 ~ 7.07 (m, 3H), 2.50 (s, 1H), 2.48 ~ 2.44 (m, 4H), 1.20 ~ 1.60 (m, 6H); ^{13}C NMR (100 MHz, $CDCl_3$): δ 148.1, 127.9, 125.3, 124.4, 111.3, 25.2, 17.9, 12.4.

Spectral data for (*E*)-4-benzylidenehexan-3-one (1a-O):

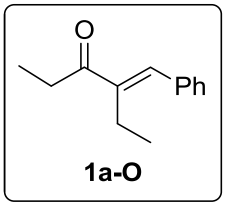

Colorless oil (0.021 g, 0.11 mmol, 19%); ^{1}H NMR (400 MHz, $CDCl_3$): δ 7.44 (s, 1H), 7.41 ~ 7.30 (m, 5H), 2.81 (q, *J* = 7.3 Hz, 2H), 2.53 (q, *J* = 7.4 Hz, 2H), 1.49 (t, *J* = 7.3 Hz, 3H), 1.08 (t, *J* = 7.5 Hz, 3H); ^{13}C NMR (100 MHz, $CDCl_3$): δ 202.9, 143.6, 137.8, 135.9, 129.2, 128.5, 128.4, 31.0, 19.8, 13.8, 8.8; HRMS (ESI+, m/z) calcd for $C_{13}H_{17}O$ [M+H]: 189.1279, found: 189.1279.

Spectral data for (3-butylcycloprop-2-ene-1,2-diyl)dibenzene (1b):

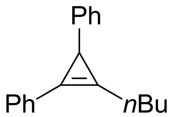

1b

Colorless oil (0.124 g, 0.499 mmol, 76%); ^{1}H NMR (400 MHz, $CDCl_3$): δ 7.45 (d, *J* = 6.8 Hz, 2H), 7.38 ~ 7.32 (m, 2H), 7.28 ~ 7.20 (m, 3H), 7.15 ~ 7.09 (m, 3H), 2.86 (s, 1H), 2.73 ~ 2.63 (m, 2H), 1.72 (p, *J* = 7.6 Hz, 2H), 1.49 ~ 1.41 (m, 2H), 0.94 (t, *J* = 7.2 Hz, 3H); ^{13}C NMR (100 MHz, $CDCl_3$): δ 146.1, 129.2, 128.6, 128.0, 127.8, 126.5, 125.6, 125.0, 116.6, 108.5, 30.0, 25.0, 24.9, 22.6, 13.8; HRMS (ESI+, m/z) calcd for $C_{19}H_{21}Na$ [M+H]: 272.1541, found: 272.1534.

Spectral data for (*E*)-1,2-diphenylhept-1-en-3-one (1b-O):

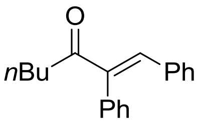

1b-O

Colorless oil (0.018 g, 0.068 mmol, 17%); ^{1}H NMR (400 MHz, $CDCl_3$): δ 7.60 (s, 1H), 7.41 ~ 7.33 (m, 3H), 7.20 ~ 7.11 (m, 5H), 7.00 (d, *J* = 8.0 Hz, 2H), 2.54 (t, *J* = 7.2 Hz, 2H), 1.62 ~ 1.55 (m, 2H), 1.31 ~ 1.24 (m, 2H), 0.86 (t, *J* = 7.4 Hz, 3H); ^{13}C NMR (100 MHz, $CDCl_3$): δ 201.8, 140.8, 137.7, 137.1, 134.8, 130.8, 129.6, 129.0, 129.0, 128.2, 127.8, 39.7, 26.5, 22.3, 13.9; HRMS (ESI+, m/z) calcd for $C_{19}H_{21}O$ [M+H]: 265.1592, found: 265.1595.

Spectral data for (*E*)-3-methyl-2-(1-phenylbut-1-en-2-yl)quinoline (3a):

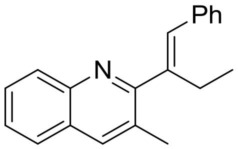

3a

Pale yellow viscous oil (0.119 g, 0.435 mmol, 75%); ^{1}H NMR (400 MHz, $CDCl_3$): δ 8.10 (d, *J* = 8.4 Hz, 1H), 7.97 (s, 1H), 7.74 (d, *J* = 8.0 Hz, 1H), 7.63 (td, *J* = 8.0, 1.2 Hz, 1H), 7.48 (td, *J* = 8.0, 0.8 Hz, 1H), 7.42 ~ 7.35 (m, 4H), 7.27 (d, *J* = 6.8 Hz, 1H), 6.51 (s, 1H), 2.89 (q, *J* = 7.6 Hz, 2H), 2.54 (s, 3H), 1.01 (t, *J* = 7.6 Hz, 3H); ^{13}C NMR (100 MHz, $CDCl_3$): δ 162.7, 146.3, 143.7, 137.4, 136.5, 130.0, 129.5, 129.0, 128.8, 128.6, 128.2, 127.4, 126.8, 126.7,

126.2, 25.1, 20.1, 12.7; HRMS (ESI+, m/z) calcd for $C_{20}H_{20}N$ [M+H]: 274.1596, found: 274.1607.

Spectral data for (*E*)-2-(1,2-diphenylvinyl)-3-propylquinoline (3b):

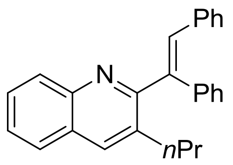

3b

Pale yellow viscous oil (0.102 g, 0.292 mmol, 72%); ^{1}H NMR (400 MHz, $CDCl_3$): δ 8.17 (d, *J* = 8.4 Hz, 1H), 7.89 (s, 1H), 7.75 (d, *J* = 8.4 Hz, 1H), 7.66 (td, *J* = 8.4, 1.2 Hz, 1H), 7.50 (td, *J* = 8.0, 1.2 Hz, 1H), 7.27 ~ 7.25 (m, 3H), 7.23 ~ 7.18 (m, 7H), 6.88 (s, 1H), 2.49 (t, *J* = 8.0 Hz, 2H), 1.51 ~ 1.41 (m, 2H), 0.80 (t, *J* = 7.4 Hz, 3H); ^{13}C NMR (100 MHz, $CDCl_3$): δ 161.9, 146.3, 142.0, 138.4, 136.7, 136.1, 134.5, 131.8, 129.8, 129.7, 129.3, 128.7, 128.3, 127.9, 127.8, 127.5, 127.2, 126.9, 126.3, 34.7, 23.5, 14.0; HRMS (ESI+, m/z) calcd for $C_{26}H_{24}N$ [M+H]: 350.1909, found: 350.1920.

Spectral data for (3,4-dimethylpenta-1,2,4-trien-1-yl)benzene (4a):

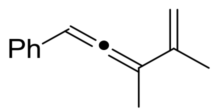

4a

Pale yellow oil (0.600 g, 3.52 mmol, 76%); ^{1}H NMR (600 MHz, $CDCl_3$): δ 7.30 ~ 7.24 (m, 4H), 7.19 ~ 7.16 (m, 1H), 6.28 (s, 1H), 5.00 (s, 1H), 4.96 (d, *J* = 1.4 Hz, 1H), 1.97 (d, *J* = 2.6 Hz, 3H), 1.85 (s, 3H); ^{13}C NMR (150 MHz, $CDCl_3$): δ 207.6, 140.5, 134.9, 128.6, 126.8, 126.7, 111.5, 106.5, 95.4, 21.5, 16.3; HRMS (EI+, m/z) calcd for $C_{13}H_{14}$ [M^+]: 170.1096, found: 170.1091.

Spectral data for 1-(3,4-dimethylpenta-1,2,4-trien-1-yl)-4-methylbenzene (4b):

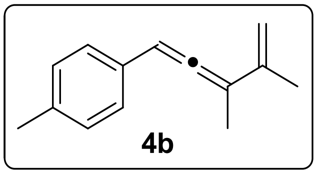

Pale yellow oil (0.282 g, 1.53 mmol, 70%); ^{1}H NMR (600 MHz, $CDCl_3$): δ 7.16 (d, *J* = 8.1 Hz, 2H), 7.09 (d, *J* = 7.9 Hz, 2H), 6.25 (s, 1H), 4.99 (s, 1H), 4.95 (d, *J* = 1.4 Hz, 1H), 2.31 (s, 3H), 1.96 (d, *J* = 2.6 Hz, 3H), 1.85 (s, 3H); ^{13}C NMR (150 MHz, $CDCl_3$): δ 207.3, 140.7, 136.5, 131.9, 129.3, 126.6, 111.3, 106.4, 95.2, 21.4, 21.1, 16.3; HRMS (EI+, m/z) calcd for $C_{14}H_{16}$ [M^+]: 184.1253, found: 184.1253.

Spectral data for 1-(3,4-dimethylpenta-1,2,4-trien-1-yl)-4-methoxybenzene (4c):

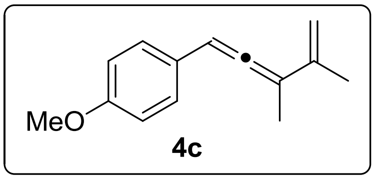

Pale yellow oil (0.260 g, 1.30 mmol, 63%); ^{1}H NMR (600 MHz, $CDCl_3$): δ 7.20 ~ 7.17 (m, 2H), 6.84 ~ 6.82 (m, 2H), 6.24 (s, 1H), 4.98 (s, 1H), 4.95 (d, *J* = 1.3 Hz, 1H), 3.78 (s, 3H), 1.95 (d, *J* = 2.7 Hz, 3H), 1.84 (s, 3H); ^{13}C NMR (150 MHz, $CDCl_3$): δ 207.0, 158.7, 140.9, 127.8, 127.2, 114.1, 111.2, 106.4, 94.7, 55.3, 21.5, 16.5; HRMS (ESI, m/z) calcd for $C_{14}H_{17}O$ [M+H]: 201.1279, found: 201.1271.

Spectral data for 1-chloro-4-(3,4-dimethylpenta-1,2,4-trien-1-yl)benzene (4d):

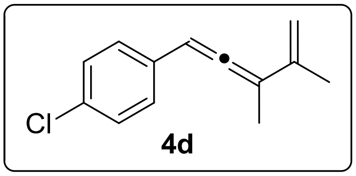

Pale yellow oil (0.340 g, 1.66 mmol, 82%); ^{1}H NMR (600 MHz, $CDCl_3$): δ 7.26 ~ 7.25 (m, 2H), 7.20 ~ 7.18 (m, 2H), 6.25 (s, 1H), 5.03 (s, 1H), 4.99 (d, *J* = 1.4 Hz, 1H), 1.98 (d, *J* = 2.6 Hz, 3H), 1.85 (s, 3H); ^{13}C NMR (150 MHz, $CDCl_3$): δ 207.7, 140.2, 133.5, 132.4, 128.7, 127.9, 111.9, 107.0, 94.5, 21.4, 16.2; HRMS (EI+, m/z) calcd for $C_{13}H_{13}Cl$ [M^+]: 204.0706, found: 204.0705.

Spectral data for 1-(3,4-dimethylpenta-1,2,4-trien-1-yl)-4-(trifluoromethyl)benzene (4e):

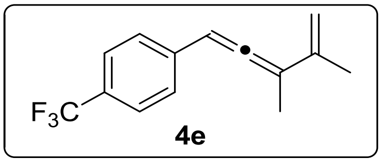

Pale yellow oil (0.360 g, 1.51 mmol, 85%); ^{1}H NMR (600 MHz, CDCl$_3$): δ 7.52 (d, *J* = 8.3 Hz, 2H), 7.39 (d, *J* = 8.3 Hz, 2H), 6.31 (s, 1H), 5.05 (s, 1H), 5.00 (d, *J* = 1.3 Hz, 1H), 1.99 (d, *J* = 2.6 Hz, 3H), 1.89 (s, 3H); ^{13}C NMR (150 MHz, CDCl$_3$): δ 208.6, 139.9, 139.0, 129.9 ($J_{C\text{-}F}$ = 283.8), 128.2 ($J_{C\text{-}F}$ = 32.0), 126.8, 125.6, 125.5, 112.3, 107.2, 94.7, 29.7, 21.4, 16.1; HRMS (EI+, m/z) calcd for $C_{14}H_{13}F_3$ [M$^+$]: 238.0969, found: 238.0966.

Spectral data for 2,3-dimethylnona-1,3,4-triene (4f):

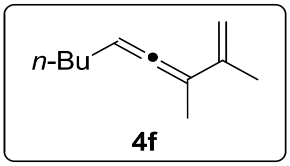

Colorless liquid (0.386 g, 2.59 mmol, 85%); ^{1}H NMR (400 MHz, CDCl$_3$): δ 5.22 (s, 1H), 4.84 (d, *J* = 2.8 Hz, 2H), 2.03 ~ 1.97 (m, 2H), 1.83 (d, *J* = 5.6 Hz, 3H), 1.81 (s, 3H), 1.39 ~ 1.33 (m, 4H), 0.88 (t, *J* = 7.2 Hz, 3H);^{13}C NMR (100 MHz, CDCl$_3$): δ 205.4, 141.5, 110.0, 102.4, 91.7, 31.4, 28.8, 22.2, 21.6, 16.8, 13.9; HRMS (EI+, m/z) calcd for $C_{11}H_{18}$ [M$^+$]: 150.1409, found: 150.1406.

Spectral data for 1-(3,4-dimethylpenta-1,2,4-trien-1-yl)-3-methoxybenzene (4g):

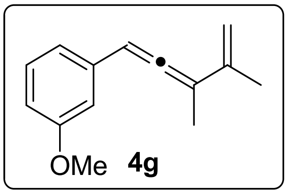

Pale yellow oil (0.295 g, 1.47 mmol, 72%); ^{1}H NMR (600 MHz, CDCl$_3$): δ 7.22 (t, *J* = 7.9 Hz, 1H), 6.89 (dd, *J* = 7.6, 1.0 Hz, 1H), 6.84 ~ 6.83 (m, 1H), 6.76 (dt, *J* = 8.3, 1.5 Hz, 1H), 6.27 (s, 1H), 5.03 (s, 1H), 4.99 (d, *J* = 1.3 Hz, 1H), 3.80 (s, 3H), 1.99 (d, *J* = 2.6 Hz, 3H), 1.88 (s, 3H); ^{13}C NMR (150 MHz, CDCl$_3$): δ 207.7, 159.9, 140.5, 136.5, 129.5, 119.4, 112.3, 112.2, 111.5, 106.6, 95.3, 55.1, 21.5, 16.3; HRMS (EI+, m/z) calcd for $C_{14}H_{16}O$ [M$^+$]:

200.1201, found: 200.1203.

Spectral data for 1-chloro-3-(3,4-dimethylpenta-1,2,4-trien-1-yl)benzene (4h):

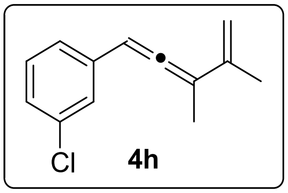

Pale yellow oil (0.330 g, 1.33 mmol, 80%); ^{1}H NMR (600 MHz, $CDCl_3$): δ 7.26 (s, 1H), 7.22 (t, *J* = 7.8 Hz, 1H), 7.17 ~ 7.13 (m, 2H), 6.24 (s, 1H), 5.05 (s, 1H), 5.01 (s, 1H), 1.99 (d, *J* = 2.7 Hz, 3H), 1.86 (s, 3H); ^{13}C NMR (150 MHz, $CDCl_3$): δ 207.9, 140.1, 137.0, 134.6, 129.8, 126.8, 126.5, 124.9, 112.1, 107.1, 94.5, 21.4, 16.2; HRMS (EI+, m/z) calcd for $C_{13}H_{13}Cl$ [M^+]: 204.0706, found: 204.0703.

Spectral data for 2-(3,4-dimethylpenta-1,2,4-trien-1-yl)naphthalene (4i):

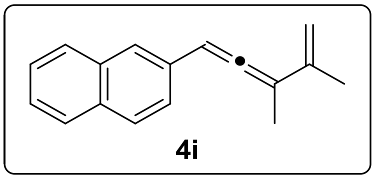

Pale yellow solid, mp: 77-78 ˚C (0.650 g, 2.95 mmol, 78%); ^{1}H NMR (600 MHz, $CDCl_3$): δ 7.79 ~ 7.75 (m, 3H), 7.66 (s, 1H), 7.47 ~ 7.41 (m, 3H), 6.49 (s, 1H), 5.07 (s, 1H), 5.02 (s, 1H), 2.05 (s, 3H), 1.91 (s, 3H); ^{13}C NMR (150 MHz, $CDCl_3$): δ 208.2, 140.6, 133.7, 132.6, 132.5, 128.2, 127.7, 127.6, 126.2, 125.5, 125.4, 124.7, 111.6, 106.7, 95.8, 21.5, 16.4; HRMS (ESI, m/z) calcd for $C_{17}H_{17}$ [M+H]: 221.1330, found: 221.1324.

Spectral data for 2-(3,4-dimethylpenta-1,2,4-trien-1-yl)furan (4j):

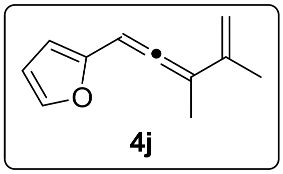

Colorless oil (0.340 g, 2.12 mmol, 72%); ^{1}H NMR (400 MHz, $CDCl_3$): δ 7.34 (dd, *J* = 2, 0.8, Hz, 1H), 6.36 (dd, *J* = 3.2, 1.6 Hz, 1H), 6.26 (d, *J* = 1.2 Hz, 1H), 6.19 (d, *J* = 3.2 Hz, 1H), 5.01 (d, *J* = 0.8 Hz, 1H), 5.00 (dd, *J* = 2.8, 1.2 Hz, 1H), 1.97 (d, *J* = 2.8 Hz, 3H), 1.86 (s, 3H);

^{13}C NMR (100 MHz, $CDCl_3$): δ 206.9, 148.7, 141.9, 140.3, 111.9, 111.3, 107.0, 106.8, 86.2, 21.5, 16.5; HRMS (EI+, m/z) calcd for $C_{11}H_{12}O$ [M^+]: 160.0888, found: 160.0883.

Spectral data for 2-(3,4-dimethylpenta-1,2,4-trien-1-yl)thiophene (4k):

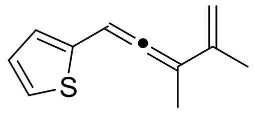

4k

Colorless oil (0.376 g, 2.13 mmol, 78%); ^1H NMR (400 MHz, $CDCl_3$): δ 7.13 (d, *J* = 5.1, Hz, 1H), 6.94 (t, *J* = 4.3 Hz, 1H), 6.89 (t, *J* = 1.7 Hz, 1H), 6.50 (s, 1H), 5.02 (s, 1H), 4.99 (d, *J* = 1.3 Hz, 1H), 1.96 (d, *J* = 2.6 Hz, 3H), 1.86 (s, 3H); ^{13}C NMR (100 MHz, $CDCl_3$): δ 207.0, 140.3, 139.5, 127.3, 124.4, 124.3, 112.0, 106.7, 89.7, 21.4, 16.3; HRMS (EI+, m/z) calcd for $C_{11}H_{12}S$ [M^+]: 176.0660, found: 176.0657.

Spectral data for (2-methylhexa-1,3,4-trien-3-yl)benzene (4l):

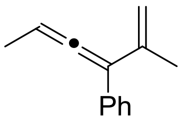

4l

Pale yellow oil (0.700 g, 4.11 mmol, 62%); ^1H NMR (600 MHz, $CDCl_3$): δ 7.32 ~ 7.31 (m, 4H), 7.25 ~ 7.23 (m, 1H), 5.48 (q, *J* = 7.1 Hz, 1H), 5.03 ~ 5.02 (m, 1H), 4.82 (d, *J* = 0.6 Hz, 1H), 1.95 (s, 3H), 1.76 (dd, *J* = 7.1, 0.8 Hz, 3H); ^{13}C NMR (150 MHz, $CDCl_3$): δ 206.2, 140.5, 137.3, 129.1, 128.1, 126.8, 114.2, 110.8, 88.0, 22.1, 14.5; HRMS (EI+, m/z) calcd for $C_{13}H_{14}$ [M^+]: 170.1096, found: 170.1089.

Spectral data for(2-methylnona-1,3,4-trien-3-yl)benzene (4m):

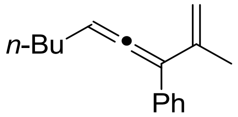

4m

Pale yellow oil (0.486 g, 2.29 mmol, 89%); ^1H NMR (400 MHz, $CDCl_3$): δ 7.33 ~ 7.30 (m,

4H), 7.28 ~ 7.21 (m, 1H), 5.52 (t, *J* = 6.4 Hz, 1H), 5.03 (d, *J* = 1.6 Hz, 1H), 4.83 (d, *J* = 0.4 Hz, 1H), 2.15 ~ 1.98 (m, 2H), 1.98 (s, 3H), 1.51 ~ 1.43 (m, 2H), 1.47 ~ 1.32 (m, 2H), 0.91 (t, *J* = 7.2 Hz, 3H); ^{13}C NMR (100 MHz, $CDCl_3$): δ 205.3, 140.4, 137.4, 129.0, 128.0, 126.7, 114.1, 111.4, 93.4, 31.3, 28.8, 22.3, 22.1, 13.9; HRMS (EI+, m/z) calcd for $C_{16}H_{20}$ [M^+]: 212.1565, found: 212.1564.

Spectral data for (2,6-dimethylhepta-1,3,4-trien-3-yl)benzene (4n):

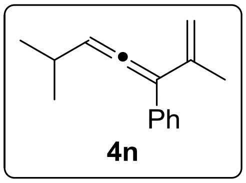

Pale yellow oil (0.725 g, 3.66 mmol, 66%); ^{1}H NMR (400 MHz, $CDCl_3$): δ 7.32 ~ 7.30 (m, 4H), 7.25 ~ 7.21 (m, 1H), 5.52 (d, *J* = 5.6 Hz, 1H), 5.02 ~ 5.00 (m, 1H), 4.80 (d, *J* = 0.4 Hz, 1H), 2.43 ~ 2.38 (m, 1H), 1.96 (s, 3H), 1.08 (d, *J* = 3.2, 3H), 1.06 (d, *J* = 3.2, 3H); ^{13}C NMR (100 MHz, $CDCl_3$): δ 203.8, 140.5, 137.4, 129.0, 128.1, 126.8, 114.1, 112.4, 100.7, 28.5, 22.5, 22.4, 22.1; HRMS (EI+, m/z) calcd for $C_{15}H_{18}$ [M^+]: 198.1409, found: 198.1407.

Spectral data for (1-cyclohexyl-4-methylpenta-1,2,4-trien-3-yl)benzene (4o):

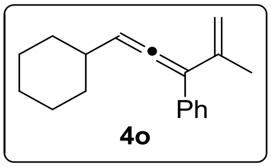

Pale yellow oil (0.350 g, 1.47 mmol, 65%); ^{1}H NMR (400 MHz, $CDCl_3$): δ 7.35 ~ 7.30 (m, 4H), 7.28 ~ 7.22 (m, 1H), 5.51 (d, *J* = 5.6 Hz, 1H), 5.03 ~ 5.01 (m, 1H), 4.82 (s, 1H), 2.18 ~ 2.05 (m, 1H), 1.97 (s, 3H), 1.83 (d, *J* = 12.0 Hz, 2H), 1.75 ~ 1.72 (m, 2H), 1.68 ~ 1.63 (m, 1H), 1.35 ~ 1.12 (m, 5H); ^{13}C NMR (100 MHz, $CDCl_3$): δ 204.2, 140.5, 137.4, 129.0, 128.0, 126.7, 114.0, 112.1, 99.3, 37.8, 33.2, 33.0, 26.1, 22.2; HRMS (EI+, m/z) calcd for $C_{18}H_{22}$ [M^+]: 238.1722, found: 238.1725.

Spectral data for (4-methylpenta-1,2,4-triene-1,3-diyl)dibenzene (4p):

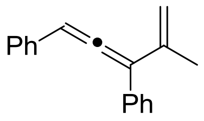

4p

Orange yellow oil (0.800 g, 3.44 mmol, 74%); ^{1}H NMR (600 MHz, $CDCl_3$): δ 7.44 ~ 7.42 (m, 2H), 7.40 ~ 7.33 (m, 6H), 7.31 ~ 7.30 (m, 1H), 7.25 ~ 7.22 (m, 1H), 6.56 (s, 1H), 5.17 ~ 5.16 (m, 1H), 5.00 ~ 5.00 (m, 1H), 2.04 (t, *J* = 0.6 Hz, 3H); ^{13}C NMR (150 MHz, $CDCl_3$): δ 207.7, 139.8, 136.2, 134.3, 129.0, 128.7, 128.3, 127.3, 127.2, 126.8, 115.5, 96.9, 22.0; HRMS (EI+, m/z) calcd for $C_{18}H_{16}$ [M^+]: 232.1252, found: 232.1253.

Spectral data for (3-(prop-1-en-2-yl)hepta-1,2-dien-1-yl)benzene (4q):

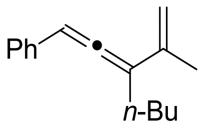

4q

Colorless liquid (0.495 g, 2.33 mmol, 90%); ^{1}H NMR (400 MHz, $CDCl_3$): δ 7.29 ~ 7.24 (m, 4H), 7.20 ~ 7.16 (m, 1H), 6.36 (s, 1H), 5.05 (s, 1H), 4.96 (s, 1H), 2.32 ~ 2.29 (m, 2H), 1.85 (s, 3H), 1.52 ~ 1.48 (m, 2H), 1.40 ~ 1.34 (m, 2H), 0.88 (t, *J* = 7.2 Hz, 3H); ^{13}C NMR (100 MHz, $CDCl_3$): δ 207.2, 140.0, 135.1, 128.6, 126.7, 126.6, 111.7, 110.9, 97.1, 30.2, 29.1, 22.7, 21.9, 13.9; HRMS (EI+, m/z) calcd for $C_{16}H_{20}$ [M^+]: 212.1595, found: 212.1557.

Spectral data for (4-methyleneocta-1,2-diene-1,3-diyl)dibenzene (4r):

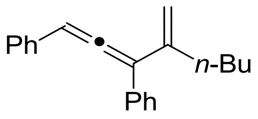

4r

Pale yellow oil (0.750 g, 2.73 mmol, 70%); ^{1}H NMR (400 MHz, $CDCl_3$): δ 7.40 ~ 7.30 (m, 8H), 7.27 ~ 7.19 (m, 2H), 6.53 (s, 1H), 5.13 (s, 1H), 5.00 (s, 1H), 2.34 ~ 2.29 (m, 2H), 1.56 ~ 1.49 (m, 2H), 1.37 ~ 1.28 (m, 2H), 0.87 (t, *J* = 7.2 Hz, 3H); ^{13}C NMR (100 MHz, $CDCl_3$): δ 207.3, 144.5, 136.5, 134.3, 128.7, 128.7, 128.3, 127.3, 127.1, 126.9, 114.8, 114.4, 96.9, 35.1, 30.8, 22.4, 13.9; HRMS (EI+, m/z) calcd for $C_{21}H_{22}$ [M^+]: 274.1722, found: 274.1726.

Spectral data for (3-methylpenta-1,2,4-trien-1-yl)benzene (4s):

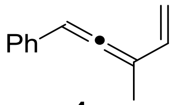

4s

Pale yellow oil (0.450 g, 2.88 mmol, 58%); ^{1}H NMR (400 MHz, $CDCl_3$): δ 7.39 ~ 7.34 (m, 4H), 7.28 ~ 7.24 (m, 1H), 6.50 (dd, *J* = 17.6, 10.8 Hz, 1H), 6.33 (s, 1H), 5.30 (dd, *J* = 17.6, 1.2 Hz, 1H), 5.19 (dd, *J* = 10.4, 0.8 Hz, 1H), 2.03 (d, *J* = 2.8 Hz, 3H); ^{13}C NMR (100 MHz, $CDCl_3$): δ 209.1, 134.8, 134.6, 128.6, 126.9, 126.9, 113.4, 104.1, 94.2, 14.5; HRMS (EI+, m/z) calcd for $C_{12}H_{12}$ [M^+]: 156.0939, found: 156.0933.

Spectral data for (3-vinylhepta-1,2-dien-1-yl)benzene (4t):

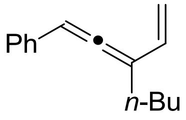

4t (5:1)

Colorless liquid (0.446 g, 2.25 mmol, 90%); ^{1}H NMR (400 MHz, $CDCl_3$); dr = 5:1; (*Major isomer*); δ 7.32 ~ 7.22 (m, 4H), 7.20 ~ 7.17 (m, 1H), 6.37 ~ 6.29 (m, 2H), 5.27 (dd, *J* = 16.7, 0.8 Hz, 1H), 5.09 (d, *J* = 10.7 Hz, 1H), 2.32 ~ 2.24 (m, 2H), 1.55 ~ 1.49 (m, 2H), 1.42 ~ 1.35 (m, 2H), 0.91 (t, *J* = 7.3 Hz, 3H); ^{13}C NMR (100 MHz, $CDCl_3$); δ 208.6, 134.7, 134.3, 128.6, 126.8, 126.8, 113.1, 109.2, 95.8, 29.8, 28.0, 22.6, 13.9; ^{1}H NMR (400 MHz, $CDCl_3$); (*Minor isomer*); δ 7.24 ~ 7.21 (m, 5H), 6.26 ~ 6.21 (m, 2H), 5.18 (d, *J* = 17.6 Hz, 1H), 5.04 (d, *J* = 10.8 Hz, 1H), 2.18 ~ 2.15 (m, 2H), 1.49 ~ 1.46 (m, 2H), 1.42 ~ 1.35 (m, 2H), 0.94 ~ 0.92 (m, 3H); ^{13}C NMR (100 MHz, $CDCl_3$); δ 212.2, 126.8, 112.9, 105.6, 90.8, 30.9, 30.3, 22.4, 13.6 other carbons are merged or not clearly visible; HRMS (EI+, m/z) calcd for $C_{15}H_{18}$ [M^+]: 198.1409, found: 198.1390.

Spectral data for 1-bromo-4-(3,4-dimethylpenta-1,2,4-trien-1-yl)benzene (4u):

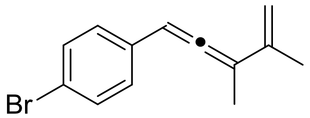

4u

Pale yellow oil (0.410 g, 1.65 mmol, 80%); ^{1}H NMR (400 MHz, $CDCl_3$): δ 7.41 (d, *J* = 8.4 Hz, 2H), 7.12 (d, *J* = 8.4 Hz, 2H), 6.24 (s, 1H), 5.03 (s, 1H), 4.99 (s, 1H), 1.98 (d, *J* = 2.8 Hz, 3H), 1.85 (s, 3H); ^{13}C NMR (100 MHz, $CDCl_3$): δ 207.7, 140.2, 131.7, 128.2, 120.4, 111.9, 107.0, 94.6, 21.4, 16.2.

Spectral data for (9*S*,9a*S*)-2,3-dimethyl-9a-phenyl-9,9a-dihydro-1*H*-cyclopenta[*b*]quinolin-9-ol (5a):

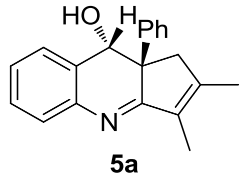

5a

Pale yellow solid, mp: 207-208 ˚C (0.144 g, 0.498 mmol, 85%); ^{1}H NMR (400 MHz, $CDCl_3$): δ 7.06 ~ 6.95 (m, 8H), 6.85 ~ 6.81 (m, 1H), 4.97 (bs, 1H), 4.73 (s, 1H), 3.68 (d, *J* = 16.8 Hz, 1H), 2.53 (d, *J* = 16.8 Hz, 1H), 1.83 (s, 3H), 1.65 (s, 3H); ^{13}C NMR (100 MHz, $CDCl_3$): δ 182.3, 158.7, 143.4, 141.6, 133.8, 129.1, 129.1, 128.2, 126.7, 126.7, 126.5, 125.7, 125.4, 73.1, 54.9, 45.3, 16.3, 8.9; HRMS (EI+, m/z) calcd for $C_{20}H_{19}NO$ [M^+]: 289.1467, found: 289.1469.

Spectral data for (9*R*,9a*S*)-2,3-dimethyl-9a-phenyl-9,9a-dihydro-1*H*-cyclopenta[*b*]quinolin-9-ol (syn-5a):

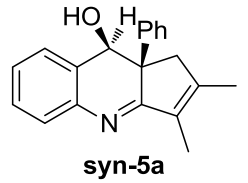

syn-5a

White solid, mp: 141-142 ˚C (0.048 g, 0.13 mmol, 19%); ^{1}H NMR (600 MHz, $CDCl_3$): δ 7.43 (d, *J* = 7.8 Hz, 1H), 7.26 ~ 7.24 (m, 1H), 7.21 (dd, *J* = 6.45, 1.0 Hz, 1H), 7.13 ~ 7.07 (m, 5H), 7.04 (t, *J* = 7.5 Hz, 1H), 4.82 (d, *J* = 10.5 Hz, 1H), 3.08 (d, *J* = 17.1 Hz, 1H), 2.99 (d, *J* = 17.1 Hz, 1H), 1.98 (s, 3H), 1.96 (s, 3H), 1.75 (s, 1H); ^{13}C NMR (100 MHz, $CDCl_3$): δ 181.2, 157.0, 145.3, 135.5, 133.9, 130.5, 128.6, 128.3, 128.2, 127.2, 126.2, 125.8, 123.2, 75.5, 50.8, 50.0, 16.3, 9.3; HRMS (EI+, m/z) calcd for $C_{20}H_{20}NO$ [M+H]: 290.1545, found: 290.1537.

Spectral data for (3a*R*,9*R*,9a*S*)-2,3-dimethyl-9a-phenyl-3a,4,9,9a-tetrahydro-1*H*-cyclopenta[*b*]quinolin-9-ol (5a-H):

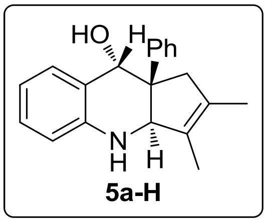

White solid, mp: 151-152 ˚C (0.090 g, 0.30 mmol, 90%); ^{1}H NMR (400 MHz, $CDCl_3$): δ 7.49 (d, *J* = 8.0 Hz, 2H), 7.17 (d, *J* = 7.6 Hz, 1H), 7.09 ~ 6.99 (m, 4H), 6.61 (dd, *J* = 9.2, 7.2 Hz, 2H), 5.07 (s, 1H), 4.44 (s, 1H), 4.30 (s, 1H), 3.03 (d, *J* = 13.6 Hz, 1H), 2.10 (d, *J* = 14.8 Hz, 1H), 2.02 (s, 1H), 1.89 (s, 3H), 1.52 (s, 3H); ^{13}C NMR (100 MHz, $CDCl_3$): δ 145.2, 143.3, 131.9, 129.1, 128.9, 128.6, 128.0, 127.0, 125.8, 125.3, 118.3, 116.4, 72.8, 60.8, 54.5, 44.4, 14.2, 10.5; HRMS (EI+, m/z) calcd for $C_{20}H_{22}NO$ [M+H]: 292.1701, found: 292.1689.

^{1}H NOE of Compound (3a*R*,9*R*,9a*S*)-2,3-dimethyl-9a-phenyl-3a,4,9,9a-tetrahydro-1*H*-cyclopenta[*b*]quinolin-9-ol (5a-H):

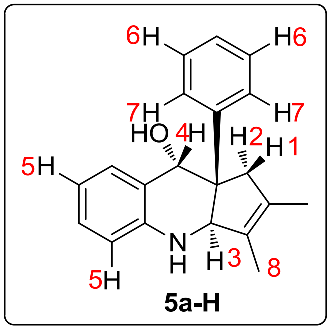

Sr. No.	Irradiation	Intensity Increase % (Key peaks)
1	H1 (δ 2.10)	H2 (δ 3.03, 24.69%), H4 (δ 5.07, 1.70%), H5 (δ 6.61, 4.78%), H7 (δ 7.49, 5.13%)
2	H2 (δ 3.03)	H1 (δ 2.10, 27.76%), H3 (δ 4.82, 4.44%), H4 (δ 5.07, 1.71%)
3	H3 (δ 4.82)	H2 (δ 3.03, 2.31%), H4 (δ 5.07, 1.68%), H5 (δ 6.61, 3.66%), $CH_3$8 (δ 1.89, 3.40%)
4	H4 (δ 5.07)	H6 (δ 7.09 ~ 6.99, 10.41%), H7 (δ 7.49, 18.61%)
5	H7 (δ 7.49)	H1 (δ 2.10, 1.94%), H4 (δ 5.07, 7.77%), H6 (δ 7.09 ~ 6.99, 10.55%), $CH_3$8 (δ 1.89, 1.35%)

Spectral data for (1a*R*,7*S*,7a*S*,8a*S*)-1a,8a-dimethyl-7a-phenyl-7,7a,8,8a-tetrahydro-1a*H*-oxireno[2',3':4,5]cyclopenta[1,2-*b*]quinolin-7-ol (5a-O):

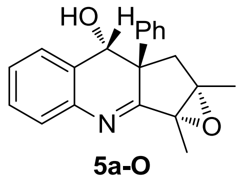

Colorless liquid (0.160 g, 0.52 mmol, 76%); ^{1}H NMR (400 MHz, $CDCl_3$): δ 7.37 (d, *J* = 7.7 Hz, 1H), 7.24 ~ 7.21 (m, 1H), 7.14 ~ 7.08 (m, 2H), 7.06 ~ 6.98 (m, 5H), 4.52 (d, *J* = 7.4 Hz, 1H), 3.21 (d, *J* = 14.4 Hz, 1H), 2.30 (s, 1H), 2.26 (d, *J* = 14.4 Hz, 1H), 1.76 (s, 3H), 1.47 (s, 3H); ^{13}C NMR (100 MHz, $CDCl_3$): δ 175.1, 142.8, 140.9, 129.6, 129.0, 128.8, 128.1, 127.7, 127.1, 126.5, 126.2, 72.7, 68.9, 68.4, 55.1, 42.6, 15.8, 10.4; HRMS (EI+, m/z) calcd for $C_{20}H_{20}NO_2$ [M+H]: 306.1494, found: 306.1503.

^{1}H NOE of Compound (1a*R*,7*S*,7a*S*,8a*S*)-1a,8a-dimethyl-7a-phenyl-7,7a,8,8a-tetrahydro-1a*H*-oxireno[2',3':4,5]cyclopenta[1,2-*b*]quinolin-7-ol (5a-O):

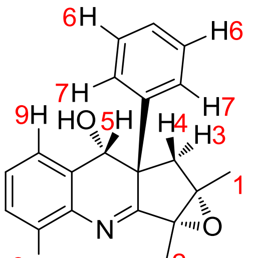

Sr. No.	Irradiation	Intensity Increase % (Key peaks)
1	$CH_3$1 (δ 1.47)	$CH_3$2 (δ 1.76, 1.01%), H3 (δ 2.26, 0.61%), H4 (δ 2.30, 0.27%)
2	$CH_3$2 (δ 1.76)	$CH_3$1 (δ 1.47, 1.76%), H8 (δ 7.06 ~ 6.98, 0.62%)
3	H3 (δ 2.26)	$CH_3$1 (δ 1.47, 0.41%), H4 (δ 2.30, 21.03%), H5 (δ 4.52, 0.32%), H6,7 (δ 7.06 ~ 6.98, 4.42%)
4	H4 (δ 2.30)	$CH_3$1 (δ 1.47, 1.39%), H3 (δ 2.26, 27.70%), H5 (δ 4.52, 3.02%), H9 (δ 7.37, 0.94%)
5	H5 (δ, 4.52)	H3 (δ 2.26, 0.69%), H4 (δ 2.30, 1.63), H6 (δ 7.06 ~ 6.98, 11.36%), H7 (δ 7.14 ~ 7.08, 9.75%)

Spectral data for (9*S*,9a*S*)-2,3-dimethyl-9a-phenyl-9,9a-dihydro-1*H*-cyclopenta[*b*]quinolin-9-yl acetate (5a-OAc):

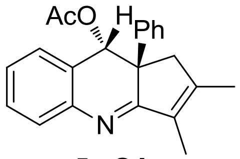

5a-OAc

White solid, mp: 188-189 ˚C (0.503 g, 1.52 mmol, 80%); ^{1}H NMR (400 MHz, $CDCl_3$): δ 7.36 (d, *J* = 7.6 Hz, 1H), 7.21 (t, *J* = 7.6 Hz, 1H), 7.12 ~ 7.09 (m, 5H), 7.07 ~ 7.05 (m, 1H), 6.90 (t, *J* = 7.2 Hz, 1H), 6.18 (s, 1H), 3.07 (d, *J* = 16.8 Hz, 1H), 2.60 (d, *J* = 16.8 Hz, 1H), 2.03 (s, 3H), 1.96 (s, 3H), 1.92 (s, 3H); ^{13}C NMR (100 MHz, $CDCl_3$): δ 180.3, 170.7, 156.9, 145.0, 140.5, 133.9, 130.1, 130.0, 128.4, 127.1, 126.8, 126.0, 125.7, 122.8, 73.2, 50.9, 46.0, 21.1, 16.2, 9.1; HRMS (EI+, m/z) calcd for $C_{22}H_{22}NO_2$ [M+H]: 332.1651, found: 332.1637.

Spectral data for (1*R*,4*S*,5a*S*,6*S*)-1,4-dimethyl-5a-phenyl-4,5,5a,6-tetrahydro-1*H*-1,4-epoxy[1,2]dioxepino[4,5-*b*]quinolin-6-yl acetate (5a-O$_3$):

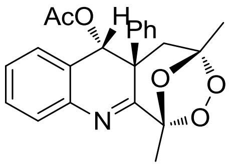

5a-O$_3$

White solid, mp: 195-196 ˚C (0.323 g, 0.85 mmol, 85%); ^{1}H NMR (600 MHz, $CDCl_3$): δ 7.37 (d, *J* = 7.8 Hz, 1H), 7.26 ~ 7.24 (m, 1H), 7.16 (t, *J* = 7.5 Hz, 2H), 7.11 ~ 7.01 (m, 5H), 5.89 (s, 1H), 2.68 (d, *J* = 14.7 Hz, 1H), 2.59 (d, *J* = 14.7 Hz, 1H), 2.04 (s, 3H), 2.02 (s, 3H), 1.52 (s, 3H); ^{13}C NMR (100 MHz, $CDCl_3$); δ 170.7, 167.1, 142.6, 140.1, 130.1, 129.3, 128.9, 128.3, 127.5, 127.3, 126.4, 123.7, 108.2, 106.5, 72.6, 44.6, 40.9, 22.1, 21.2, 17.2; HRMS (EI+, m/z) calcd for $C_{22}H_{22}NO_5$ [M+H]: 380.1498, found: 380.1495.

Spectral data for (9*S*,9a*S*)-2,3-dimethyl-9a-(*p*-tolyl)-9,9a-dihydro-1*H*-cyclopenta[*b*]quinolin-9-ol (5b):

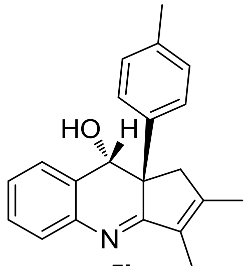

5b

Pale yellow solid, mp: 223-224 ˚C (0.135 g, 0.445 mmol, 82%); ^{1}H NMR (400 MHz, $CDCl_3$): δ 6.99 ~ 6.96 (m, 3H), 6.89 ~ 6.83 (m, 5H), 4.72 (s, 1H), 3.66 (dd, *J* = 16.8, 1.2 Hz, 1H), 2.52 (d, *J* = 16.8 Hz, 1H), 2.13 (s, 3H), 1.83 (s, 3H), 1.66 (s, 3H); ^{13}C NMR (100 MHz, $CDCl_3$): δ 182.5, 158.7, 143.5, 138.6, 136.1, 133.8, 129.2, 129.1, 128.9, 126.8, 126.6, 125.7, 125.4, 73.2, 54.5, 45.3, 20.8, 16.3, 8.9; HRMS (EI+, m/z) calcd for $C_{21}H_{21}NO$ [M^+]: 303.1623, found: 303.1620.

Spectral data for (9*S*,9a*S*)-9a-(4-methoxyphenyl)-2,3-dimethyl-9,9a-dihydro-1*H*-cyclopenta[*b*]quinolin-9-ol (5c):

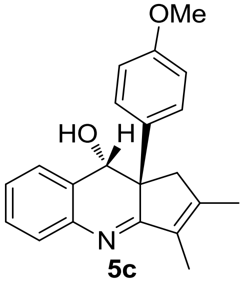

5c

Pale yellow solid, mp: 168-169 ˚C (0.124 g, 0.388 mmol, 78%); ^{1}H NMR (400 MHz, $CDCl_3$): δ 6.99 ~ 6.95 (m, 3H), 6.90 ~ 6.83 (m, 3H), 6.57 (d, *J* = 8.4 Hz, 2H), 4.68 (s, 1H), 3.64 (d, *J* = 16.4 Hz, 1H), 3.60 (s, 3H), 2.49 (d, *J* = 16.4 Hz, 1H), 1.83 (s, 3H), 1.66 (s, 3H); ^{13}C NMR (100 MHz, $CDCl_3$): δ 182.6, 158.7, 157.9, 143.4, 133.8, 133.4, 129.2, 129.1, 127.7, 126.8, 125.7, 125.4, 113.5, 73.2, 54.9, 54.1, 45.3, 16.3, 8.9; HRMS (EI+, m/z) calcd for $C_{21}H_{21}NO_2$ [M^+]: 319.1572, found: 319.1574.

Spectral data for (9*S*,9a*S*)-9a-(4-chlorophenyl)-2,3-dimethyl-9,9a-dihydro-1*H*-cyclopenta[*b*]quinolin-9-ol (5d):

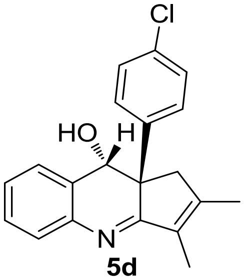

5d

White solid, mp: 208-209 ˚C (0.139 g, 0.429 mmol, 88%); ^{1}H NMR (400 MHz, $CDCl_3$): δ 7.01 ~ 6.94 (m, 5H), 6.90 ~ 6.84 (m, 3H), 4.66 (s, 1H), 3.64 (d, *J* = 16.4 Hz, 1H), 2.45 (d, *J* = 16.8 Hz, 1H), 1.83 (s, 3H), 1.62 (s, 3H); ^{13}C NMR (100 MHz, $CDCl_3$): δ 181.9, 158.9, 143.3, 140.1, 133.8, 132.3, 129.4, 129.2, 128.4, 128.1, 126.4, 125.9, 125.5, 73.0, 54.3, 45.2, 16.3, 8.9; HRMS (ESI, m/z) calcd for $C_{20}H_{19}ClNO$ [M+H]: 324.1155, found: 324.1134.

Spectral data for (9*S*,9a*S*)-2,3-dimethyl-9a-(4-(trifluoromethyl)phenyl)-9,9a-dihydro-1*H*-cyclopenta[*b*]quinolin-9-ol (5e):

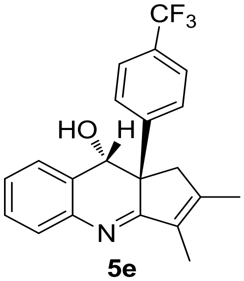

5e

White solid, mp: 231-232 ˚C (0.128 g, 0.358 mmol, 85%); ^{1}H NMR (400 MHz, $CDCl_3$): δ 7.32 (d, *J* = 8.0 Hz, 2H), 7.11 (d, *J* = 8.0 Hz, 2H), 7.07 ~ 7.05 (m, 2H), 6.97 (d, *J* = 7.2 Hz, 1H), 6.92 ~ 6.87 (m, 1H), 4.74 (s, 1H), 3.95 (bs, 1H), 3.67 (d, *J* = 16.8 Hz, 1H), 2.50 (d, *J* = 16.8 Hz, 1H), 1.87 (s, 3H), 1.74 (s, 3H); ^{19}F NMR (500 MHz, $CDCl_3$): δ -62.6; ^{13}C NMR (100 MHz, $CDCl_3$): δ 181.4, 158.9, 145.7, 143.5, 134.0, 129.8, 129.1, 127.2, 126.2, 126.1, 125.9, 125.3, 125.3, 72.9, 54.4, 45.2, 16.3, 9.0; HRMS (ESI, m/z) calcd for $C_{21}H_{19}F_3NO$ [M+H]: 358.1419, found: 358.1413.

Spectral data for (9*S*,9a*R*)-9a-butyl-2,3-dimethyl-9,9a-dihydro-1*H*-cyclopenta[*b*]quinolin-9-ol (5f):

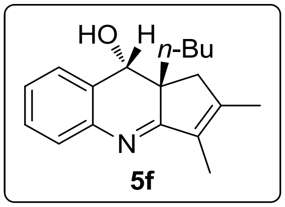

White solid, mp: 139-140 ˚C (0.15 g, 0.557 mmol, 84%); ^{1}H NMR (400 MHz, $CDCl_3$): δ 7.14 (t, *J* = 7.6 Hz, 1H), 7.10 (d, *J* = 6.4 Hz, 1H), 7.01 (t, *J* = 8.0 Hz, 2H), 4.33 (s, 1H), 3.13 (d, *J* = 16.8 Hz, 1H), 2.16 (d, *J* = 17.2 Hz, 1H), 1.93 (s, 3H), 1.63 (s, 3H), 1.05 ~ 1.0 (m, 6H), 0.69 (t, *J* = 6.0 Hz, 3H); ^{13}C NMR (100 MHz, $CDCl_3$): δ 185.4, 159.1, 142.7, 133.5, 129.2, 129.1, 127.1, 125.7, 125.5, 70.8, 49.0, 40.0, 34.3, 26.9, 23.0, 16.2, 13.7, 8.6; HRMS (EI+, m/z) calcd for $C_{18}H_{24}NO$ [M+H]: 270.1858, found: 270.1963.

Spectral data for (9*S*,9a*S*)-9a-(3-methoxyphenyl)-2,3-dimethyl-9,9a-dihydro-1*H*-cyclopenta[*b*]quinolin-9-ol (5g):

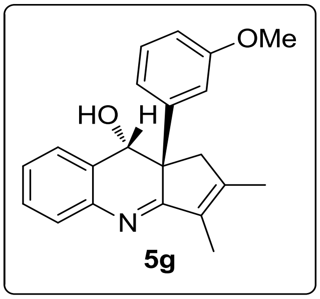

Yellow solid, mp: 180-181 ˚C (0.134 g, 0.420 mmol, 84%); ^{1}H NMR (400 MHz, $CDCl_3$): δ 7.07 ~ 6.96 (m, 4H), 6.86 (t, *J* = 6.88 Hz, 1H), 6.61 (d, *J* = 7.6 Hz, 1H), 6.55 ~ 6.54 (m, 2H), 4.73 (s, 1H), 3.64 ~ 3.60 (m, 4H), 2.54 (d, *J* = 16.8 Hz, 1H), 1.86 (s, 3H), 1.72 (s, 3H); ^{13}C NMR (100 MHz, $CDCl_3$): δ 182.2, 159.2, 158.8, 143.5, 143.2, 133.8, 129.3, 129.1, 129.1, 126.7, 125.7, 125.5, 119.3, 113.1, 111.5, 73.1, 54.9, 54.7, 45.3, 16.3, 8.9; HRMS (ESI, m/z) calcd for $C_{21}H_{22}NO_2$ [M+H]: 320.1651, found: 320.1641.

Spectral data for (9*S*,9a*S*)-9a-(3-chlorophenyl)-2,3-dimethyl-9,9a-dihydro-1*H*-cyclopenta[*b*]quinolin-9-ol (5h):

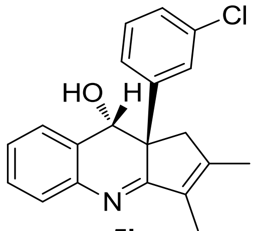

5h

White solid, mp: 216-217 ˚C (0.138 g, 0.426 mmol, 87%); ^{1}H NMR (400 MHz, $CDCl_3$): δ 7.03 (d, *J* = 3.2 Hz, 2H), 6.99 ~ 6.94 (m, 4H), 6.90 ~ 6.86 (m, 1H), 6.86 ~ 6.82 (m, 1H), 4.69 (s, 1H), 3.63 (d, *J* = 16.8, 1H), 2.48 (d, *J* = 16.8, 1H), 1.86 (s, 3H), 1.70 (s, 3H); ^{13}C NMR (100 MHz, $CDCl_3$): δ 181.5, 158.9, 143.7, 143.4, 134.1, 134.0, 129.6, 129.5, 129.1, 127.1, 127.0, 126.3, 126.0, 125.7, 124.9, 72.9, 54.3, 45.2, 16.3, 9.0; HRMS (ESI, m/z) calcd for $C_{20}H_{19}ClNO$ [M+H]: 324.1155, found: 324.1190.

Spectral data for (9*S*,9a*S*)-2,3-dimethyl-9a-(naphthalen-2-yl)-9,9a-dihydro-1*H*-cyclopenta[*b*]quinolin-9-ol (5i):

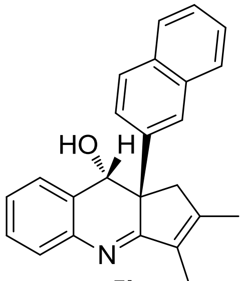

5i

Pale yellow solid, mp: 240-241 ˚C (0.126 g, 0.371 mmol, 82%); ^{1}H NMR (400 MHz, $CDCl_3$): δ 7.66 ~ 7.57 (m, 3H), 7.33 ~ 7.29 (m, 4H), 7.09 (d, *J* = 7.6 Hz, 1H), 7.01 ~ 6.95 (m, 2H), 6.81 (t, *J* = 7.4 Hz, 1H), 4.91 (s, 1H), 4.26 (bs, 1H), 3.74 (d, *J* = 16.8 Hz, 1H), 2.63 (d, *J* = 17.2 Hz, 1H), 1.86 (s, 3H), 1.79 (s, 3H); ^{13}C NMR (100 MHz, $CDCl_3$): δ 182.2, 158.7, 143.6, 138.8, 134.0, 133.0, 132.1, 129.4, 129.1, 128.1, 127.9, 127.2, 126.6, 125.8, 125.7, 125.6, 125.3, 125.1, 73.0, 54.8, 45.2, 16.3, 9.0; HRMS (EI+, m/z) calcd for $C_{24}H_{21}NO$ [M^+]: 339.1623, found: 339.1629.

Spectral data for (9*S*,9a*S*)-9a-(furan-2-yl)-2,3-dimethyl-9,9a-dihydro-1*H*-cyclopenta[*b*]quinolin-9-ol (5j):

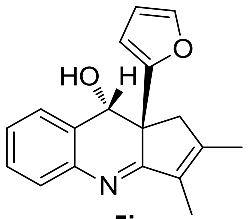

5j

Brown solid, mp: 204-205 ˚C (0.146 g, 0.522 mmol, 83%); ^{1}H NMR (400 MHz, $CDCl_3$): δ 7.15 ~ 7.10 (m, 4H), 7.0 ~ 6.96 (m, 1H), 5.97 ~ 5.95 (m, 1H), 5.55 (d, *J* = 2.8 Hz, 1H), 4.83 (s, 1H), 3.39 (d, *J* = 17.2 Hz, 1H), 2.68 (d, *J* = 17.2 Hz, 1H), 1.94 (s, 3H), 1.78 (s, 3H); ^{13}C NMR (100 MHz, $CDCl_3$): δ 179.9, 159.3, 153.3, 143.4, 141.5, 133.5, 129.7, 129.1, 126.8, 126.0, 109.8, 105.8, 69.9, 50.0, 41.2, 16.4, 9.1; HRMS (ESI, m/z) calcd for $C_{18}H_{18}NO_2$ [M+H]: 280.1338, found: 280.1337.

Spectral data for (9*S*,9a*S*)-2,3-dimethyl-9a-(thiophen-2-yl)-9,9a-dihydro-1*H*-cyclopenta[*b*]quinolin-9-ol (5k):

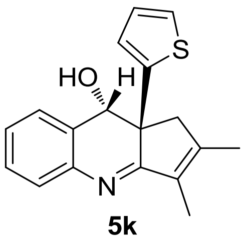

5k

Brown solid, mp: 213-214 ˚C (0.141 g, 0.477 mmol, 84%); ^{1}H NMR (400 MHz, $CDCl_3$): δ 7.13 ~ 7.10 (m, 3H), 6.99 ~ 6.93 (m, 2H), 6.61 (t, *J* = 4.4 Hz, 1H), 6.47 (d, *J* = 3.6 Hz, 1H), 4.80 (s, 1H), 3.60 (d, *J* = 16.8 Hz, 1H), 3.45 (bs, 1H), 2.58 (d, *J* = 16.8 Hz, 1H), 1.93 (s, 3H), 1.76 (s, 3H), ^{13}C NMR (100 MHz, $CDCl_3$): δ 181.3, 158.9, 145.3, 143.3, 133.4, 129.6, 129.4, 126.8, 126.1, 126.0, 124.1, 123.5, 72.7, 51.0, 45.8, 16.4, 9.1; HRMS (ESI, m/z) calcd for $C_{18}H_{18}NOS$ [M+H]: 296.1109, found: 296.1101.

Spectral data for (9*S*,9a*R*)-2,9a-dimethyl-3-phenyl-9,9a-dihydro-1*H*-cyclopenta[*b*]quinolin-9-ol (5l):

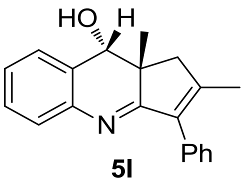

5l

Pale yellow solid, mp: 191-192 ˚C (0.142 g, 0.491 mmol, 84%); ^{1}H NMR (400 MHz, $CDCl_3$): δ 7.44 ~ 7.40 (m, 4H), 7.34 ~ 7.22 (m, 4H), 7.15 ~ 7.09 (m, 1H), 4.39 (s, 1H), 3.36 (d, *J* = 17.2 Hz, 1H), 2.26 (d, *J* = 17.6 Hz, 1H), 2.13 (s, 3H), 0.99 (s, 3H); ^{13}C NMR (100 MHz, $CDCl_3$): δ 183.3, 160.7, 143.2, 137.0, 133.0, 129.9, 129.8, 129.1, 128.1, 127.4, 127.1, 126.7, 126.1, 72.3, 44.7, 43.3, 22.4, 17.7; HRMS (EI+, m/z) calcd for $C_{20}H_{19}NO$ [M^+]: 289.1467, found: 289.1466.

^{1}H NOE of Compound (9*S*,9a*R*)-2,9a-dimethyl-3-phenyl-9,9a-dihydro-1*H*-cyclopenta[*b*]quinolin-9-ol (5l):

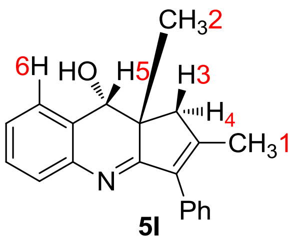

5l

Sr. No.	Irradiation	Intensity Increase % (Key peaks)
1	$CH_3$2 (δ 0.98)	H3 (δ 2.26, 1.86%), H5 (δ 4.36, 3.19%)
2	OH (δ 1.76)	H4 (δ 3.33, 0.74%), H5 (δ 4.36, 0.89%)
3	H3 (δ 2.26)	$CH_3$2 (δ 0.98, 4.05%), H4 (δ 3.33, 30.53%)
4	H4 (δ 3.33)	OH (δ 1.76, 0.61%), $CH_3$1 (δ 2.13, 0.26%), H3 (δ 2.26, 26.20%), H5 (δ 4.36, 2.36%)
5	H5 (δ 4.36)	$CH_3$2 (δ 0.98, 3.72%), OH (δ 1.76, 1.67%), H4 (δ 3.33, 1.62%), H6 (δ 7.08 ~ 7.01, 1.51%)

Spectral data for (9*S*,9a*R*)-9a-butyl-2-methyl-3-phenyl-9,9a-dihydro-1*H*-cyclopenta[*b*]quinolin-9-ol (5m):

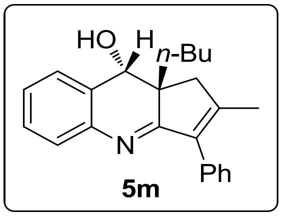

White solid, mp: 170-171 ˚C (0.133 g, 0.401 mmol, 85%); ^{1}H NMR (600 MHz, CDCl3): δ 7.45 ~ 7.40 (m, 4H), 7.33 ~ 7.25 (m, 3H), 7.20 (dd, *J* = 6.1, 1.2 1H), 7.10 ~ 7.08 (m, 1H), 4.43 (s, 1H), 3.29 (dd, *J* = 16.1, 1.3 Hz, 1H), 2.36 (dd, *J* = 16.7, 0.7 Hz, 1H), 2.12 (s, 3H), 1.66 (s, 1H), 1.33 ~ 1.30 (m, 1H), 1.23 ~ 1.17 (m, 3H), 1.13 ~ 1.08 (m, 2H), 0.76 (t, *J* = 7.3 Hz, 3H); ^{13}C NMR (100 MHz, CDCl3): δ 183.2, 161.1, 143.4, 138.2, 133.0, 129.8, 129.8, 128.9, 128.1, 127.4, 126.9, 126.7, 126.0, 71.2, 48.4, 40.7, 34.5, 27.0, 23.2, 17.5, 13.9; HRMS (ESI, m/z) calcd for $C_{23}H_{26}NO$ [M+H]: 332.2014, found: 332.2022.

Spectral data for (9*S*,9a*S*)-9a-isopropyl-2-methyl-3-phenyl-9,9a-dihydro-1*H*-cyclopenta[*b*]quinolin-9-ol (5n):

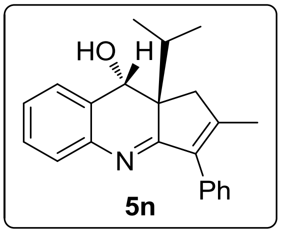

Pale yellow solid, mp: 192-193 ˚C (0.136 g, 0.428 mmol, 85%); ^{1}H NMR (400 MHz, $CDCl_3$): δ 7.44 ~ 7.40 (m, 4H), 7.34 ~ 7.31 (m, 1H), 7.27 ~ 7.26 (m, 2H), 7.17 (d, *J* = 6.8 Hz, 1H), 7.09 ~ 7.05 (m, 1H), 4.53 (s, 1H), 3.09 (d, *J* = 18.0 Hz, 1H), 2.40 (d, *J* = 17.6 Hz, 1H), 2.11 (s, 3H), 1.87 (bs, 1H), 1.78 ~ 1.77 (m, 1H), 0.83 (d, *J* = 6.4 Hz, 3H), 0.78 (d, *J* = 6.8 Hz, 3H); ^{13}C NMR (100 MHz, $CDCl_3$): δ 183.9, 162.2, 143.7, 139.3, 133.0, 129.8, 129.7, 128.6, 128.1, 127.4, 126.6, 126.4, 125.8, 70.7, 51.6, 36.1, 29.3, 19.1, 17.3, 17.2; HRMS (EI+, m/z) calcd for $C_{22}H_{23}NO$ [M^+]: 317.1780, found: 317.1785.

Spectral data for (9*S*,9a*S*)-9a-cyclohexyl-2-methyl-3-phenyl-9,9a-dihydro-1*H*-cyclopenta[*b*]quinolin-9-ol (5o):

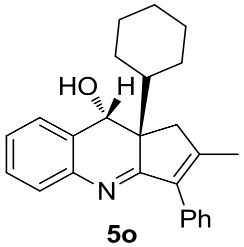

5o

Pale yellow solid, mp: 151-152 ˚C (0.123 g, 0.344 mmol, 82%); ^{1}H NMR (400 MHz, $CDCl_3$): δ 7.45 ~ 7.39 (m, 4H), 7.34 ~ 7.29 (m, 1H), 7.27 ~ 7.26 (m, 2H), 7.15 ~ 7.13 (m, 1H), 7.09 ~ 7.05 (m, 1H), 4.55 (d, J = 2.8 Hz, 1H), 3.12 (d, J = 18.0 Hz, 1H), 2.47 (d, J = 17.6 Hz, 1H), 2.11 (s, 3H), 1.67 ~ 1.52 (m, 5H), 1.42 ~ 1.39 (m, 1H), 1.05 ~ 0.94 (m, 5H); ^{13}C NMR (100 MHz, $CDCl_3$): δ 183.9, 161.9, 143.6, 139.2, 133.0, 129.8, 129.7, 128.5, 128.0, 127.4, 126.6, 126.5, 125.8, 70.1, 51.6, 39.9, 37.4, 29.1, 26.8, 26.4, 26.3, 26.2, 17.2; HRMS (EI+, m/z) calcd for $C_{25}H_{27}NO$ [M^+]: 357.2093, found: 357.2095.

Spectral data for (9*S*,9a*S*)-2-methyl-3,9a-diphenyl-9,9a-dihydro-1*H*-cyclopenta[*b*]quinolin-9-ol (5p):

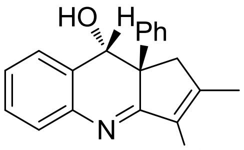

5p

Pale yellow solid, mp: 177-178 ˚C (0.121 g, 0.344 mmol, 80%); ^{1}H NMR (400 MHz, $CDCl_3$): δ 7.53 (d, J = 8.0 Hz, 2H), 7.48 (t, J = 7.4 Hz, 2H), 7.38 (t, J = 7.6 Hz, 1H), 7.28 ~ 7.07 (m, 7H), 7.98 (d, J = 7.2 Hz, 1H), 6.91 (t, J = 7.2 Hz, 1H), 4.80 (s, 1H), 3.76 (d, J = 17.2 Hz, 1H), 2.73 (d, J = 17.2 Hz, 1H), 2.16 (bs, 1H), 2.06 (s, 3H); ^{13}C NMR (100 MHz, $CDCl_3$): δ 180.3, 160.8, 143.9, 141.3, 138.4, 133.0, 129.9, 129.8, 128.7, 128.5, 128.2, 127.6, 126.9, 126.8, 126.5, 126.3, 126.2, 73.2, 54.0, 45.8, 17.6; HRMS (EI+, m/z) calcd for $C_{25}H_{21}NO$ [M^+]: 351.1623, found: 351.1622.

Spectral data for (9*S*,9a*S*)-3-butyl-2-methyl-9a-phenyl-9,9a-dihydro-1*H*-cyclopenta[*b*]quinolin-9-ol (5q):

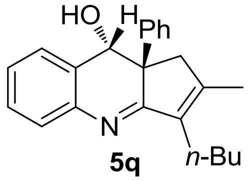

White solid, mp: 187-188 ˚C (0.134 g, 0.404 mmol, 86%); ^{1}H NMR (400 MHz, $CDCl_3$): δ 7.19 (d, *J* = 7.6 Hz, 1H), 7.12 ~ 7.03 (m, 6H), 6.96 ~ 6.94 (m, 1H), 6.89 ~ 6.85 (m, 1 H), 4.75 (s, 1 H), 3.61 (d, *J* = 16.8 Hz, 1H), 2.57 (d, *J* = 16.9 Hz, 1H), 2.53 ~ 2.48 (m, 1H), 2.23 ~ 2.15 (m, 1H), 1.90 (s, 3H), 1.66 ~ 1.55 (m, 2H), 1.36 ~ 1.30 (m, 2H), 0.91 (t, *J* = 7.3 Hz, 3H); ^{13}C NMR (100 MHz, $CDCl_3$): δ 181.6, 158.4, 143.5, 141.7, 138.6, 129.1, 129.0, 128.2, 126.8, 126.5, 126.5, 125.6, 125.5, 73.3, 54.6, 45.3, 30.4, 23.3, 22.6, 16.3, 13.9; HRMS (ESI, m/z) calcd for $C_{23}H_{26}NO$ [M+H]: 332.2014, found: 332.1997.

Spectral data for (9*S*,9a*S*)-2-butyl-3,9a-diphenyl-9,9a-dihydro-1*H*-cyclopenta[*b*]quinolin-9-ol (5r):

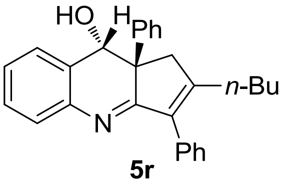

Pale yellow solid, mp: 159-160 ˚C (0.115 g, 0.292 mmol, 80%); ^{1}H NMR (400 MHz, $CDCl_3$): δ 7.49 ~ 7.46 (m, 4H), 7.39 ~ 7.34 (m, 1H), 7.27 (d, *J* = 7.6 Hz, 1H), 7.22 ~ 7.06 (m, 6H), 6.99 (dd, *J* = 7.2, 1.2 Hz, 1H), 6.92 (td, *J* = 7.2, 1.2 Hz, 1H), 4.82 (s, 1H), 3.76 (d, *J* = 17.2 Hz, 1H), 2.75 (d, *J* = 17.2 Hz, 1H), 2.53 ~ 2.45 (m, 1H), 2.36 ~ 2.28 (m, 1H), 2.15 (bs, 1H), 1.46 ~ 1.41 (m, 2H), 1.25 ~ 1.15 (m, 2H), 0.79 (t, *J* = 7.2 Hz, 3H); ^{13}C NMR (100 MHz, $CDCl_3$): δ 180.6, 165.1, 144.1, 141.3, 138.5, 133.3, 129.9, 129.8, 128.8, 128.4, 128.2, 127.6, 126.9, 126.7, 126.7, 126.4, 126.2, 73.2, 53.8, 43.2, 30.7, 29.9, 22.5, 13.7; HRMS (EI+, m/z) calcd for $C_{28}H_{27}NO$ [M^+]: 393.2093, found: 393.2099.

Spectral data for (9*S*,9a*S*)-3-methyl-9a-phenyl-9,9a-dihydro-1*H*-cyclopenta[*b*]quinolin-9-ol (5s):

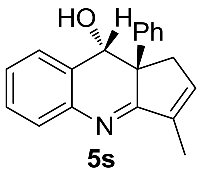

5s

White solid, mp: 189-190 ˚C (0.144 g, 0.523 mmol, 82%); ^{1}H NMR (400 MHz, $CDCl_3$): δ 7.22 ~ 7.20 (m, 1H), 7.13 ~ 7.03 (m, 6H), 6.98 (d, *J* = 7.3 Hz, 1H), 6.92 (t, *J* = 7.3 Hz, 1H), 6.64 (s, 1H), 4.81 (s, 1H), 3.64 (dd, *J* = 17.3, 1.5 Hz, 1H), 2.84 (bs, 1H), 2.63 (d, *J* = 17.3, Hz, 1H), 1.97 (s, 3H); ^{13}C NMR (100 MHz, $CDCl_3$): δ 181.5, 147.9, 143.6, 141.2, 140.2, 129.7, 128.9, 128.3, 126.8, 126.7, 126.4, 126.4, 126.2, 73.2, 54.8, 40.5, 11.6; HRMS (ESI, m/z) calcd for $C_{19}H_{18}NO$ [M+H]: 276.1388, found: 276.1379.

Spectral data for (9*S*,9a*S*)-3-butyl-9a-phenyl-9,9a-dihydro-1*H*-cyclopenta[*b*]quinolin-9-ol (5t):

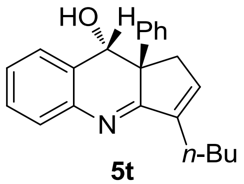

5t

White solid, mp: 161-162 ˚C (0.133 g, 0.419 mmol, 83%); ^{1}H NMR (400 MHz, $CDCl_3$): δ 7.18 ~ 7.10 (m, 7 H), 7.05 (d, *J* = 7.2 Hz, 1H), 6.97 (t, *J* = 7.6 Hz, 1H), 6.67 (s, 1H), 4.87 (s, 1H), 3.76 (dd, *J* = 15.2, 2.4 Hz, 1H), 2.73 (d, *J* = 17.6 Hz, 1H), 2.50 (d, *J* = 7.2 Hz, 1H), 2.36 (d, *J* = 6.4 Hz, 1H), 1.74 ~ 1.64 (m, 1H), 1.62 ~ 1.56 (m, 1H), 1.49 ~ 1.41 (m, 2H), 1.02 (t, *J* = 7.6 Hz, 3H); ^{13}C NMR (100 MHz, $CDCl_3$): δ 181.2, 146.4, 144.7, 143.3, 141.4, 129.5, 128.9, 128.2, 126.7, 126.4, 126.2, 125.8, 124.2, 73.2, 55.4, 40.5, 29.5, 25.6, 25.5, 14.0; HRMS (ESI, m/z) calcd for $C_{22}H_{24}NO$ [M+H]: 318.1858, found: 318.1850.

Spectral data for 1-(4-bromophenyl)-4-methylpent-4-en-2-yn-1-yl acetate (6c):

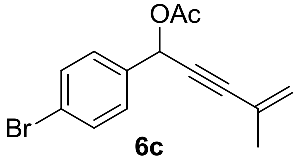

6c

Colorless liquid (0.537 g, 1.8317 mmol, 92%); ^{1}H NMR (400 MHz, $CDCl_3$): δ 7.49 (d, *J* = 8.4 Hz, 2H), 7.37 (d, *J* = 8.4 Hz, 2H), 6.51 (s, 1H), 5.35 (s, 1H), 5.28 (s, 1H), 2.08 (s, 3H), 1.88 (s, 3H); ^{13}C NMR (100 MHz, $CDCl_3$): δ 169.7, 136.3, 131.8, 129.4, 125.7, 123.5, 123.0, 88.5, 83.9, 65.3, 23.1, 21.0; HRMS (EI+, m/z) calcd for $C_{14}H_{13}BrO_2$ [M^+]: 292.0099, found: 292.0093.

Spectral data for 1-(4-methoxyphenyl)-4-methylpent-4-en-2-yn-1-yl acetate (6e):

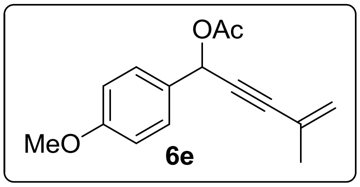

Colorless liquid (0.549 g, 2.25 mmol, 91%); ^{1}H NMR (400 MHz, $CDCl_3$): δ 7.45 (d, *J* = 8.4 Hz, 2H), 6.88 (d, *J* = 8.0 Hz, 2H), 6.52 (s, 1H), 5.35 (s, 1H), 5.26 (s, 1H), 3.79 (s, 3H), 2.06 (s, 3H), 1.89 (s, 3H); ^{13}C NMR (100 MHz, $CDCl_3$): δ 169.9, 160.0, 129.3, 128.1, 126.0, 123.0, 113.9, 87.9, 84.7, 65.7, 55.3, 23.2, 21.1; HRMS (ESI, m/z) calcd for $C_{15}H_{16}NaO_3$ [M+Na]: 267.0997, found: 267.1013.

Spectral data for 4-methyl-1-(thiophen-2-yl)pent-4-en-2-yn-1-yl acetate (6f):

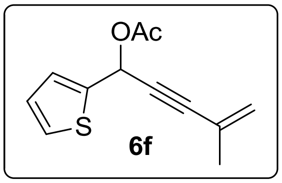

Colorless liquid (0.556 g, 2.52 mmol, 90%); ^{1}H NMR (400 MHz, $CDCl_3$): δ 7.30 (d, *J* = 5.2 Hz, 1H), 7.22 ~ 7.21 (m, 1H), 6.96 ~ 6.94 (m, 1H), 6.79 (s, 1H), 5.38 (s, 1H), 5.29 (s, 1H), 2.06 (s, 3H), 1.89 (s, 3H); ^{13}C NMR (100 MHz, $CDCl_3$): δ 169.4, 140.0, 127.5, 126.8, 126.5, 125.5, 123.4, 87.5, 83.8, 60.8, 22.9, 20.8; HRMS (ESI, m/z) calcd for $C_{12}H_{12}NaO_2S$ [M+Na]: 243.0456, found: 243.0430.

Spectral data for 2,6-dimethylhept-6-en-4-yn-3-yl acetate (6g):

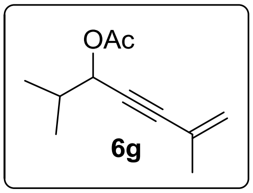

Colorless liquid (0.586 g, 3.2559 mmol, 90%); ^{1}H NMR (400 MHz, $CDCl_3$): δ 5.30 (d, *J* = 5.6 Hz, 1H), 5.26 (s, 1H), 5.19 (t, *J* = 1.6 Hz, 1H), 2.04 (s, 3H), 1.98 ~ 1.93 (m, 1H), 1.83 (s, 3H), 0.97 (d, *J* = 6.8 Hz, 3H), 0.95 (d, *J* = 6.8 Hz, 3H); ^{13}C NMR (100 MHz, $CDCl_3$): δ 170.0, 126.0, 122.5, 86.9, 84.2, 69.2, 32.5, 23.2, 20.9, 18.2, 17.4; HRMS (ESI, m/z) calcd for $C_{11}H_{16}NaO_2$ [M+Na]: 203.1048, found: 203.1026.

Spectral data for 4-methylpent-4-en-2-yn-1-yl acetate (6h):

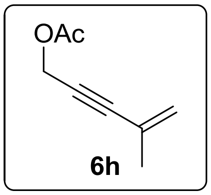

Colorless liquid (0.895 g, 6.48 mmol, 85%); ^{1}H NMR (400 MHz, $CDCl_3$): δ 5.31 (s, 1H), 5.24 (d, *J* = 1.6 Hz, 1H), 4.76 (s, 2H), 2.07 (s, 3H), 1.86 (s, 3H); ^{13}C NMR (100 MHz, $CDCl_3$): δ 170.3, 125.9, 123.0, 87.6, 81.8, 52.7, 23.1, 20.7; HRMS (ESI+, m/z) calcd for $C_8H_{10}NaO_2$ [M+Na]: 161.0578, found: 161.0579.

Spectral data for (9*S*,9a*S*)-9-hydroxy-2-methyl-9a-phenyl-9,9a-dihydro-1*H*-cyclopenta[*b*]quinolin-3-yl acetate (7a):

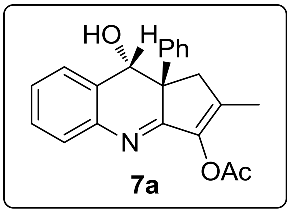

White solid, mp: 208-209 ˚C (0.115 g, 0.345 mmol, 74%); ^{1}H NMR (400 MHz, $CDCl_3$): δ 7.17 ~ 7.16 (m, 2H), 7.09 ~ 7.07 (m, 2H), 7.02 ~ 7.0 (m, 1H), 6.96 ~ 6.95 (m, 1H), 6.85 ~ 6.80 (m, 2H), 6.65 ~ 6.64 (m, 1H), 4.71 (s, 1H), 3.78 (dd, *J* = 15.2, 1.1 Hz, 1H), 2.59 (dd, *J* = 15.6, 0.8 Hz, 1H), 2.36 (s, 3H), 1.84 (s, 3H); ^{13}C NMR (100 MHz, $CDCl_3$): δ 173.2, 167.9,

150.0, 143.0, 141.9, 140.8, 129.1, 129.0, 128.3, 126.8, 126.7, 126.5, 126.1, 124.9, 72.9, 53.1, 42.0, 20.5, 14.4; HRMS (EI+, m/z) calcd for $C_{21}H_{20}NO_3$ [M+H]: 334.1443, found: 334.1437.

Spectral data for (9*S*,9a*S*)-2-methyl-9a-phenyl-9,9a-dihydro-1*H*-cyclopenta[*b*]quinoline-3,9-diol (7a'):

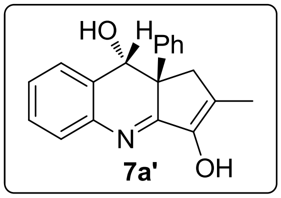

Brown solid, mp: 163-164 ˚C (0.113 g, 0.38 mmol, 80%); ^{1}H NMR (400 MHz,$(CD_3)_2CO$): δ 7.24 ~ 7.21 (m, 2H), 7.14 ~ 7.07 (m, 2H), 7.03 ~ 7.0 (m, 4H), 6.92 ~ 6.88 (m, 1H), 4.93 (s, 1H), 3.61 (dd, *J* = 14.4, 1.7 Hz, 1H), 2.90 (bs, 2H), 2.46 (d, *J* = 16.2 Hz, 1H), 1.92 (s, 3H); ^{13}C NMR (100 MHz, $(CD_3)_2CO$): δ 176.5, 149.0, 144.4, 143.1, 136.1, 130.1, 129.8, 129.1, 129.0, 127.7, 127.5, 126.7, 126.0, 73.1, 52.2, 42.2, 13.7; HRMS (EI+, m/z) calcd for $C_{19}H_{18}NO_2$ [M+H]: 292.1338, found: 292.1331.

Spectral data for (9*S*,9a*S*)-9a-(4-chlorophenyl)-9-hydroxy-2-methyl-9,9a-dihydro-1*H*-cyclopenta[*b*]quinolin-3-yl acetate (7b):

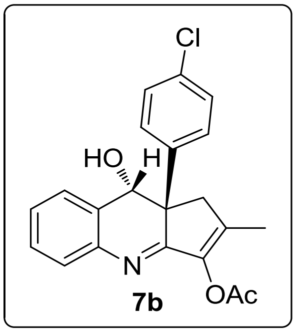

White solid, mp: 216-217 ˚C (0.099 g, 0.269 mmol, 67%); ^{1}H NMR (400 MHz, $CDCl_3$): δ 7.12 (d, *J* = 8.4 Hz, 2H), 7.06 (d, *J* = 8.7 Hz, 2H), 6.97 (d, *J* = 7.2 Hz, 1H), 6.94 ~ 6.86 (m, 2H), 6.83 (d, *J* = 7.4 Hz, 1H), 4.68 (s, 1H), 4.48 (bs, 1H), 3.73 (d, *J* = 16.8 Hz, 1H), 2.53 (d, *J* = 16.6 Hz, 1H), 2.36 (s, 3H), 1.85 (s, 3H); ^{13}C NMR (100 MHz, $CDCl_3$): δ 172.8, 168.0, 150.3, 143.1, 142.0, 139.2, 132.7, 129.6, 129.1, 128.6, 128.3, 126.6, 126.2, 125.4, 72.8, 52.4, 42.0, 20.6, 14.5; HRMS (EI+, m/z) calcd for $C_{21}H_{18}ClNO_3$ [M+H]: 368.1053, found: 368.1058.

Spectral data for (9S,9aS)-9a-(4-bromophenyl)-9-hydroxy-2-methyl-9,9a-dihydro-1*H*-cyclopenta[*b*]quinolin-3-yl acetate (7c):

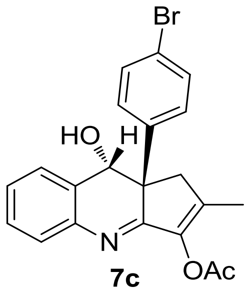

7c

Yellow solid, mp: 189-190 ˚C (0.093 g, 0.226 mmol, 66%); ^{1}H NMR (400 MHz, $CDCl_3$): δ 7.21 (d, *J* = 8.4 Hz, 2H),7.05 (d, *J* = 8.4 Hz, 2H), 6.97 ~ 6.96 (m, 1H), 6.91 ~ 6.85 (m, 2H), 6.75 (d, *J* = 6.2 Hz, 1H), 4.88 (bs, 1H), 4.66 (s, 1H), 3.74 (d, *J* = 16.4 Hz, 1H), 2.52 (d, *J* = 16.8 Hz, 1H),2.35 (s, 3H), 1.84 (s, 3H); ^{13}C NMR (100 MHz, $CDCl_3$): δ 172.8, 168.0, 150.2, 143.0, 141.9, 139.9, 131.5, 129.5, 129.2, 128.7, 126.5, 126.2, 125.3, 120.8, 72.8, 52.6, 41.9, 20.5, 14.5; HRMS (EI+, m/z) calcd for $C_{21}H_{19}BrNO_3$ [M+H]: 412.0548, found: 412.0541.

Spectral data for (9*S*,9a*S*)-9-hydroxy-2-methyl-9a-(*p*-tolyl)-9,9a-dihydro-1*H*-cyclopenta[*b*]quinolin-3-yl acetate (7d):

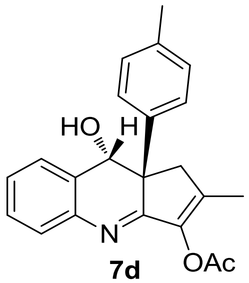

7d

White solid, mp: 211-212 ˚C (0.108 g, 0.311 mmol, 71%); ^{1}H NMR (400 MHz, $CDCl_3$): δ 7.05 (d, *J* = 8.0 Hz, 2H), 6.96 (t, *J* = 8.4 Hz, 1H), 6.89 (d, *J* = 7.9 Hz, 2H), 6.83 (t, *J* = 3.6 Hz, 2H), 6.71 (t, *J* = 2.9 Hz, 1H), 4.69 (s, 1H), 3.75 (d, *J* = 16.4 Hz, 1H), 2.57 (d, *J* = 16.4 Hz, 1H), 2.35 (s, 3H), 2.12 (s, 3H), 1.83 (s, 3H); ^{13}C NMR (100 MHz, $CDCl_3$): δ 173.5, 168.0, 150.1, 143.1, 142.2, 137.7, 136.4, 129.3, 129.1, 126.7, 126.6, 126.2, 125.3, 73.0, 52.7, 42.1, 20.8, 20.6, 14.5; HRMS (EI+, m/z) calcd for $C_{22}H_{22}NO_3$ [M+H]: 348.1600, found: 348.1597.

Spectral data for (9*S*,9a*S*)-9-hydroxy-9a-(4-methoxyphenyl)-2-methyl-9,9a-dihydro-1*H*-cyclopenta[*b*]quinolin-3-yl acetate (7e):

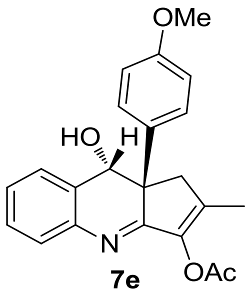

7e

White solid, mp: 204-205 ˚C (0.109 g, 0.300 mmol, 73%); ^{1}H NMR (400 MHz, $CDCl_3$): δ 7.07 (d, *J* = 8.8 Hz, 2H), 6.98 ~ 6.96 (m, 1H), 6.85 (t, *J* = 3.8 Hz, 2H), 6.73 ~ 6.71 (m, 1H), 6.61 (d, *J* = 8.8 Hz, 2H), 4.68 (s, 1H), 3.73 (d, *J* = 16.5 Hz, 1H), 3.60 (s, 3H), 2.55 (d, *J* = 16.5 Hz, 1H), 2.35 (s, 3H), 1.84 (s, 3H); ^{13}C NMR (100 MHz, $CDCl_3$): δ 173.6, 168.1, 158.2, 150.5, 143.0, 141.9, 132.5, 129.3, 129.1, 127.9, 126.6, 126.3, 125.2, 113.8, 73.0, 55.0, 52.4, 42.1, 20.6, 14.6; HRMS (EI+, m/z) calcd for $C_{22}H_{22}NO_4$ [M+H]: 364.1549, found: 364.1545.

Spectral data for (9*S*,9a*S*)-9-hydroxy-2-methyl-9a-(thiophen-2-yl)-9,9a-dihydro-1*H*-cyclopenta[*b*]quinolin-3-yl acetate (7f):

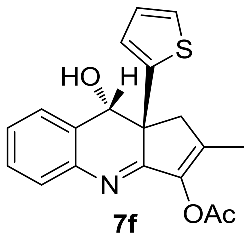

7f

White solid, mp: 198-199 ˚C (0.114 g, 0.336 mmol, 74%); ^{1}H NMR (600 MHz, $CDCl_3$): δ 7.09 (d, *J* = 7.2 Hz, 1H), 6.96 (d, *J* = 5.0 Hz, 1H), 6.91 (t, *J* = 7.4 Hz, 1H), 6.86 (t, *J* = 7.4 1H), 6.76 (d, *J* = 3.2 Hz, 1H), 6.67 (d, *J* = 7.6 Hz, 1H), 6.60 (t, *J* = 4.2 Hz, 1H), 5.19 (s, 1H), 4.76 (s, 1H), 3.71 (d, *J* = 16.4 Hz, 1H), 2.59 (d, *J* = 16.4 Hz, 1H), 2.35 (s, 3H), 1.88 (s, 3H); ^{13}C NMR (150 MHz, $CDCl_3$): δ 172.3, 168.0, 150.3, 144.4, 142.8, 141.6, 129.4, 129.4, 126.6, 126.5, 126.3, 125.5, 125.0, 124.0, 72.7, 49.8, 42.5, 20.6, 14.6; HRMS (EI+, m/z) calcd for $C_{19}H_{18}NO_3S$ [M+H]: 340.1007, found: 340.1003.

Spectral data for (9*S*,9a*S*)-9-hydroxy-9a-isopropyl-2-methyl-9,9a-dihydro-1*H*-cyclopenta[*b*]quinolin-3-yl acetate (7g):

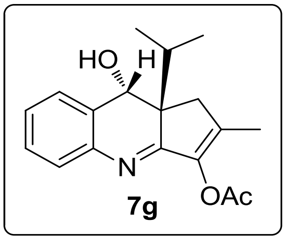

White solid, mp: 194-195 ˚C (0.102 g, 0.341 mmol, 61%); ^{1}H NMR (400 MHz, $CDCl_3$): δ 7.15 (d, *J* = 6.8 Hz, 1H), 7.07 (t, *J* = 6.9 Hz, 1H), 7.02 (t, *J* = 7.2 Hz, 1H), 6.95 (d, *J* = 7.3 Hz, 1H), 4.44 (s, 1H), 3.0 (d, *J* = 17.2 Hz, 1H), 2.32 (d, *J* = 12.4 Hz, 4H), 1.91 (s, 3H), 1.63 ~ 1.59 (m, 1H), 0.81 (d, *J* = 6.4 Hz, 3H), 0.77 (d, *J* = 6.4 Hz, 3H); ^{13}C NMR (150 MHz, $CDCl_3$): δ 176.8, 168.1, 152.2, 144.2, 142.0, 129.7, 128.9, 126.6, 126.1, 125.7, 70.2, 50.7, 32.0, 28.8, 20.5, 18.8, 17.2, 14.4; HRMS (EI+, m/z) calcd for $C_{18}H_{22}NO_3$ [M+H]: 300.1600, found: 300.1591.

Spectral data for (9*S*,9a*S*)-2,3,7-trimethyl-9a-phenyl-9,9a-dihydro-1*H*-cyclopenta[*b*]quinolin-9-ol (8b):

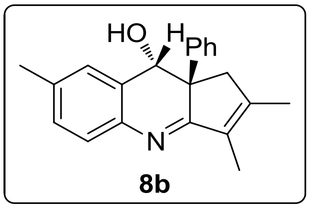

Pale yellow solid, mp: 199-200 ˚C (0.150 g, 0.494 mmol, 84%); ^{1}H NMR (400 MHz, $CDCl_3$): δ 7.09 ~ 6.99 (m, 5H), 6.90 (d, *J* = 8.0 Hz, 1H), 6.78 (d, *J* = 8.0 Hz, 1H), 6.76 (s, 1H), 4.68 (s, 1H), 3.64 (d, *J* = 16.8 Hz, 1H), 2.52 (d, *J* = 16.8 Hz, 1H), 2.15 (s, 3H), 1.84 (s, 3H), 1.70 (s, 3H); ^{13}C NMR (100 MHz, $CDCl_3$): δ 181.3, 157.8, 141.9, 141.2, 135.4, 133.8, 129.9, 129.6, 128.2, 126.8, 126.5, 126.4, 125.5, 73.3, 54.7, 45.3, 20.9, 16.2, 8.9; HRMS (EI+, m/z) calcd for $C_{21}H_{21}NO$ [M^+]: 303.1623, found: 303.1621.

Spectral data for (9*S*,9a*S*)-7-methoxy-2,3-dimethyl-9a-phenyl-9,9a-dihydro-1*H*-cyclopenta[*b*]quinolin-9-ol (8c):

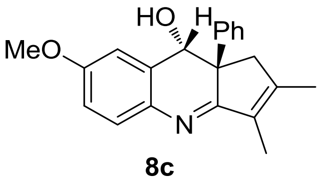

8c

White solid, mp: 193-194 ˚C (0.157 g, 0.49 mmol, 84%); ^{1}H NMR (400 MHz, $CDCl_3$): δ 7.08 ~ 6.93 (m, 5H), 6.94 (d, *J* = 9.1 Hz, 1H), 6.51 ~ 6.50 (m, 2H), 4.76 (s, 1H), 3.69 ~ 3.61 (m, 4H), 2.52 (d, *J* = 16.7 Hz, 1H), 1.86 (s, 3H), 1.76 (s, 3H); ^{13}C NMR (100 MHz, $CDCl_3$): δ 180.3, 157.3, 141.8, 137.1, 133.7, 128.3, 127.9, 126.9, 126.6, 126.5, 114.9, 113.6, 73.5, 55.2, 54.7, 45.3, 16.2, 8.9; HRMS (EI+, m/z) calcd for $C_{21}H_{22}NO_2$ [M+H]: 320.1651, found: 320.1648.

Spectral data for (9*S*,9a*S*)-7-bromo-2,3-dimethyl-9a-phenyl-9,9a-dihydro-1*H*-cyclopenta[*b*]quinolin-9-ol (8d):

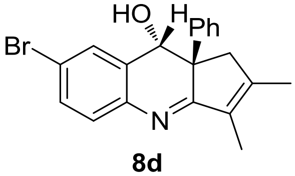

8d

Pale yellow solid, mp: 215-216 ˚C (0.195 g, 0.529 mmol, 90%); ^{1}H NMR (400 MHz, $CDCl_3$): δ 7.08 ~ 7.01 (m, 5H), 6.94 (d, *J* = 6.8, Hz, 2H), 6.68 (d, *J* = 8.4 Hz, 1H), 5.32 (bs, 1H), 4.68 (s, 1H), 3.68 (d, *J* = 16.8 Hz, 1H), 2.54 (d, *J* = 17.2 Hz, 1H), 1.87 (s, 3H), 1.70 (s, 3H); ^{13}C NMR (100 MHz, $CDCl_3$): δ 183.1, 160.1, 142.1, 141.0, 133.8, 131.9, 131.9, 128.6, 128.4, 126.9, 126.8, 126.7, 118.6, 72.6, 55.0, 45.4, 16.4, 8.8; HRMS (EI+, m/z) calcd for $C_{20}H_{18}BrNO$ [M^+]: 367.0572, found: 367.0574.

Spectral data for (9*S*,9a*S*)-7-chloro-2,3-dimethyl-9a-phenyl-9,9a-dihydro-1*H*-cyclopenta[*b*]quinolin-9-ol (8e):

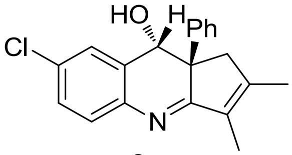

8e

Pale yellow solid, mp: 198-199 ˚C (0.162 g, 0.500 mmol, 85%); ^{1}H NMR (400 MHz, $CDCl_3$): δ 7.09 ~ 7.01 (m, 3H), 6.96 ~ 6.93 (m, 3H), 6.90 (dd, *J* = 8.4, 2.4 Hz, 1H), 6.75 (d, *J* = 8.4 Hz, 1H), 5.19 (bs, 1H), 4.68 (s, 1H), 3.68 (d, *J* = 16.8 Hz, 1H), 2.54 (d, *J* = 16.8 Hz, 1H), 1.88 (s, 3H), 1.71 (s, 3H); ^{13}C NMR (100 MHz, $CDCl_3$): δ 183.0, 159.9, 141.7, 141.1, 133.8, 130.6, 129.1, 128.9, 128.4, 128.3, 126.9, 126.7, 126.5, 72.7, 54.9, 45.4, 16.3, 8.8; HRMS (EI+, m/z) calcd for $C_{20}H_{18}ClNO$ [M^+]: 323.1077, found: 323.1073.

Spectral data for ethyl ((9*S*,9a*S*)-9-hydroxy-2,3-dimethyl-9a-phenyl-9,9a-dihydro-1*H*-cyclopenta[*b*]quinolin-7-yl) carbonate (8f):

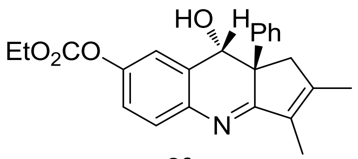

8f

White solid, mp: 195-196 ˚C (0.177 g, 0.47 mmol, 80%); ^{1}H NMR (400 MHz, $CDCl_3$): δ 7.13 (d, *J* = 8.6 Hz, 1H), 7.10 ~ 7.0 (m, 5H), 6.91 (dd, *J* = 5.9, 2.6 Hz, 1H), 6.81 (d, *J* = 2.6 Hz, 1H), 4.74 (s, 1H), 4.25 ~ 4.20 (q, 2H), 3.62 (d, *J* = 16.9 Hz, 1H), 2.55 (d, *J* = 16.9 Hz, 1H), 1.90 (s, 3H), 1.86 (s, 3H), 1.31 (t, *J* = 7.1 Hz, 3H); ^{13}C NMR (100 MHz, $CDCl_3$): δ 182.2, 159.1, 153.4, 148.4, 141.7, 141.1, 134.0, 128.4, 127.7, 126.9, 126.8, 126.7, 121.8, 121.3, 73.0, 64.7, 53.8, 45.3, 16.4, 14.2, 9.0; HRMS (EI+, m/z) calcd for $C_{23}H_{24}NO_4$ [M+H]: 378.1705, found: 378.1709.

Spectral data for (9*S*,9a*S*)-2,3,6-trimethyl-9a-phenyl-9,9a-dihydro-1*H*-cyclopenta[*b*]quinolin-9-ol (8g):

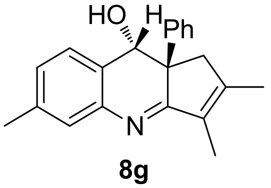

8g

Pale yellow solid, mp: 192-193 ˚C (0.158 g, 0.521 mmol, 88%); ^{1}H NMR (400 MHz, $CDCl_3$): δ 7.06 ~ 6.96 (m, 5H), 6.81 (d, *J* = 7.6 Hz, 1H), 6.60 (d, *J* = 6.4 Hz, 2H), 5.01 (bs, 1H), 4.70 (s, 1H), 3.71 (dd, *J* = 16.8, 1.6 Hz, 1H), 2.54 (d, *J* = 16.8 Hz, 1H), 2.05 (s, 3H), 1.87 (s, 3H),

1.77 (s, 3H); ^{13}C NMR (100 MHz, $CDCl_3$): δ 182.2, 158.4, 143.0, 141.9, 138.5, 133.9, 128.5, 128.2, 126.7, 126.5, 126.4, 126.1, 123.9, 72.9, 55.1, 45.5, 21.1, 16.3, 8.9; HRMS (EI+, m/z) calcd for $C_{21}H_{21}NO$ [M^+]: 303.1623, found: 303.1625.

Spectral data for (9*S*,9a*S*)-6-bromo-2,3-dimethyl-9a-phenyl-9,9a-dihydro-1*H*-cyclopenta[*b*]quinolin-9-ol (8h):

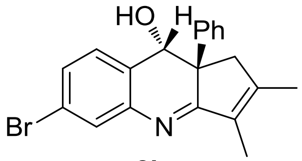

8h

Yellow solid, mp: 200-201 ˚C (0.159 g, 0.43 mmol, 86%); ^{1}H NMR (400 MHz, $CDCl_3$): δ 7.09 ~ 7.01 (m, 5H), 6.97 ~ 6.95 (m, 2H), 6.84 (d, *J* = 7.9 Hz, 1H), 4.73 (s, 1H), 3.68 (d, *J* = 16.9 Hz, 1H), 2.56 (d, *J* = 16.9 Hz, 1H), 1.92 (s, 3H), 1.78 (s, 3H); ^{13}C NMR (100 MHz, $CDCl_3$): δ 183.8, 160.7, 144.6, 141.1, 133.8, 130.1, 129.1, 128.4, 128.3, 126.8, 126.6, 125.8, 122.6, 72.6, 55.0, 45.5, 16.5, 8.9; HRMS (EI+, m/z) calcd for $C_{20}H_{19}BrNO$ [M+H]: 368.0650, found: 368.0658.

Spectral data for (9*S*,9a*S*)-6-chloro-2,3-dimethyl-9a-phenyl-9,9a-dihydro-1*H*-cyclopenta[*b*]quinolin-9-ol (8i):

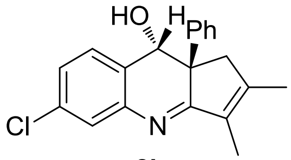

8i

Pale yellow solid, mp: 231-232 ˚C (0.175 g, 0.54 mmol, 92%); ^{1}H NMR (400 MHz, $CDCl_3$): δ 7.08 ~ 7.00 (m, 3H), 6.95 ~ 6.83 (m, 4H), 6.78 (d, *J* = 1.6 Hz, 1H), 5.09 (bs, 1H), 4.72 (s, 1H), 3.72 (d, *J* = 17.2 Hz, 1H), 2.56 (d, *J* = 17.2 Hz, 1H), 1.91 (s, 3H), 1.71 (s, 3H); ^{13}C NMR (100 MHz, $CDCl_3$): δ 183.9, 160.8, 144.2, 141.2, 134.2, 133.8, 129.8, 128.4, 126.8, 126.6, 126.1, 125.4, 125.3, 72.6, 55.3, 45.5, 16.5, 8.9; HRMS (EI+, m/z) calcd for $C_{20}H_{18}ClNO$ [M^+]: 323.1077, found: 323.1072.

Spectral data for (9*S*,9a*S*)-2,3,9-trimethyl-9a-phenyl-9,9a-dihydro-1*H*-cyclopenta[*b*]quinolin-9-ol (8j):

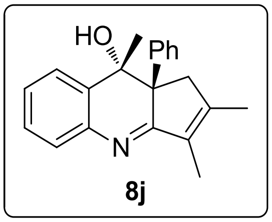

White solid, mp: 179-180 °C (0.156 g, 0.52 mmol, 82%); ^{1}H NMR (400 MHz, $CDCl_3$): δ 7.25 (t, *J* = 7.6 Hz, 1H), 7.15 (t, *J* = 7.3 Hz, 1H), 7.07 ~ 7.06 (m, 3H), 7.05 ~ 7.03 (m, 3H), 6.94 (t, *J* = 7.2 Hz, 1H), 3.56 (d, *J* = 16.4 Hz, 1H), 3.15 (s, 1H), 2.85 (d, *J* = 16.5 Hz, 1H), 1.90 (s, 3H), 1.75 (s, 3H), 1.67 (s, 3H); ^{13}C NMR (100 MHz, $CDCl_3$): δ 183.5, 158.6, 144.2, 138.4, 133.8, 130.8, 128.8, 128.8, 127.5, 126.7, 125.9, 125.8, 124.0, 72.1, 57.4, 43.5, 22.1, 16.3, 8.8; HRMS (EI+, m/z) calcd for $C_{21}H_{22}NO$ [M+H]: 304.1701, found: 304.1715.

^{1}H NOE of Compound (9*S*,9a*S*)-2,3,9-trimethyl-9a-phenyl-9,9a-dihydro-1*H*-cyclopenta[*b*]quinolin-9-ol (8j):

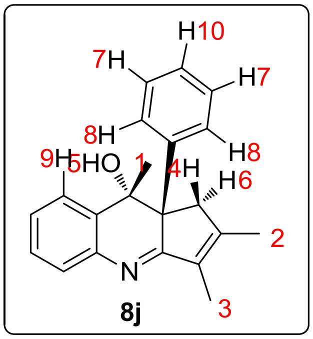

Sr. No.	Irradiation	Intensity Increase % (Key peaks)
1	$CH_3$1 (δ 1.67)	H6 (δ 3.56, 1.79%), H78 (δ 7.07 ~ 7.06, 8.71%), H9 (δ 7.25, 0.90%)
2	H4 (δ 2.85)	H6 (δ 3.56, 28.16%), H7 (δ 7.05 ~ 7.03, 16.77%)
3	OH5 (δ 3.15)	H6 (δ 3.56, 2.07%, H8 (δ 7.07 ~ 7.06, 2.55%), H9 (δ 7.25, 3.68%)
4	H6 (δ 3.56)	H4 (δ 2.85, 22.63%), H9 (δ 7.25, 0.94%)
5	H8 (δ 7.07 ~ 7.06)	$CH_3$1 (δ 1.67, 3.06%), H4 (δ 2.85, 2.76%), H10 (δ 6.94, 2.66%)

Spectral data for (*Z*)-4-((4,5-dimethyl-2-phenylcyclopenta-1,4-dien-1-yl)amino)pent-3-en-2-one (9a):

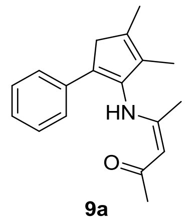

9a

Brown oil (0.113 g, 0.423 mmol, 72%); ^{1}H NMR (400 MHz, $CDCl_3$): δ 11.99 (bs, 1H), 7.40 (d, *J* = 8.4 Hz, 2H), 7.26 (t, *J* = 7.6 Hz, 2H), 7.12 (t, *J* = 7.2 Hz, 1H), 5.14 (s, 1H), 3.27 (s, 2H), 2.10 (s, 3H), 1.98 (s, 3H), 1.76 (s, 3H), 1.60 (s, 3H); ^{13}C NMR (100 MHz, $CDCl_3$): δ 196.1, 162.0, 137.9, 135.7, 135.4, 134.9, 132.4, 128.6, 126.3, 125.9, 96.8, 44.4, 29.1, 19.0, 13.9, 10.1; HRMS (ESI, m/z) calcd for $C_{18}H_{21}NaNO$ [M+]: 290.1521, found: 290.1526.

Spectral data for (*Z*)-4-((2-(4-bromophenyl)-4,5-dimethylcyclopenta-1,4-dien-1-yl)amino)pent-3-en-2-one (9b):

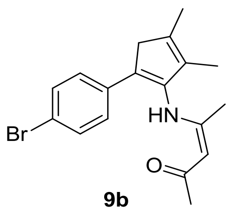

9b

Pale yellow solid, mp: 146-147 ˚C (0.089 g, 0.26 mmol, 64%); ^{1}H NMR (400 MHz, $CDCl_3$): δ 11.96 (bs, 1H), 7.36 (d, *J* = 8.0 Hz, 2H), 7.25 (d, *J* = 8.8 Hz, 2H), 5.15 (s, 1H), 3.22 (s, 2H), 2.1 (s, 3H), 1.96 (s, 3H), 1.74 (s, 3H), 1.59 (s, 3H); ^{13}C NMR (100 MHz, $CDCl_3$): δ 196.4, 161.7, 138.8, 136.2, 135.1, 134.3, 131.7, 131.2, 127.4, 119.9, 97.1, 44.2, 29.1, 19.1, 14.0, 10.1; HRMS (EI+, m/z) calcd for $C_{18}H_{20}BrNO$ [M^+]: 345.0728, found: 345.0724.

(5) (a) X-ray crystallographic structure and data for compound (5a)

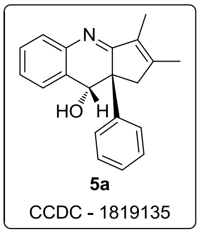

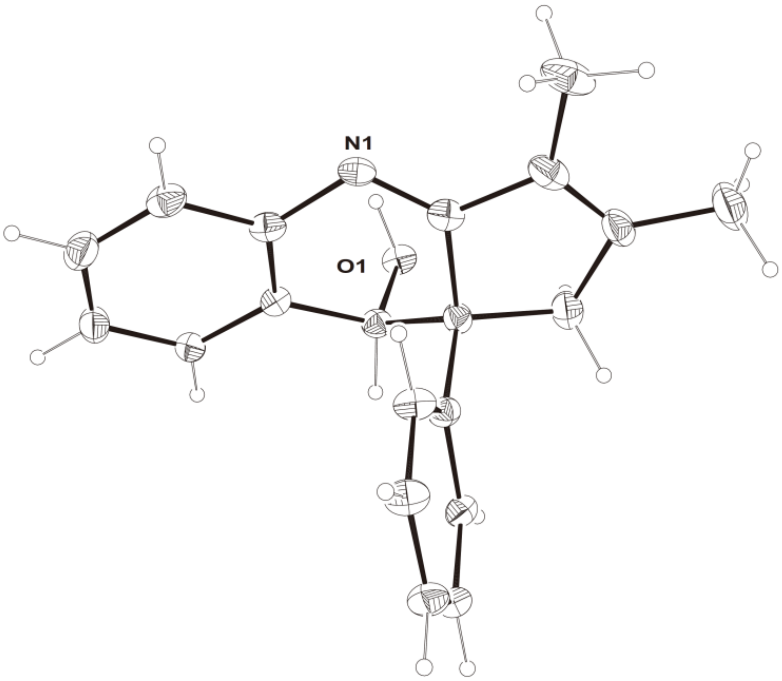

Table S1. Crystal data and structure refinement for d19364.

Identification code	d19364	
Empirical formula	C20 H19 N O	
Formula weight	289.36	
Temperature	200(2) K	
Wavelength	0.71073 Å	
Crystal system	Monoclinic	
Space group	P 21/c	
Unit cell dimensions	a = 9.5578(12) Å	α= 90°.
	b = 20.653(2) Å	β= 108.514(6)°.
	c = 8.4644(10) Å	γ = 90°.
Volume	1584.4(3) Å ^{3}	
Z	4	
Density (calculated)	1.213 Mg/m^3	
Absorption coefficient	0.074 mm^{-1}	
F(000)	616	
Crystal size	0.22 x 0.21 x 0.08 mm^3	
Theta range for data collection	2.25 to 25.04°.	
Index ranges	-11<=h<=10, -24<=k<=24, -9<=l<=10	
Reflections collected	21837	
Independent reflections	2789 [R(int) = 0.0500]	
Completeness to theta = 25.04°	99.5 %	
Absorption correction	multi-scan	
Max. and min. transmission	0.9941 and 0.9839	
Refinement method	Full-matrix least-squares on F^2	
Data / restraints / parameters	2789 / 0 / 202	
Goodness-of-fit on F^2	1.049	
Final R indices [I>2sigma(I)]	R1 = 0.0687, wR2 = 0.1679	
R indices (all data)	R1 = 0.0953, wR2 = 0.1948	
Extinction coefficient	0.016(5)	
Largest diff. peak and hole	0.525 and -0.196 e.Å $^{-3}$	

Table S2. Atomic coordinates (x 10^4) and equivalent isotropic displacement parameters (Å ^{2}x 10^3) for d19364. U(eq) is defined as one third of the trace of the orthogonalized U^{ij} tensor.

	x	y	z	U(eq)
C(1)	1904(3)	5191(1)	4632(4)	39(1)
C(2)	2152(3)	5011(1)	3025(3)	39(1)
C(3)	1218(3)	4609(1)	1866(4)	45(1)
C(4)	1478(4)	4432(2)	418(4)	59(1)
C(5)	2726(4)	4671(2)	110(4)	63(1)
C(6)	3678(4)	5068(2)	1230(4)	54(1)
C(7)	3404(3)	5252(1)	2715(4)	43(1)
C(8)	3975(3)	5954(1)	4948(4)	44(1)
C(9)	4825(4)	6374(2)	6264(5)	57(1)
C(10)	6289(4)	6633(2)	6327(6)	77(1)
C(11)	4077(4)	6456(2)	7389(4)	60(1)
C(12)	4600(5)	6821(2)	8961(6)	95(2)
C(13)	2641(4)	6107(2)	6863(4)	54(1)
C(14)	2416(3)	5893(1)	5049(3)	39(1)
C(15)	1346(3)	6347(1)	3783(4)	40(1)
C(16)	1756(3)	6714(1)	2635(4)	48(1)
C(17)	784(3)	7138(2)	1576(4)	54(1)
C(18)	-639(4)	7197(2)	1624(4)	55(1)
C(19)	-1085(3)	6825(2)	2720(4)	49(1)
C(20)	-110(3)	6399(1)	3787(4)	42(1)
N(1)	4436(2)	5662(1)	3868(3)	41(1)
O(1)	2626(2)	4757(1)	5970(2)	43(1)

Table S3. Bond lengths [Å] and angles [°] for d19364.

C(1)-O(1)	1.438(3)
C(1)-C(2)	1.500(4)
C(1)-C(14)	1.534(4)
C(1)-H(1)	1.0000
C(2)-C(3)	1.376(4)
C(2)-C(7)	1.395(4)
C(3)-C(4)	1.376(4)
C(3)-H(3)	0.9500
C(4)-C(5)	1.390(5)
C(4)-H(4)	0.9500
C(5)-C(6)	1.361(5)
C(5)-H(5)	0.9500
C(6)-C(7)	1.415(4)
C(6)-H(6)	0.9500
C(7)-N(1)	1.425(4)
C(8)-N(1)	1.285(4)
C(8)-C(9)	1.441(4)
C(8)-C(14)	1.525(4)
C(9)-C(11)	1.371(5)
C(9)-C(10)	1.484(5)
C(10)-H(10A)	0.9800
C(10)-H(10B)	0.9800
C(10)-H(10C)	0.9800
C(11)-C(12)	1.472(5)
C(11)-C(13)	1.487(5)
C(12)-H(12A)	0.9800
C(12)-H(12B)	0.9800
C(12)-H(12C)	0.9800
C(13)-C(14)	1.546(4)
C(13)-H(13A)	0.9900
C(13)-H(13B)	0.9900
C(14)-C(15)	1.542(4)
C(15)-C(16)	1.383(4)
C(15)-C(20)	1.398(4)

C(16)-C(17)	1.380(4)
C(16)-H(16)	0.9500
C(17)-C(18)	1.380(4)
C(17)-H(17)	0.9500
C(18)-C(19)	1.372(5)
C(18)-H(18)	0.9500
C(19)-C(20)	1.387(4)
C(19)-H(19)	0.9500
C(20)-H(20)	0.9500
O(1)-H(1')	0.8885

O(1)-C(1)-C(2)	112.6(2)
O(1)-C(1)-C(14)	111.7(2)
C(2)-C(1)-C(14)	108.2(2)
O(1)-C(1)-H(1)	108.1
C(2)-C(1)-H(1)	108.1
C(14)-C(1)-H(1)	108.1
C(3)-C(2)-C(7)	118.8(3)
C(3)-C(2)-C(1)	122.8(3)
C(7)-C(2)-C(1)	118.3(3)
C(4)-C(3)-C(2)	122.4(3)
C(4)-C(3)-H(3)	118.8
C(2)-C(3)-H(3)	118.8
C(3)-C(4)-C(5)	118.8(3)
C(3)-C(4)-H(4)	120.6
C(5)-C(4)-H(4)	120.6
C(6)-C(5)-C(4)	120.5(3)
C(6)-C(5)-H(5)	119.8
C(4)-C(5)-H(5)	119.8
C(5)-C(6)-C(7)	120.6(3)
C(5)-C(6)-H(6)	119.7
C(7)-C(6)-H(6)	119.7
C(2)-C(7)-C(6)	118.9(3)
C(2)-C(7)-N(1)	122.1(3)
C(6)-C(7)-N(1)	119.0(3)
N(1)-C(8)-C(9)	126.7(3)

N(1)-C(8)-C(14)	124.2(3)
C(9)-C(8)-C(14)	109.1(3)
C(11)-C(9)-C(8)	108.7(3)
C(11)-C(9)-C(10)	128.7(3)
C(8)-C(9)-C(10)	122.6(4)
C(9)-C(10)-H(10A)	109.5
C(9)-C(10)-H(10B)	109.5
H(10A)-C(10)-H(10B)	109.5
C(9)-C(10)-H(10C)	109.5
H(10A)-C(10)-H(10C)	109.5
H(10B)-C(10)-H(10C)	109.5
C(9)-C(11)-C(12)	126.0(4)
C(9)-C(11)-C(13)	111.6(3)
C(12)-C(11)-C(13)	122.3(4)
C(11)-C(12)-H(12A)	109.5
C(11)-C(12)-H(12B)	109.5
H(12A)-C(12)-H(12B)	109.5
C(11)-C(12)-H(12C)	109.5
H(12A)-C(12)-H(12C)	109.5
H(12B)-C(12)-H(12C)	109.5
C(11)-C(13)-C(14)	105.1(3)
C(11)-C(13)-H(13A)	110.7
C(14)-C(13)-H(13A)	110.7
C(11)-C(13)-H(13B)	110.7
C(14)-C(13)-H(13B)	110.7
H(13A)-C(13)-H(13B)	108.8
C(8)-C(14)-C(1)	108.2(2)
C(8)-C(14)-C(15)	110.7(2)
C(1)-C(14)-C(15)	108.8(2)
C(8)-C(14)-C(13)	101.6(2)
C(1)-C(14)-C(13)	115.7(2)
C(15)-C(14)-C(13)	111.6(2)
C(16)-C(15)-C(20)	117.6(3)
C(16)-C(15)-C(14)	123.3(2)
C(20)-C(15)-C(14)	119.1(2)
C(17)-C(16)-C(15)	121.5(3)

C(17)-C(16)-H(16)	119.3
C(15)-C(16)-H(16)	119.3
C(18)-C(17)-C(16)	120.2(3)
C(18)-C(17)-H(17)	119.9
C(16)-C(17)-H(17)	119.9
C(19)-C(18)-C(17)	119.4(3)
C(19)-C(18)-H(18)	120.3
C(17)-C(18)-H(18)	120.3
C(18)-C(19)-C(20)	120.5(3)
C(18)-C(19)-H(19)	119.8
C(20)-C(19)-H(19)	119.8
C(19)-C(20)-C(15)	120.7(3)
C(19)-C(20)-H(20)	119.6
C(15)-C(20)-H(20)	119.6
C(8)-N(1)-C(7)	116.6(2)
C(1)-O(1)-H(1')	105.6

Symmetry transformations used to generate equivalent atoms:

Table S4. Anisotropic displacement parameters (Å ^{2}x 10^3)for d19364. The anisotropic displacement factor exponent takes the form: $-2\pi^2$[$h^2a^{*2}U^{11}$ + ... + 2 h k a* b* U^{12}]

	U^{11}	U^{22}	U^{33}	U^{23}	U^{13}	U^{12}
C(1)	35(1)	40(2)	44(2)	0(1)	15(1)	-3(1)
C(2)	39(1)	37(1)	42(2)	1(1)	15(1)	2(1)
C(3)	40(2)	46(2)	44(2)	-5(1)	8(1)	4(1)
C(4)	69(2)	57(2)	44(2)	-9(2)	6(2)	17(2)
C(5)	73(2)	72(2)	46(2)	2(2)	24(2)	23(2)
C(6)	52(2)	65(2)	51(2)	16(2)	26(2)	14(2)
C(7)	38(2)	46(2)	46(2)	13(1)	16(1)	5(1)
C(8)	42(2)	34(1)	54(2)	10(1)	13(1)	-1(1)
C(9)	48(2)	39(2)	68(2)	7(2)	-4(2)	-2(1)
C(10)	47(2)	46(2)	115(3)	19(2)	-8(2)	-7(2)
C(11)	61(2)	39(2)	60(2)	-9(2)	-10(2)	5(2)
C(12)	101(3)	65(3)	84(3)	-28(2)	-23(3)	8(2)
C(13)	65(2)	47(2)	46(2)	-2(1)	12(2)	1(2)
C(14)	39(2)	38(2)	40(2)	-4(1)	11(1)	-4(1)
C(15)	38(1)	34(1)	49(2)	0(1)	14(1)	1(1)
C(16)	40(2)	45(2)	62(2)	14(2)	19(1)	3(1)
C(17)	51(2)	47(2)	67(2)	18(2)	21(2)	5(1)
C(18)	51(2)	51(2)	61(2)	10(2)	14(2)	15(2)
C(19)	41(2)	53(2)	54(2)	-5(2)	14(1)	10(1)
C(20)	41(2)	39(2)	49(2)	-2(1)	20(1)	1(1)
N(1)	32(1)	41(1)	49(1)	12(1)	12(1)	-1(1)
O(1)	44(1)	44(1)	46(1)	14(1)	21(1)	3(1)

Table S5. Hydrogen coordinates (x 10^4) and isotropic displacement parameters (Å ^{2}x 10^3) for d19364.

	x	y	z	U(eq)
H(1)	819	5173	4455	47
H(3)	361	4448	2074	53
H(4)	817	4151	-358	71
H(5)	2916	4556	-889	75
H(6)	4535	5223	1013	64
H(10A)	7027	6288	6652	116
H(10B)	6565	6985	7143	116
H(10C)	6241	6799	5225	116
H(12A)	5522	7044	9026	143
H(12B)	4774	6522	9903	143
H(12C)	3854	7140	9000	143
H(13A)	1832	6397	6917	65
H(13B)	2674	5727	7587	65
H(16)	2728	6673	2575	58
H(17)	1097	7390	811	65
H(18)	-1305	7493	906	66
H(19)	-2068	6859	2746	59
H(20)	-436	6141	4531	50
H(1')	3476	4653	5817	51

(b) X-ray crystallographic structure and data for compound ($5a-O_3$)

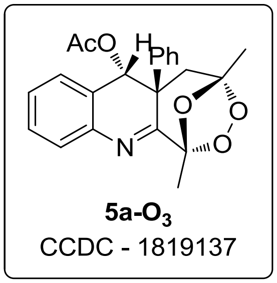

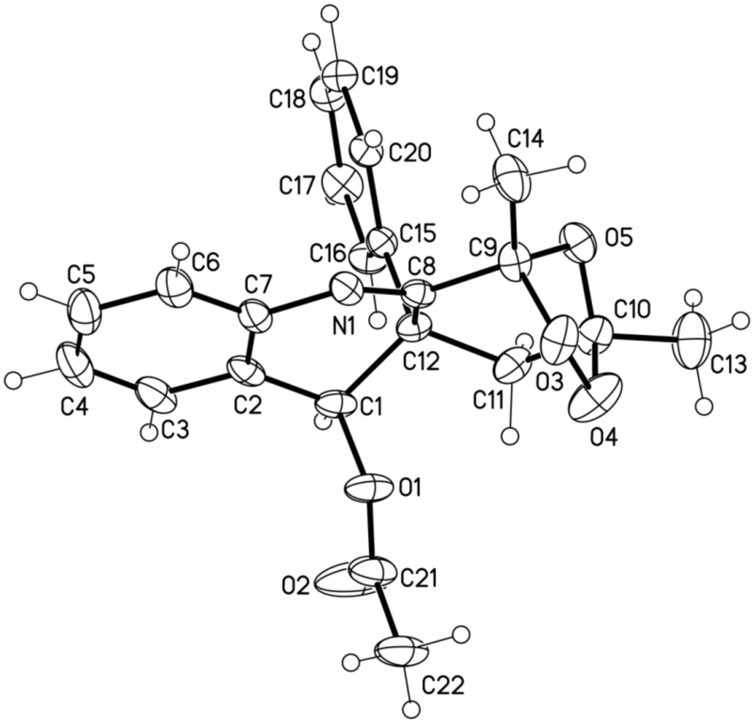

Table S6. Crystal data and structure refinement for 180108LT_0M_A_SQ.

Identification code	180108lt_0m_a_sq	
Empirical formula	C22 H21 N O5	
Formula weight	379.40	
Temperature	100(2) K	
Wavelength	0.71073 Å	
Crystal system	Monoclinic	
Space group	P 21/c	
Unit cell dimensions	a = 11.8837(10) Å	α= 90°.
	b = 10.0611(7) Å	β= 107.518(3)°.
	c = 19.6889(16) Å	γ = 90°.
Volume	2244.9(3) $Å^3$	
Z	4	
Density (calculated)	1.123 Mg/m^3	
Absorption coefficient	0.080 mm^{-1}	
F(000)	800	
Crystal size	0.20 x 0.15 x 0.15 mm^3	
Theta range for data collection	2.169 to 26.414°.	
Index ranges	-14<=h<=14, -12<=k<=12, -22<=l<=24	
Reflections collected	19526	
Independent reflections	4606 [R(int) = 0.0304]	
Completeness to theta = 25.242°	100.0 %	
Absorption correction	Semi-empirical from equivalents	
Max. and min. transmission	0.9485 and 0.8566	
Refinement method	Full-matrix least-squares on F^2	
Data / restraints / parameters	4606 / 0 / 256	
Goodness-of-fit on F^2	1.064	
Final R indices [I>2sigma(I)]	R1 = 0.0513, wR2 = 0.1177	
R indices (all data)	R1 = 0.0637, wR2 = 0.1254	
Extinction coefficient	n/a	
Largest diff. peak and hole	0.409 and -0.392 e.$Å^{-3}$	

Table S7. Atomic coordinates (x 10^4) and equivalent isotropic displacement parameters (Å ^{2}x 10^3) for 180108LT_0M_A_SQ. U(eq) is defined as one third of the trace of the orthogonalized U^{ij} tensor.

	x	y	z	U(eq)
C(1)	6145(2)	4106(2)	2494(1)	24(1)
C(2)	4877(2)	4300(2)	2084(1)	24(1)
C(3)	3971(2)	3633(2)	2247(1)	31(1)
C(4)	2811(2)	3881(2)	1862(1)	38(1)
C(5)	2547(2)	4810(2)	1322(1)	36(1)
C(6)	3445(2)	5496(2)	1155(1)	30(1)
C(7)	4608(2)	5230(2)	1530(1)	23(1)
C(8)	6554(2)	5466(2)	1552(1)	20(1)
C(9)	7501(2)	6209(2)	1324(1)	24(1)
C(10)	8951(2)	5245(2)	2152(1)	27(1)
C(11)	8258(2)	4230(2)	2428(1)	29(1)
C(12)	6924(2)	4207(2)	1998(1)	21(1)
C(13)	10256(2)	5001(3)	2368(2)	47(1)
C(14)	7102(2)	6802(2)	591(1)	35(1)
C(15)	6652(2)	2980(2)	1505(1)	19(1)
C(16)	6866(2)	1714(2)	1806(1)	24(1)
C(17)	6668(2)	586(2)	1385(1)	28(1)
C(18)	6247(2)	697(2)	654(1)	28(1)
C(19)	6008(2)	1944(2)	341(1)	25(1)
C(20)	6208(2)	3074(2)	768(1)	21(1)
C(21)	6796(2)	4882(2)	3697(1)	36(1)
C(22)	7020(3)	6092(2)	4157(1)	43(1)
N(1)	5509(1)	5927(2)	1337(1)	22(1)
O(1)	6468(2)	5192(1)	3009(1)	30(1)
O(2)	6915(3)	3760(2)	3909(1)	76(1)
O(3)	7963(1)	7259(1)	1818(1)	34(1)
O(4)	8747(2)	6551(2)	2419(1)	44(1)
O(5)	8482(1)	5372(1)	1404(1)	28(1)

Table S8. Bond lengths [Å] and angles [°] for 180108LT_0M_A_SQ.

C(1)-O(1)	1.462(2)
C(1)-C(2)	1.492(3)
C(1)-C(12)	1.538(3)
C(1)-H(1)	1.0000
C(2)-C(3)	1.385(3)
C(2)-C(7)	1.400(3)
C(3)-C(4)	1.381(3)
C(3)-H(3)	0.9500
C(4)-C(5)	1.380(4)
C(4)-H(4)	0.9500
C(5)-C(6)	1.391(3)
C(5)-H(5)	0.9500
C(6)-C(7)	1.382(3)
C(6)-H(6)	0.9500
C(7)-N(1)	1.424(2)
C(8)-N(1)	1.273(3)
C(8)-C(9)	1.527(3)
C(8)-C(12)	1.529(3)
C(9)-O(5)	1.408(2)
C(9)-O(3)	1.428(2)
C(9)-C(14)	1.500(3)
C(10)-O(5)	1.416(3)
C(10)-O(4)	1.462(2)
C(10)-C(13)	1.499(3)
C(10)-C(11)	1.513(3)
C(11)-C(12)	1.556(3)
C(11)-H(11A)	0.9900
C(11)-H(11B)	0.9900
C(12)-C(15)	1.542(3)
C(13)-H(13A)	0.9800
C(13)-H(13B)	0.9800
C(13)-H(13C)	0.9800
C(14)-H(14A)	0.9800
C(14)-H(14B)	0.9800

C(14)-H(14C)	0.9800
C(15)-C(20)	1.390(3)
C(15)-C(16)	1.396(3)
C(16)-C(17)	1.382(3)
C(16)-H(16)	0.9500
C(17)-C(18)	1.379(3)
C(17)-H(17)	0.9500
C(18)-C(19)	1.389(3)
C(18)-H(18)	0.9500
C(19)-C(20)	1.391(3)
C(19)-H(19)	0.9500
C(20)-H(20)	0.9500
C(21)-O(2)	1.196(3)
C(21)-O(1)	1.329(3)
C(21)-C(22)	1.492(3)
C(22)-H(22A)	0.9800
C(22)-H(22B)	0.9800
C(22)-H(22C)	0.9800
O(3)-O(4)	1.453(2)
O(1)-C(1)-C(2)	106.80(16)
O(1)-C(1)-C(12)	107.99(15)
C(2)-C(1)-C(12)	110.51(16)
O(1)-C(1)-H(1)	110.5
C(2)-C(1)-H(1)	110.5
C(12)-C(1)-H(1)	110.5
C(3)-C(2)-C(7)	119.5(2)
C(3)-C(2)-C(1)	122.72(19)
C(7)-C(2)-C(1)	117.78(17)
C(4)-C(3)-C(2)	120.2(2)
C(4)-C(3)-H(3)	119.9
C(2)-C(3)-H(3)	119.9
C(5)-C(4)-C(3)	120.2(2)
C(5)-C(4)-H(4)	119.9
C(3)-C(4)-H(4)	119.9
C(4)-C(5)-C(6)	120.4(2)

C(4)-C(5)-H(5)	119.8
C(6)-C(5)-H(5)	119.8
C(7)-C(6)-C(5)	119.4(2)
C(7)-C(6)-H(6)	120.3
C(5)-C(6)-H(6)	120.3
C(6)-C(7)-C(2)	120.26(19)
C(6)-C(7)-N(1)	118.22(18)
C(2)-C(7)-N(1)	121.52(18)
N(1)-C(8)-C(9)	117.16(17)
N(1)-C(8)-C(12)	125.10(17)
C(9)-C(8)-C(12)	117.71(16)
O(5)-C(9)-O(3)	103.35(15)
O(5)-C(9)-C(14)	110.88(17)
O(3)-C(9)-C(14)	107.83(17)
O(5)-C(9)-C(8)	109.47(15)
O(3)-C(9)-C(8)	109.17(16)
C(14)-C(9)-C(8)	115.42(17)
O(5)-C(10)-O(4)	103.71(16)
O(5)-C(10)-C(13)	111.06(19)
O(4)-C(10)-C(13)	108.32(19)
O(5)-C(10)-C(11)	110.41(17)
O(4)-C(10)-C(11)	108.07(18)
C(13)-C(10)-C(11)	114.62(19)
C(10)-C(11)-C(12)	112.98(17)
C(10)-C(11)-H(11A)	109.0
C(12)-C(11)-H(11A)	109.0
C(10)-C(11)-H(11B)	109.0
C(12)-C(11)-H(11B)	109.0
H(11A)-C(11)-H(11B)	107.8
C(8)-C(12)-C(1)	107.23(16)
C(8)-C(12)-C(15)	109.37(15)
C(1)-C(12)-C(15)	107.14(15)
C(8)-C(12)-C(11)	111.69(16)
C(1)-C(12)-C(11)	111.35(16)
C(15)-C(12)-C(11)	109.92(16)
C(10)-C(13)-H(13A)	109.5

C(10)-C(13)-H(13B)	109.5
H(13A)-C(13)-H(13B)	109.5
C(10)-C(13)-H(13C)	109.5
H(13A)-C(13)-H(13C)	109.5
H(13B)-C(13)-H(13C)	109.5
C(9)-C(14)-H(14A)	109.5
C(9)-C(14)-H(14B)	109.5
H(14A)-C(14)-H(14B)	109.5
C(9)-C(14)-H(14C)	109.5
H(14A)-C(14)-H(14C)	109.5
H(14B)-C(14)-H(14C)	109.5
C(20)-C(15)-C(16)	117.92(17)
C(20)-C(15)-C(12)	122.96(17)
C(16)-C(15)-C(12)	119.11(17)
C(17)-C(16)-C(15)	121.25(18)
C(17)-C(16)-H(16)	119.4
C(15)-C(16)-H(16)	119.4
C(18)-C(17)-C(16)	120.15(19)
C(18)-C(17)-H(17)	119.9
C(16)-C(17)-H(17)	119.9
C(17)-C(18)-C(19)	119.80(19)
C(17)-C(18)-H(18)	120.1
C(19)-C(18)-H(18)	120.1
C(18)-C(19)-C(20)	119.72(18)
C(18)-C(19)-H(19)	120.1
C(20)-C(19)-H(19)	120.1
C(15)-C(20)-C(19)	121.14(18)
C(15)-C(20)-H(20)	119.4
C(19)-C(20)-H(20)	119.4
O(2)-C(21)-O(1)	123.0(2)
O(2)-C(21)-C(22)	125.3(2)
O(1)-C(21)-C(22)	111.72(19)
C(21)-C(22)-H(22A)	109.5
C(21)-C(22)-H(22B)	109.5
H(22A)-C(22)-H(22B)	109.5
C(21)-C(22)-H(22C)	109.5

H(22A)-C(22)-H(22C)	109.5
H(22B)-C(22)-H(22C)	109.5
C(8)-N(1)-C(7)	118.01(16)
C(21)-O(1)-C(1)	117.81(16)
C(9)-O(3)-O(4)	102.28(13)
O(3)-O(4)-C(10)	106.44(15)
C(9)-O(5)-C(10)	102.88(15)

Symmetry transformations used to generate equivalent atoms:

Table S9. Anisotropic displacement parameters (Å ^{2}x 10^3) for 180108LT_0M_A_SQ. The anisotropic displacement factor exponent takes the form: $-2\pi^2[h^2 a^{*2}U^{11} + ... + 2 h k a^* b^* U^{12}]$

	U^{11}	U^{22}	U^{33}	U^{23}	U^{13}	U^{12}
C(1)	40(1)	15(1)	18(1)	-3(1)	12(1)	-5(1)
C(2)	36(1)	17(1)	26(1)	-6(1)	19(1)	-3(1)
C(3)	45(1)	22(1)	36(1)	-6(1)	27(1)	-6(1)
C(4)	42(1)	28(1)	56(2)	-13(1)	35(1)	-12(1)
C(5)	26(1)	33(1)	52(2)	-11(1)	18(1)	-3(1)
C(6)	30(1)	24(1)	39(1)	-5(1)	16(1)	1(1)
C(7)	27(1)	18(1)	28(1)	-6(1)	14(1)	-3(1)
C(8)	26(1)	16(1)	18(1)	-2(1)	9(1)	-1(1)
C(9)	24(1)	17(1)	33(1)	2(1)	10(1)	1(1)
C(10)	25(1)	22(1)	33(1)	-4(1)	6(1)	1(1)
C(11)	28(1)	30(1)	24(1)	0(1)	0(1)	-2(1)
C(12)	26(1)	17(1)	18(1)	0(1)	5(1)	-1(1)
C(13)	25(1)	50(2)	62(2)	7(1)	8(1)	8(1)
C(14)	32(1)	37(1)	41(1)	17(1)	17(1)	1(1)
C(15)	19(1)	19(1)	20(1)	0(1)	7(1)	1(1)
C(16)	30(1)	22(1)	21(1)	2(1)	7(1)	4(1)
C(17)	35(1)	18(1)	34(1)	3(1)	14(1)	4(1)
C(18)	30(1)	21(1)	34(1)	-7(1)	13(1)	-1(1)
C(19)	26(1)	28(1)	21(1)	-3(1)	7(1)	-2(1)
C(20)	24(1)	20(1)	21(1)	3(1)	8(1)	1(1)
C(21)	60(2)	31(1)	22(1)	-4(1)	20(1)	-17(1)
C(22)	72(2)	36(1)	26(1)	-12(1)	22(1)	-19(1)
N(1)	26(1)	17(1)	25(1)	-1(1)	12(1)	-2(1)
O(1)	53(1)	20(1)	19(1)	-4(1)	12(1)	-5(1)
O(2)	166(3)	35(1)	24(1)	-1(1)	22(1)	-33(1)
O(3)	27(1)	18(1)	50(1)	-2(1)	5(1)	0(1)
O(4)	48(1)	24(1)	46(1)	-7(1)	-9(1)	5(1)
O(5)	28(1)	24(1)	35(1)	3(1)	15(1)	4(1)

Table S10. Hydrogen coordinates (x 10^4) and isotropic displacement parameters (Å ^{2}x 10 ^{3}) for 180108LT_0M_A_SQ.

	x	y	z	U(eq)
H(1)	6262	3228	2744	29
H(3)	4148	3003	2624	37
H(4)	2193	3411	1971	45
H(5)	1747	4982	1062	43
H(6)	3261	6142	786	35
H(11A)	8339	4429	2933	35
H(11B)	8597	3337	2408	35
H(13A)	10411	4139	2183	70
H(13B)	10571	5000	2889	70
H(13C)	10639	5706	2174	70
H(14A)	7760	7279	499	53
H(14B)	6451	7422	557	53
H(14C)	6834	6093	239	53
H(16)	7152	1625	2309	29
H(17)	6822	-266	1601	34
H(18)	6120	-78	365	33
H(19)	5710	2025	-162	30
H(20)	6039	3925	552	26
H(22A)	7355	5829	4657	65
H(22B)	6277	6567	4095	65
H(22C)	7577	6674	4020	65

(c) X-ray crystallographic structure and data for compound (7a)

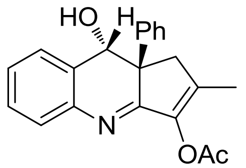

7a
CCDC - 1819138

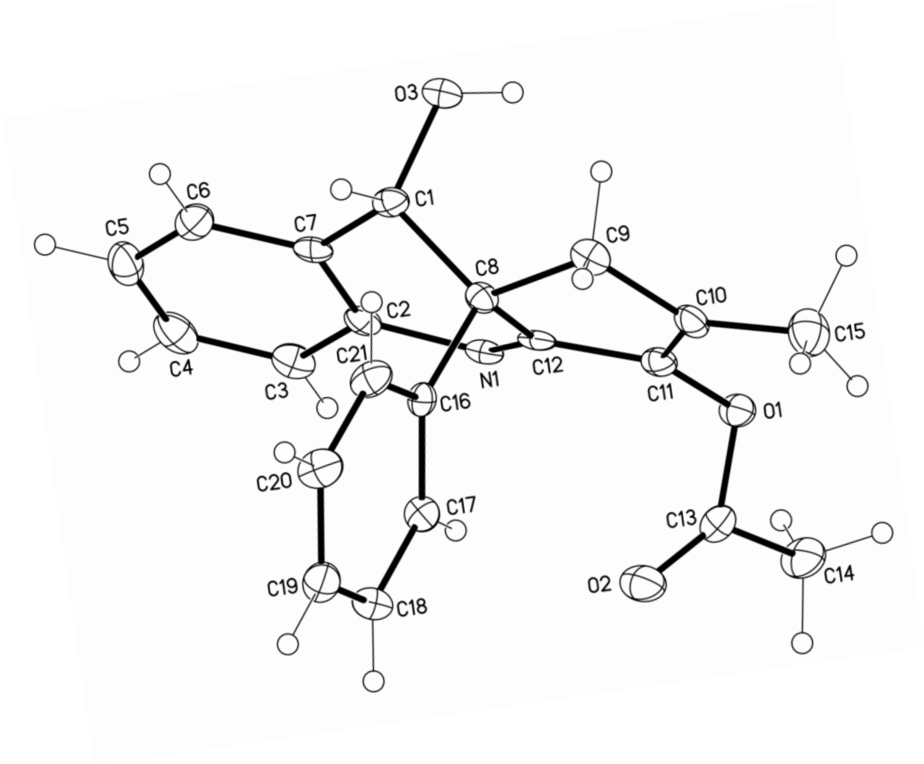

Table S11. Crystal data and structure refinement for TWIN5.

Identification code	TWIN5	
Empirical formula	C43 H39 Cl3 N2 O6	
Formula weight	786.11	
Temperature	100(2) K	
Wavelength	0.71073 Å	
Crystal system	Triclinic	
Space group	P -1	
Unit cell dimensions	a = 11.282(7) Å	α= 84.071(19)°.
	b = 11.457(7) Å	β= 88.539(15)°.
	c = 14.934(9) Å	γ = 84.074(13)°.
Volume	1909(2) Å ^{3}	
Z	2	
Density (calculated)	1.367 Mg/m^3	
Absorption coefficient	0.292 mm^{-1}	
F(000)	820	
Crystal size	0.18 x 0.15 x 0.04 mm^3	
Theta range for data collection	1.371 to 24.997°.	
Index ranges	-13<=h<=13, -13<=k<=13, 0<=l<=17	
Reflections collected	6443	
Independent reflections	6443 [R(int) = 0.0495]	
Completeness to theta = 25.000°	95.9 %	
Absorption correction	Semi-empirical from equivalents	
Max. and min. transmission	0.9484 and 0.8556	
Refinement method	Full-matrix least-squares on F^2	
Data / restraints / parameters	6443 / 0 / 494	
Goodness-of-fit on F^2	1.028	
Final R indices [I>2sigma(I)]	R1 = 0.0451, wR2 = 0.0929	
R indices (all data)	R1 = 0.0704, wR2 = 0.1031	
Extinction coefficient	n/a	
Largest diff. peak and hole	0.302 and -0.328 e.Å $^{-3}$	

Table S12. Atomic coordinates (x 10^4) and equivalent isotropic displacement parameters (Å ^{2}x 10^3) for TWIN5. U(eq) is defined as one third of the trace of the orthogonalized U^{ij} tensor.

	x	y	z	U(eq)
C(1)	8091(3)	4161(2)	6166(2)	15(1)
C(2)	10340(3)	3893(2)	6227(2)	15(1)
C(3)	11420(3)	3181(3)	6334(2)	18(1)
C(4)	11426(3)	1976(3)	6554(2)	24(1)
C(5)	10366(3)	1463(3)	6660(2)	24(1)
C(6)	9286(3)	2164(3)	6543(2)	20(1)
C(7)	9253(3)	3383(2)	6326(2)	15(1)
C(8)	8239(2)	5360(2)	6517(2)	14(1)
C(9)	7305(3)	6404(2)	6192(2)	18(1)
C(10)	8032(3)	7440(2)	6000(2)	16(1)
C(11)	9185(3)	7053(2)	5938(2)	16(1)
C(12)	9402(2)	5786(2)	6133(2)	14(1)
C(13)	10952(3)	7868(2)	6226(2)	19(1)
C(14)	11874(3)	8595(3)	5768(2)	26(1)
C(15)	7494(3)	8689(3)	5881(2)	27(1)
C(16)	8248(2)	5219(2)	7555(2)	13(1)
C(17)	9217(3)	5472(2)	8043(2)	16(1)
C(18)	9153(3)	5394(3)	8977(2)	20(1)
C(19)	8147(3)	5057(2)	9444(2)	21(1)
C(20)	7188(3)	4793(3)	8965(2)	22(1)
C(21)	7236(3)	4888(3)	8034(2)	20(1)
C(22)	5704(3)	2013(2)	8814(2)	16(1)
C(23)	6533(3)	905(3)	8721(2)	18(1)
C(24)	7742(3)	932(3)	8528(2)	23(1)
C(25)	8475(3)	-103(3)	8453(2)	28(1)
C(26)	8010(3)	-1188(3)	8576(2)	26(1)
C(27)	6802(3)	-1232(3)	8779(2)	20(1)
C(28)	4120(2)	670(2)	8816(2)	13(1)
C(29)	2844(3)	843(2)	8970(2)	14(1)
C(30)	2410(3)	1975(3)	8842(2)	17(1)
C(31)	3415(3)	2731(2)	8605(2)	17(1)

C(32)	4503(3)	1851(2)	8389(2)	15(1)
C(33)	1145(3)	2474(3)	8955(2)	25(1)
O(6)	2183(2)	-78(2)	9304(1)	17(1)
C(35)	2042(2)	-905(2)	8727(2)	15(1)
C(36)	4696(2)	1847(2)	7369(2)	13(1)
C(37)	4974(3)	2884(3)	6861(2)	18(1)
C(38)	5183(3)	2915(3)	5938(2)	23(1)
C(39)	5102(3)	1921(3)	5504(2)	22(1)
C(40)	4804(3)	896(3)	6001(2)	20(1)
C(41)	4604(3)	859(2)	6923(2)	16(1)
C(42)	6058(2)	-191(2)	8836(2)	15(1)
C(43)	3645(3)	6718(2)	7504(2)	21(1)
Cl(1)	3697(1)	5908(1)	6562(1)	38(1)
Cl(2)	3862(1)	5771(1)	8510(1)	39(1)
Cl(3)	4755(1)	7703(1)	7392(1)	37(1)
N(1)	10375(2)	5136(2)	5996(1)	14(1)
N(2)	4816(2)	-276(2)	9026(1)	14(1)
O(1)	10070(2)	7779(2)	5635(1)	17(1)
O(2)	10959(2)	7417(2)	6987(1)	26(1)
O(3)	7745(2)	4276(2)	5248(1)	18(1)
O(4)	5578(2)	2292(2)	9727(1)	21(1)
O(5)	2349(2)	-790(2)	7955(1)	21(1)
C(34)	1463(3)	-1920(3)	9203(2)	23(1)

Table S13. Bond lengths [Å] and angles [°] for TWIN5.

C(1)-O(3)	1.423(3)
C(1)-C(7)	1.517(4)
C(1)-C(8)	1.546(4)
C(1)-H(1)	1.0000
C(2)-C(3)	1.398(4)
C(2)-C(7)	1.411(4)
C(2)-N(1)	1.434(4)
C(3)-C(4)	1.384(4)
C(3)-H(3)	0.9500
C(4)-C(5)	1.384(4)
C(4)-H(4)	0.9500
C(5)-C(6)	1.392(4)
C(5)-H(5)	0.9500
C(6)-C(7)	1.397(4)
C(6)-H(6)	0.9500
C(8)-C(12)	1.524(4)
C(8)-C(16)	1.542(4)
C(8)-C(9)	1.556(4)
C(9)-C(10)	1.511(4)
C(9)-H(9A)	0.9900
C(9)-H(9B)	0.9900
C(10)-C(11)	1.335(4)
C(10)-C(15)	1.490(4)
C(11)-O(1)	1.402(3)
C(11)-C(12)	1.447(4)
C(12)-N(1)	1.286(4)
C(13)-O(2)	1.199(3)
C(13)-O(1)	1.365(3)
C(13)-C(14)	1.506(4)
C(14)-H(14A)	0.9800
C(14)-H(14B)	0.9800
C(14)-H(14C)	0.9800
C(15)-H(15A)	0.9800
C(15)-H(15B)	0.9800

C(15)-H(15C)	0.9800
C(16)-C(21)	1.397(4)
C(16)-C(17)	1.400(4)
C(17)-C(18)	1.389(4)
C(17)-H(17)	0.9500
C(18)-C(19)	1.384(4)
C(18)-H(18)	0.9500
C(19)-C(20)	1.388(4)
C(19)-H(19)	0.9500
C(20)-C(21)	1.383(4)
C(20)-H(20)	0.9500
C(21)-H(21)	0.9500
C(22)-O(4)	1.432(3)
C(22)-C(23)	1.513(4)
C(22)-C(32)	1.546(4)
C(22)-H(22)	1.0000
C(23)-C(24)	1.390(4)
C(23)-C(42)	1.408(4)
C(24)-C(25)	1.387(5)
C(24)-H(24)	0.9500
C(25)-C(26)	1.392(5)
C(25)-H(25)	0.9500
C(26)-C(27)	1.394(4)
C(26)-H(26)	0.9500
C(27)-C(42)	1.395(4)
C(27)-H(27)	0.9500
C(28)-N(2)	1.287(4)
C(28)-C(29)	1.449(4)
C(28)-C(32)	1.533(4)
C(29)-C(30)	1.335(4)
C(29)-O(6)	1.398(3)
C(30)-C(33)	1.494(4)
C(30)-C(31)	1.510(4)
C(31)-C(32)	1.556(4)
C(31)-H(31A)	0.9900
C(31)-H(31B)	0.9900

C(32)-C(36)	1.534(4)
C(33)-H(33A)	0.9800
C(33)-H(33B)	0.9800
C(33)-H(33C)	0.9800
O(6)-C(35)	1.368(3)
C(35)-O(5)	1.194(3)
C(35)-C(34)	1.498(4)
C(36)-C(41)	1.386(4)
C(36)-C(37)	1.401(4)
C(37)-C(38)	1.391(4)
C(37)-H(37)	0.9500
C(38)-C(39)	1.379(4)
C(38)-H(38)	0.9500
C(39)-C(40)	1.389(4)
C(39)-H(39)	0.9500
C(40)-C(41)	1.386(4)
C(40)-H(40)	0.9500
C(41)-H(41)	0.9500
C(42)-N(2)	1.433(4)
C(43)-Cl(1)	1.760(3)
C(43)-Cl(3)	1.764(3)
C(43)-Cl(2)	1.766(3)
C(43)-H(43)	1.0000
O(3)-H(3A)	0.8400
O(4)-H(4A)	0.8400
C(34)-H(34A)	0.9800
C(34)-H(34B)	0.9800
C(34)-H(34C)	0.9800

O(3)-C(1)-C(7)	112.4(2)
O(3)-C(1)-C(8)	112.9(2)
C(7)-C(1)-C(8)	107.5(2)
O(3)-C(1)-H(1)	108.0
C(7)-C(1)-H(1)	108.0
C(8)-C(1)-H(1)	108.0
C(3)-C(2)-C(7)	120.0(3)

C(3)-C(2)-N(1)	118.4(3)
C(7)-C(2)-N(1)	121.6(2)
C(4)-C(3)-C(2)	120.2(3)
C(4)-C(3)-H(3)	119.9
C(2)-C(3)-H(3)	119.9
C(5)-C(4)-C(3)	120.5(3)
C(5)-C(4)-H(4)	119.7
C(3)-C(4)-H(4)	119.7
C(4)-C(5)-C(6)	119.7(3)
C(4)-C(5)-H(5)	120.1
C(6)-C(5)-H(5)	120.1
C(5)-C(6)-C(7)	121.0(3)
C(5)-C(6)-H(6)	119.5
C(7)-C(6)-H(6)	119.5
C(6)-C(7)-C(2)	118.5(3)
C(6)-C(7)-C(1)	122.1(3)
C(2)-C(7)-C(1)	119.3(2)
C(12)-C(8)-C(16)	110.8(2)
C(12)-C(8)-C(1)	108.4(2)
C(16)-C(8)-C(1)	109.9(2)
C(12)-C(8)-C(9)	102.4(2)
C(16)-C(8)-C(9)	108.9(2)
C(1)-C(8)-C(9)	116.2(2)
C(10)-C(9)-C(8)	104.1(2)
C(10)-C(9)-H(9A)	110.9
C(8)-C(9)-H(9A)	110.9
C(10)-C(9)-H(9B)	110.9
C(8)-C(9)-H(9B)	110.9
H(9A)-C(9)-H(9B)	109.0
C(11)-C(10)-C(15)	127.0(3)
C(11)-C(10)-C(9)	109.6(2)
C(15)-C(10)-C(9)	123.3(3)
C(10)-C(11)-O(1)	123.5(2)
C(10)-C(11)-C(12)	112.5(2)
O(1)-C(11)-C(12)	123.8(3)
N(1)-C(12)-C(11)	127.1(2)

N(1)-C(12)-C(8)	126.3(2)
C(11)-C(12)-C(8)	106.7(2)
O(2)-C(13)-O(1)	123.3(3)
O(2)-C(13)-C(14)	127.2(3)
O(1)-C(13)-C(14)	109.5(2)
C(13)-C(14)-H(14A)	109.5
C(13)-C(14)-H(14B)	109.5
H(14A)-C(14)-H(14B)	109.5
C(13)-C(14)-H(14C)	109.5
H(14A)-C(14)-H(14C)	109.5
H(14B)-C(14)-H(14C)	109.5
C(10)-C(15)-H(15A)	109.5
C(10)-C(15)-H(15B)	109.5
H(15A)-C(15)-H(15B)	109.5
C(10)-C(15)-H(15C)	109.5
H(15A)-C(15)-H(15C)	109.5
H(15B)-C(15)-H(15C)	109.5
C(21)-C(16)-C(17)	118.1(2)
C(21)-C(16)-C(8)	119.5(2)
C(17)-C(16)-C(8)	122.3(2)
C(18)-C(17)-C(16)	120.0(3)
C(18)-C(17)-H(17)	120.0
C(16)-C(17)-H(17)	120.0
C(19)-C(18)-C(17)	121.3(3)
C(19)-C(18)-H(18)	119.3
C(17)-C(18)-H(18)	119.3
C(18)-C(19)-C(20)	119.0(3)
C(18)-C(19)-H(19)	120.5
C(20)-C(19)-H(19)	120.5
C(21)-C(20)-C(19)	120.1(3)
C(21)-C(20)-H(20)	119.9
C(19)-C(20)-H(20)	119.9
C(20)-C(21)-C(16)	121.4(3)
C(20)-C(21)-H(21)	119.3
C(16)-C(21)-H(21)	119.3
O(4)-C(22)-C(23)	112.5(2)

O(4)-C(22)-C(32)	113.0(2)
C(23)-C(22)-C(32)	107.6(2)
O(4)-C(22)-H(22)	107.8
C(23)-C(22)-H(22)	107.8
C(32)-C(22)-H(22)	107.8
C(24)-C(23)-C(42)	119.1(3)
C(24)-C(23)-C(22)	122.4(3)
C(42)-C(23)-C(22)	118.5(3)
C(25)-C(24)-C(23)	120.7(3)
C(25)-C(24)-H(24)	119.6
C(23)-C(24)-H(24)	119.6
C(24)-C(25)-C(26)	120.3(3)
C(24)-C(25)-H(25)	119.8
C(26)-C(25)-H(25)	119.8
C(25)-C(26)-C(27)	119.7(3)
C(25)-C(26)-H(26)	120.1
C(27)-C(26)-H(26)	120.1
C(26)-C(27)-C(42)	120.1(3)
C(26)-C(27)-H(27)	120.0
C(42)-C(27)-H(27)	120.0
N(2)-C(28)-C(29)	127.0(2)
N(2)-C(28)-C(32)	126.0(3)
C(29)-C(28)-C(32)	107.0(2)
C(30)-C(29)-O(6)	124.6(3)
C(30)-C(29)-C(28)	112.7(2)
O(6)-C(29)-C(28)	122.3(2)
C(29)-C(30)-C(33)	127.2(3)
C(29)-C(30)-C(31)	109.6(3)
C(33)-C(30)-C(31)	123.1(2)
C(30)-C(31)-C(32)	105.1(2)
C(30)-C(31)-H(31A)	110.7
C(32)-C(31)-H(31A)	110.7
C(30)-C(31)-H(31B)	110.7
C(32)-C(31)-H(31B)	110.7
H(31A)-C(31)-H(31B)	108.8
C(28)-C(32)-C(36)	111.4(2)

C(28)-C(32)-C(22)	107.0(2)
C(36)-C(32)-C(22)	108.4(2)
C(28)-C(32)-C(31)	102.2(2)
C(36)-C(32)-C(31)	110.8(2)
C(22)-C(32)-C(31)	116.9(2)
C(30)-C(33)-H(33A)	109.5
C(30)-C(33)-H(33B)	109.5
H(33A)-C(33)-H(33B)	109.5
C(30)-C(33)-H(33C)	109.5
H(33A)-C(33)-H(33C)	109.5
H(33B)-C(33)-H(33C)	109.5
C(35)-O(6)-C(29)	116.0(2)
O(5)-C(35)-O(6)	122.4(2)
O(5)-C(35)-C(34)	127.0(3)
O(6)-C(35)-C(34)	110.6(2)
C(41)-C(36)-C(37)	118.2(3)
C(41)-C(36)-C(32)	122.8(2)
C(37)-C(36)-C(32)	119.0(2)
C(38)-C(37)-C(36)	121.0(3)
C(38)-C(37)-H(37)	119.5
C(36)-C(37)-H(37)	119.5
C(39)-C(38)-C(37)	120.1(3)
C(39)-C(38)-H(38)	120.0
C(37)-C(38)-H(38)	120.0
C(38)-C(39)-C(40)	119.2(3)
C(38)-C(39)-H(39)	120.4
C(40)-C(39)-H(39)	120.4
C(41)-C(40)-C(39)	120.9(3)
C(41)-C(40)-H(40)	119.5
C(39)-C(40)-H(40)	119.5
C(36)-C(41)-C(40)	120.6(3)
C(36)-C(41)-H(41)	119.7
C(40)-C(41)-H(41)	119.7
C(27)-C(42)-C(23)	120.1(3)
C(27)-C(42)-N(2)	118.2(2)
C(23)-C(42)-N(2)	121.7(3)

Cl(1)-C(43)-Cl(3)	109.66(16)
Cl(1)-C(43)-Cl(2)	110.99(16)
Cl(3)-C(43)-Cl(2)	109.49(16)
Cl(1)-C(43)-H(43)	108.9
Cl(3)-C(43)-H(43)	108.9
Cl(2)-C(43)-H(43)	108.9
C(12)-N(1)-C(2)	115.8(2)
C(28)-N(2)-C(42)	115.6(2)
C(13)-O(1)-C(11)	116.6(2)
C(1)-O(3)-H(3A)	109.5
C(22)-O(4)-H(4A)	109.5
C(35)-C(34)-H(34A)	109.5
C(35)-C(34)-H(34B)	109.5
H(34A)-C(34)-H(34B)	109.5
C(35)-C(34)-H(34C)	109.5
H(34A)-C(34)-H(34C)	109.5
H(34B)-C(34)-H(34C)	109.5

Symmetry transformations used to generate equivalent atoms:

Table S14. Anisotropic displacement parameters ($Å^2 x 10^3$) for TWIN5. The anisotropic displacement factor exponent takes the form: $-2\pi^2[h^2 a^{*2}U^{11} + ... + 2 h k a^* b^* U^{12}]$

	U^{11}	U^{22}	U^{33}	U^{23}	U^{13}	U^{12}
C(1)	17(2)	19(2)	9(1)	-3(1)	2(1)	-6(1)
C(2)	18(2)	20(2)	7(1)	-3(1)	0(1)	0(1)
C(3)	15(2)	25(2)	15(2)	-4(1)	1(1)	0(1)
C(4)	26(2)	29(2)	15(2)	-4(1)	0(1)	7(1)
C(5)	35(2)	18(2)	16(2)	0(1)	0(1)	3(1)
C(6)	26(2)	21(2)	13(2)	-4(1)	4(1)	-7(1)
C(7)	17(2)	21(2)	7(1)	-5(1)	0(1)	-2(1)
C(8)	10(2)	16(2)	14(2)	0(1)	-2(1)	-1(1)
C(9)	15(2)	23(2)	16(2)	-1(1)	0(1)	-1(1)
C(10)	20(2)	18(2)	10(1)	0(1)	-1(1)	0(1)
C(11)	19(2)	21(2)	9(1)	0(1)	-1(1)	-8(1)
C(12)	13(2)	22(2)	7(1)	-3(1)	-3(1)	-6(1)
C(13)	19(2)	16(2)	22(2)	-3(1)	0(1)	-4(1)
C(14)	26(2)	28(2)	25(2)	-1(1)	2(1)	-14(2)
C(15)	22(2)	25(2)	33(2)	1(1)	-2(1)	1(1)
C(16)	14(2)	10(1)	15(2)	-2(1)	1(1)	1(1)
C(17)	14(2)	17(2)	17(2)	-1(1)	1(1)	-3(1)
C(18)	21(2)	23(2)	16(2)	-3(1)	-4(1)	-3(1)
C(19)	29(2)	19(2)	15(2)	-4(1)	5(1)	-4(1)
C(20)	21(2)	28(2)	18(2)	-5(1)	7(1)	-9(1)
C(21)	18(2)	22(2)	21(2)	-6(1)	4(1)	-7(1)
C(22)	24(2)	16(2)	10(1)	0(1)	-1(1)	-10(1)
C(23)	21(2)	25(2)	9(1)	-1(1)	-5(1)	-4(1)
C(24)	21(2)	32(2)	17(2)	6(1)	-3(1)	-11(2)
C(25)	16(2)	44(2)	22(2)	4(2)	0(1)	-6(2)
C(26)	20(2)	34(2)	21(2)	-1(1)	-2(1)	7(1)
C(27)	23(2)	21(2)	15(2)	0(1)	-3(1)	-2(1)
C(28)	20(2)	14(2)	6(1)	-4(1)	-1(1)	-5(1)
C(29)	18(2)	16(2)	10(1)	-2(1)	1(1)	-6(1)
C(30)	20(2)	22(2)	10(1)	-5(1)	-1(1)	-1(1)
C(31)	24(2)	12(1)	16(2)	-4(1)	1(1)	-1(1)

C(32)	20(2)	12(1)	13(2)	-1(1)	-1(1)	-4(1)
C(33)	24(2)	25(2)	23(2)	-5(1)	3(1)	4(1)
O(6)	19(1)	18(1)	15(1)	-3(1)	3(1)	-7(1)
C(35)	14(2)	14(2)	17(2)	-4(1)	-3(1)	0(1)
C(36)	11(2)	13(1)	14(1)	-1(1)	-1(1)	0(1)
C(37)	21(2)	19(2)	16(2)	-1(1)	-5(1)	-7(1)
C(38)	22(2)	27(2)	19(2)	5(1)	-6(1)	-10(1)
C(39)	18(2)	34(2)	14(2)	-1(1)	1(1)	-1(1)
C(40)	23(2)	23(2)	15(2)	-6(1)	0(1)	2(1)
C(41)	17(2)	14(2)	17(2)	-1(1)	-1(1)	0(1)
C(42)	16(2)	20(2)	8(1)	2(1)	-2(1)	-4(1)
C(43)	20(2)	18(2)	24(2)	-4(1)	-1(1)	-4(1)
Cl(1)	53(1)	31(1)	31(1)	-17(1)	-7(1)	0(1)
Cl(2)	49(1)	38(1)	27(1)	6(1)	2(1)	2(1)
Cl(3)	26(1)	21(1)	68(1)	-16(1)	14(1)	-9(1)
N(1)	12(1)	21(1)	10(1)	-3(1)	-3(1)	-3(1)
N(2)	15(1)	15(1)	12(1)	-3(1)	-2(1)	-2(1)
O(1)	18(1)	20(1)	15(1)	1(1)	0(1)	-9(1)
O(2)	28(1)	35(1)	19(1)	3(1)	-3(1)	-17(1)
O(3)	14(1)	30(1)	11(1)	-4(1)	2(1)	-6(1)
O(4)	35(1)	16(1)	12(1)	0(1)	-3(1)	-11(1)
O(5)	25(1)	22(1)	17(1)	-6(1)	2(1)	-8(1)
C(34)	23(2)	21(2)	27(2)	-2(1)	2(1)	-9(1)

Table S15. Hydrogen coordinates (x 10^4) and isotropic displacement parameters (Å ^{2}x 10^3) for TWIN5.

	x	y	z	U(eq)
H(1)	7454	3789	6536	18
H(3)	12152	3523	6256	22
H(4)	12162	1498	6634	29
H(5)	10375	637	6811	28
H(6)	8561	1808	6612	23
H(9A)	6905	6241	5643	22
H(9B)	6695	6552	6667	22
H(14A)	12401	8816	6220	39
H(14B)	11476	9310	5443	39
H(14C)	12345	8133	5342	39
H(15A)	8128	9216	5846	40
H(15B)	6964	8847	6394	40
H(15C)	7036	8824	5324	40
H(17)	9919	5696	7736	19
H(18)	9812	5575	9302	24
H(19)	8114	5007	10083	25
H(20)	6498	4547	9277	26
H(21)	6565	4724	7714	24
H(22)	6051	2683	8453	19
H(24)	8070	1668	8446	28
H(25)	9299	-71	8316	33
H(26)	8514	-1895	8523	31
H(27)	6485	-1971	8878	24
H(31A)	3587	3168	9117	20
H(31B)	3211	3304	8076	20
H(33A)	639	1829	9081	37
H(33B)	883	2956	8401	37
H(33C)	1082	2965	9458	37
H(37)	5021	3576	7153	22
H(38)	5382	3623	5605	27

H(39)	5248	1936	4874	26
H(40)	4737	211	5705	24
H(41)	4402	150	7251	19
H(43)	2846	7182	7535	25
H(3A)	8332	4427	4915	27
H(4A)	5566	1666	10073	31
H(34A)	1192	-2402	8758	35
H(34B)	778	-1618	9559	35
H(34C)	2039	-2402	9602	35

(d) X-ray crystallographic structure and data for compound (9b)

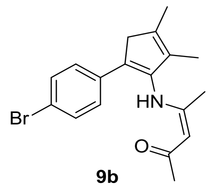

9b

CCDC - 1819136

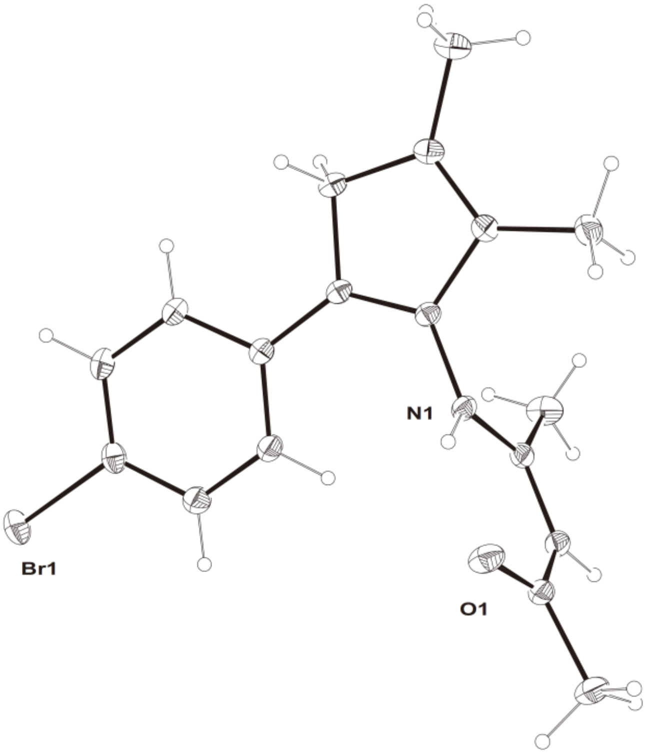

Table S16. Crystal data and structure refinement for d19332.

Identification code	d19332	
Empirical formula	C18 H20 Br N O	
Formula weight	346.26	
Temperature	200(2) K	
Wavelength	0.71073 Å	
Crystal system	Triclinic	
Space group	P -1	
Unit cell dimensions	a = 7.6388(10) Å	α= 116.079(3)°.
	b = 11.2206(13) Å	β= 109.218(4)°.
	c = 11.3511(13) Å	γ = 91.505(4)°.
Volume	808.23(17) Å ^{3}	
Z	2	
Density (calculated)	1.423 Mg/m^{3}	
Absorption coefficient	2.542 mm^{-1}	
F(000)	356	
Crystal size	0.43 x 0.31 x 0.15 mm^{3}	
Theta range for data collection	2.87 to 25.07°.	
Index ranges	-9<=h<=9, -13<=k<=13, -13<=l<=13	
Reflections collected	26621	
Independent reflections	2830 [R(int) = 0.0345]	
Completeness to theta = 25.07°	98.7 %	
Absorption correction	multi-scan	
Max. and min. transmission	0.7017 and 0.4078	
Refinement method	Full-matrix least-squares on F^{2}	
Data / restraints / parameters	2830 / 0 / 192	
Goodness-of-fit on F^{2}	1.060	
Final R indices [I>2sigma(I)]	R1 = 0.0237, wR2 = 0.0630	
R indices (all data)	R1 = 0.0258, wR2 = 0.0643	
Largest diff. peak and hole	0.228 and -0.318 e.Å $^{-3}$	

Table S17. Atomic coordinates (x 10^4) and equivalent isotropic displacement parameters (Å ^{2}x 10^3) for d19332. U(eq) is defined as one third of the trace of the orthogonalized U^{ij} tensor.

	x	y	z	U(eq)
C(1)	4367(3)	8155(2)	7066(2)	39(1)
C(2)	3160(3)	7474(2)	5659(2)	41(1)
C(3)	2584(3)	6087(2)	5005(2)	37(1)
C(4)	3178(2)	5336(2)	5724(2)	31(1)
C(5)	4390(3)	6070(2)	7156(2)	40(1)
C(6)	4982(3)	7458(2)	7822(2)	44(1)
C(7)	2509(2)	3872(2)	4993(2)	30(1)
C(8)	1050(2)	3113(2)	3503(2)	34(1)
C(9)	797(2)	1663(2)	3184(2)	36(1)
C(10)	-481(3)	543(2)	1771(2)	50(1)
C(11)	1944(2)	1566(2)	4312(2)	34(1)
C(12)	2222(3)	320(2)	4467(2)	45(1)
C(13)	3002(2)	2930(2)	5414(2)	30(1)
C(14)	4258(2)	3204(2)	7831(2)	31(1)
C(15)	2314(3)	3133(3)	7840(2)	52(1)
C(16)	5801(3)	3330(2)	8962(2)	35(1)
C(17)	7660(3)	3336(2)	8970(2)	37(1)
C(18)	9204(3)	3379(2)	10236(2)	54(1)
Br(1)	5171(1)	10062(1)	7971(1)	51(1)
N(1)	4473(2)	3152(2)	6689(2)	31(1)
O(1)	8081(2)	3301(2)	7987(1)	47(1)

Table S18. Bond lengths [Å] and angles [°] for d19332.

C(1)-C(2)	1.381(3)
C(1)-C(6)	1.382(3)
C(1)-Br(1)	1.8961(19)
C(2)-C(3)	1.379(3)
C(2)-H(2)	0.9500
C(3)-C(4)	1.402(2)
C(3)-H(3)	0.9500
C(4)-C(5)	1.403(3)
C(4)-C(7)	1.458(3)
C(5)-C(6)	1.380(3)
C(5)-H(5)	0.9500
C(6)-H(6)	0.9500
C(7)-C(13)	1.354(2)
C(7)-C(8)	1.507(2)
C(8)-C(9)	1.495(3)
C(8)-H(8A)	0.9900
C(8)-H(8B)	0.9900
C(9)-C(11)	1.345(3)
C(9)-C(10)	1.496(3)
C(10)-H(10A)	0.9800
C(10)-H(10B)	0.9800
C(10)-H(10C)	0.9800
C(11)-C(13)	1.465(2)
C(11)-C(12)	1.496(3)
C(12)-H(12A)	0.9800
C(12)-H(12B)	0.9800
C(12)-H(12C)	0.9800
C(13)-N(1)	1.421(2)
C(14)-N(1)	1.336(2)
C(14)-C(16)	1.376(2)
C(14)-C(15)	1.489(3)
C(15)-H(15A)	0.9549
C(15)-H(15B)	0.8910
C(15)-H(15C)	0.9907

C(16)-C(17)	1.417(3)
C(16)-H(16)	0.9500
C(17)-O(1)	1.244(2)
C(17)-C(18)	1.509(3)
C(18)-H(18A)	0.9645
C(18)-H(18B)	1.0108
C(18)-H(18C)	0.9528
N(1)-H(1')	0.9247
C(2)-C(1)-C(6)	120.53(18)
C(2)-C(1)-Br(1)	119.63(14)
C(6)-C(1)-Br(1)	119.83(15)
C(3)-C(2)-C(1)	119.40(17)
C(3)-C(2)-H(2)	120.3
C(1)-C(2)-H(2)	120.3
C(2)-C(3)-C(4)	122.14(17)
C(2)-C(3)-H(3)	118.9
C(4)-C(3)-H(3)	118.9
C(3)-C(4)-C(5)	116.50(17)
C(3)-C(4)-C(7)	120.12(16)
C(5)-C(4)-C(7)	123.37(15)
C(6)-C(5)-C(4)	121.94(17)
C(6)-C(5)-H(5)	119.0
C(4)-C(5)-H(5)	119.0
C(5)-C(6)-C(1)	119.49(18)
C(5)-C(6)-H(6)	120.3
C(1)-C(6)-H(6)	120.3
C(13)-C(7)-C(4)	130.85(16)
C(13)-C(7)-C(8)	106.27(15)
C(4)-C(7)-C(8)	122.87(15)
C(9)-C(8)-C(7)	104.79(14)
C(9)-C(8)-H(8A)	110.8
C(7)-C(8)-H(8A)	110.8
C(9)-C(8)-H(8B)	110.8
C(7)-C(8)-H(8B)	110.8
H(8A)-C(8)-H(8B)	108.9

C(11)-C(9)-C(10)	128.15(18)
C(11)-C(9)-C(8)	109.32(15)
C(10)-C(9)-C(8)	122.44(17)
C(9)-C(10)-H(10A)	109.5
C(9)-C(10)-H(10B)	109.5
H(10A)-C(10)-H(10B)	109.5
C(9)-C(10)-H(10C)	109.5
H(10A)-C(10)-H(10C)	109.5
H(10B)-C(10)-H(10C)	109.5
C(9)-C(11)-C(13)	107.95(16)
C(9)-C(11)-C(12)	128.44(17)
C(13)-C(11)-C(12)	123.56(16)
C(11)-C(12)-H(12A)	109.5
C(11)-C(12)-H(12B)	109.5
H(12A)-C(12)-H(12B)	109.5
C(11)-C(12)-H(12C)	109.5
H(12A)-C(12)-H(12C)	109.5
H(12B)-C(12)-H(12C)	109.5
C(7)-C(13)-N(1)	126.50(16)
C(7)-C(13)-C(11)	111.65(15)
N(1)-C(13)-C(11)	121.59(15)
N(1)-C(14)-C(16)	120.42(16)
N(1)-C(14)-C(15)	118.03(16)
C(16)-C(14)-C(15)	121.55(16)
C(14)-C(15)-H(15A)	107.3
C(14)-C(15)-H(15B)	112.4
H(15A)-C(15)-H(15B)	108.9
C(14)-C(15)-H(15C)	112.9
H(15A)-C(15)-H(15C)	104.3
H(15B)-C(15)-H(15C)	110.7
C(14)-C(16)-C(17)	123.39(16)
C(14)-C(16)-H(16)	118.3
C(17)-C(16)-H(16)	118.3
O(1)-C(17)-C(16)	123.46(16)
O(1)-C(17)-C(18)	118.45(18)
C(16)-C(17)-C(18)	118.09(18)

C(17)-C(18)-H(18A)	107.9
C(17)-C(18)-H(18B)	108.3
H(18A)-C(18)-H(18B)	117.6
C(17)-C(18)-H(18C)	110.7
H(18A)-C(18)-H(18C)	104.5
H(18B)-C(18)-H(18C)	107.8
C(14)-N(1)-C(13)	126.37(14)
C(14)-N(1)-H(1')	114.2
C(13)-N(1)-H(1')	119.4

Symmetry transformations used to generate equivalent atoms:

Table S19. Anisotropic displacement parameters (Å ^{2}x 10^3)for d19332. The anisotropic displacement factor exponent takes the form: $-2\pi^2$[$h^2a^{*2}U^{11}$ + ... + 2 h k a* b* U^{12}]

	U^{11}	U^{22}	U^{33}	U^{23}	U^{13}	U^{12}
C(1)	45(1)	32(1)	47(1)	20(1)	25(1)	11(1)
C(2)	50(1)	41(1)	47(1)	29(1)	23(1)	16(1)
C(3)	40(1)	41(1)	34(1)	22(1)	14(1)	12(1)
C(4)	31(1)	36(1)	32(1)	19(1)	15(1)	11(1)
C(5)	50(1)	36(1)	35(1)	20(1)	10(1)	9(1)
C(6)	51(1)	37(1)	37(1)	16(1)	12(1)	7(1)
C(7)	28(1)	36(1)	28(1)	16(1)	11(1)	9(1)
C(8)	30(1)	43(1)	28(1)	18(1)	10(1)	10(1)
C(9)	30(1)	39(1)	32(1)	12(1)	12(1)	6(1)
C(10)	46(1)	50(1)	36(1)	9(1)	9(1)	1(1)
C(11)	31(1)	33(1)	36(1)	15(1)	14(1)	7(1)
C(12)	48(1)	35(1)	49(1)	20(1)	16(1)	7(1)
C(13)	27(1)	35(1)	29(1)	16(1)	10(1)	7(1)
C(14)	37(1)	26(1)	30(1)	12(1)	12(1)	6(1)
C(15)	41(1)	68(2)	42(1)	19(1)	19(1)	6(1)
C(16)	45(1)	32(1)	27(1)	14(1)	12(1)	5(1)
C(17)	42(1)	29(1)	32(1)	14(1)	5(1)	6(1)
C(18)	52(1)	57(1)	41(1)	26(1)	0(1)	10(1)
Br(1)	62(1)	33(1)	62(1)	21(1)	30(1)	7(1)
N(1)	28(1)	36(1)	33(1)	20(1)	9(1)	7(1)
O(1)	34(1)	68(1)	40(1)	28(1)	10(1)	10(1)

Table S20. Hydrogen coordinates (x 10^4) and isotropic displacement parameters (Å ^{2}x 10^3) for d19332.

	x	y	z	U(eq)
H(2)	2729	7958	5146	50
H(3)	1758	5625	4035	44
H(5)	4816	5598	7682	49
H(6)	5805	7931	8792	53
H(8A)	-156	3434	3443	40
H(8B)	1501	3232	2830	40
H(10A)	132	344	1089	76
H(10B)	-1674	820	1440	76
H(10C)	-738	-269	1857	76
H(12A)	1798	353	5206	67
H(12B)	3566	275	4732	67
H(12C)	1482	-483	3566	67
H(16)	5610	3418	9780	42
H(1')	5715	3242	6760	57
H(15A)	1793	3808	7619	57
H(15B)	1564	2327	7203	57
H(15C)	2305	3391	8792	57
H(18A)	10006	4269	10773	57
H(18B)	9818	2575	9887	57
H(18C)	8691	3308	10867	57

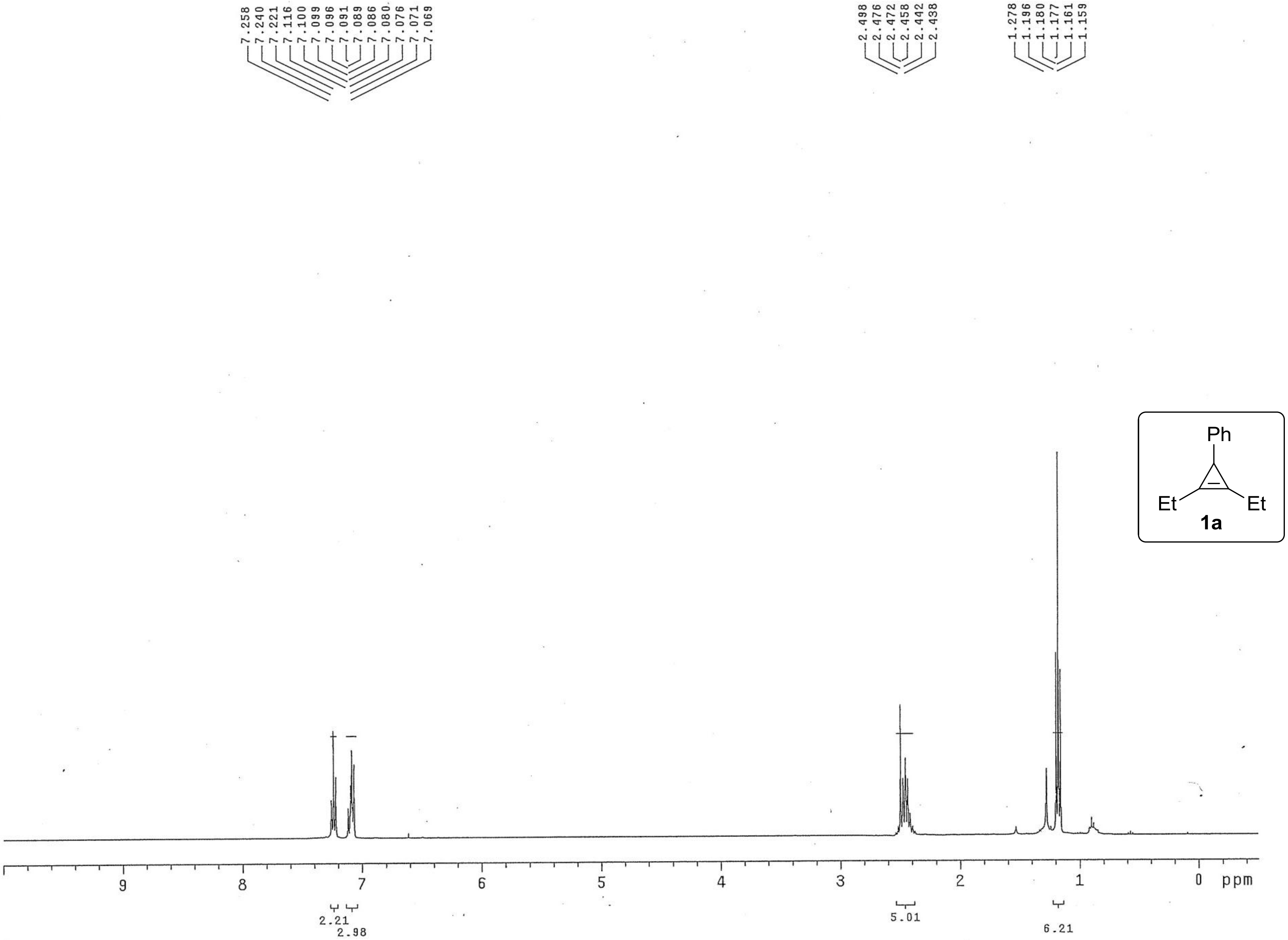
7.258
7.240
7.221
7.116
7.100
7.099
7.096
7.091
7.089
7.086
7.080
7.076
7.071
7.069
2.498
2.476
2.472
2.458
2.442
2.438
1.278
1.196
1.180
1.177
1.161
1.159
Ph
Et
Et
1a
9
8
7
6
5
4
3
2
1
0 ppm
2.21
2.98
5.01
6.21

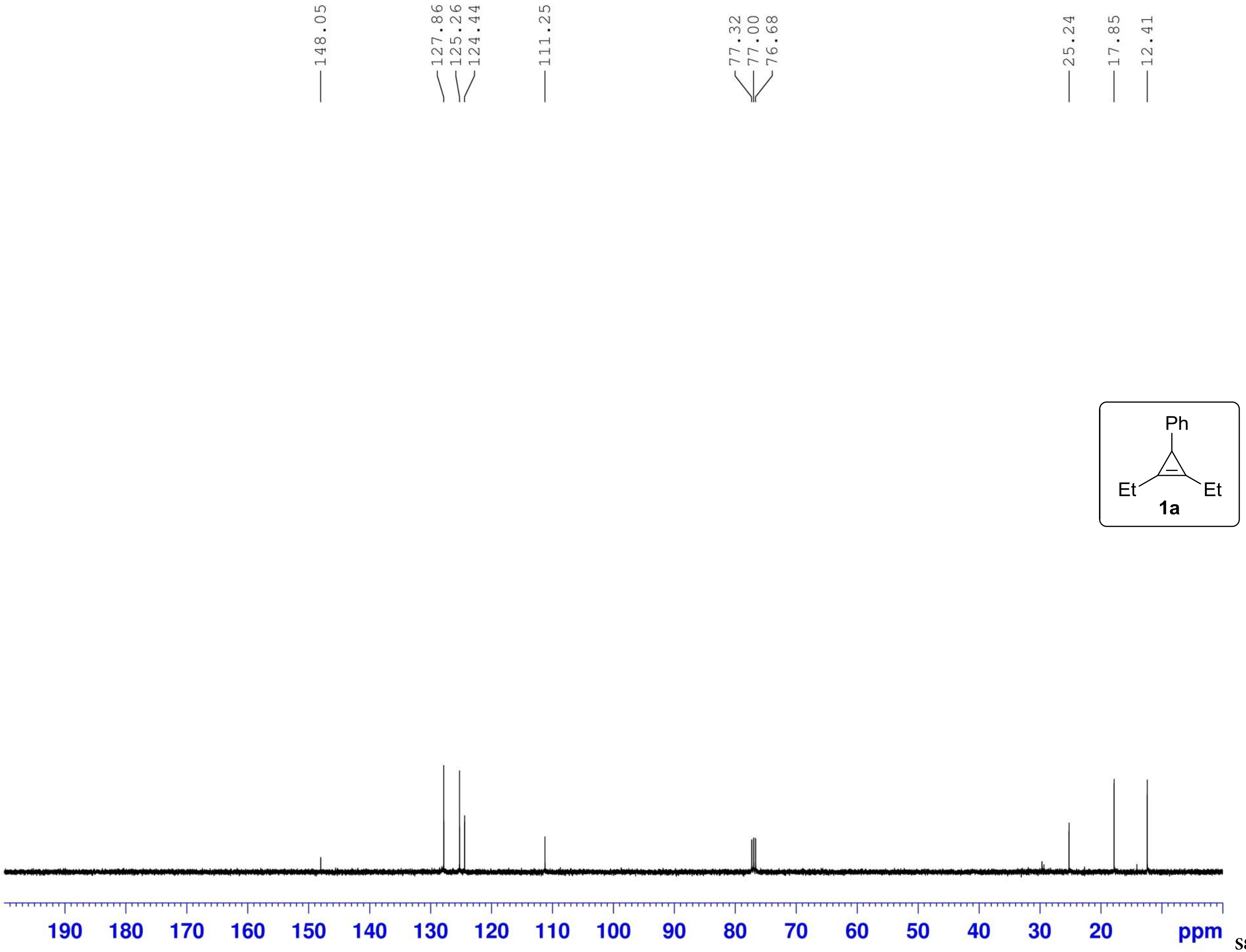
148.05
127.86
125.26
124.44
111.25
77.32
77.00
76.68
25.24
17.85
12.41
Ph
Et
Et
1a
190 180 170 160 150 140 130 120 110 100 90 80 70 60 50 40 30 20 ppm

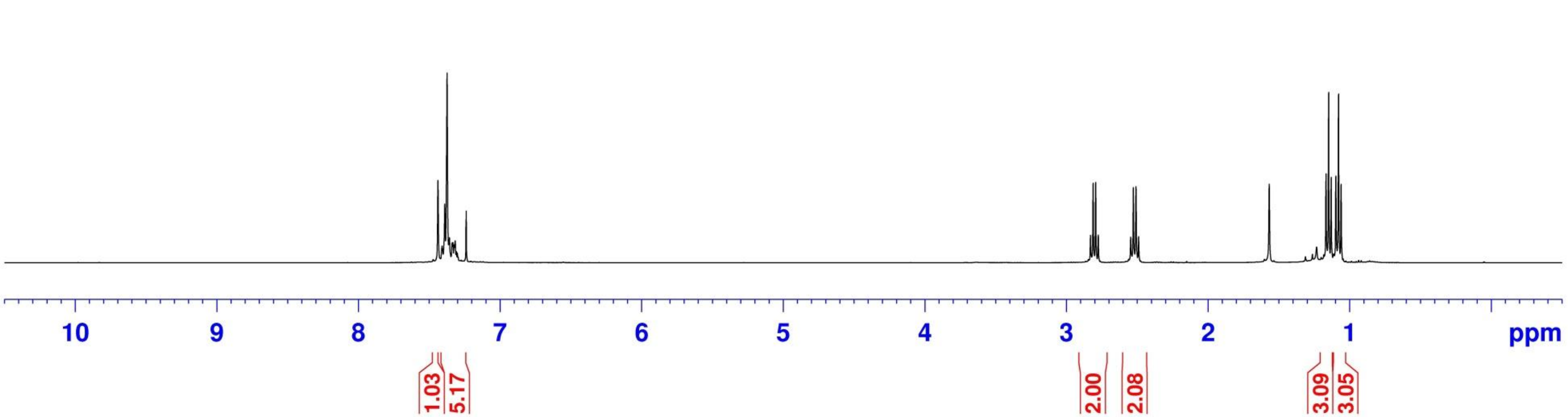
7.440
7.410
7.392
7.375
7.359
7.340
7.334
7.326
7.318
7.310
7.240
2.831
2.812
2.794
2.776
2.547
2.528
2.510
2.491
1.167
1.149
1.131
1.098
1.079
1.060
10
9
8
7
6
5
4
3
2
1
ppm
1.03
5.17
2.00
2.08
3.09
3.05

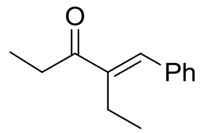
O
Ph

1a-O

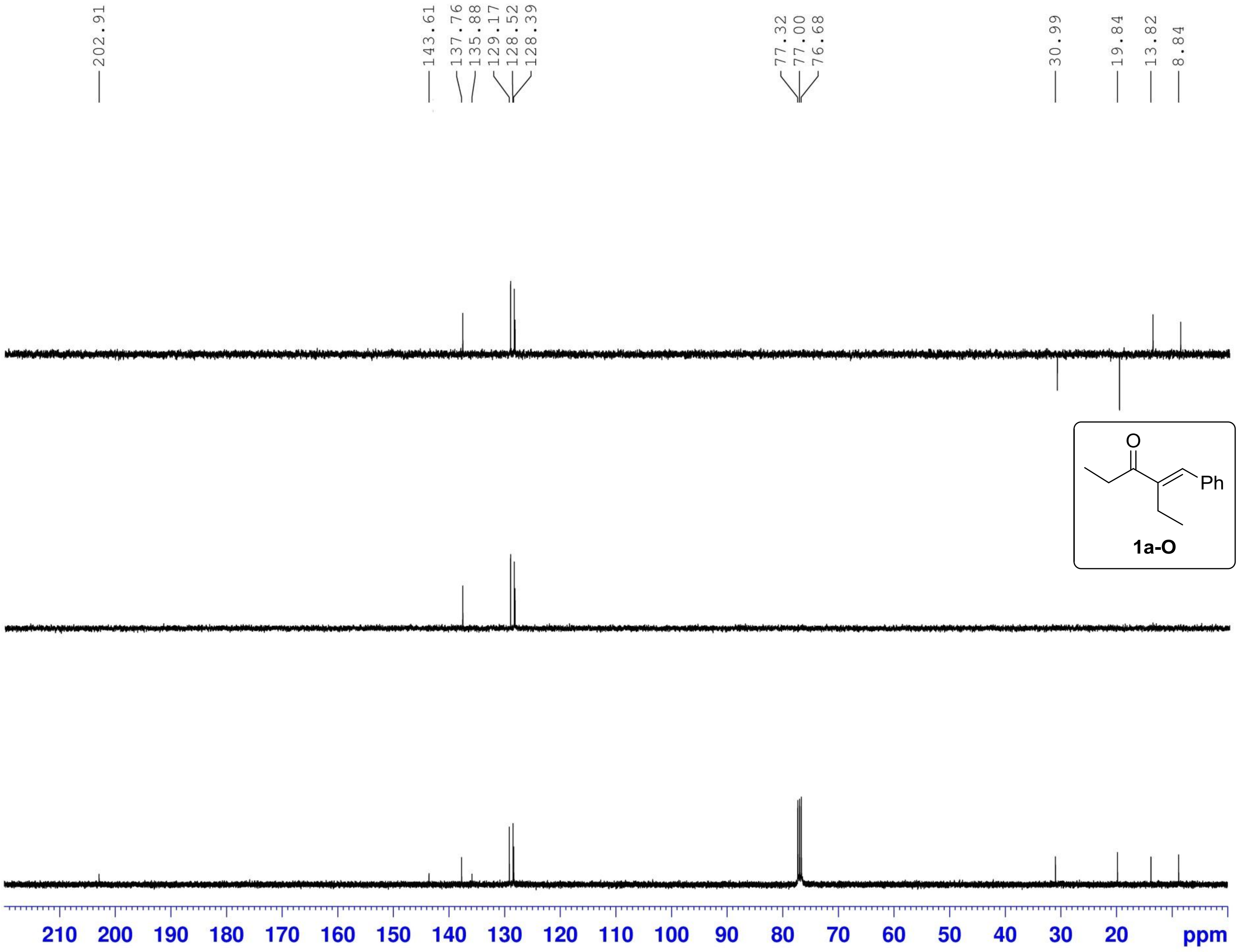
202.91
143.61
137.76
135.88
129.17
128.52
128.39
77.32
77.00
76.68
30.99
19.84
13.82
8.84
O
Ph
1a-O
210 200 190 180 170 160 150 140 130 120 110 100 90 80 70 60 50 40 30 20 ppm

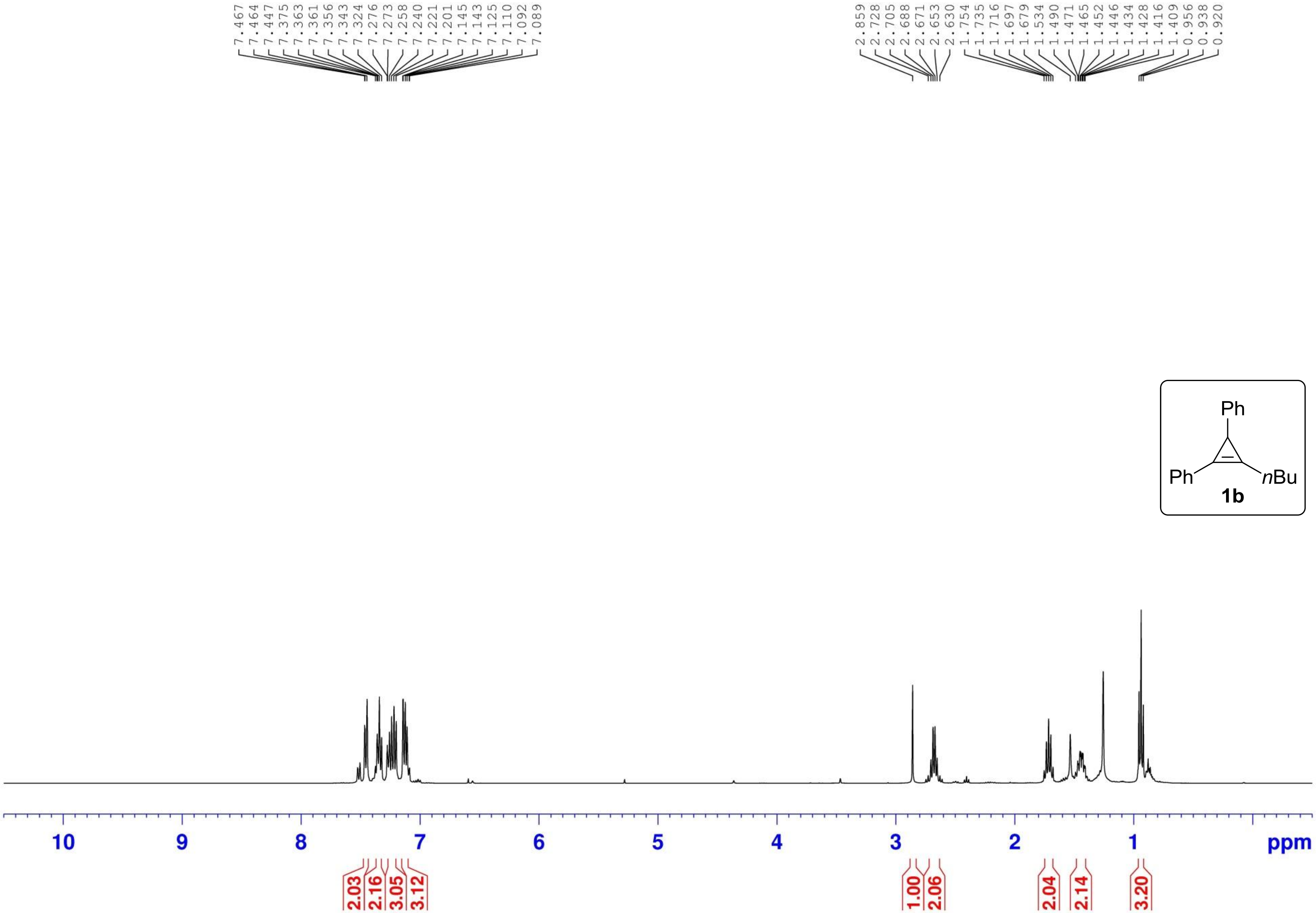
7.467
7.464
7.447
7.375
7.363
7.361
7.356
7.343
7.324
7.276
7.273
7.258
7.240
7.221
7.201
7.145
7.143
7.125
7.110
7.092
7.089
2.859
2.728
2.705
2.688
2.671
2.653
2.630
1.754
1.735
1.716
1.697
1.679
1.534
1.490
1.471
1.465
1.452
1.446
1.434
1.428
1.416
1.409
0.956
0.938
0.920
Ph
Ph
nBu
1b
10
9
8
7
6
5
4
3
2
1
ppm
2.03
2.16
3.05
3.12
1.00
2.06
2.04
2.14
3.20

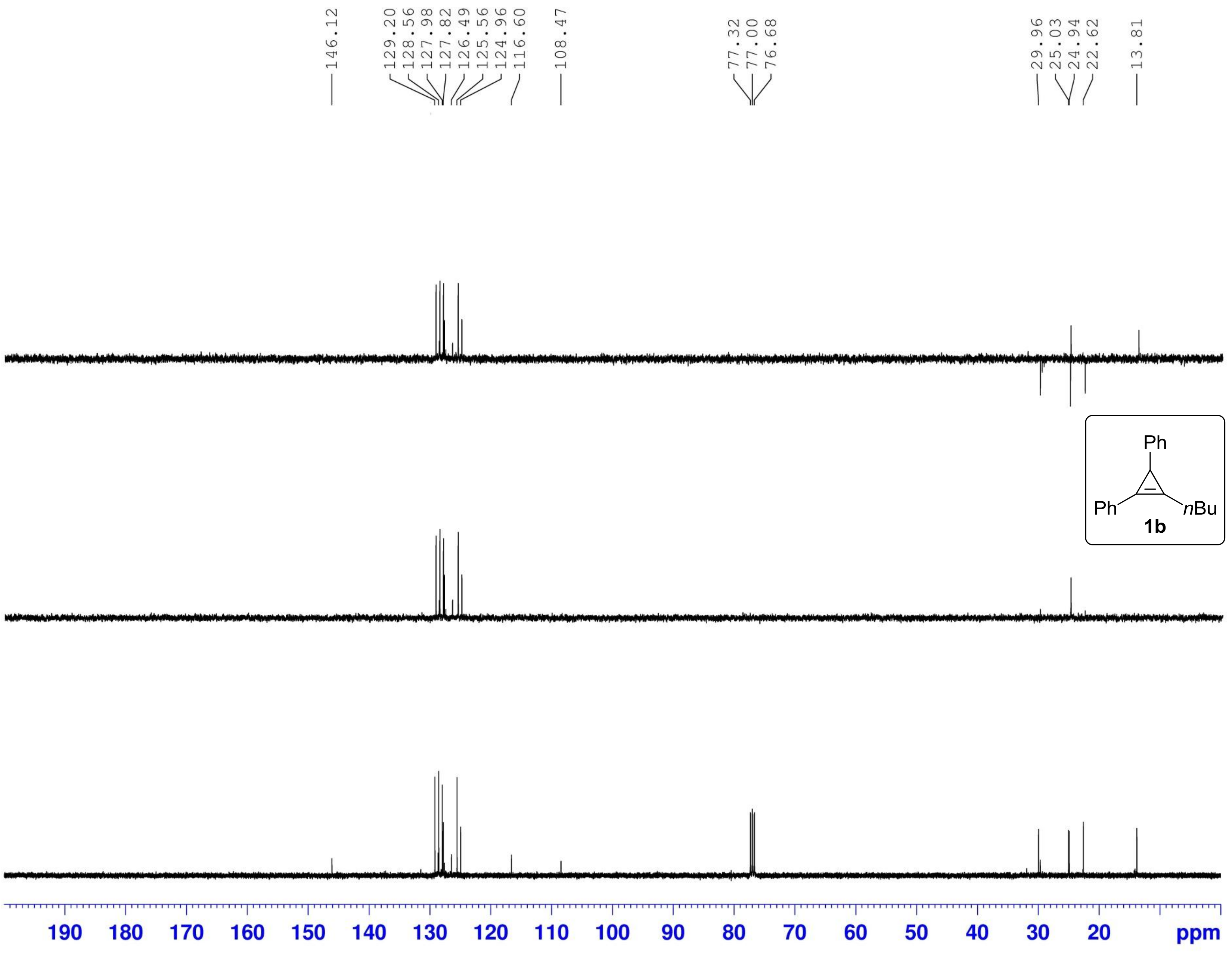
146.12
129.20
128.56
127.98
127.82
126.49
125.56
124.96
116.60
108.47
77.32
77.00
76.68
29.96
25.03
24.94
22.62
13.81
Ph
Ph
nBu
1b
190 180 170 160 150 140 130 120 110 100 90 80 70 60 50 40 30 20 ppm

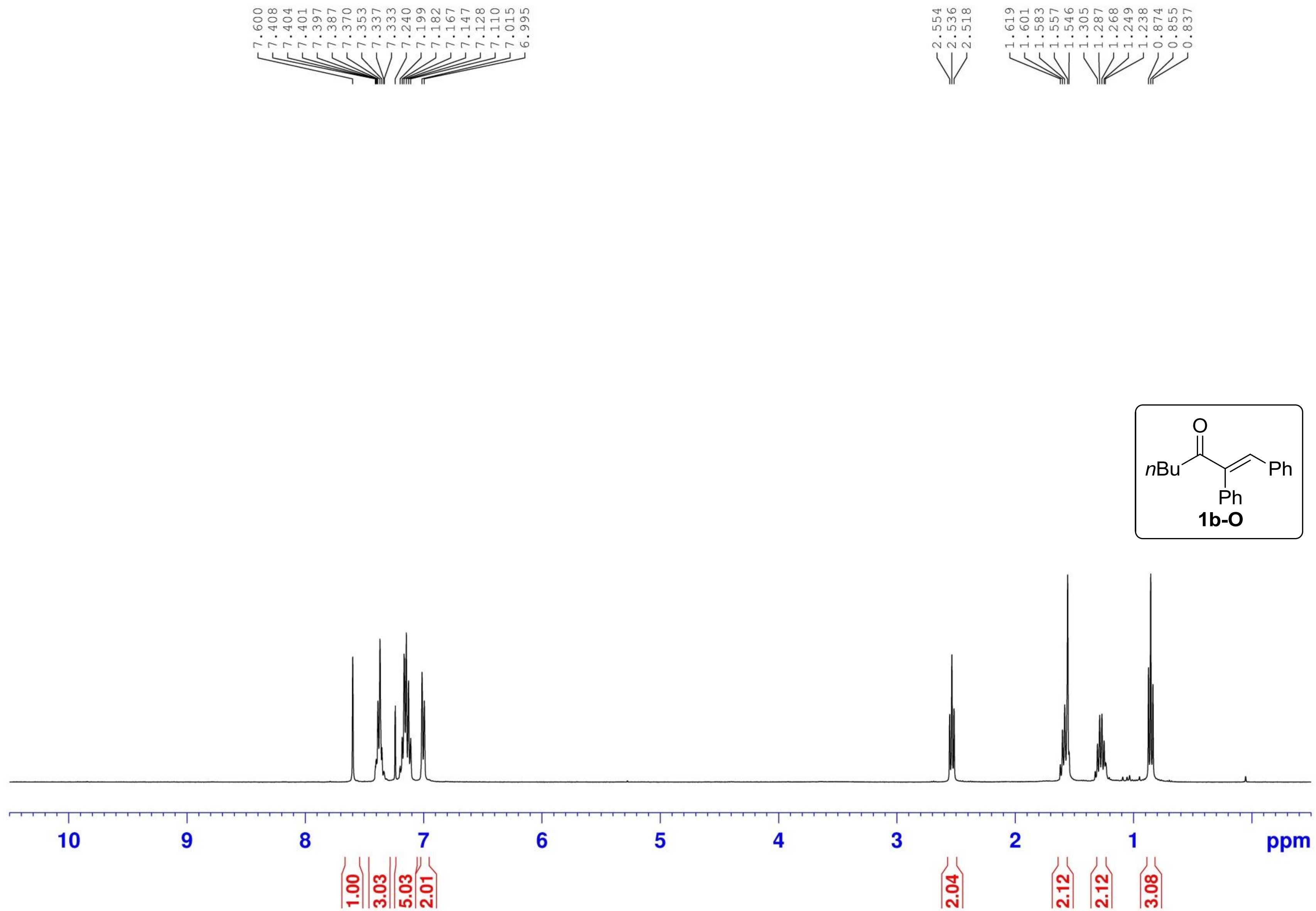

7.600
7.408
7.404
7.401
7.397
7.387
7.370
7.353
7.337
7.333
7.240
7.199
7.182
7.167
7.147
7.128
7.110
7.015
6.995
2.554
2.536
2.518
1.619
1.601
1.583
1.557
1.546
1.305
1.287
1.268
1.249
1.238
0.874
0.855
0.837
O
nBu
Ph
Ph
1b-O
10
9
8
7
6
5
4
3
2
1
ppm
1.00
3.03
5.03
2.01
2.04
2.12
2.12
3.08

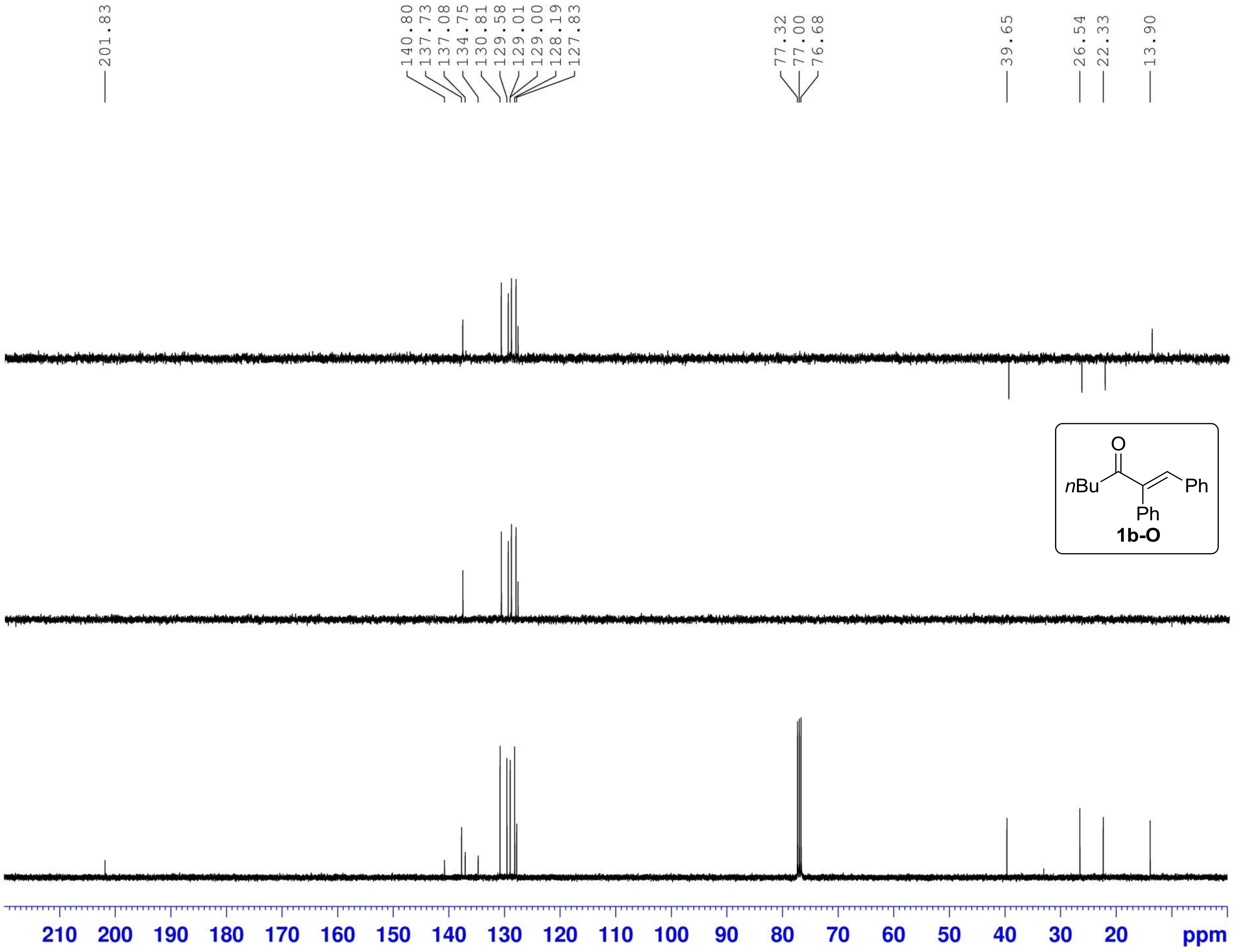
201.83
140.80
137.73
137.08
134.75
130.81
129.58
129.01
129.00
128.19
127.83
77.32
77.00
76.68
39.65
26.54
22.33
13.90
O
nBu
Ph
Ph
1b-O
210 200 190 180 170 160 150 140 130 120 110 100 90 80 70 60 50 40 30 20 ppm

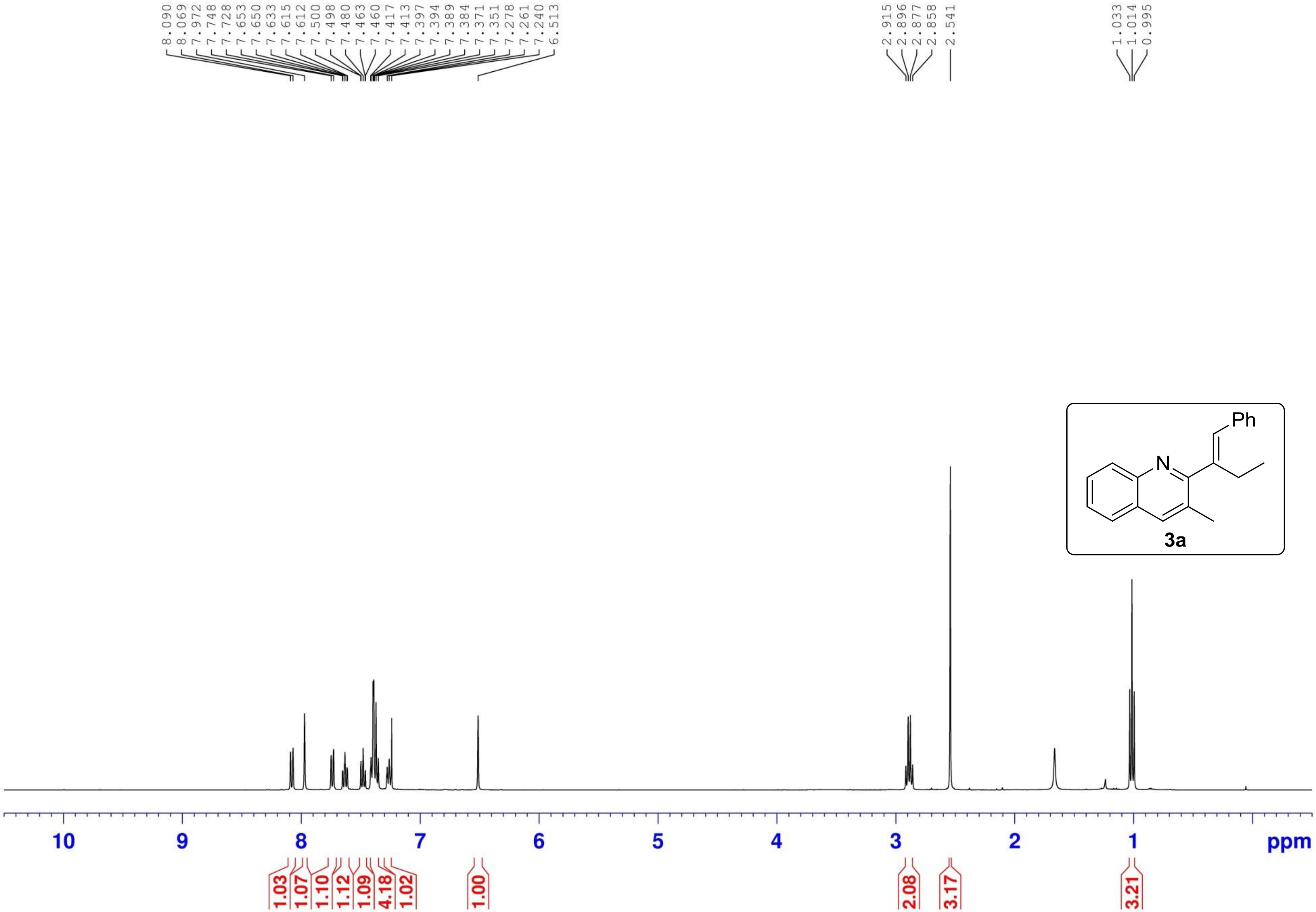
8.090
8.069
7.972
7.748
7.728
7.653
7.650
7.633
7.615
7.612
7.500
7.498
7.480
7.463
7.460
7.417
7.413
7.397
7.394
7.389
7.384
7.371
7.351
7.278
7.261
7.240
6.513
2.915
2.896
2.877
2.858
2.541
1.033
1.014
0.995
Ph
N
3a
10 9 8 7 6 5 4 3 2 1 ppm
1.03 1.07 1.10 1.12 1.09 4.18 1.02 1.00 2.08 3.17 3.21

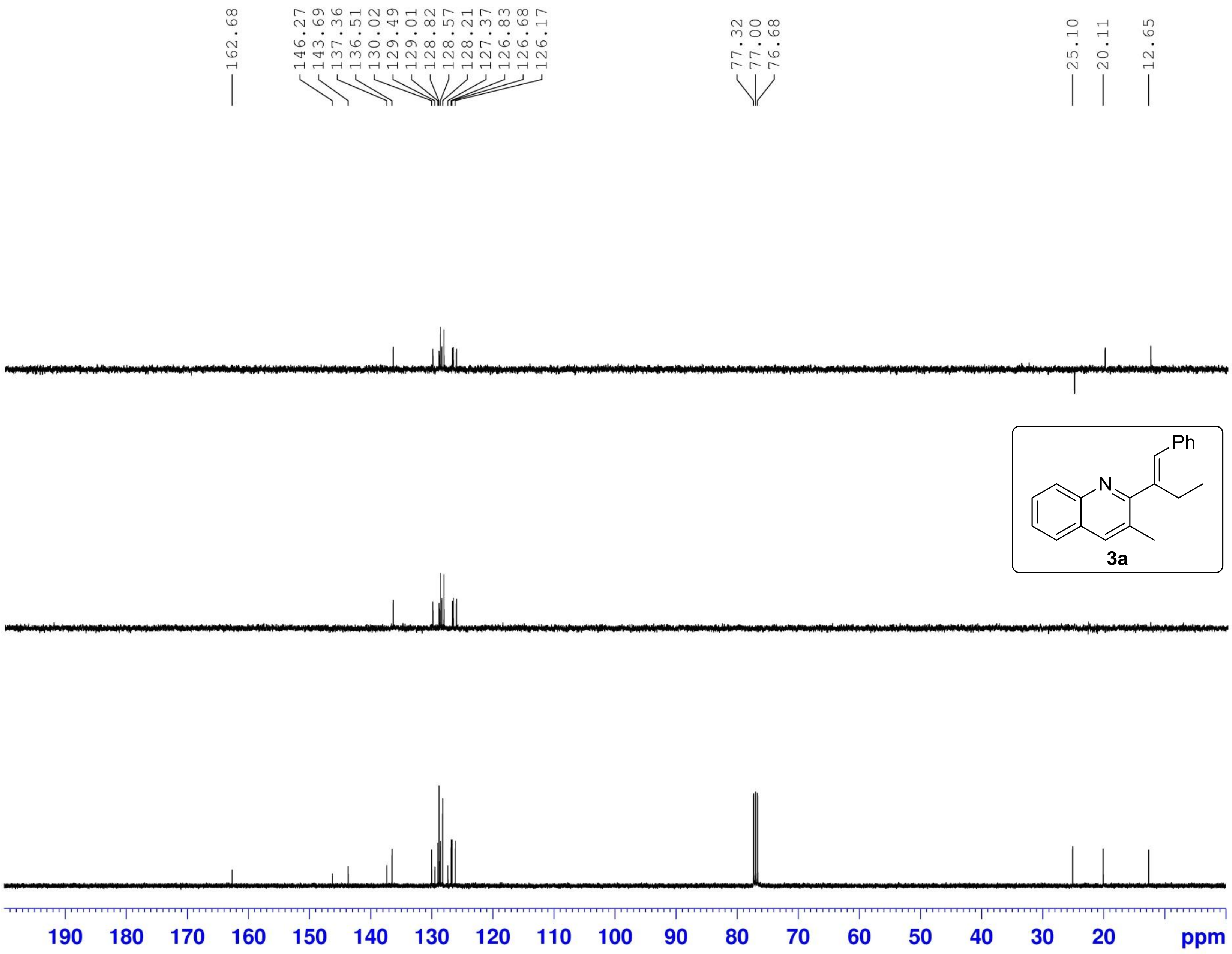
162.68
146.27
143.69
137.36
136.51
130.02
129.49
129.01
128.82
128.57
128.21
127.37
126.83
126.68
126.17
77.32
77.00
76.68
25.10
20.11
12.65
190
180
170
160
150
140
130
120
110
100
90
80
70
60
50
40
30
20
ppm
Ph
N

3a

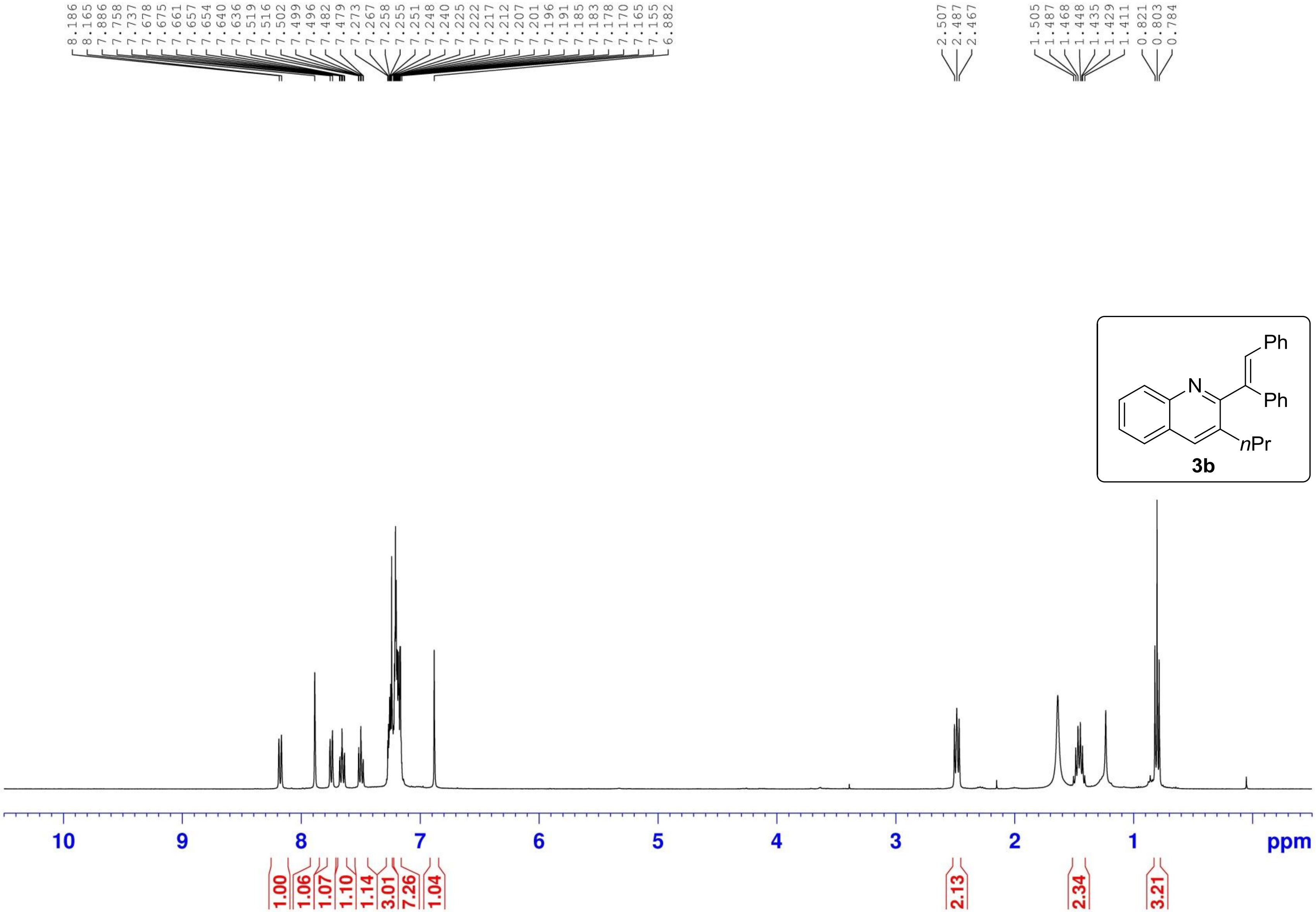
8.186
8.165
7.886
7.758
7.737
7.678
7.675
7.661
7.657
7.654
7.640
7.636
7.519
7.516
7.502
7.499
7.496
7.482
7.479
7.273
7.267
7.258
7.255
7.251
7.248
7.240
7.225
7.222
7.217
7.212
7.207
7.201
7.196
7.191
7.185
7.183
7.178
7.170
7.165
7.155
6.882
2.507
2.487
2.467
1.505
1.487
1.468
1.448
1.435
1.429
1.411
0.821
0.803
0.784
Ph
N
Ph
nPr
3b
10
9
8
7
6
5
4
3
2
1
ppm
1.00
1.06
1.07
1.10
1.14
3.01
7.26
1.04
2.13
2.34
3.21

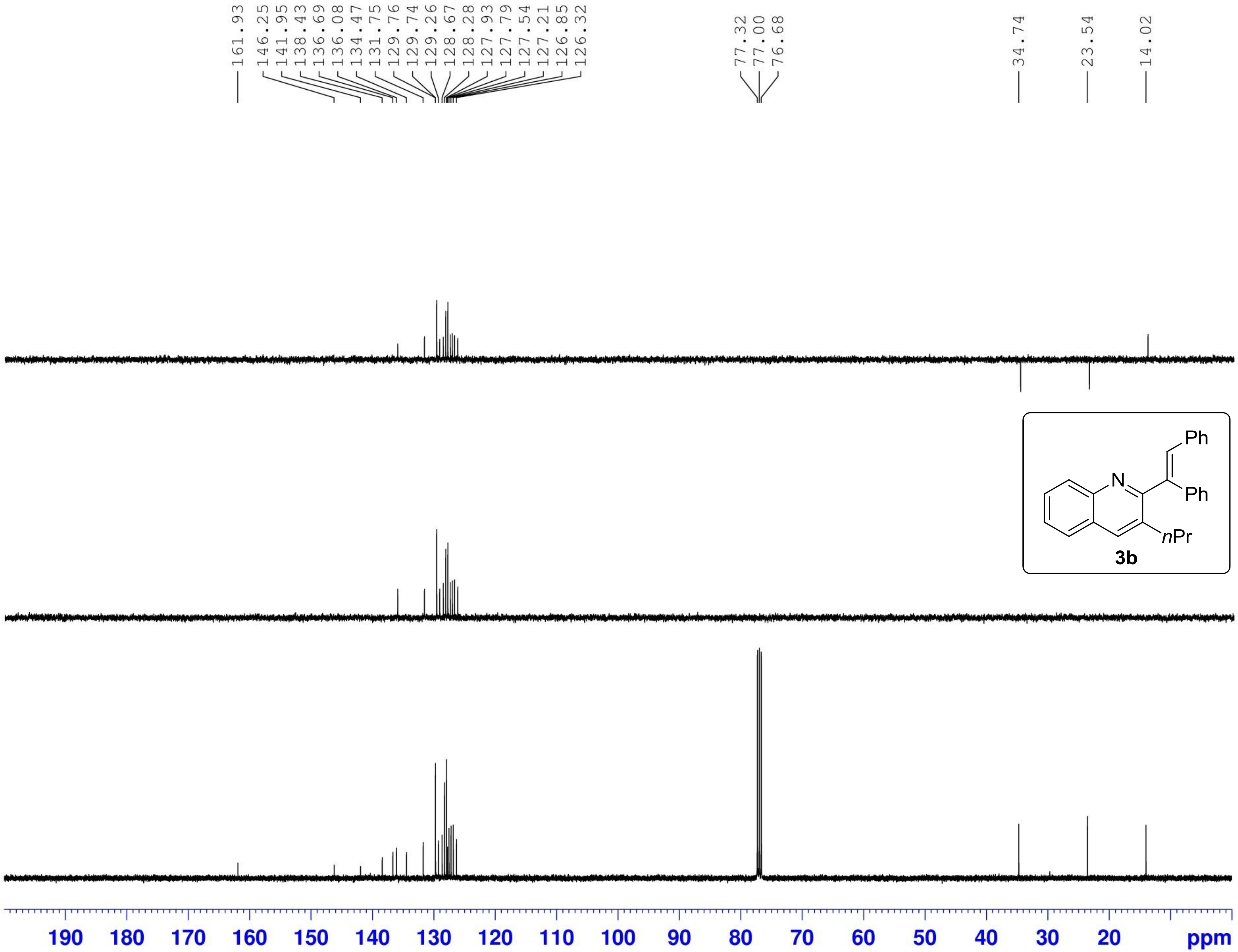
161.93
146.25
141.95
138.43
136.69
136.08
134.47
131.75
129.76
129.74
129.26
128.67
128.28
127.93
127.79
127.54
127.21
126.85
126.32
77.32
77.00
76.68
34.74
23.54
14.02
Ph
N
Ph
nPr
3b
190 180 170 160 150 140 130 120 110 100 90 80 70 60 50 40 30 20 ppm

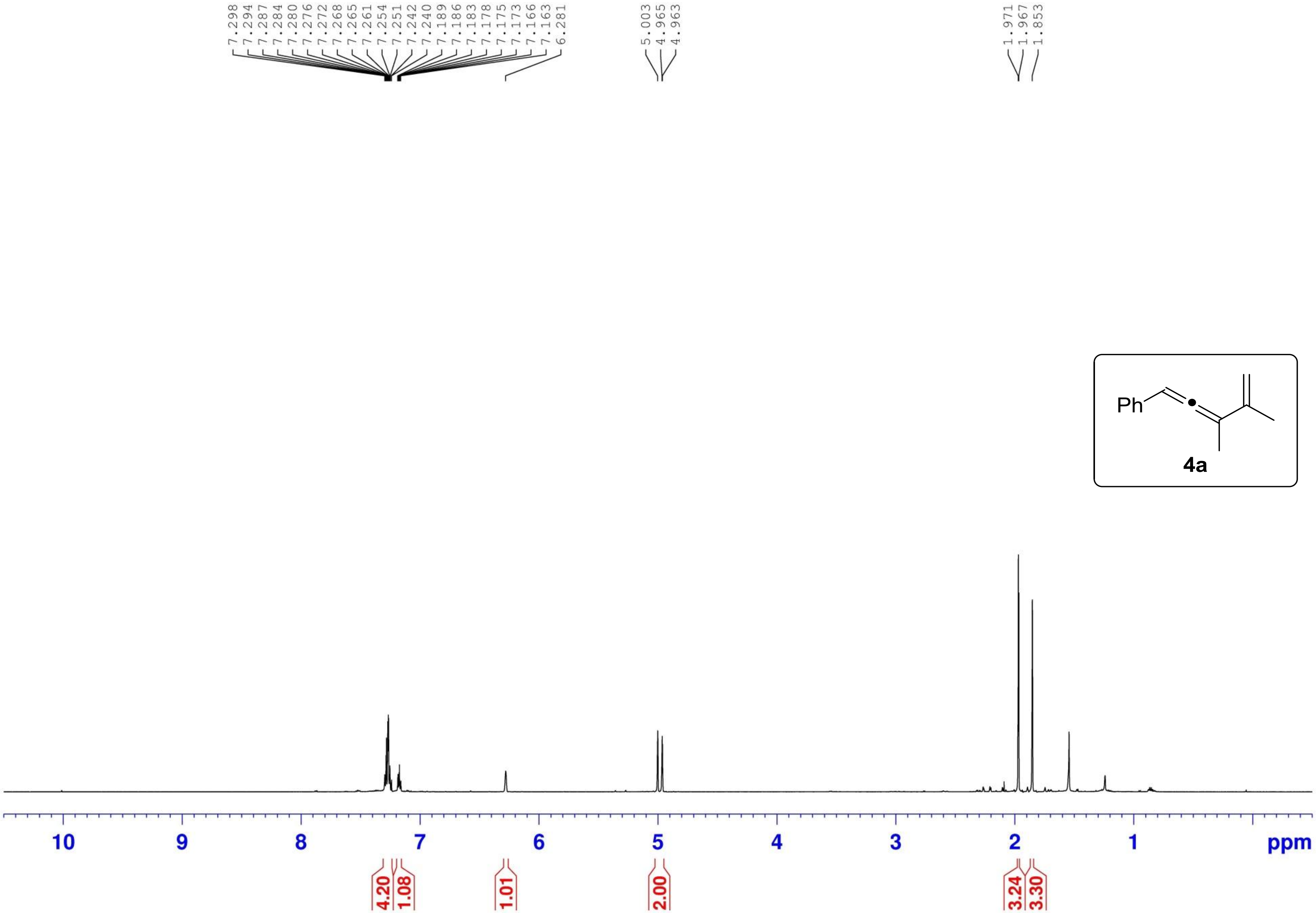
7.298
7.294
7.287
7.284
7.280
7.276
7.272
7.268
7.265
7.261
7.254
7.251
7.242
7.240
7.189
7.186
7.183
7.178
7.175
7.173
7.166
7.163
6.281
5.003
4.965
4.963
1.971
1.967
1.853
Ph
4a
10
9
8
7
6
5
4
3
2
1
ppm
4.20
1.08
1.01
2.00
3.24
3.30

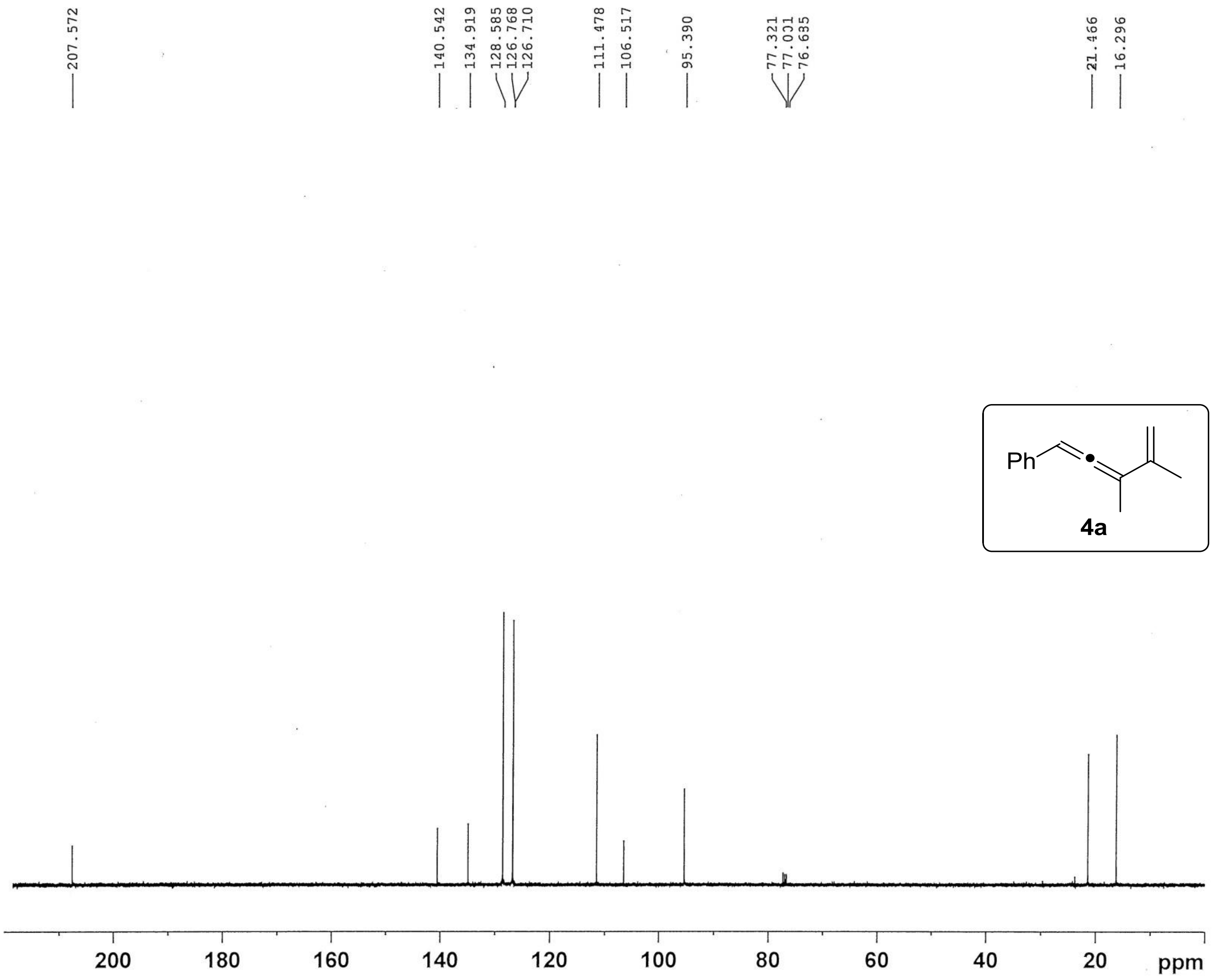
207.572
140.542
134.919
128.585
126.768
126.710
111.478
106.517
95.390
77.321
77.001
76.635
21.466
16.296
Ph
4a
200
180
160
140
120
100
80
60
40
20
ppm

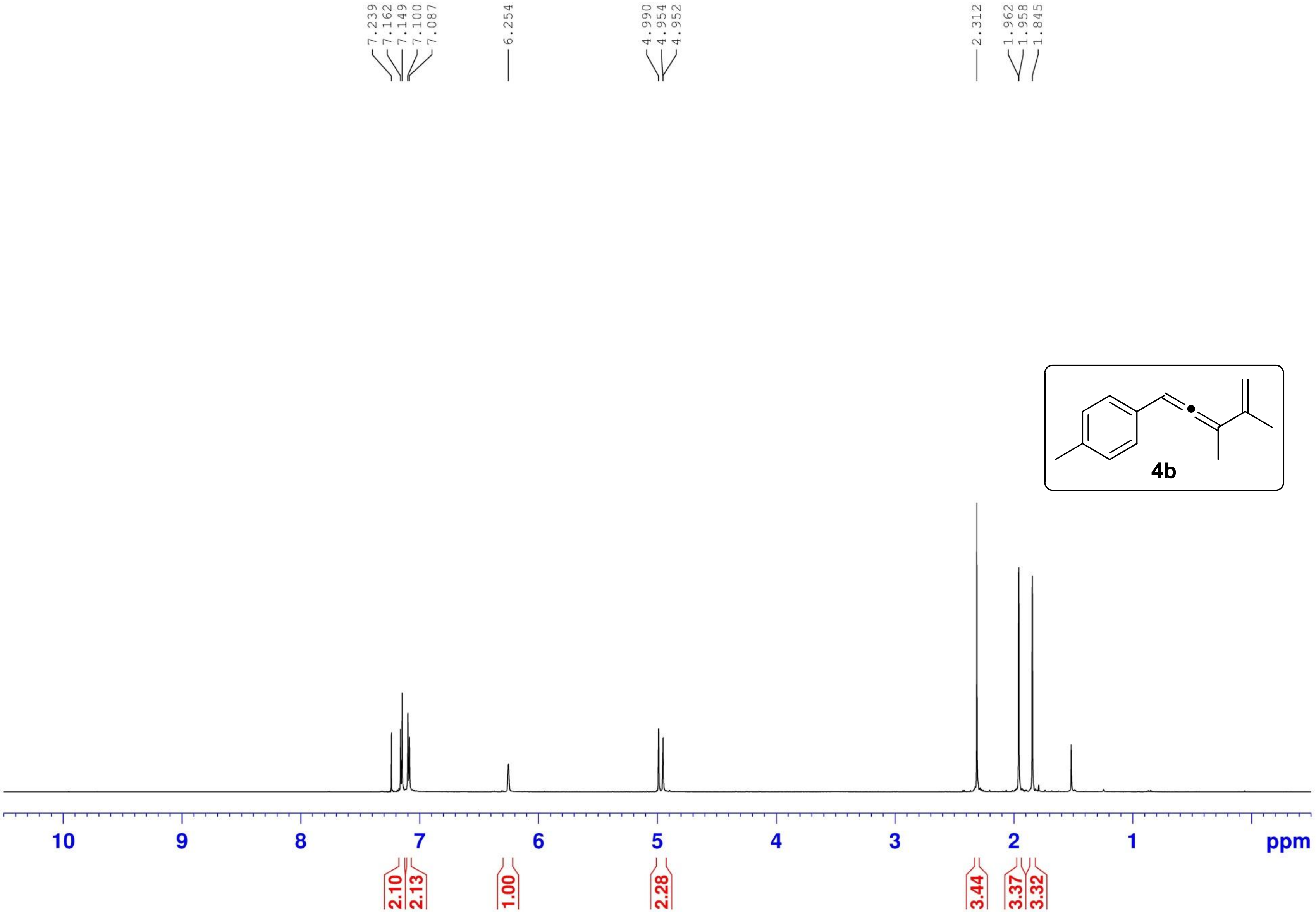
7.239
7.162
7.149
7.100
7.087
6.254
4.990
4.954
4.952
2.312
1.962
1.958
1.845
4b
10
9
8
7
6
5
4
3
2
1
ppm
2.10
2.13
1.00
2.28
3.44
3.37
3.32

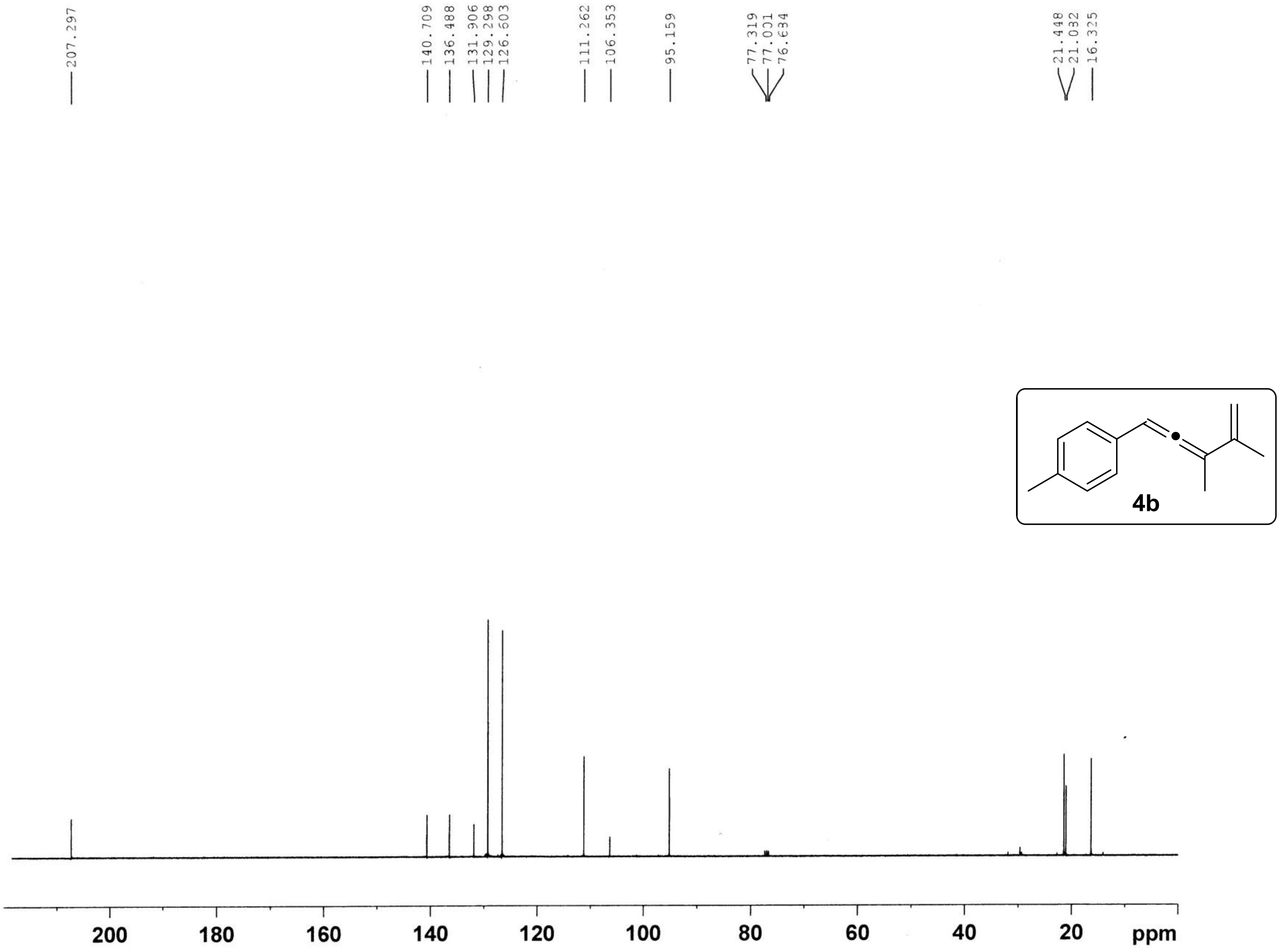
207.297
140.709
136.488
131.906
129.298
126.603
111.262
106.353
95.159
77.319
77.001
76.634
21.448
21.032
16.325
200
180
160
140
120
100
80
60
40
20
ppm

4b

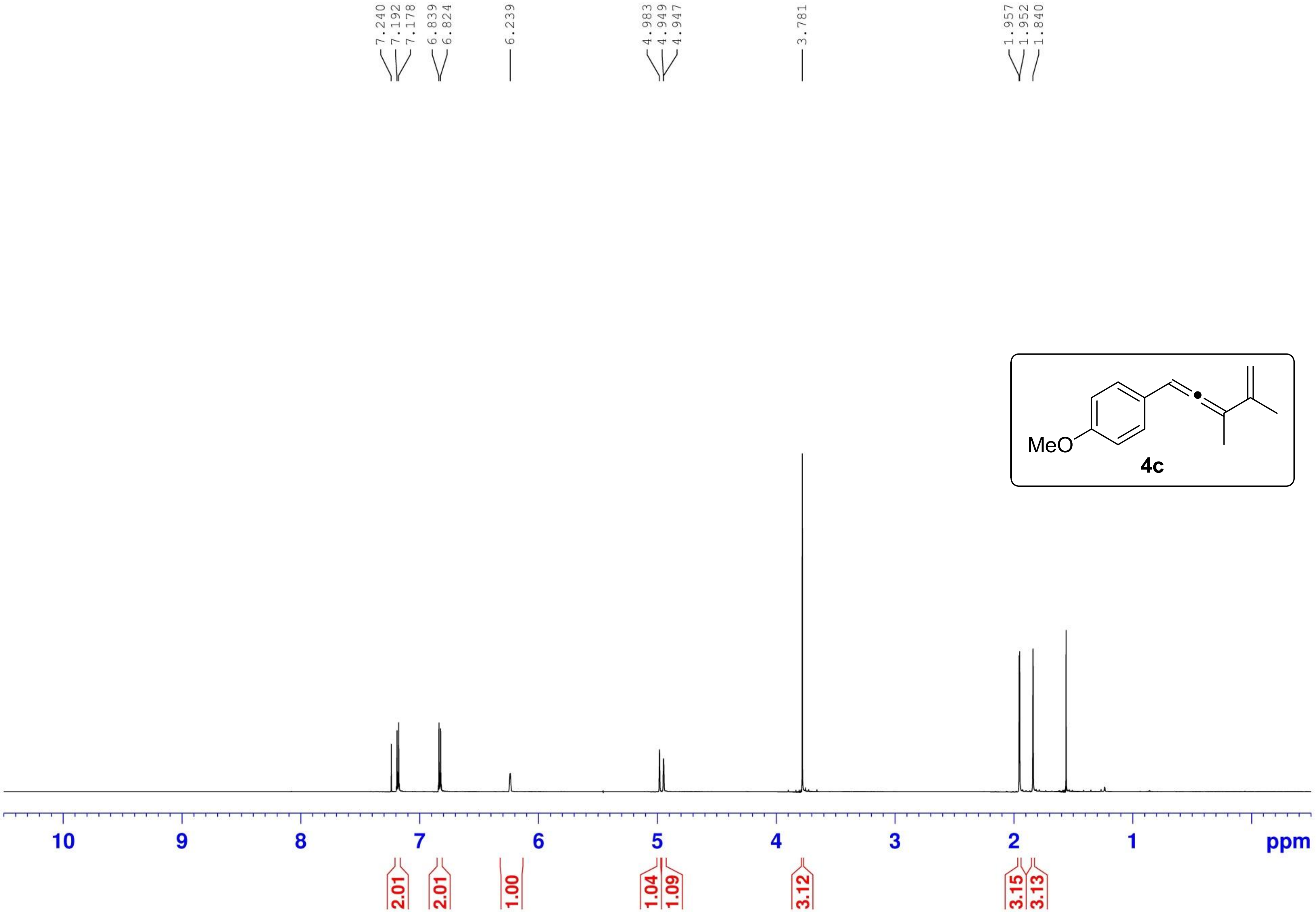
7.240
7.192
7.178
6.839
6.824
6.239
4.983
4.949
4.947
3.781
1.957
1.952
1.840
MeO
4c
10
9
8
7
6
5
4
3
2
1
ppm
2.01
2.01
1.00
1.04
1.09
3.12
3.15
3.13

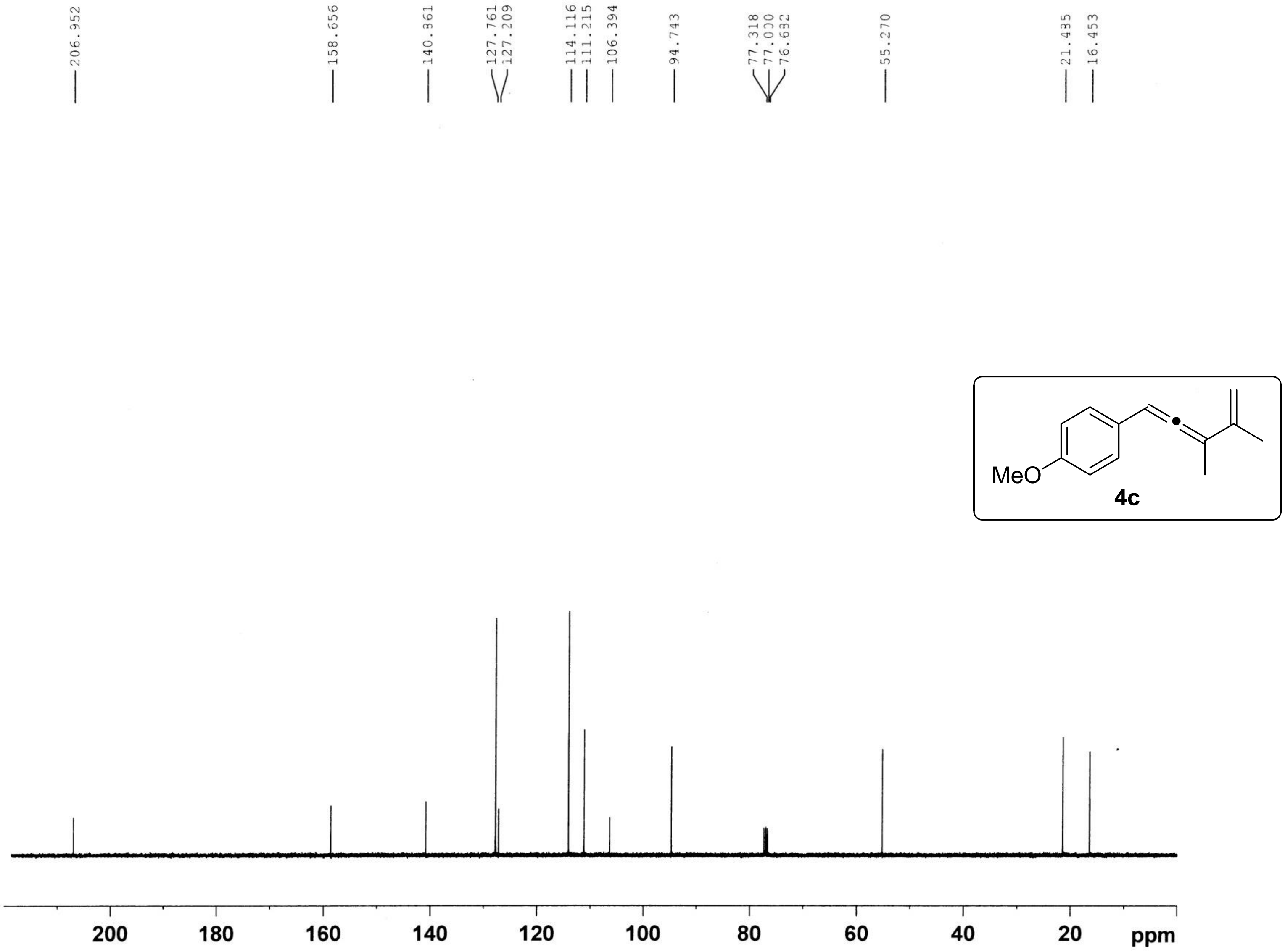
206.952
158.656
140.361
127.761
127.209
114.116
111.215
106.394
94.743
77.318
77.030
76.632
55.270
21.435
16.453
200
180
160
140
120
100
80
60
40
20
ppm
MeO

4c

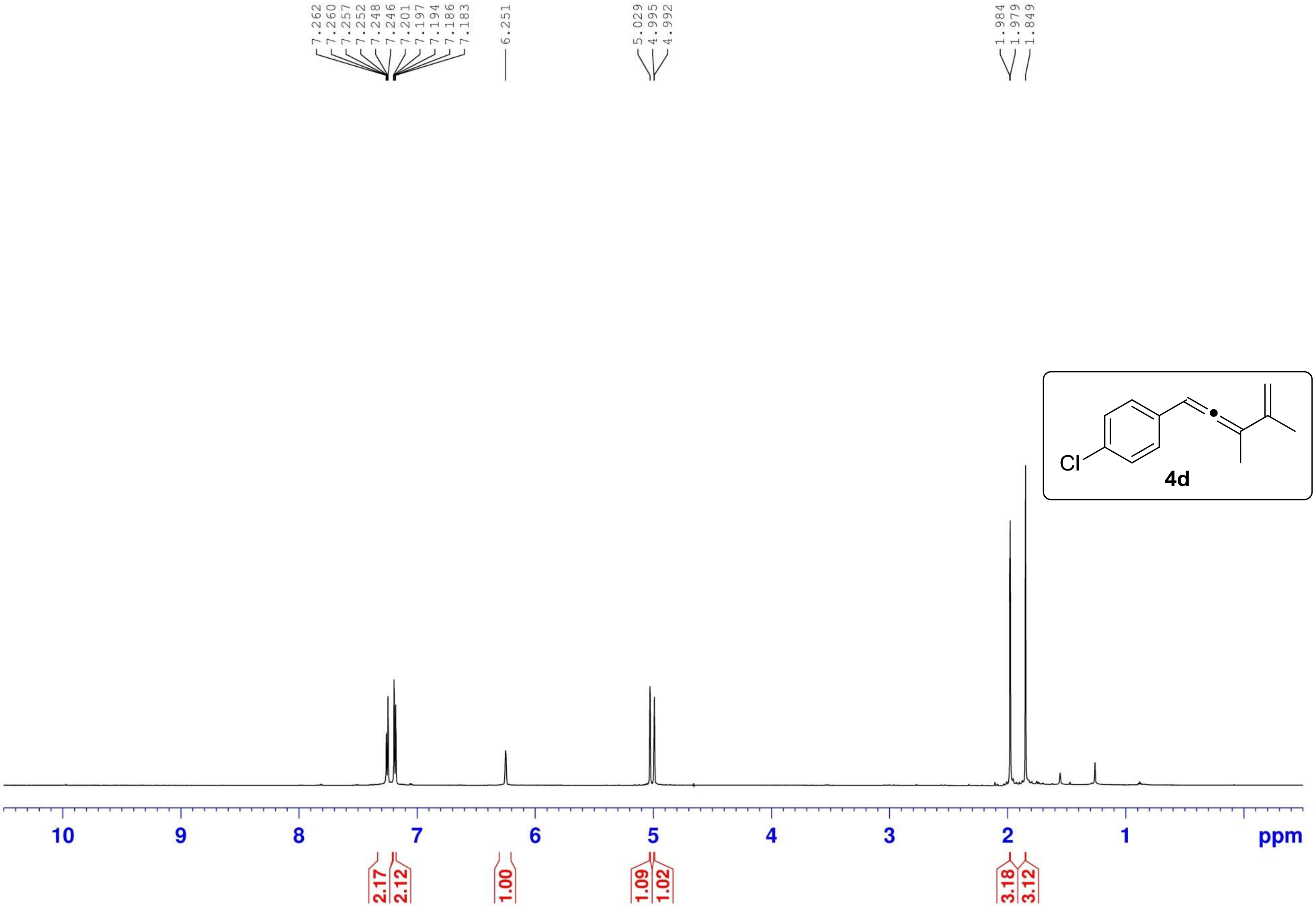
7.262
7.260
7.257
7.252
7.248
7.246
7.201
7.197
7.194
7.186
7.183
6.251
5.029
4.995
4.992
1.984
1.979
1.849
Cl
4d
10
9
8
7
6
5
4
3
2
1
ppm
2.17
2.12
1.00
1.09
1.02
3.18
3.12

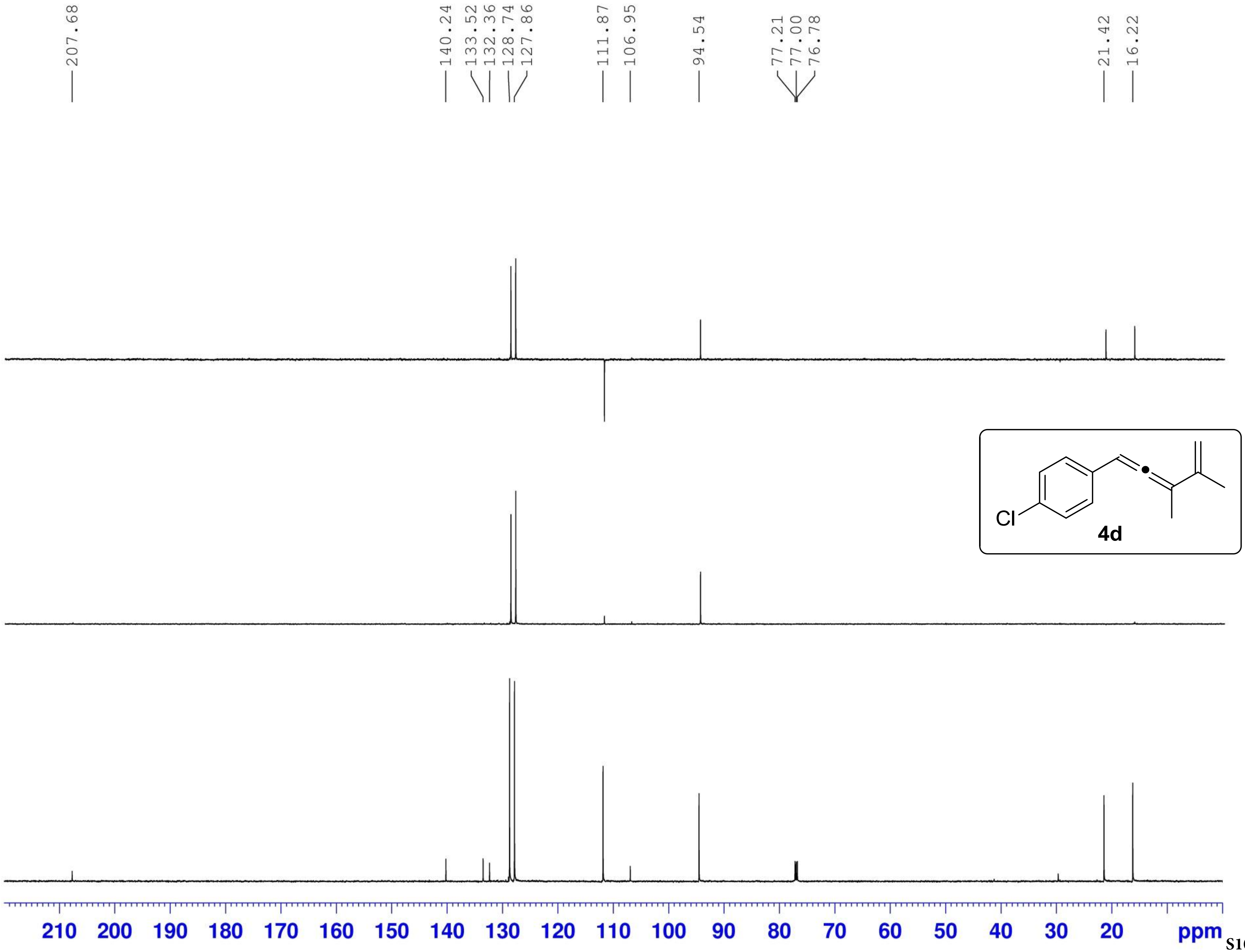

207.68
140.24
133.52
132.36
128.74
127.86
111.87
106.95
94.54
77.21
77.00
76.78
21.42
16.22
Cl
4d
210 200 190 180 170 160 150 140 130 120 110 100 90 80 70 60 50 40 30 20 ppm

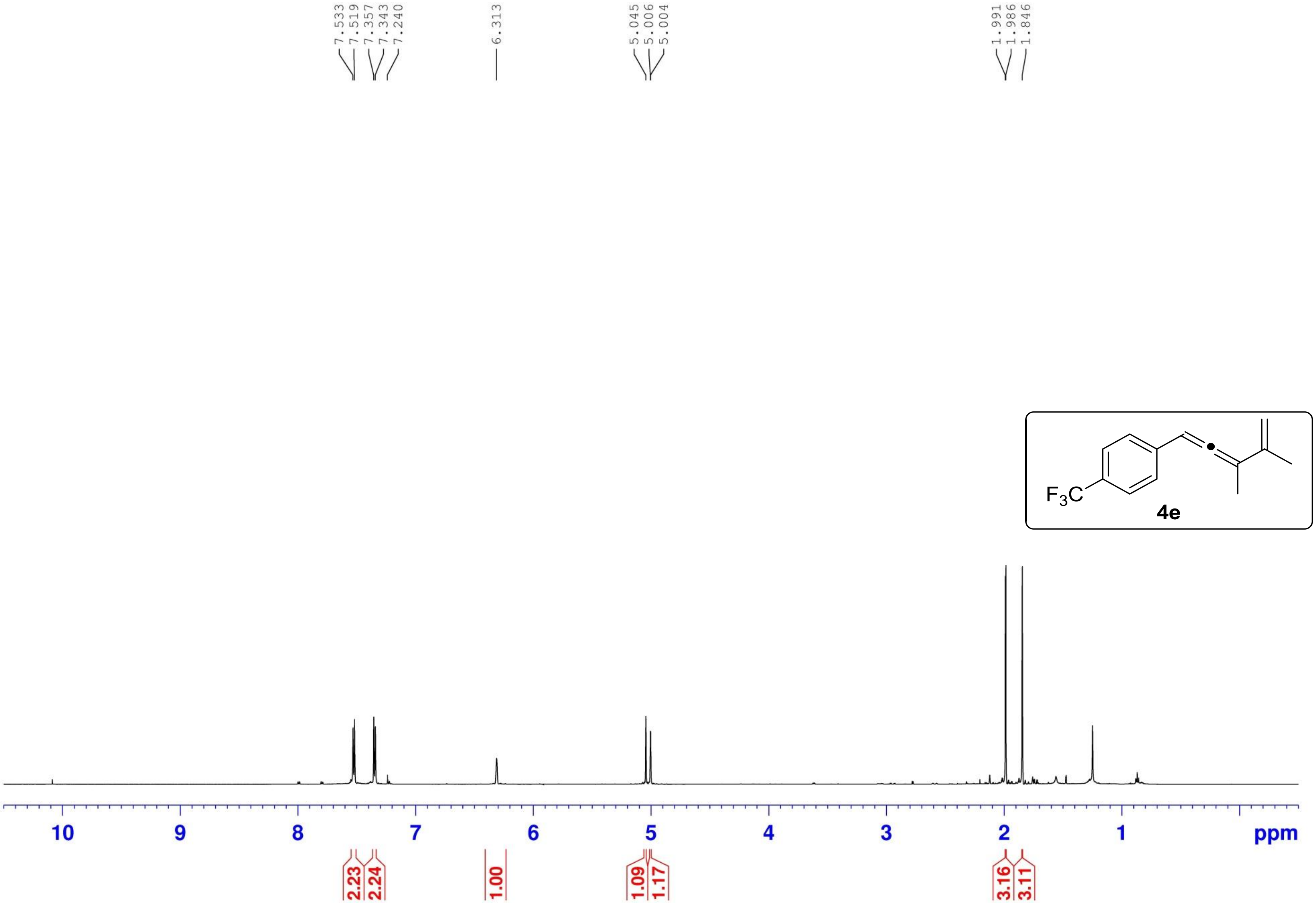
7.533
7.519
7.357
7.343
7.240
6.313
5.045
5.006
5.004
1.991
1.986
1.846
F3C
4e
10
9
8
7
6
5
4
3
2
1
ppm
2.23
2.24
1.00
1.09
1.17
3.16
3.11

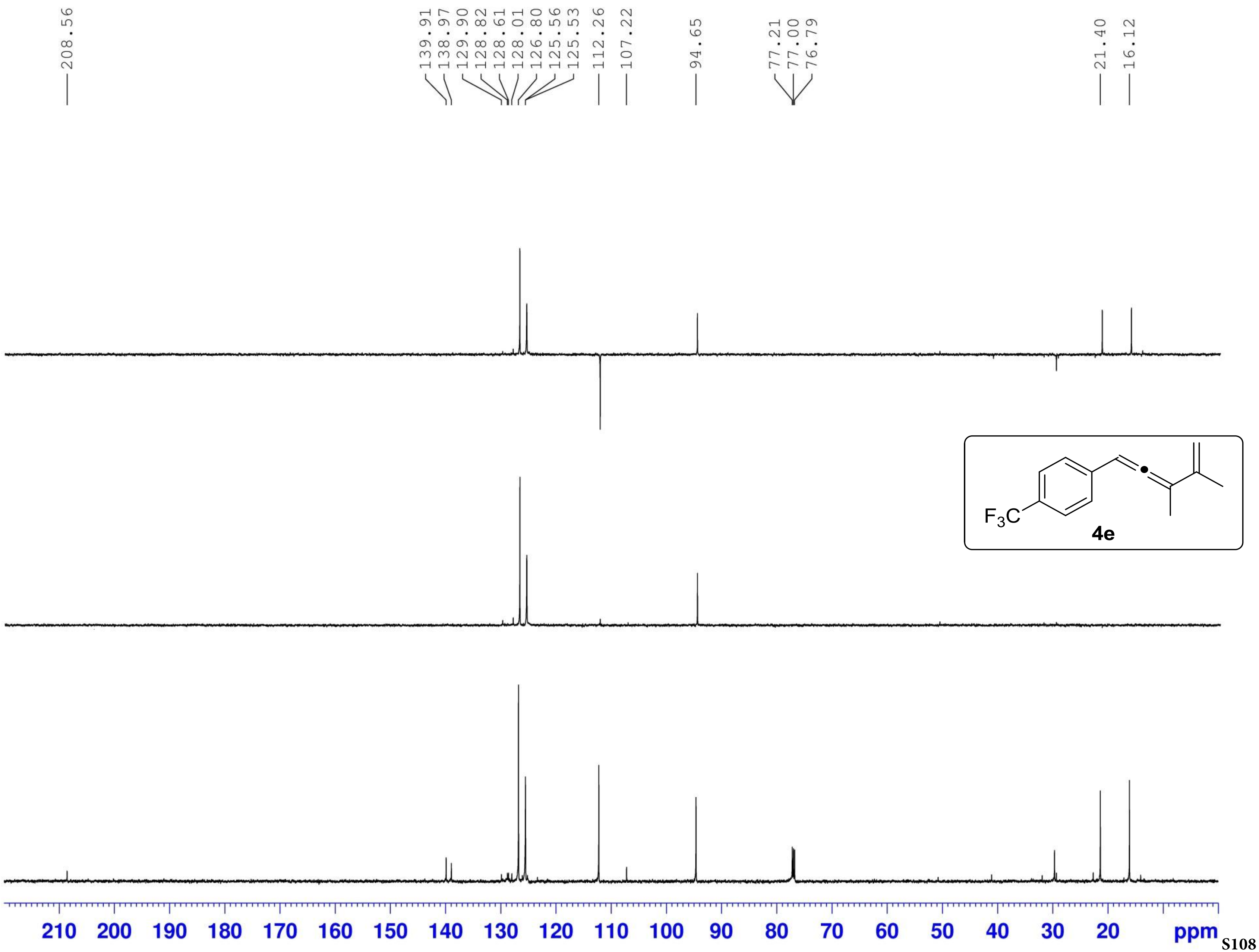
208.56
139.91
138.97
129.90
128.82
128.61
128.01
126.80
125.56
125.53
112.26
107.22
94.65
77.21
77.00
76.79
21.40
16.12
F3C
4e
210 200 190 180 170 160 150 140 130 120 110 100 90 80 70 60 50 40 30 20 ppm

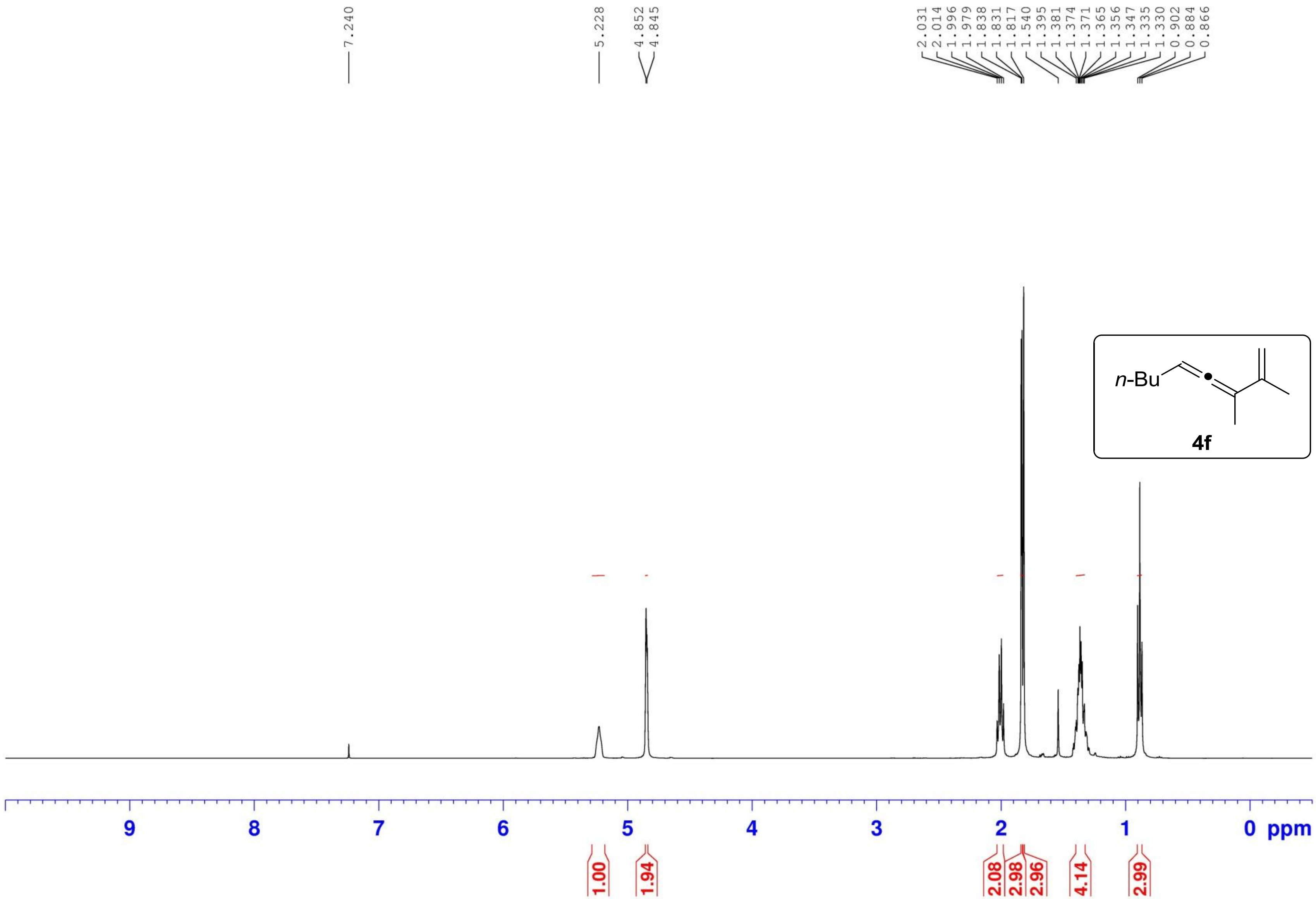
7.240
5.228
4.852
4.845
2.031
2.014
1.996
1.979
1.838
1.831
1.817
1.540
1.395
1.381
1.374
1.371
1.365
1.356
1.347
1.335
1.330
0.902
0.884
0.866
n-Bu
4f
9
8
7
6
5
4
3
2
1
0 ppm
1.00
1.94
2.08
2.98
2.96
4.14
2.99

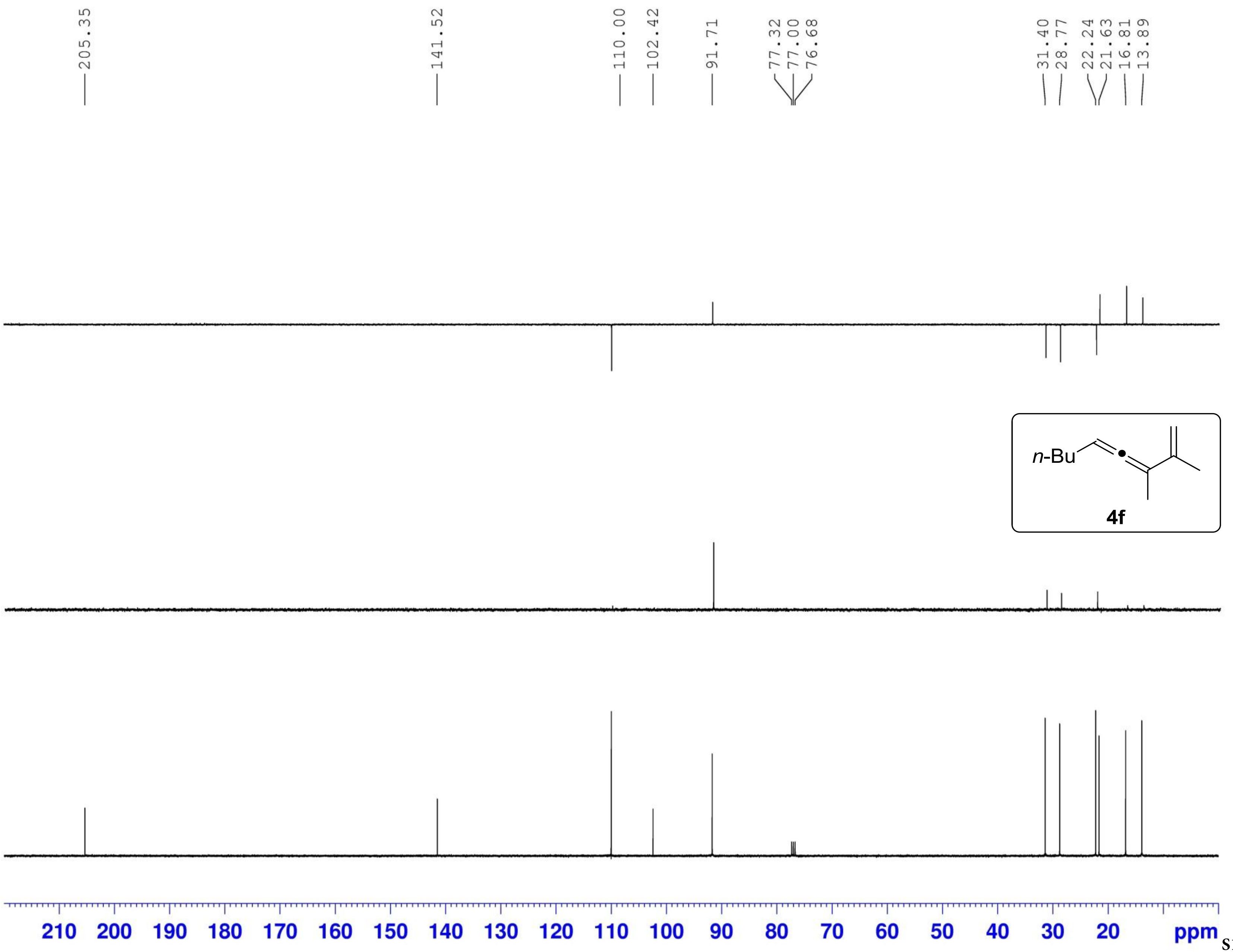
205.35
141.52
110.00
102.42
91.71
77.32
77.00
76.68
31.40
28.77
22.24
21.63
16.81
13.89
n-Bu
4f
210 200 190 180 170 160 150 140 130 120 110 100 90 80 70 60 50 40 30 20 ppm

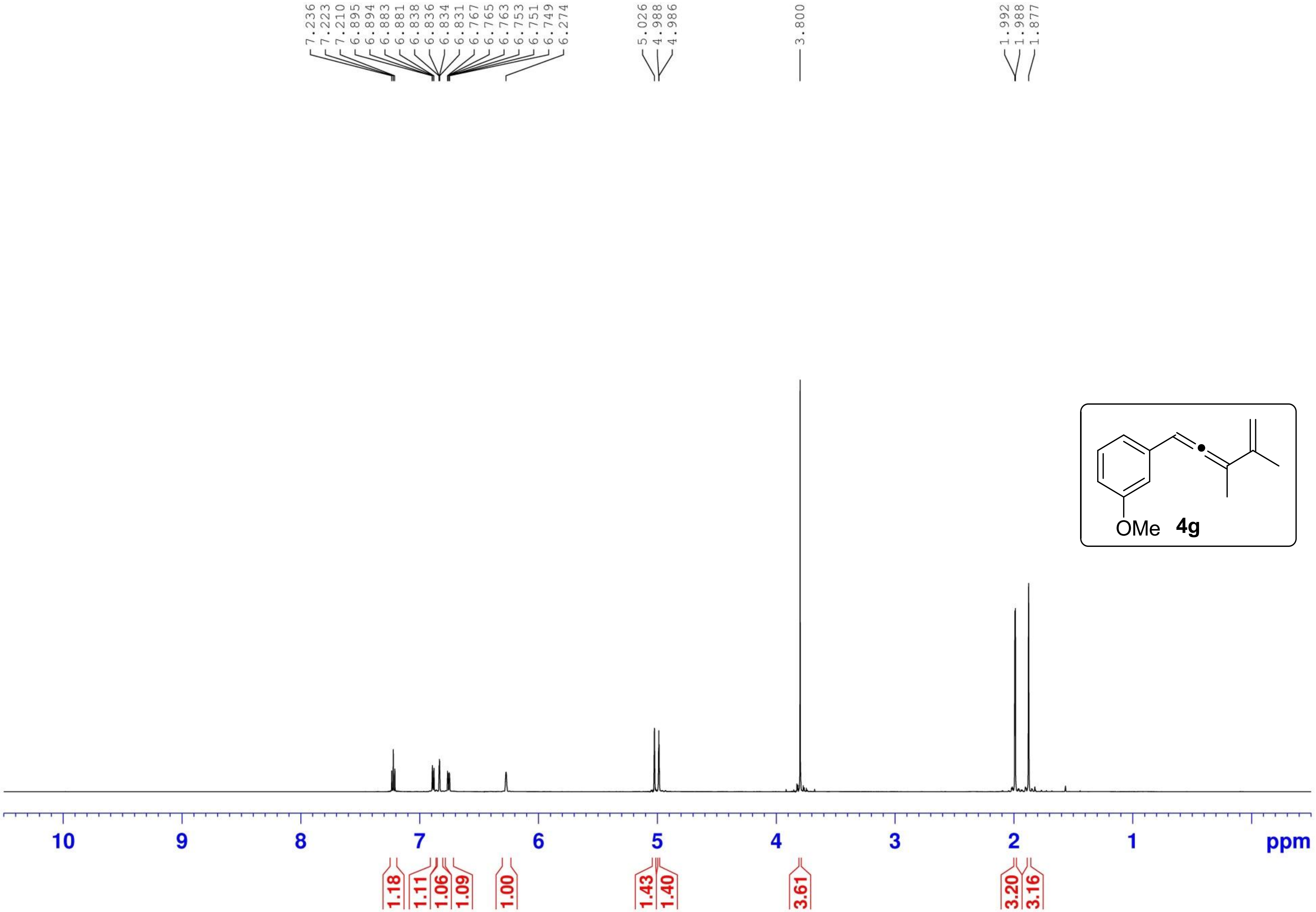
7.236
7.223
7.210
6.895
6.894
6.883
6.881
6.838
6.836
6.834
6.831
6.767
6.765
6.763
6.753
6.751
6.749
6.274
5.026
4.988
4.986
3.800
1.992
1.988
1.877
OMe
4g
10
9
8
7
6
5
4
3
2
1
ppm
1.18
1.11
1.06
1.09
1.00
1.43
1.40
3.61
3.20
3.16

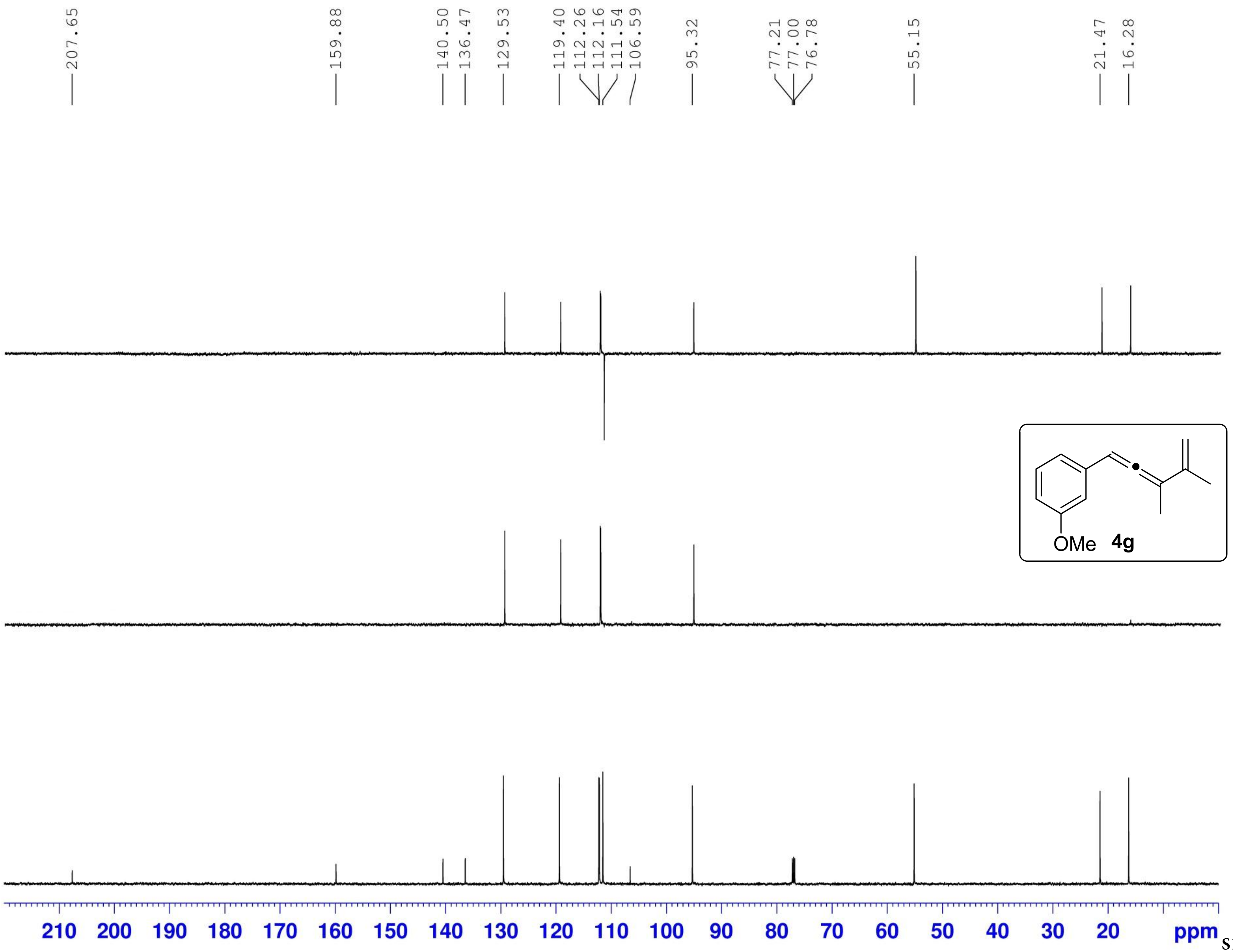
207.65
159.88
140.50
136.47
129.53
119.40
112.26
112.16
111.54
106.59
95.32
77.21
77.00
76.78
55.15
21.47
16.28
OMe
4g
210 200 190 180 170 160 150 140 130 120 110 100 90 80 70 60 50 40 30 20 ppm

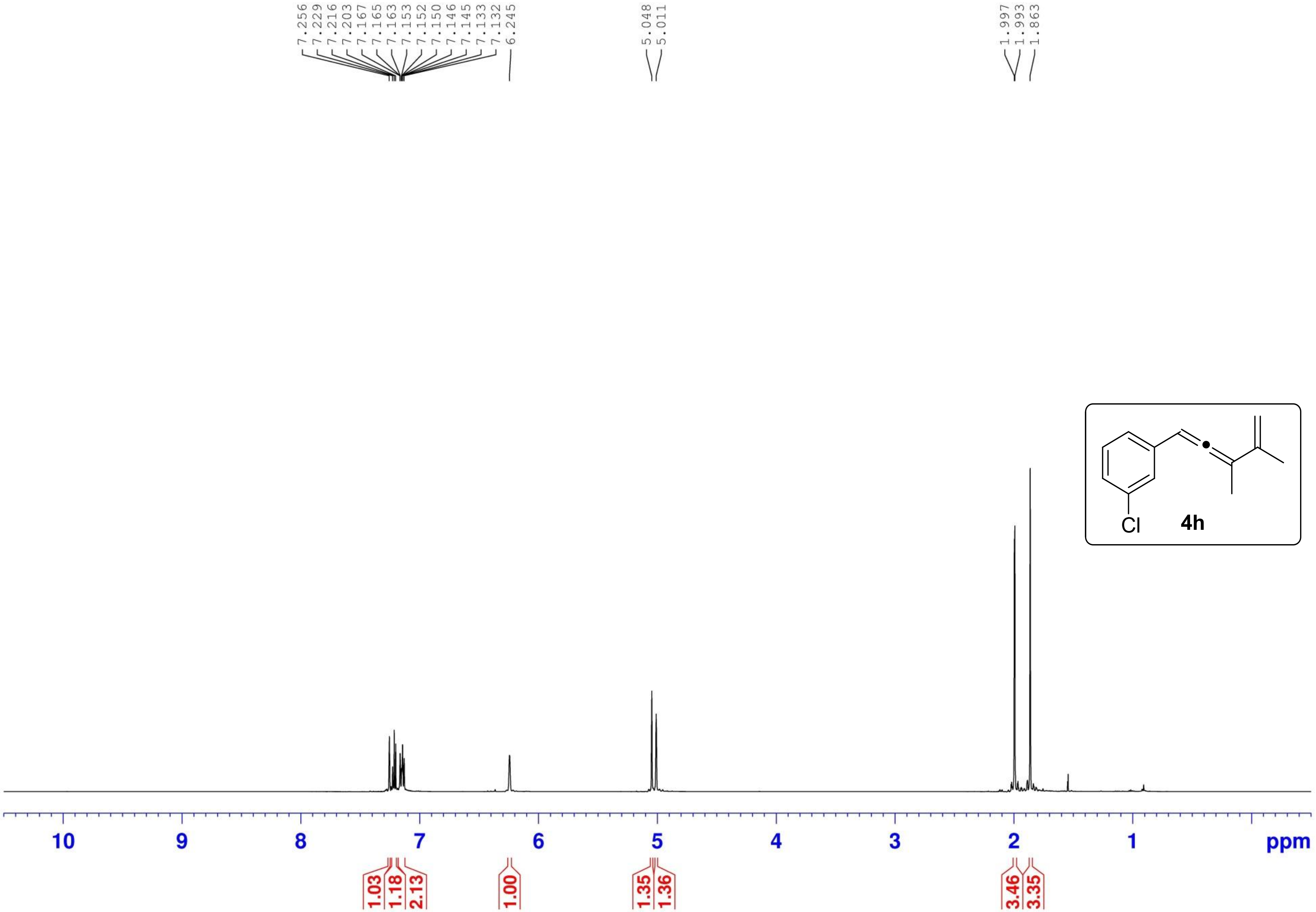
7.256
7.229
7.216
7.203
7.167
7.165
7.163
7.153
7.152
7.150
7.146
7.145
7.133
7.132
6.245
5.048
5.011
1.997
1.993
1.863
Cl
4h
10
9
8
7
6
5
4
3
2
1
ppm
1.03
1.18
2.13
1.00
1.35
1.36
3.46
3.35

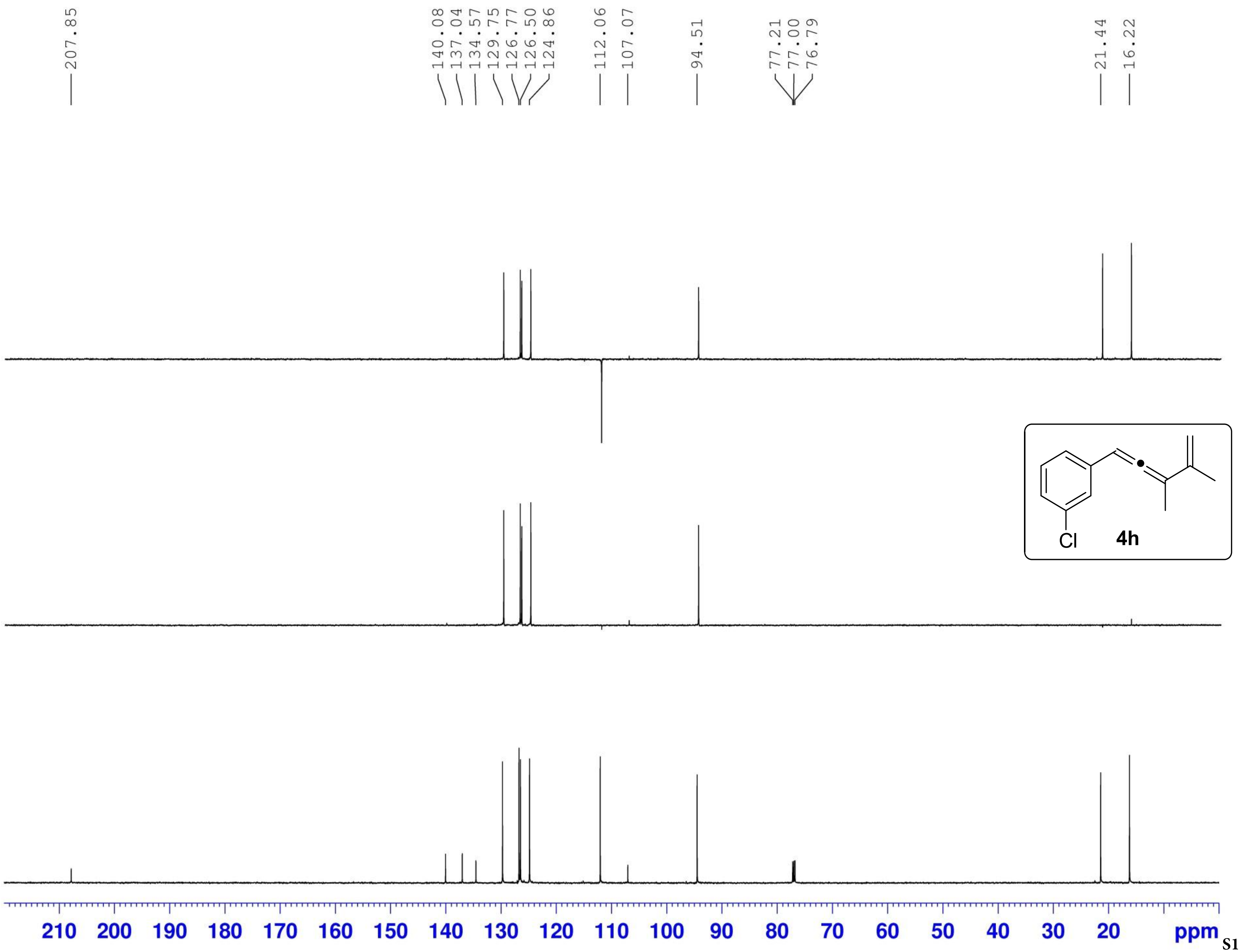
207.85
140.08
137.04
134.57
129.75
126.77
126.50
124.86
112.06
107.07
94.51
77.21
77.00
76.79
21.44
16.22
Cl
4h
210 200 190 180 170 160 150 140 130 120 110 100 90 80 70 60 50 40 30 20 ppm

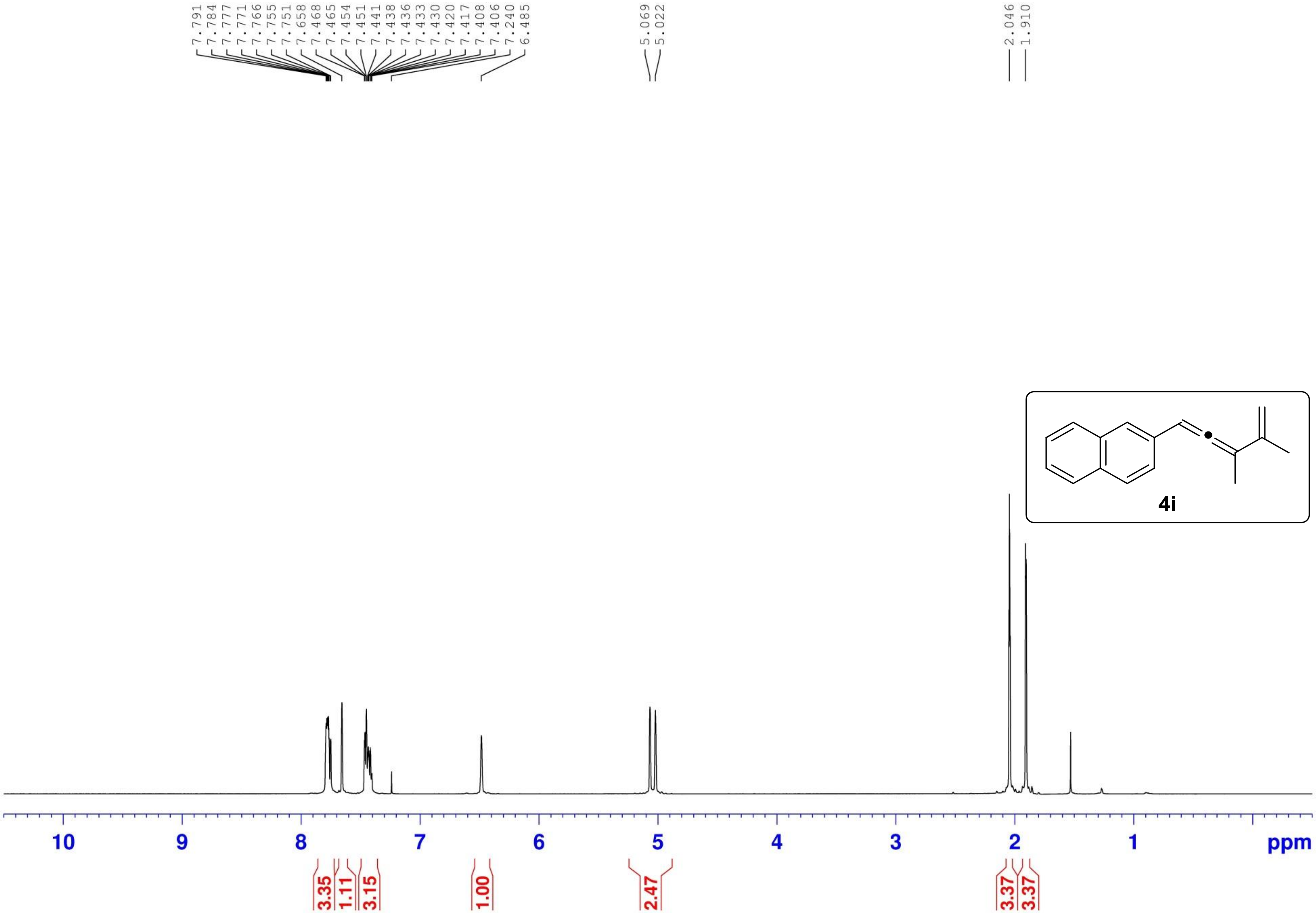
7.791
7.784
7.777
7.771
7.766
7.755
7.751
7.658
7.468
7.465
7.454
7.451
7.441
7.438
7.436
7.433
7.430
7.420
7.417
7.408
7.406
7.240
6.485
5.069
5.022
2.046
1.910
4i
10
9
8
7
6
5
4
3
2
1
ppm
3.35
1.11
3.15
1.00
2.47
3.37
3.37

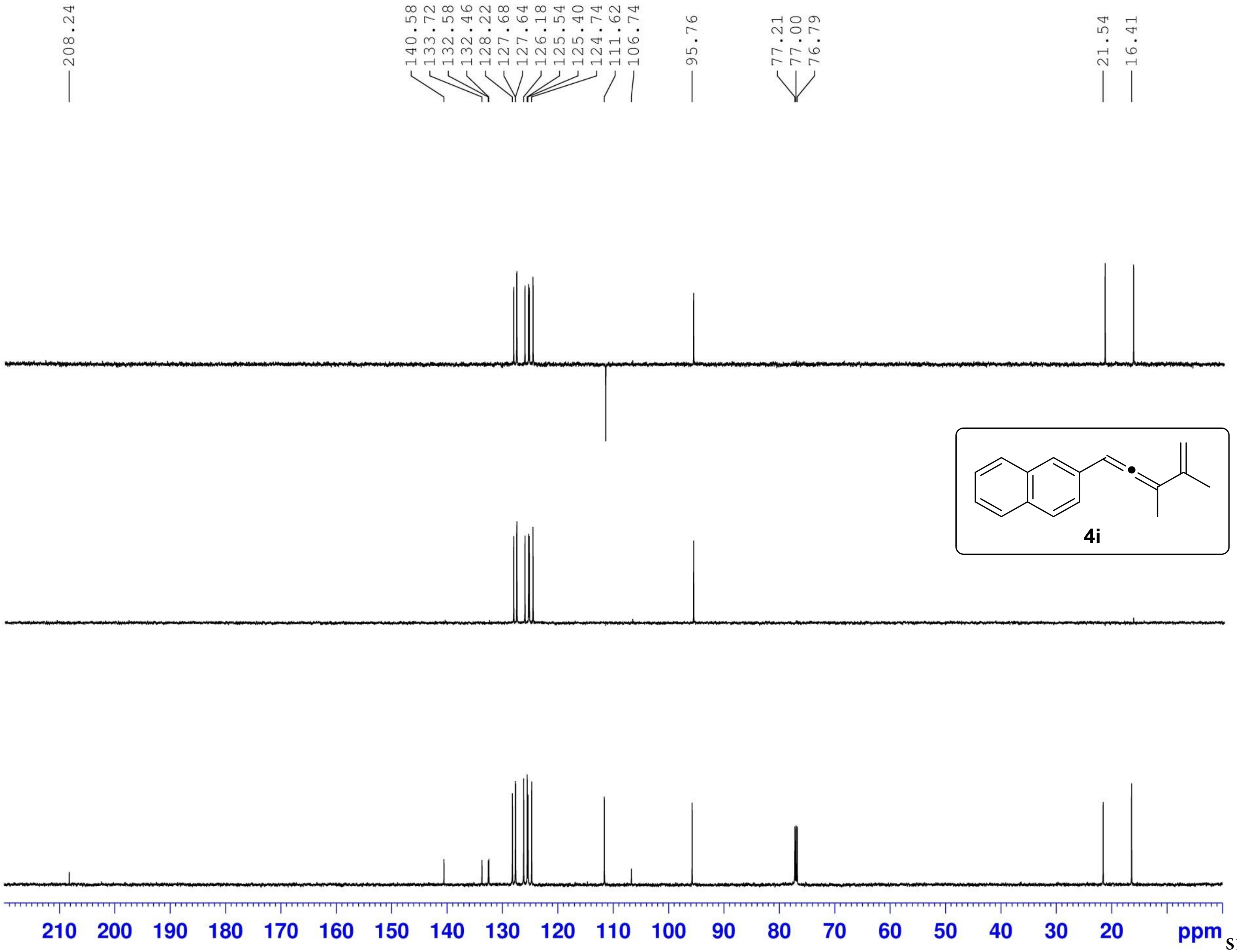
208.24
140.58
133.72
132.58
132.46
128.22
127.68
127.64
126.18
125.54
125.40
124.74
111.62
106.74
95.76
77.21
77.00
76.79
21.54
16.41
4i
210 200 190 180 170 160 150 140 130 120 110 100 90 80 70 60 50 40 30 20 ppm

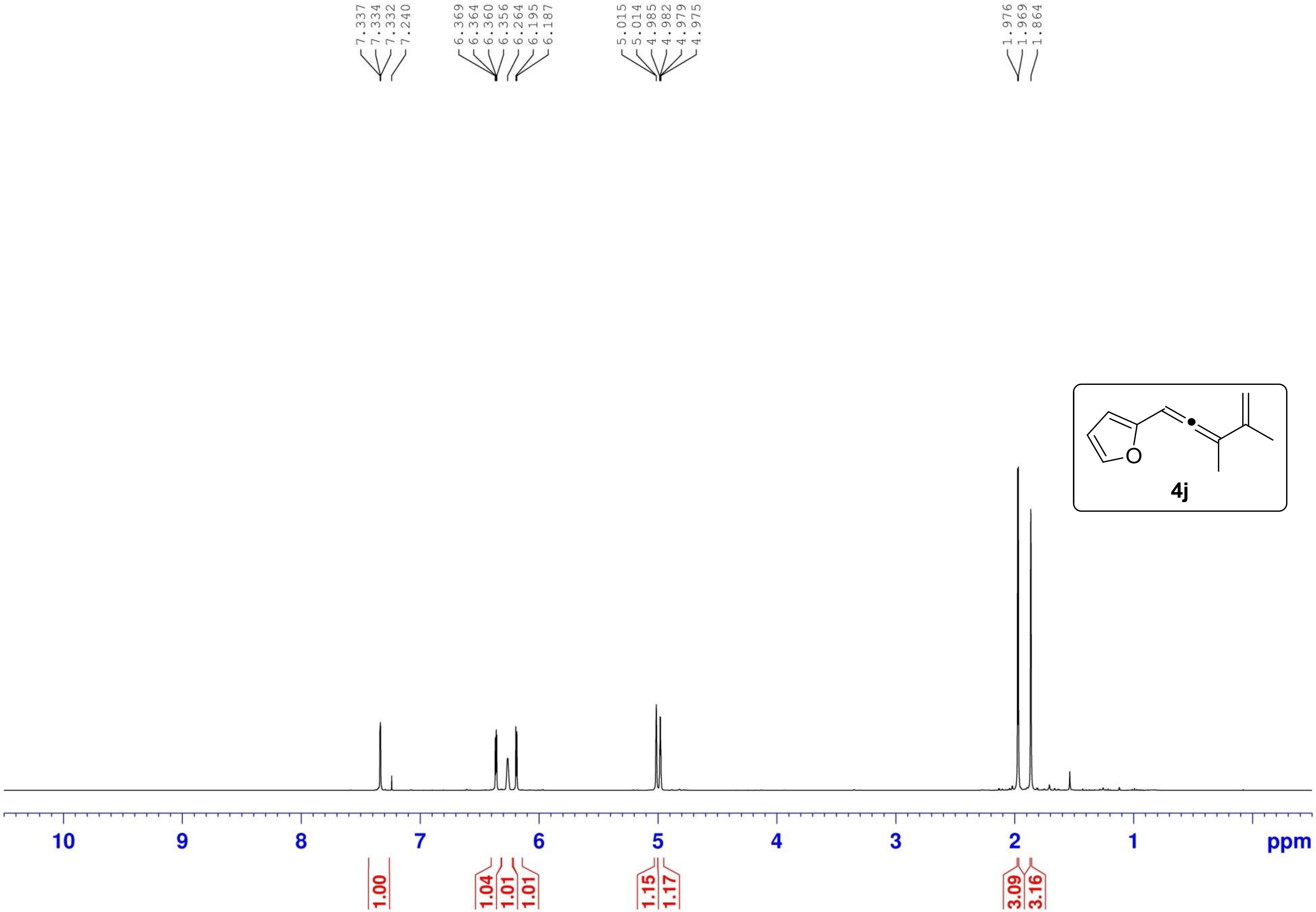
7.337
7.334
7.332
7.240
6.369
6.364
6.360
6.356
6.264
6.195
6.187
5.015
5.014
4.985
4.982
4.979
4.975
1.976
1.969
1.864
4j
10
9
8
7
6
5
4
3
2
1
ppm
1.00
1.04
1.01
1.01
1.15
1.17
3.09
3.16

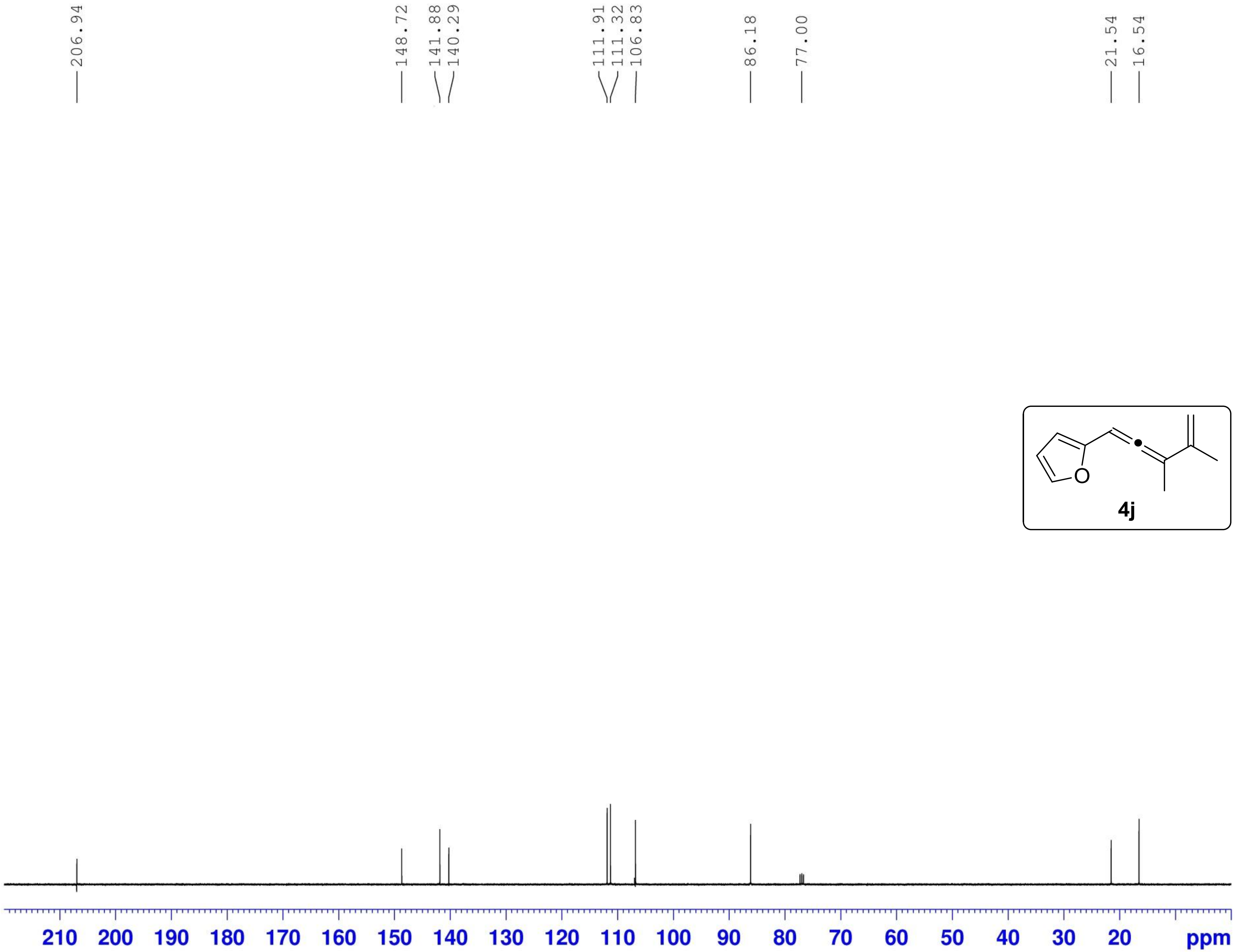
206.94
148.72
141.88
140.29
111.91
111.32
106.83
86.18
77.00
21.54
16.54
O
4j
210 200 190 180 170 160 150 140 130 120 110 100 90 80 70 60 50 40 30 20 ppm

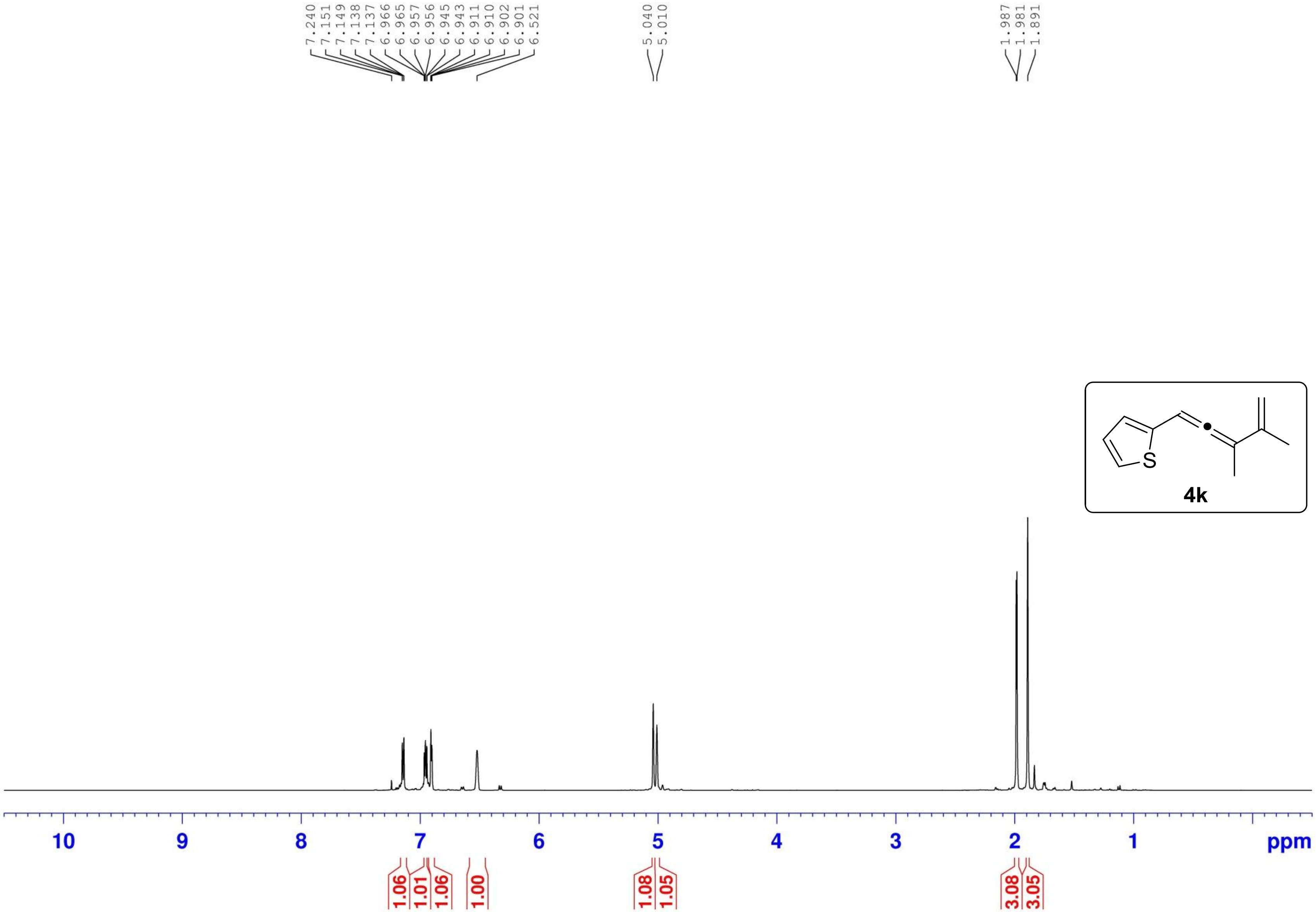
7.240
7.151
7.149
7.138
7.137
6.966
6.965
6.957
6.956
6.945
6.943
6.911
6.910
6.902
6.901
6.521
5.040
5.010
1.987
1.981
1.891
S
4k
10
9
8
7
6
5
4
3
2
1
ppm
1.06
1.01
1.06
1.00
1.08
1.05
3.08
3.05

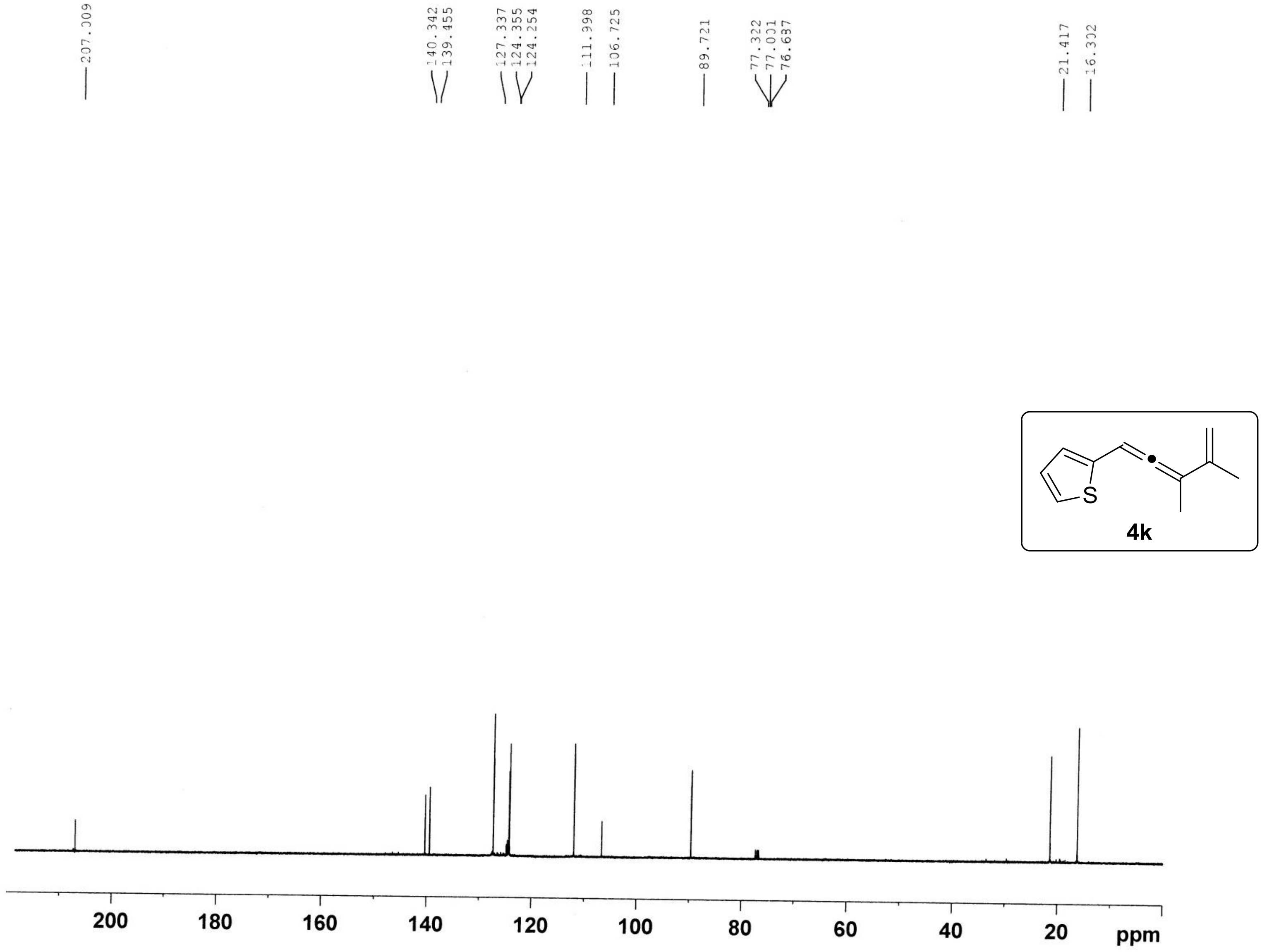
207.309
140.342
139.455
127.337
124.355
124.254
111.998
106.725
89.721
77.322
77.001
76.637
21.417
16.302
200
180
160
140
120
100
80
60
40
20
ppm

4k

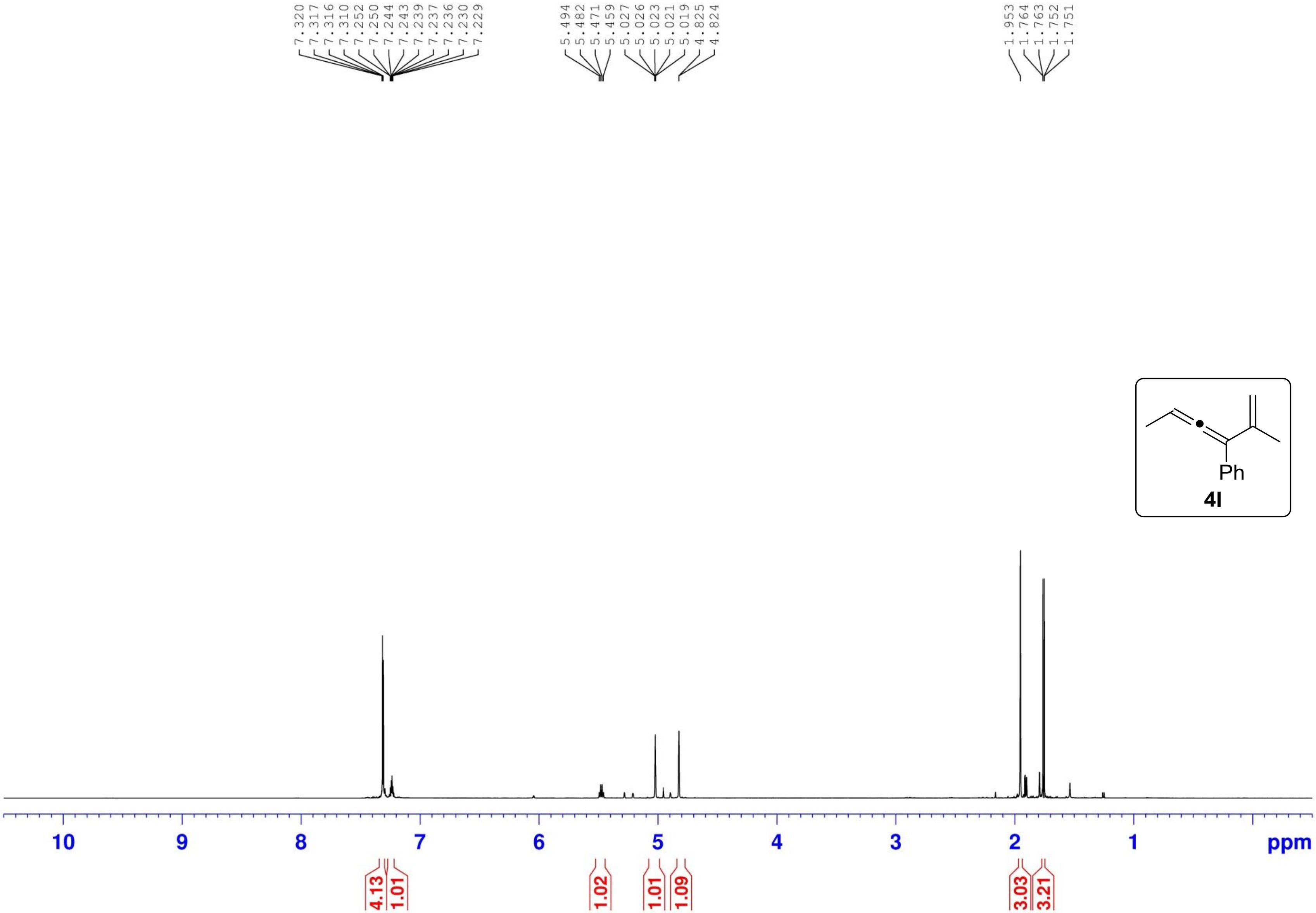

7.320
7.317
7.316
7.310
7.252
7.250
7.244
7.243
7.239
7.237
7.236
7.230
7.229
5.494
5.482
5.471
5.459
5.027
5.026
5.023
5.021
5.019
4.825
4.824
1.953
1.764
1.763
1.752
1.751
Ph
4l
10
9
8
7
6
5
4
3
2
1
ppm
4.13
1.01
1.02
1.01
1.09
3.03
3.21

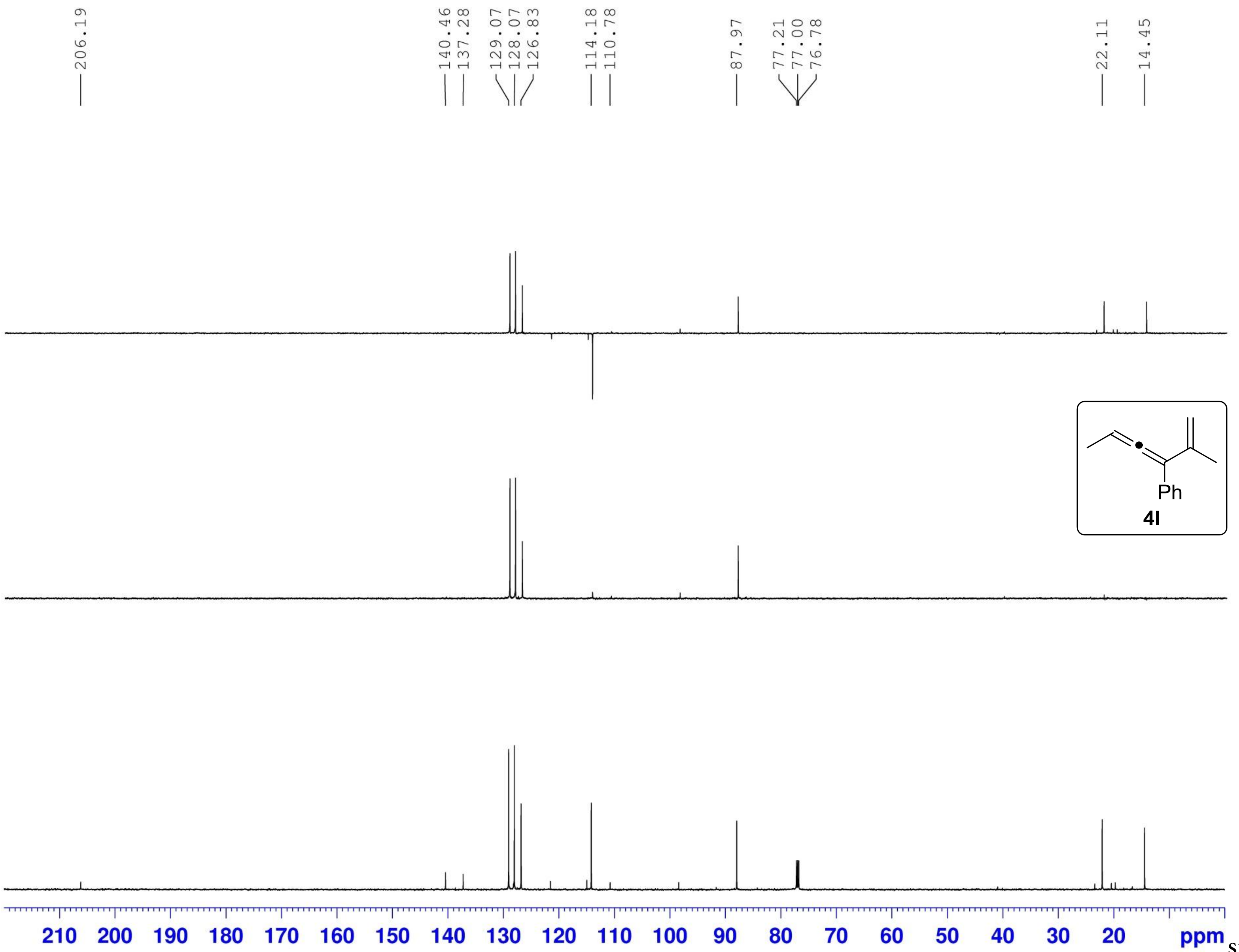
206.19
140.46
137.28
129.07
128.07
126.83
114.18
110.78
87.97
77.21
77.00
76.78
22.11
14.45
Ph
4l
210 200 190 180 170 160 150 140 130 120 110 100 90 80 70 60 50 40 30 20 ppm

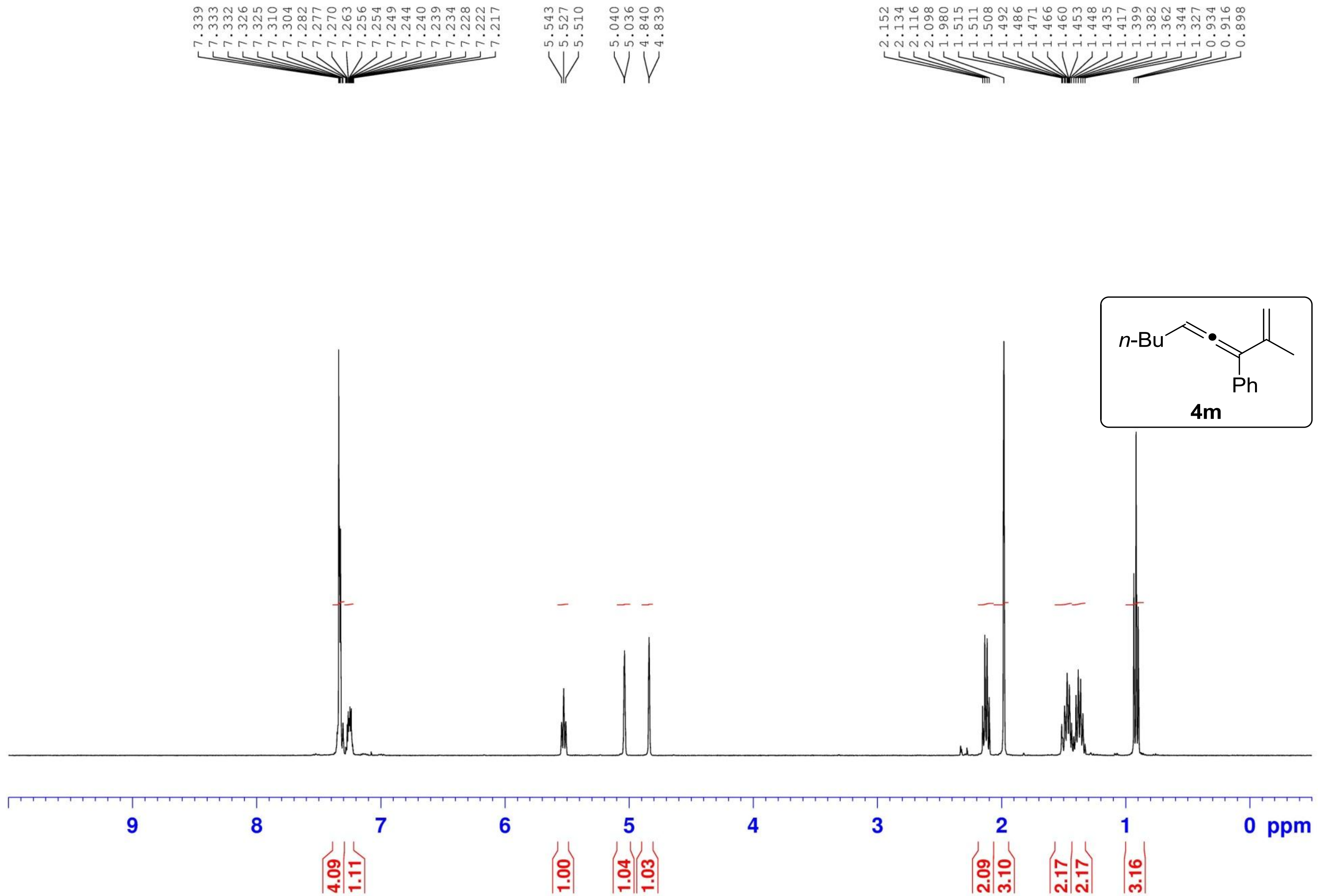
7.339
7.333
7.332
7.326
7.325
7.310
7.304
7.282
7.277
7.270
7.263
7.256
7.254
7.249
7.244
7.240
7.239
7.234
7.228
7.222
7.217
5.543
5.527
5.510
5.040
5.036
4.840
4.839
2.152
2.134
2.116
2.098
1.980
1.515
1.511
1.508
1.492
1.486
1.471
1.466
1.460
1.453
1.448
1.435
1.417
1.399
1.382
1.362
1.344
1.327
0.934
0.916
0.898
n-Bu
Ph
4m
9
8
7
6
5
4
3
2
1
0 ppm
4.09
1.11
1.00
1.04
1.03
2.09
3.10
2.17
2.17
3.16

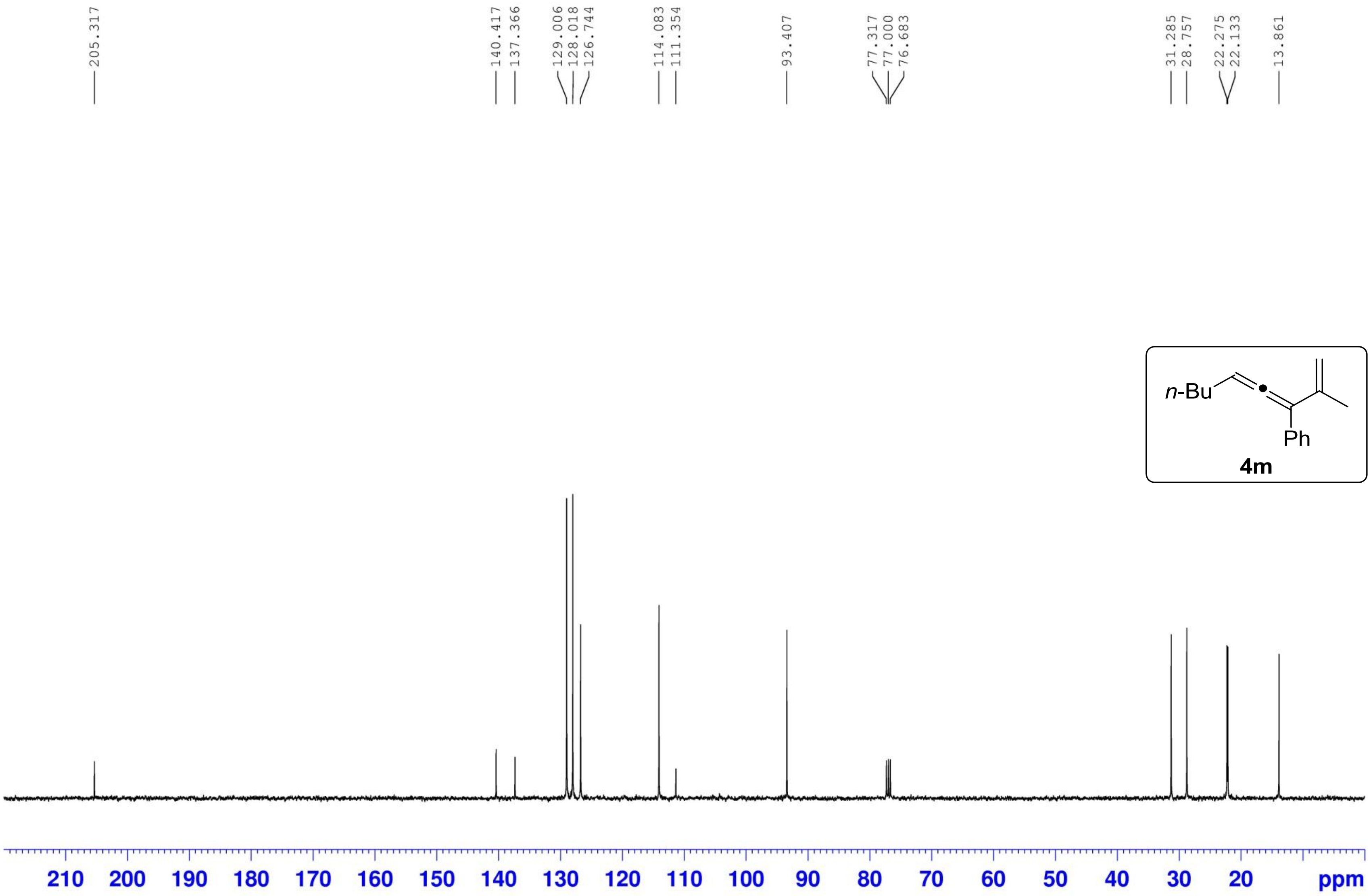
205.317
140.417
137.366
129.006
128.018
126.744
114.083
111.354
93.407
77.317
77.000
76.683
31.285
28.757
22.275
22.133
13.861
n-Bu
Ph
4m
210
200
190
180
170
160
150
140
130
120
110
100
90
80
70
60
50
40
30
20
ppm

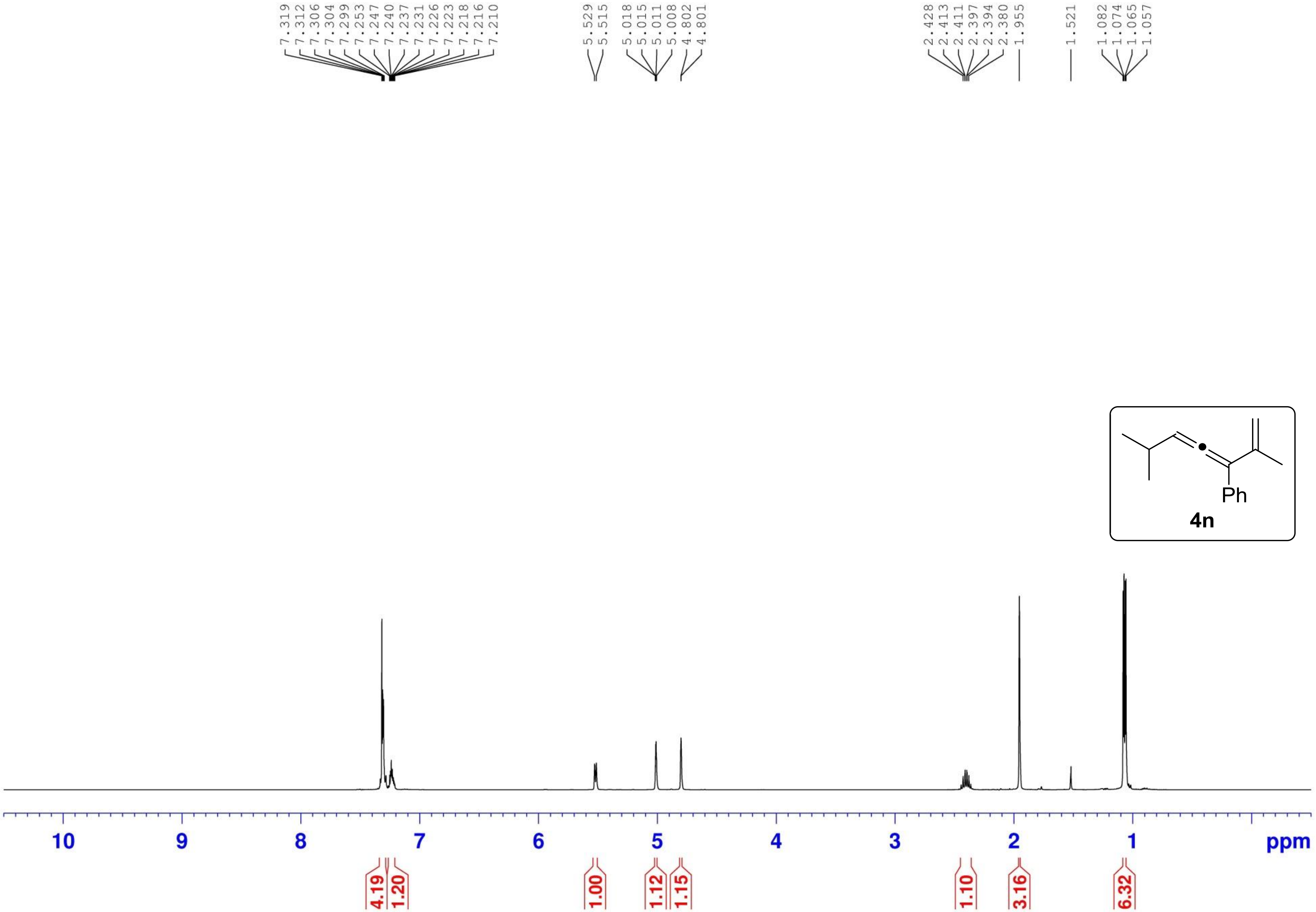
7.319
7.312
7.306
7.304
7.299
7.253
7.247
7.240
7.237
7.231
7.226
7.223
7.218
7.216
7.210
5.529
5.515
5.018
5.015
5.011
5.008
4.802
4.801
2.428
2.413
2.411
2.397
2.394
2.380
1.955
1.521
1.082
1.074
1.065
1.057
Ph
4n
10
9
8
7
6
5
4
3
2
1
ppm
4.19
1.20
1.00
1.12
1.15
1.10
3.16
6.32

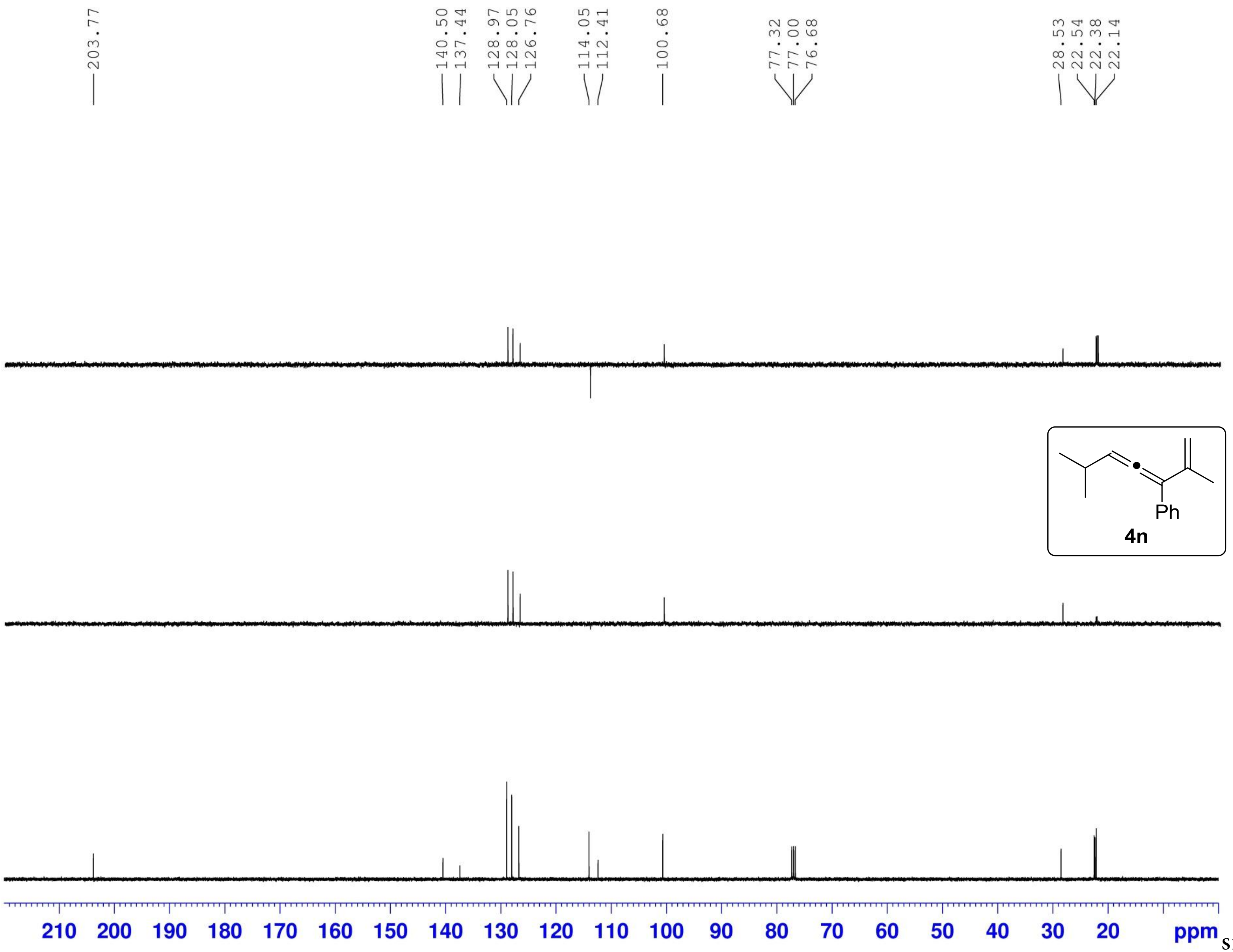

203.77
140.50
137.44
128.97
128.05
126.76
114.05
112.41
100.68
77.32
77.00
76.68
28.53
22.54
22.38
22.14
Ph
4n
210 200 190 180 170 160 150 140 130 120 110 100 90 80 70 60 50 40 30 20 ppm

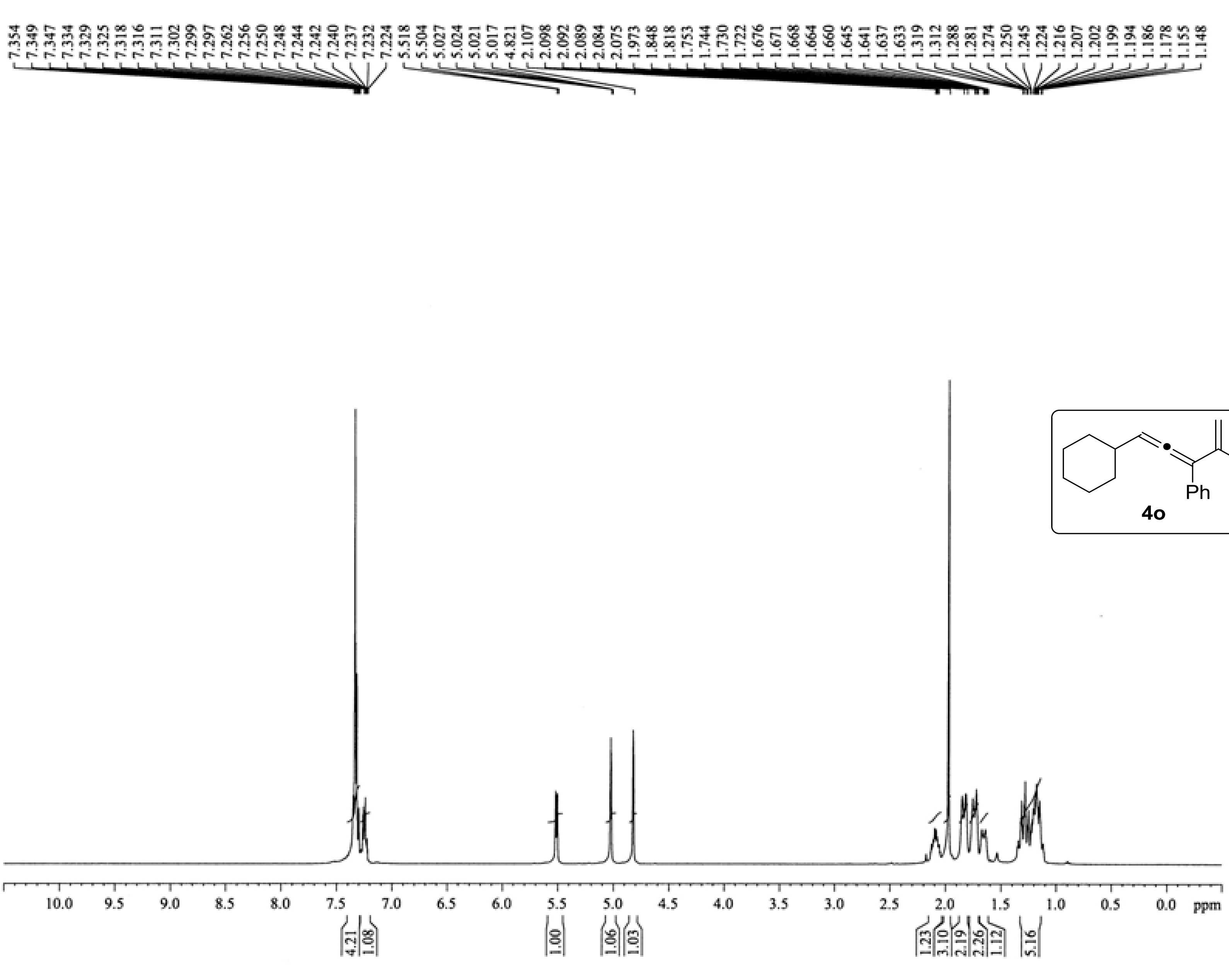
7.354 7.349 7.347 7.334 7.329 7.325 7.318 7.316 7.311 7.302 7.299 7.297 7.262 7.256 7.250 7.248 7.244 7.242 7.240 7.237 7.232 7.224
5.518 5.504 5.027 5.024 5.021 5.017 4.821 2.107 2.098 2.092 2.089 2.084 2.075 1.973 1.848 1.818 1.753 1.744 1.730 1.722 1.676 1.671 1.668 1.664 1.660 1.645 1.641 1.637 1.633 1.319 1.312 1.288 1.281 1.274 1.250 1.245 1.224 1.216 1.207 1.202 1.199 1.194 1.186 1.178 1.155 1.148
Ph
4o
10.0 9.5 9.0 8.5 8.0 7.5 7.0 6.5 6.0 5.5 5.0 4.5 4.0 3.5 3.0 2.5 2.0 1.5 1.0 0.5 0.0 ppm
4.21 1.08 1.00 1.06 1.03 1.23 3.10 2.19 2.26 1.12 5.16

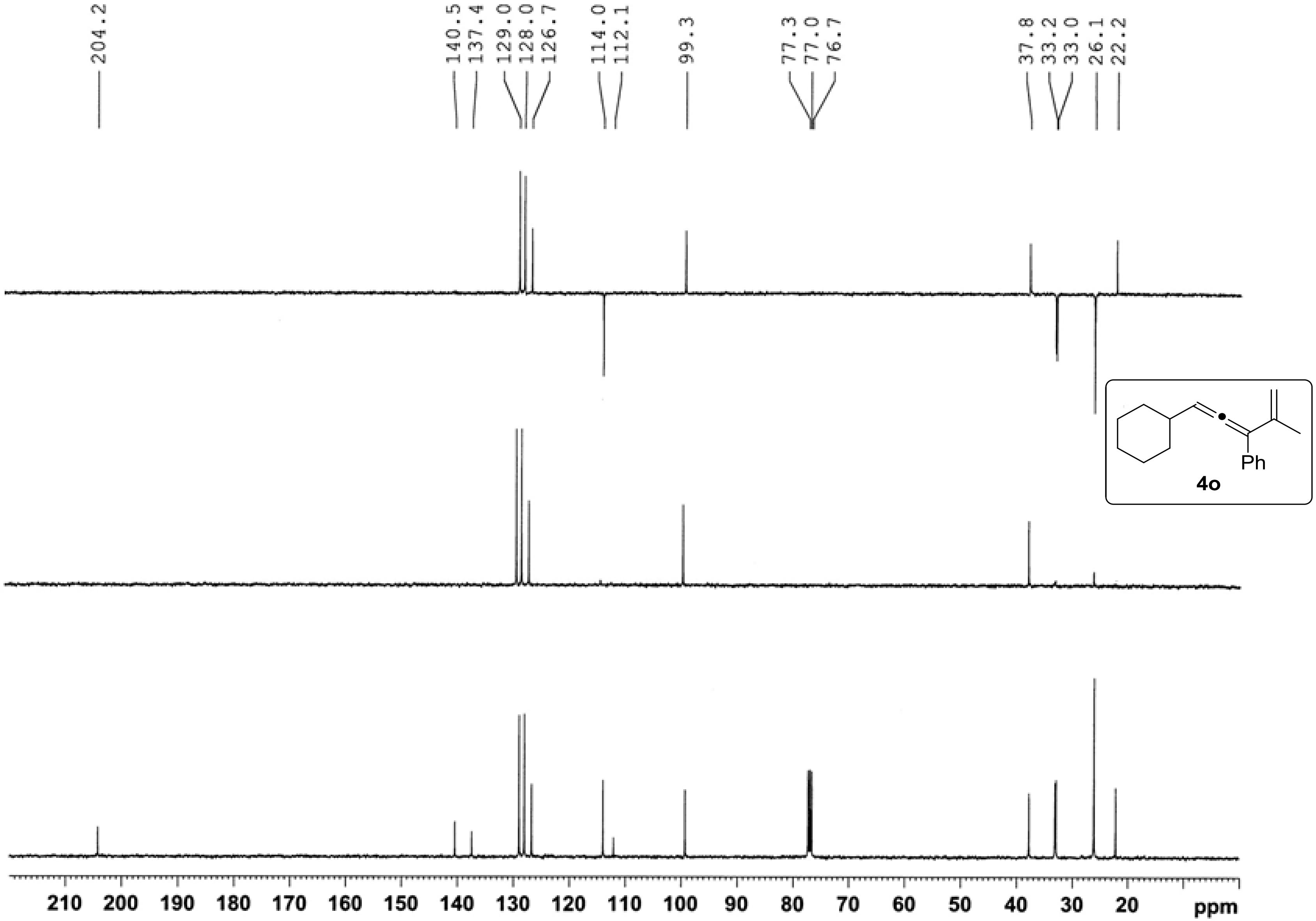
204.2
140.5
137.4
129.0
128.0
126.7
114.0
112.1
99.3
77.3
77.0
76.7
37.8
33.2
33.0
26.1
22.2
Ph
4o
210 200 190 180 170 160 150 140 130 120 110 100 90 80 70 60 50 40 30 20
ppm

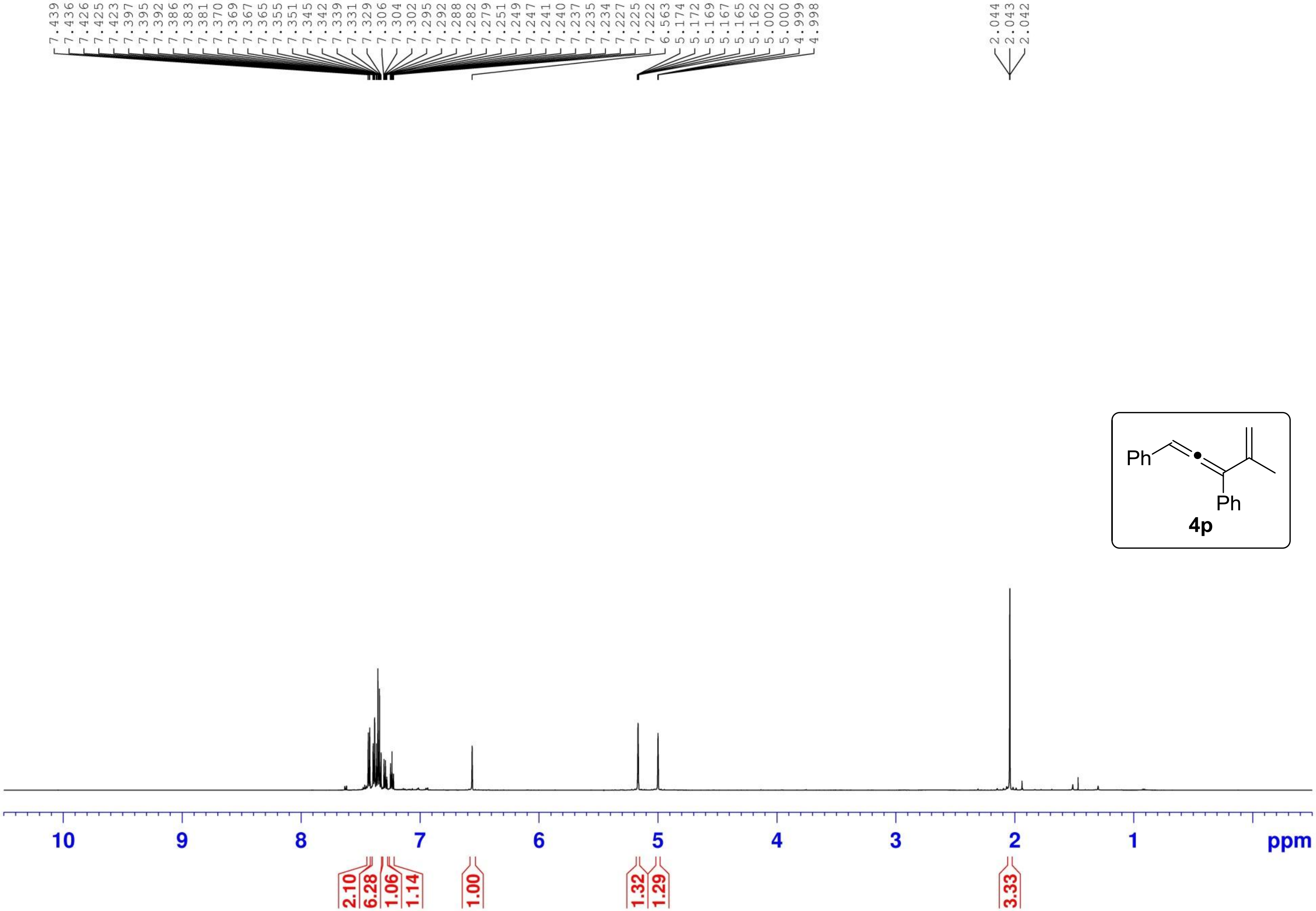
7.439 7.436 7.426 7.425 7.423 7.397 7.395 7.392 7.386 7.383 7.381 7.370 7.369 7.367 7.365 7.355 7.351 7.345 7.342 7.339 7.331 7.329 7.306 7.304 7.302 7.295 7.292 7.288 7.282 7.279 7.251 7.249 7.247 7.241 7.240 7.237 7.235 7.234 7.227 7.225 7.222 6.563 5.174 5.172 5.169 5.167 5.165 5.162 5.002 5.000 4.999 4.998
2.044 2.043 2.042
Ph
Ph
4p
10 9 8 7 6 5 4 3 2 1 ppm
2.10 6.28 1.06 1.14 1.00 1.32 1.29 3.33

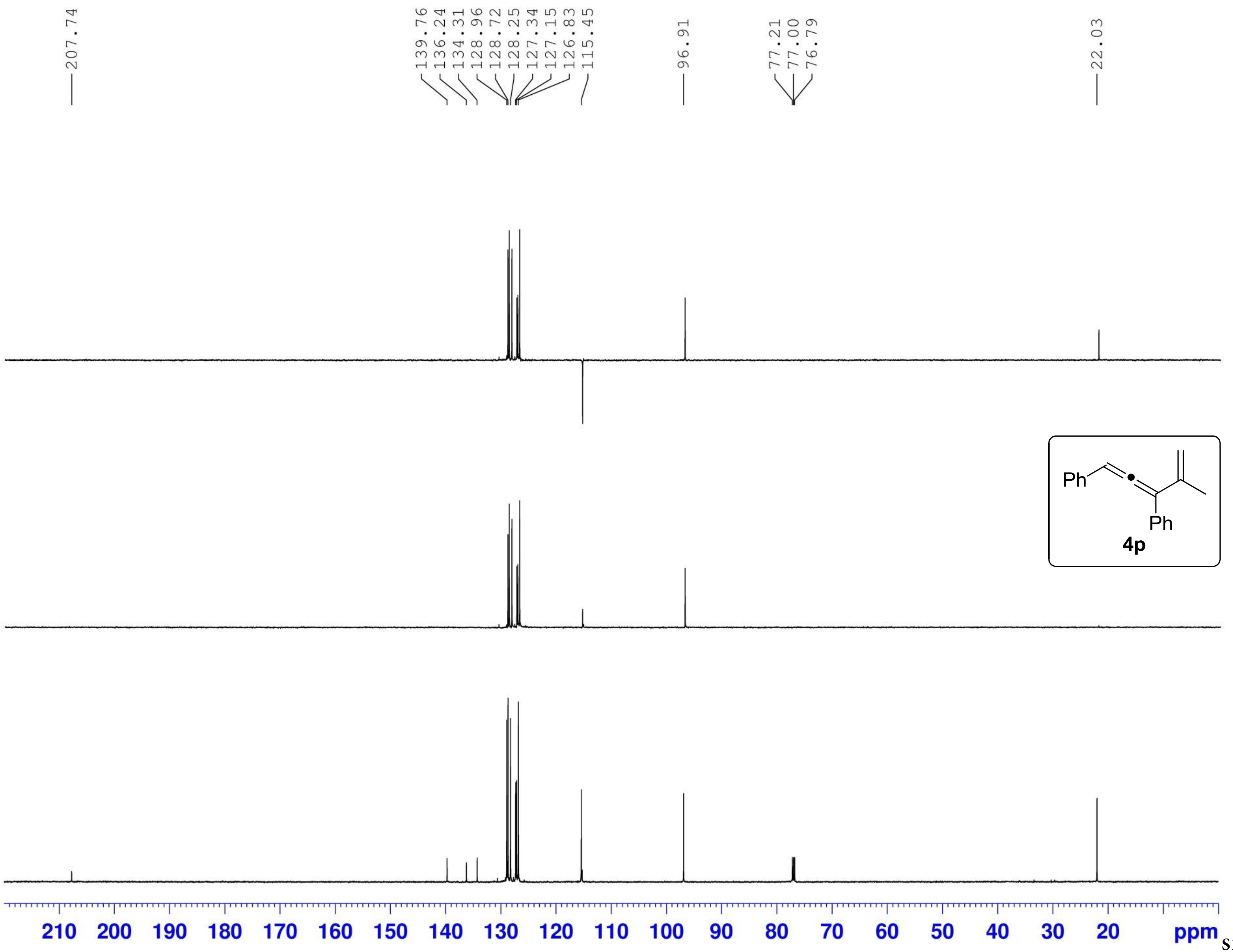
207.74
139.76
136.24
134.31
128.96
128.72
128.25
127.34
127.15
126.83
115.45
96.91
77.21
77.00
76.79
22.03
Ph
Ph
4p
210 200 190 180 170 160 150 140 130 120 110 100 90 80 70 60 50 40 30 20 ppm

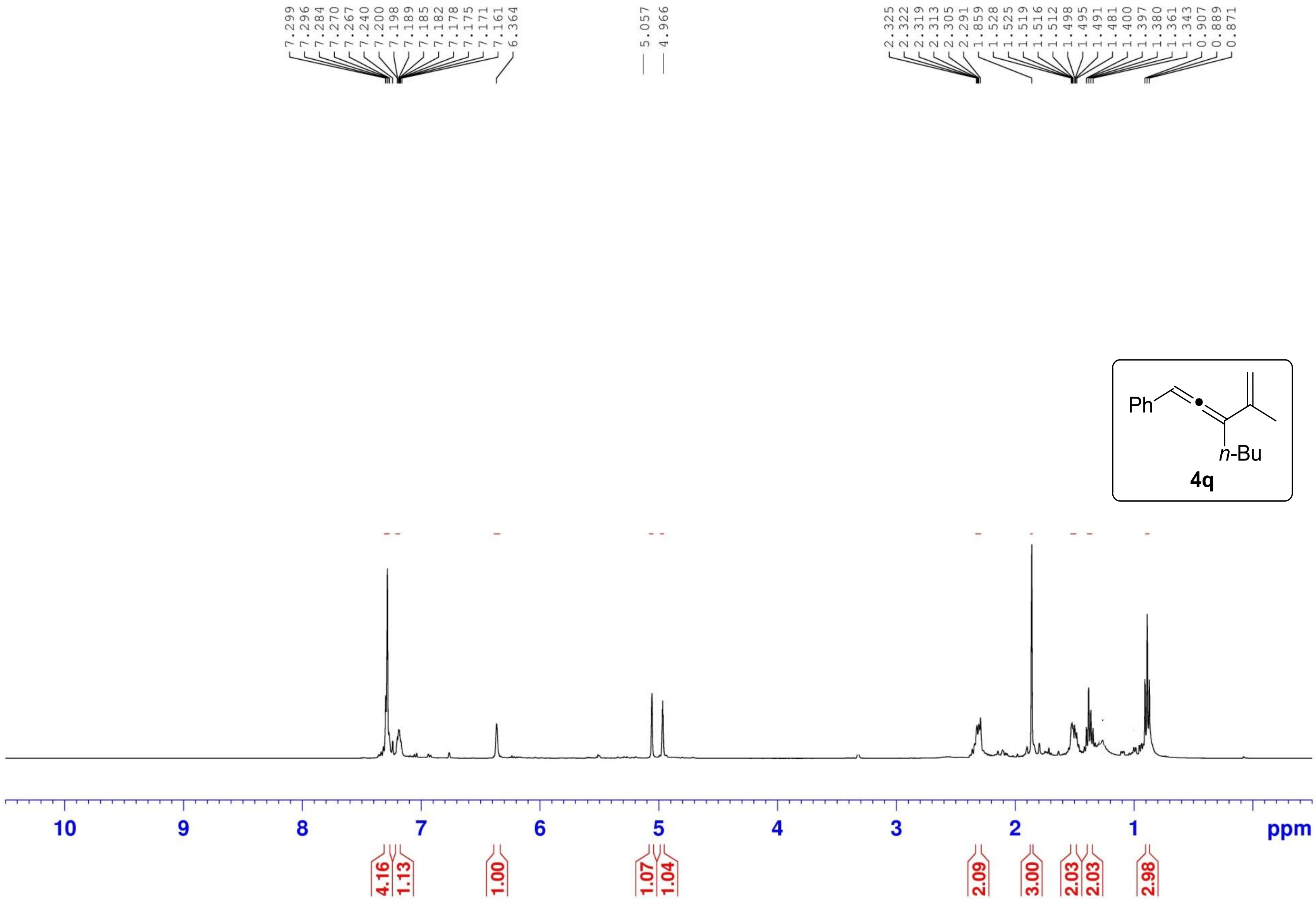

7.299
7.296
7.284
7.270
7.267
7.240
7.200
7.198
7.189
7.185
7.182
7.178
7.175
7.171
7.161
6.364
5.057
4.966
2.325
2.322
2.319
2.313
2.305
2.291
1.859
1.528
1.525
1.519
1.516
1.512
1.498
1.495
1.491
1.481
1.400
1.397
1.380
1.361
1.343
0.907
0.889
0.871
Ph
n-Bu
4q
10
9
8
7
6
5
4
3
2
1
ppm
4.16
1.13
1.00
1.07
1.04
2.09
3.00
2.03
2.03
2.98

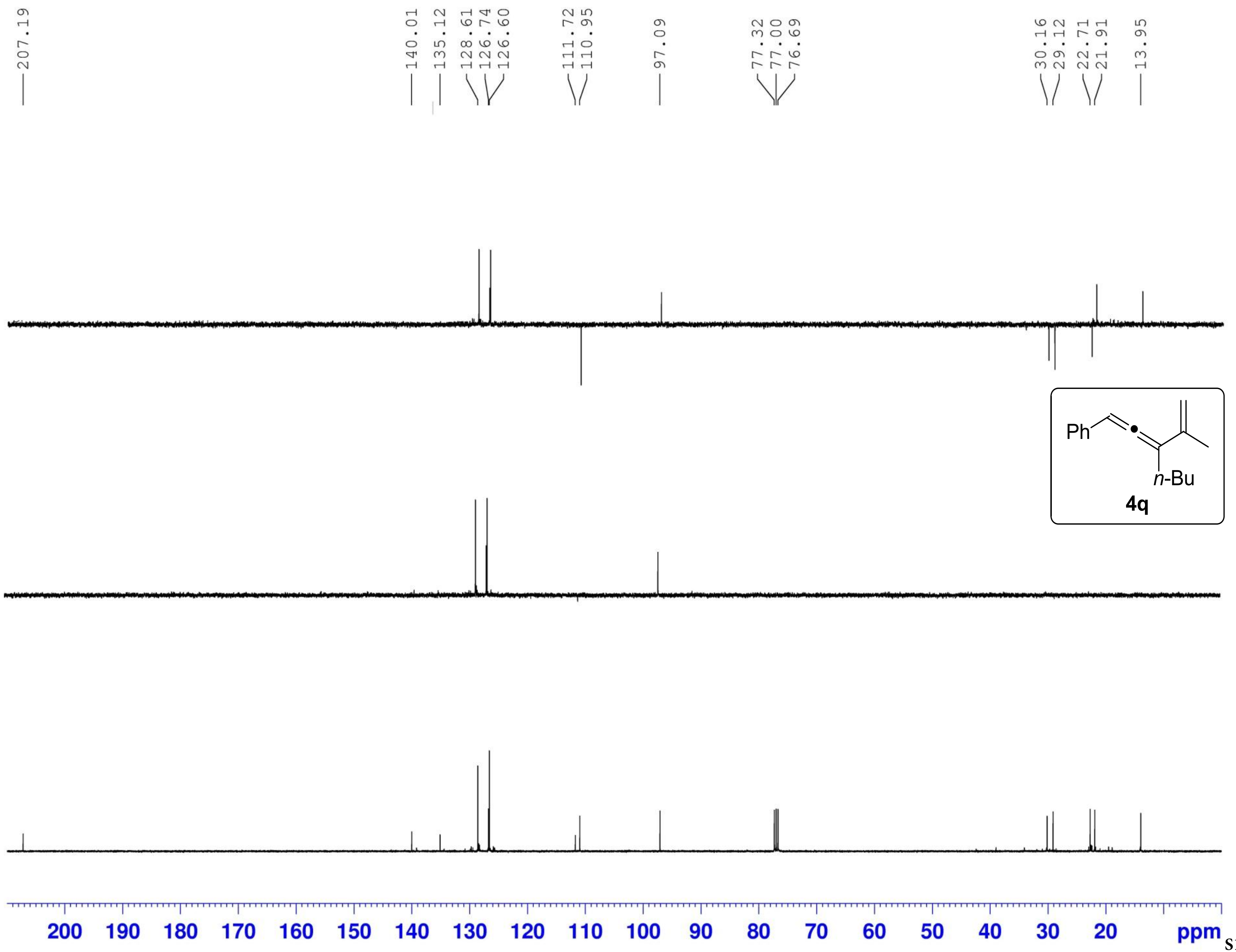
207.19
140.01
135.12
128.61
126.74
126.60
111.72
110.95
97.09
77.32
77.00
76.69
30.16
29.12
22.71
21.91
13.95
Ph
n-Bu
4q
200 190 180 170 160 150 140 130 120 110 100 90 80 70 60 50 40 30 20 ppm

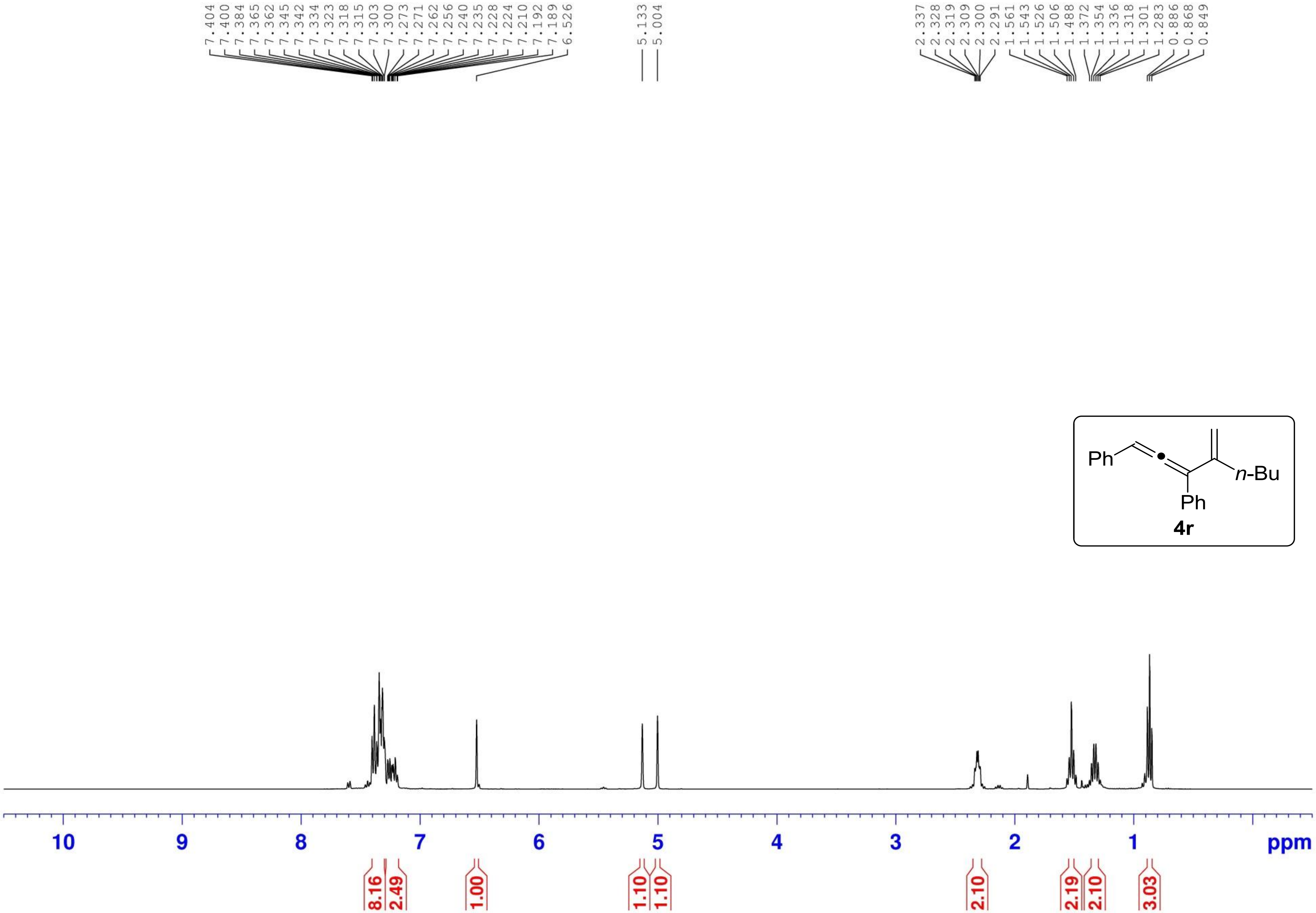
7.404
7.400
7.384
7.365
7.362
7.345
7.342
7.334
7.323
7.318
7.315
7.303
7.300
7.273
7.271
7.262
7.256
7.240
7.235
7.228
7.224
7.210
7.192
7.189
6.526
5.133
5.004
2.337
2.328
2.319
2.309
2.300
2.291
1.561
1.543
1.526
1.506
1.488
1.372
1.354
1.336
1.318
1.301
1.283
0.886
0.868
0.849
Ph
n-Bu
Ph
4r
10
9
8
7
6
5
4
3
2
1
ppm
8.16
2.49
1.00
1.10
1.10
2.10
2.19
2.10
3.03

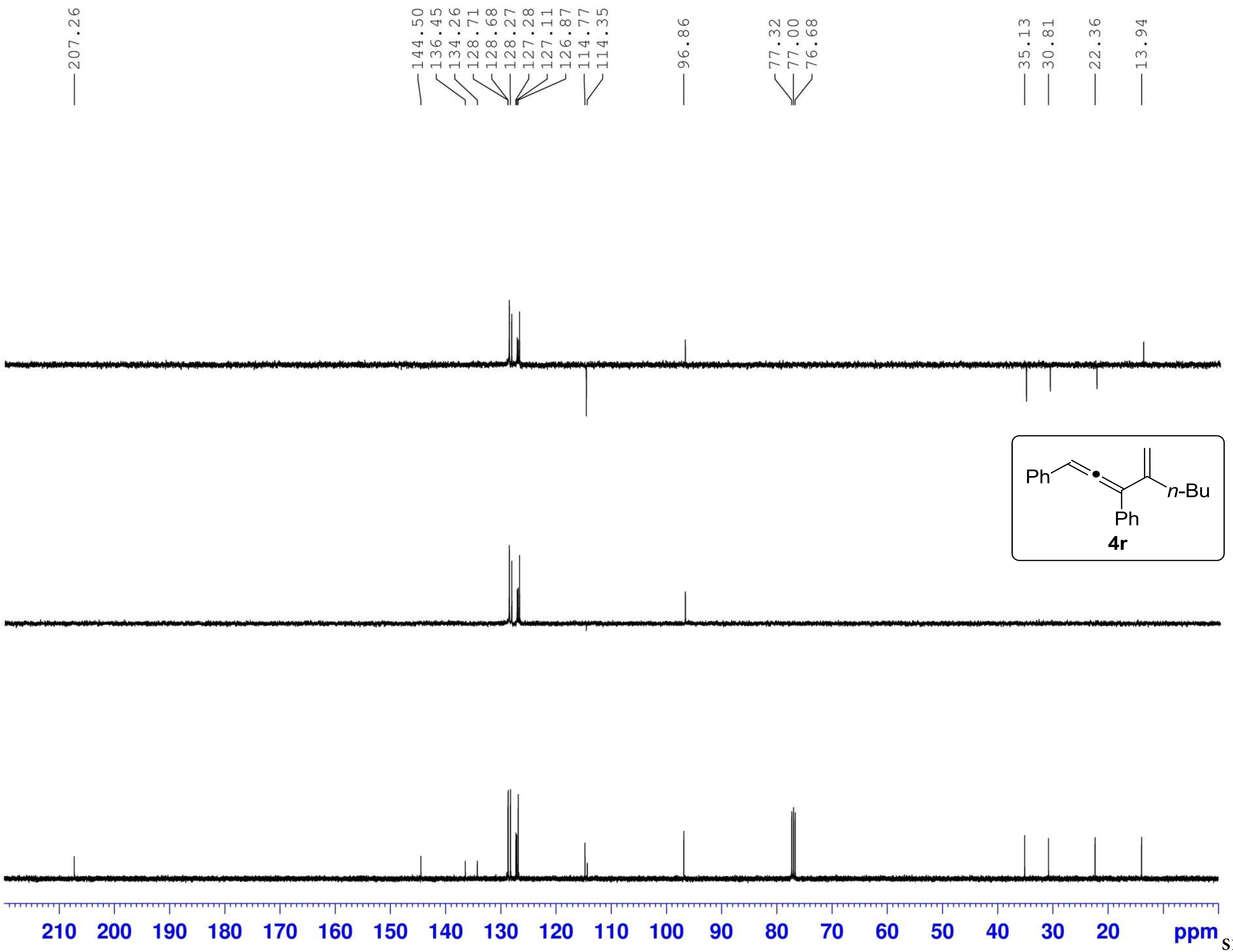
207.26
144.50
136.45
134.26
128.71
128.68
128.27
127.28
127.11
126.87
114.77
114.35
96.86
77.32
77.00
76.68
35.13
30.81
22.36
13.94
210 200 190 180 170 160 150 140 130 120 110 100 90 80 70 60 50 40 30 20 ppm
Ph
n-Bu
Ph

4r

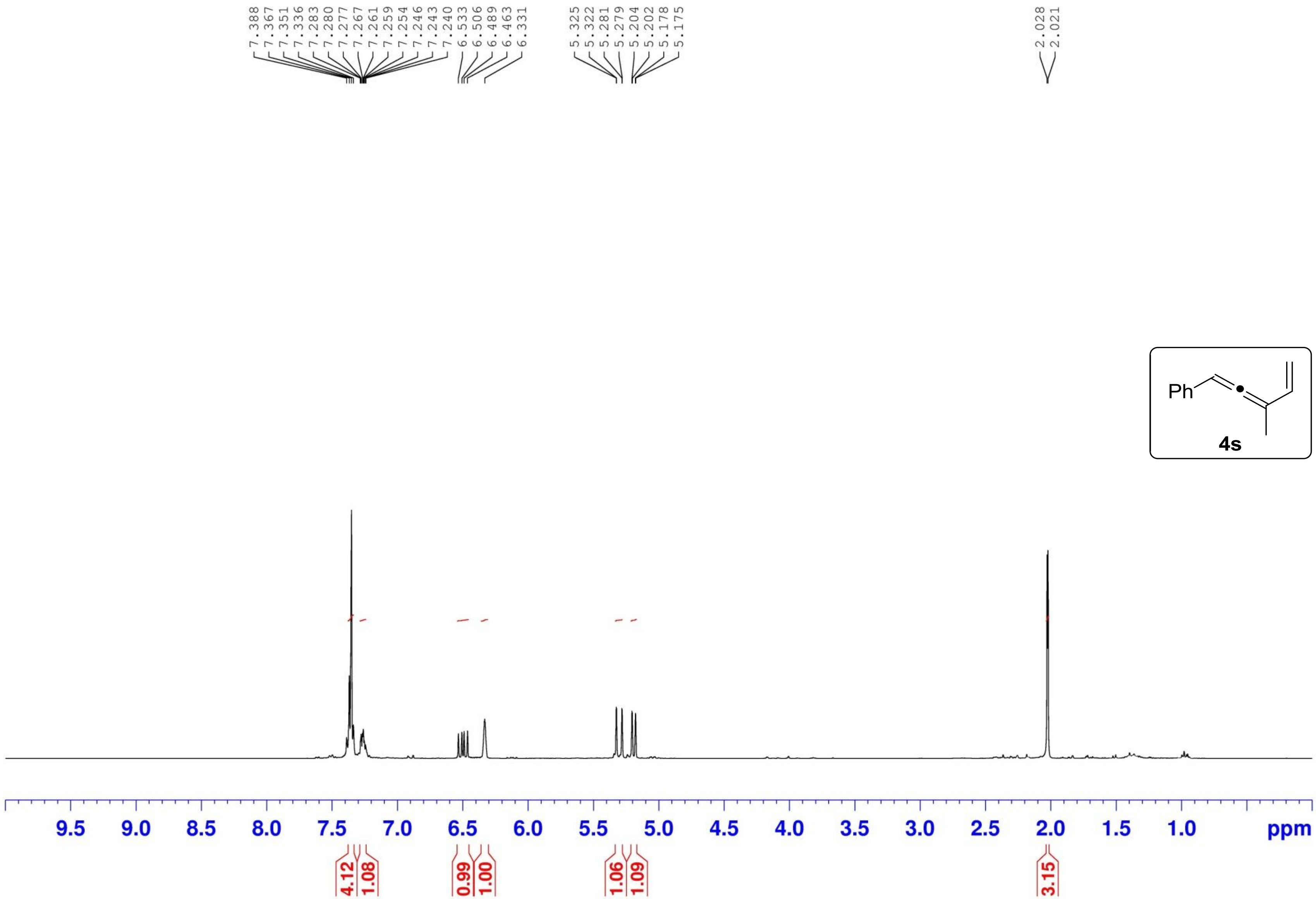
7.388
7.367
7.351
7.336
7.283
7.280
7.277
7.267
7.261
7.259
7.254
7.246
7.243
7.240
6.533
6.506
6.489
6.463
6.331
5.325
5.322
5.281
5.279
5.204
5.202
5.178
5.175
2.028
2.021
Ph
4s
9.5
9.0
8.5
8.0
7.5
7.0
6.5
6.0
5.5
5.0
4.5
4.0
3.5
3.0
2.5
2.0
1.5
1.0
ppm
4.12
1.08
0.99
1.00
1.06
1.09
3.15

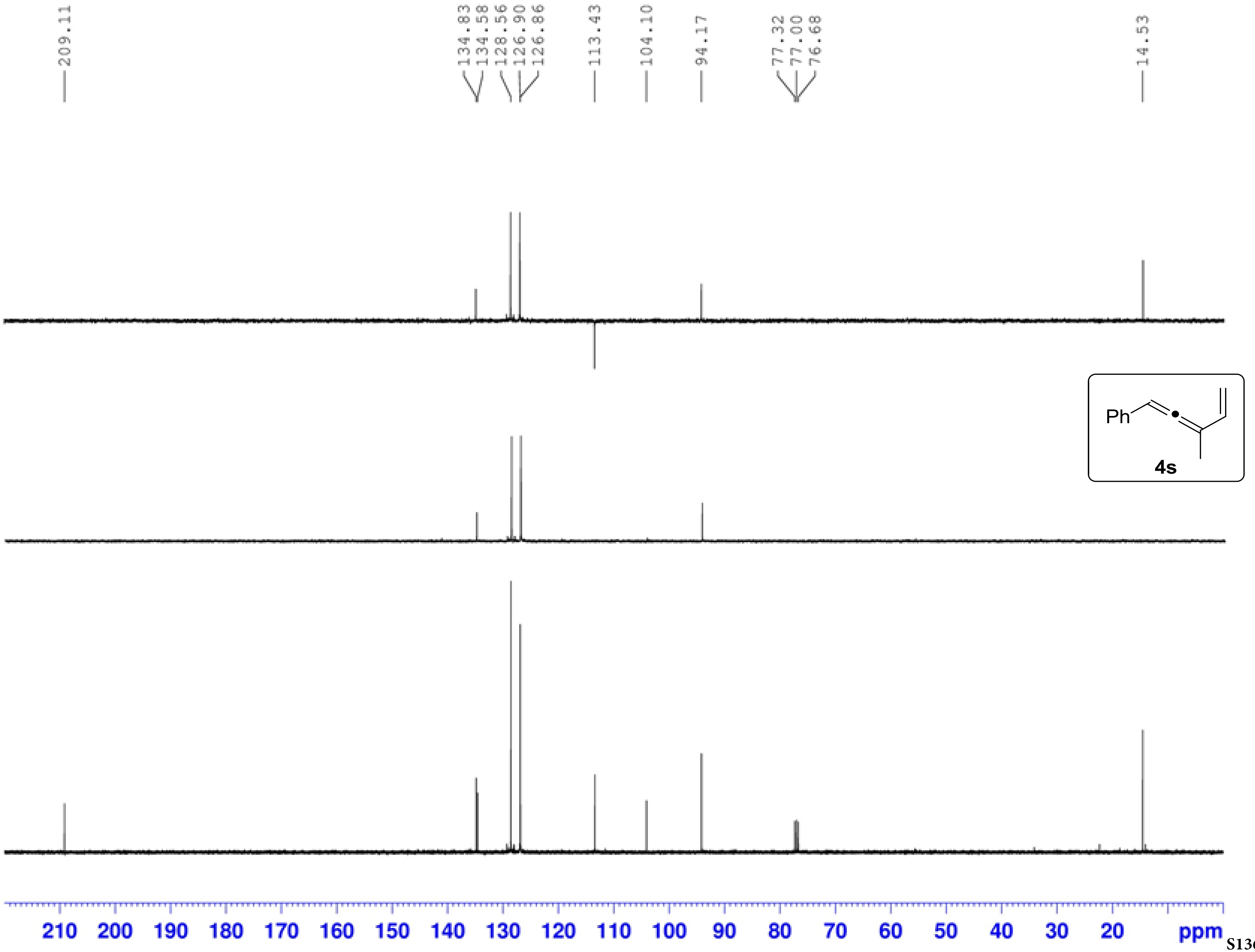
209.11
134.83
134.58
128.56
126.90
126.86
113.43
104.10
94.17
77.32
77.00
76.68
14.53
Ph
4s
210 200 190 180 170 160 150 140 130 120 110 100 90 80 70 60 50 40 30 20 ppm

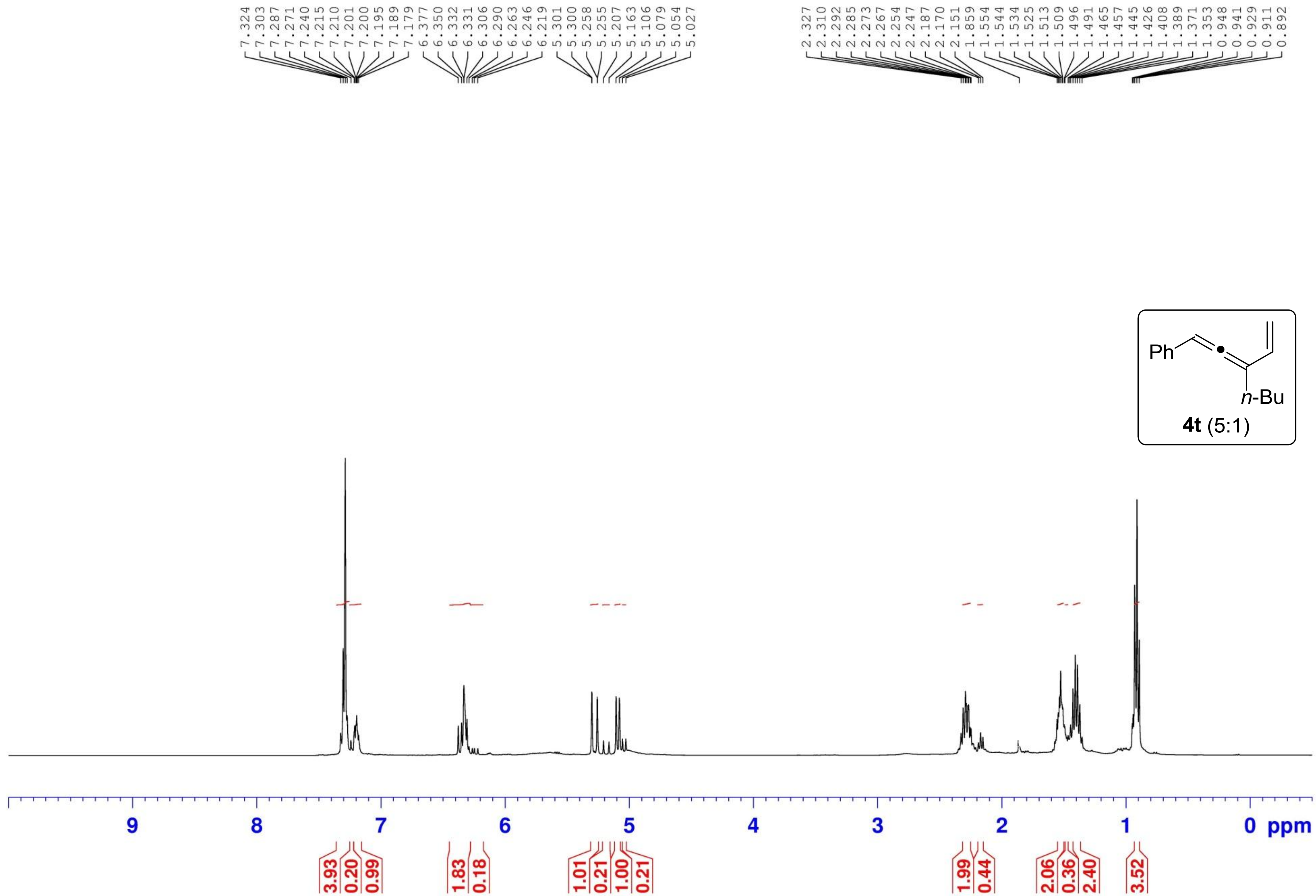
7.324 7.303 7.287 7.271 7.240 7.215 7.210 7.201 7.200 7.195 7.189 7.179 6.377 6.350 6.332 6.331 6.306 6.290 6.263 6.246 6.219 5.301 5.300 5.258 5.255 5.207 5.163 5.106 5.079 5.054 5.027
2.327 2.310 2.292 2.285 2.273 2.267 2.254 2.247 2.187 2.170 2.151 1.859 1.554 1.544 1.534 1.525 1.513 1.509 1.496 1.491 1.465 1.457 1.445 1.426 1.408 1.389 1.371 1.353 0.948 0.941 0.929 0.911 0.892
Ph
n-Bu
4t (5:1)
9 8 7 6 5 4 3 2 1 0 ppm
3.93 0.20 0.99 1.83 0.18 1.01 0.21 1.00 0.21 1.99 0.44 2.06 0.36 2.40 3.52

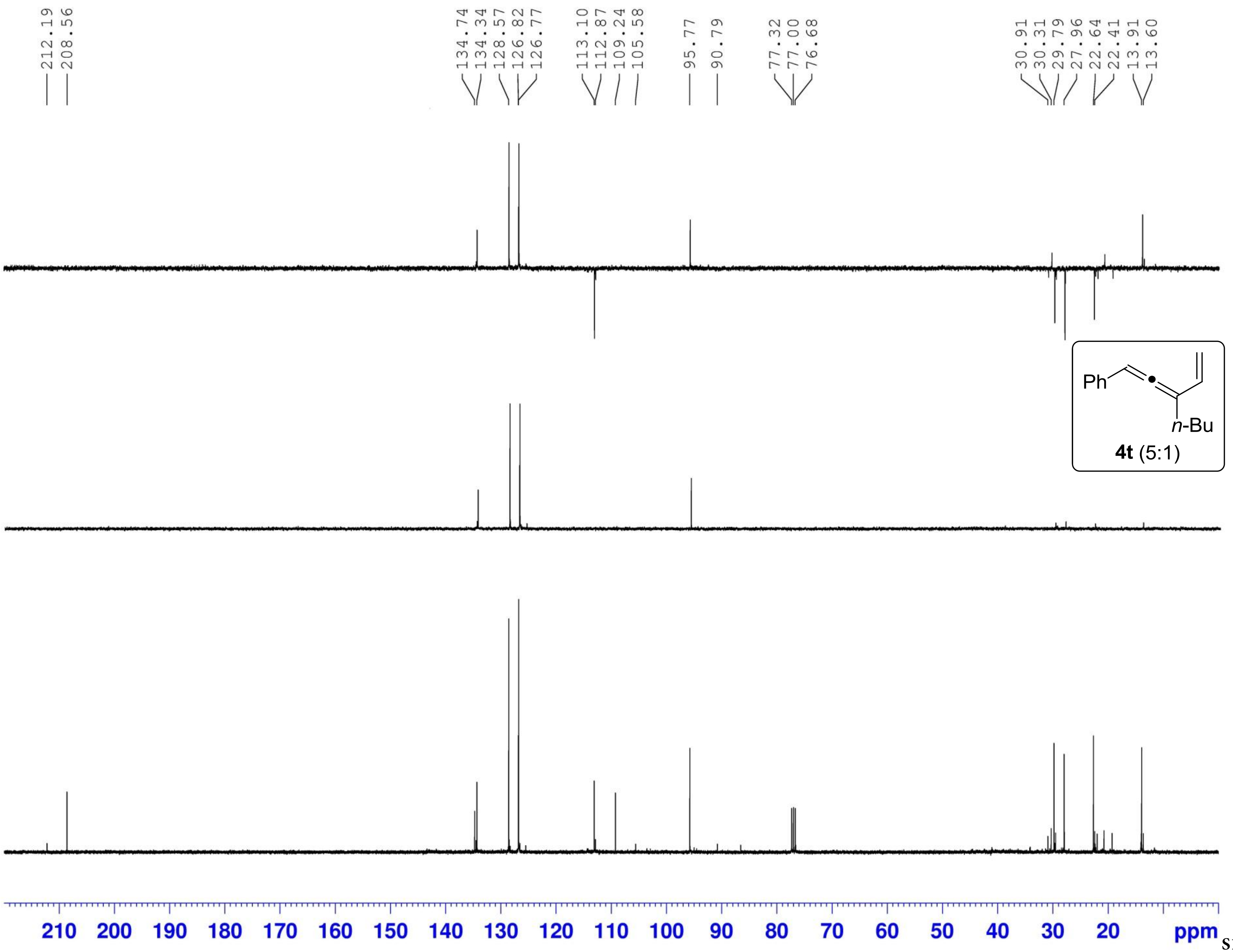

212.19
208.56
134.74
134.34
128.57
126.82
126.77
113.10
112.87
109.24
105.58
95.77
90.79
77.32
77.00
76.68
30.91
30.31
29.79
27.96
22.64
22.41
13.91
13.60
Ph
n-Bu
4t (5:1)
210 200 190 180 170 160 150 140 130 120 110 100 90 80 70 60 50 40 30 20 ppm

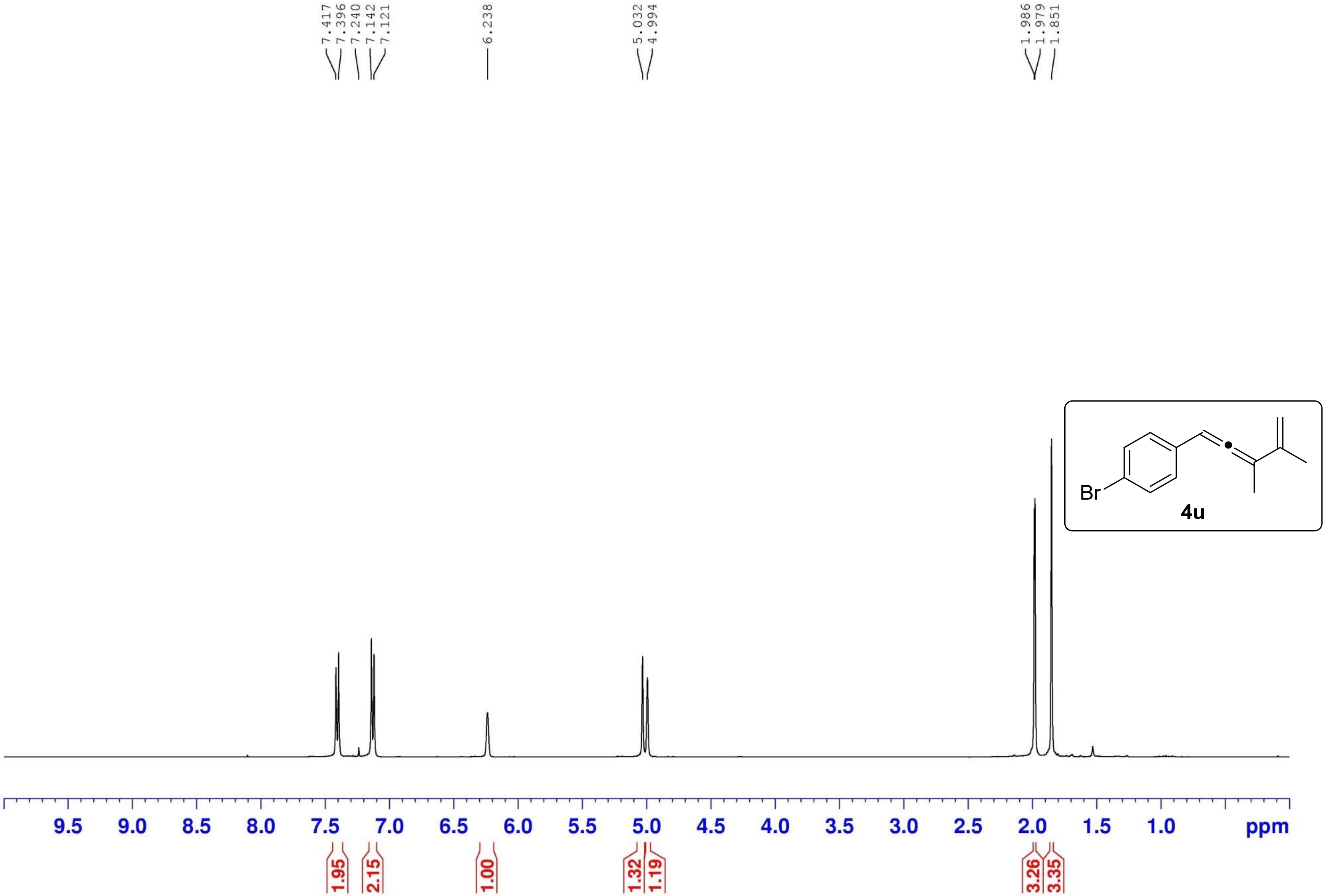
7.417
7.396
7.240
7.142
7.121
6.238
5.032
4.994
1.986
1.979
1.851
Br
4u
9.5 9.0 8.5 8.0 7.5 7.0 6.5 6.0 5.5 5.0 4.5 4.0 3.5 3.0 2.5 2.0 1.5 1.0 ppm
1.95
2.15
1.00
1.32
1.19
3.26
3.35

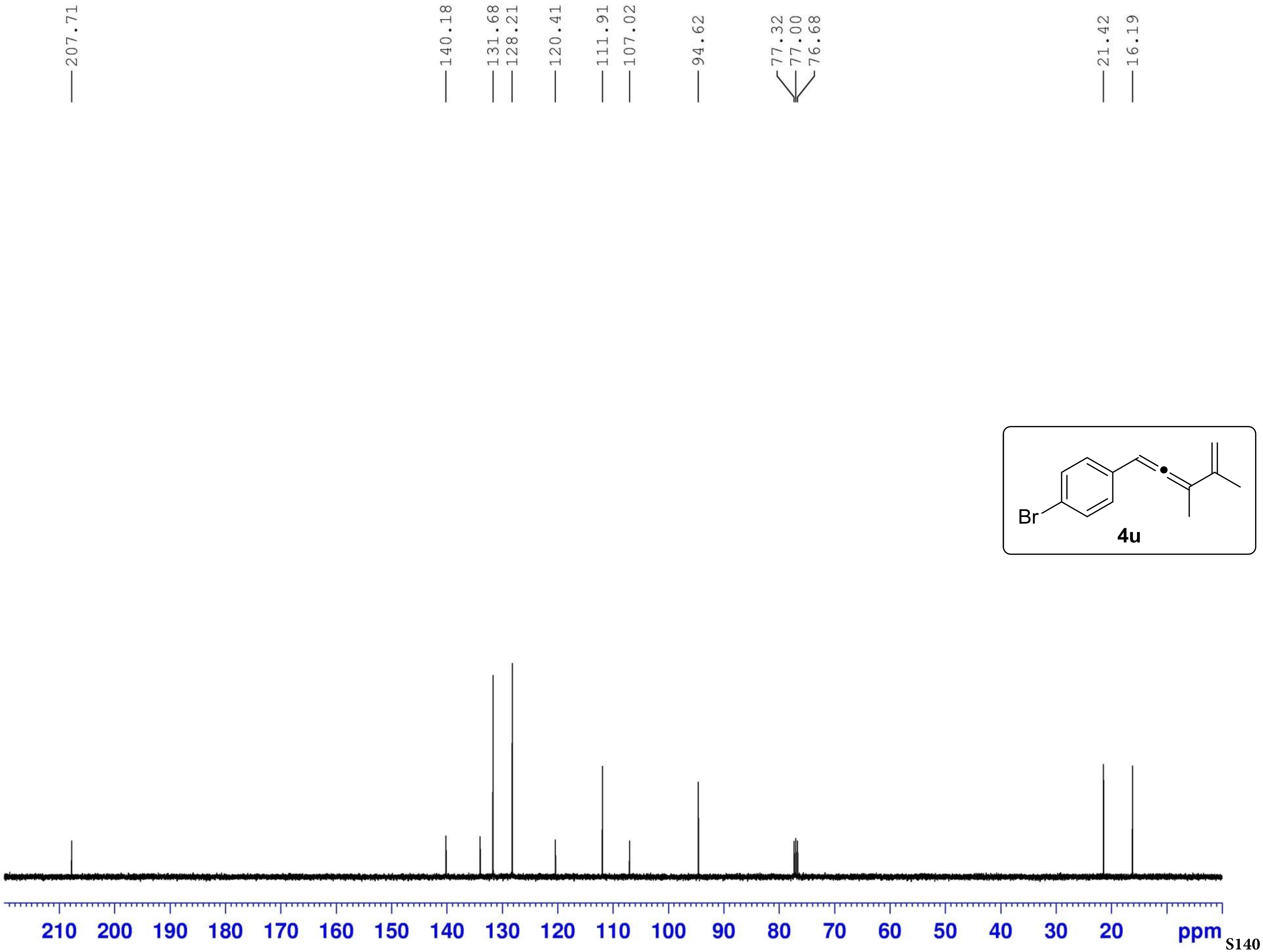
207.71
140.18
131.68
128.21
120.41
111.91
107.02
94.62
77.32
77.00
76.68
21.42
16.19
Br
4u
210 200 190 180 170 160 150 140 130 120 110 100 90 80 70 60 50 40 30 20
ppm

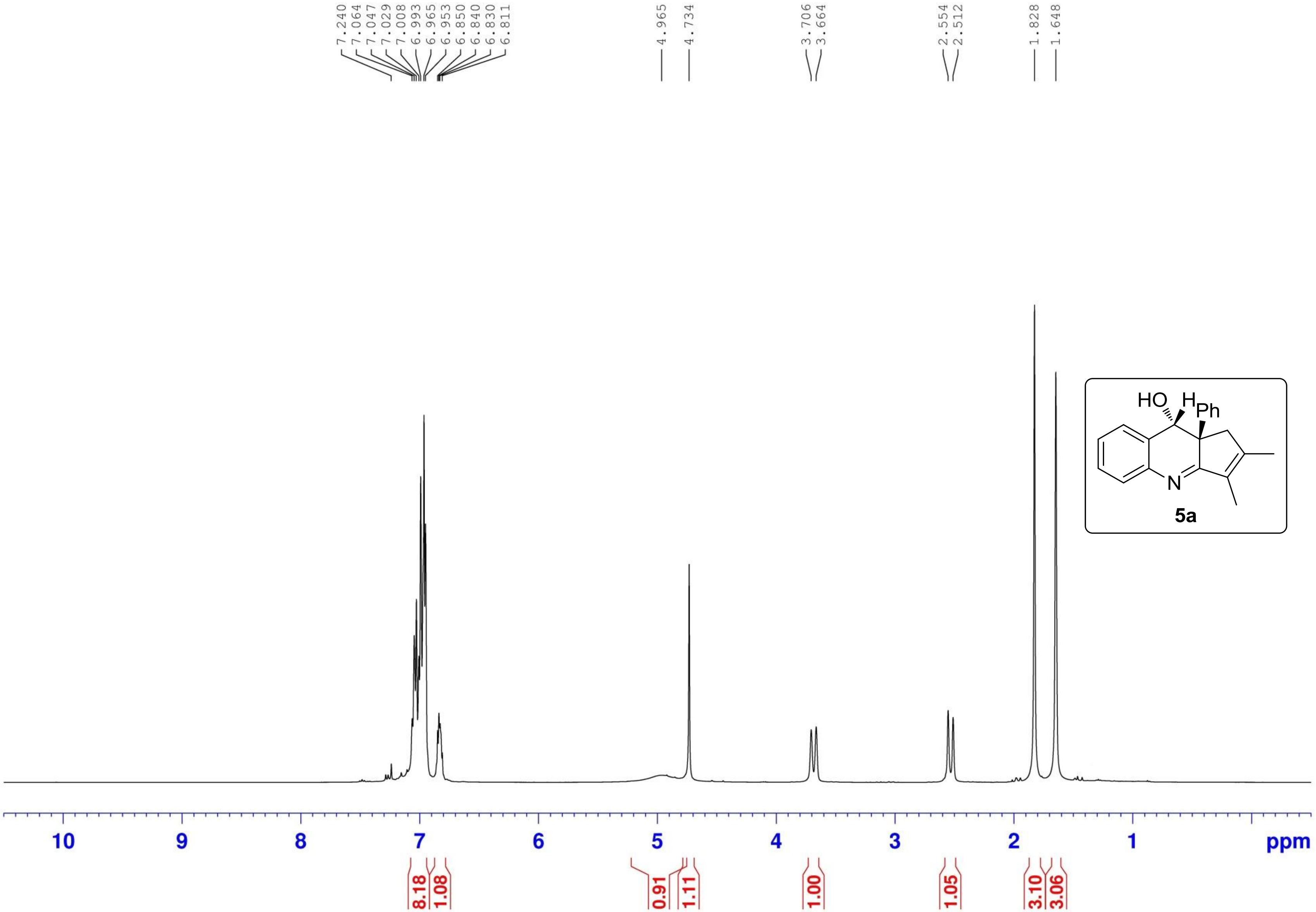

7.240
7.064
7.047
7.029
7.008
6.993
6.965
6.953
6.850
6.840
6.830
6.811
4.965
4.734
3.706
3.664
2.554
2.512
1.828
1.648
HO
H
Ph
N
5a
10
9
8
7
6
5
4
3
2
1
ppm
8.18
1.08
0.91
1.11
1.00
1.05
3.10
3.06

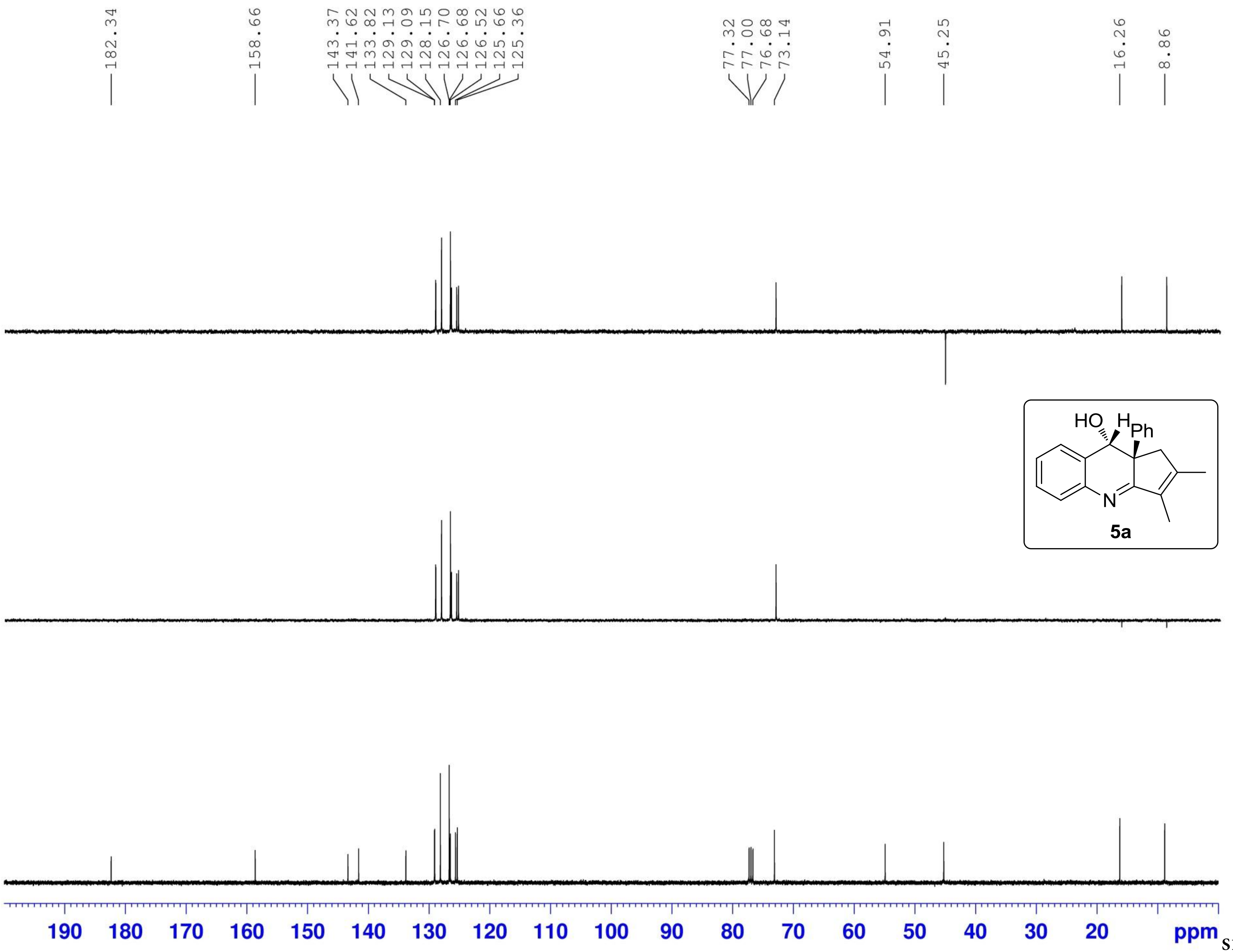
182.34
158.66
143.37
141.62
133.82
129.13
129.09
128.15
126.70
126.68
126.52
125.66
125.36
77.32
77.00
76.68
73.14
54.91
45.25
16.26
8.86
HO
H
Ph
N
5a
190
180
170
160
150
140
130
120
110
100
90
80
70
60
50
40
30
20
ppm

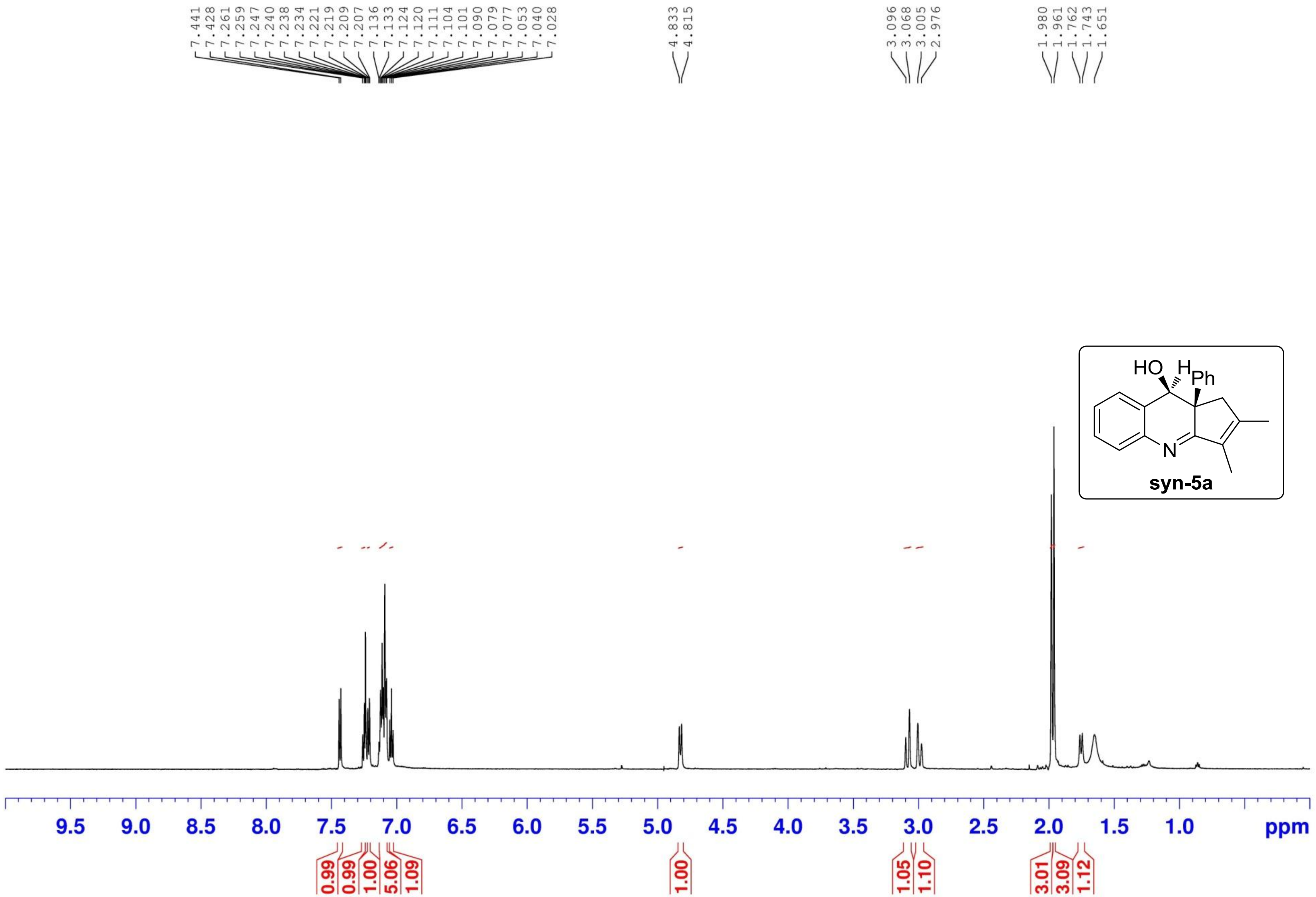
7.441
7.428
7.261
7.259
7.247
7.240
7.238
7.234
7.221
7.219
7.209
7.207
7.136
7.133
7.124
7.120
7.111
7.104
7.101
7.090
7.079
7.077
7.053
7.040
7.028
4.833
4.815
3.096
3.068
3.005
2.976
1.980
1.961
1.762
1.743
1.651
HO
H
Ph
N
syn-5a
9.5
9.0
8.5
8.0
7.5
7.0
6.5
6.0
5.5
5.0
4.5
4.0
3.5
3.0
2.5
2.0
1.5
1.0
ppm
0.99
0.99
1.00
5.06
1.09
1.00
1.05
1.10
3.01
3.09
1.12

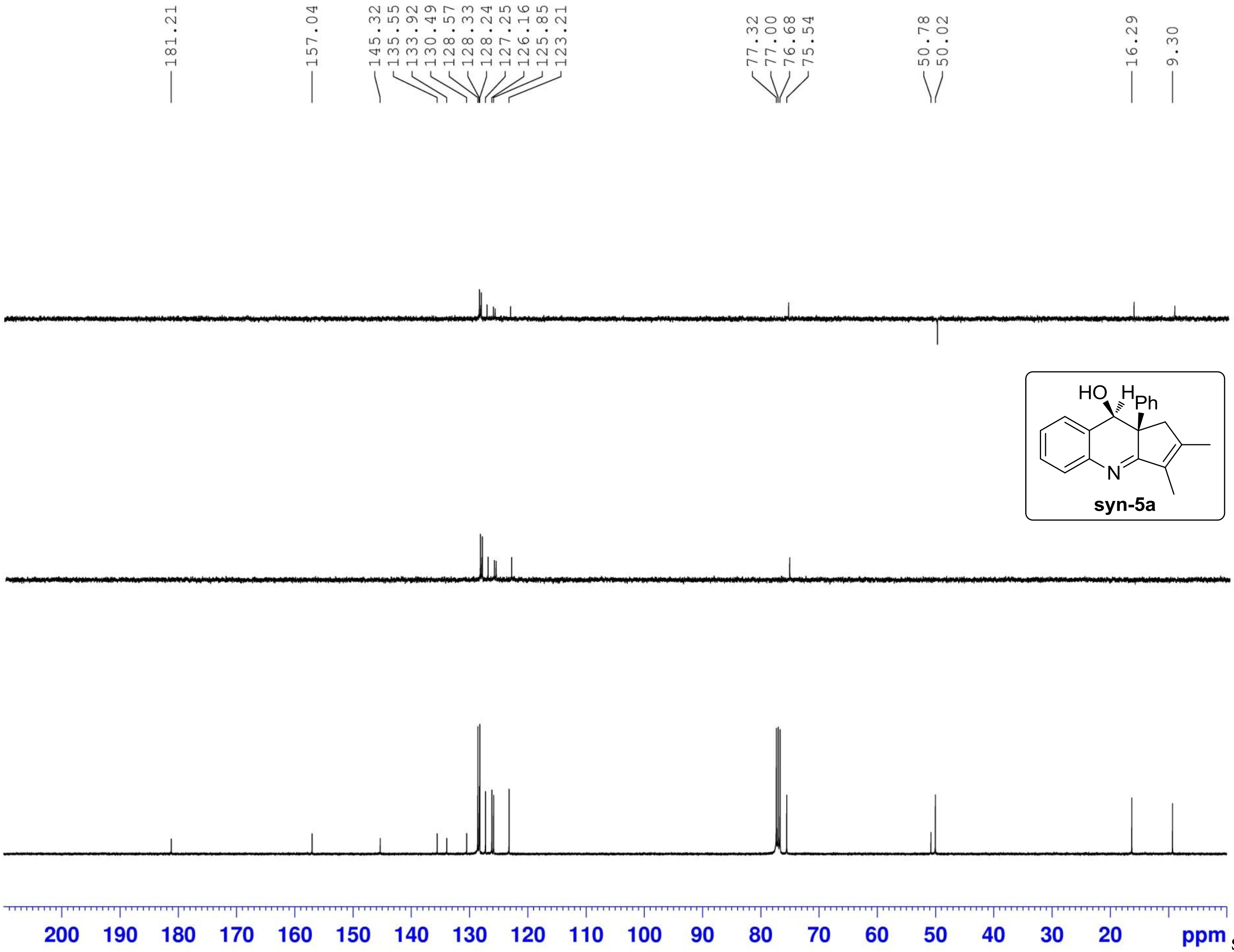
181.21
157.04
145.32
135.55
133.92
130.49
128.57
128.33
128.24
127.25
126.16
125.85
123.21
77.32
77.00
76.68
75.54
50.78
50.02
16.29
9.30
HO
H
Ph
N
syn-5a
200 190 180 170 160 150 140 130 120 110 100 90 80 70 60 50 40 30 20 ppm

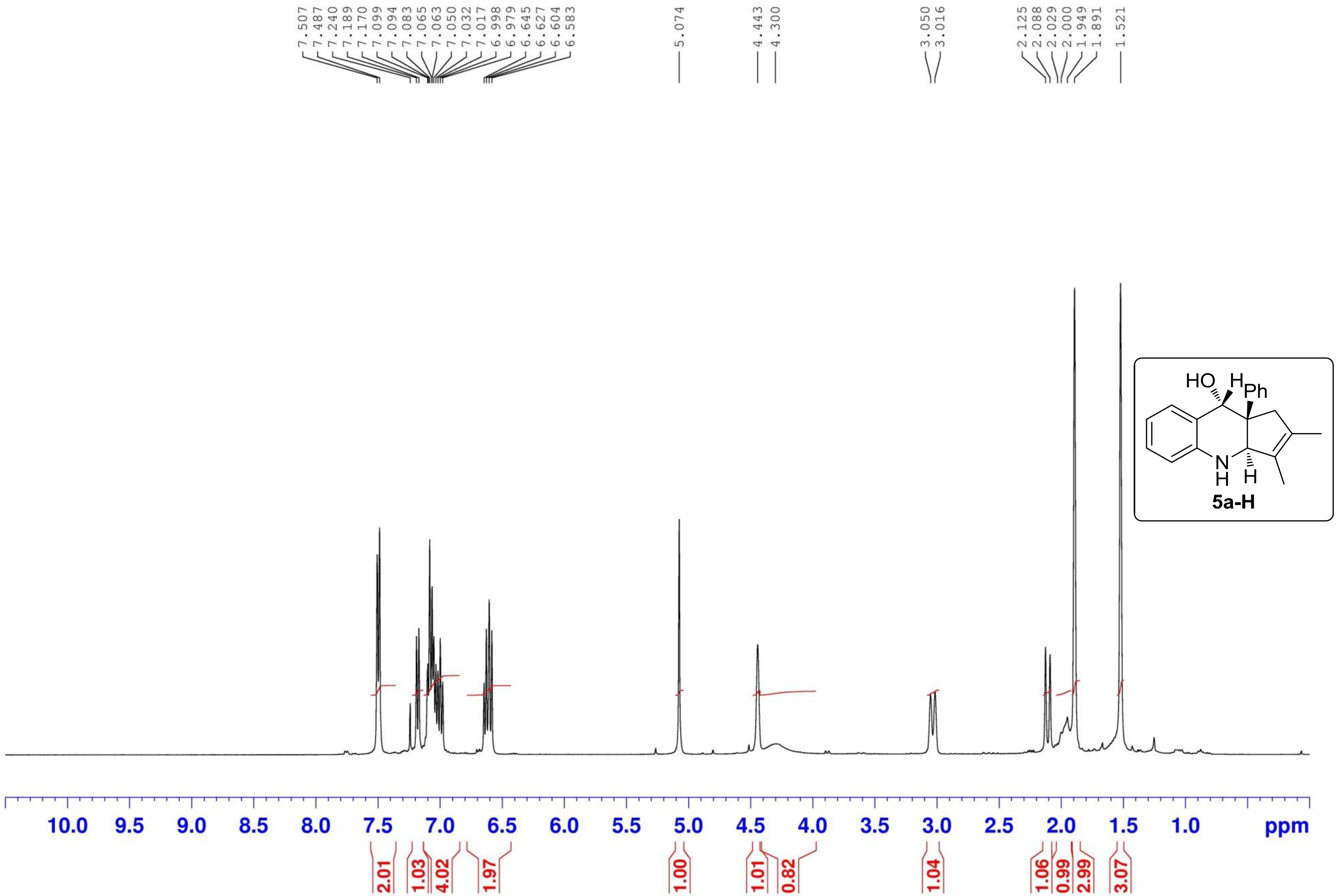

7.507
7.487
7.240
7.189
7.170
7.099
7.094
7.083
7.065
7.063
7.050
7.032
7.017
6.998
6.979
6.645
6.627
6.604
6.583
5.074
4.443
4.300
3.050
3.016
2.125
2.088
2.029
2.000
1.949
1.891
1.521
HO
H
Ph
N
H
H
5a-H
10.0 9.5 9.0 8.5 8.0 7.5 7.0 6.5 6.0 5.5 5.0 4.5 4.0 3.5 3.0 2.5 2.0 1.5 1.0 ppm
2.01
1.03
4.02
1.97
1.00
1.01
0.82
1.04
1.06
0.99
2.99
3.07

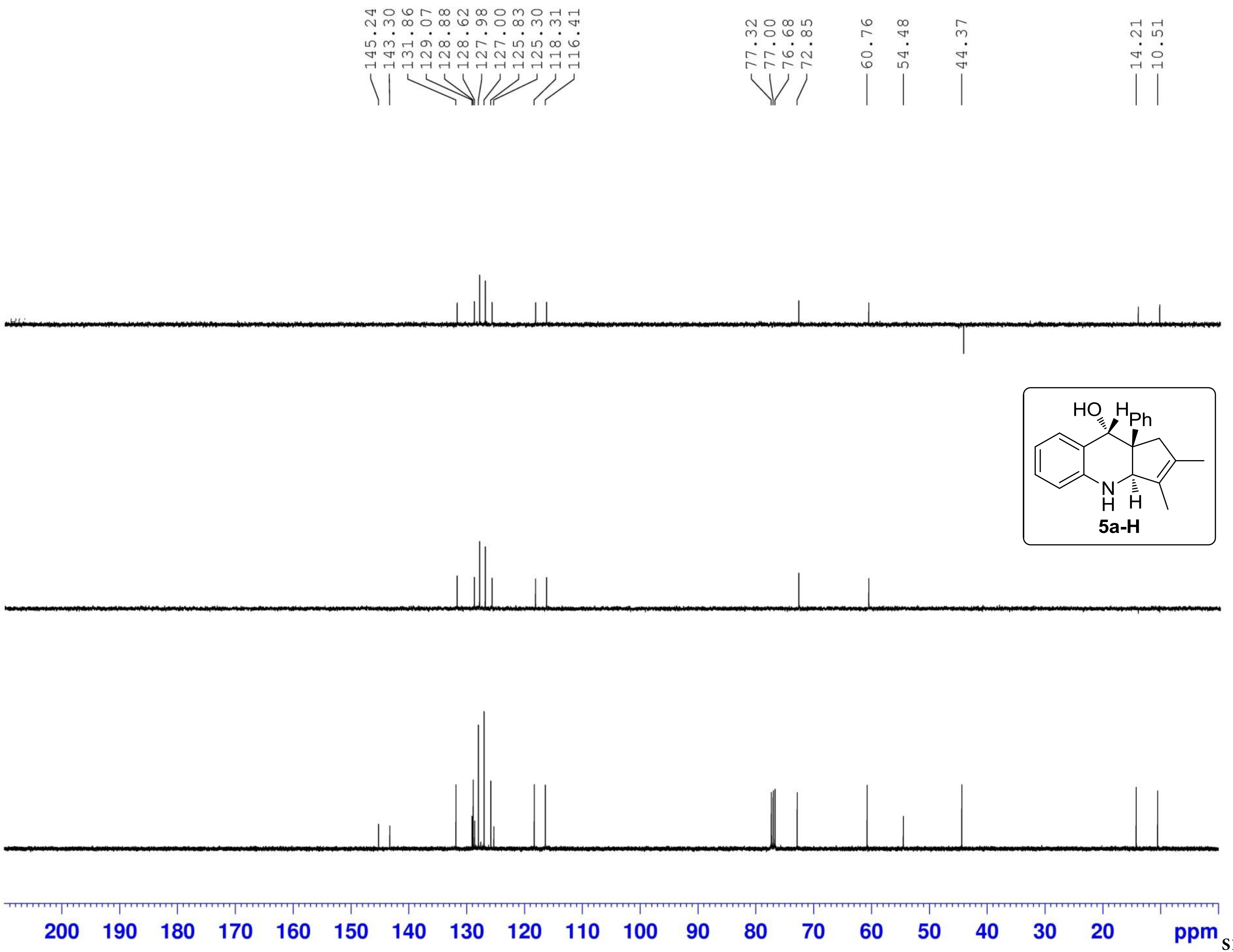
145.24
143.30
131.86
129.07
128.88
128.62
127.98
127.00
125.83
125.30
118.31
116.41
77.32
77.00
76.68
72.85
60.76
54.48
44.37
14.21
10.51
HO
H
Ph
N
H
H
5a-H
200
190
180
170
160
150
140
130
120
110
100
90
80
70
60
50
40
30
20
ppm

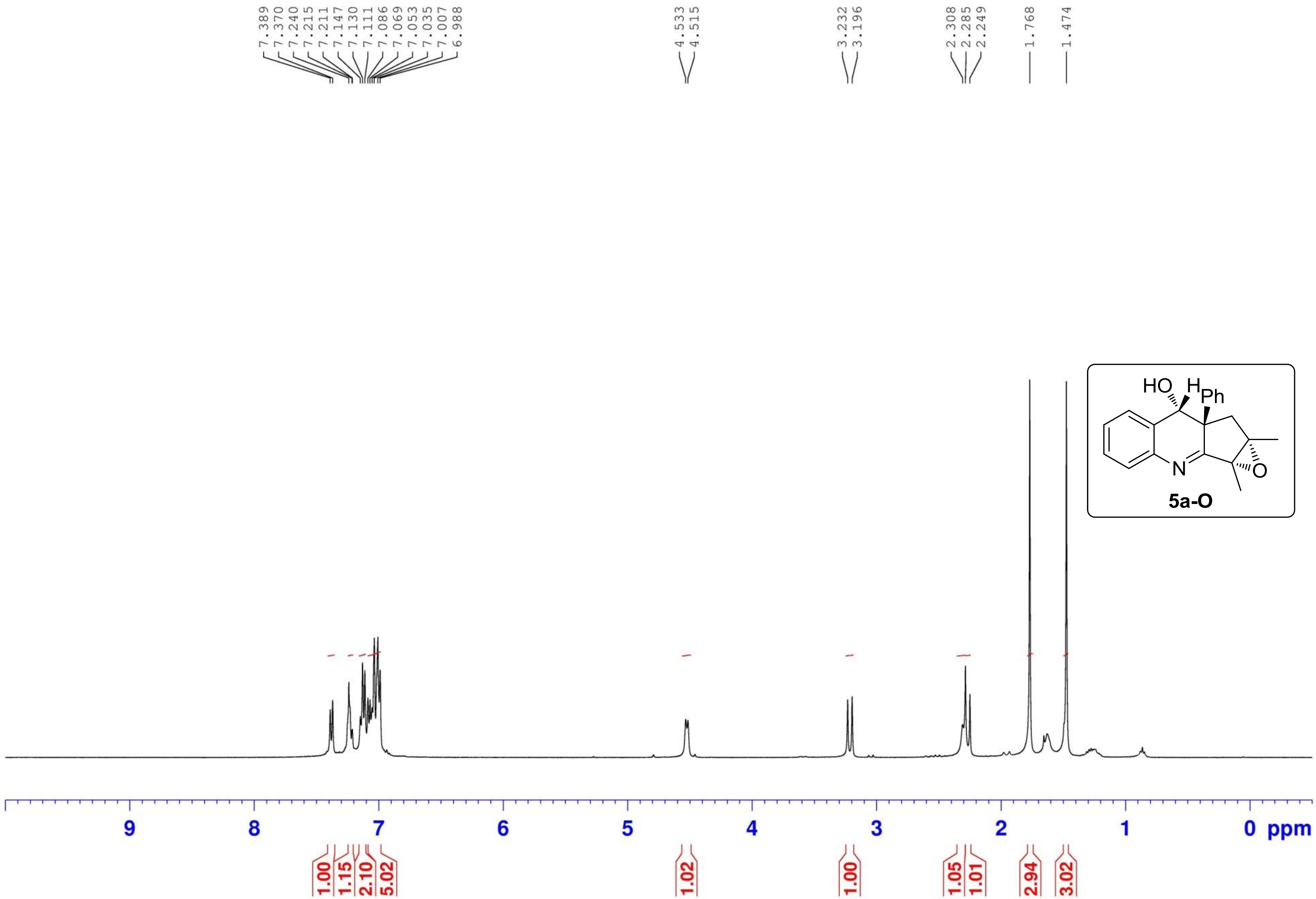
7.389
7.370
7.240
7.215
7.211
7.147
7.130
7.111
7.086
7.069
7.053
7.035
7.007
6.988
4.533
4.515
3.232
3.196
2.308
2.285
2.249
1.768
1.474
HO
H
Ph
N
O
5a-O
9
8
7
6
5
4
3
2
1
0 ppm
1.00
1.15
2.10
5.02
1.02
1.00
1.05
1.01
2.94
3.02

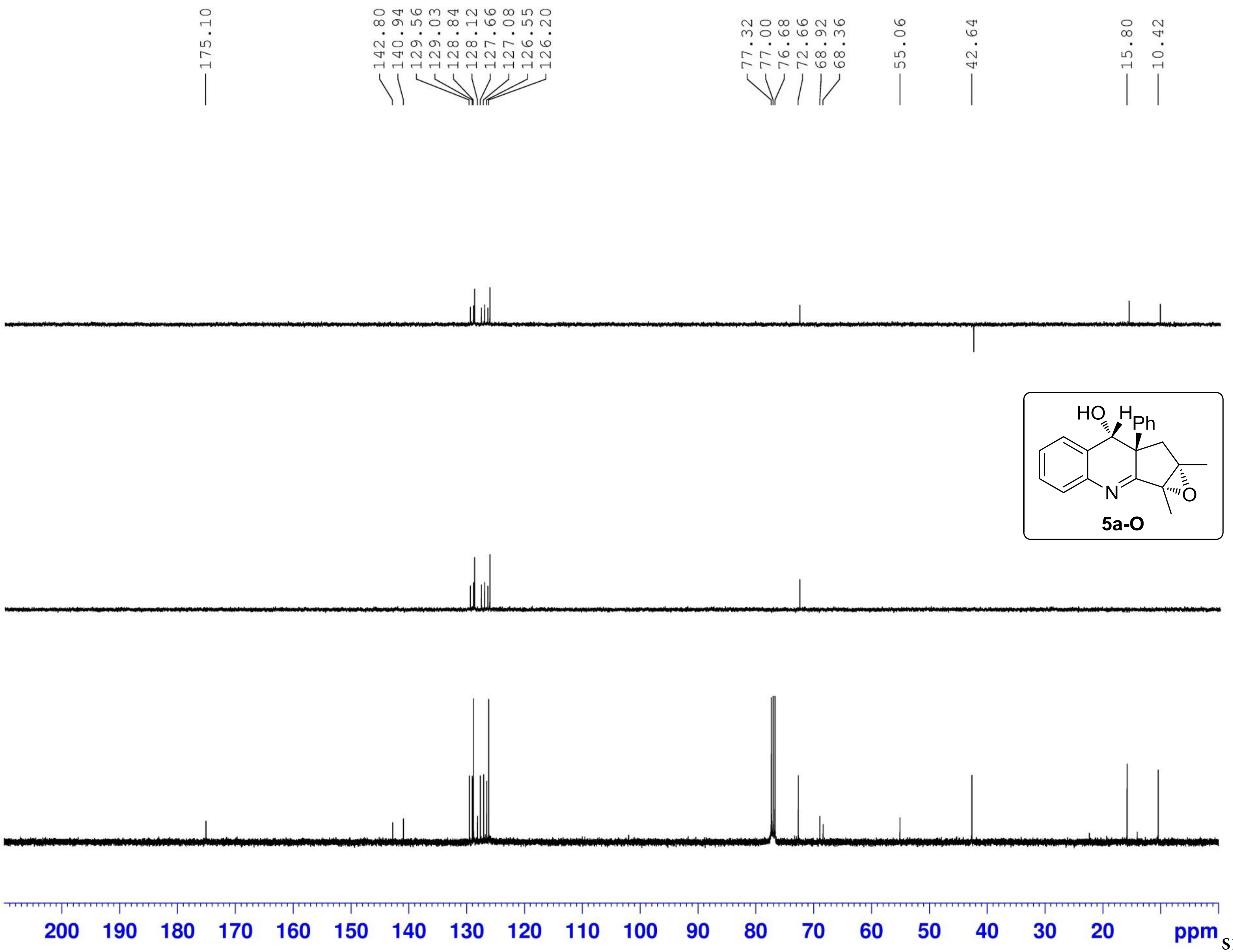
175.10
142.80
140.94
129.56
129.03
128.84
128.12
127.66
127.08
126.55
126.20
77.32
77.00
76.68
72.66
68.92
68.36
55.06
42.64
15.80
10.42
HO
H
Ph
N
O
5a-O
200 190 180 170 160 150 140 130 120 110 100 90 80 70 60 50 40 30 20 ppm

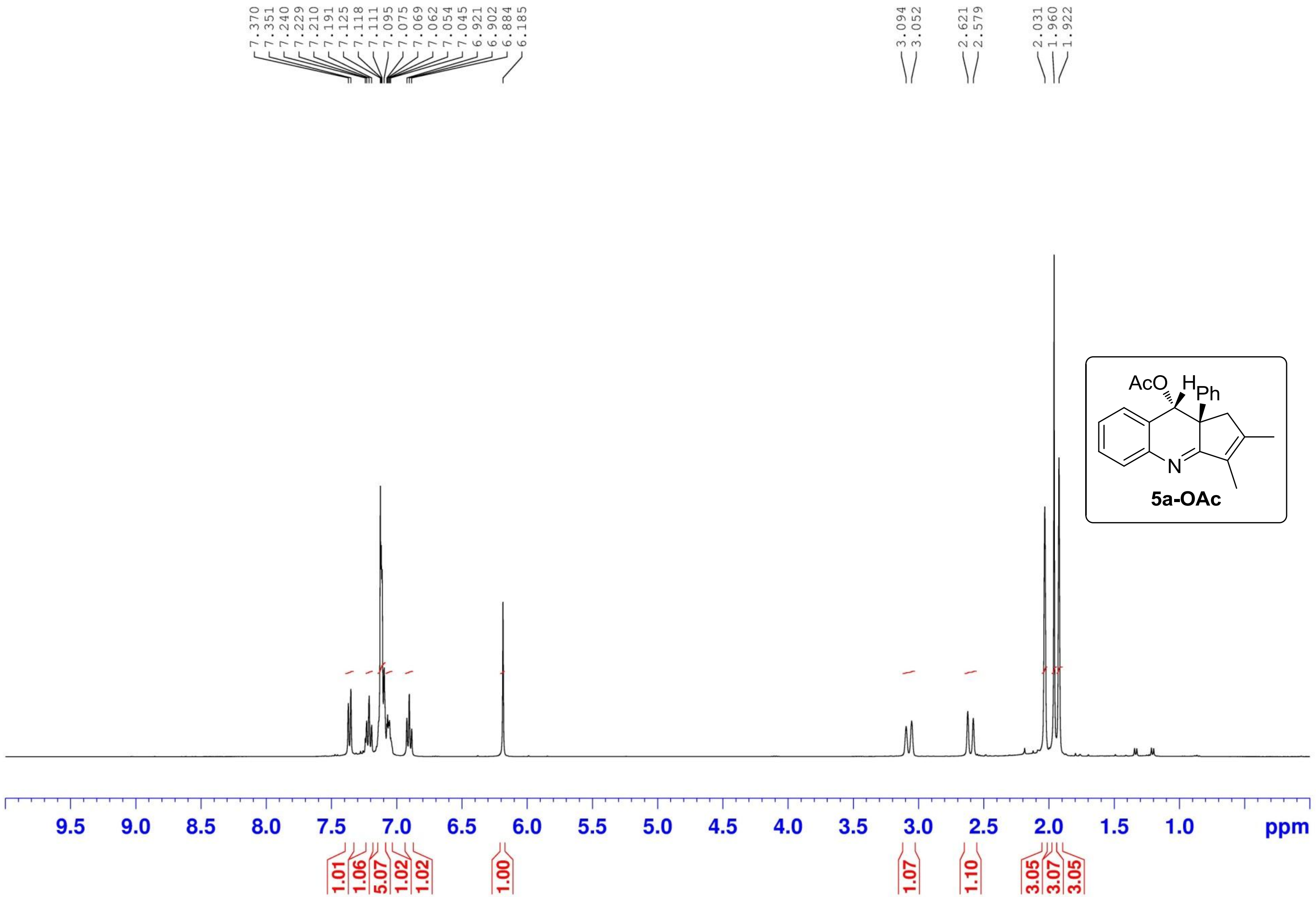
7.370
7.351
7.240
7.229
7.210
7.191
7.125
7.118
7.111
7.095
7.075
7.069
7.062
7.054
7.045
6.921
6.902
6.884
6.185
3.094
3.052
2.621
2.579
2.031
1.960
1.922
AcO
H
Ph
N
5a-OAc
9.5
9.0
8.5
8.0
7.5
7.0
6.5
6.0
5.5
5.0
4.5
4.0
3.5
3.0
2.5
2.0
1.5
1.0
ppm
1.01
1.06
5.07
1.02
1.02
1.00
1.07
1.10
3.05
3.07
3.05

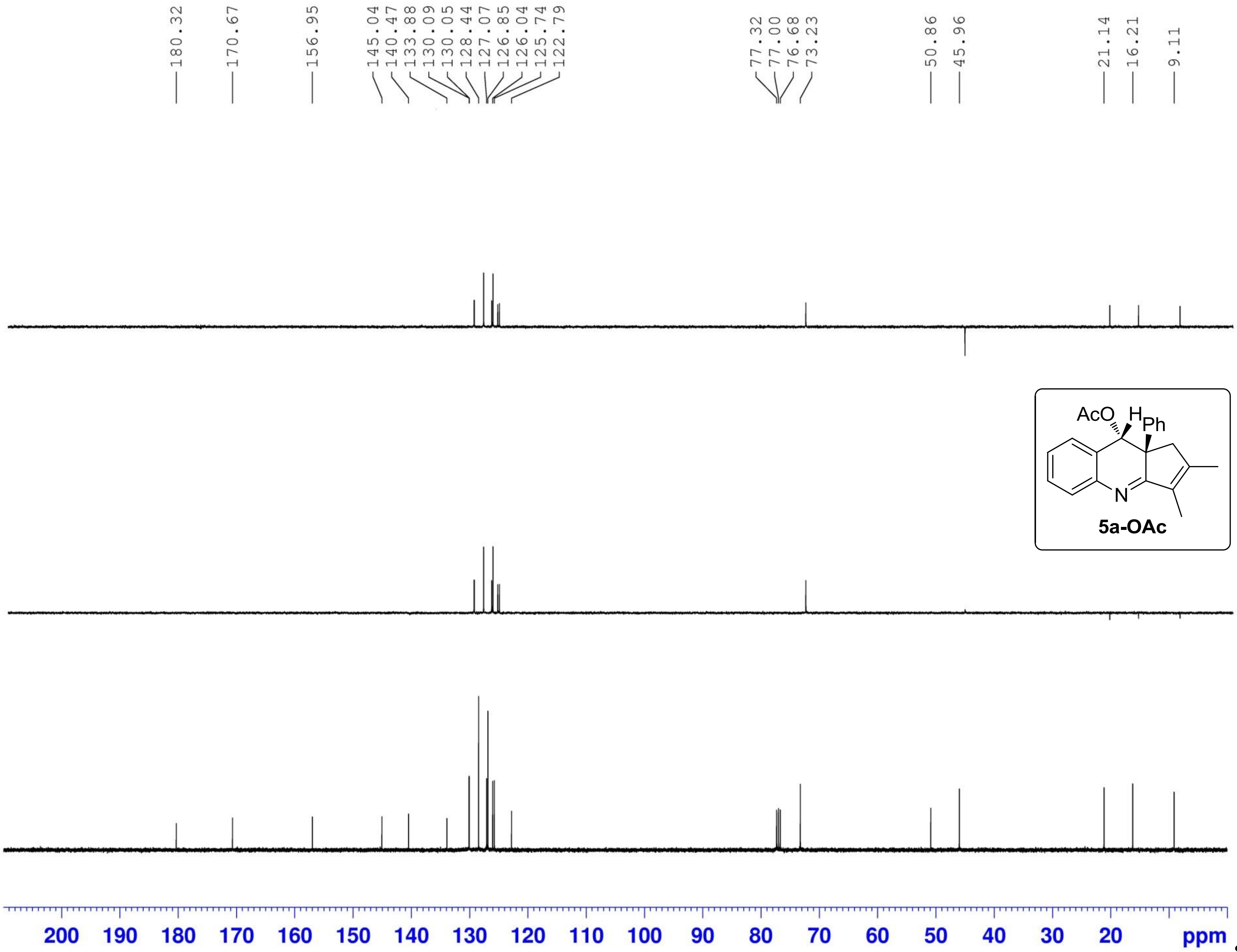

180.32
170.67
156.95
145.04
140.47
133.88
130.09
130.05
128.44
127.07
126.85
126.04
125.74
122.79
77.32
77.00
76.68
73.23
50.86
45.96
21.14
16.21
9.11
AcO
H
Ph
N
5a-OAc
200 190 180 170 160 150 140 130 120 110 100 90 80 70 60 50 40 30 20 ppm

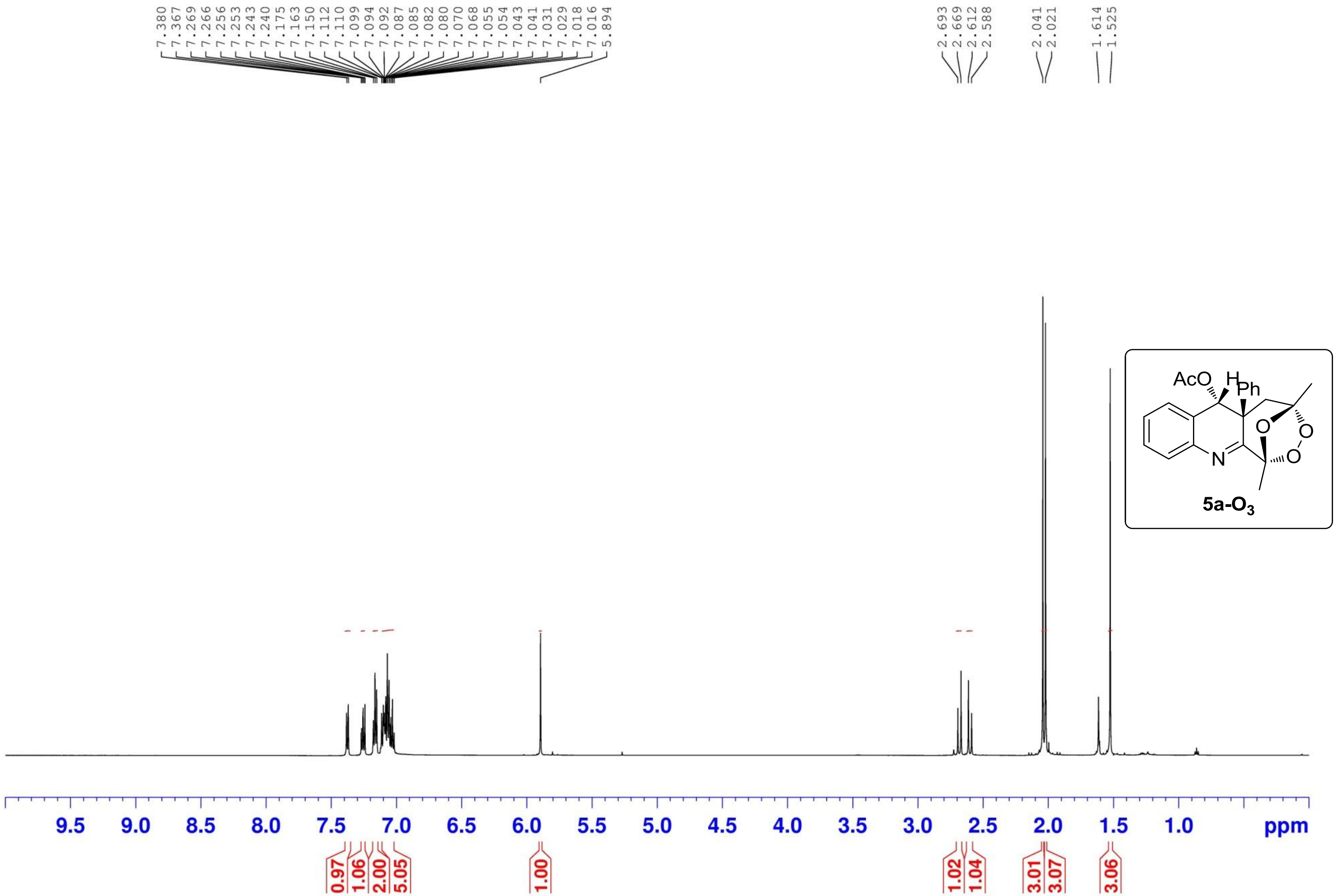
7.380
7.367
7.269
7.266
7.256
7.253
7.243
7.240
7.175
7.163
7.150
7.112
7.110
7.099
7.094
7.092
7.087
7.085
7.082
7.080
7.070
7.068
7.055
7.054
7.043
7.041
7.031
7.029
7.018
7.016
5.894
2.693
2.669
2.612
2.588
2.041
2.021
1.614
1.525
AcO
H
Ph
N
O
O
O
5a-O3
9.5
9.0
8.5
8.0
7.5
7.0
6.5
6.0
5.5
5.0
4.5
4.0
3.5
3.0
2.5
2.0
1.5
1.0
ppm
0.97
1.06
2.00
5.05
1.00
1.02
1.04
3.01
3.07
3.06

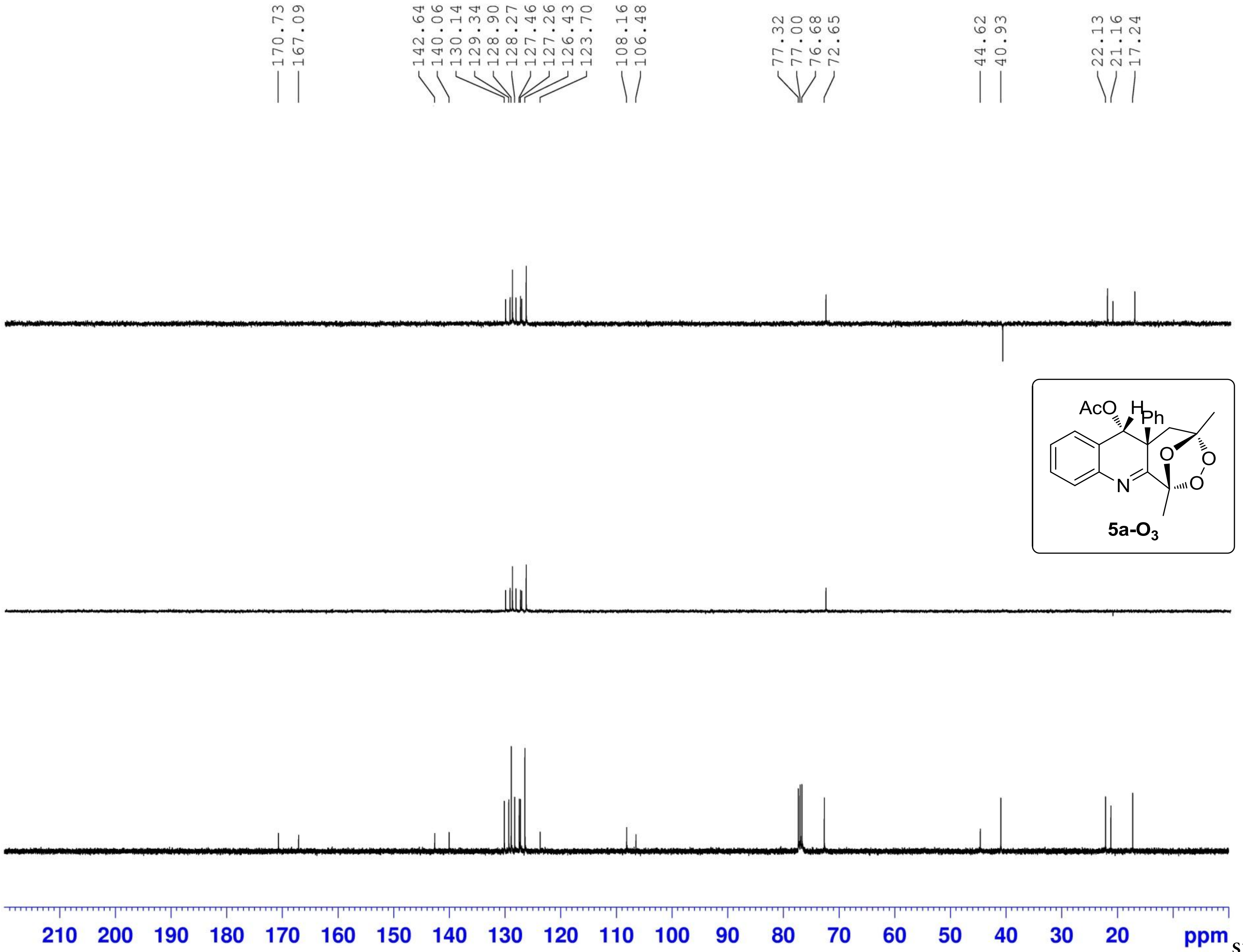
170.73
167.09
142.64
140.06
130.14
129.34
128.90
128.27
127.46
127.26
126.43
123.70
108.16
106.48
77.32
77.00
76.68
72.65
44.62
40.93
22.13
21.16
17.24
AcO
H
Ph
O
O
O
N
5a-O_3
210 200 190 180 170 160 150 140 130 120 110 100 90 80 70 60 50 40 30 20
ppm

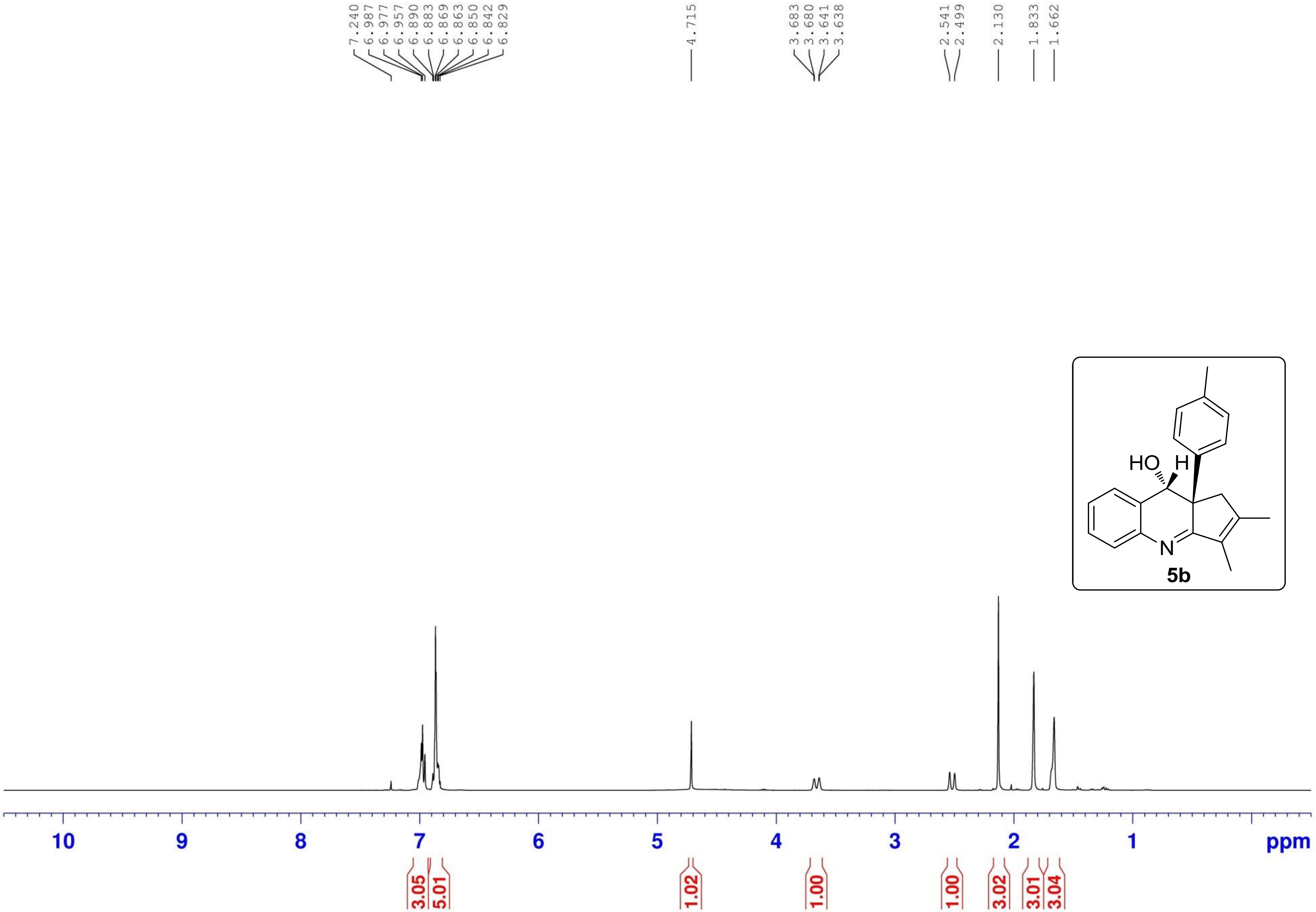

7.240
6.987
6.977
6.957
6.890
6.883
6.869
6.863
6.850
6.842
6.829
4.715
3.683
3.680
3.641
3.638
2.541
2.499
2.130
1.833
1.662
HO
H
N
5b
10
9
8
7
6
5
4
3
2
1
ppm
3.05
5.01
1.02
1.00
1.00
3.02
3.01
3.04

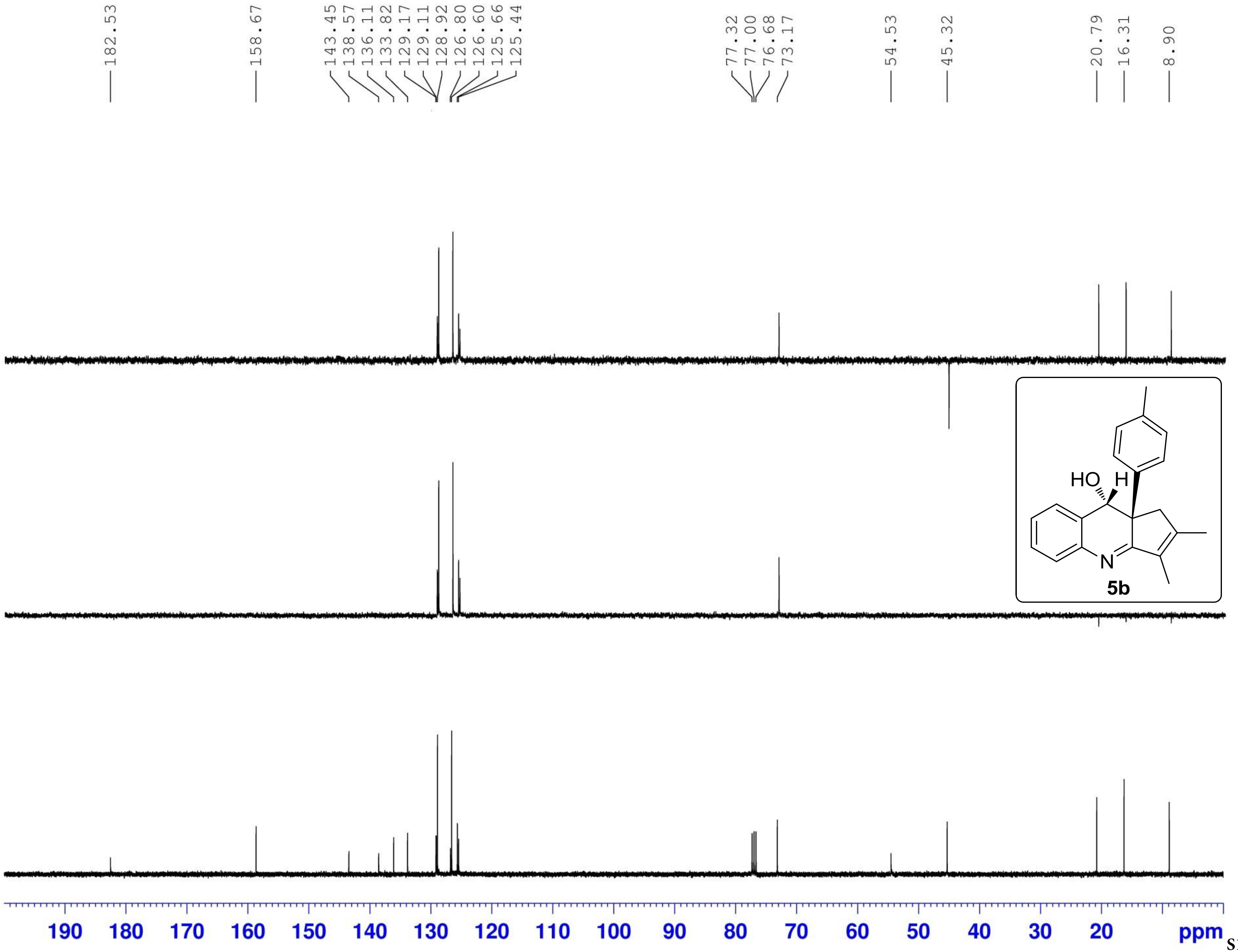
182.53
158.67
143.45
138.57
136.11
133.82
129.17
129.11
128.92
126.80
126.60
125.66
125.44
77.32
77.00
76.68
73.17
54.53
45.32
20.79
16.31
8.90
HO
H
N
5b
190
180
170
160
150
140
130
120
110
100
90
80
70
60
50
40
30
20
ppm

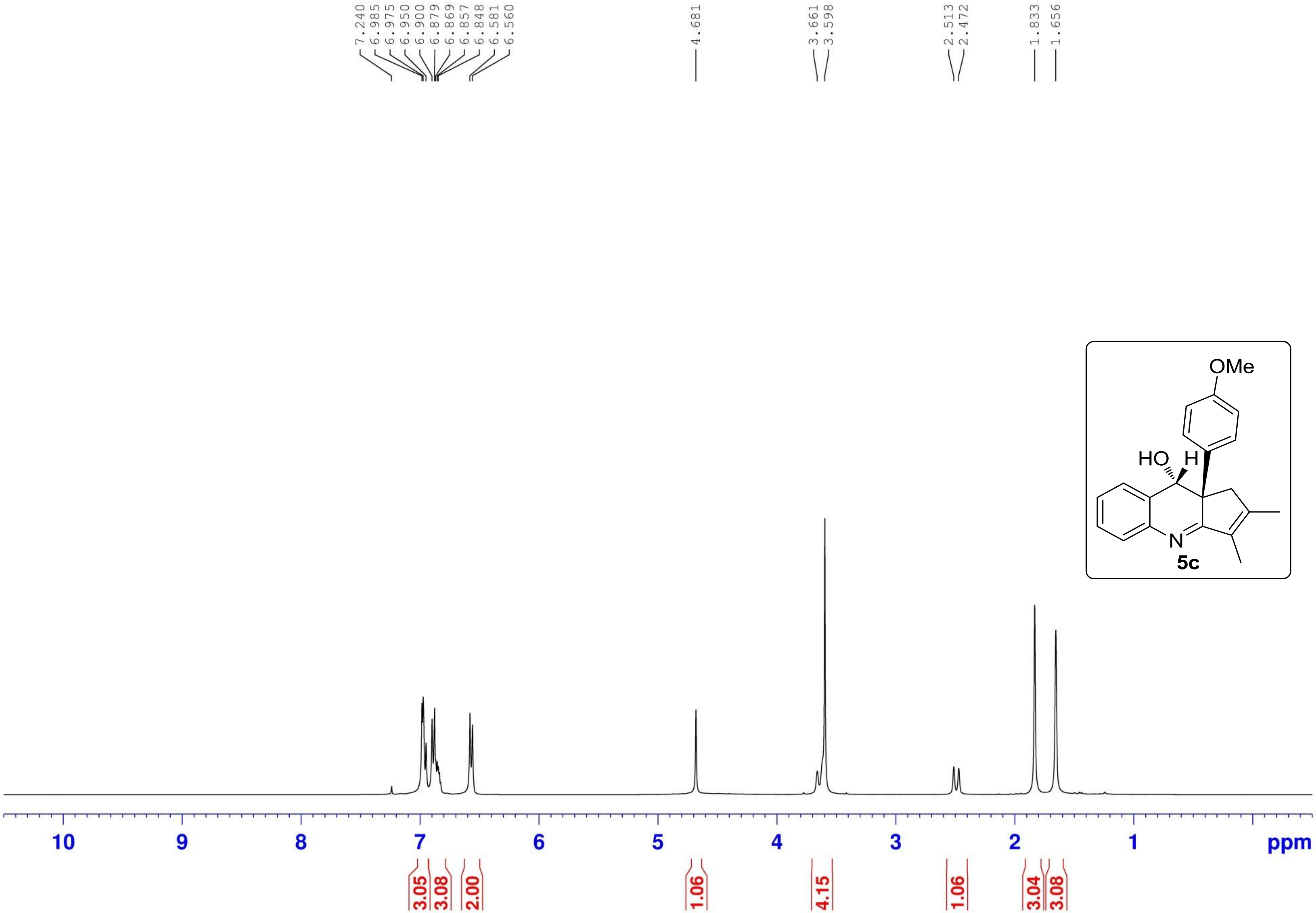
7.240
6.985
6.975
6.950
6.900
6.879
6.869
6.857
6.848
6.581
6.560
4.681
3.661
3.598
2.513
2.472
1.833
1.656
OMe
HO
H
N
5c
10
9
8
7
6
5
4
3
2
1
ppm
3.05
3.08
2.00
1.06
4.15
1.06
3.04
3.08

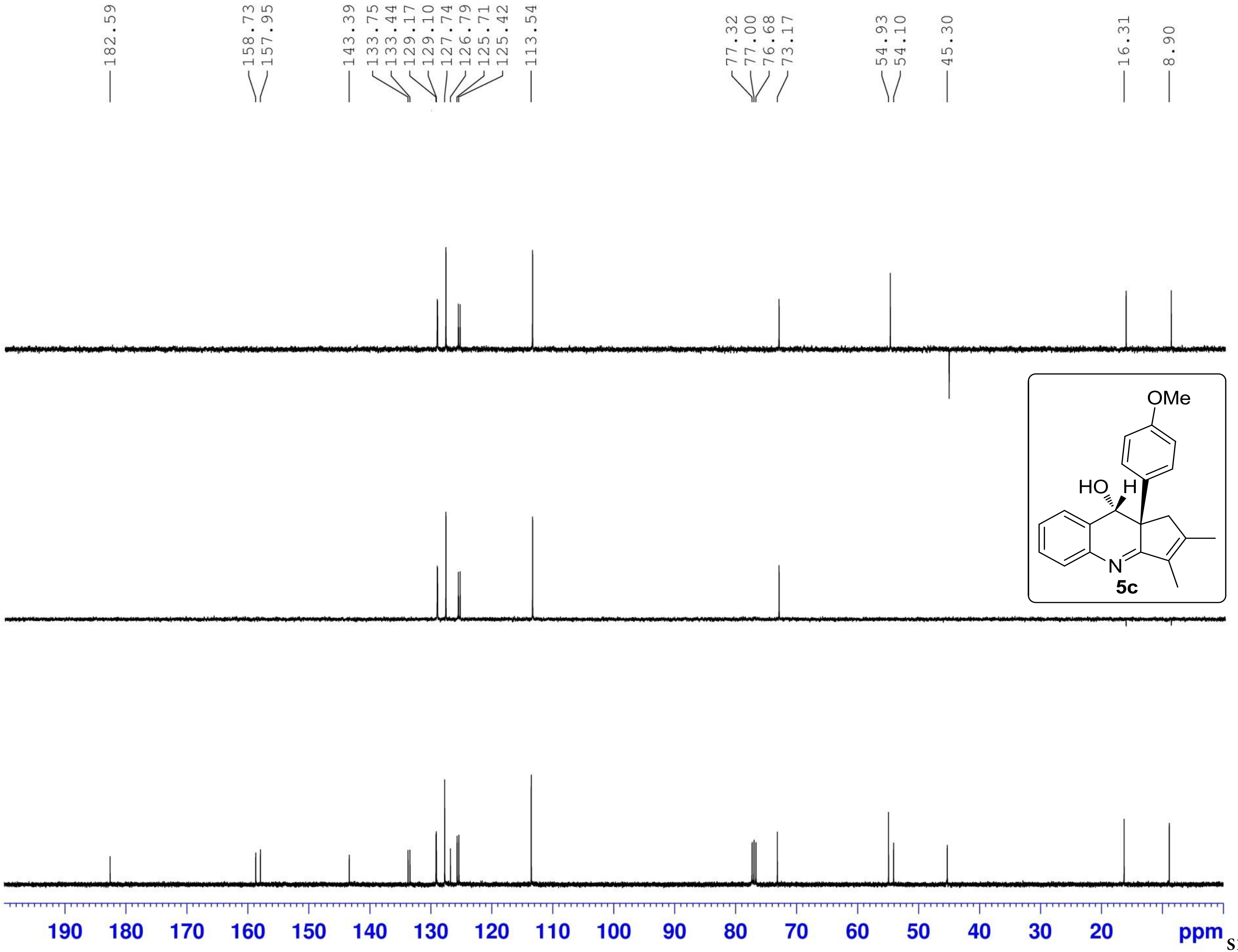
182.59
158.73
157.95
143.39
133.75
133.44
129.17
129.10
127.74
126.79
125.71
125.42
113.54
77.32
77.00
76.68
73.17
54.93
54.10
45.30
16.31
8.90
OMe
HO
H
N
5c
190
180
170
160
150
140
130
120
110
100
90
80
70
60
50
40
30
20
ppm

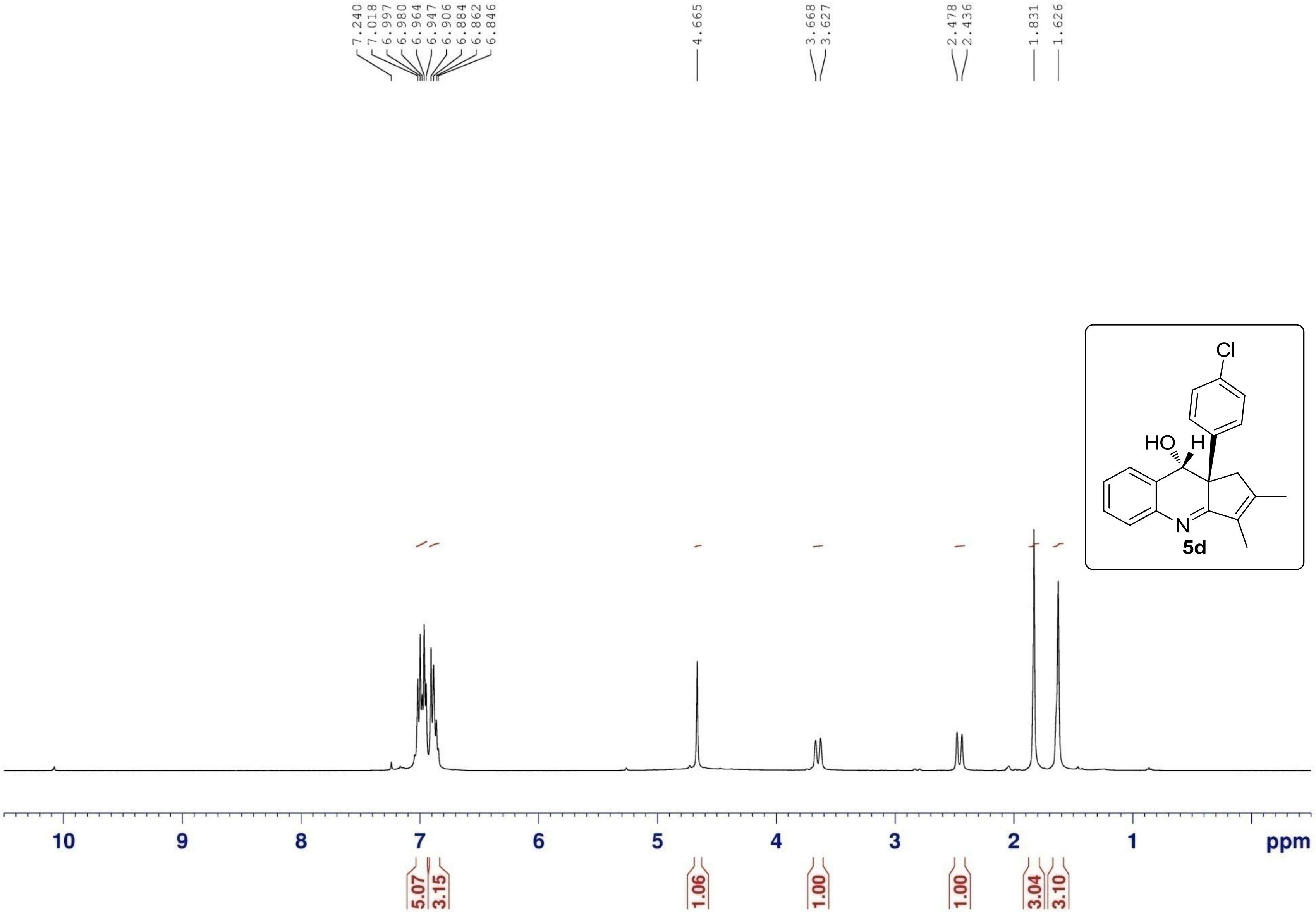
7.240
7.018
6.997
6.980
6.964
6.947
6.906
6.884
6.862
6.846
4.665
3.668
3.627
2.478
2.436
1.831
1.626
Cl
HO
H
N
5d
10
9
8
7
6
5
4
3
2
1
ppm
5.07
3.15
1.06
1.00
1.00
3.04
3.10

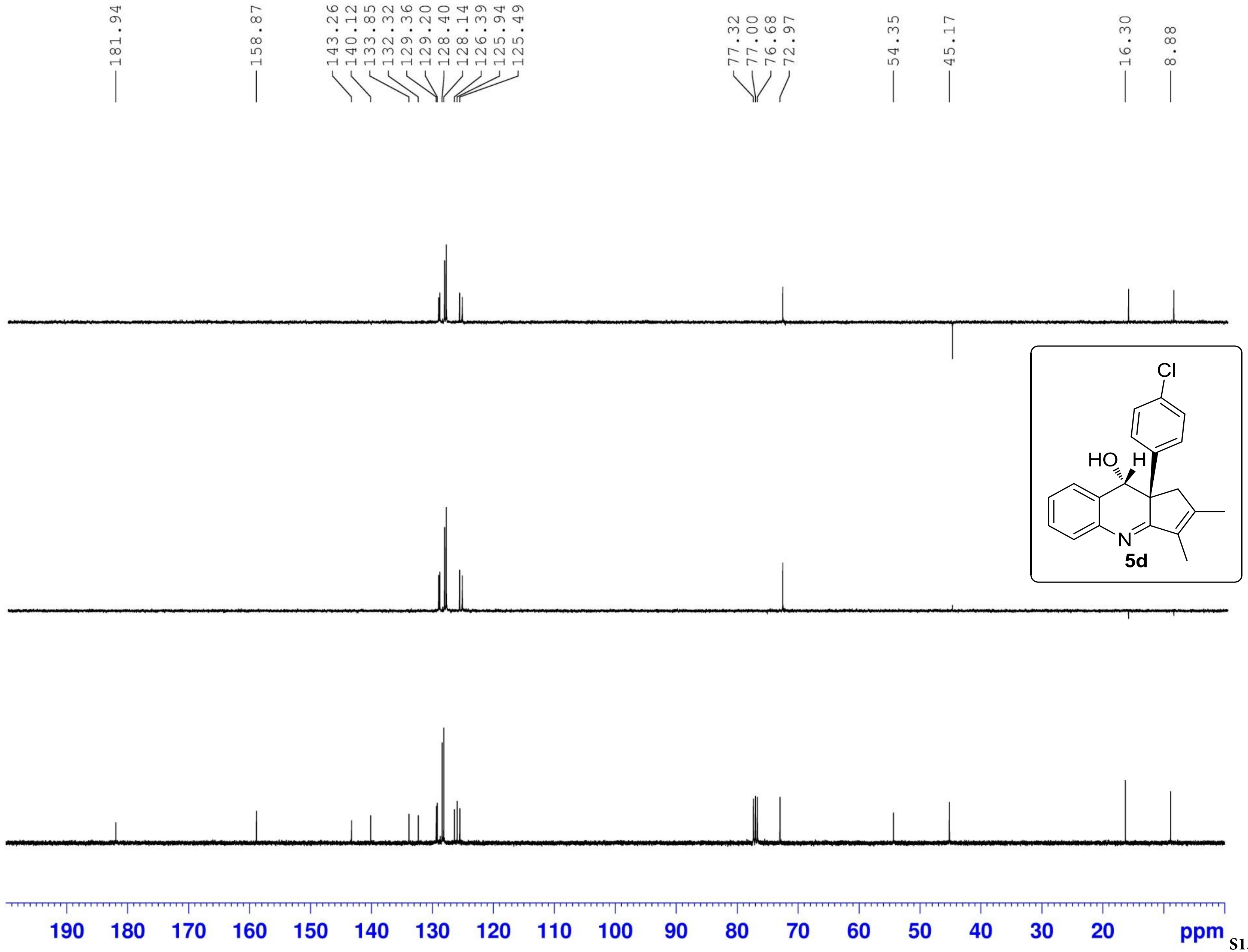
181.94
158.87
143.26
140.12
133.85
132.32
129.36
129.20
128.40
128.14
126.39
125.94
125.49
77.32
77.00
76.68
72.97
54.35
45.17
16.30
8.88
Cl
HO
H
N
5d
190 180 170 160 150 140 130 120 110 100 90 80 70 60 50 40 30 20 ppm

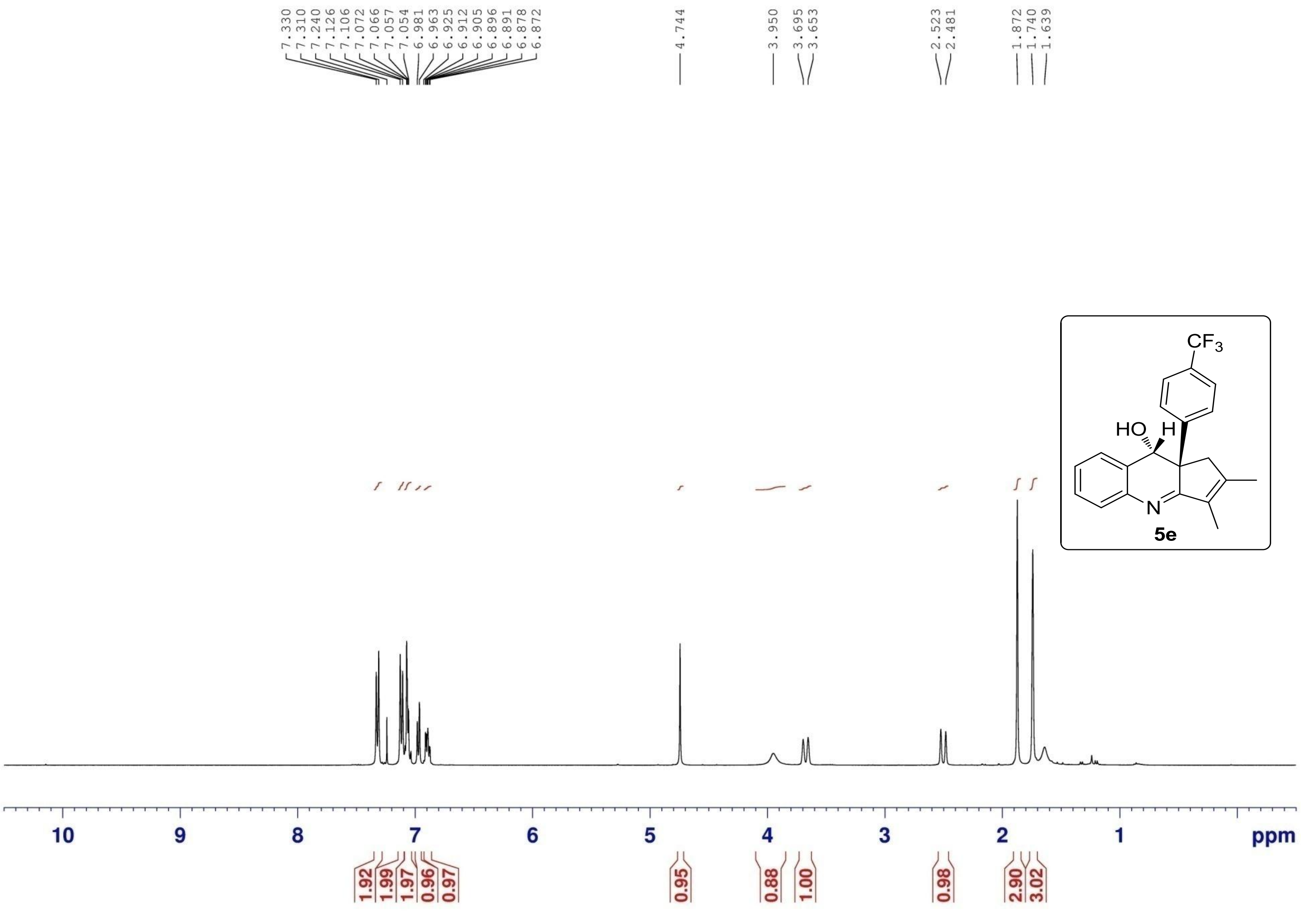
7.330
7.310
7.240
7.126
7.106
7.072
7.066
7.057
7.054
6.981
6.963
6.925
6.912
6.905
6.896
6.891
6.878
6.872
4.744
3.950
3.695
3.653
2.523
2.481
1.872
1.740
1.639
CF3
HO
H
N
5e
10
9
8
7
6
5
4
3
2
1
ppm
1.92
1.99
1.97
0.96
0.97
0.95
0.88
1.00
0.98
2.90
3.02

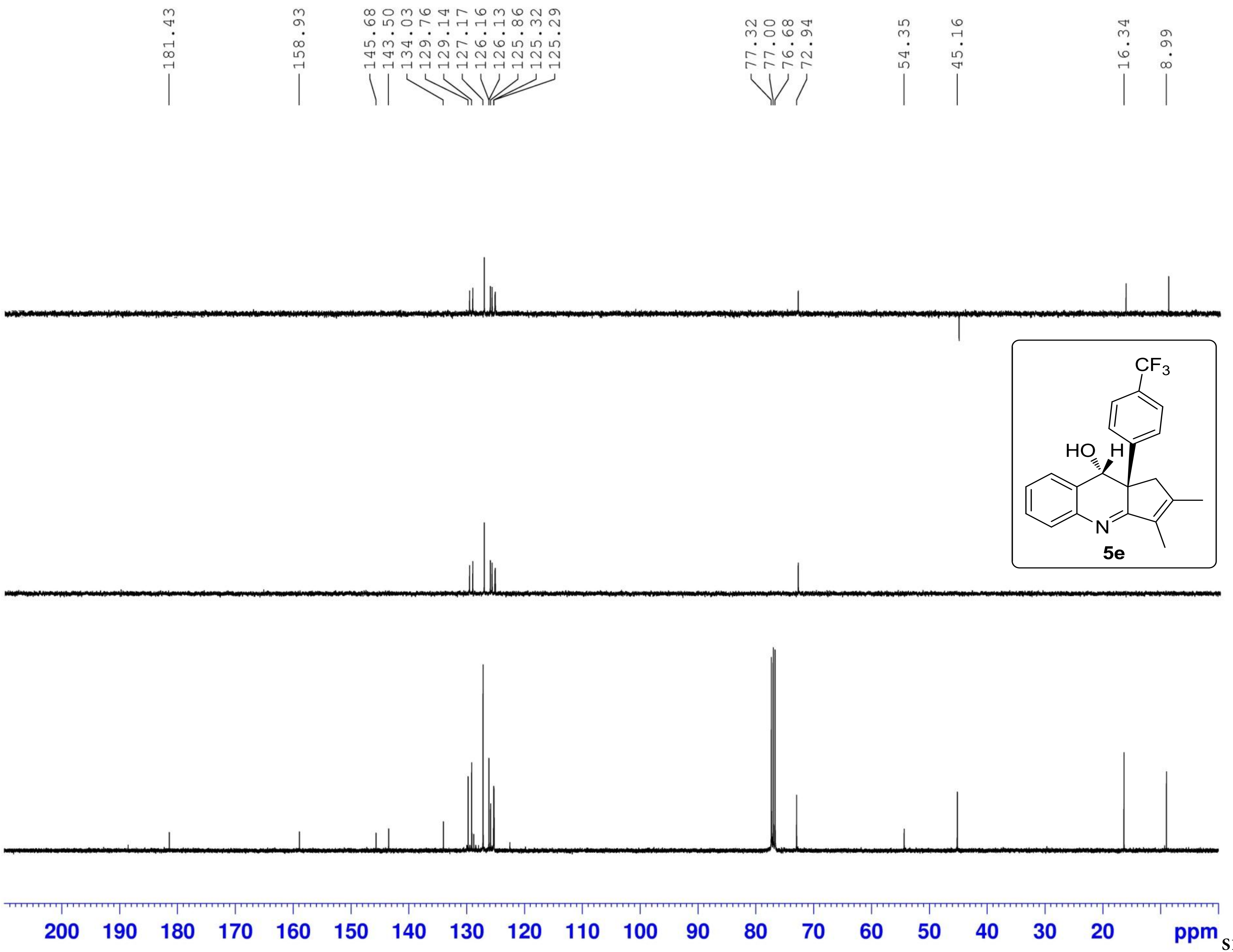
181.43
158.93
145.68
143.50
134.03
129.76
129.14
127.17
126.16
126.13
125.86
125.32
125.29
77.32
77.00
76.68
72.94
54.35
45.16
16.34
8.99
CF3
HO
H
N
5e
200
190
180
170
160
150
140
130
120
110
100
90
80
70
60
50
40
30
20
ppm

PDJ-C33

Current Data Parameters
NAME liou0403.001
EXPNO 1
PROCNO 1

F2 - Acquisition Parameters
Date_ 20180403
Time 15.17 h
INSTRUM spect
PROBHD Z119470_0234 (
PULPROG zgfhigqn.2
TD 131072
SOLVENT CDCl3
NS 128
DS 4
SWH 113636.367 Hz
FIDRES 0.866977 Hz
AQ 0.5767168 sec
RG 191.01
DW 4.400 usec
DE 6.50 usec
TE 299.6 K
D1 1.00000000 sec
D11 0.03000000 sec
D12 0.00002000 sec
TD0 1
SFO1 470.5735434 MHz
NUC1 19F
P1 15.00 usec
PLW1 45.00000000 W
SFO2 500.1620006 MHz
NUC2 1H
CPDPRG[2 waltz16
PCPD2 80.00 usec
PLW2 26.00000000 W
PLW12 0.40625000 W

F2 - Processing parameters
SI 65536
SF 470.6206054 MHz
WDW EM
SSB 0
LB 3.00 Hz
GB 0
PC 1.00

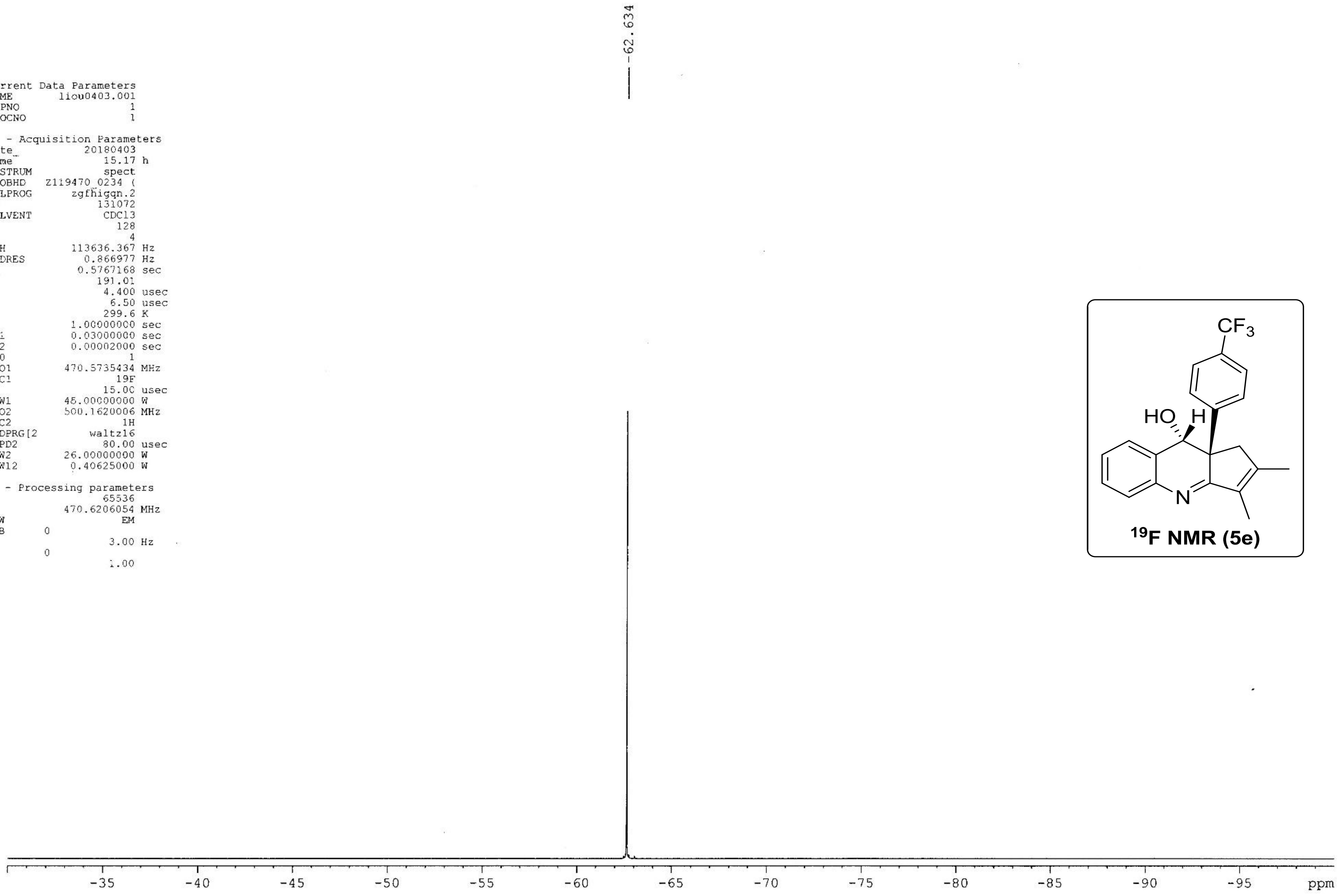

^{19}F NMR (5e)

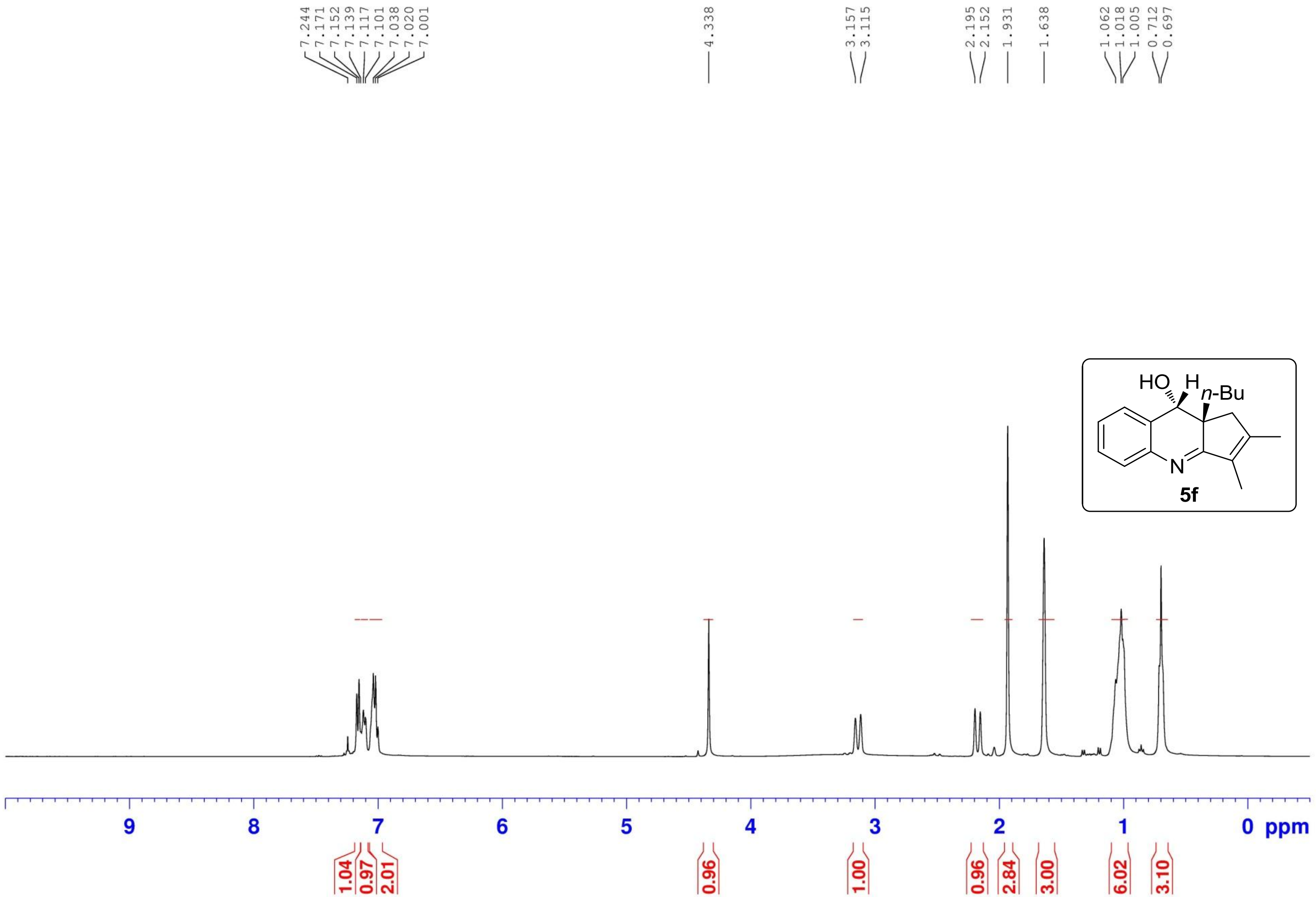
7.244
7.171
7.152
7.139
7.117
7.101
7.038
7.020
7.001
4.338
3.157
3.115
2.195
2.152
1.931
1.638
1.062
1.018
1.005
0.712
0.697
HO
H
n-Bu
N
5f
9
8
7
6
5
4
3
2
1
0 ppm
1.04
0.97
2.01
0.96
1.00
0.96
2.84
3.00
6.02
3.10

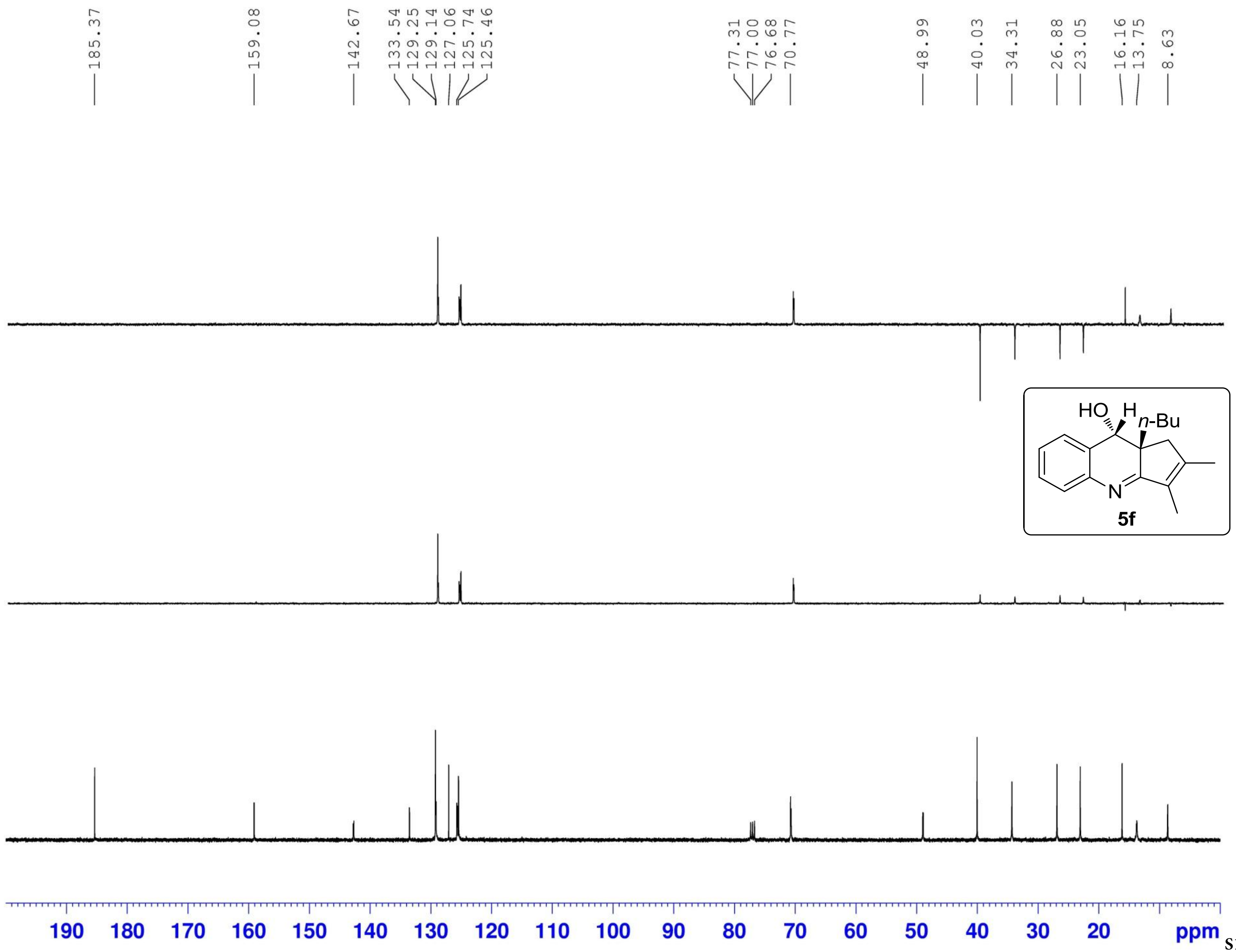
185.37
159.08
142.67
133.54
129.25
129.14
127.06
125.74
125.46
77.31
77.00
76.68
70.77
48.99
40.03
34.31
26.88
23.05
16.16
13.75
8.63
HO
H
n-Bu
N
5f
190 180 170 160 150 140 130 120 110 100 90 80 70 60 50 40 30 20
ppm

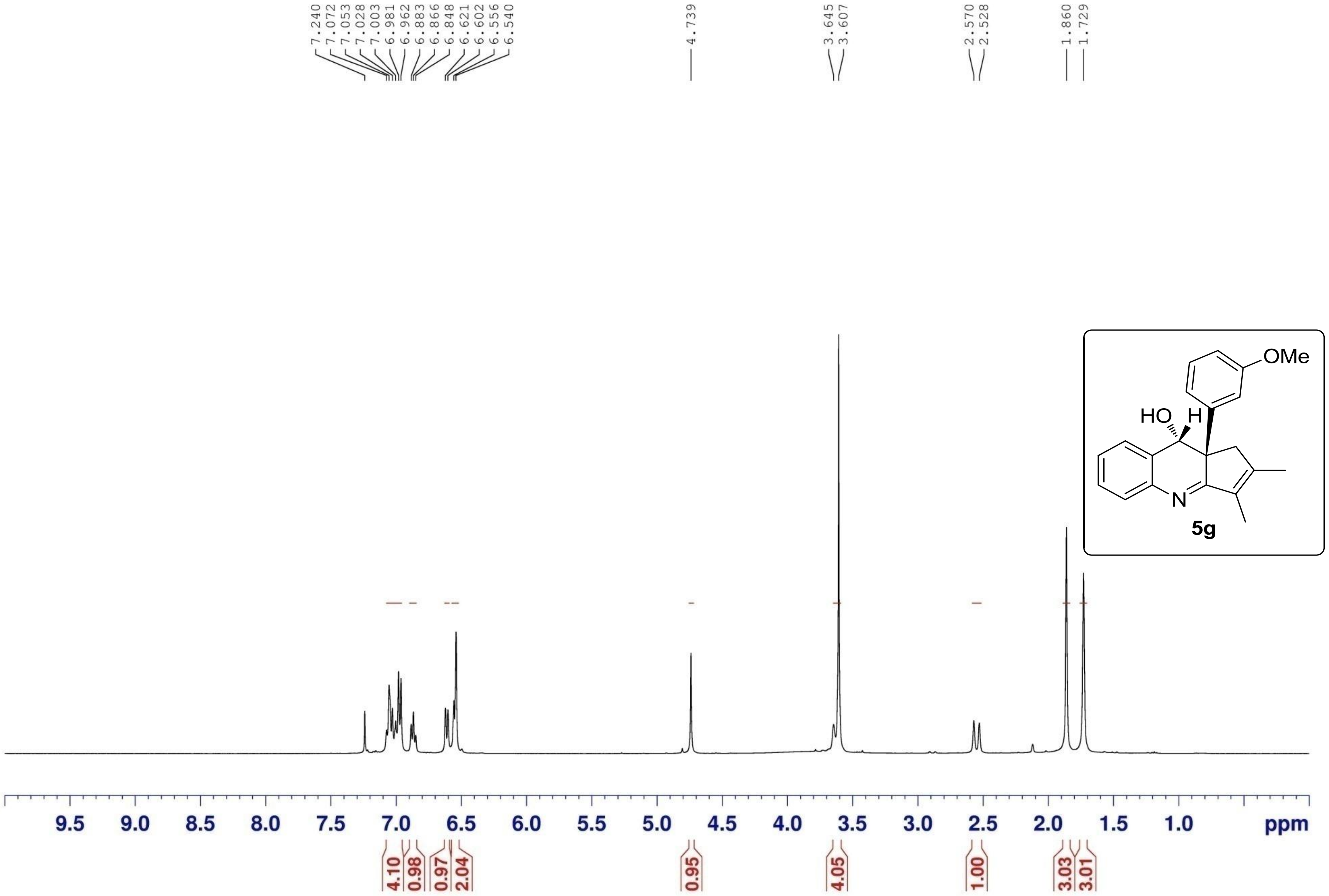
7.240
7.072
7.053
7.028
7.003
6.981
6.962
6.883
6.866
6.848
6.621
6.602
6.556
6.540
4.739
3.645
3.607
2.570
2.528
1.860
1.729
OMe
HO
H
N
5g
9.5
9.0
8.5
8.0
7.5
7.0
6.5
6.0
5.5
5.0
4.5
4.0
3.5
3.0
2.5
2.0
1.5
1.0
ppm
4.10
0.98
0.97
2.04
0.95
4.05
1.00
3.03
3.01

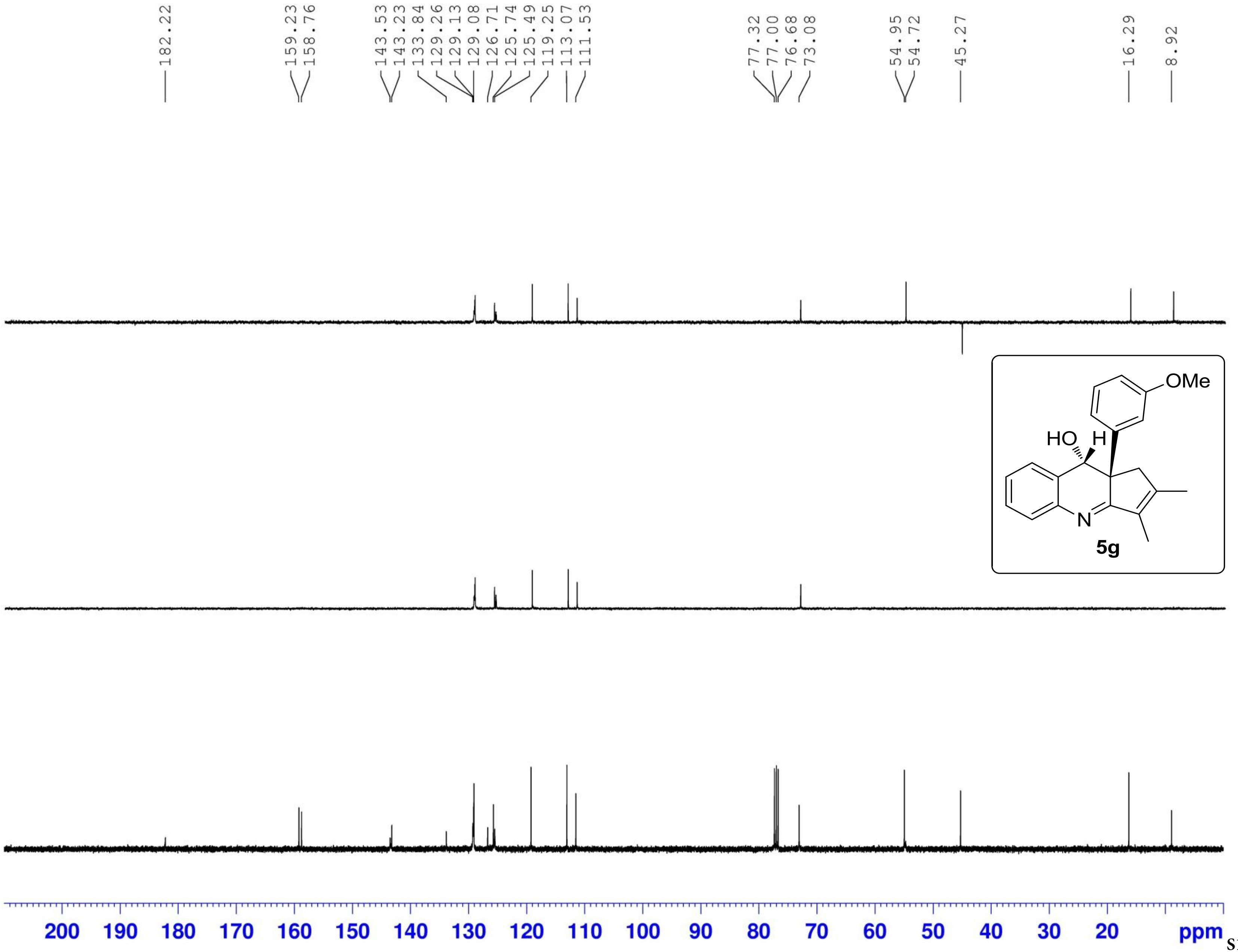

182.22
159.23
158.76
143.53
143.23
133.84
129.26
129.13
129.08
126.71
125.74
125.49
119.25
113.07
111.53
77.32
77.00
76.68
73.08
54.95
54.72
45.27
16.29
8.92
OMe
HO
H
N
5g
200 190 180 170 160 150 140 130 120 110 100 90 80 70 60 50 40 30 20 ppm

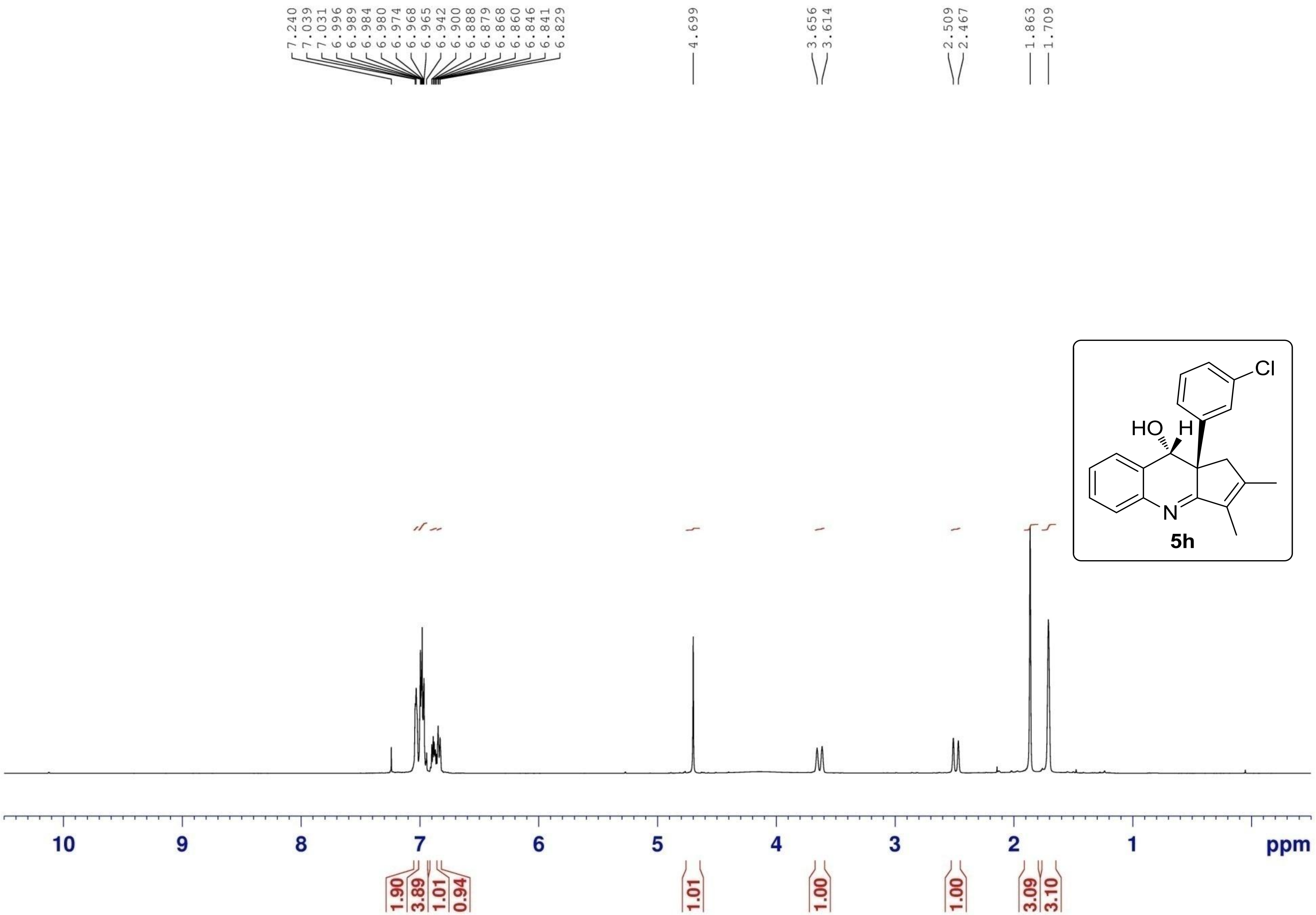
7.240
7.039
7.031
6.996
6.989
6.984
6.980
6.974
6.968
6.965
6.942
6.900
6.888
6.879
6.868
6.860
6.846
6.841
6.829
4.699
3.656
3.614
2.509
2.467
1.863
1.709
Cl
HO
H
N
5h
10
9
8
7
6
5
4
3
2
1
ppm
1.90
3.89
1.01
0.94
1.01
1.00
1.00
3.09
3.10

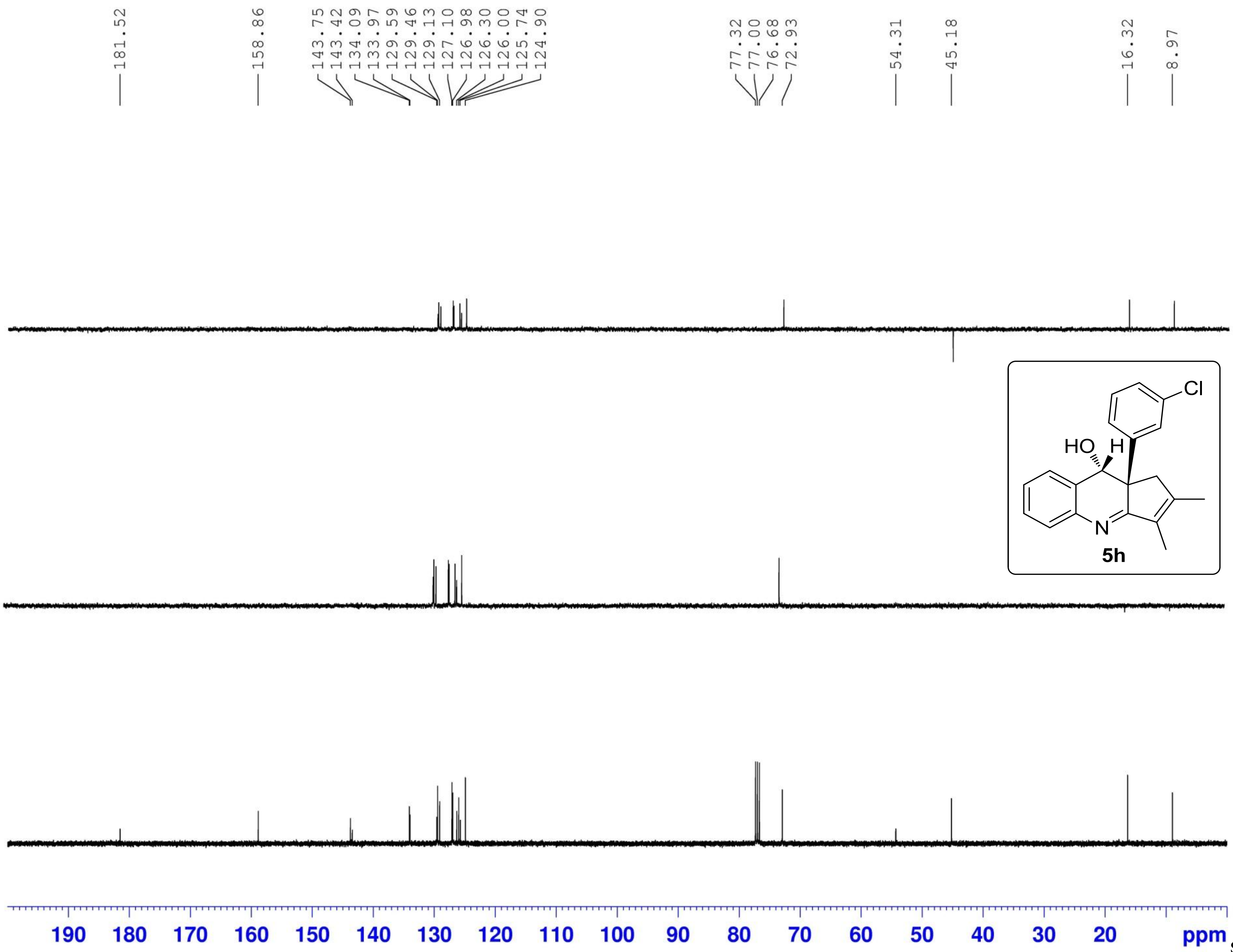
181.52
158.86
143.75
143.42
134.09
133.97
129.59
129.46
129.13
127.10
126.98
126.30
126.00
125.74
124.90
77.32
77.00
76.68
72.93
54.31
45.18
16.32
8.97
HO
H
Cl
N
5h
190 180 170 160 150 140 130 120 110 100 90 80 70 60 50 40 30 20 ppm

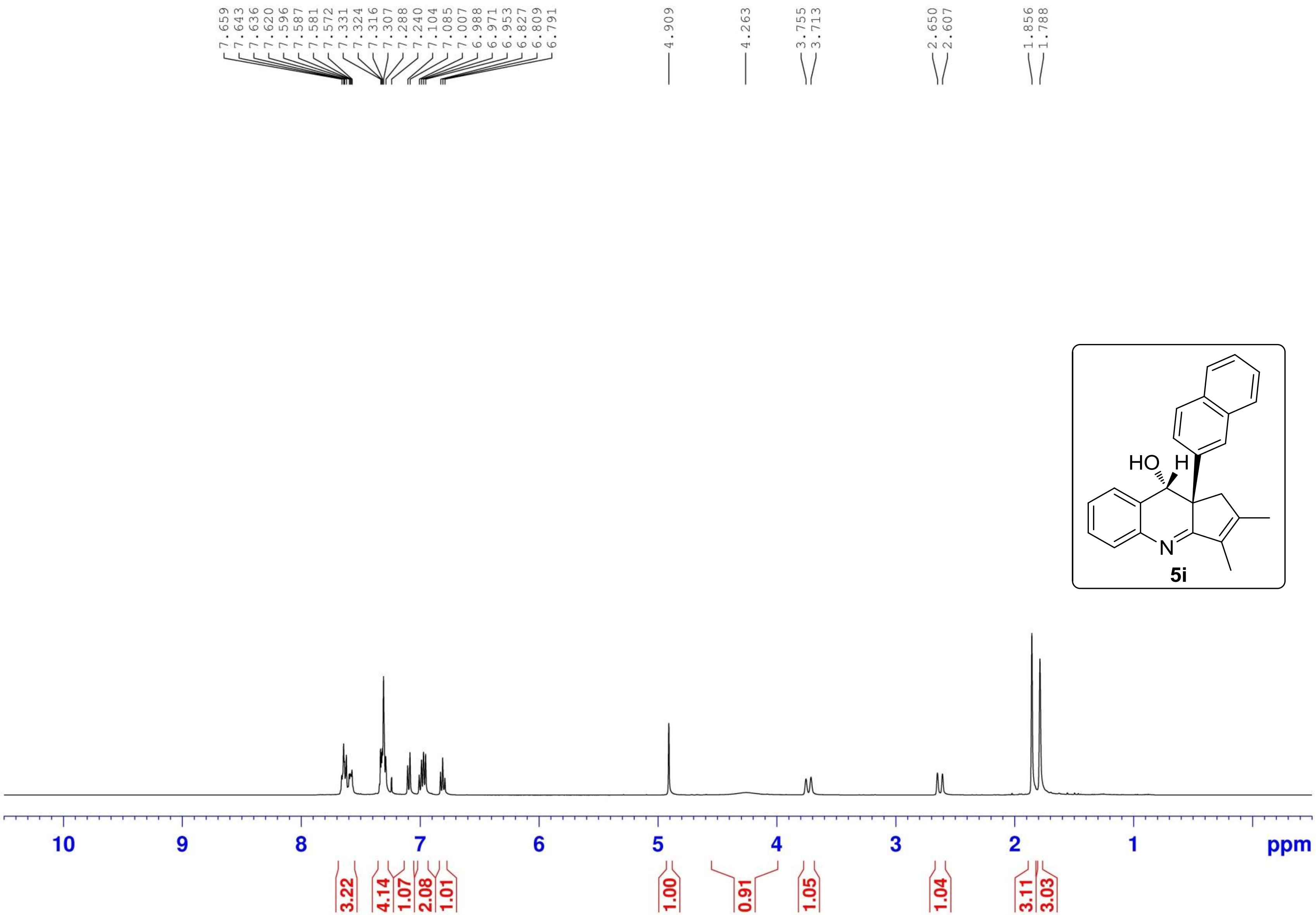
7.659
7.643
7.636
7.620
7.596
7.587
7.581
7.572
7.331
7.324
7.316
7.307
7.288
7.240
7.104
7.085
7.007
6.988
6.971
6.953
6.827
6.809
6.791
4.909
4.263
3.755
3.713
2.650
2.607
1.856
1.788
HO
H
N
5i
10
9
8
7
6
5
4
3
2
1
ppm
3.22
4.14
1.07
2.08
1.01
1.00
0.91
1.05
1.04
3.11
3.03

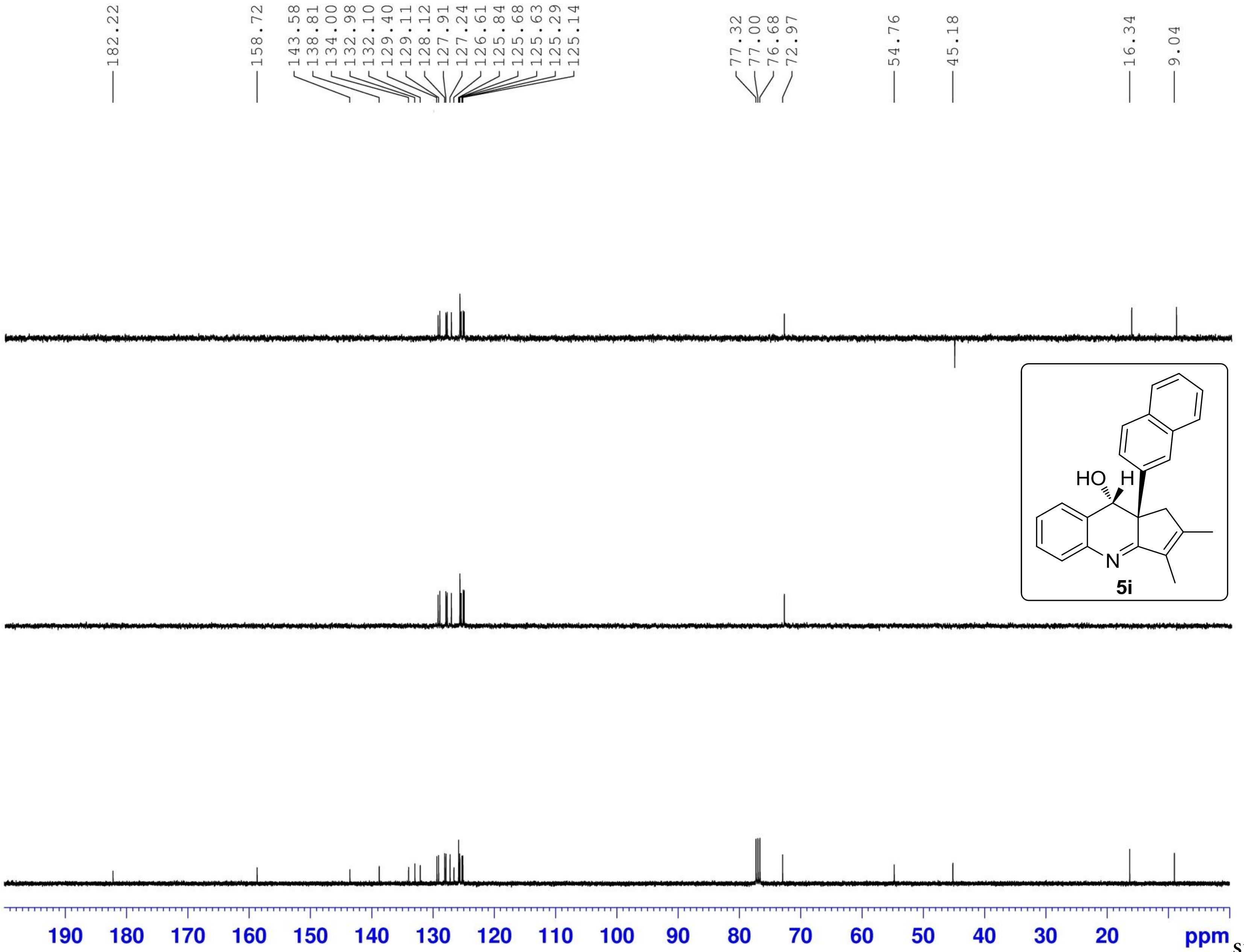
182.22
158.72
143.58
138.81
134.00
132.98
132.10
129.40
129.11
128.12
127.91
127.24
126.61
125.84
125.68
125.63
125.29
125.14
77.32
77.00
76.68
72.97
54.76
45.18
16.34
9.04
HO
H
N
5i
190 180 170 160 150 140 130 120 110 100 90 80 70 60 50 40 30 20 ppm

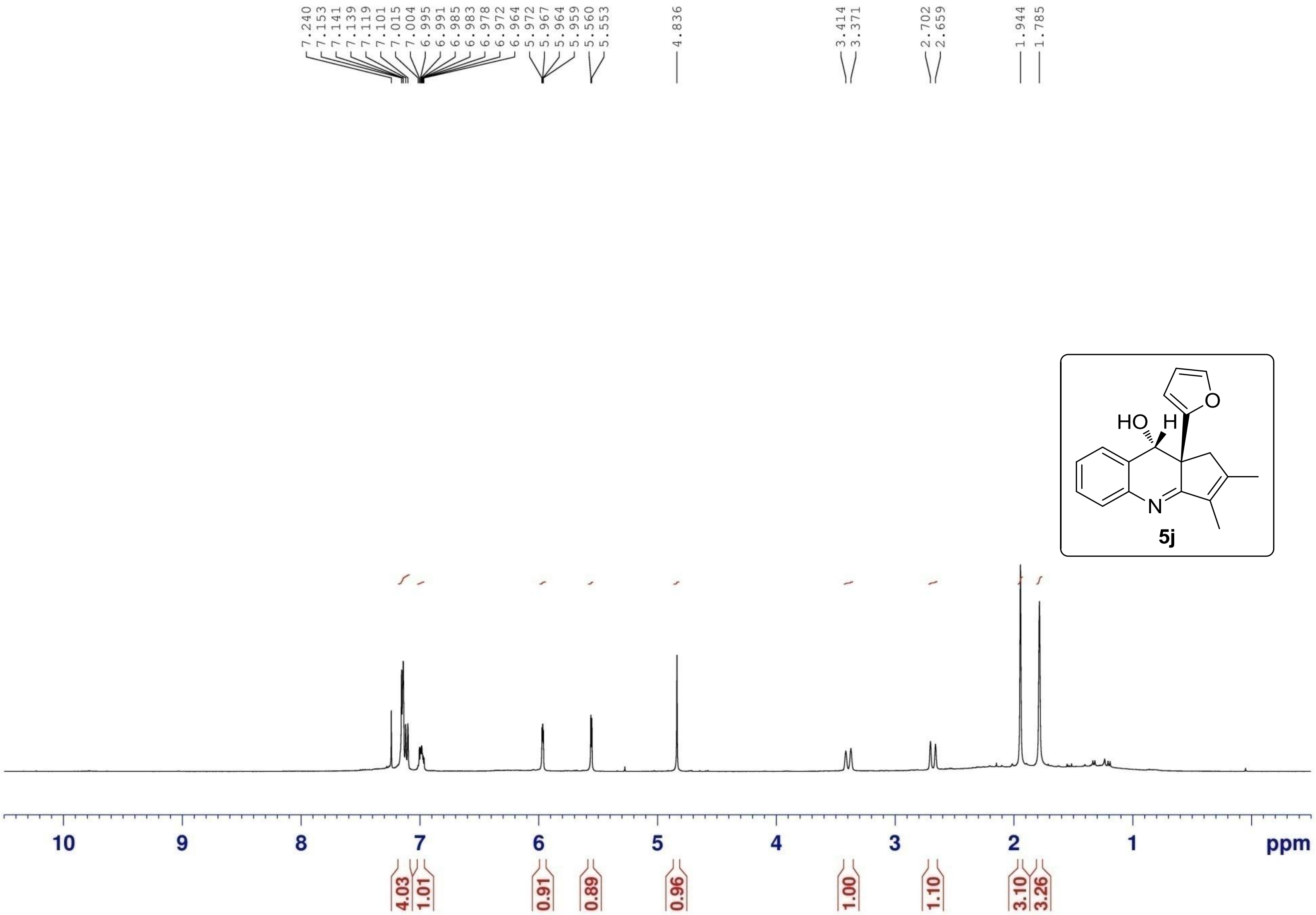
7.240
7.153
7.141
7.139
7.119
7.101
7.015
7.004
6.995
6.991
6.985
6.983
6.978
6.972
6.964
5.972
5.967
5.964
5.959
5.560
5.553
4.836
3.414
3.371
2.702
2.659
1.944
1.785
HO
H
O
N
5j
10
9
8
7
6
5
4
3
2
1
ppm
4.03
1.01
0.91
0.89
0.96
1.00
1.10
3.10
3.26

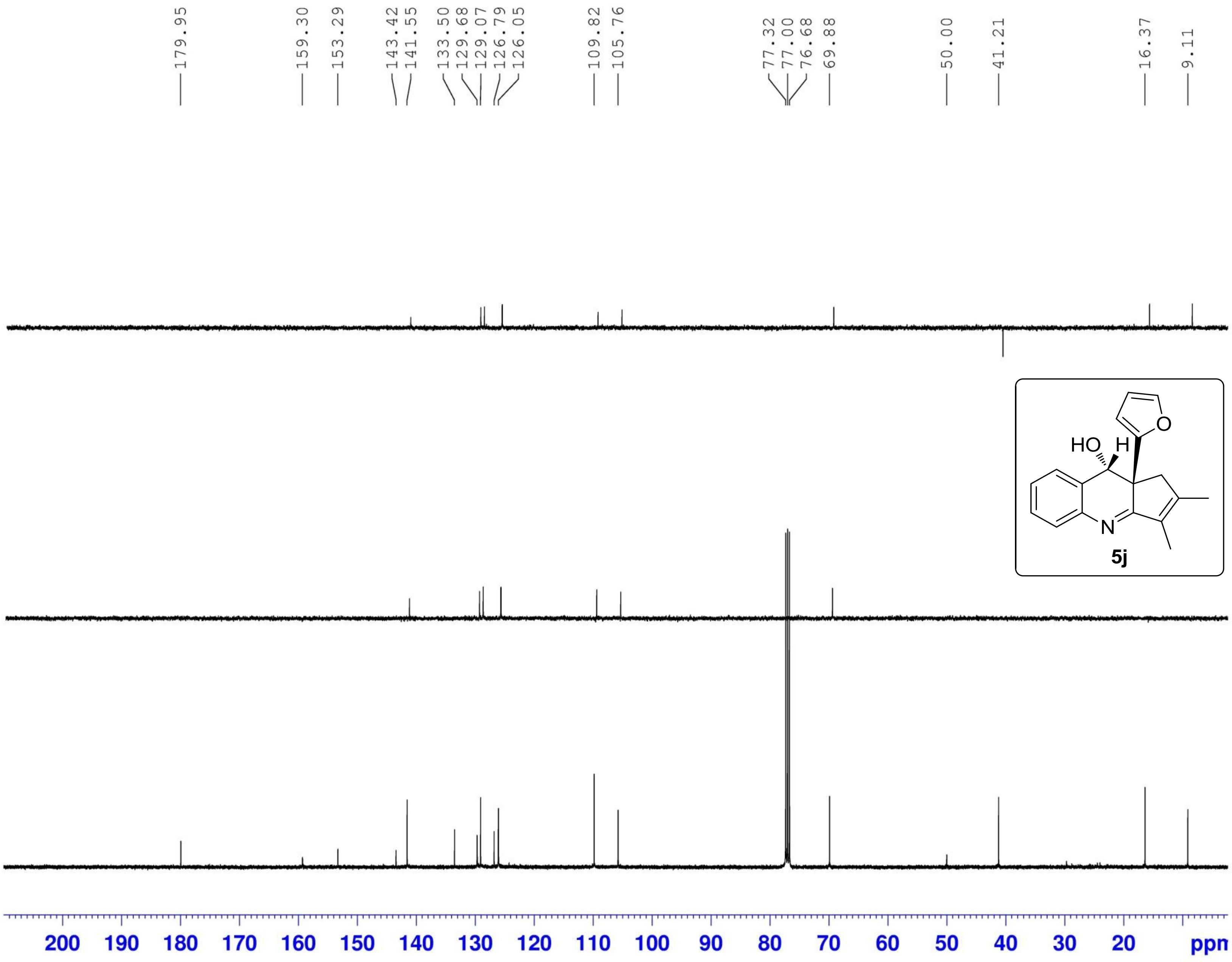
179.95
159.30
153.29
143.42
141.55
133.50
129.68
129.07
126.79
126.05
109.82
105.76
77.32
77.00
76.68
69.88
50.00
41.21
16.37
9.11
HO
H
O
N
5j
200 190 180 170 160 150 140 130 120 110 100 90 80 70 60 50 40 30 20 ppm

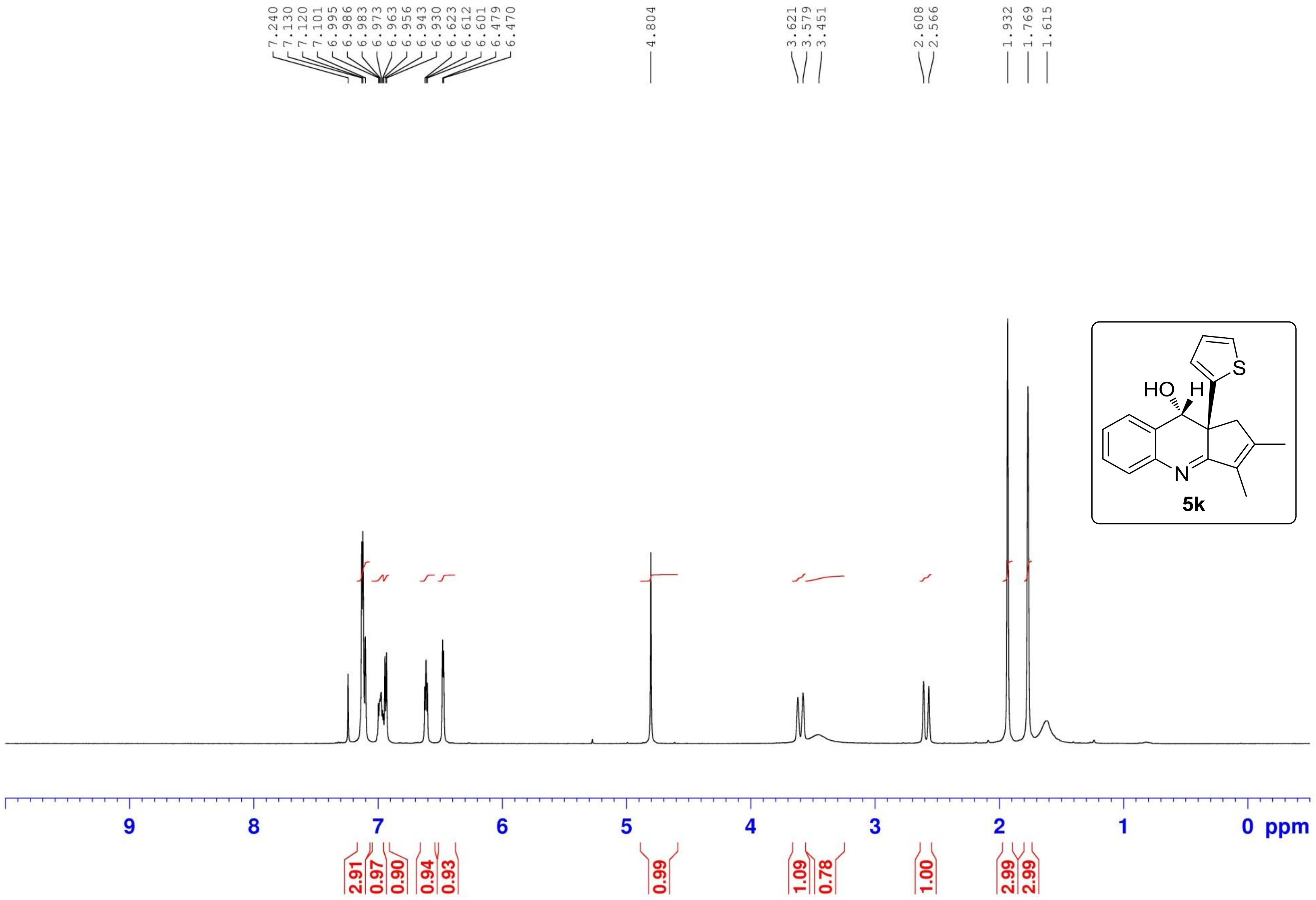

7.240
7.130
7.120
7.101
6.995
6.986
6.983
6.973
6.963
6.956
6.943
6.930
6.623
6.612
6.601
6.479
6.470
4.804
3.621
3.579
3.451
2.608
2.566
1.932
1.769
1.615
HO
H
S
N
5k
9
8
7
6
5
4
3
2
1
0 ppm
2.91
0.97
0.90
0.94
0.93
0.99
1.09
0.78
1.00
2.99
2.99

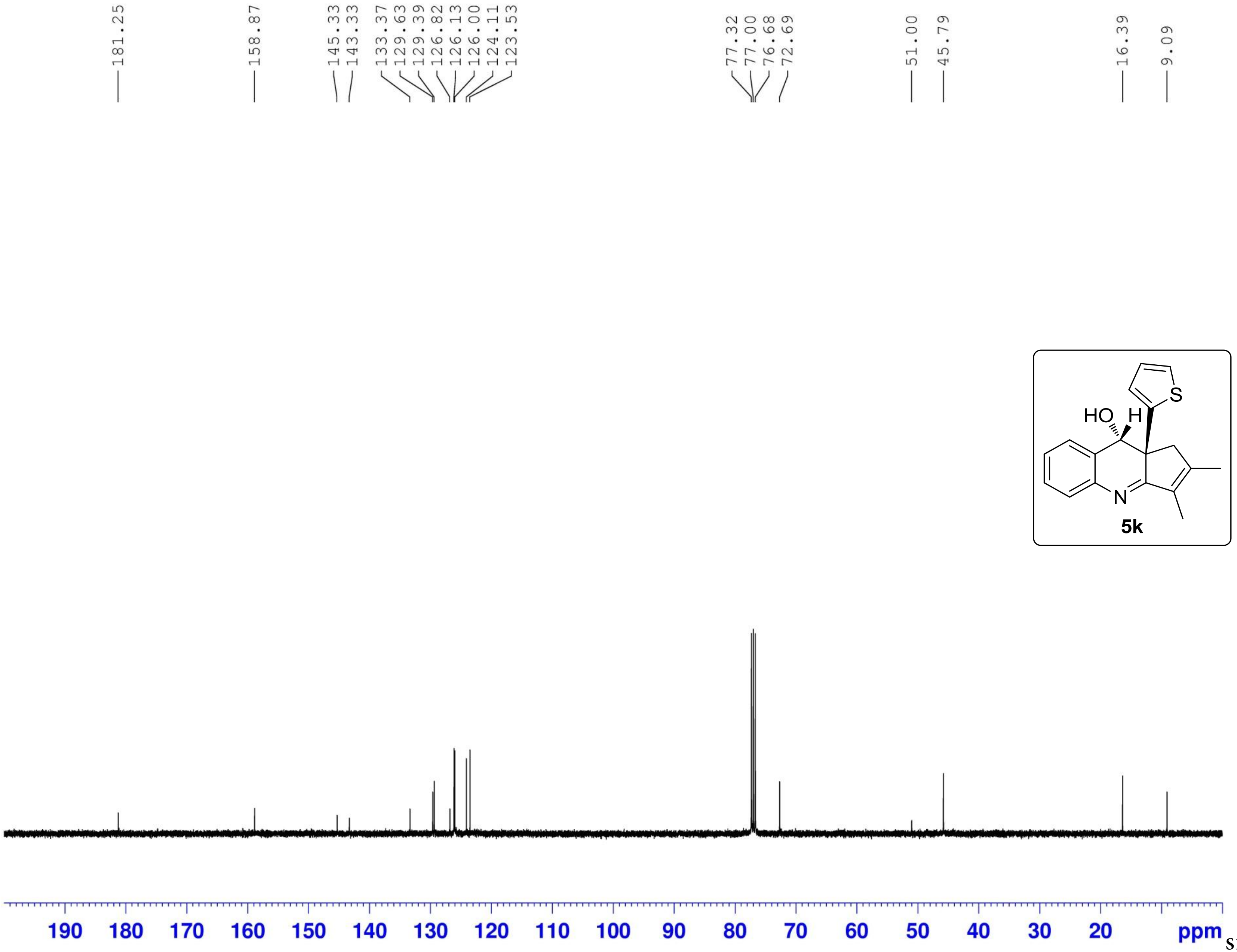
181.25
158.87
145.33
143.33
133.37
129.63
129.39
126.82
126.13
126.00
124.11
123.53
77.32
77.00
76.68
72.69
51.00
45.79
16.39
9.09
190 180 170 160 150 140 130 120 110 100 90 80 70 60 50 40 30 20 ppm
S
HO
H
N

5k

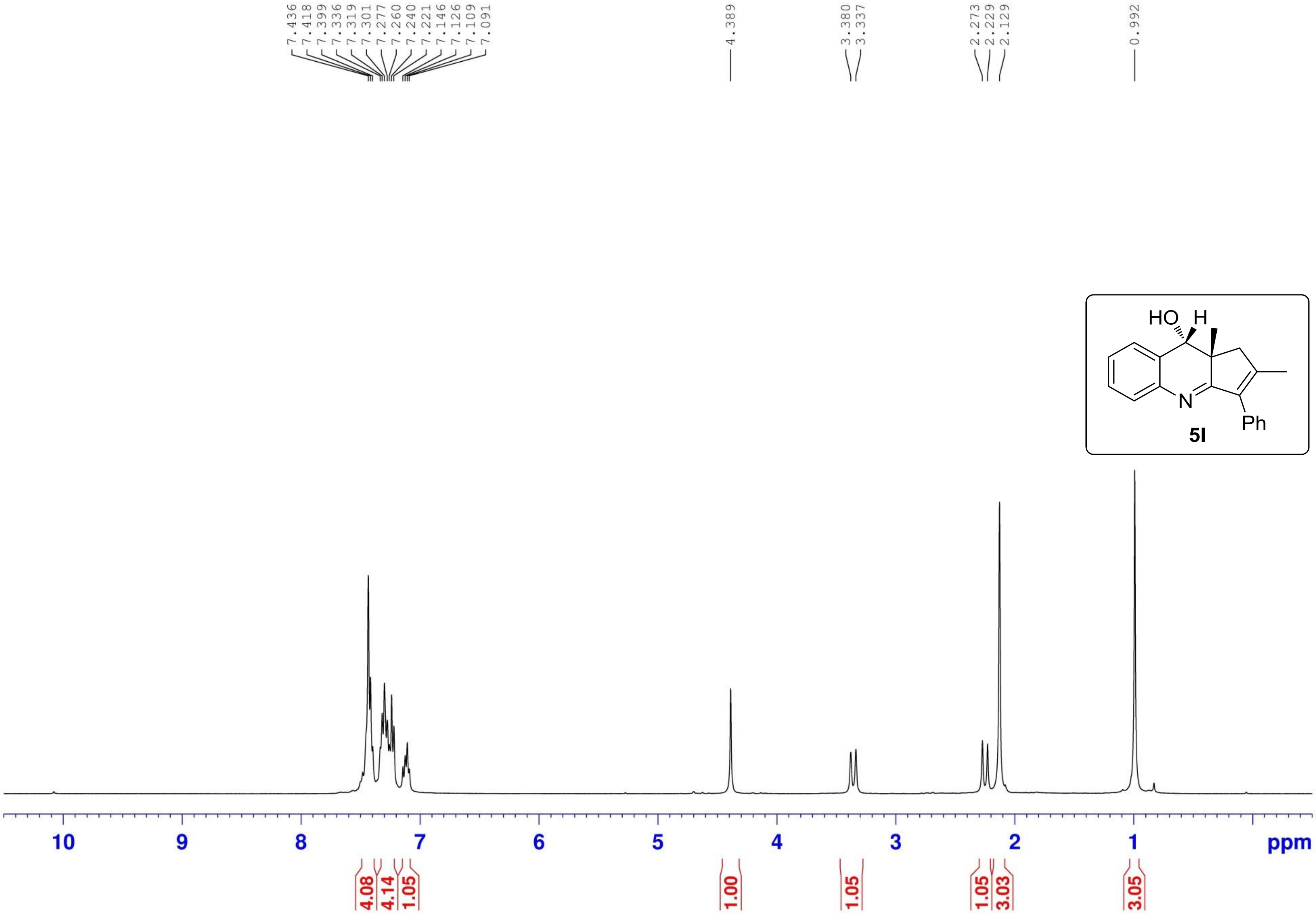

7.436
7.418
7.399
7.336
7.319
7.301
7.277
7.260
7.240
7.221
7.146
7.126
7.109
7.091
4.389
3.380
3.337
2.273
2.229
2.129
0.992
HO
H
N
Ph
5l
10
9
8
7
6
5
4
3
2
1
ppm
4.08
4.14
1.05
1.00
1.05
1.05
3.03
3.05

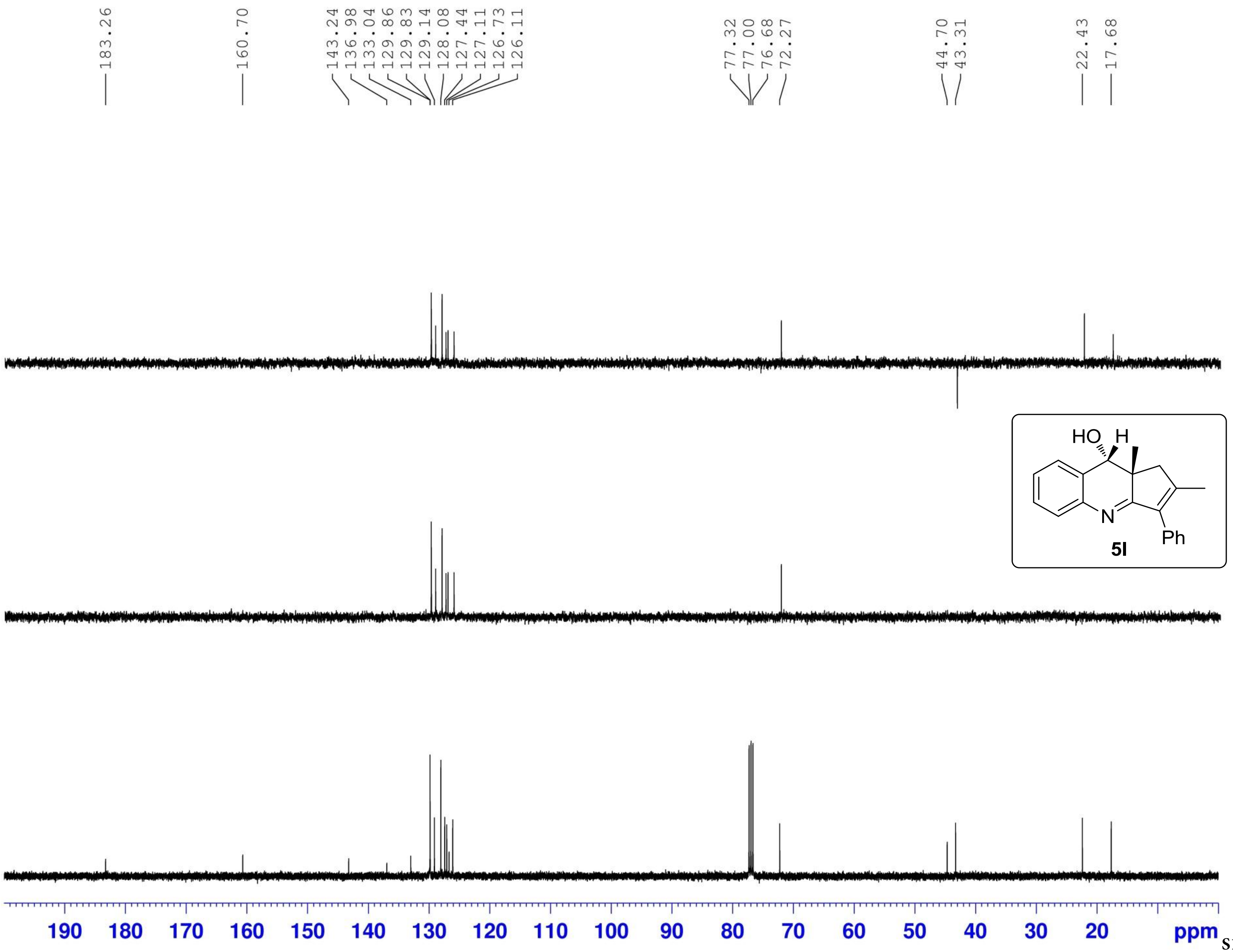

183.26
160.70
143.24
136.98
133.04
129.86
129.83
129.14
128.08
127.44
127.11
126.73
126.11
77.32
77.00
76.68
72.27
44.70
43.31
22.43
17.68
HO
H
N
Ph
5l
190
180
170
160
150
140
130
120
110
100
90
80
70
60
50
40
30
20
ppm

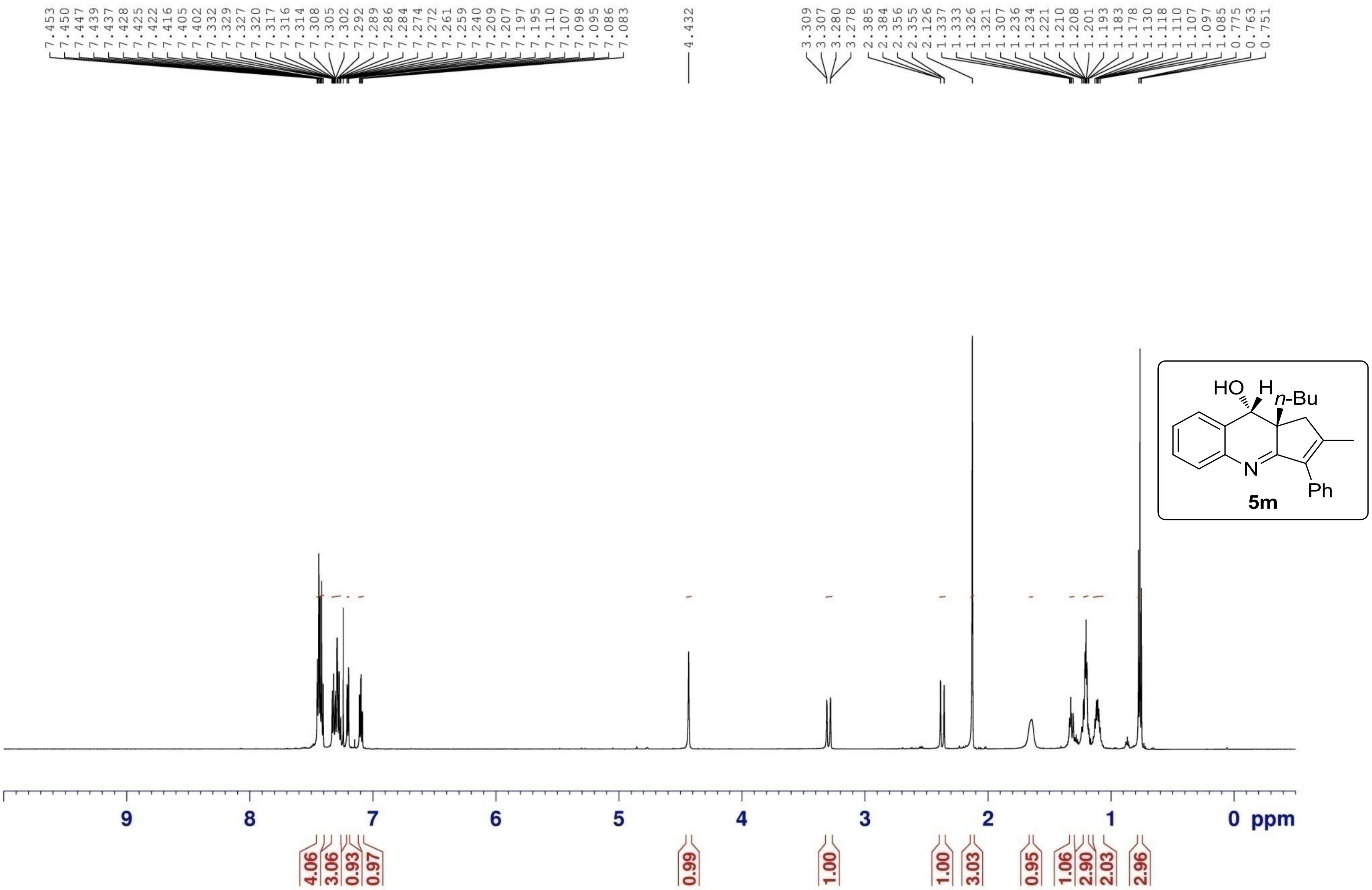
HO
H
n-Bu
N
Ph
5m
ppm

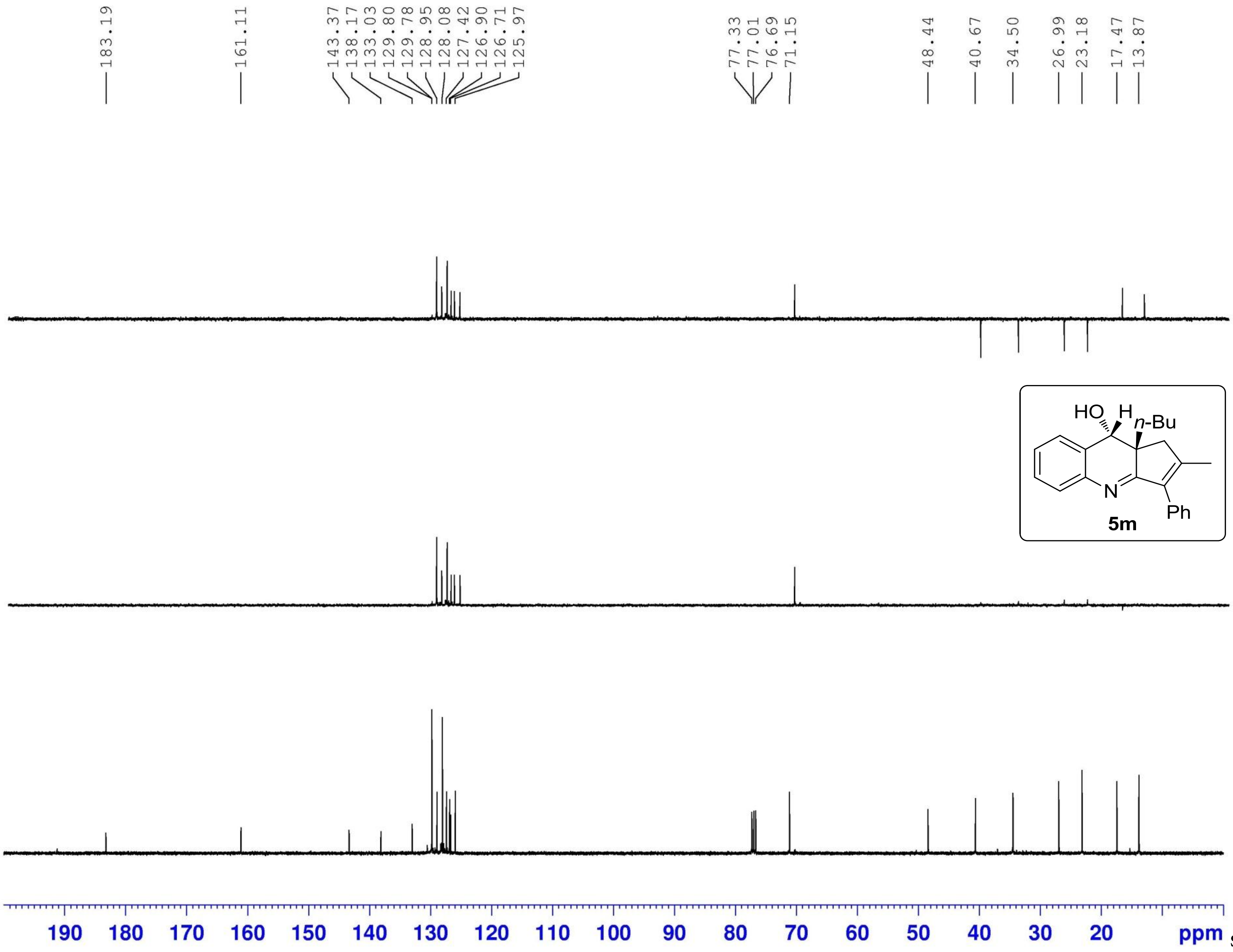
183.19
161.11
143.37
138.17
133.03
129.80
129.78
128.95
128.08
127.42
126.90
126.71
125.97
77.33
77.01
76.69
71.15
48.44
40.67
34.50
26.99
23.18
17.47
13.87
HO
H
n-Bu
N
Ph
5m
190
180
170
160
150
140
130
120
110
100
90
80
70
60
50
40
30
20
ppm

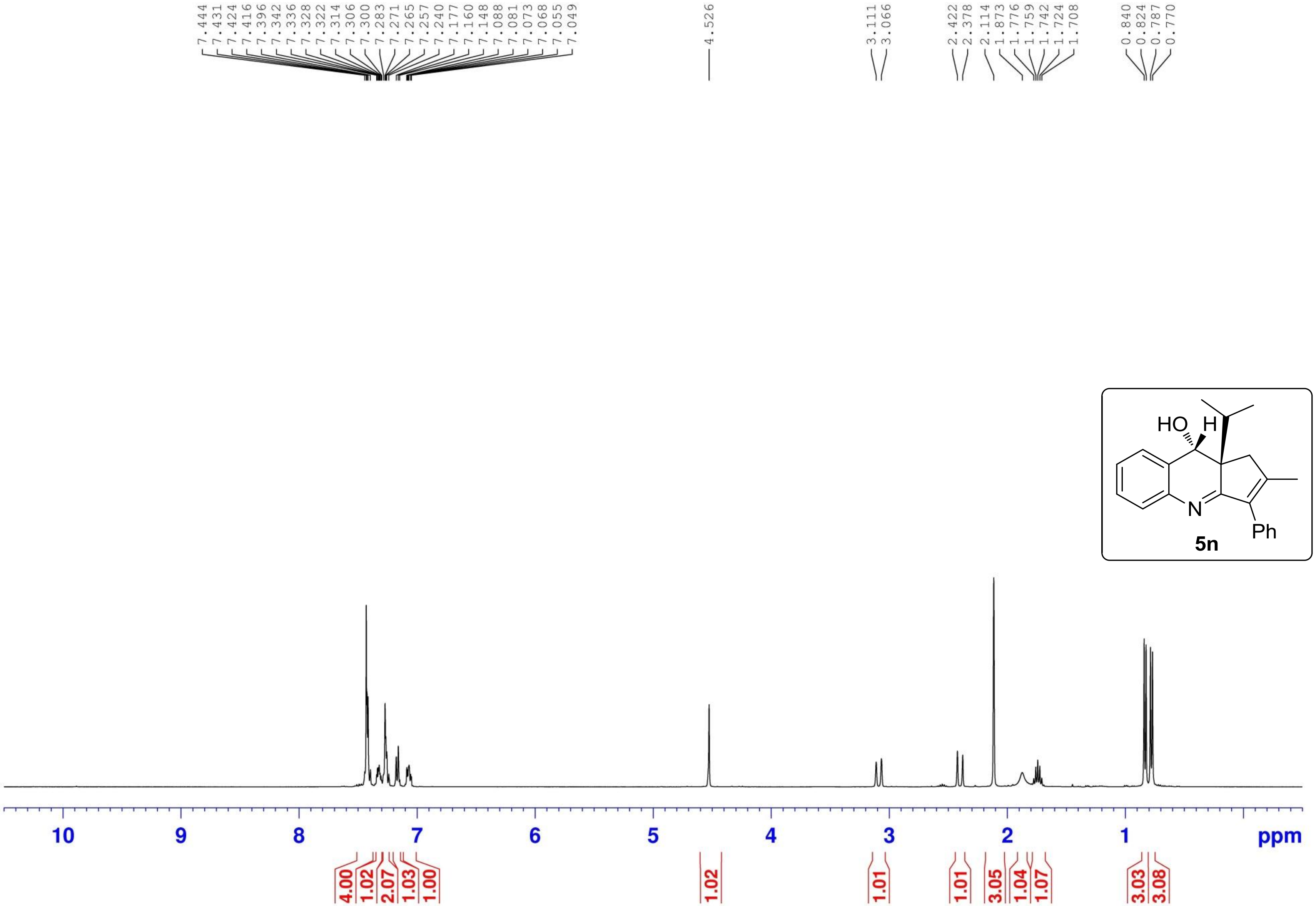
7.444 7.431 7.424 7.416 7.396 7.342 7.336 7.328 7.322 7.314 7.306 7.300 7.283 7.271 7.265 7.257 7.240 7.177 7.160 7.148 7.088 7.081 7.073 7.068 7.055 7.049
4.526
3.111 3.066
2.422 2.378 2.114 1.873 1.776 1.759 1.742 1.724 1.708
0.840 0.824 0.787 0.770
HO
H
N
Ph
5n
10 9 8 7 6 5 4 3 2 1 ppm
4.00 1.02 2.07 1.03 1.00
1.02
1.01
1.01 3.05 1.04 1.07
3.03 3.08

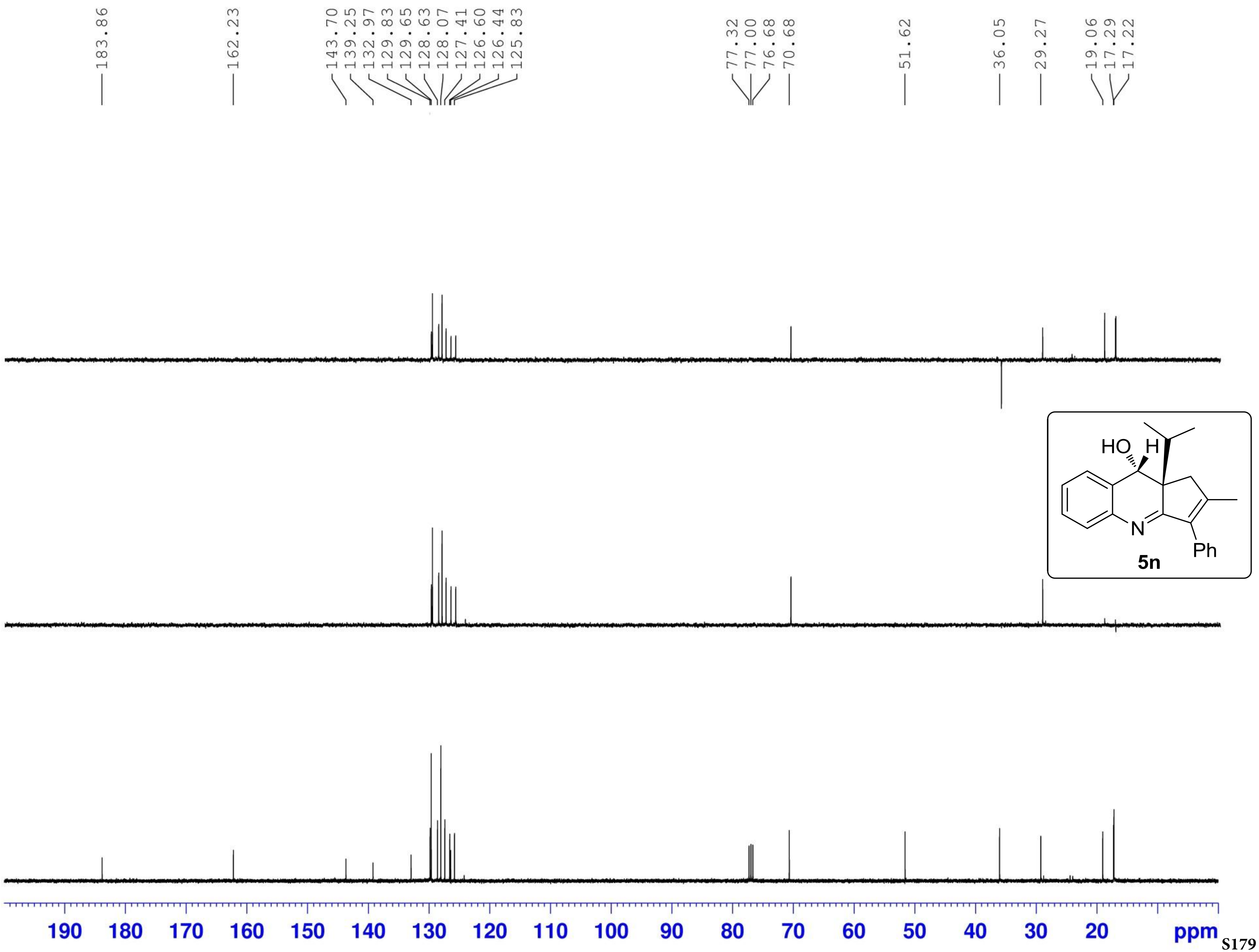
183.86
162.23
143.70
139.25
132.97
129.83
129.65
128.63
128.07
127.41
126.60
126.44
125.83
77.32
77.00
76.68
70.68
51.62
36.05
29.27
19.06
17.29
17.22
HO
H
N
Ph
5n
190
180
170
160
150
140
130
120
110
100
90
80
70
60
50
40
30
20
ppm

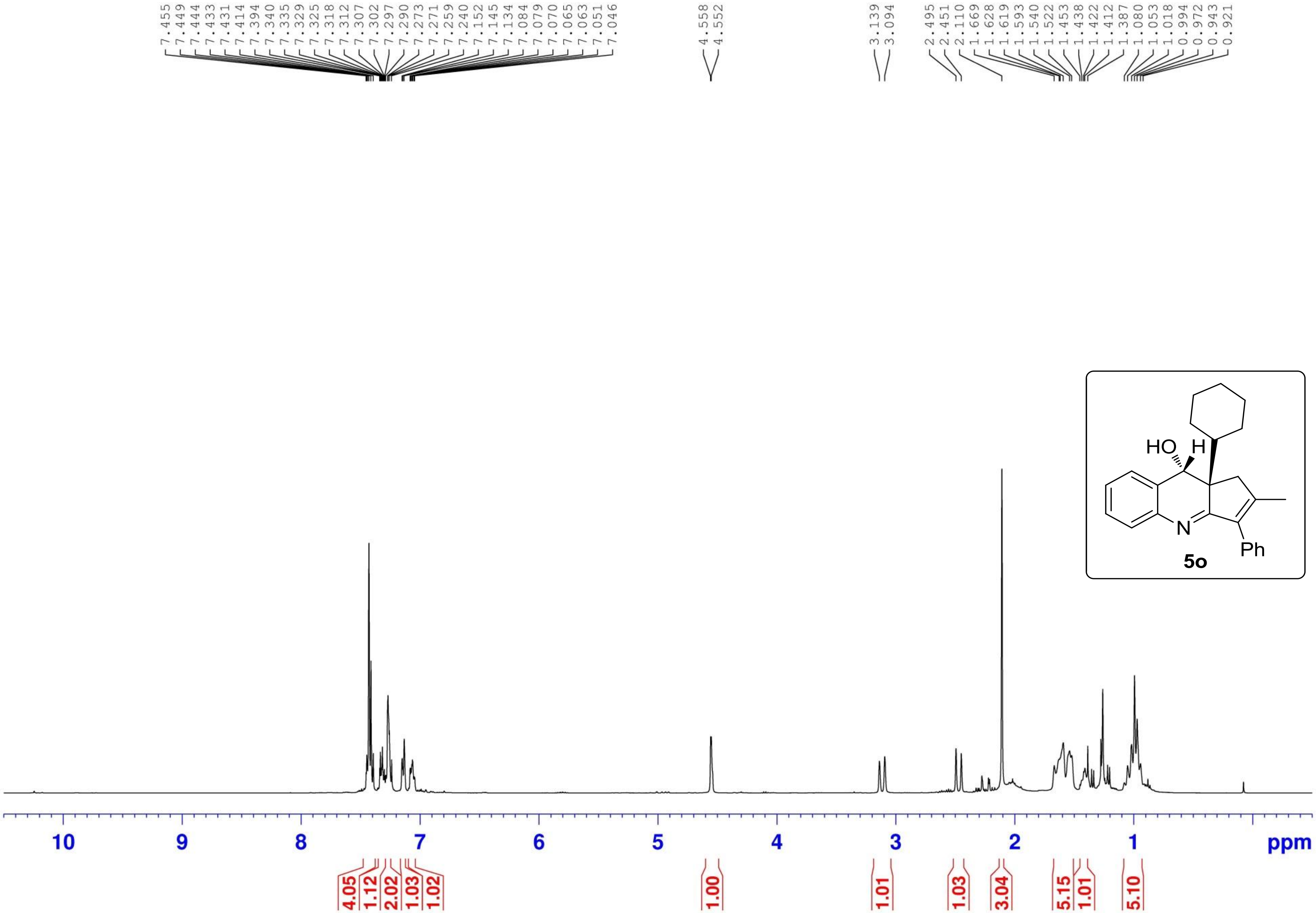
7.455
7.449
7.444
7.433
7.431
7.414
7.394
7.340
7.335
7.329
7.325
7.318
7.312
7.307
7.302
7.297
7.290
7.273
7.271
7.259
7.240
7.152
7.145
7.134
7.084
7.079
7.070
7.065
7.063
7.051
7.046
4.558
4.552
3.139
3.094
2.495
2.451
2.110
1.669
1.628
1.619
1.593
1.540
1.522
1.453
1.438
1.422
1.412
1.387
1.080
1.053
1.018
0.994
0.972
0.943
0.921
HO
H
N
Ph
5o
10
9
8
7
6
5
4
3
2
1
ppm
4.05
1.12
2.02
1.03
1.02
1.00
1.01
1.03
3.04
5.15
1.01
5.10

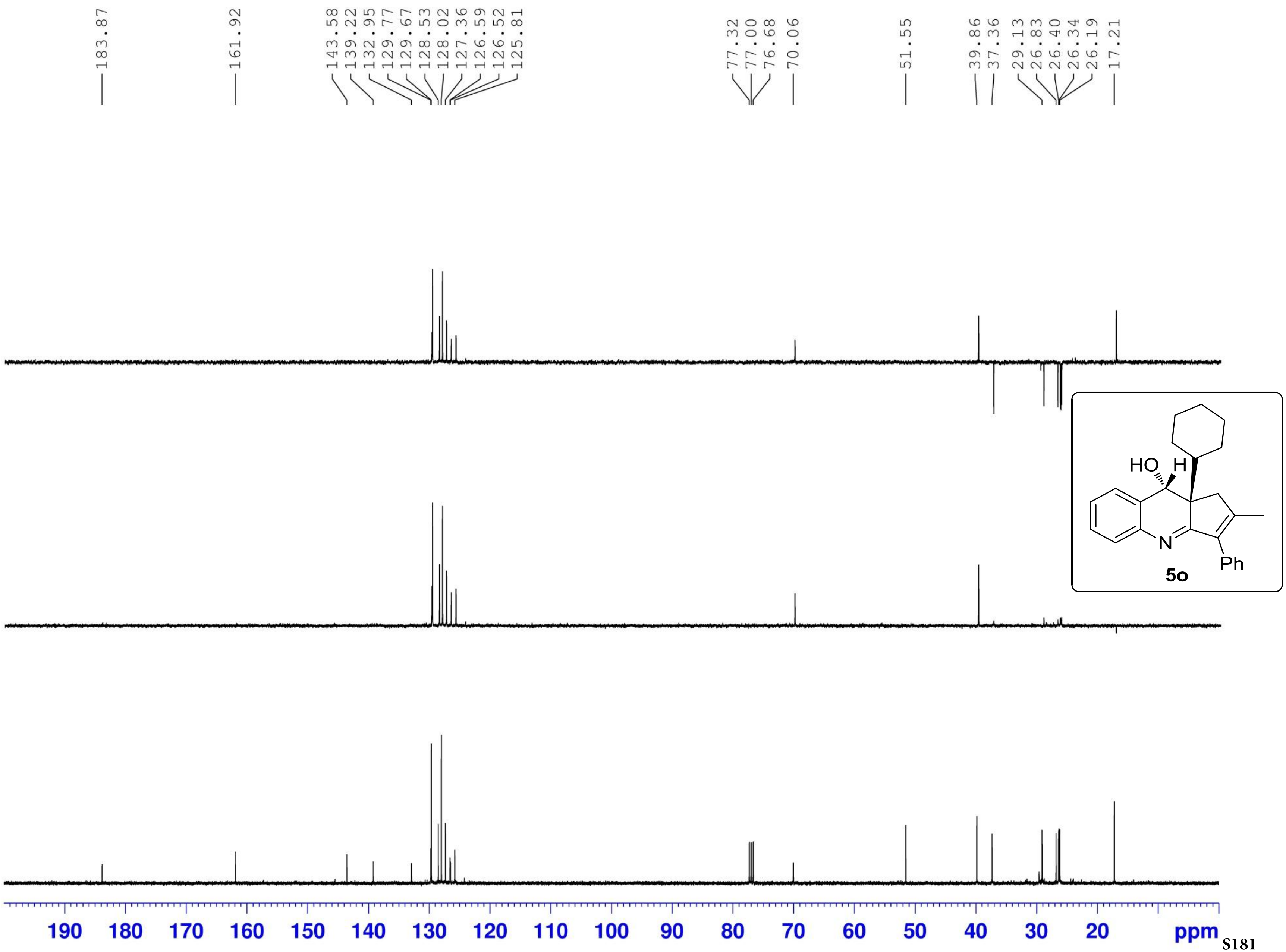
183.87
161.92
143.58
139.22
132.95
129.77
129.67
128.53
128.02
127.36
126.59
126.52
125.81
77.32
77.00
76.68
70.06
51.55
39.86
37.36
29.13
26.83
26.40
26.34
26.19
17.21
HO
H
N
Ph
5o
190 180 170 160 150 140 130 120 110 100 90 80 70 60 50 40 30 20
ppm

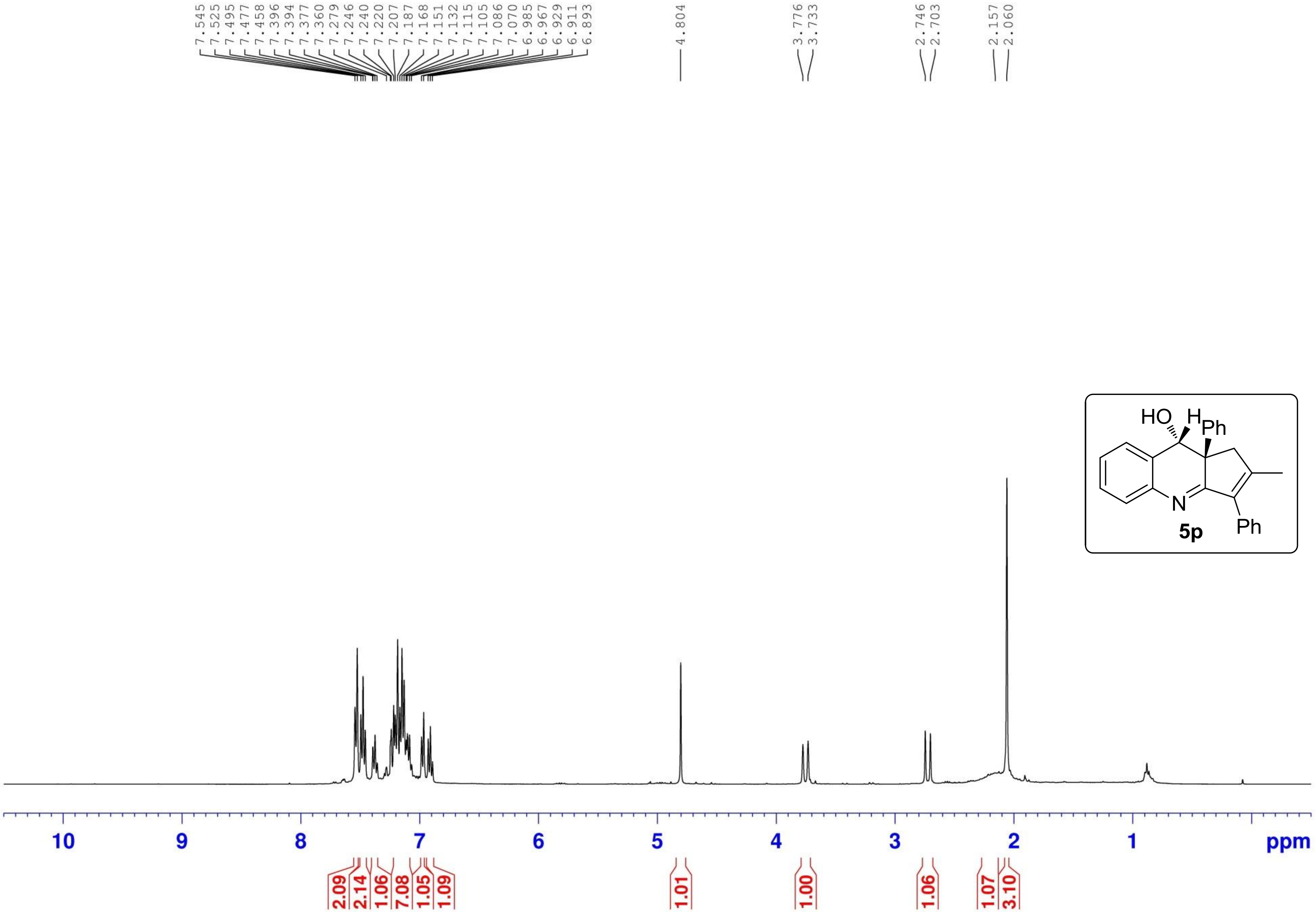
7.545
7.525
7.495
7.477
7.458
7.396
7.394
7.377
7.360
7.279
7.246
7.240
7.220
7.207
7.187
7.168
7.151
7.132
7.115
7.105
7.086
7.070
6.985
6.967
6.929
6.911
6.893
4.804
3.776
3.733
2.746
2.703
2.157
2.060
HO
H
Ph
N
Ph
5p
10
9
8
7
6
5
4
3
2
1
ppm
2.09
2.14
1.06
7.08
1.05
1.09
1.01
1.00
1.06
1.07
3.10

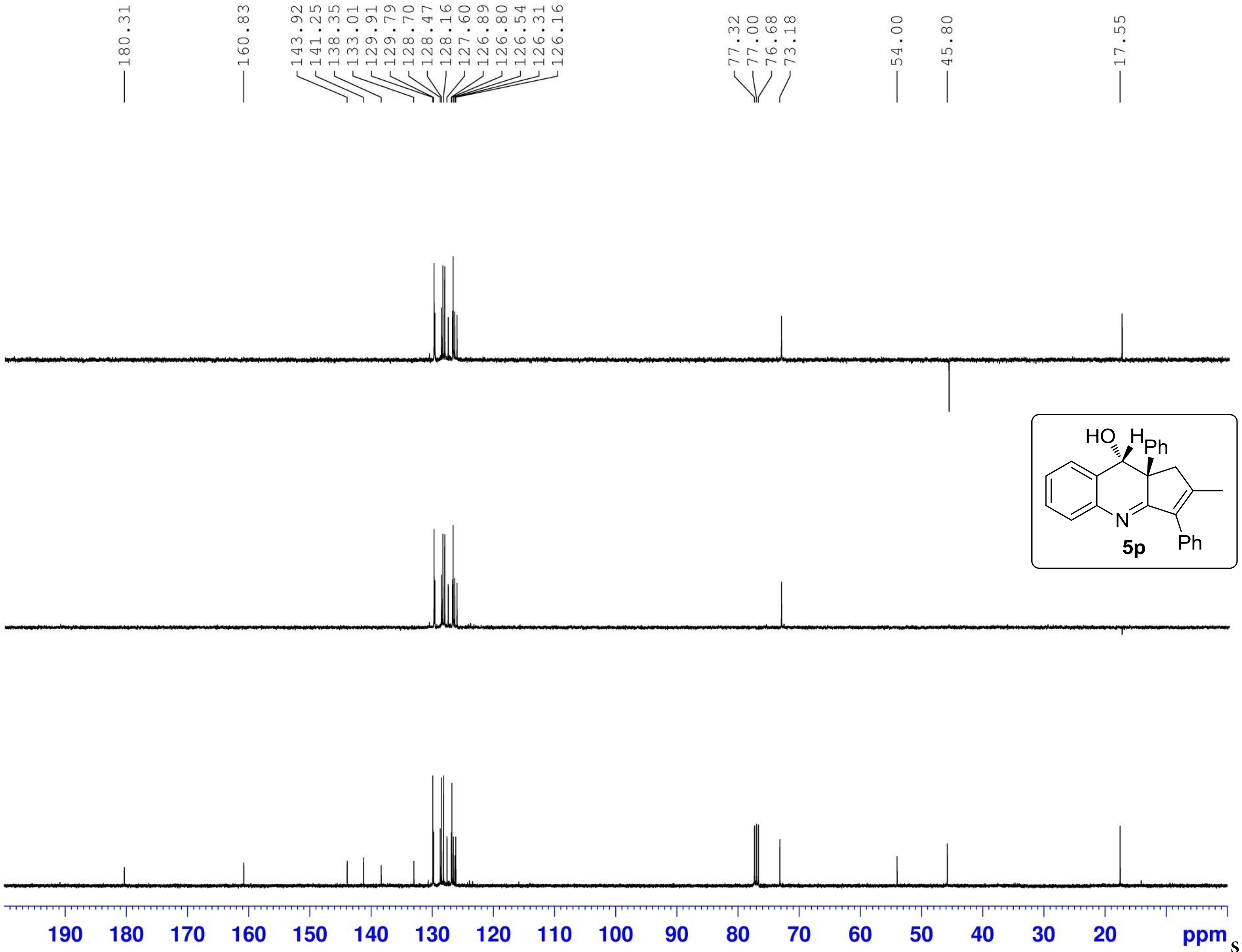
180.31
160.83
143.92
141.25
138.35
133.01
129.91
129.79
128.70
128.47
128.16
127.60
126.89
126.80
126.54
126.31
126.16
77.32
77.00
76.68
73.18
54.00
45.80
17.55
HO
H
Ph
N
Ph
5p
190 180 170 160 150 140 130 120 110 100 90 80 70 60 50 40 30 20 ppm

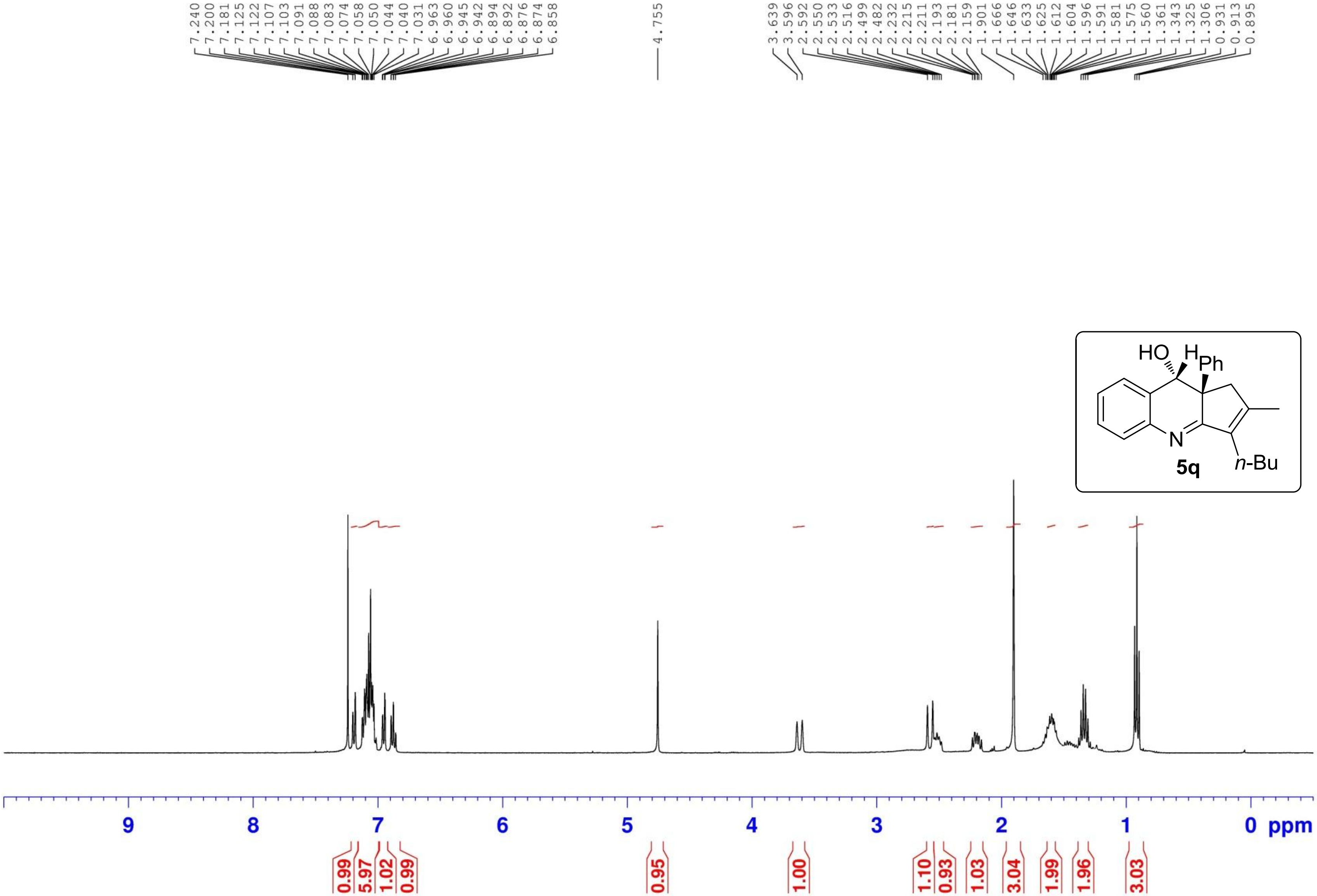
7.240
7.200
7.181
7.125
7.122
7.107
7.103
7.091
7.088
7.083
7.074
7.058
7.050
7.044
7.040
7.031
6.963
6.960
6.945
6.942
6.894
6.892
6.876
6.874
6.858
4.755
3.639
3.596
2.592
2.550
2.533
2.516
2.499
2.482
2.232
2.215
2.211
2.193
2.181
2.159
1.901
1.666
1.646
1.633
1.625
1.612
1.604
1.596
1.591
1.581
1.575
1.560
1.361
1.343
1.325
1.306
0.931
0.913
0.895
9
8
7
6
5
4
3
2
1
0 ppm
0.99
5.97
1.02
0.99
0.95
1.00
1.10
0.93
1.03
3.04
1.99
1.96
3.03
HO
H
Ph
N
n-Bu

5q

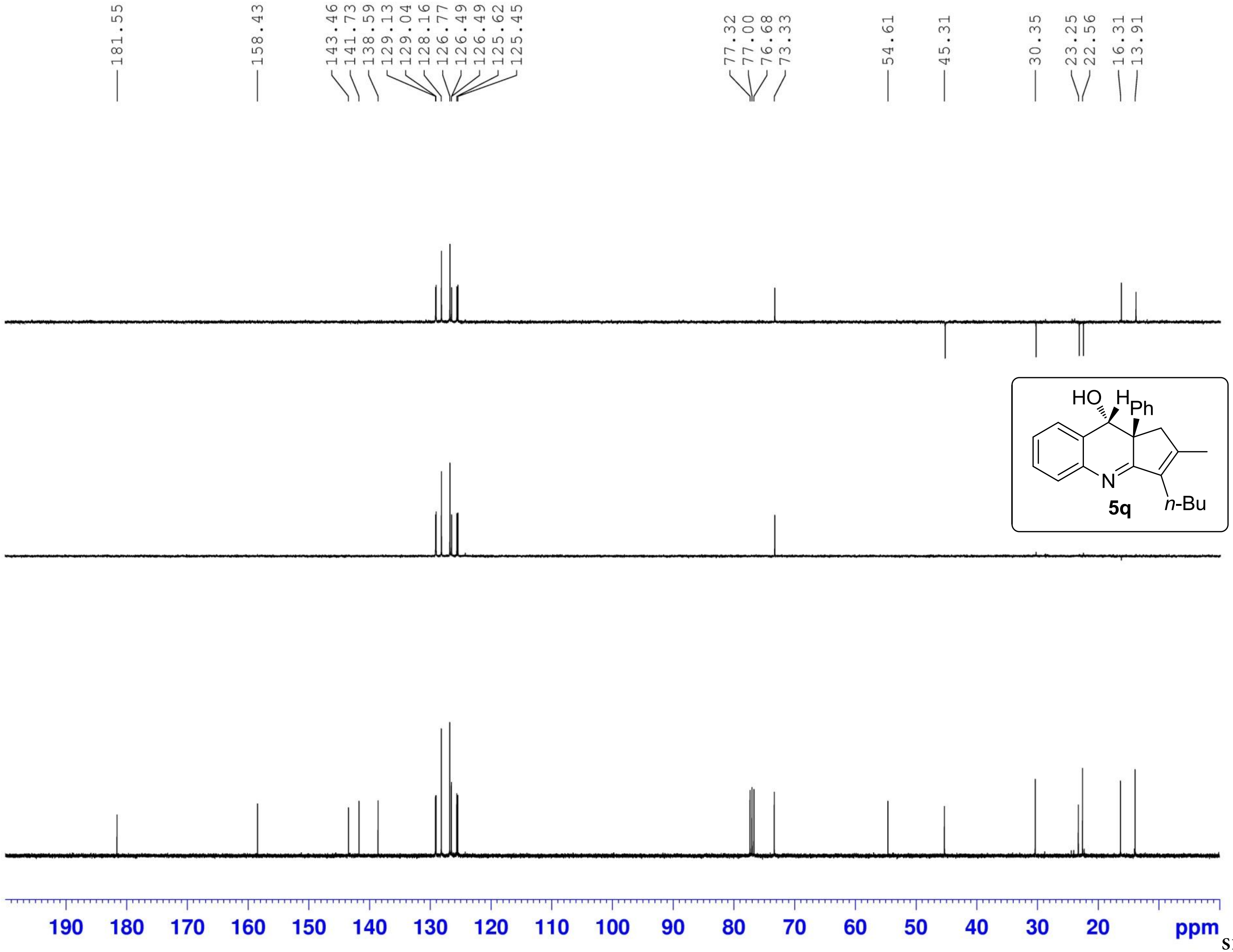
181.55
158.43
143.46
141.73
138.59
129.13
129.04
128.16
126.77
126.49
126.49
125.62
125.45
77.32
77.00
76.68
73.33
54.61
45.31
30.35
23.25
22.56
16.31
13.91
HO
H
Ph
N
n-Bu
5q
190 180 170 160 150 140 130 120 110 100 90 80 70 60 50 40 30 20 ppm

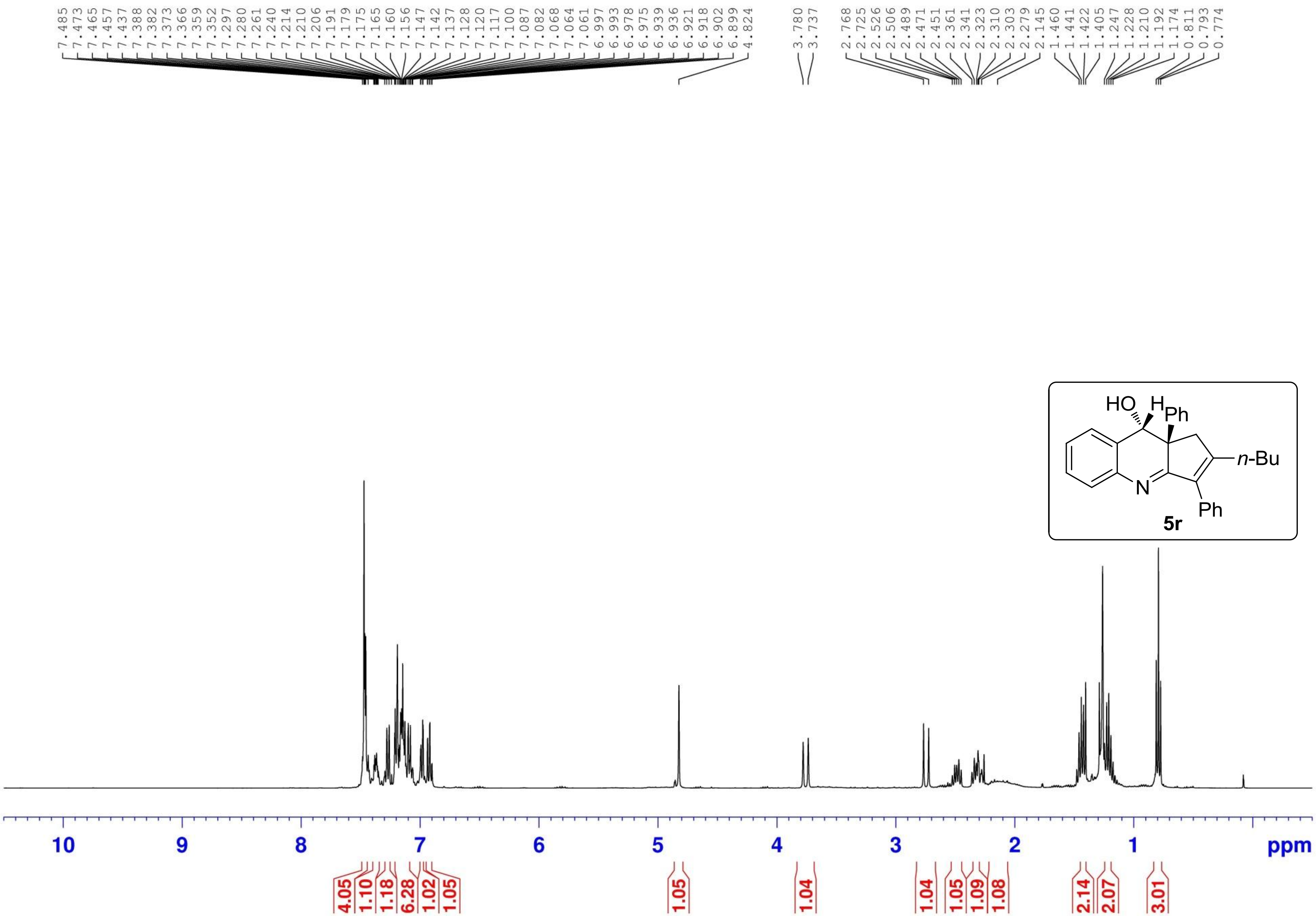
7.485
7.473
7.465
7.457
7.437
7.388
7.382
7.373
7.366
7.359
7.352
7.297
7.280
7.261
7.240
7.214
7.210
7.206
7.191
7.179
7.175
7.165
7.160
7.156
7.147
7.142
7.137
7.128
7.120
7.117
7.100
7.087
7.082
7.068
7.064
7.061
6.997
6.993
6.978
6.975
6.939
6.936
6.921
6.918
6.902
6.899
4.824
3.780
3.737
2.768
2.725
2.526
2.506
2.489
2.471
2.451
2.361
2.341
2.323
2.310
2.303
2.279
2.145
1.460
1.441
1.422
1.405
1.247
1.228
1.210
1.192
1.174
0.811
0.793
0.774
HO
H
Ph
n-Bu
N
Ph
5r
10
9
8
7
6
5
4
3
2
1
ppm
4.05
1.10
1.18
6.28
1.02
1.05
1.05
1.04
1.04
1.05
1.09
1.08
2.14
2.07
3.01

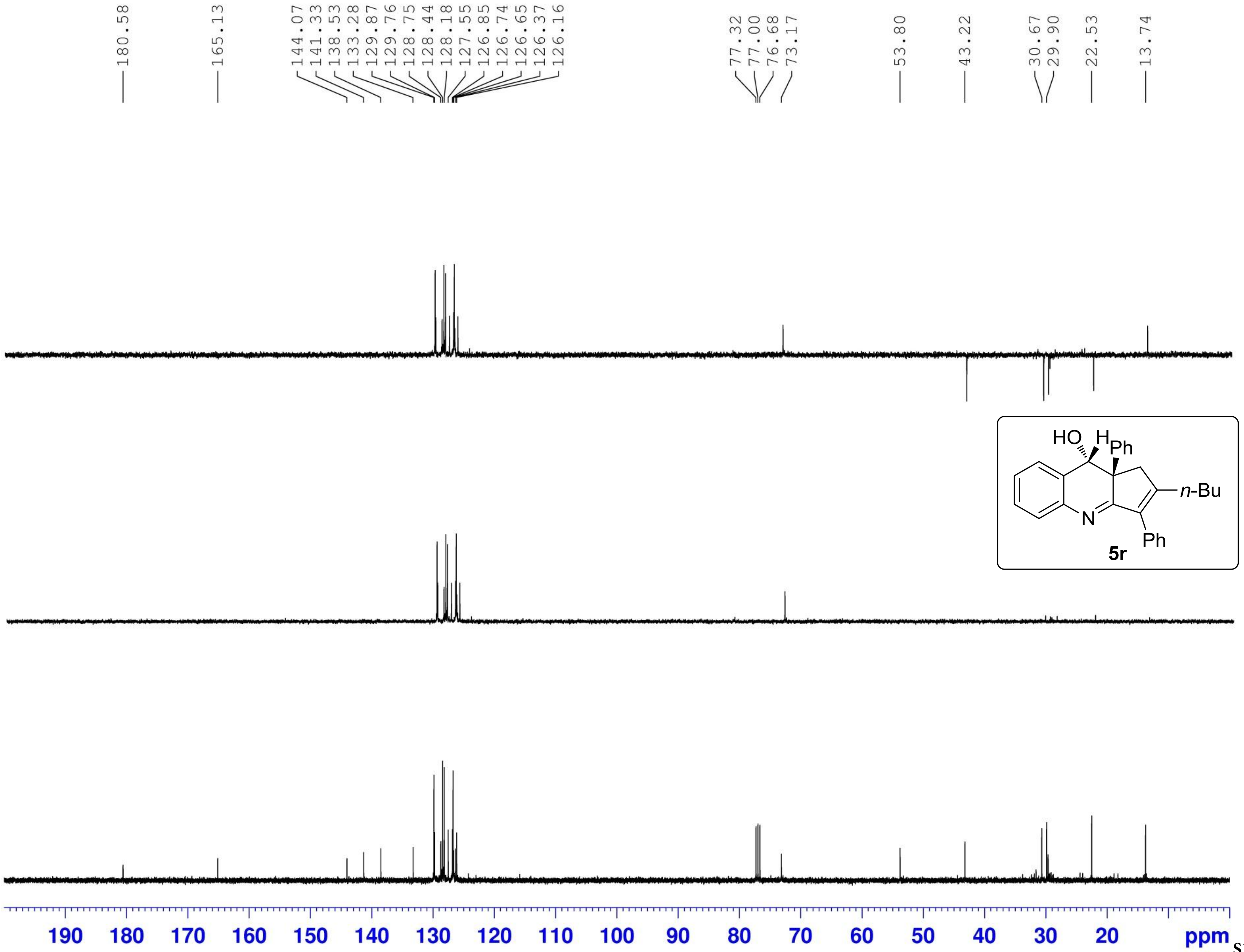
180.58
165.13
144.07
141.33
138.53
133.28
129.87
129.76
128.75
128.44
128.18
127.55
126.85
126.74
126.65
126.37
126.16
77.32
77.00
76.68
73.17
53.80
43.22
30.67
29.90
22.53
13.74
HO
H
Ph
n-Bu
N
Ph
5r
190 180 170 160 150 140 130 120 110 100 90 80 70 60 50 40 30 20 ppm

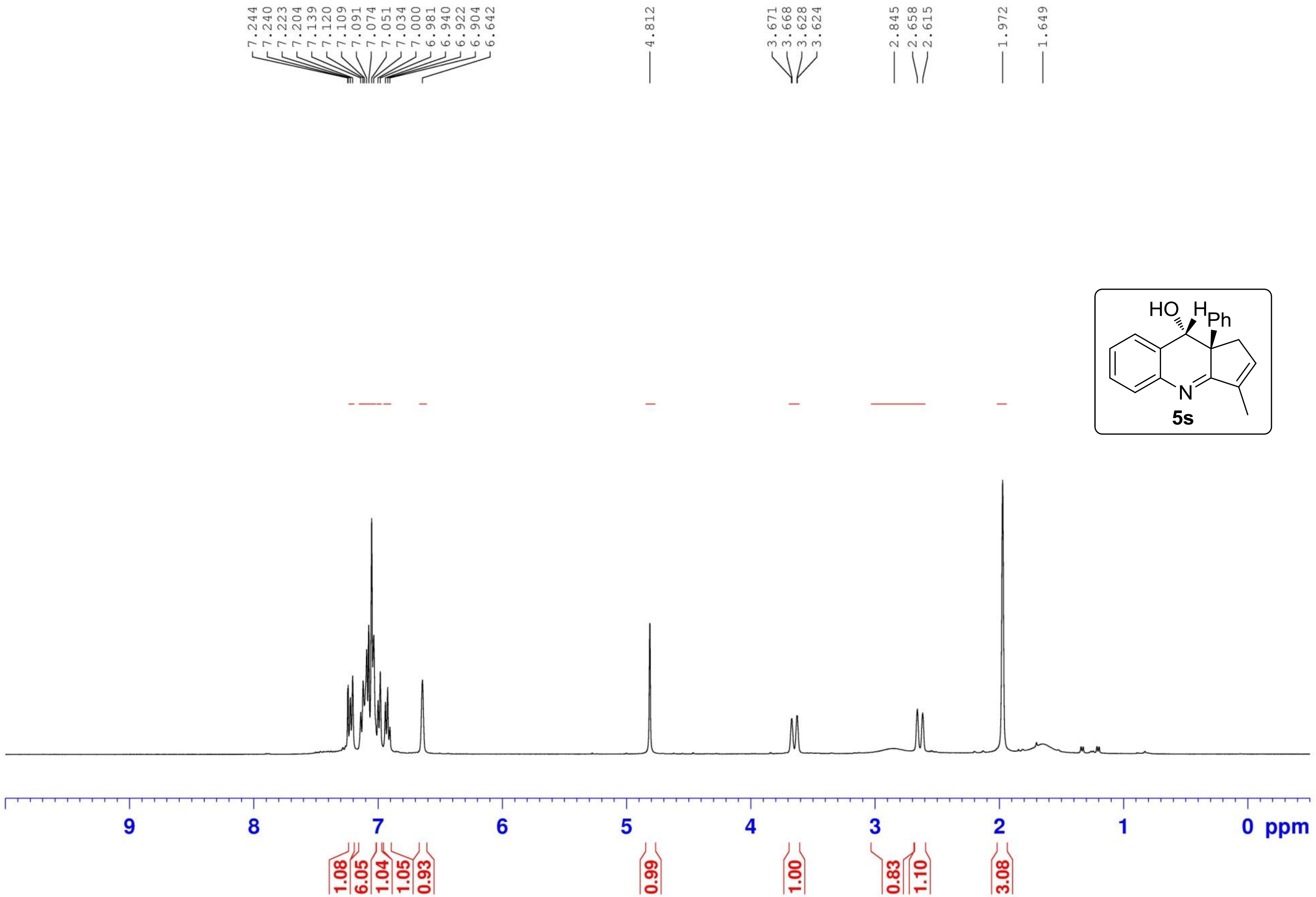
7.244
7.240
7.223
7.204
7.139
7.120
7.109
7.091
7.074
7.051
7.034
7.000
6.981
6.940
6.922
6.904
6.642
4.812
3.671
3.668
3.628
3.624
2.845
2.658
2.615
1.972
1.649
HO
H
Ph
N
5s
9
8
7
6
5
4
3
2
1
0 ppm
1.08
6.05
1.04
1.05
0.93
0.99
1.00
0.83
1.10
3.08

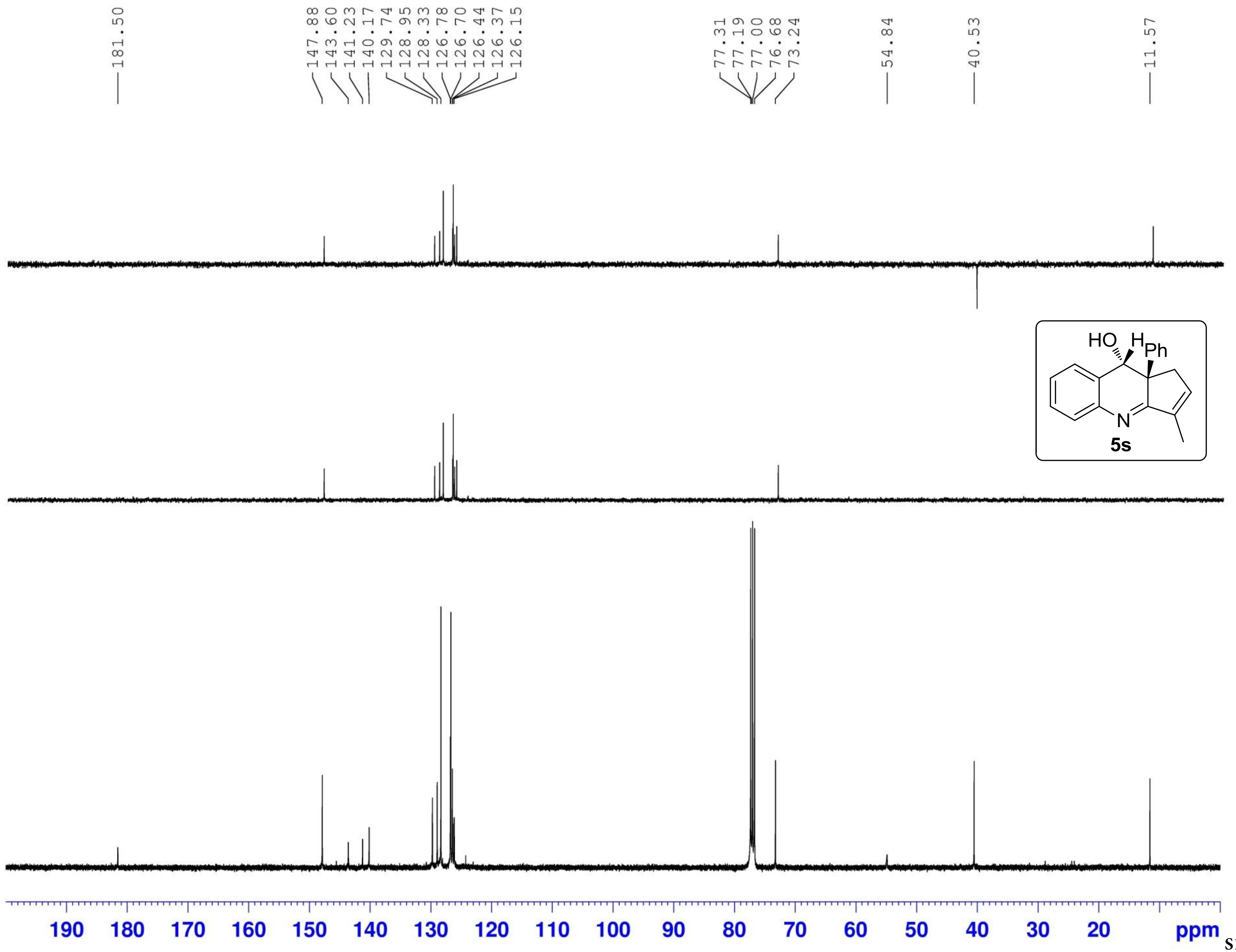
181.50
147.88
143.60
141.23
140.17
129.74
128.95
128.33
126.78
126.70
126.44
126.37
126.15
77.31
77.19
77.00
76.68
73.24
54.84
40.53
11.57
HO
H
Ph
N
5s
190 180 170 160 150 140 130 120 110 100 90 80 70 60 50 40 30 20 ppm

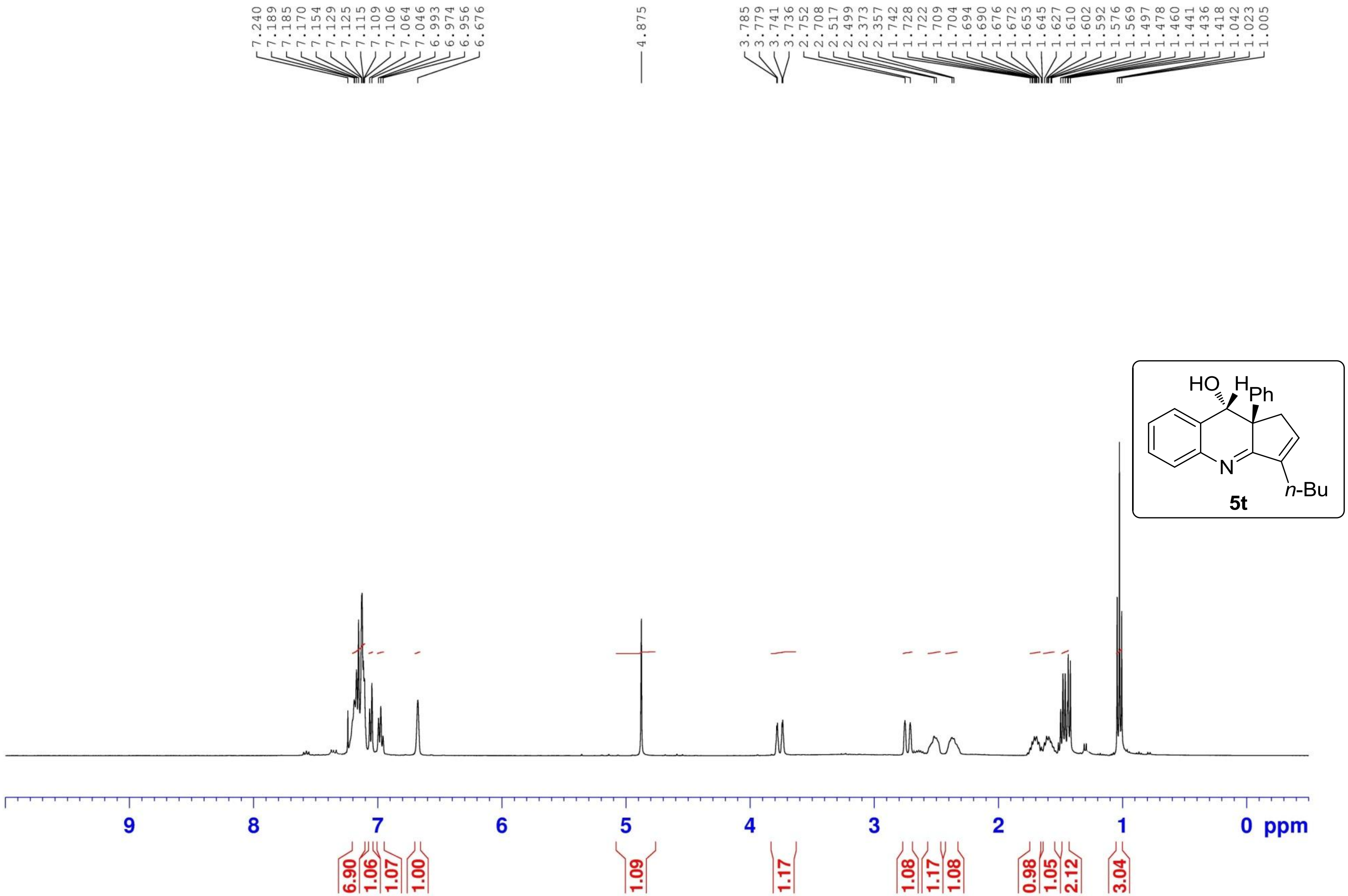
7.240
7.189
7.185
7.170
7.154
7.129
7.125
7.115
7.109
7.106
7.064
7.046
6.993
6.974
6.956
6.676
4.875
3.785
3.779
3.741
3.736
2.752
2.708
2.517
2.499
2.373
2.357
1.742
1.728
1.722
1.709
1.704
1.694
1.690
1.676
1.672
1.653
1.645
1.627
1.610
1.602
1.592
1.576
1.569
1.497
1.478
1.460
1.441
1.436
1.418
1.042
1.023
1.005
HO
H
Ph
N
n-Bu
5t
9
8
7
6
5
4
3
2
1
0 ppm
6.90
1.06
1.07
1.00
1.09
1.17
1.08
1.17
1.08
0.98
1.05
2.12
3.04

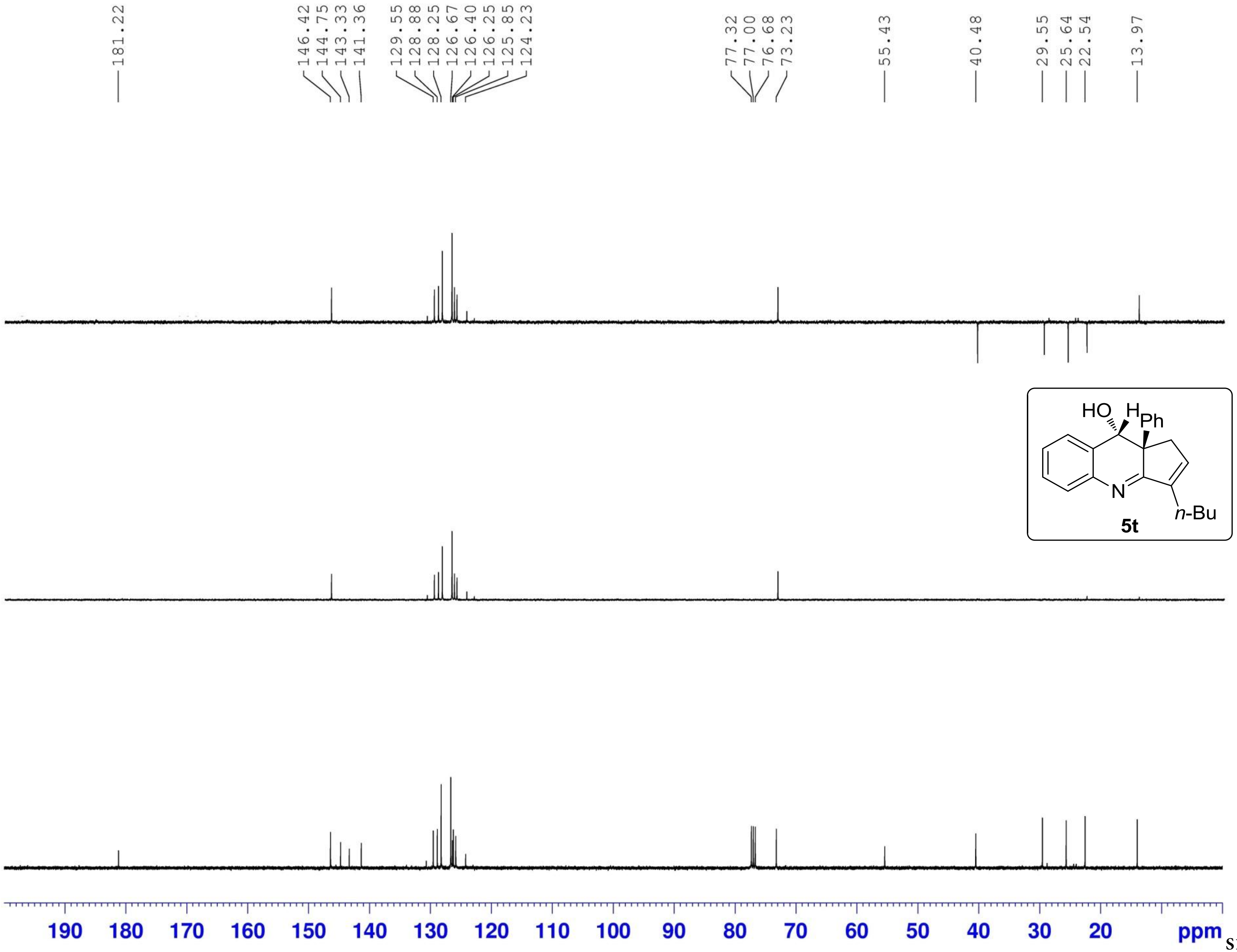
181.22
146.42
144.75
143.33
141.36
129.55
128.88
128.25
126.67
126.40
126.25
125.85
124.23
77.32
77.00
76.68
73.23
55.43
40.48
29.55
25.64
22.54
13.97
HO
H
Ph
N
n-Bu
5t
190
180
170
160
150
140
130
120
110
100
90
80
70
60
50
40
30
20
ppm

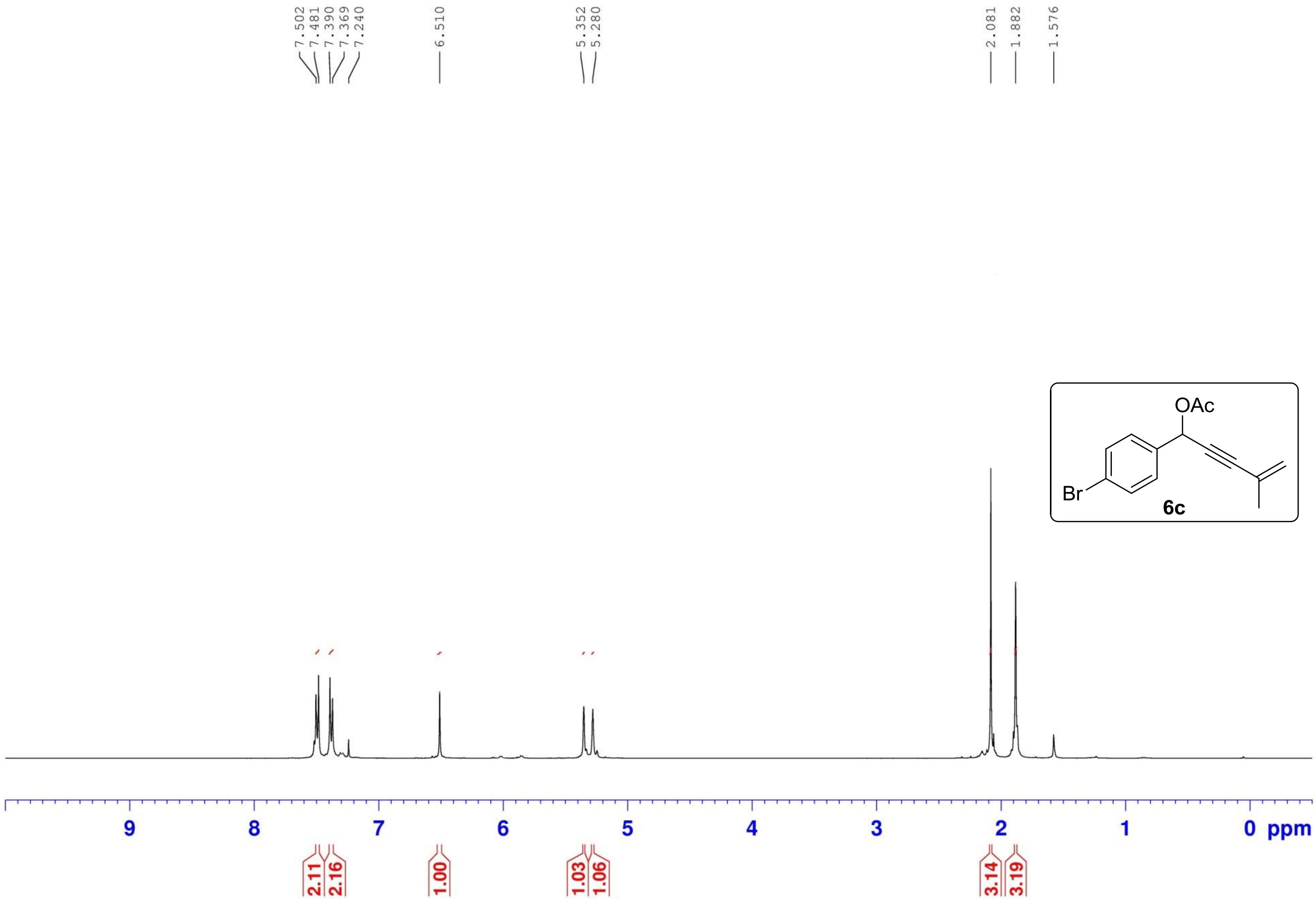
7.502
7.481
7.390
7.369
7.240
6.510
5.352
5.280
2.081
1.882
1.576
OAc
Br
6c
9
8
7
6
5
4
3
2
1
0 ppm
2.11
2.16
1.00
1.03
1.06
3.14
3.19

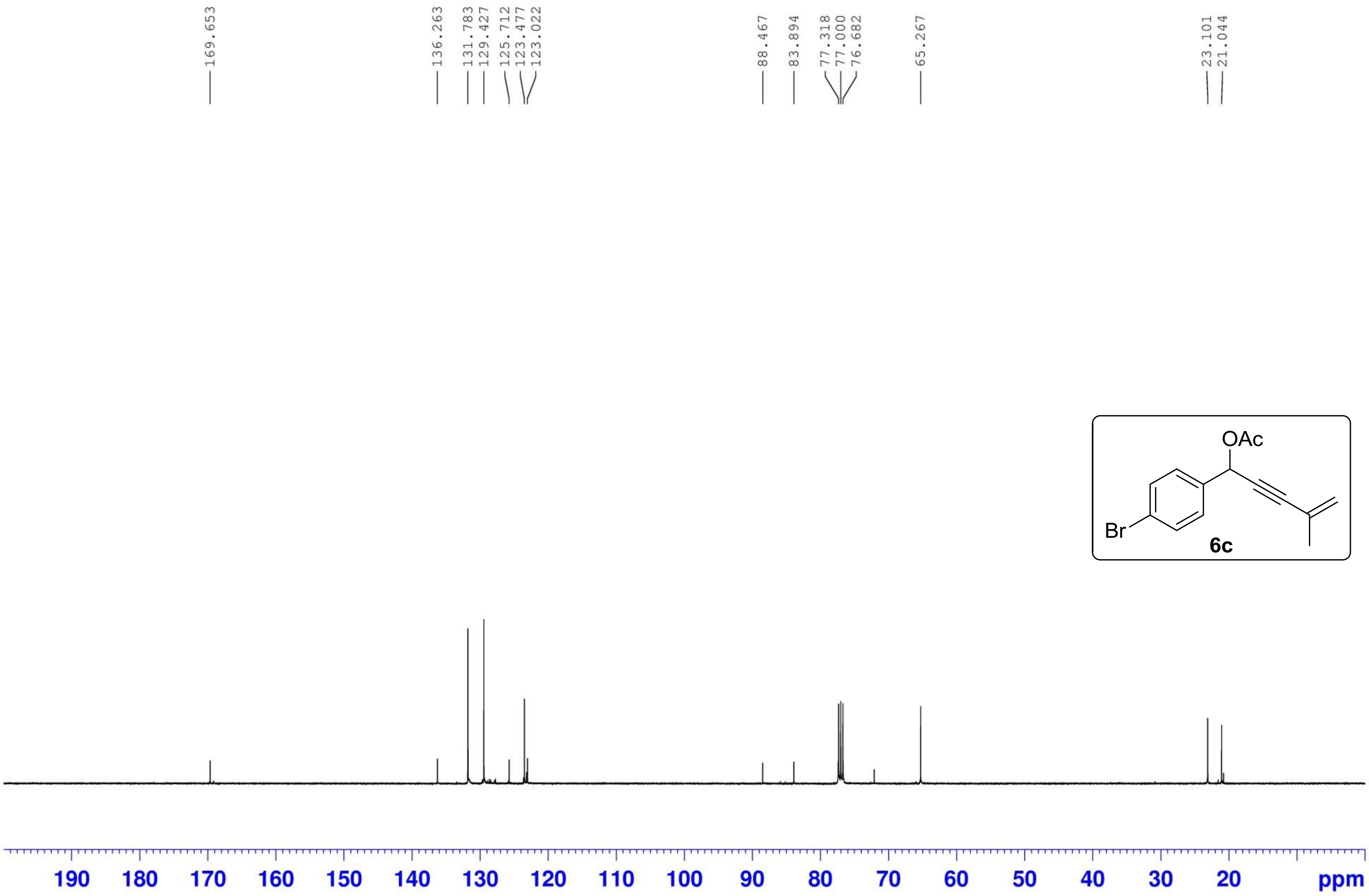
169.653
136.263
131.783
129.427
125.712
123.477
123.022
88.467
83.894
77.318
77.000
76.682
65.267
23.101
21.044
190 180 170 160 150 140 130 120 110 100 90 80 70 60 50 40 30 20 ppm
OAc
Br
6c

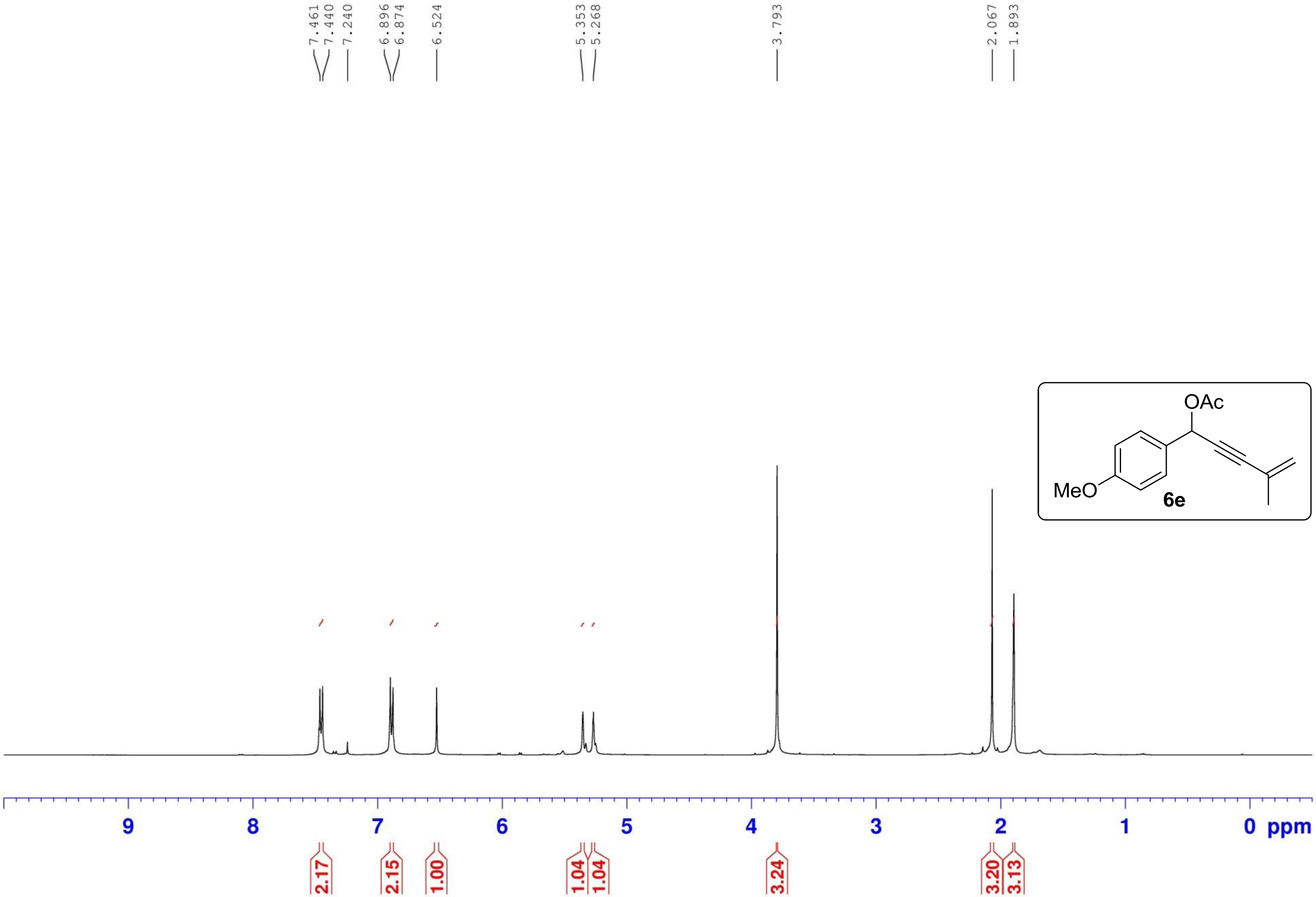
7.461
7.440
7.240
6.896
6.874
6.524
5.353
5.268
3.793
2.067
1.893
OAc
MeO
6e
9
8
7
6
5
4
3
2
1
0 ppm
2.17
2.15
1.00
1.04
1.04
3.24
3.20
3.13

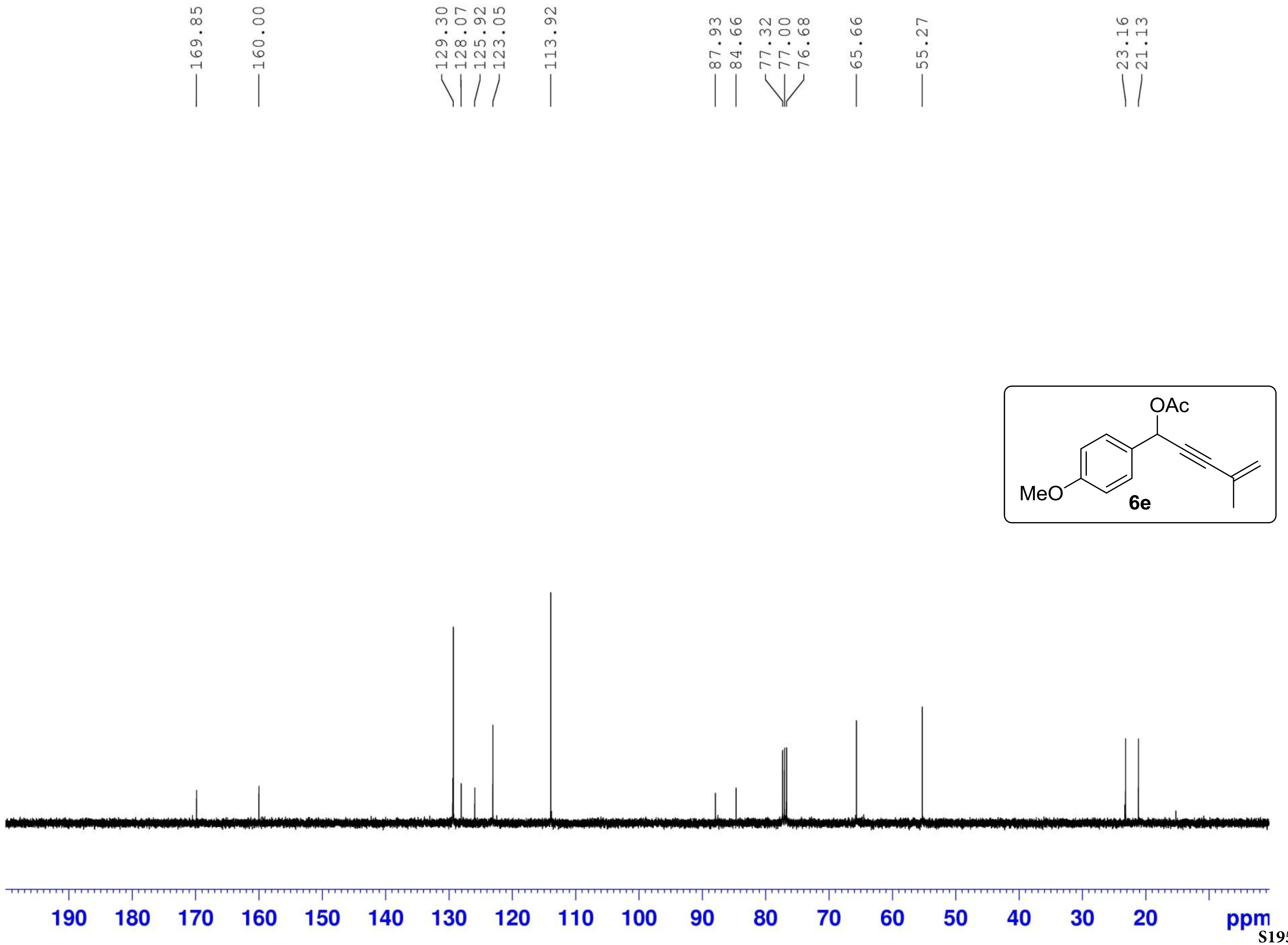
169.85
160.00
129.30
128.07
125.92
123.05
113.92
87.93
84.66
77.32
77.00
76.68
65.66
55.27
23.16
21.13
OAc
MeO
6e
190 180 170 160 150 140 130 120 110 100 90 80 70 60 50 40 30 20
ppm

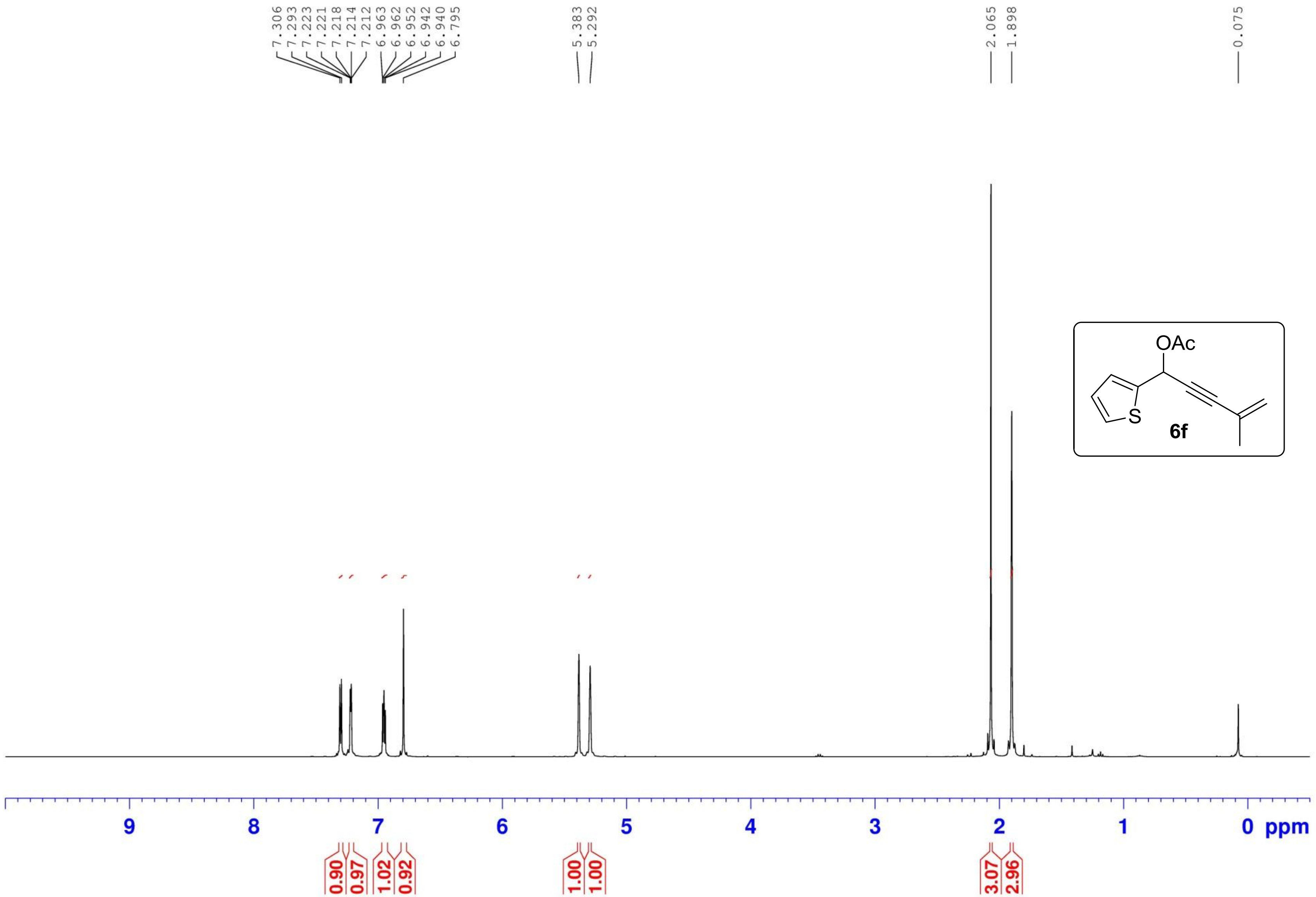
7.306
7.293
7.223
7.221
7.218
7.214
7.212
6.963
6.962
6.952
6.942
6.940
6.795
5.383
5.292
2.065
1.898
0.075
OAc
S
6f
9
8
7
6
5
4
3
2
1
0 ppm
0.90
0.97
1.02
0.92
1.00
1.00
3.07
2.96

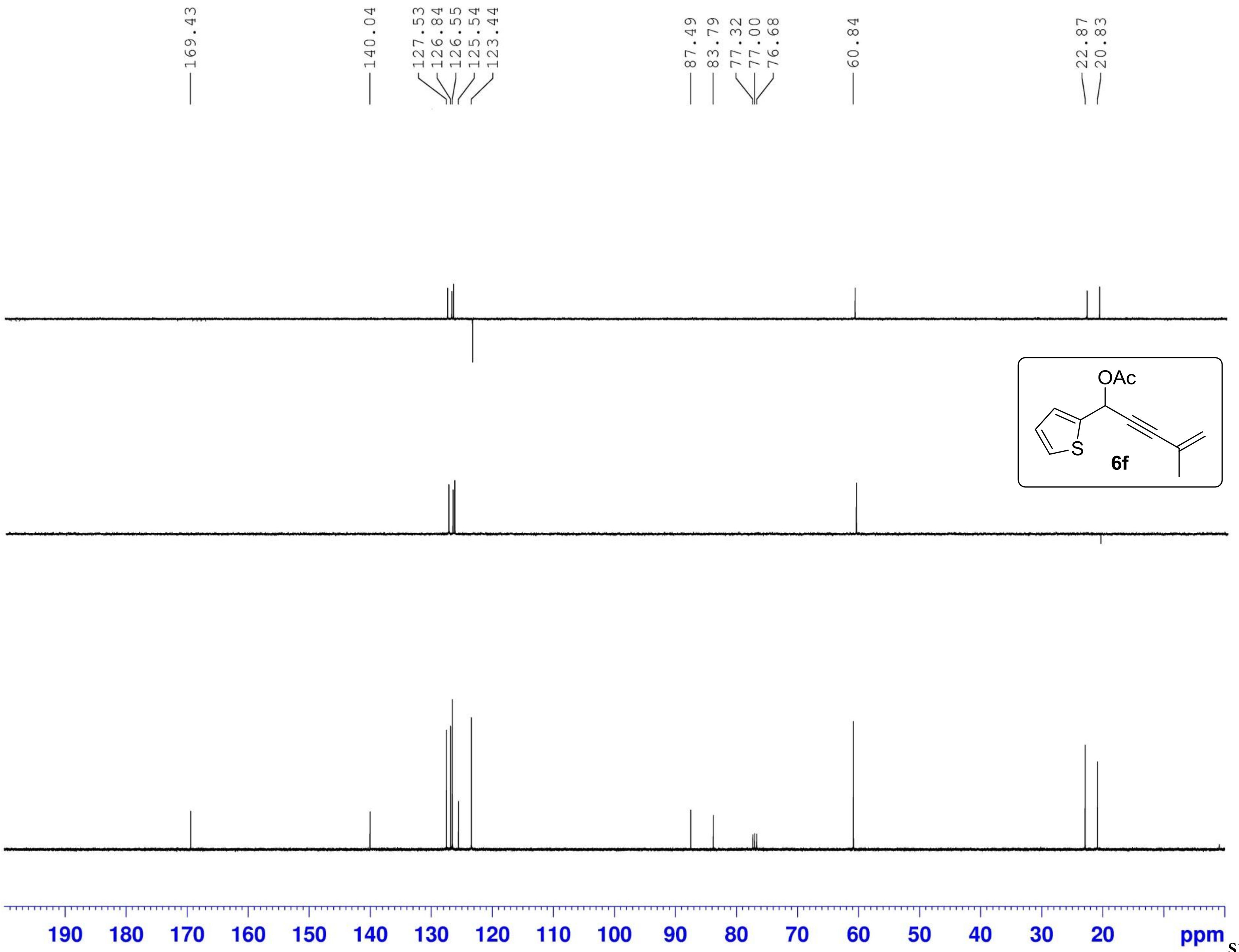
169.43
140.04
127.53
126.84
126.55
125.54
123.44
87.49
83.79
77.32
77.00
76.68
60.84
22.87
20.83
OAc
S
6f
190 180 170 160 150 140 130 120 110 100 90 80 70 60 50 40 30 20 ppm

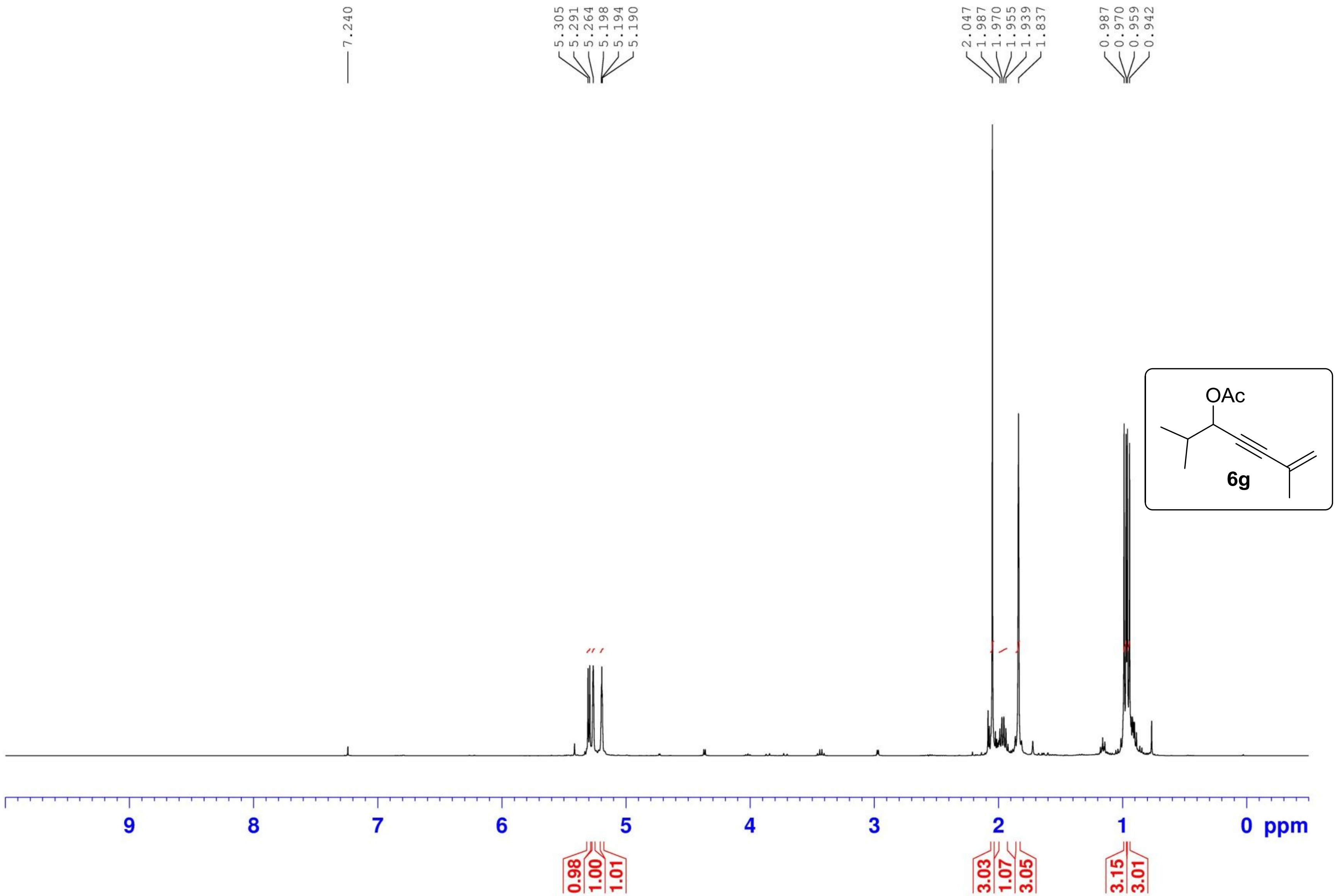
7.240
5.305
5.291
5.264
5.198
5.194
5.190
2.047
1.987
1.970
1.955
1.939
1.837
0.987
0.970
0.959
0.942
OAc
6g
9
8
7
6
5
4
3
2
1
0 ppm
0.98
1.00
1.01
3.03
1.07
3.05
3.15
3.01

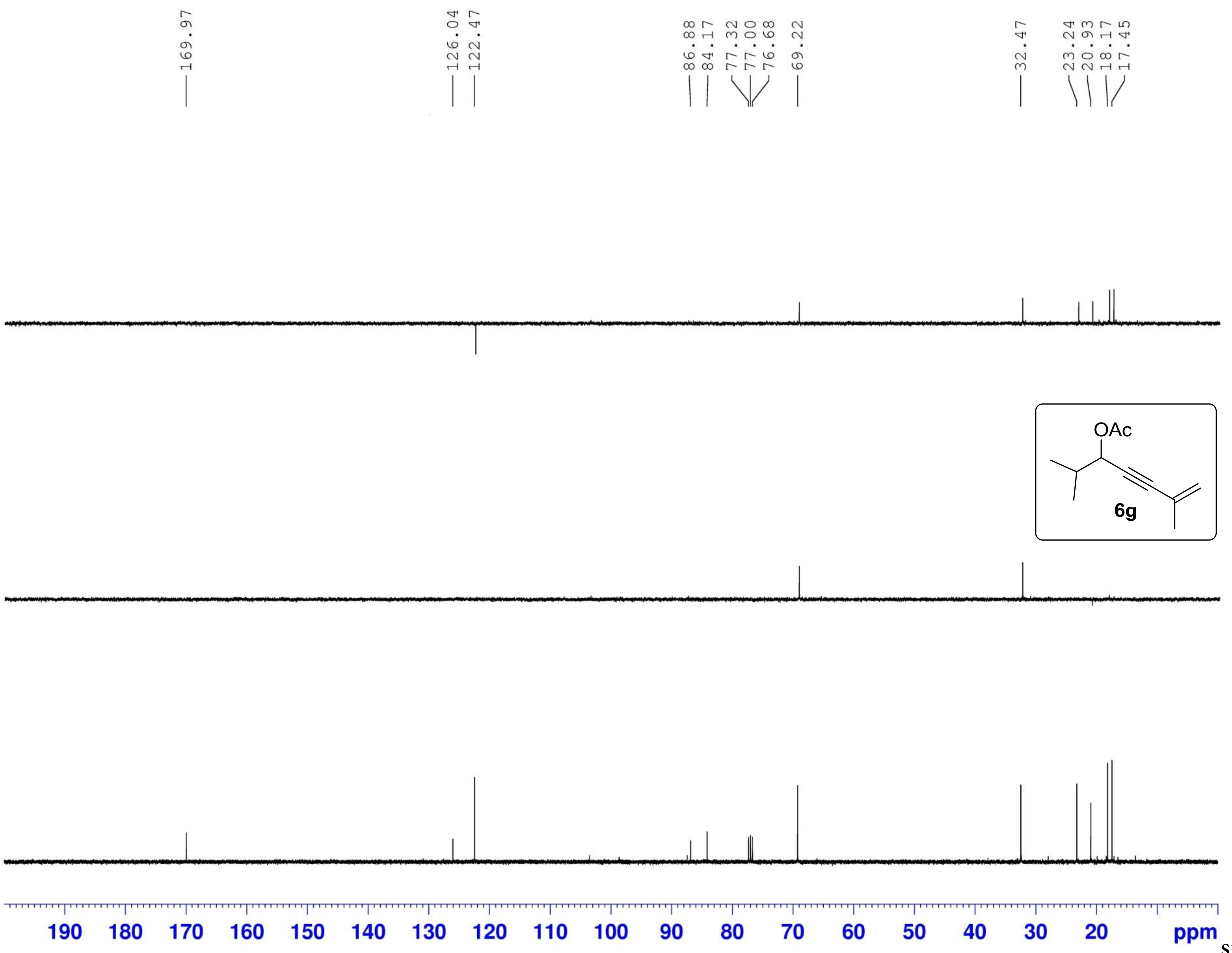
169.97
126.04
122.47
86.88
84.17
77.32
77.00
76.68
69.22
32.47
23.24
20.93
18.17
17.45
OAc
6g
190
180
170
160
150
140
130
120
110
100
90
80
70
60
50
40
30
20
ppm

BM-07-89

Current Data Parameters
NAME BM-07-89-1H.fid
EXPNO 1
PROCNO 1

F2 - Processing parameters
SI 32768
SF 399.7611797 MHz
WDW EM
SSB 0
LB 0.30 Hz
GB 0
PC 1.00

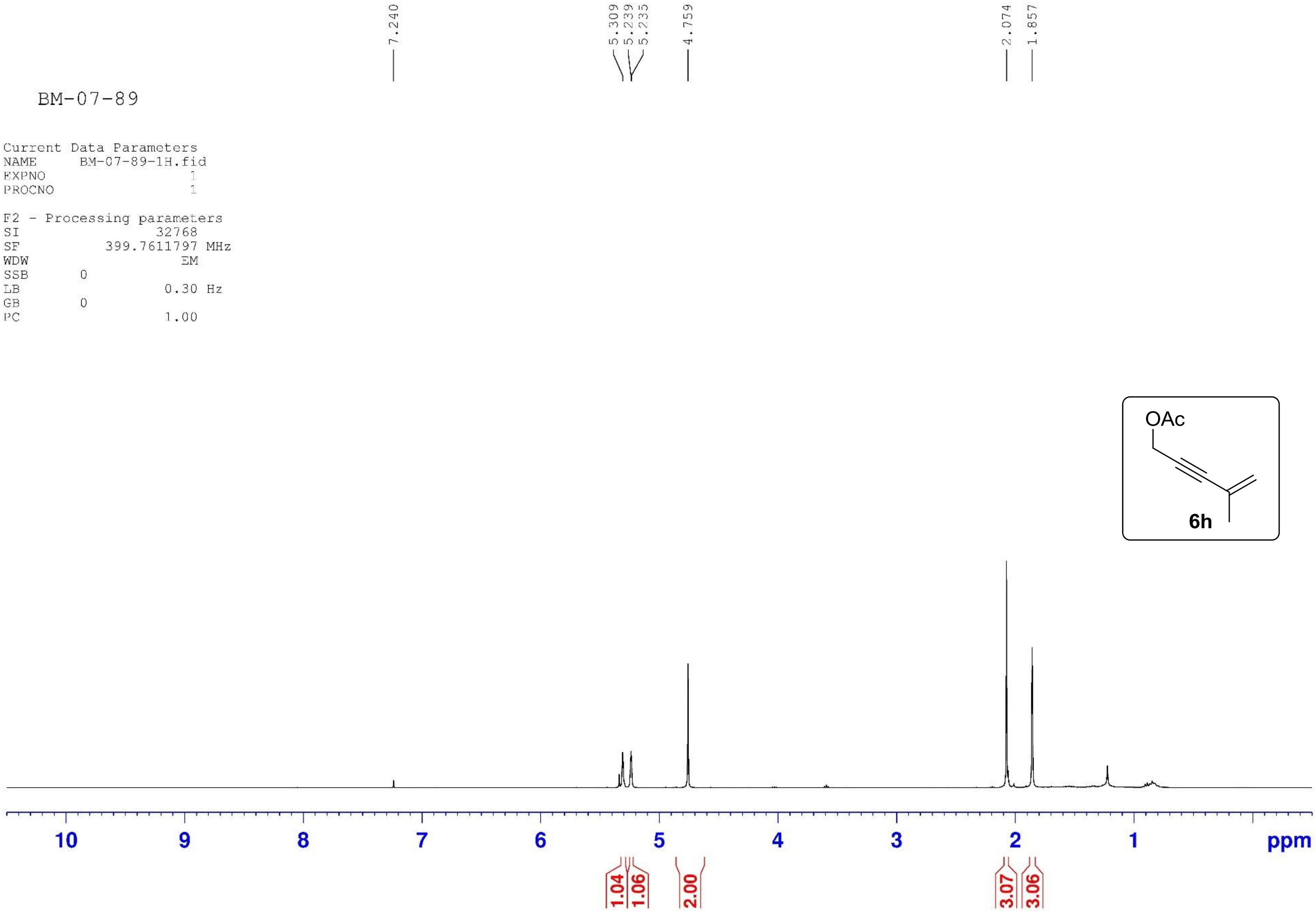

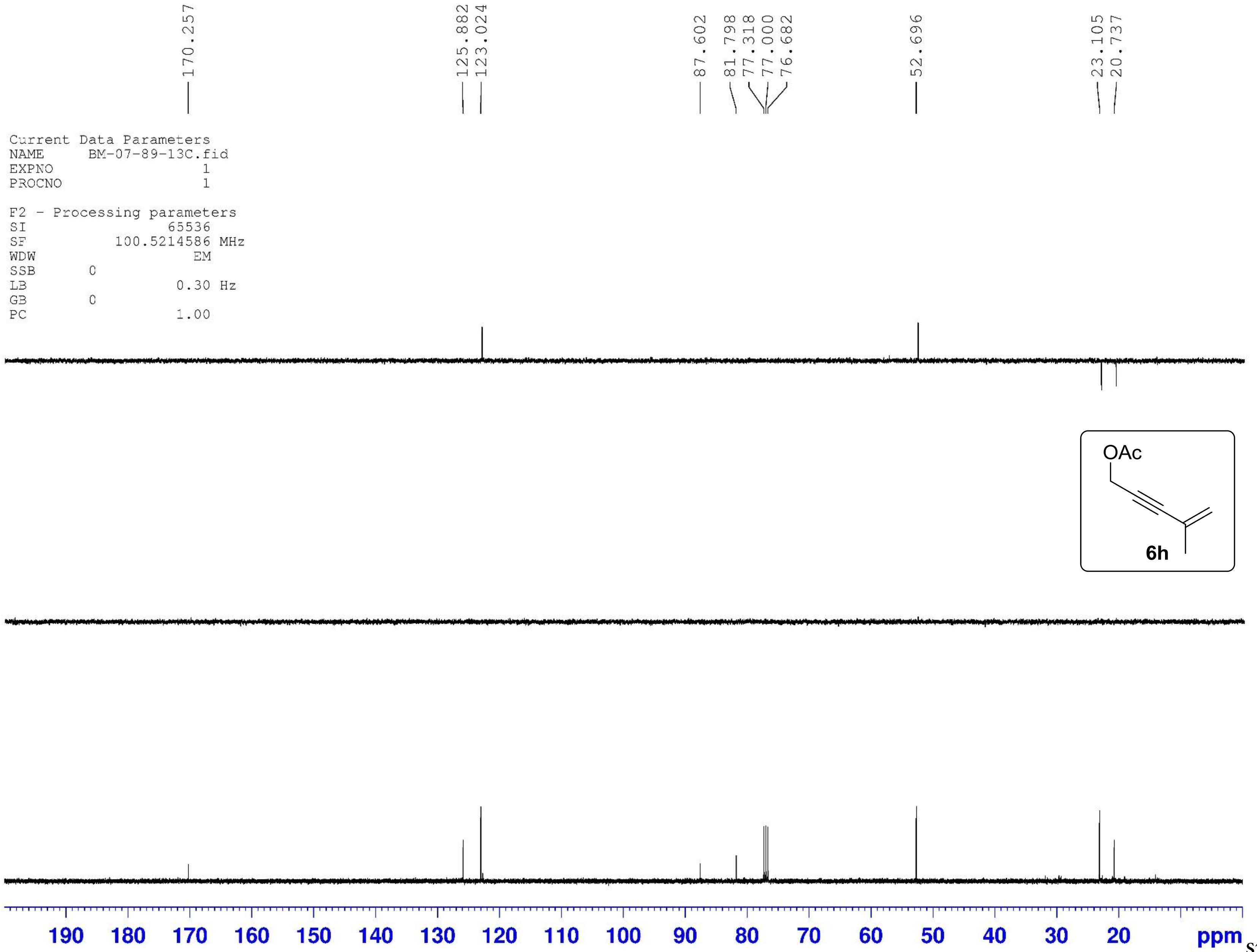
170.257
125.882
123.024
87.602
81.798
77.318
77.000
76.682
52.696
23.105
20.737
Current Data Parameters
NAME BM-07-89-13C.fid
EXPNO 1
PROCNO 1
F2 - Processing parameters
SI 65536
SF 100.5214586 MHz
WDW EM
SSB 0
LB 0.30 Hz
GB 0
PC 1.00
OAc
6h
190 180 170 160 150 140 130 120 110 100 90 80 70 60 50 40 30 20 ppm

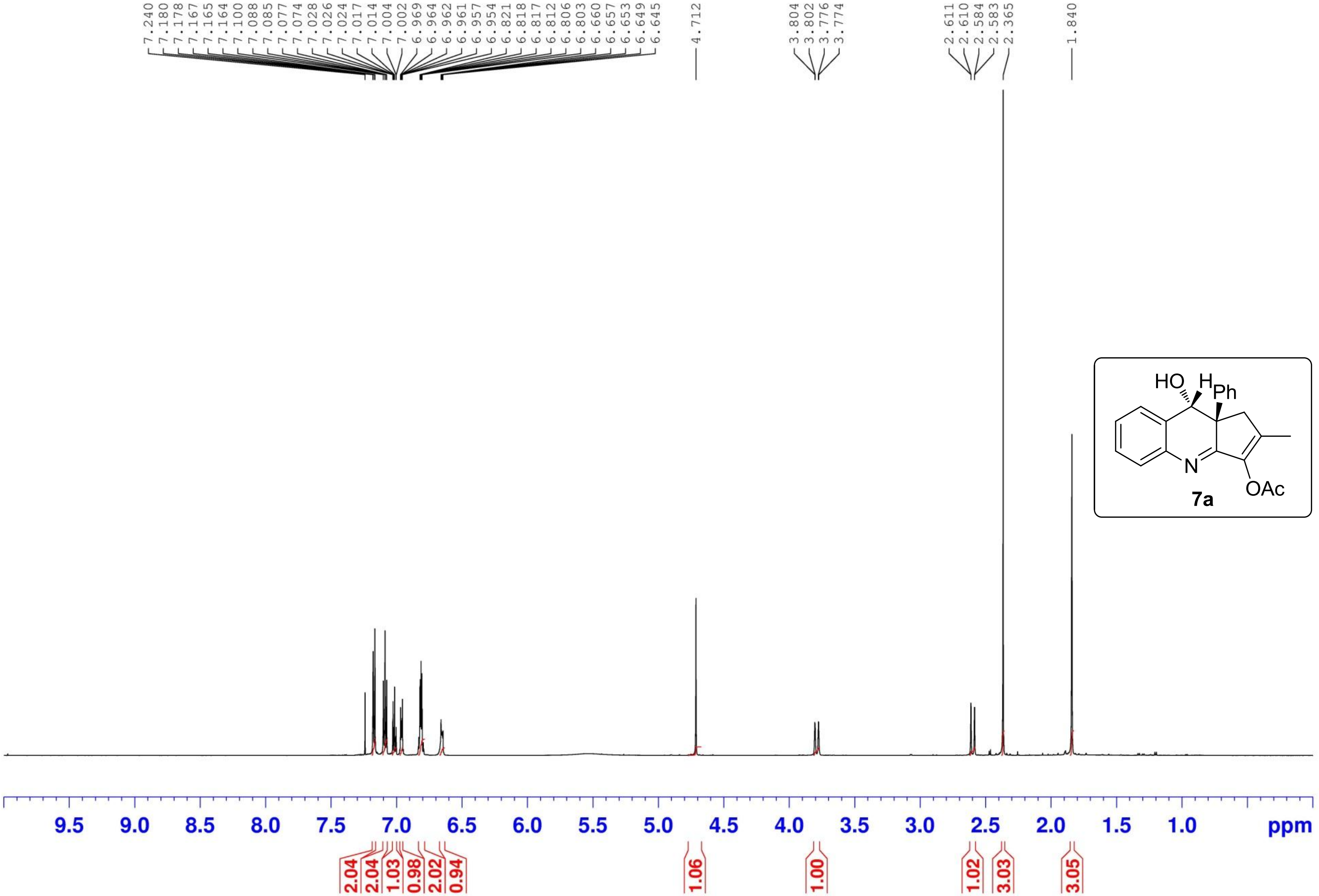
7.240
7.180
7.178
7.167
7.165
7.164
7.100
7.088
7.085
7.077
7.074
7.028
7.026
7.024
7.017
7.014
7.004
7.002
6.969
6.964
6.962
6.961
6.957
6.954
6.821
6.818
6.817
6.812
6.806
6.803
6.660
6.657
6.653
6.649
6.645
4.712
3.804
3.802
3.776
3.774
2.611
2.610
2.584
2.583
2.365
1.840
HO
H
Ph
N
OAc
7a
9.5
9.0
8.5
8.0
7.5
7.0
6.5
6.0
5.5
5.0
4.5
4.0
3.5
3.0
2.5
2.0
1.5
1.0
ppm
2.04
2.04
1.03
0.98
2.02
0.94
1.06
1.00
1.02
3.03
3.05

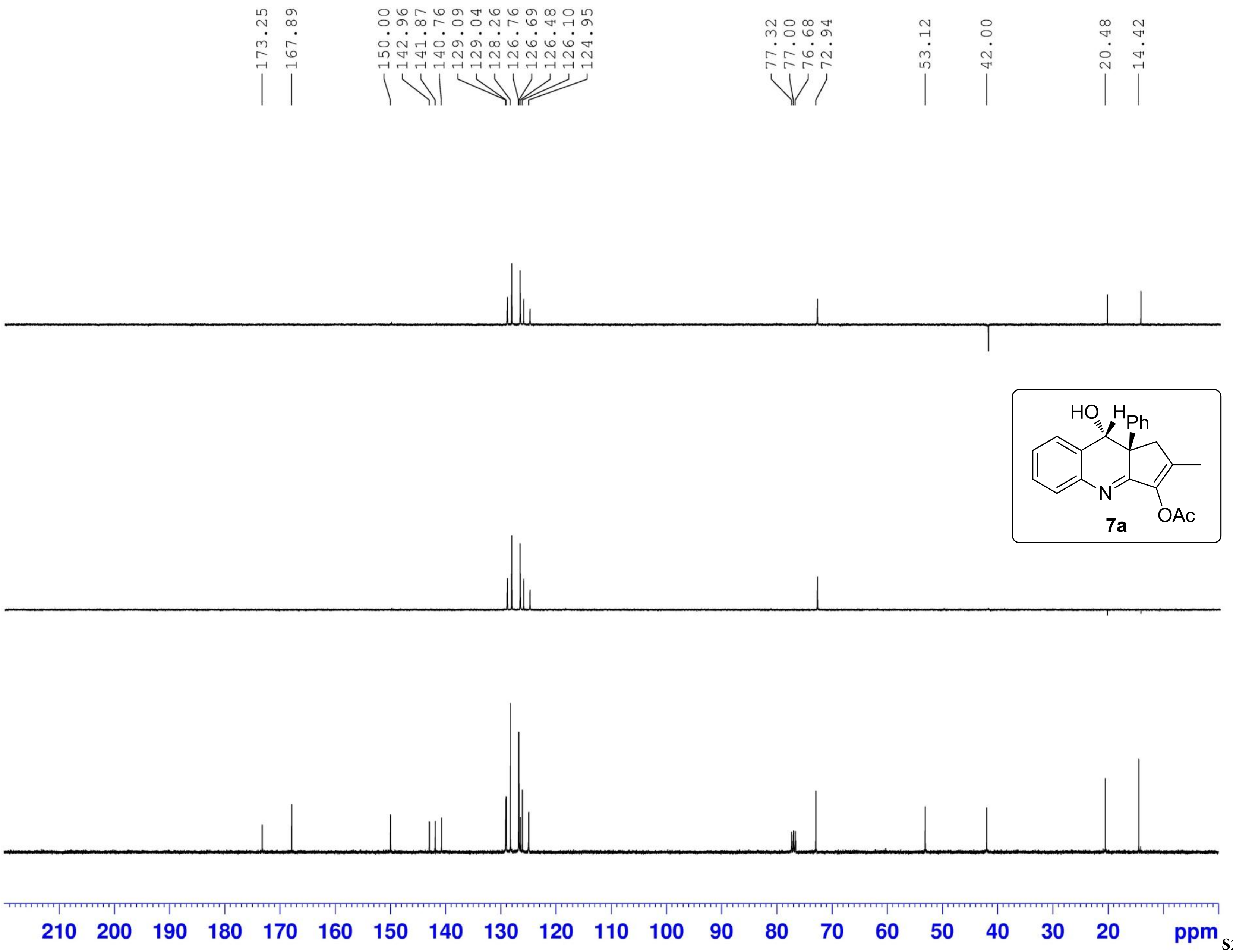
173.25
167.89
150.00
142.96
141.87
140.76
129.09
129.04
128.26
126.76
126.69
126.48
126.10
124.95
77.32
77.00
76.68
72.94
53.12
42.00
20.48
14.42
HO
H
Ph
N
OAc
7a
210 200 190 180 170 160 150 140 130 120 110 100 90 80 70 60 50 40 30 20 ppm

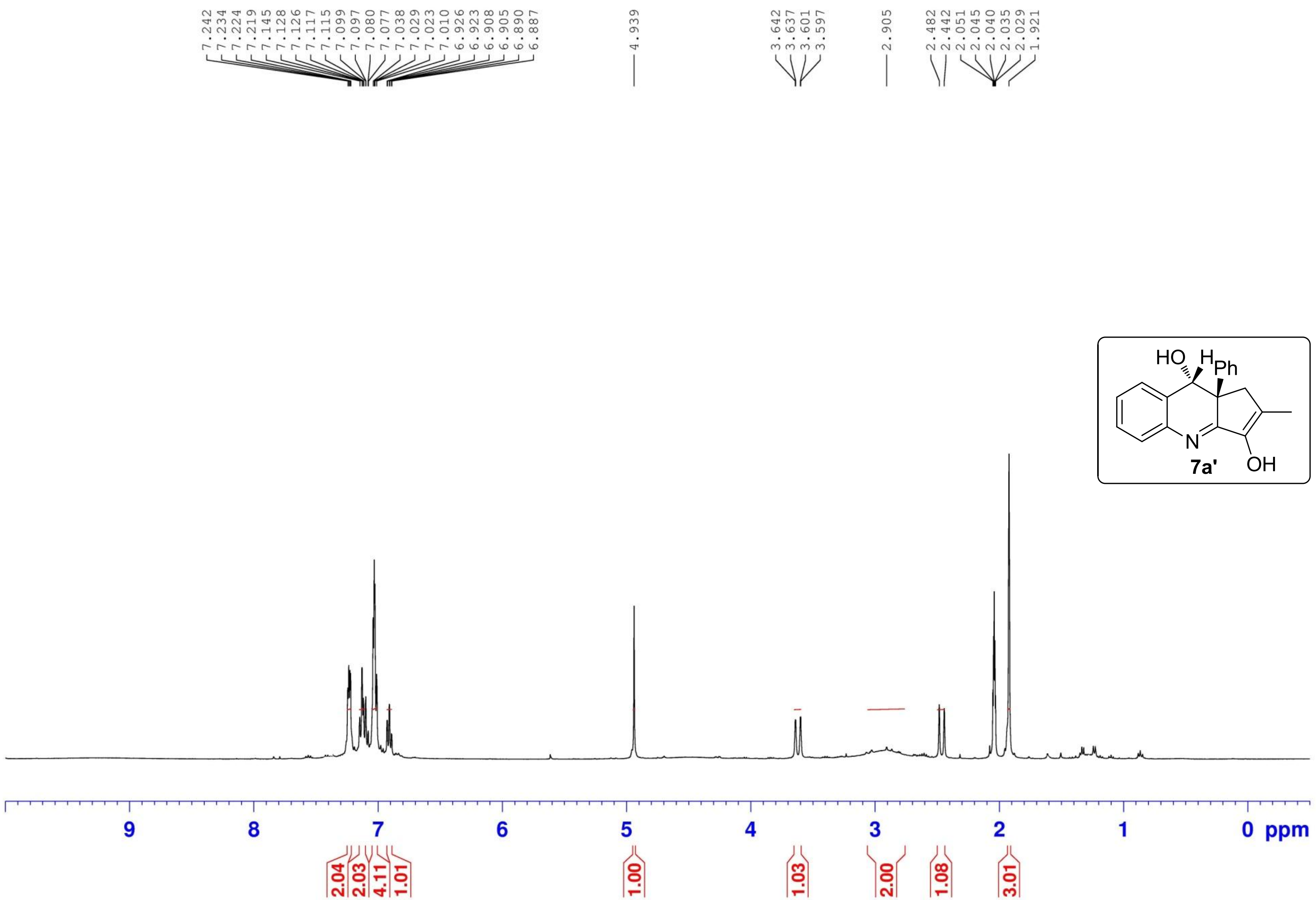
7.242
7.234
7.224
7.219
7.145
7.128
7.126
7.117
7.115
7.099
7.097
7.080
7.077
7.038
7.029
7.023
7.010
6.926
6.923
6.908
6.905
6.890
6.887
4.939
3.642
3.637
3.601
3.597
2.905
2.482
2.442
2.051
2.045
2.040
2.035
2.029
1.921
HO
H
Ph
N
OH
7a'
9
8
7
6
5
4
3
2
1
0 ppm
2.04
2.03
4.11
1.01
1.00
1.03
2.00
1.08
3.01

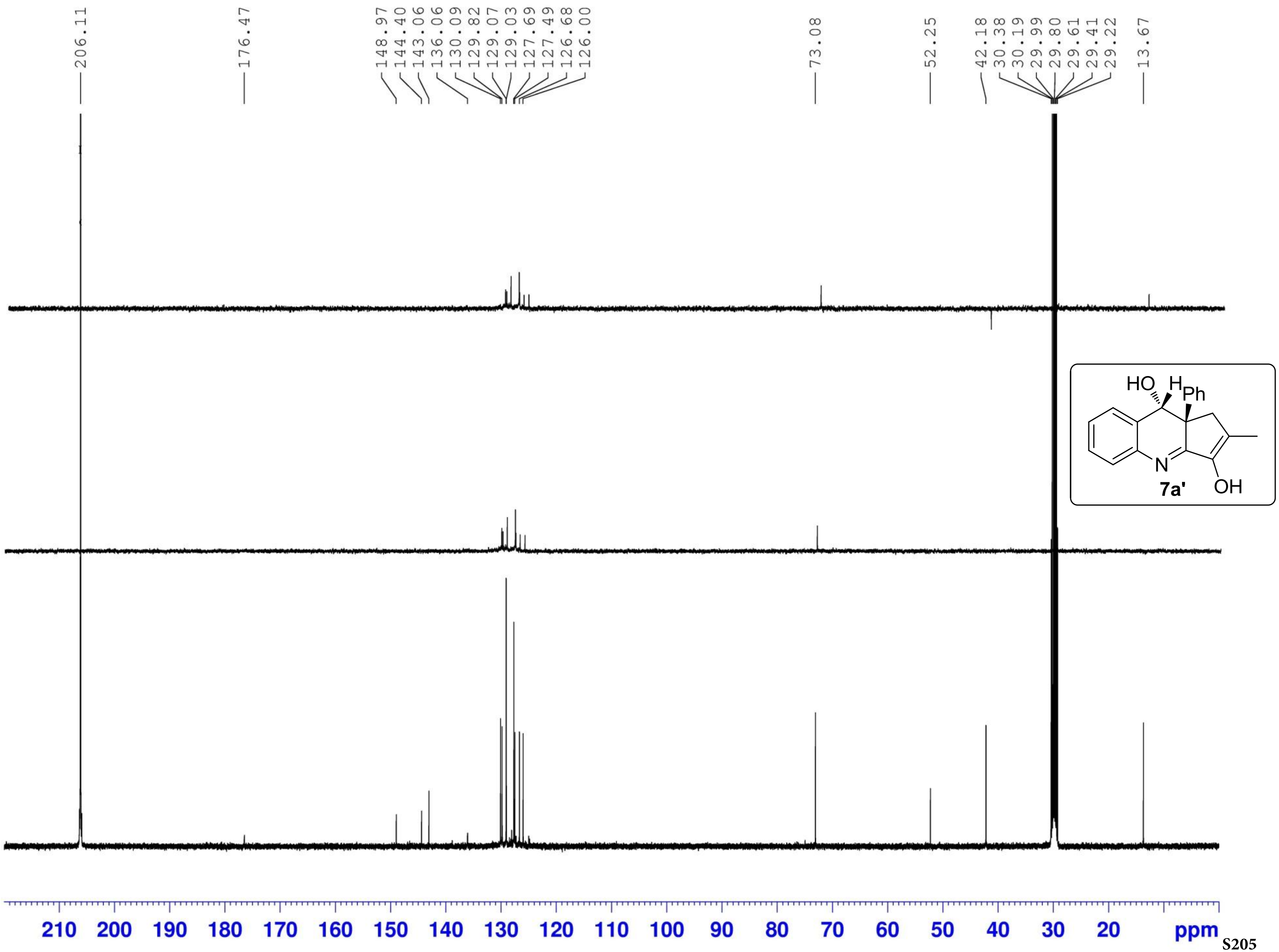

206.11
176.47
148.97
144.40
143.06
136.06
130.09
129.82
129.07
129.03
127.69
127.49
126.68
126.00
73.08
52.25
42.18
30.38
30.19
29.99
29.80
29.61
29.41
29.22
13.67
HO
H
Ph
N
OH
7a'
210 200 190 180 170 160 150 140 130 120 110 100 90 80 70 60 50 40 30 20 ppm

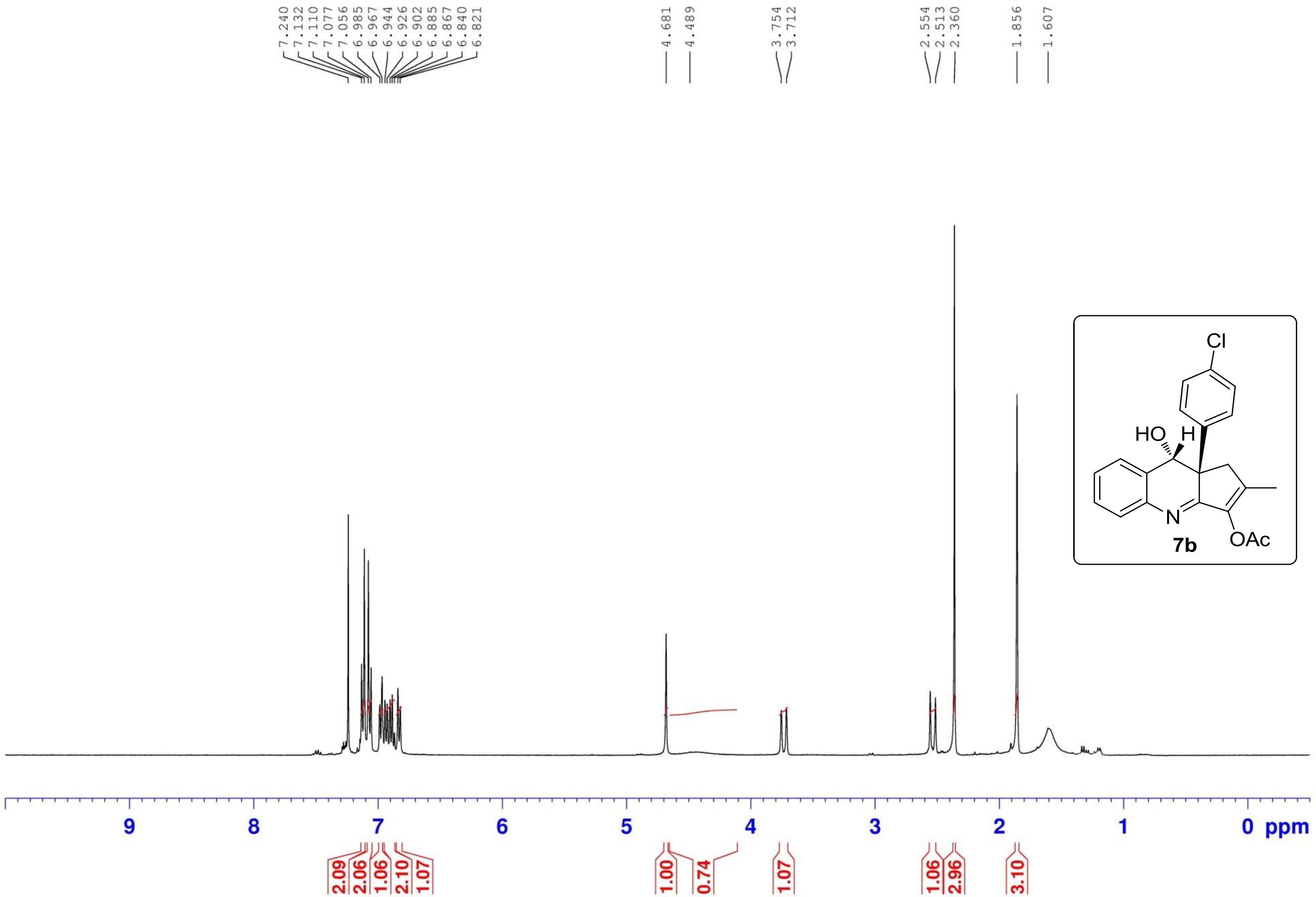

7.240
7.132
7.110
7.077
7.056
6.985
6.967
6.944
6.926
6.902
6.885
6.867
6.840
6.821
4.681
4.489
3.754
3.712
2.554
2.513
2.360
1.856
1.607
9
8
7
6
5
4
3
2
1
0 ppm
2.09
2.06
1.06
2.10
1.07
1.00
0.74
1.07
1.06
2.96
3.10
Cl
HO
H
N
OAc
7b

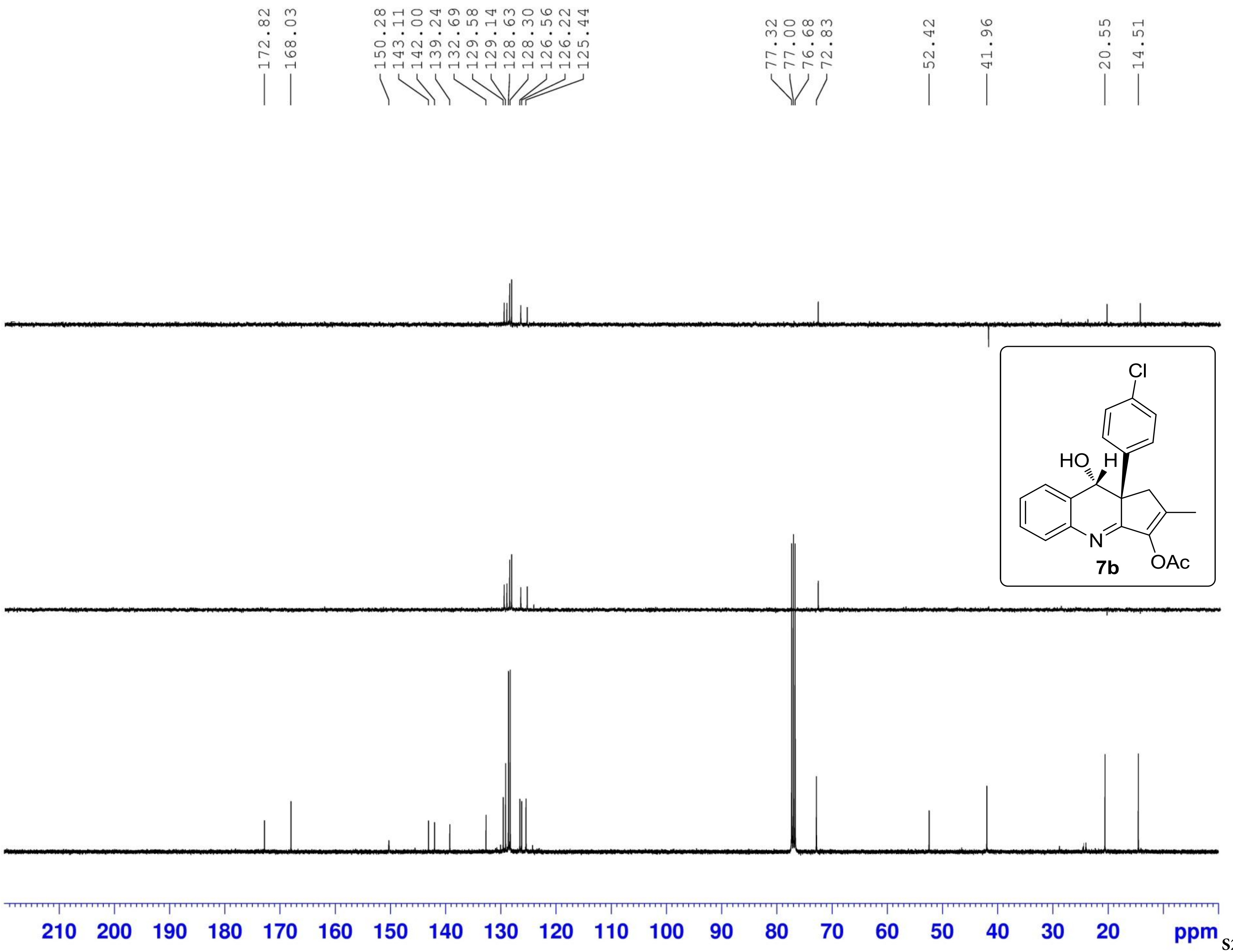
172.82
168.03
150.28
143.11
142.00
139.24
132.69
129.58
129.14
128.63
128.30
126.56
126.22
125.44
77.32
77.00
76.68
72.83
52.42
41.96
20.55
14.51
Cl
HO
H
N
OAc
7b
210
200
190
180
170
160
150
140
130
120
110
100
90
80
70
60
50
40
30
20
ppm

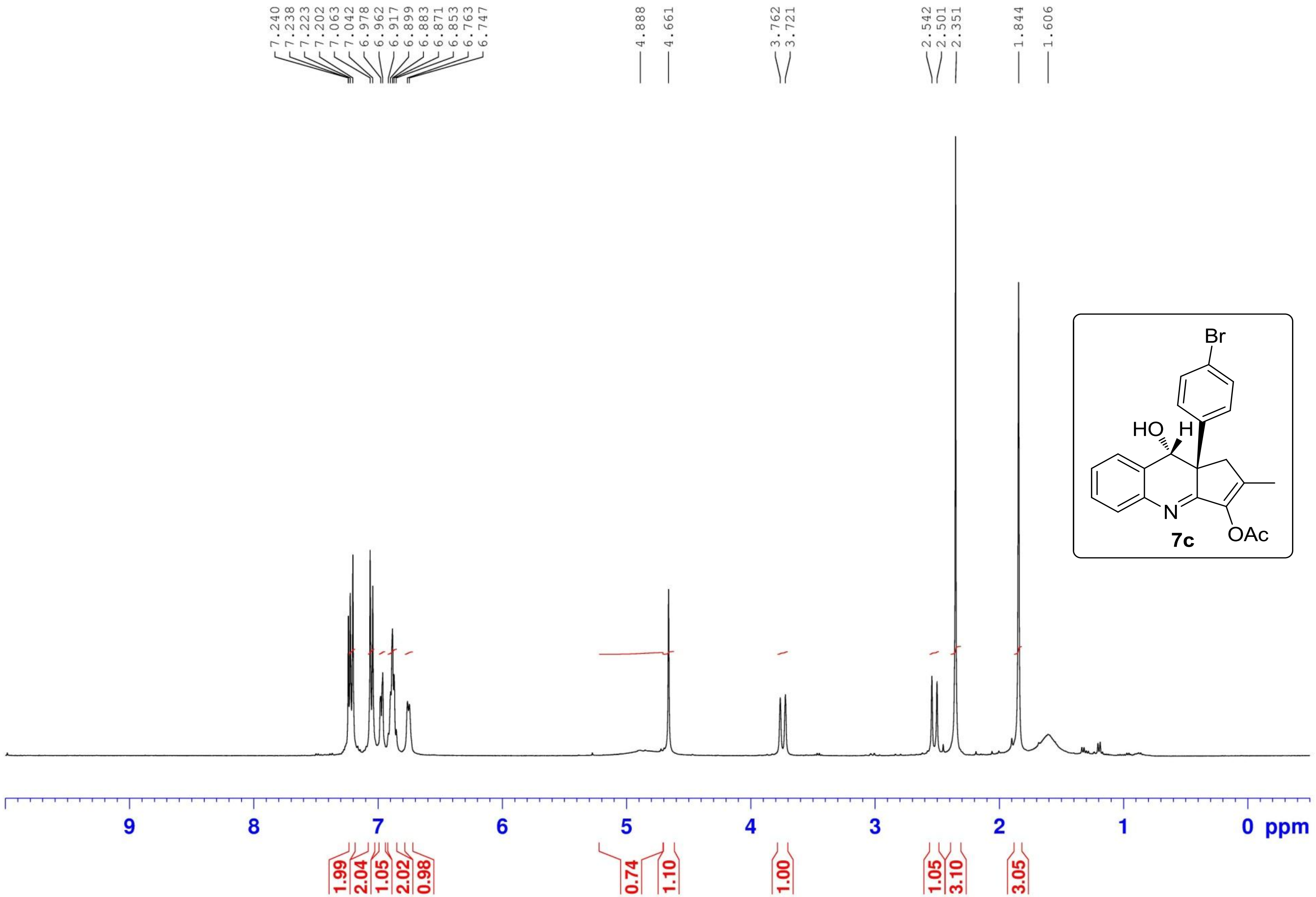

7.240
7.238
7.223
7.202
7.063
7.042
6.978
6.962
6.917
6.899
6.883
6.871
6.853
6.763
6.747
4.888
4.661
3.762
3.721
2.542
2.501
2.351
1.844
1.606
Br
HO
H
N
OAc
7c
9
8
7
6
5
4
3
2
1
0 ppm
1.99
2.04
1.05
2.02
0.98
0.74
1.10
1.00
1.05
3.10
3.05

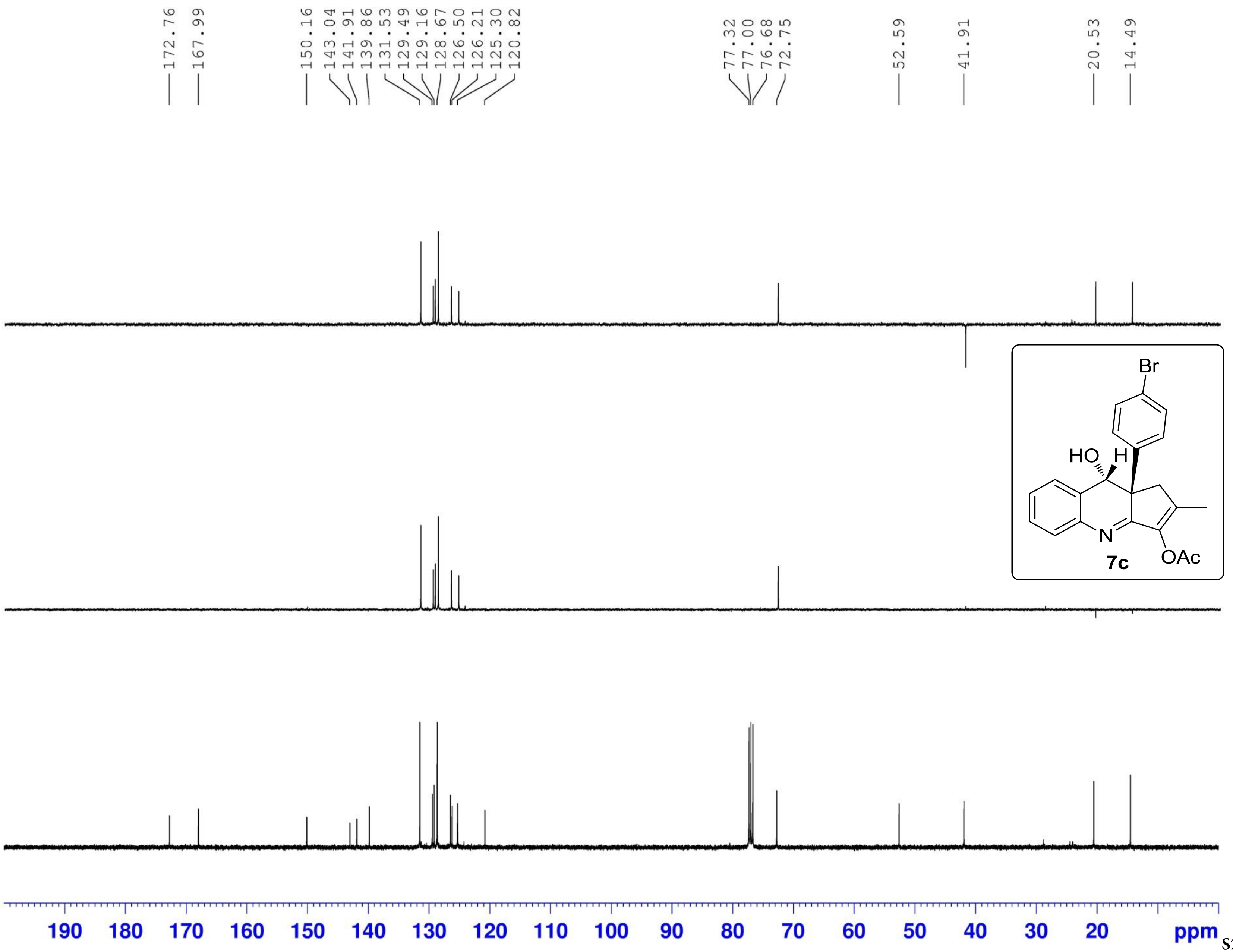
172.76
167.99
150.16
143.04
141.91
139.86
131.53
129.49
129.16
128.67
126.50
126.21
125.30
120.82
77.32
77.00
76.68
72.75
52.59
41.91
20.53
14.49
Br
HO
H
N
OAc
7c
190 180 170 160 150 140 130 120 110 100 90 80 70 60 50 40 30 20 ppm

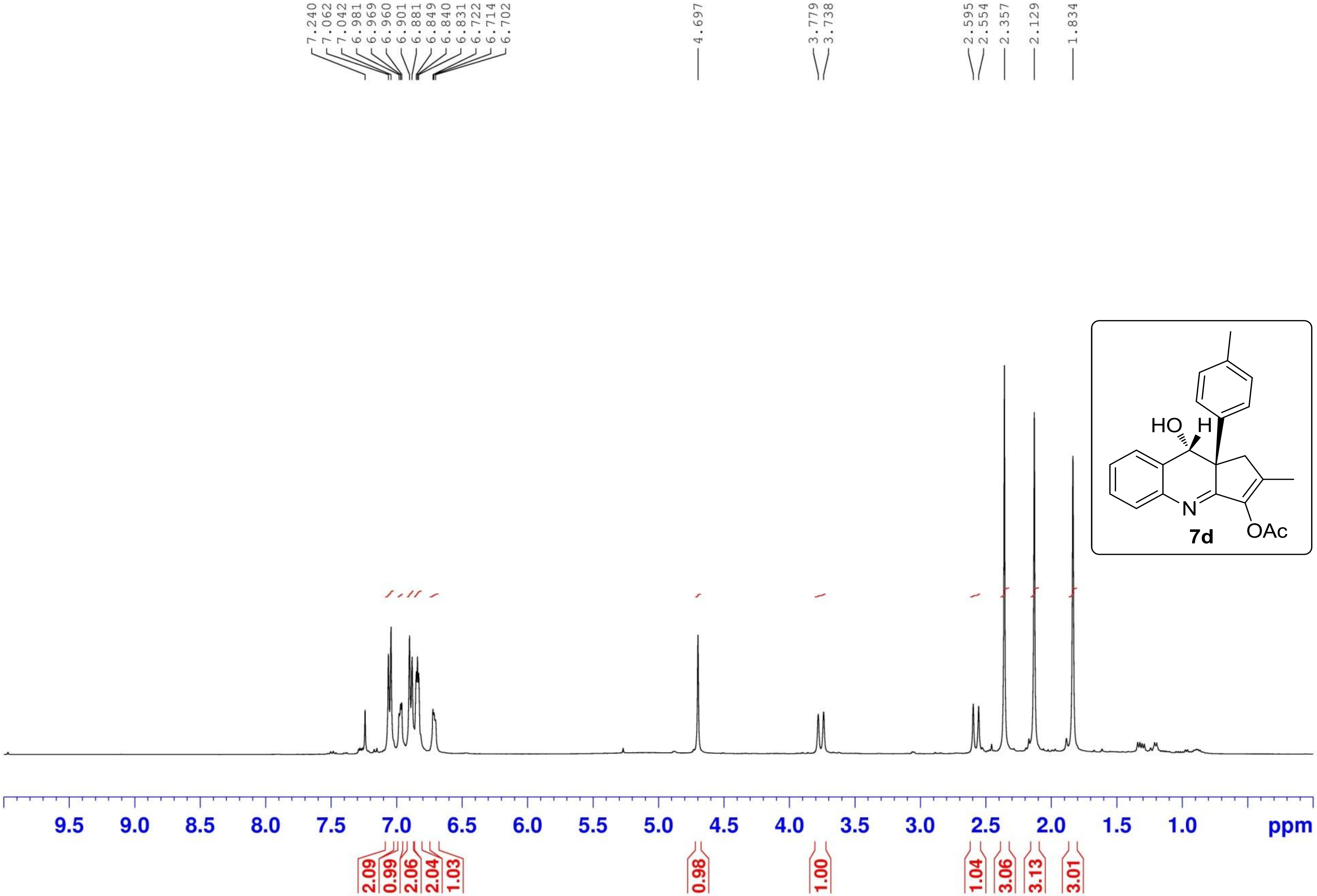
7.240
7.062
7.042
6.981
6.969
6.960
6.901
6.881
6.849
6.840
6.831
6.722
6.714
6.702
4.697
3.779
3.738
2.595
2.554
2.357
2.129
1.834
HO
H
N
OAc
7d
9.5
9.0
8.5
8.0
7.5
7.0
6.5
6.0
5.5
5.0
4.5
4.0
3.5
3.0
2.5
2.0
1.5
1.0
ppm
2.09
0.99
2.06
2.04
1.03
0.98
1.00
1.04
3.06
3.13
3.01

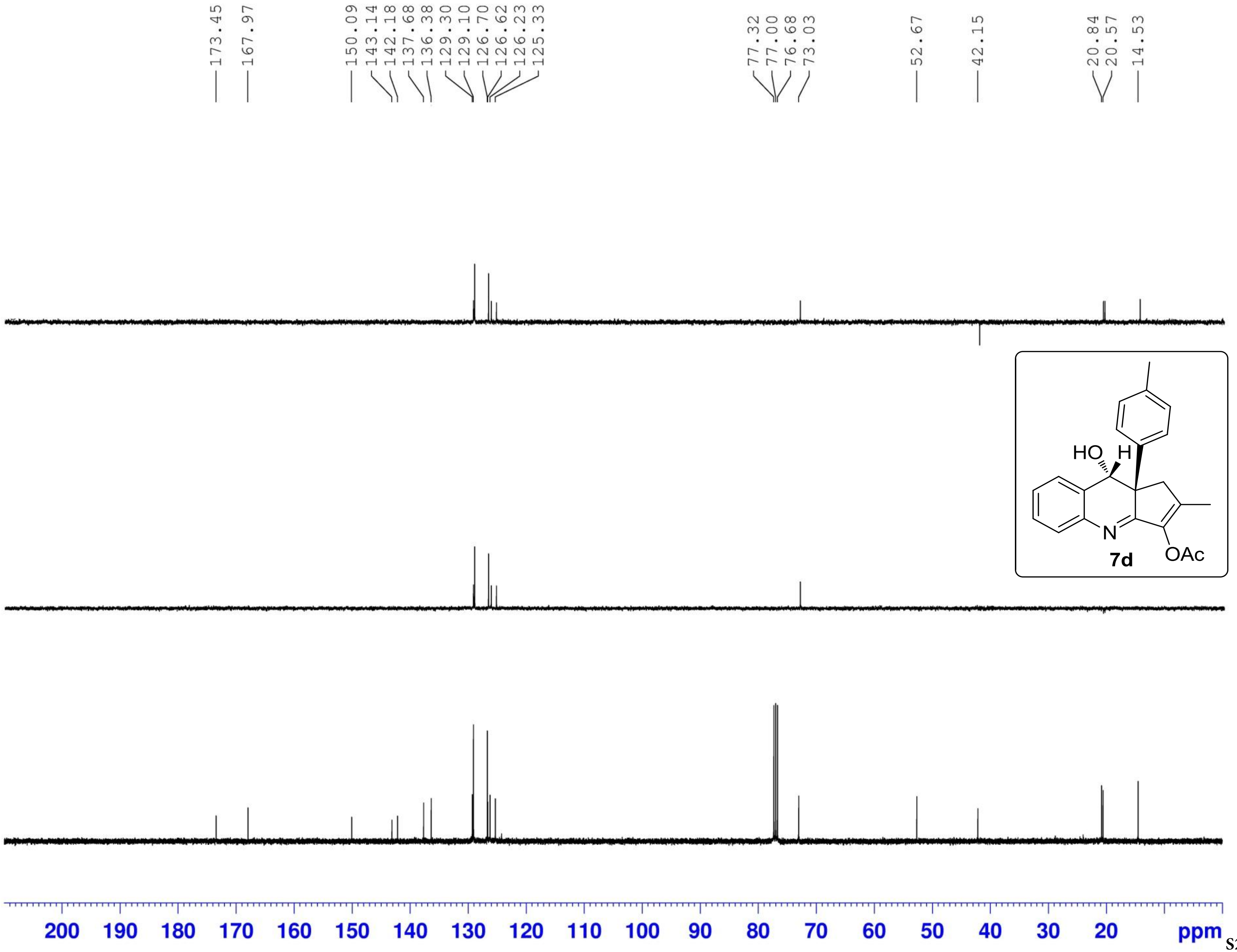
173.45
167.97
150.09
143.14
142.18
137.68
136.38
129.30
129.10
126.70
126.62
126.23
125.33
77.32
77.00
76.68
73.03
52.67
42.15
20.84
20.57
14.53
HO
H
N
OAc
7d
200 190 180 170 160 150 140 130 120 110 100 90 80 70 60 50 40 30 20 ppm

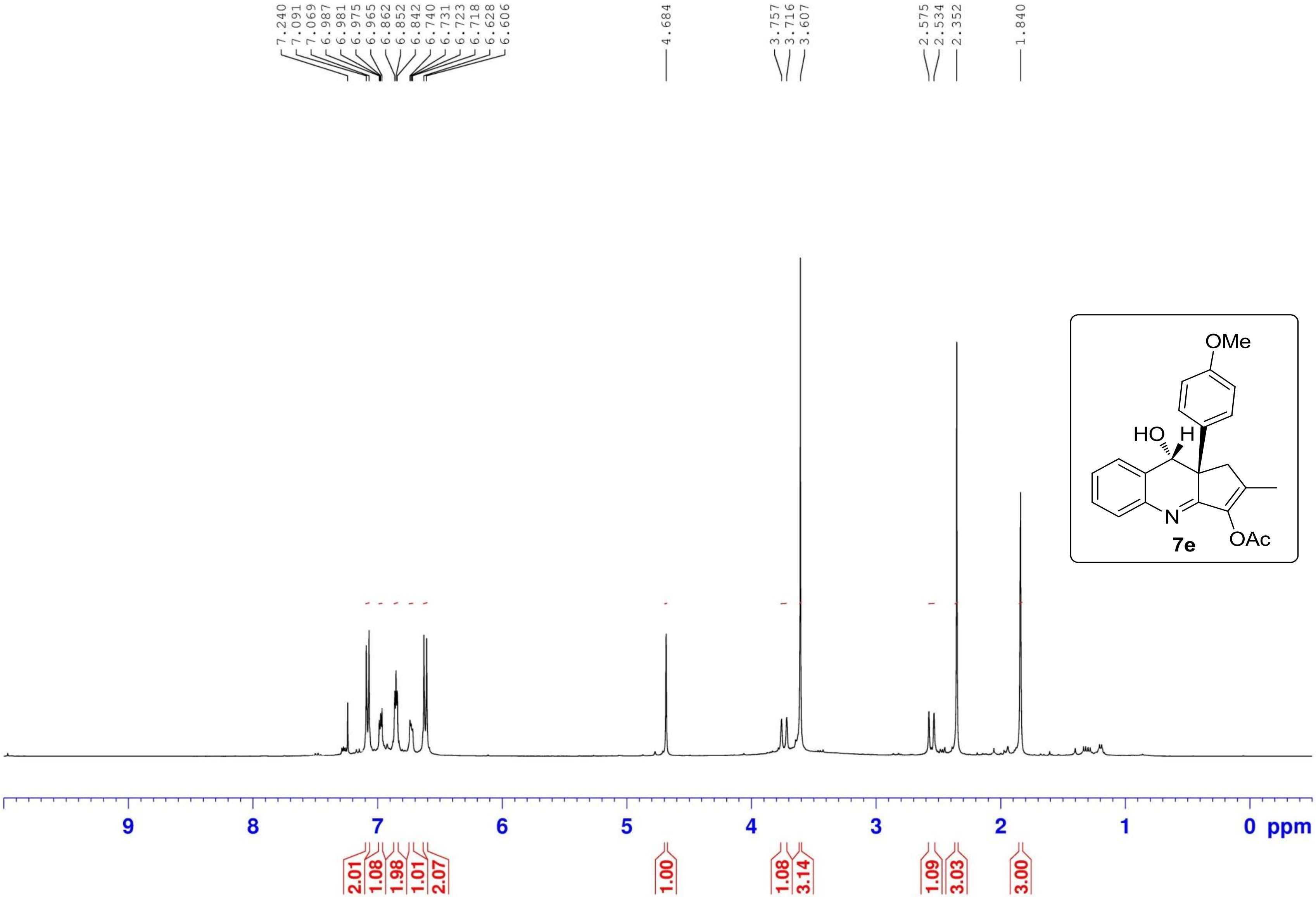
7.240
7.091
7.069
6.987
6.981
6.975
6.965
6.862
6.852
6.842
6.740
6.731
6.723
6.718
6.628
6.606
4.684
3.757
3.716
3.607
2.575
2.534
2.352
1.840
OMe
HO
H
N
OAc
7e
9
8
7
6
5
4
3
2
1
0 ppm
2.01
1.08
1.98
1.01
2.07
1.00
1.08
3.14
1.09
3.03
3.00

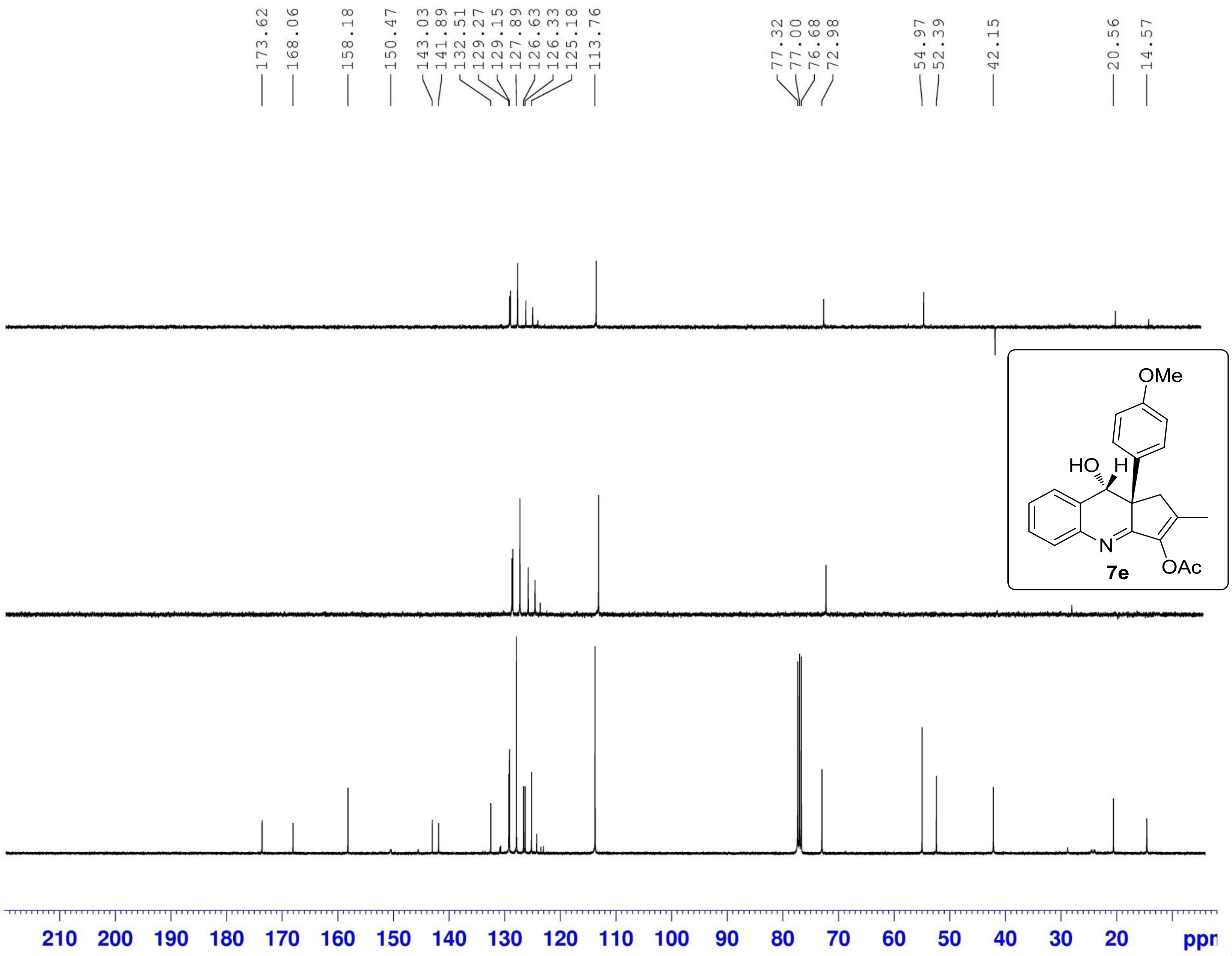
173.62
168.06
158.18
150.47
143.03
141.89
132.51
129.27
129.15
127.89
126.63
126.33
125.18
113.76
77.32
77.00
76.68
72.98
54.97
52.39
42.15
20.56
14.57
OMe
HO
H
N
OAc
7e
210 200 190 180 170 160 150 140 130 120 110 100 90 80 70 60 50 40 30 20 ppm

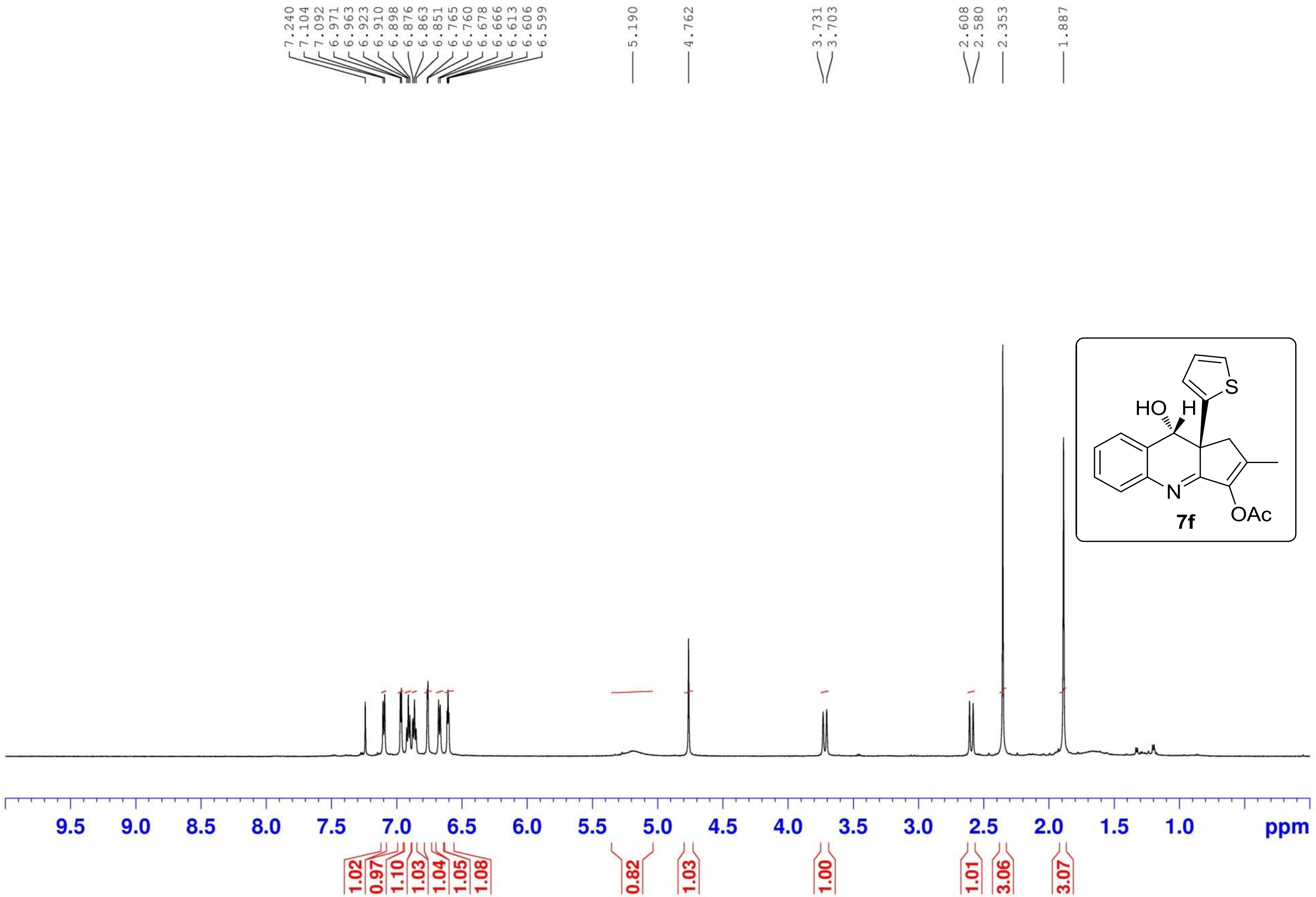

7.240
7.104
7.092
6.971
6.963
6.923
6.910
6.898
6.876
6.863
6.851
6.765
6.760
6.678
6.666
6.613
6.606
6.599
5.190
4.762
3.731
3.703
2.608
2.580
2.353
1.887
HO
H
S
N
OAc
7f
9.5
9.0
8.5
8.0
7.5
7.0
6.5
6.0
5.5
5.0
4.5
4.0
3.5
3.0
2.5
2.0
1.5
1.0
ppm
1.02
0.97
1.10
1.03
1.04
1.05
1.08
0.82
1.03
1.00
1.01
3.06
3.07

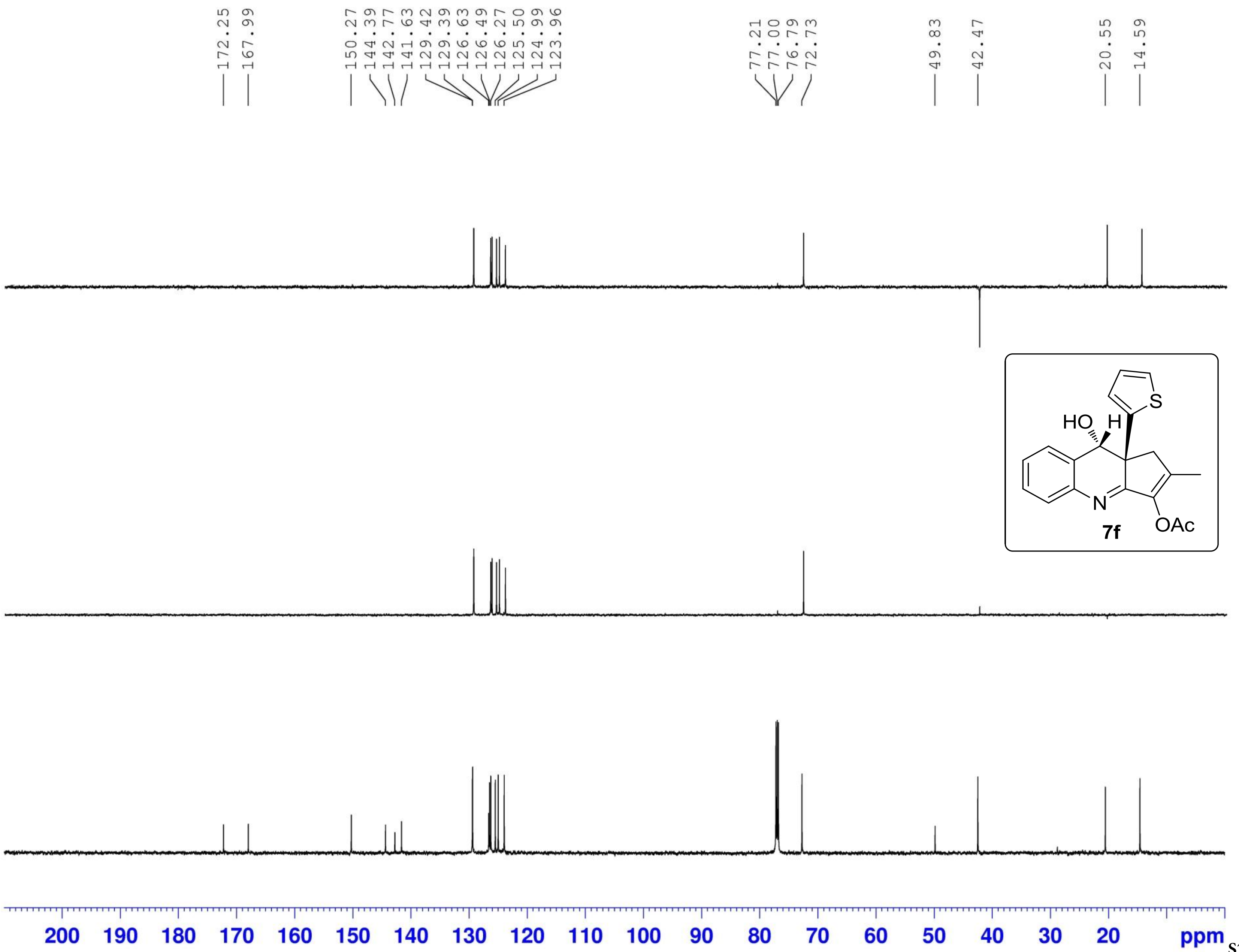
172.25
167.99
150.27
144.39
142.77
141.63
129.42
129.39
126.63
126.49
126.27
125.50
124.99
123.96
77.21
77.00
76.79
72.73
49.83
42.47
20.55
14.59
HO
H
S
N
OAc
7f
200
190
180
170
160
150
140
130
120
110
100
90
80
70
60
50
40
30
20
ppm

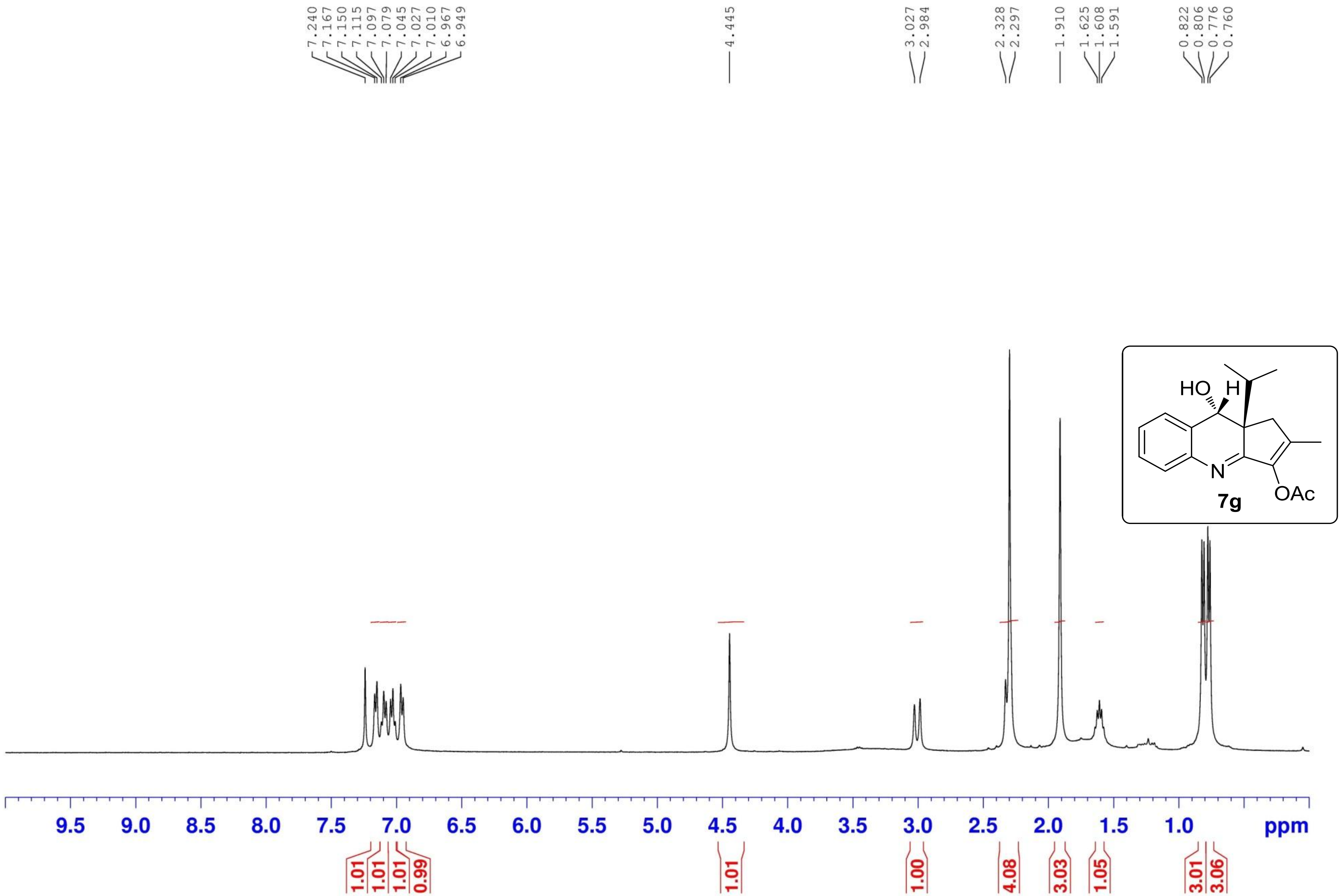
7.240
7.167
7.150
7.115
7.097
7.079
7.045
7.027
7.010
6.967
6.949
4.445
3.027
2.984
2.328
2.297
1.910
1.625
1.608
1.591
0.822
0.806
0.776
0.760
HO
H
N
OAc
7g
9.5
9.0
8.5
8.0
7.5
7.0
6.5
6.0
5.5
5.0
4.5
4.0
3.5
3.0
2.5
2.0
1.5
1.0
ppm
1.01
1.01
1.01
0.99
1.01
1.00
4.08
3.03
1.05
3.01
3.06

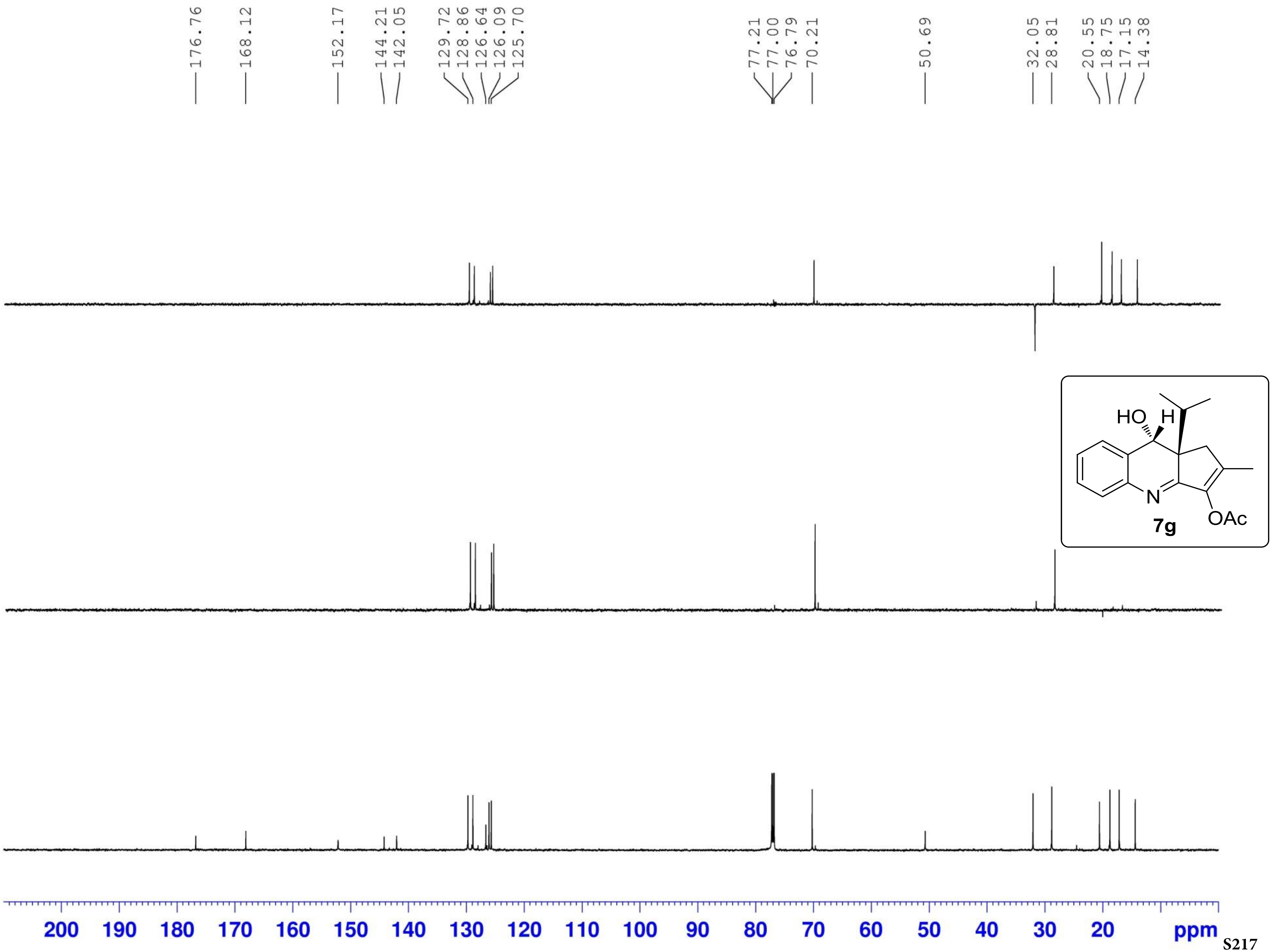
176.76
168.12
152.17
144.21
142.05
129.72
128.86
126.64
126.09
125.70
77.21
77.00
76.79
70.21
50.69
32.05
28.81
20.55
18.75
17.15
14.38
HO
H
N
OAc
7g
200 190 180 170 160 150 140 130 120 110 100 90 80 70 60 50 40 30 20 ppm

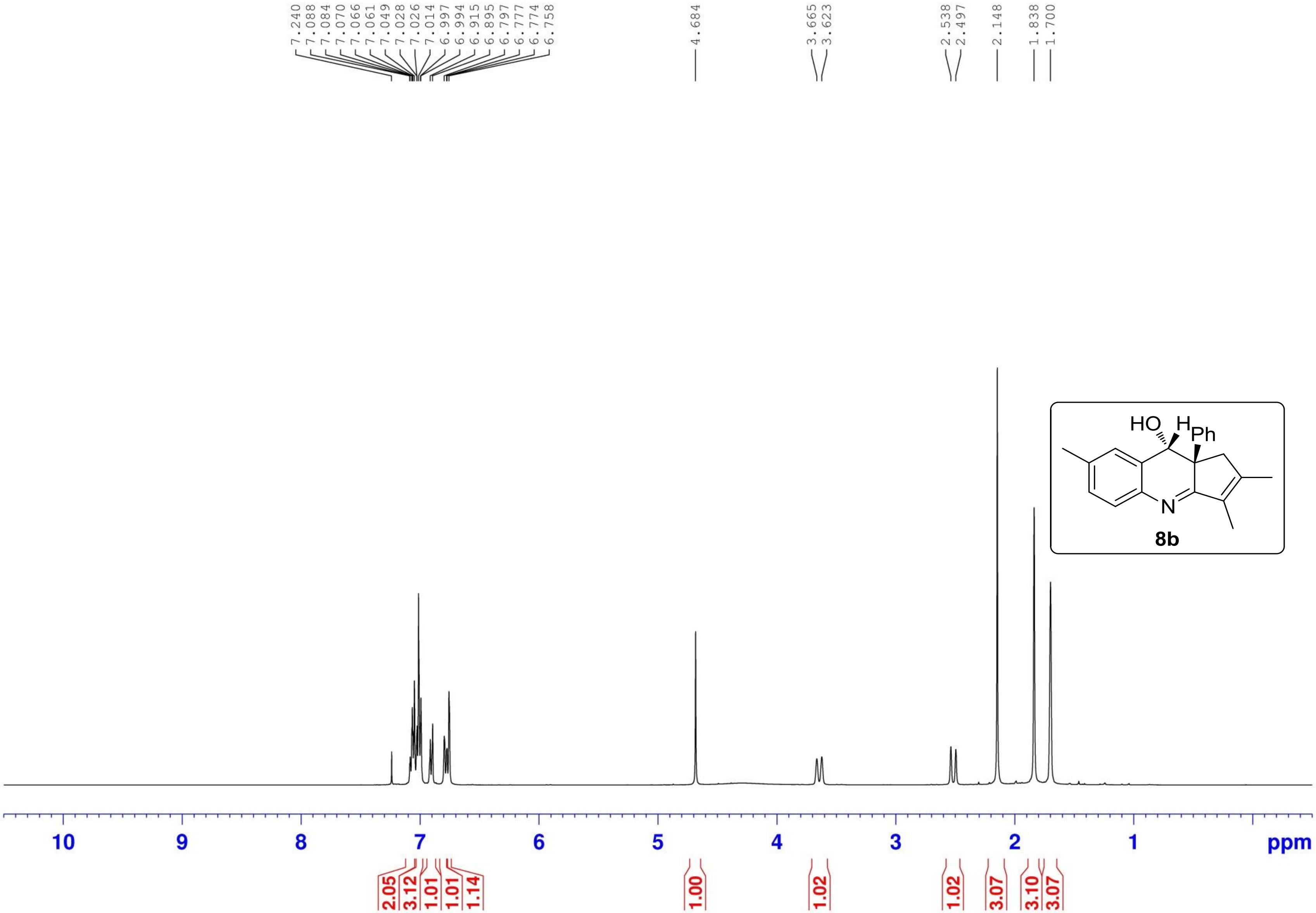
7.240
7.088
7.084
7.070
7.066
7.061
7.049
7.028
7.026
7.014
6.997
6.994
6.915
6.895
6.797
6.777
6.774
6.758
4.684
3.665
3.623
2.538
2.497
2.148
1.838
1.700
HO
H
Ph
N
8b
10
9
8
7
6
5
4
3
2
1
ppm
2.05
3.12
1.01
1.01
1.14
1.00
1.02
1.02
3.07
3.10
3.07

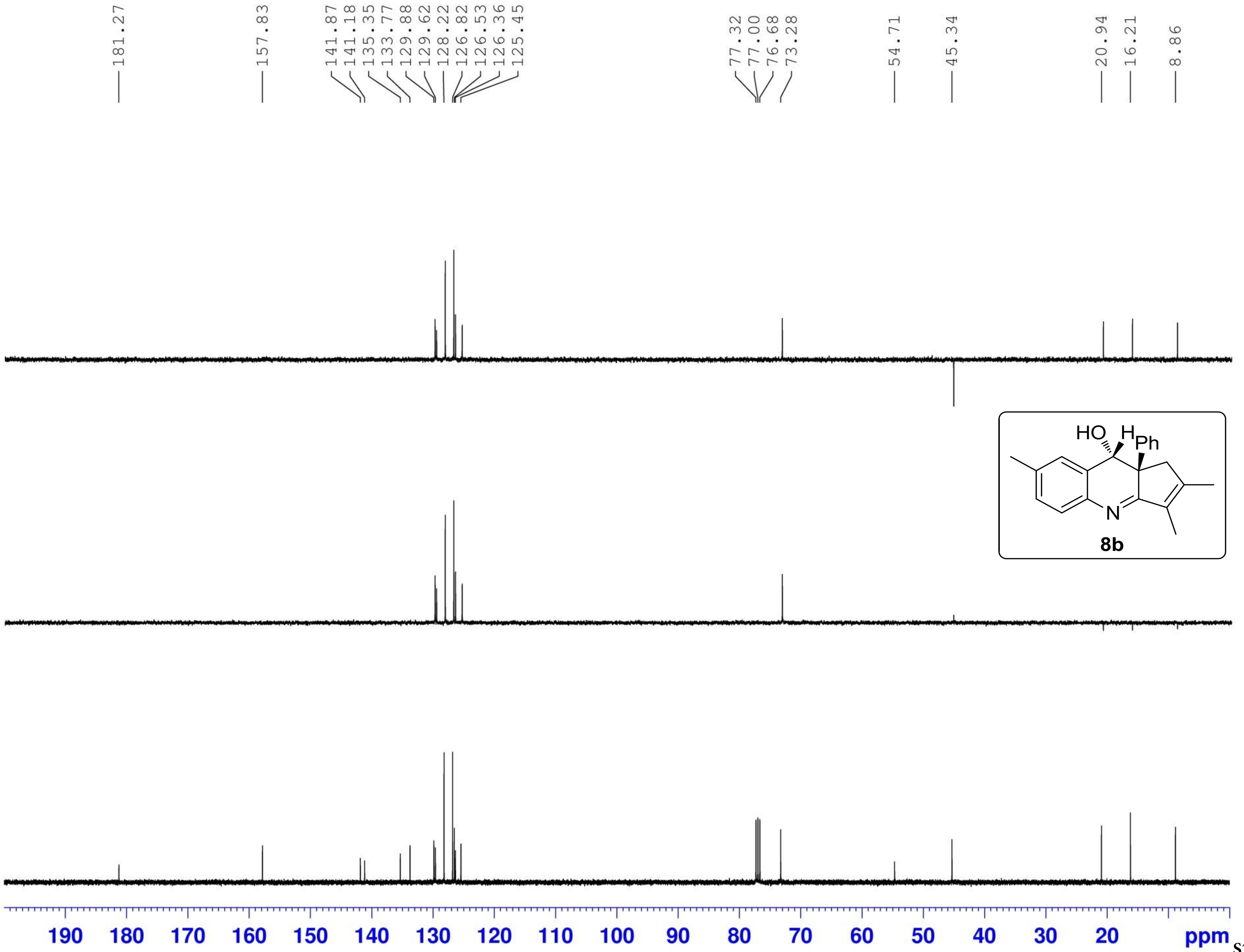
181.27
157.83
141.87
141.18
135.35
133.77
129.88
129.62
128.22
126.82
126.53
126.36
125.45
77.32
77.00
76.68
73.28
54.71
45.34
20.94
16.21
8.86
HO
H
Ph
N
8b
190
180
170
160
150
140
130
120
110
100
90
80
70
60
50
40
30
20
ppm

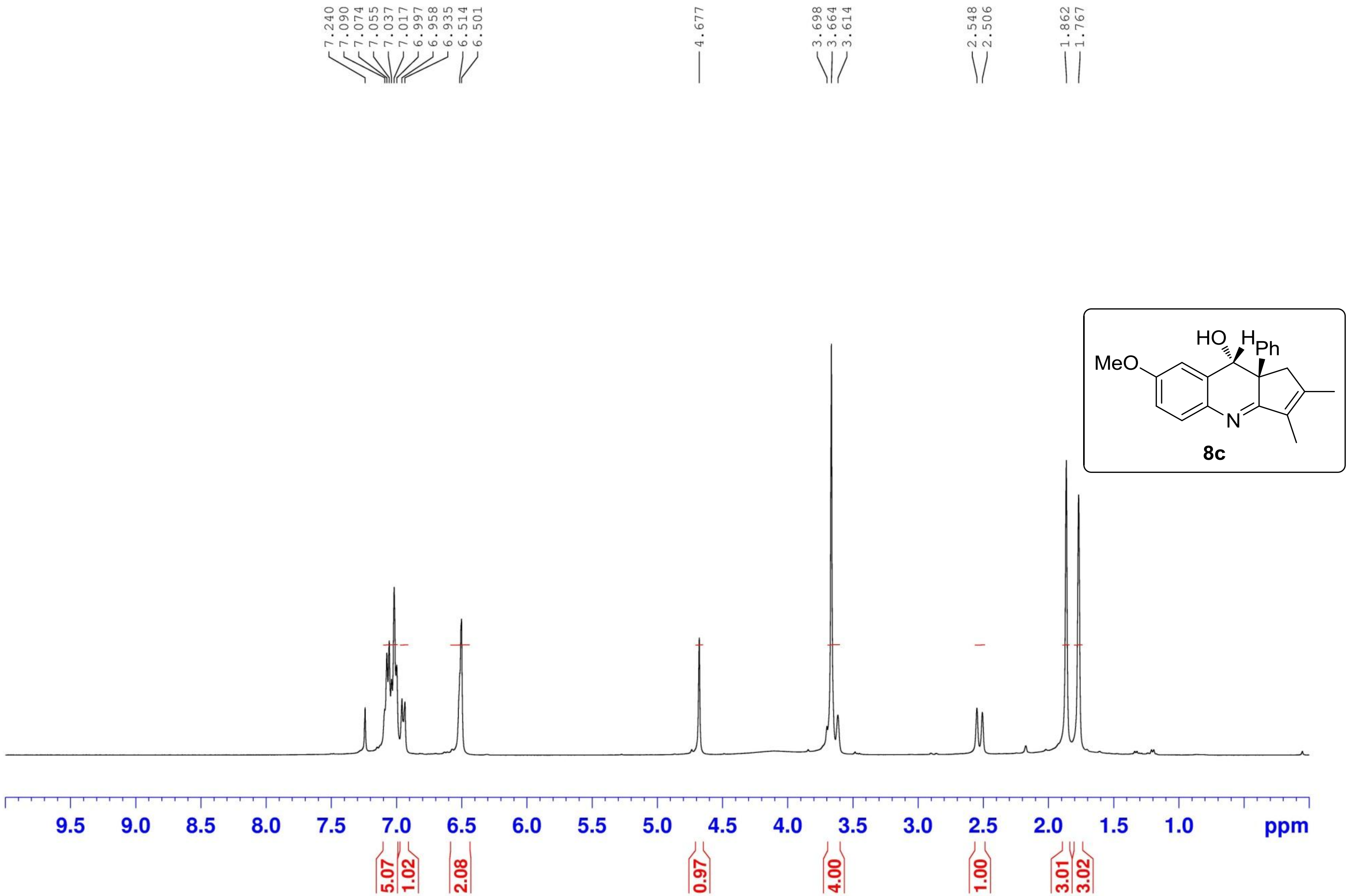
7.240
7.090
7.074
7.055
7.037
7.017
6.997
6.958
6.935
6.514
6.501
4.677
3.698
3.664
3.614
2.548
2.506
1.862
1.767
9.5
9.0
8.5
8.0
7.5
7.0
6.5
6.0
5.5
5.0
4.5
4.0
3.5
3.0
2.5
2.0
1.5
1.0
ppm
5.07
1.02
2.08
0.97
4.00
1.00
3.01
3.02
HO
H
Ph
MeO
N

8c

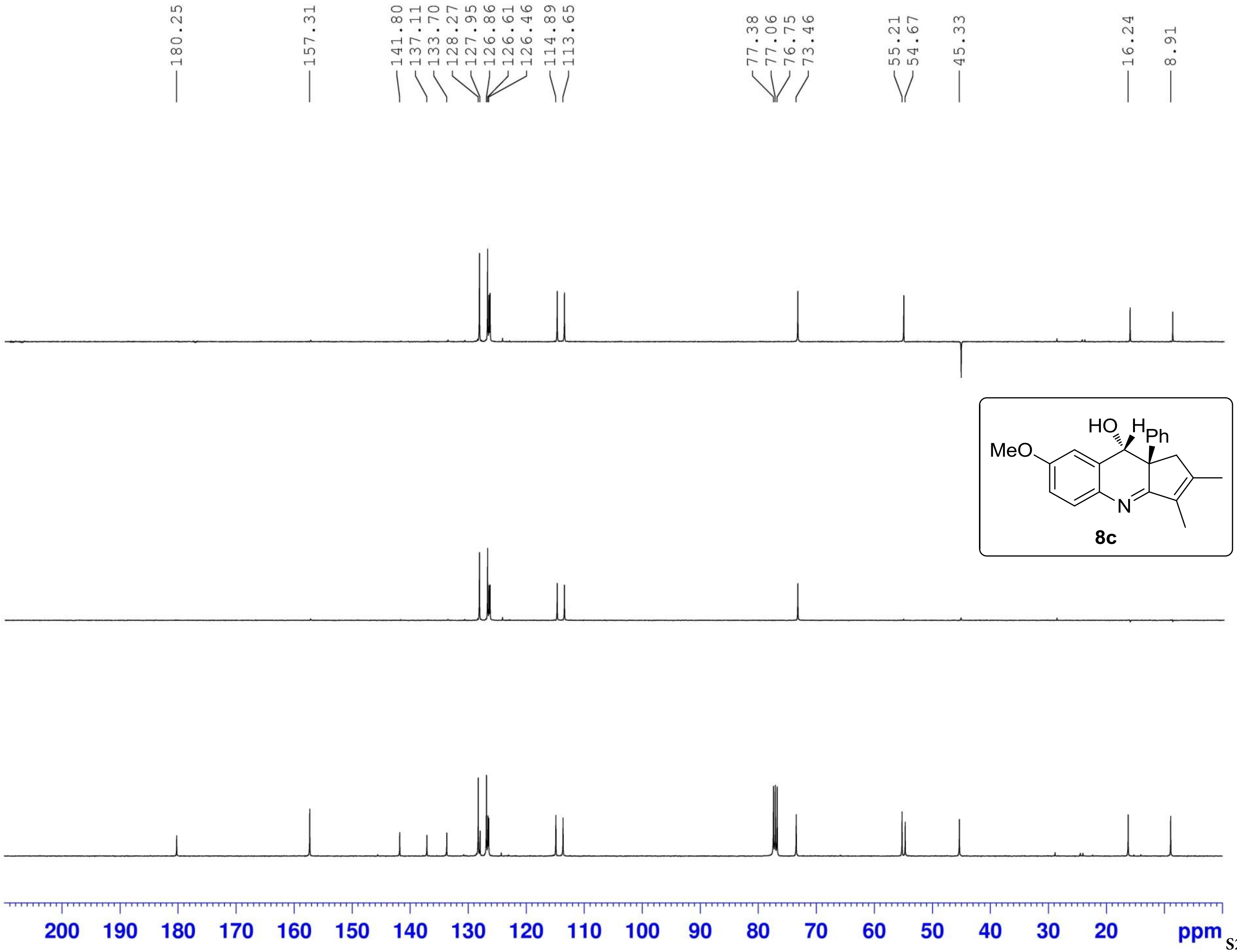
180.25
157.31
141.80
137.11
133.70
128.27
127.95
126.86
126.61
126.46
114.89
113.65
77.38
77.06
76.75
73.46
55.21
54.67
45.33
16.24
8.91
HO
H
Ph
MeO
N
8c
200 190 180 170 160 150 140 130 120 110 100 90 80 70 60 50 40 30 20
ppm

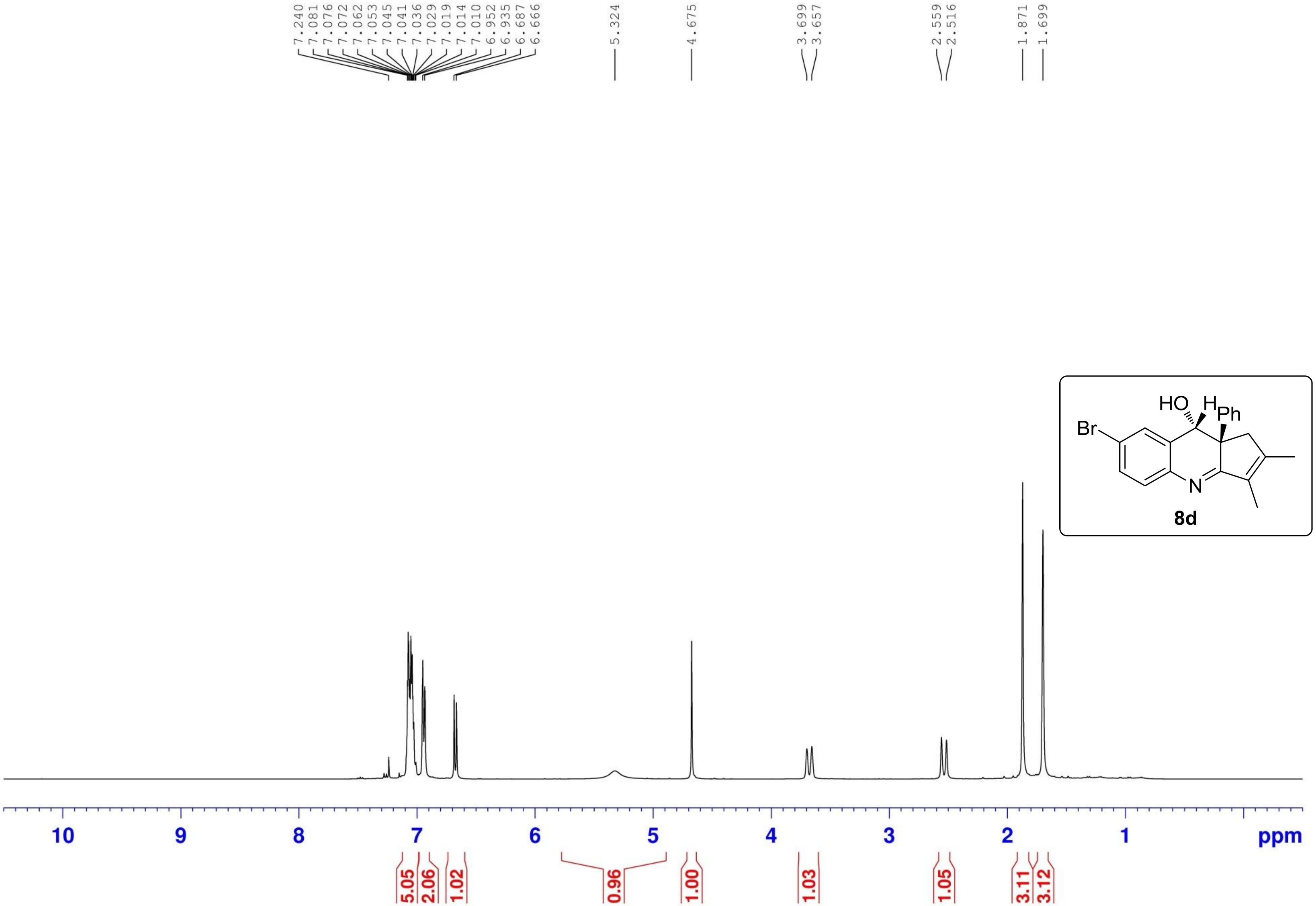
7.240
7.081
7.076
7.072
7.062
7.053
7.045
7.041
7.036
7.029
7.019
7.014
7.010
6.952
6.935
6.687
6.666
5.324
4.675
3.699
3.657
2.559
2.516
1.871
1.699
HO
H
Ph
Br
N
8d
10
9
8
7
6
5
4
3
2
1
ppm
5.05
2.06
1.02
0.96
1.00
1.03
1.05
3.11
3.12

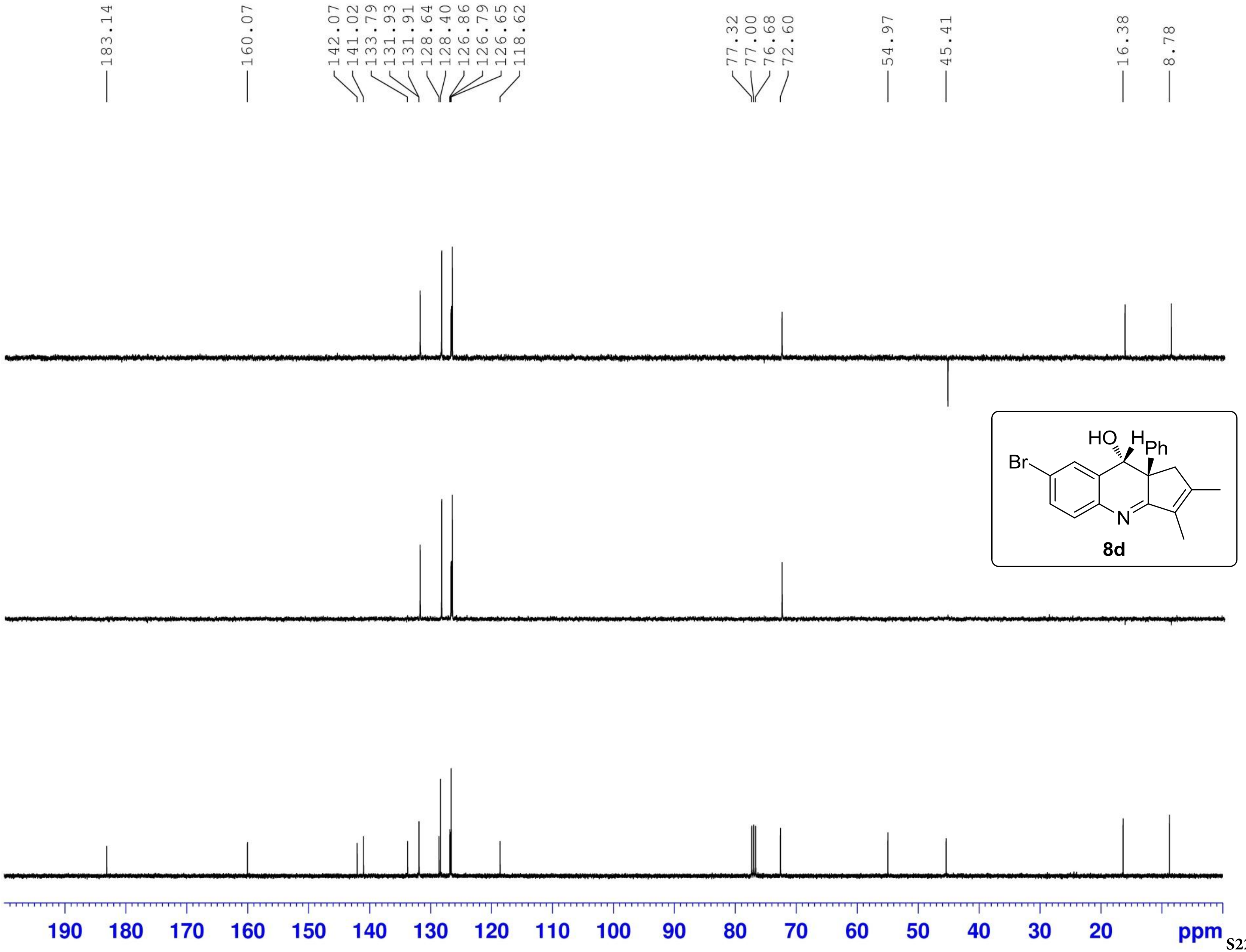
183.14
160.07
142.07
141.02
133.79
131.93
131.91
128.64
128.40
126.86
126.79
126.65
118.62
77.32
77.00
76.68
72.60
54.97
45.41
16.38
8.78
HO
H
Ph
Br
N
8d
190 180 170 160 150 140 130 120 110 100 90 80 70 60 50 40 30 20 ppm

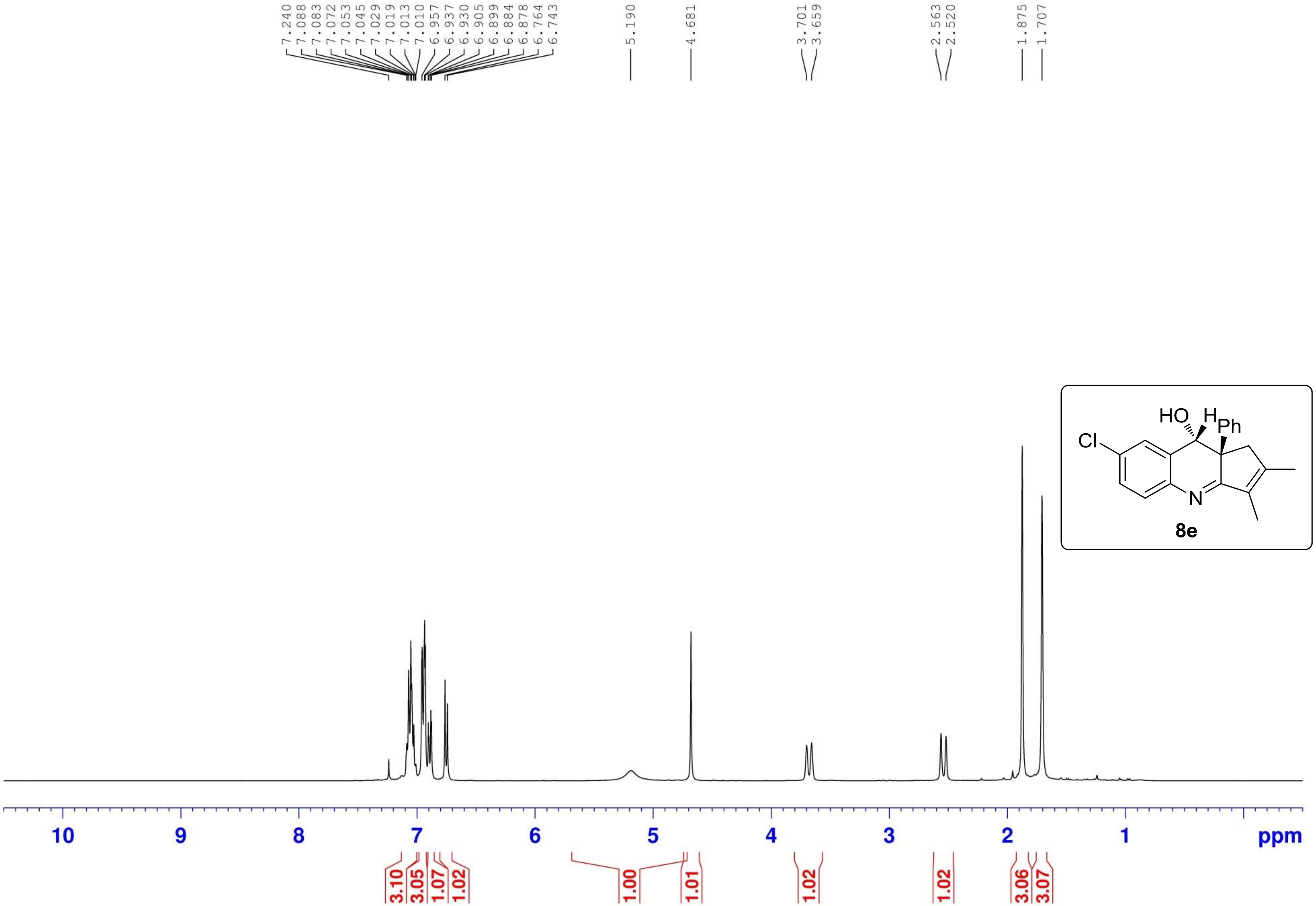
7.240
7.088
7.083
7.072
7.053
7.045
7.029
7.019
7.013
7.010
6.957
6.937
6.930
6.905
6.899
6.884
6.878
6.764
6.743
5.190
4.681
3.701
3.659
2.563
2.520
1.875
1.707
HO
H
Ph
Cl
N
8e
10
9
8
7
6
5
4
3
2
1
ppm
3.10
3.05
1.07
1.02
1.00
1.01
1.02
1.02
3.06
3.07

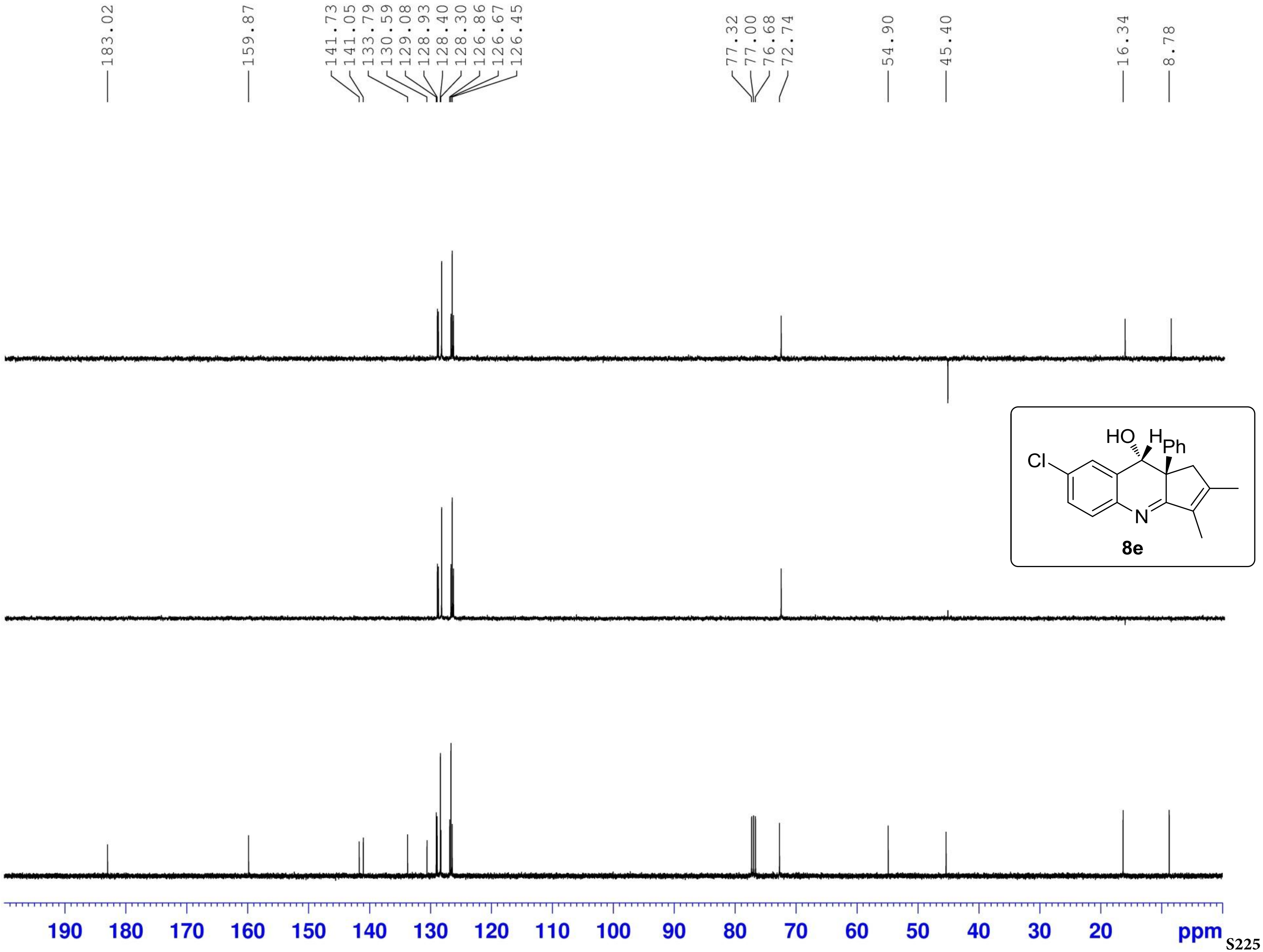
183.02
159.87
141.73
141.05
133.79
130.59
129.08
128.93
128.40
128.30
126.86
126.67
126.45
77.32
77.00
76.68
72.74
54.90
45.40
16.34
8.78
HO
H
Ph
Cl
N
8e
190 180 170 160 150 140 130 120 110 100 90 80 70 60 50 40 30 20 ppm

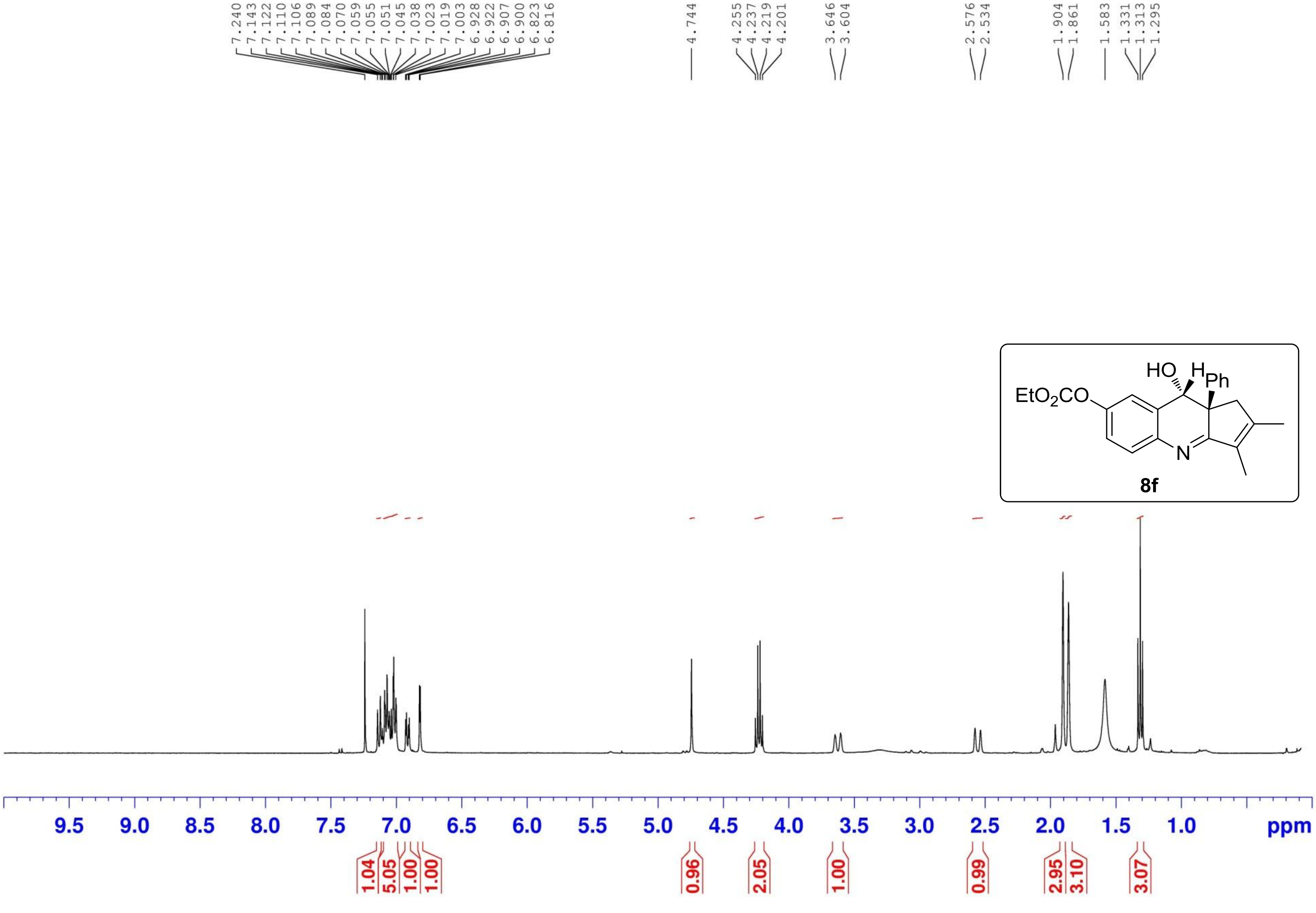
7.240
7.143
7.122
7.110
7.106
7.089
7.084
7.070
7.059
7.055
7.051
7.045
7.038
7.023
7.019
7.003
6.928
6.922
6.907
6.900
6.823
6.816
4.744
4.255
4.237
4.219
4.201
3.646
3.604
2.576
2.534
1.904
1.861
1.583
1.331
1.313
1.295
HO
H
Ph
EtO2CO
N
9.5
9.0
8.5
8.0
7.5
7.0
6.5
6.0
5.5
5.0
4.5
4.0
3.5
3.0
2.5
2.0
1.5
1.0
ppm
1.04
5.05
1.00
1.00
0.96
2.05
1.00
0.99
2.95
3.10
3.07

8f

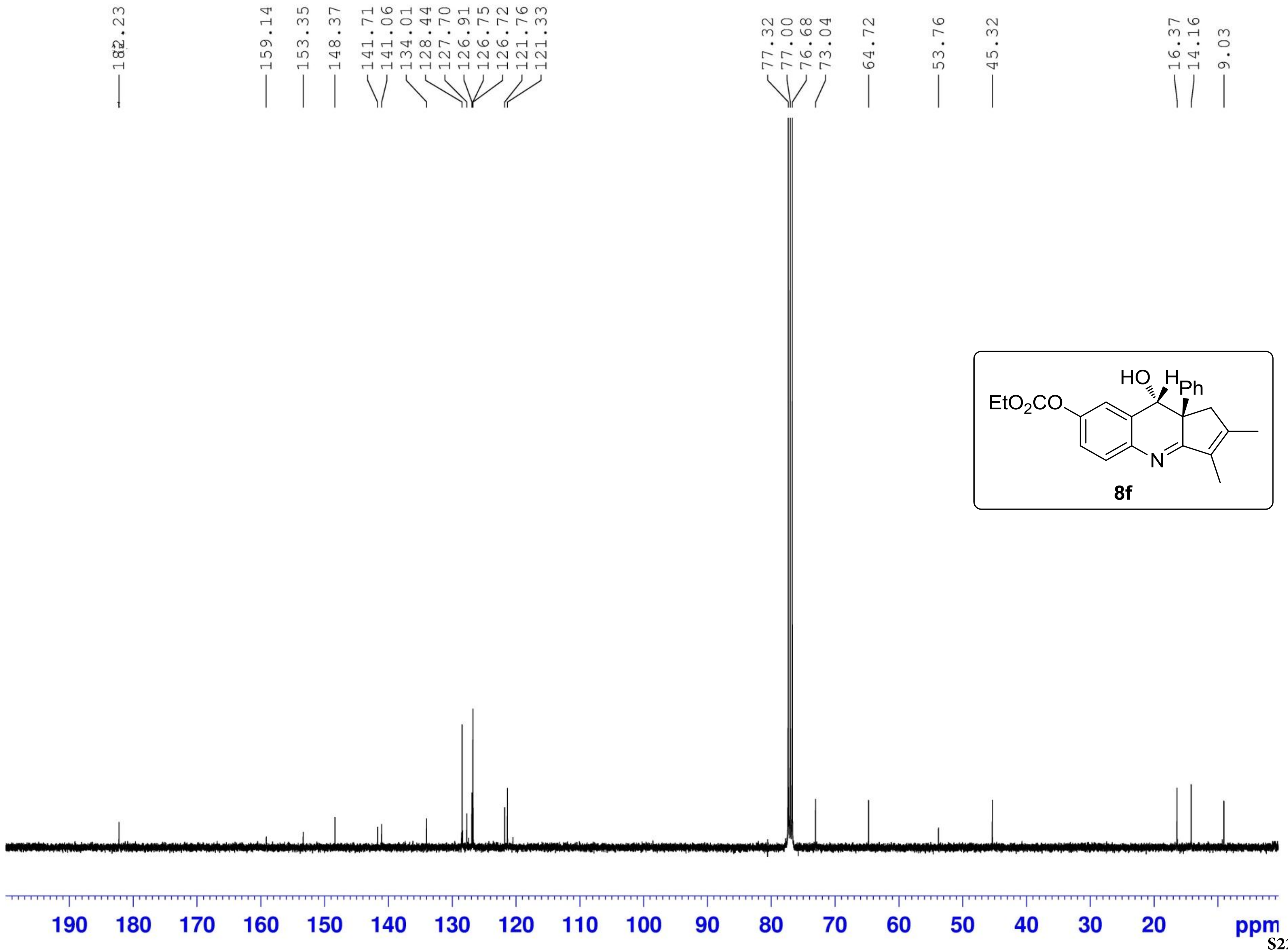
182.23
159.14
153.35
148.37
141.71
141.06
134.01
128.44
127.70
126.91
126.75
126.72
121.76
121.33
77.32
77.00
76.68
73.04
64.72
53.76
45.32
16.37
14.16
9.03
HO
H
Ph
EtO_2CO
N
8f
190 180 170 160 150 140 130 120 110 100 90 80 70 60 50 40 30 20
ppm

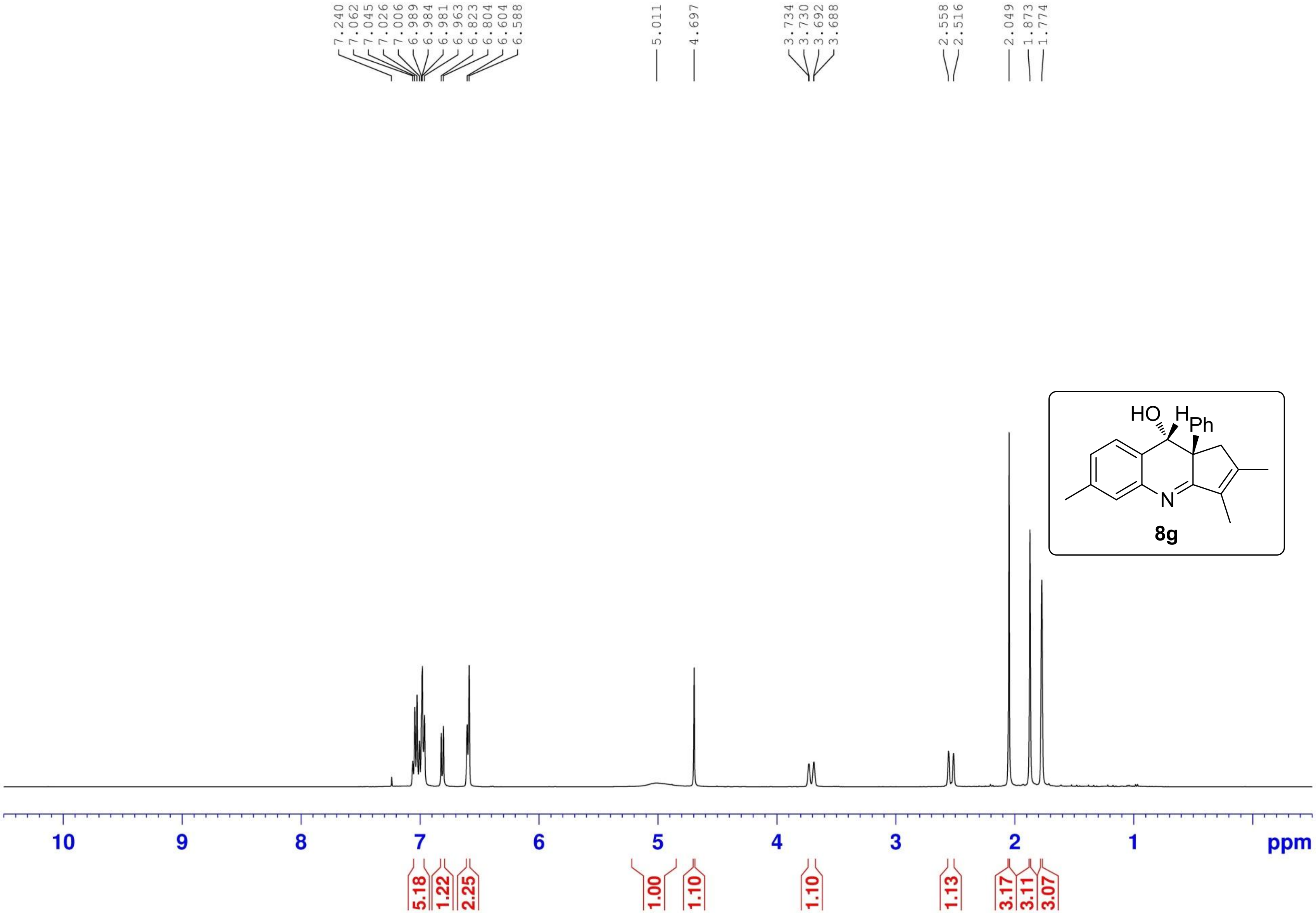
7.240
7.062
7.045
7.026
7.006
6.989
6.984
6.981
6.963
6.823
6.804
6.604
6.588
5.011
4.697
3.734
3.730
3.692
3.688
2.558
2.516
2.049
1.873
1.774
HO
H
Ph
N
8g
10
9
8
7
6
5
4
3
2
1
ppm
5.18
1.22
2.25
1.00
1.10
1.10
1.13
3.17
3.11
3.07

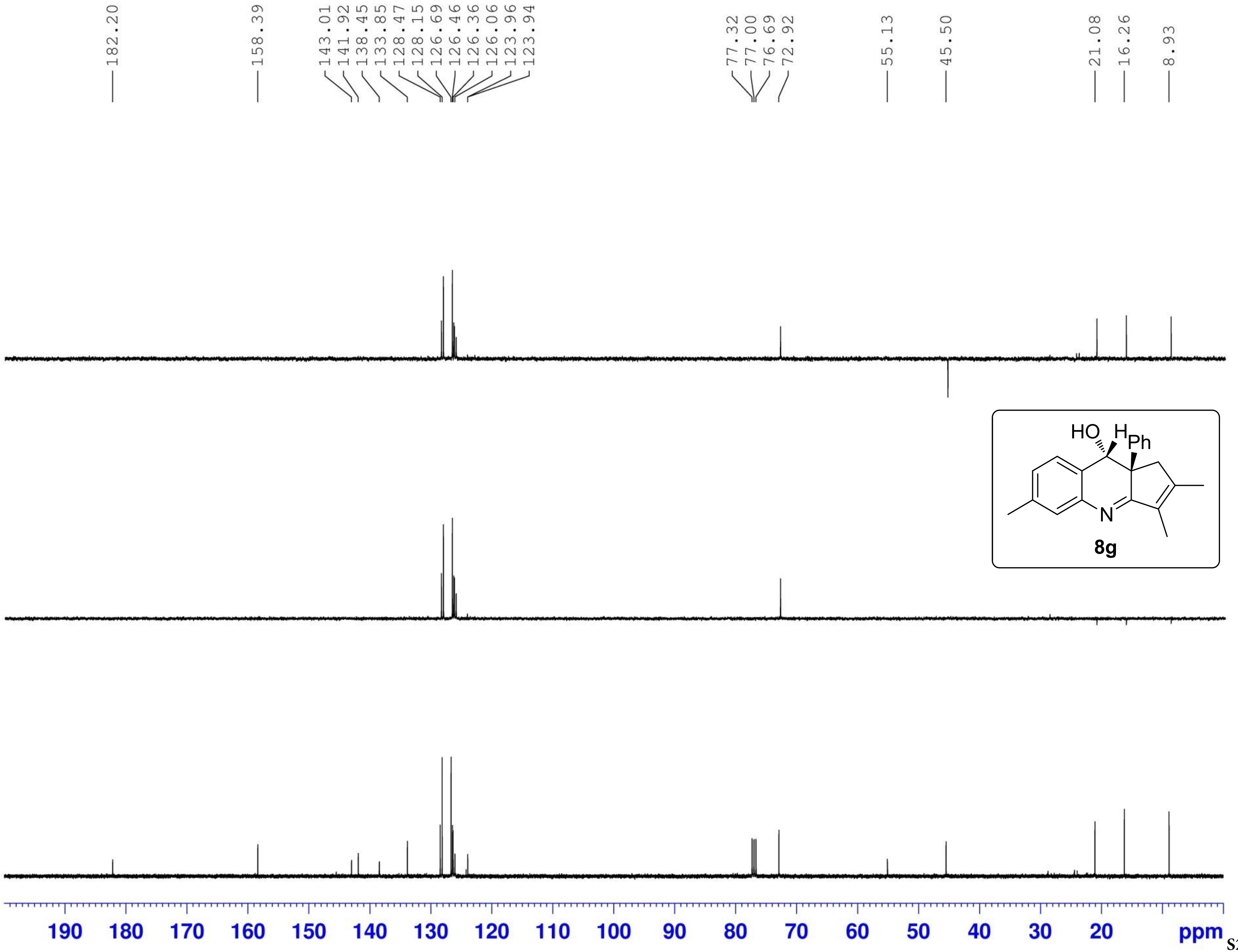
182.20
158.39
143.01
141.92
138.45
133.85
128.47
128.15
126.69
126.46
126.36
126.06
123.96
123.94
77.32
77.00
76.69
72.92
55.13
45.50
21.08
16.26
8.93
HO
H
Ph
N
8g
190 180 170 160 150 140 130 120 110 100 90 80 70 60 50 40 30 20 ppm

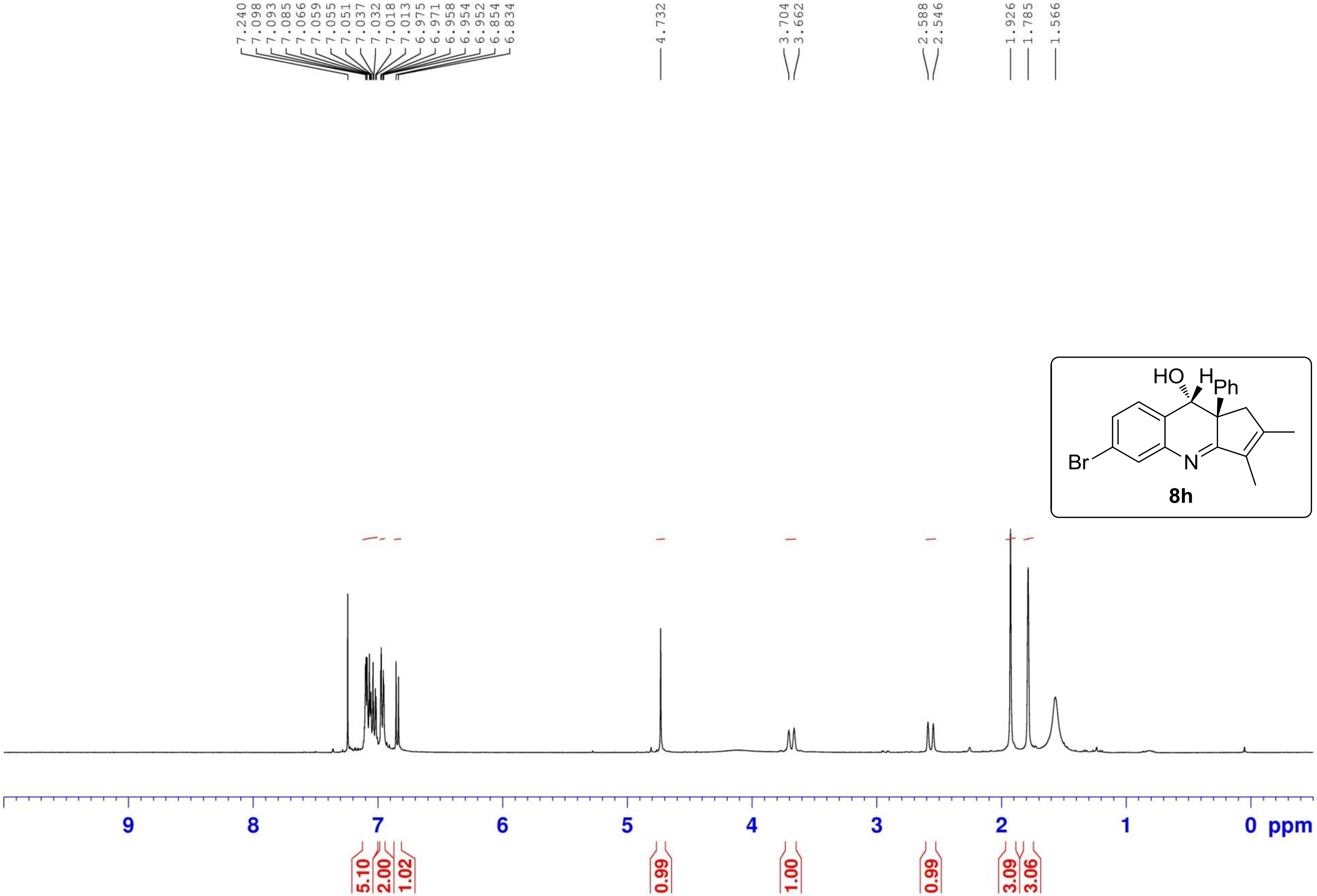
7.240
7.098
7.093
7.085
7.066
7.059
7.055
7.051
7.037
7.032
7.018
7.013
6.975
6.971
6.958
6.954
6.952
6.854
6.834
4.732
3.704
3.662
2.588
2.546
1.926
1.785
1.566
HO
H
Ph
Br
N
8h
9
8
7
6
5
4
3
2
1
0 ppm
5.10
2.00
1.02
0.99
1.00
0.99
3.09
3.06

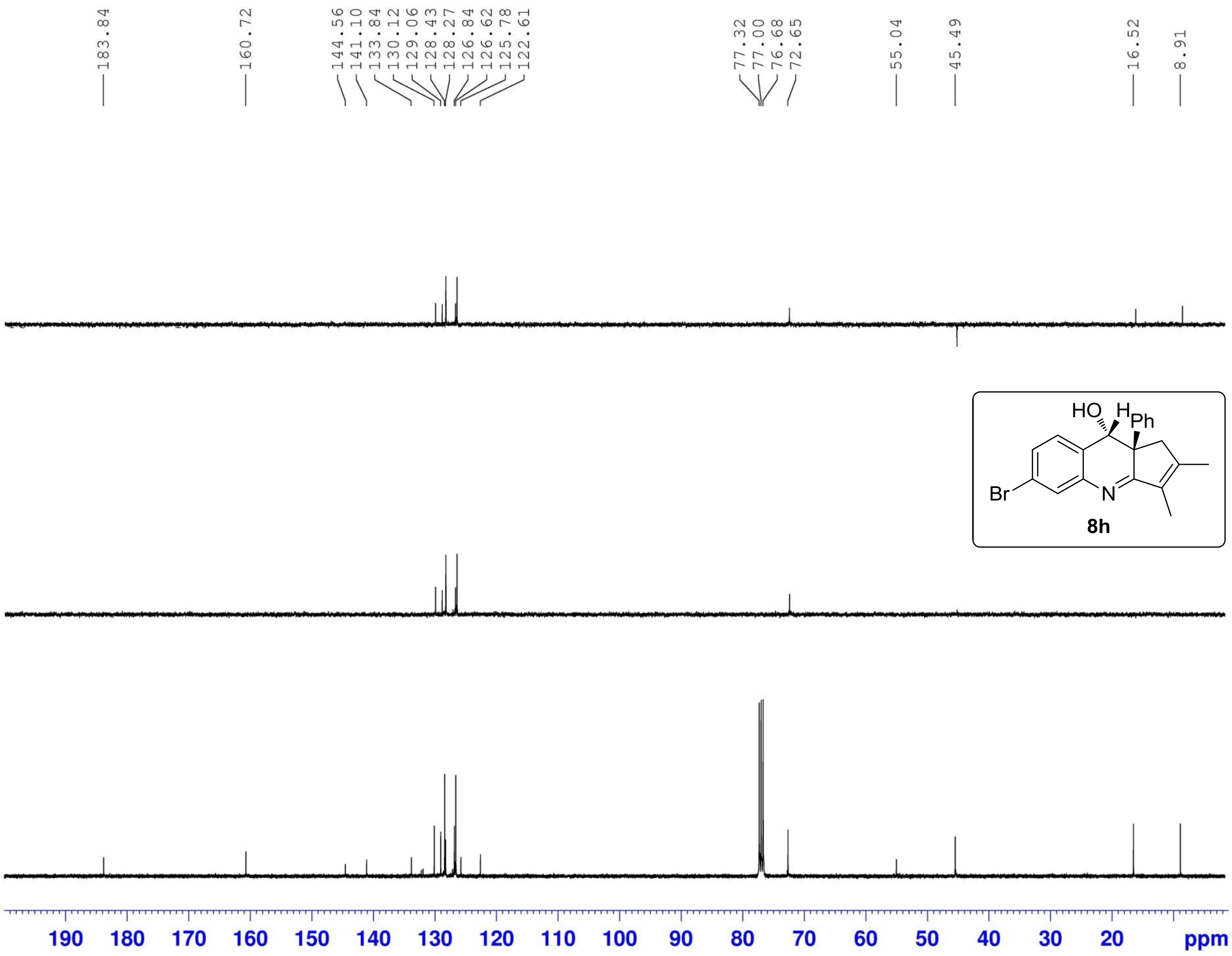
183.84
160.72
144.56
141.10
133.84
130.12
129.06
128.43
128.27
126.84
126.62
125.78
122.61
77.32
77.00
76.68
72.65
55.04
45.49
16.52
8.91
HO
H
Ph
Br
N
8h
190
180
170
160
150
140
130
120
110
100
90
80
70
60
50
40
30
20
ppm

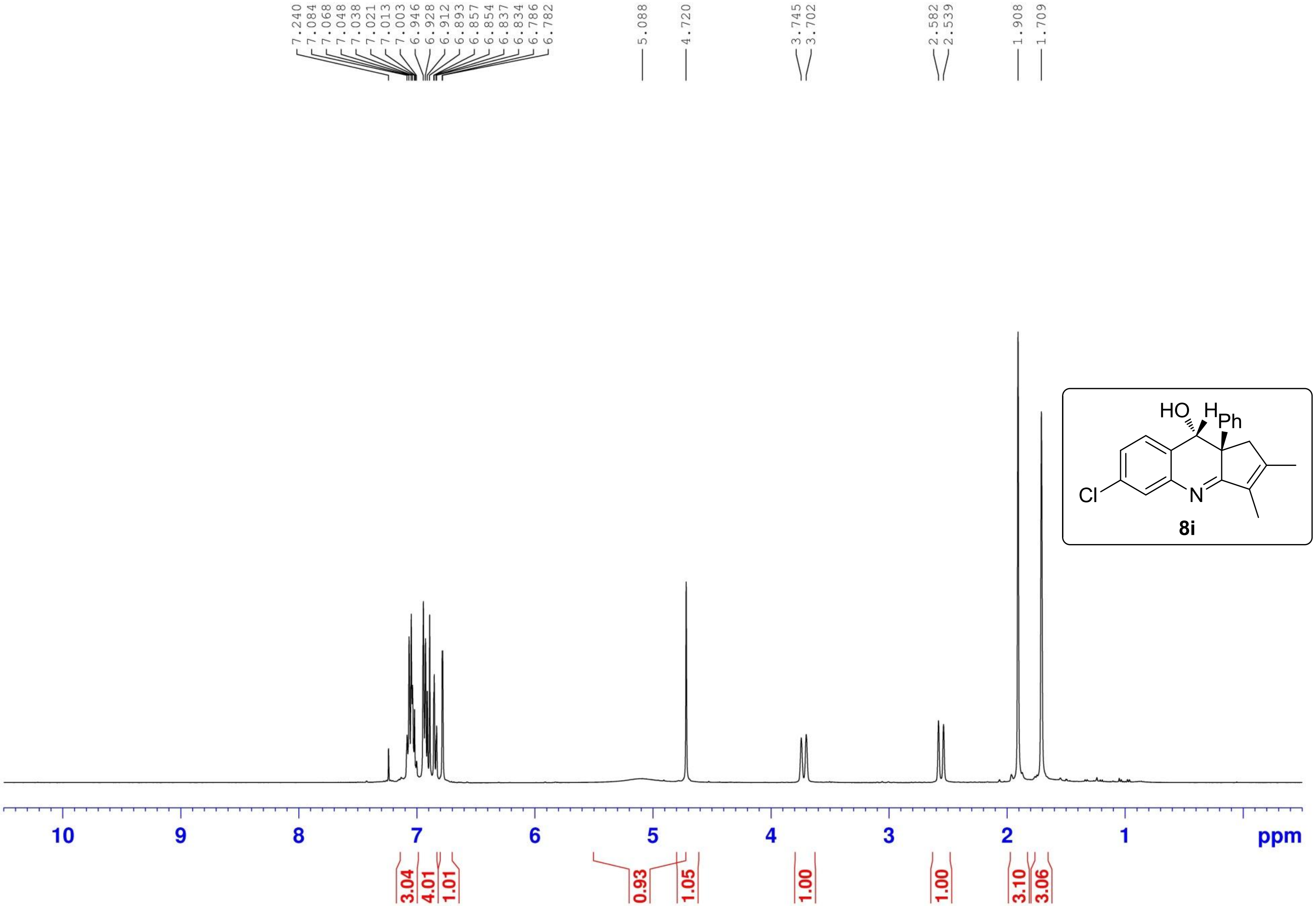
7.240
7.084
7.068
7.048
7.038
7.021
7.013
7.003
6.946
6.928
6.912
6.893
6.857
6.854
6.837
6.834
6.786
6.782
5.088
4.720
3.745
3.702
2.582
2.539
1.908
1.709
HO
H
Ph
Cl
N
8i
10
9
8
7
6
5
4
3
2
1
ppm
3.04
4.01
1.01
0.93
1.05
1.00
1.00
3.10
3.06

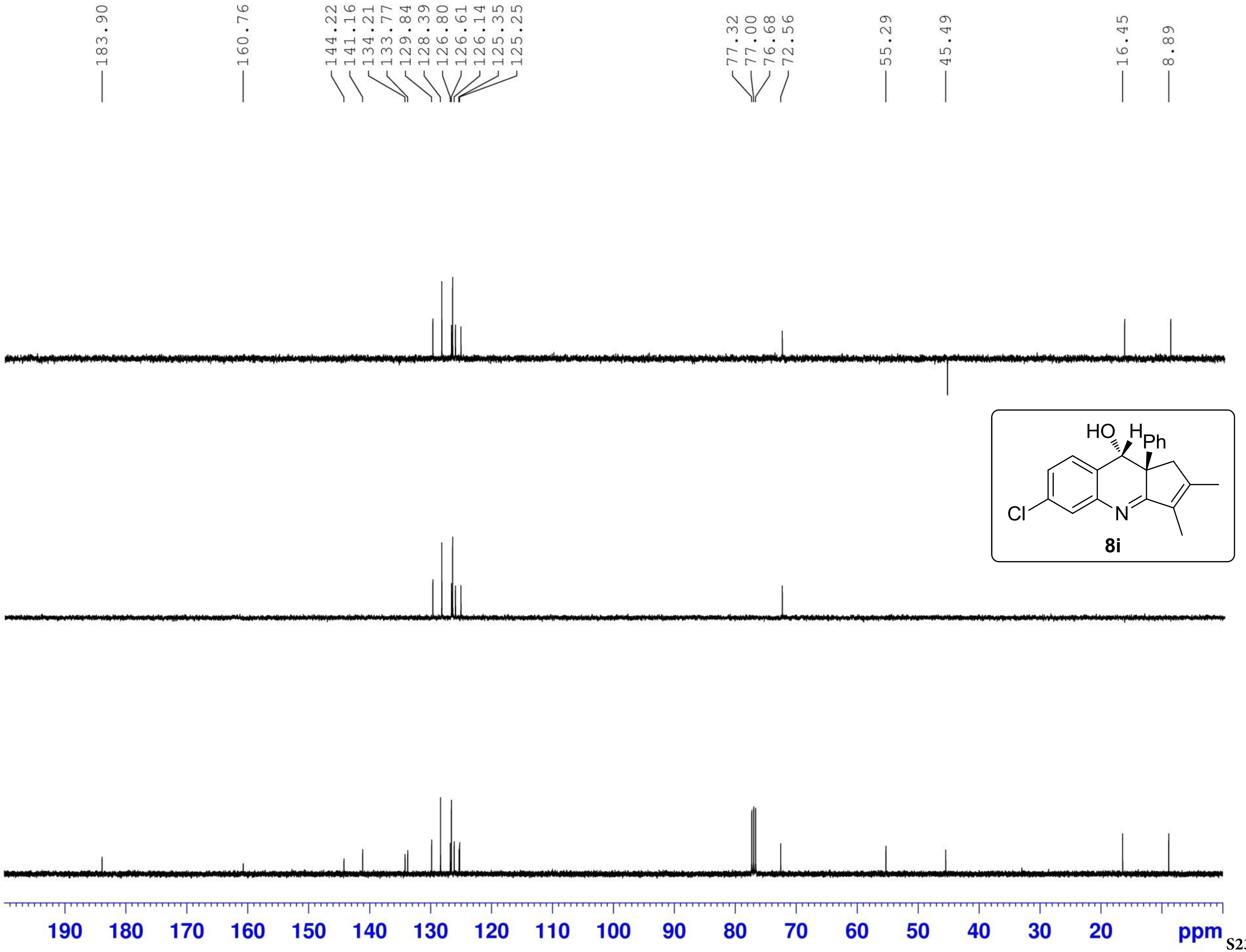
183.90
160.76
144.22
141.16
134.21
133.77
129.84
128.39
126.80
126.61
126.14
125.35
125.25
77.32
77.00
76.68
72.56
55.29
45.49
16.45
8.89
HO
H
Ph
Cl
N
8i
190
180
170
160
150
140
130
120
110
100
90
80
70
60
50
40
30
20
ppm

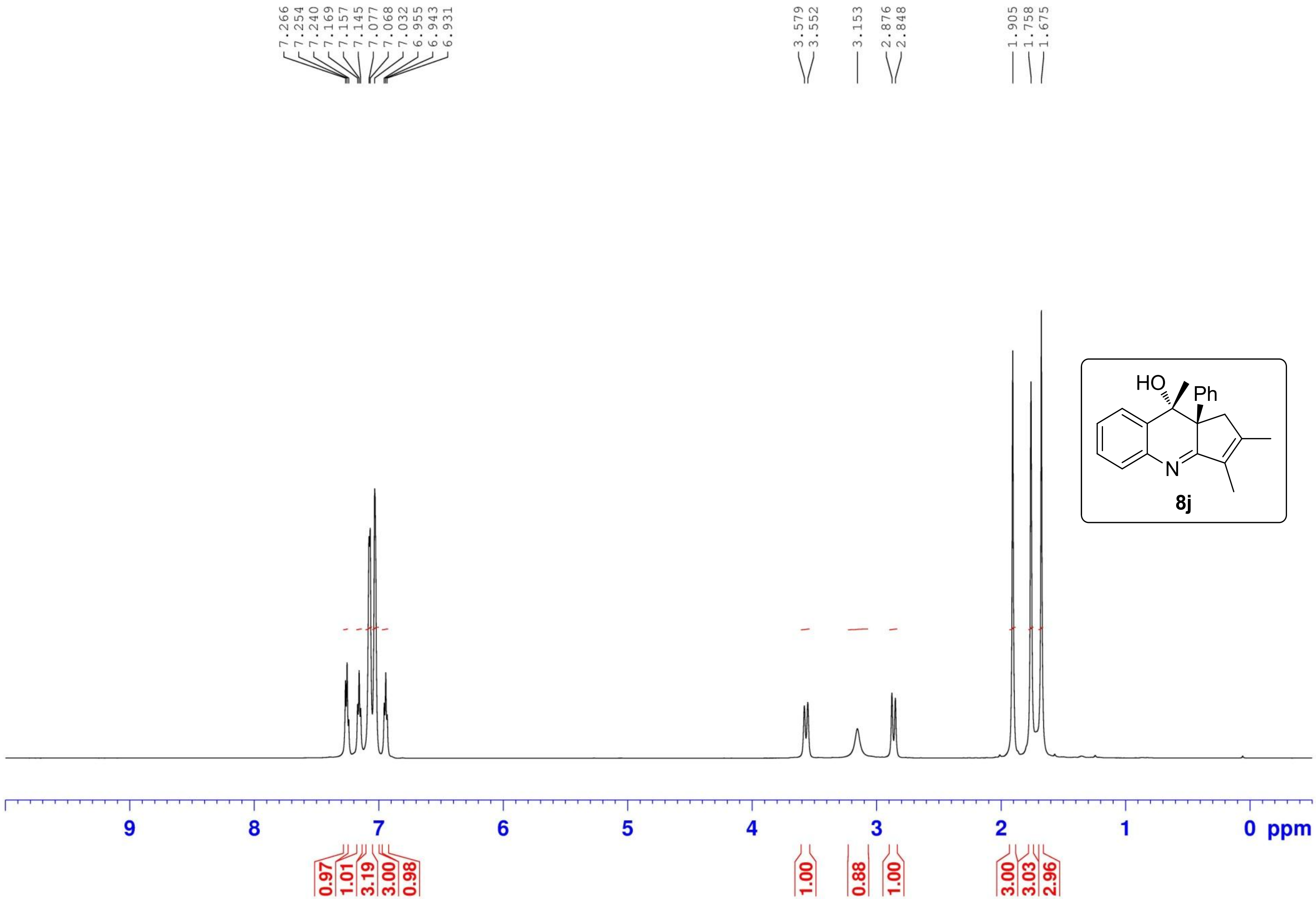
7.266
7.254
7.240
7.169
7.157
7.145
7.077
7.068
7.032
6.955
6.943
6.931
3.579
3.552
3.153
2.876
2.848
1.905
1.758
1.675
HO
Ph
N
8j
9
8
7
6
5
4
3
2
1
0 ppm
0.97
1.01
3.19
3.00
0.98
1.00
0.88
1.00
3.00
3.03
2.96

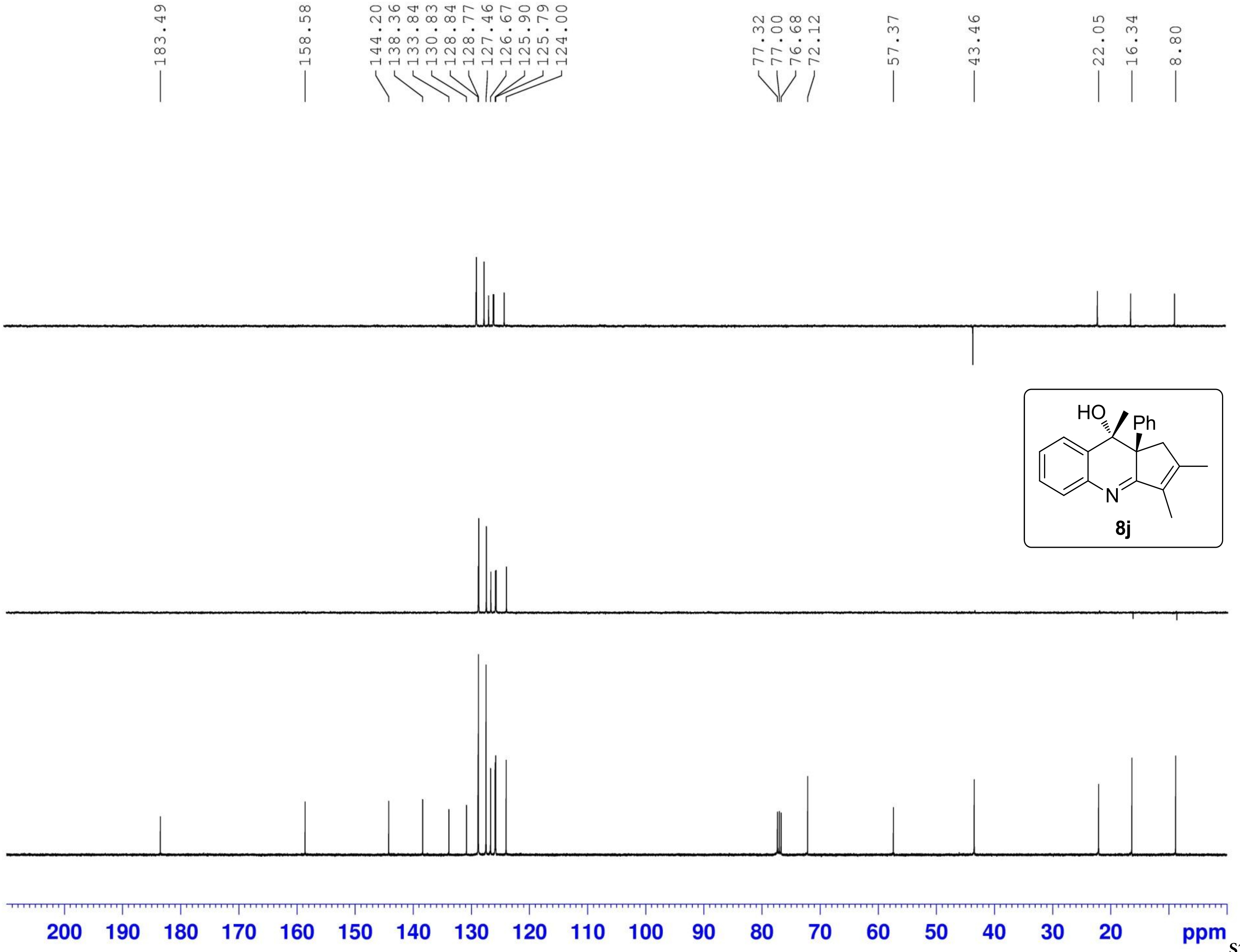
183.49
158.58
144.20
138.36
133.84
130.83
128.84
128.77
127.46
126.67
125.90
125.79
124.00
77.32
77.00
76.68
72.12
57.37
43.46
22.05
16.34
8.80
HO
Ph
N
8j
200 190 180 170 160 150 140 130 120 110 100 90 80 70 60 50 40 30 20 ppm

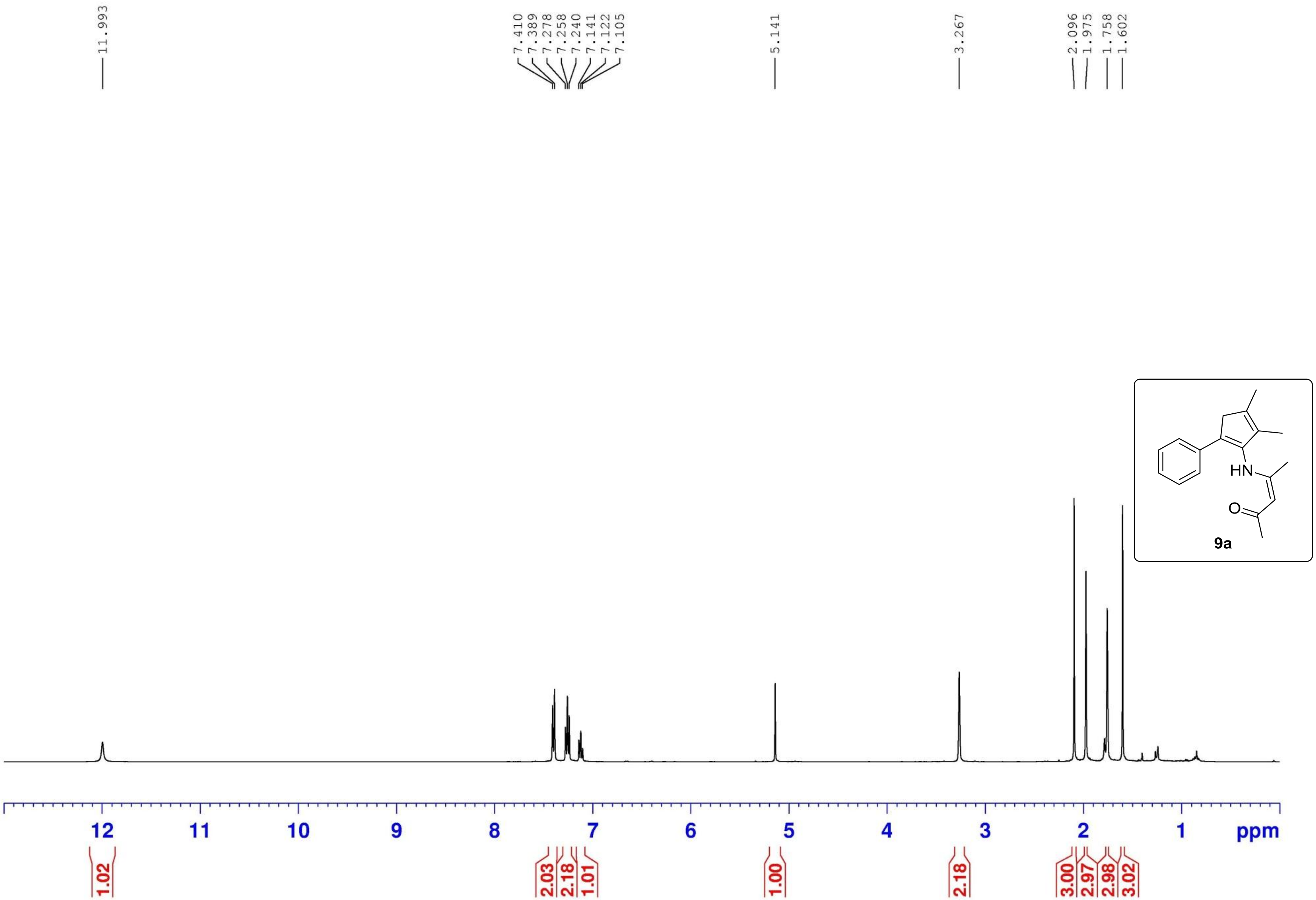
11.993
7.410
7.389
7.278
7.258
7.240
7.141
7.122
7.105
5.141
3.267
2.096
1.975
1.758
1.602
HN
O
9a
12
11
10
9
8
7
6
5
4
3
2
1
ppm
1.02
2.03
2.18
1.01
1.00
2.18
3.00
2.97
2.98
3.02

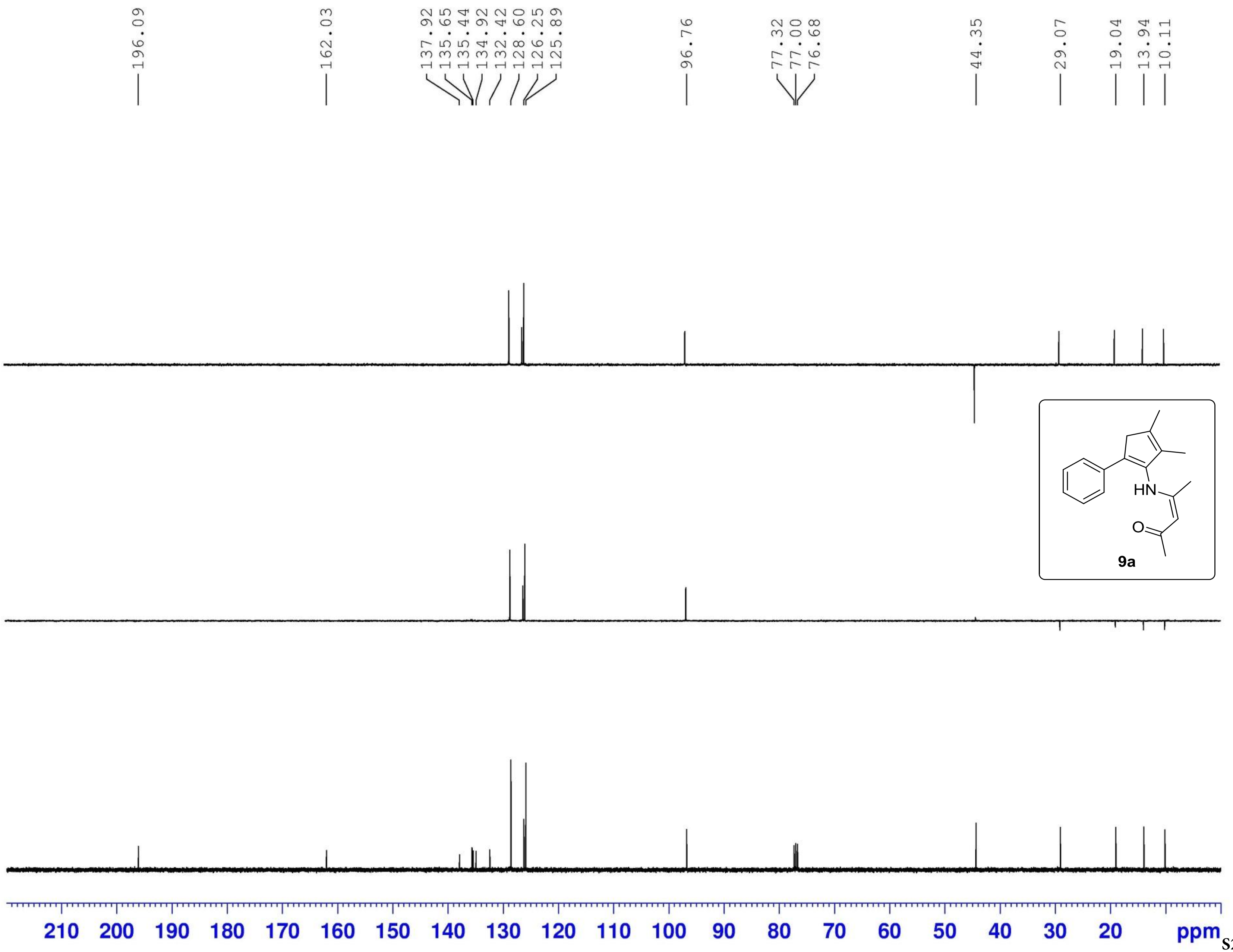
196.09
162.03
137.92
135.65
135.44
134.92
132.42
128.60
126.25
125.89
96.76
77.32
77.00
76.68
44.35
29.07
19.04
13.94
10.11
HN
O
9a
210 200 190 180 170 160 150 140 130 120 110 100 90 80 70 60 50 40 30 20 ppm

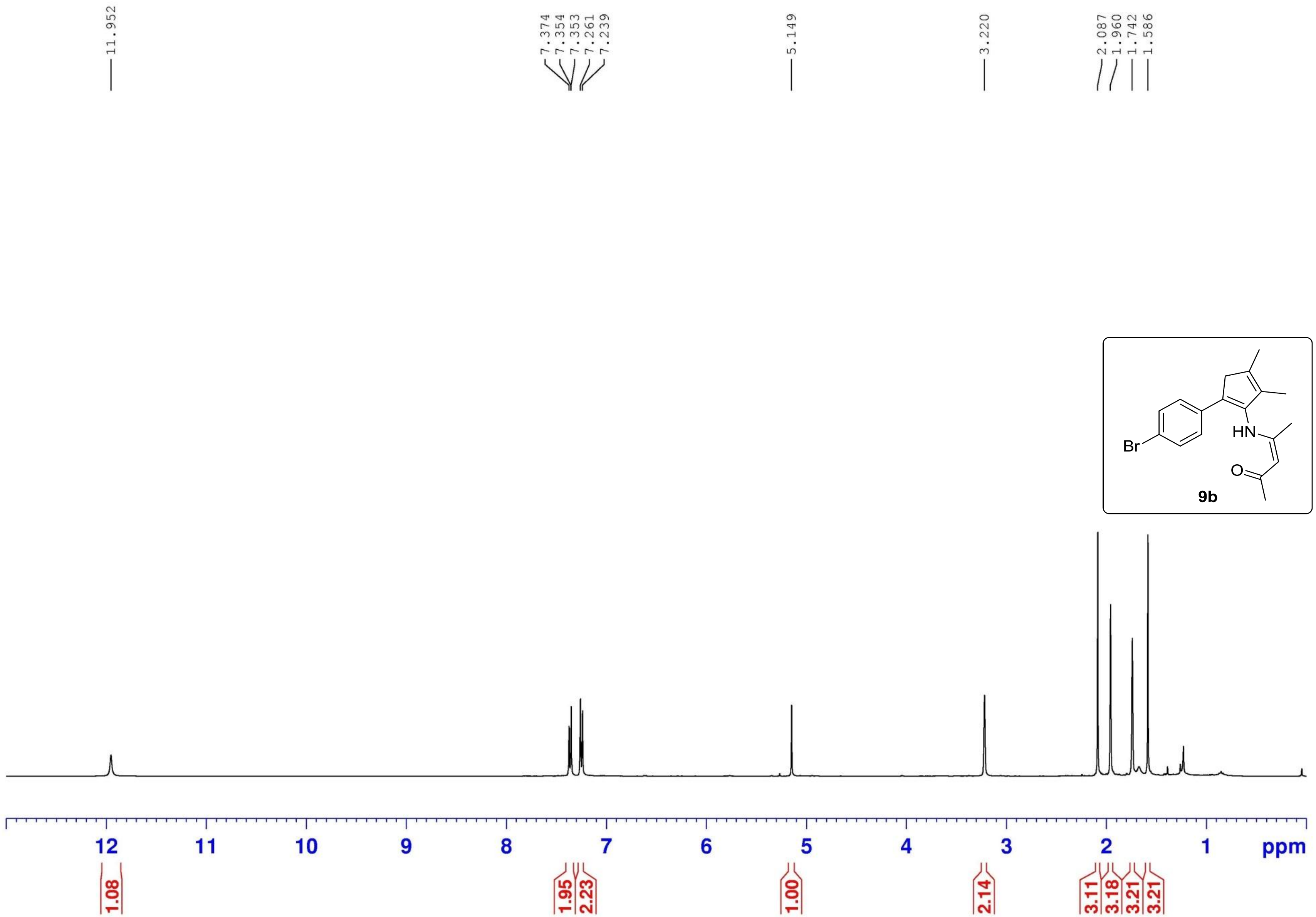

11.952
7.374
7.354
7.353
7.261
7.239
5.149
3.220
2.087
1.960
1.742
1.586
Br
HN
O
9b
12
11
10
9
8
7
6
5
4
3
2
1
ppm
1.08
1.95
2.23
1.00
2.14
3.11
3.18
3.21
3.21

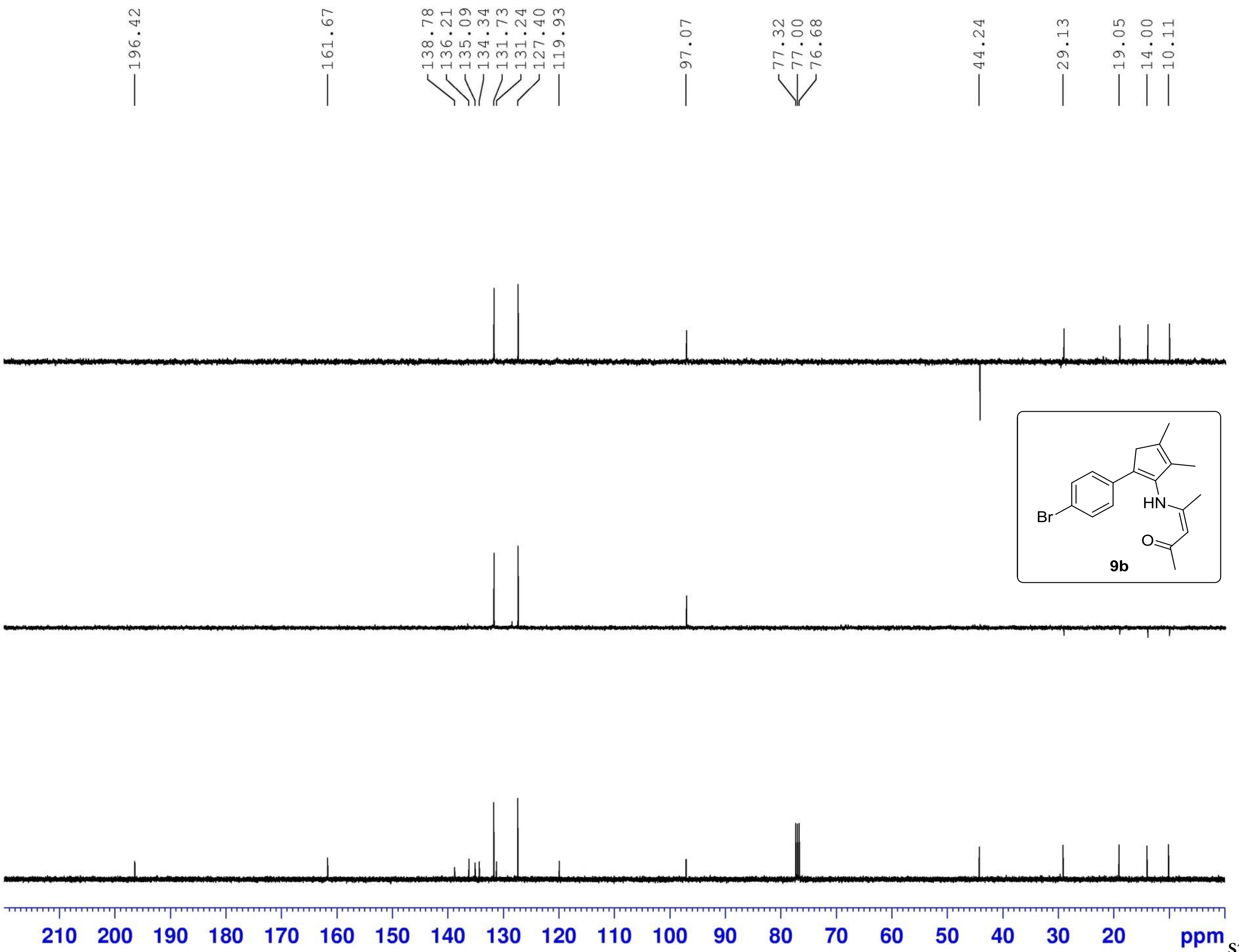

196.42
161.67
138.78
136.21
135.09
134.34
131.73
131.24
127.40
119.93
97.07
77.32
77.00
76.68
44.24
29.13
19.05
14.00
10.11
Br
HN
O
9b
210 200 190 180 170 160 150 140 130 120 110 100 90 80 70 60 50 40 30 20 ppm

^{1}H NOE of compound **5a-H**

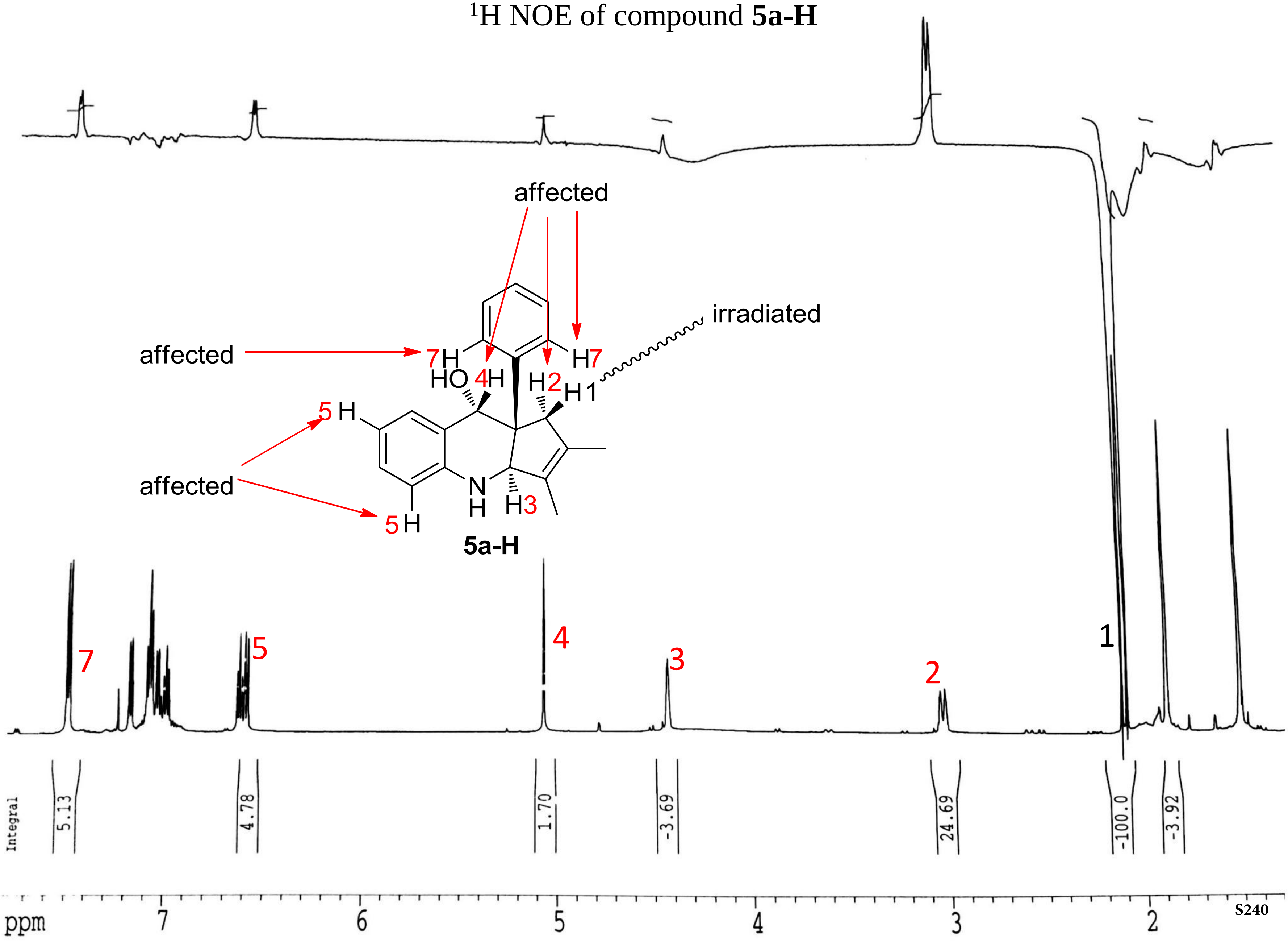

^{1}H NOE of compound **5a-H**

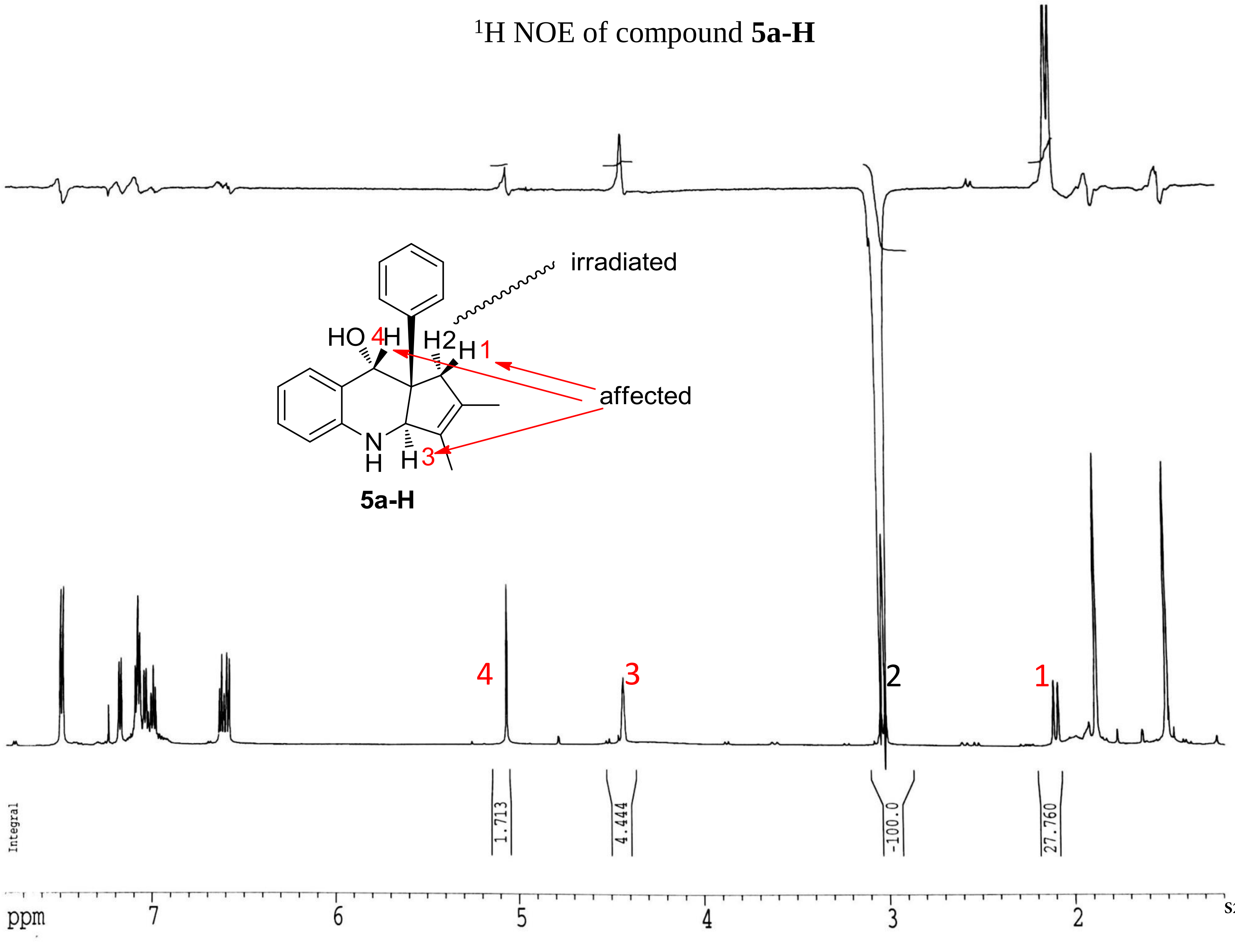

[1]H NOE of compound **5a-H**

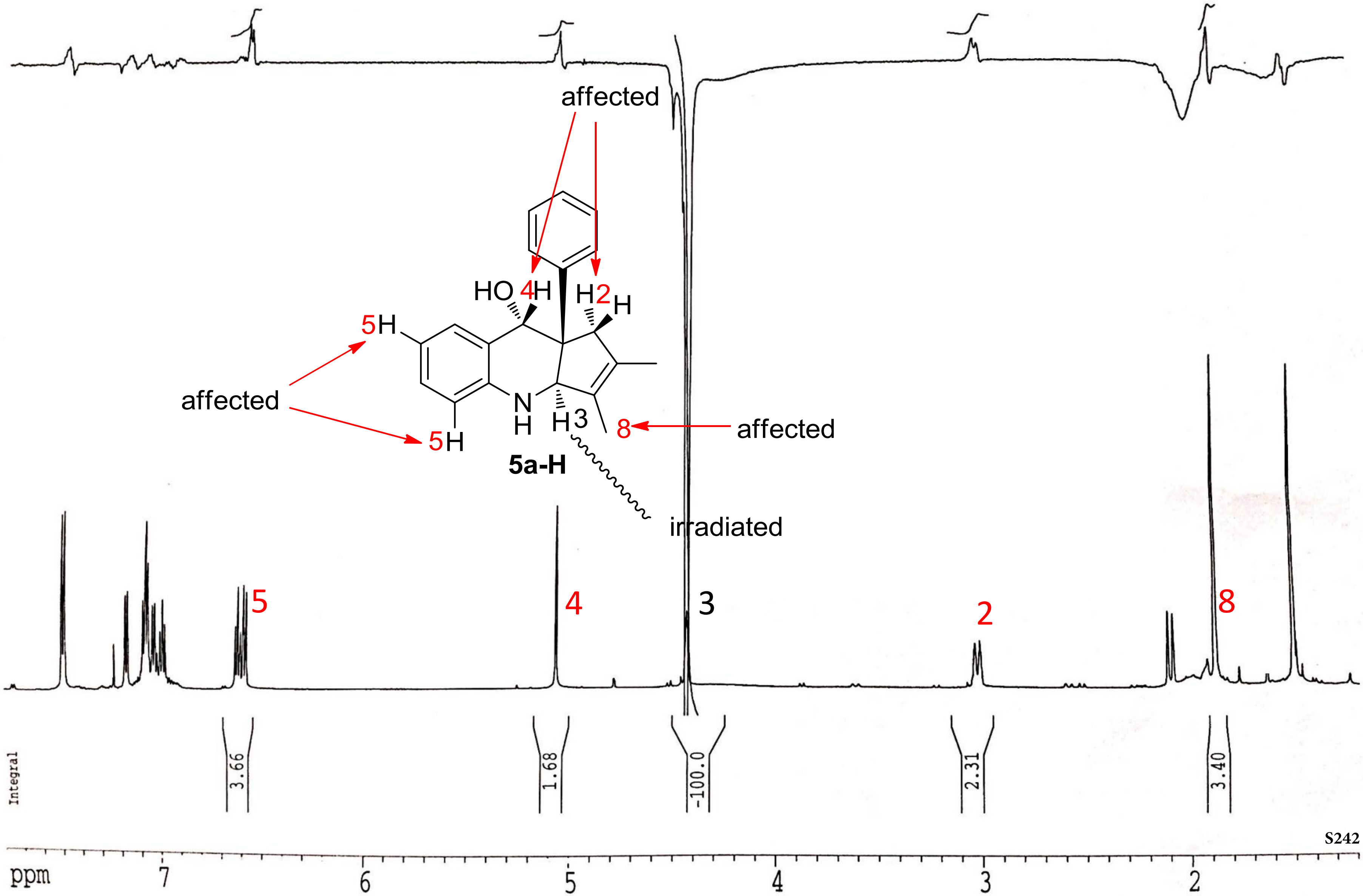

^{1}H NOE of compound **5a-H**

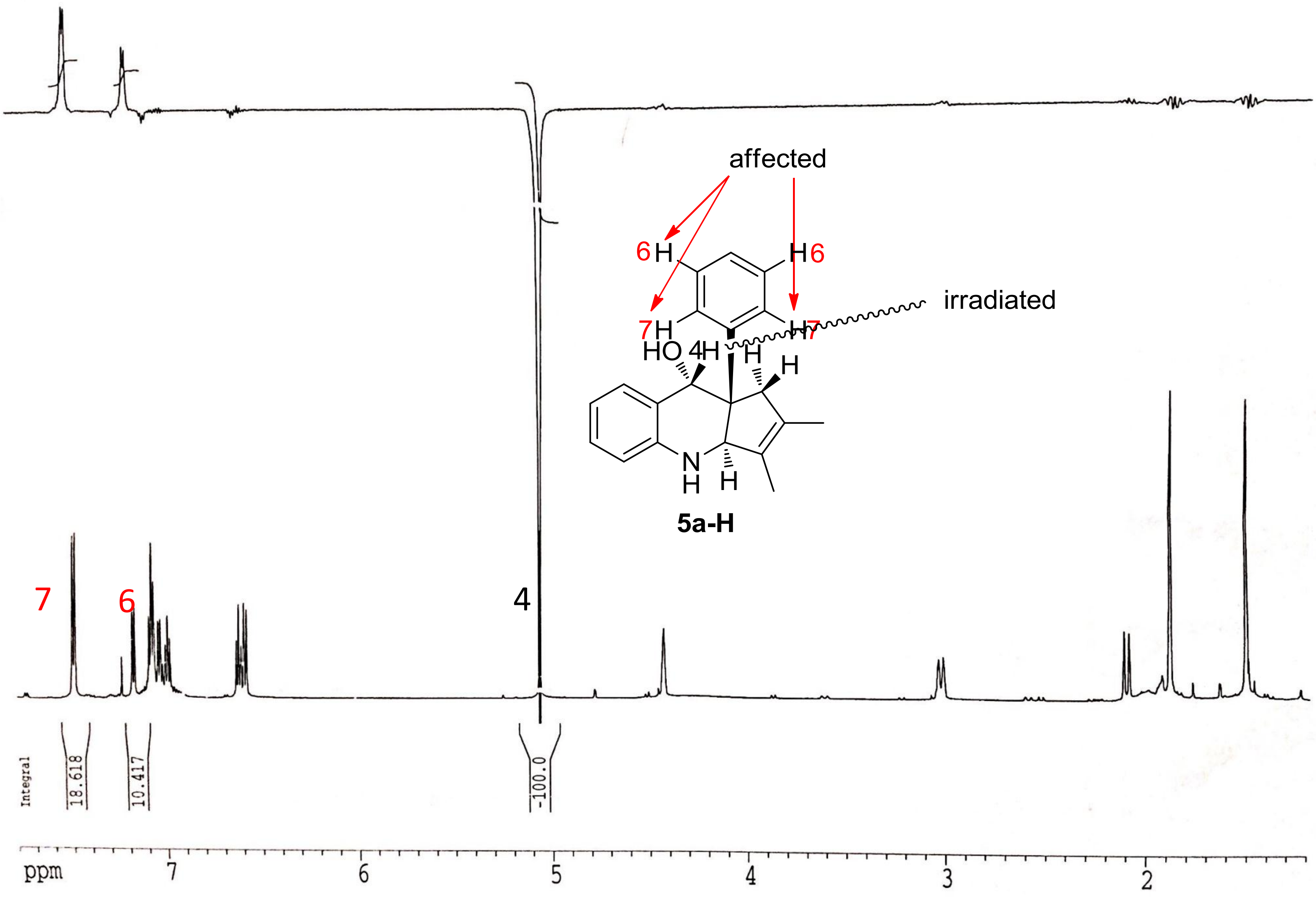

[1]H NOE of compound **5a-H**

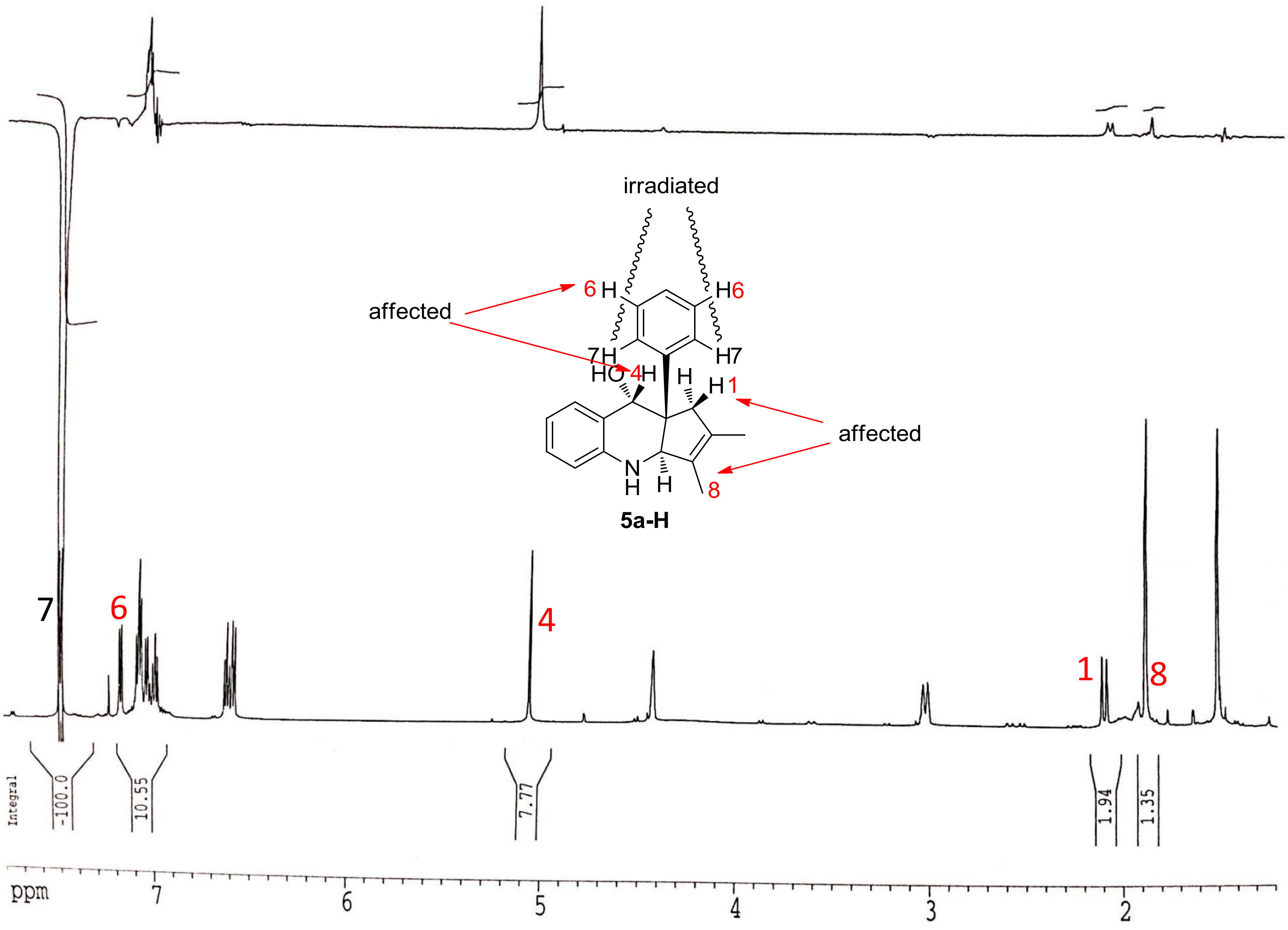

[1]H NOE of compound **5a-O**

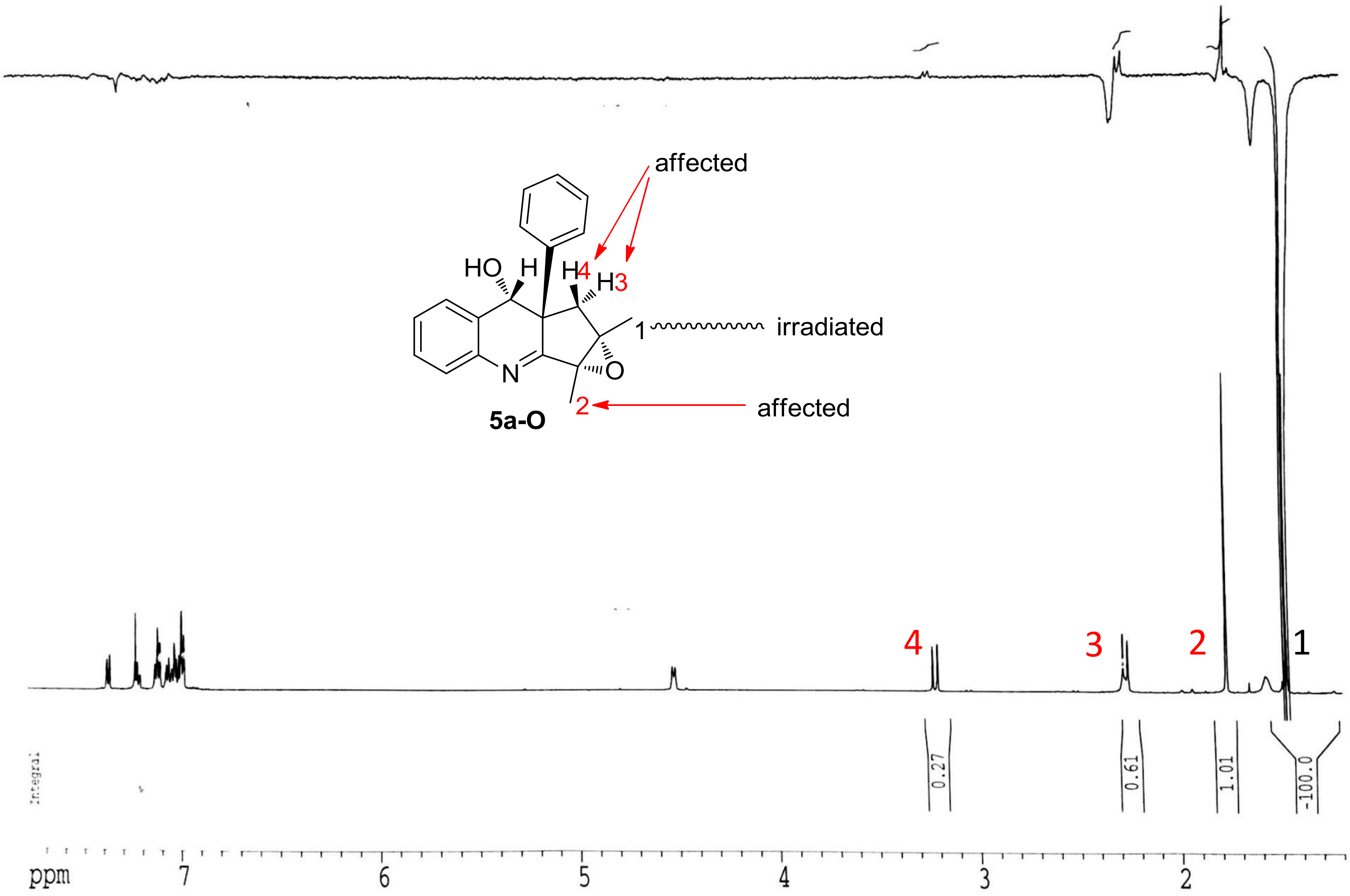

[1]H NOE of compound **5a-O**

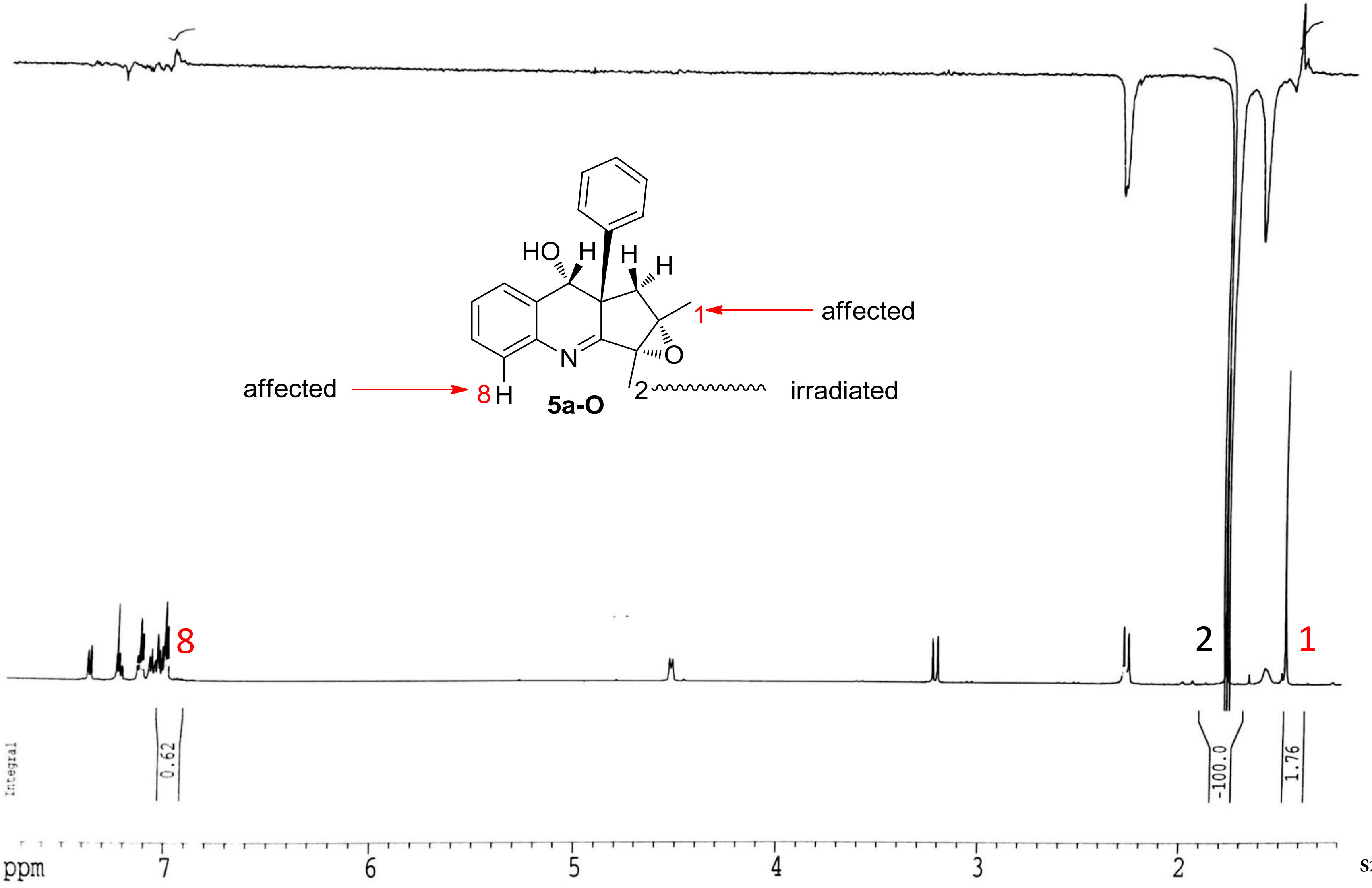

[1]H NOE of compound **5a-O**

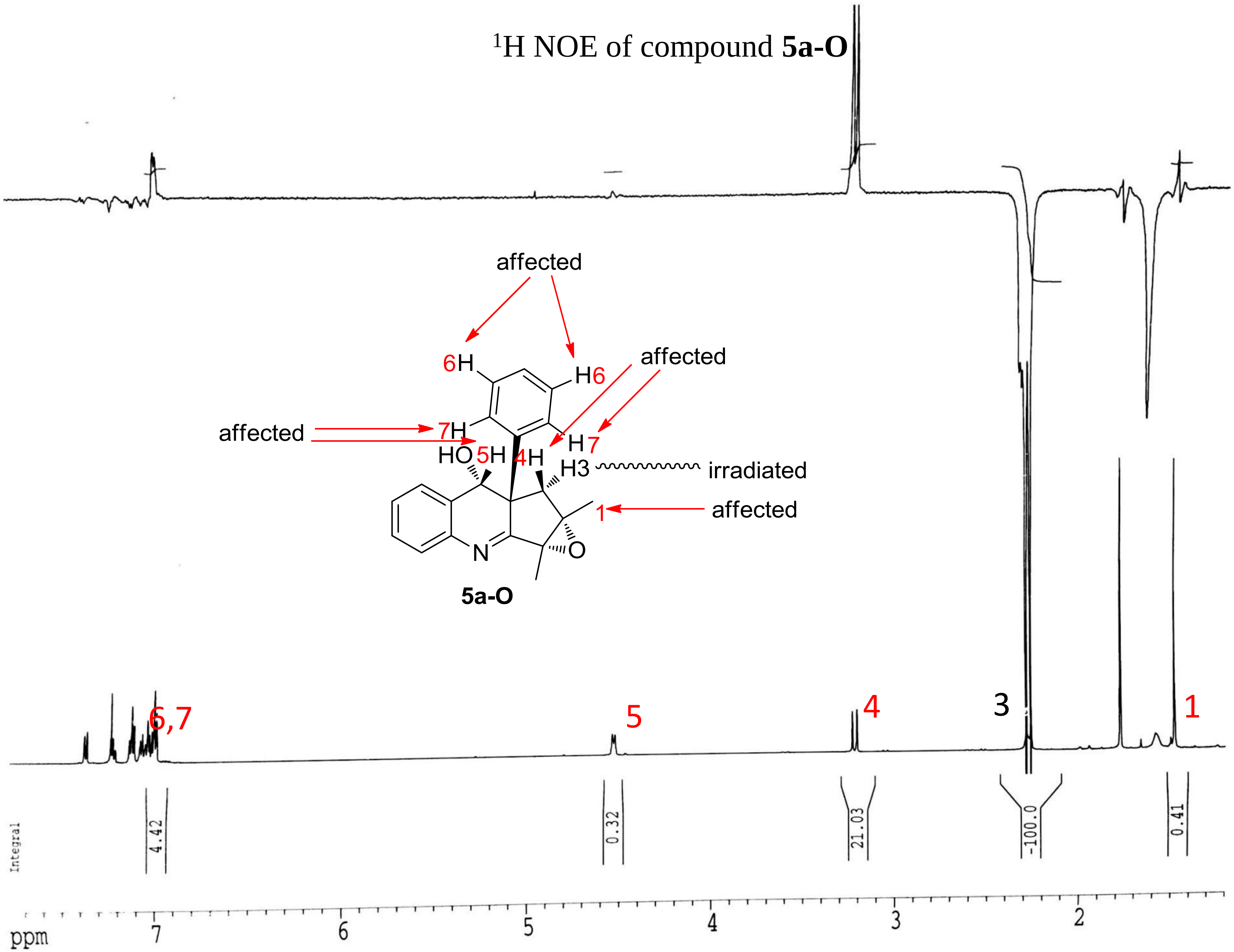

[1]H NOE of compound **5a-O**

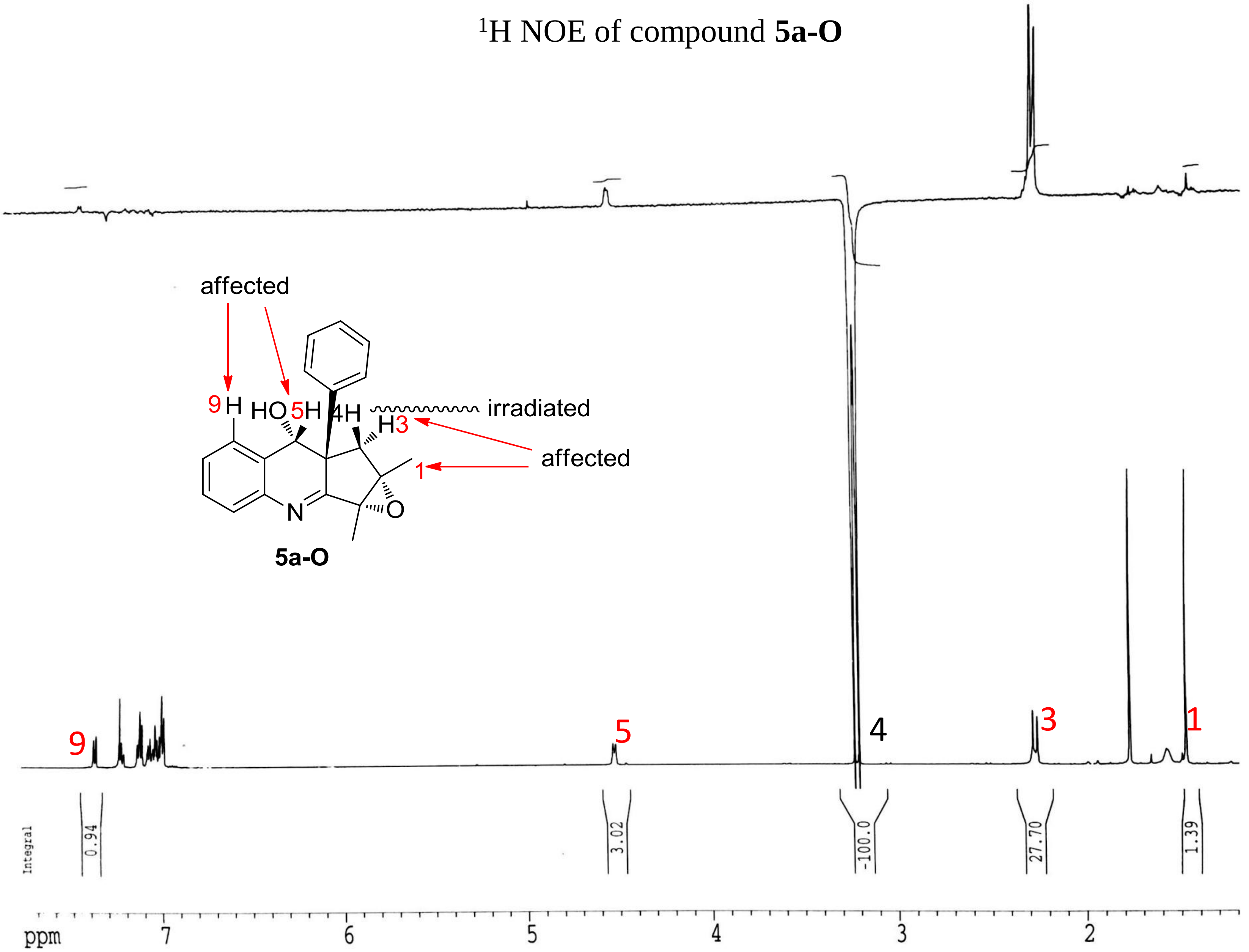

[1]H NOE of compound **5a-O**

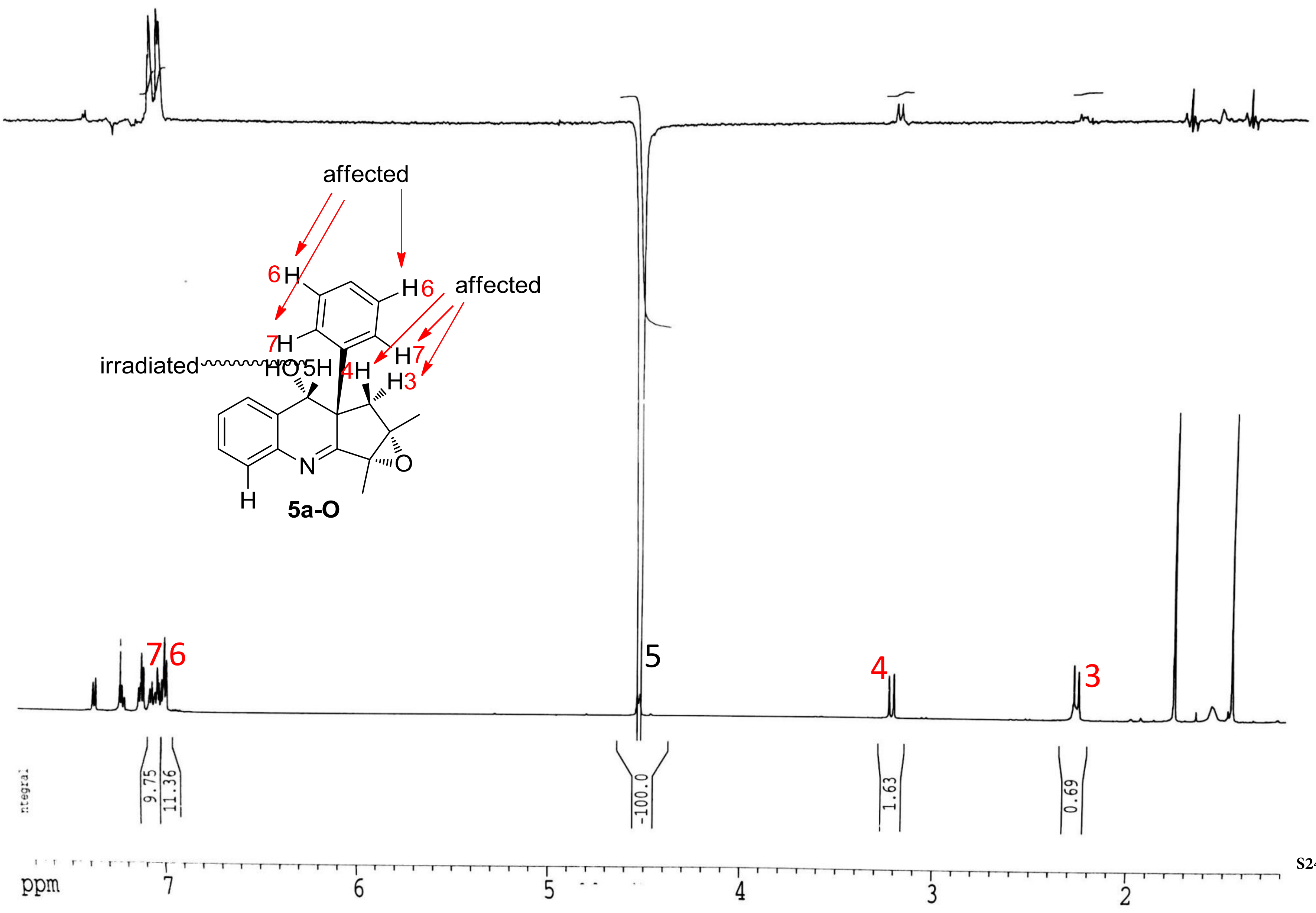

^{1}H NOE of compound **5l**

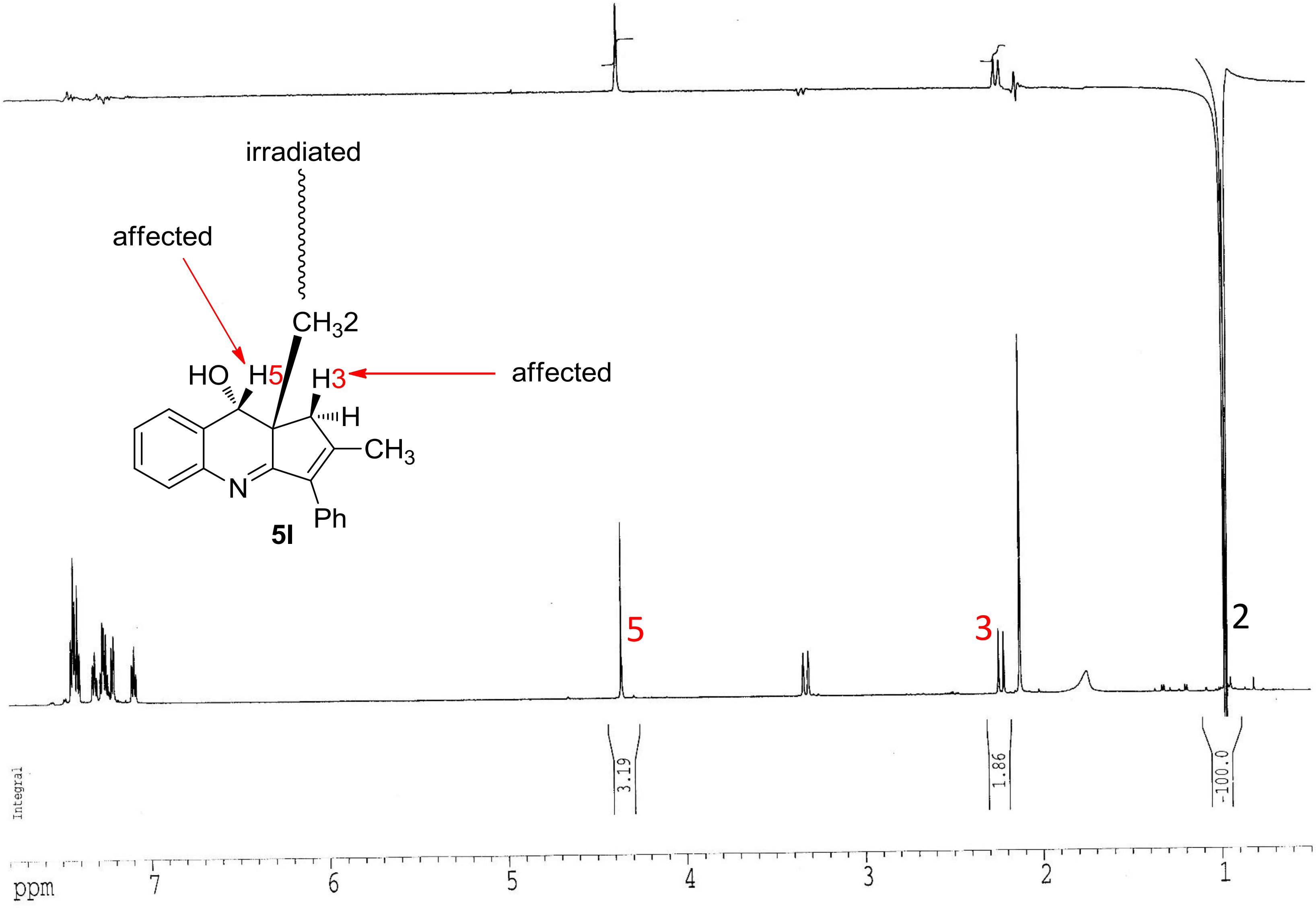

^{1}H NOE of compound **5l**

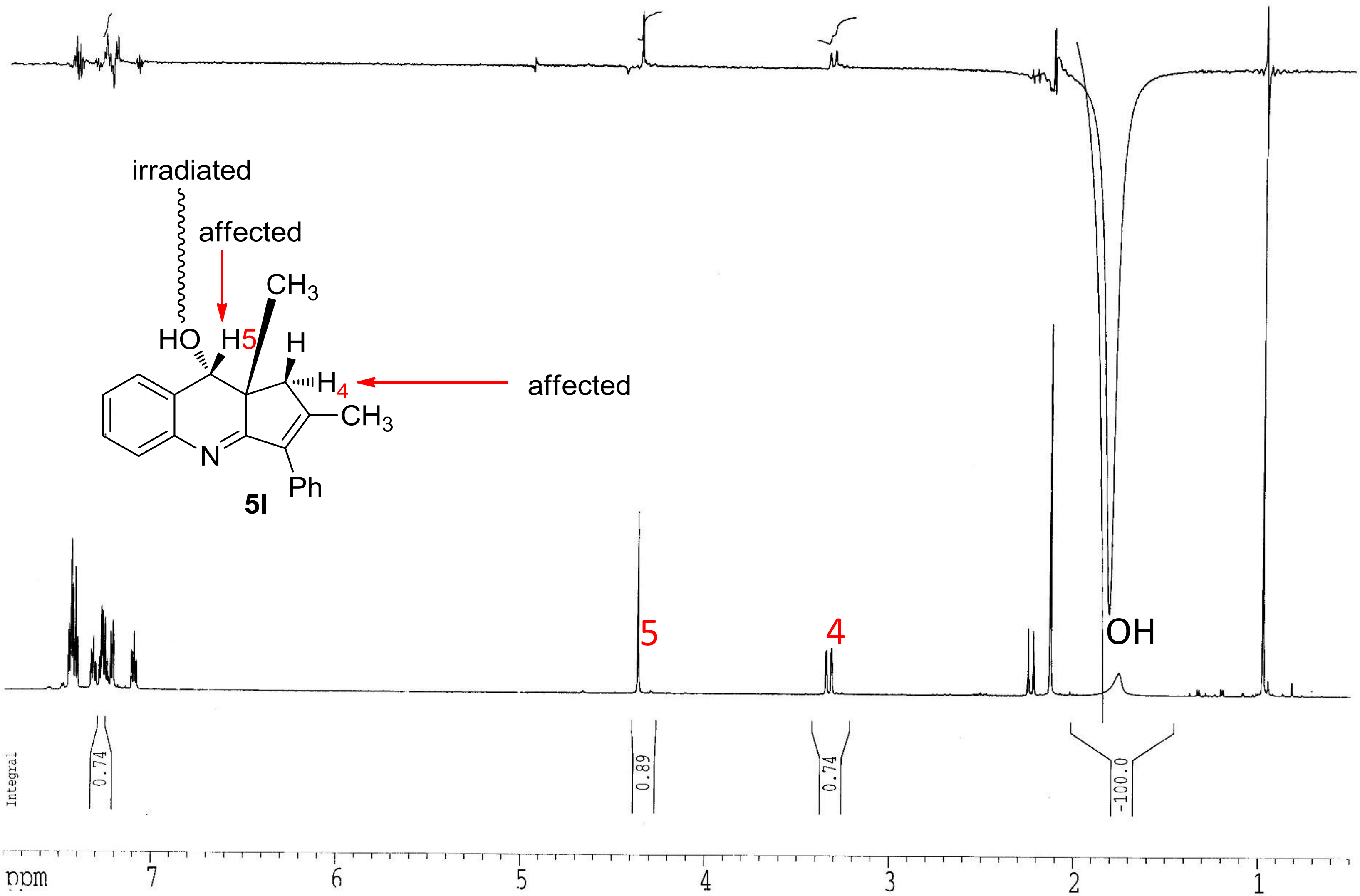

^{1}H NOE of compound **5l**

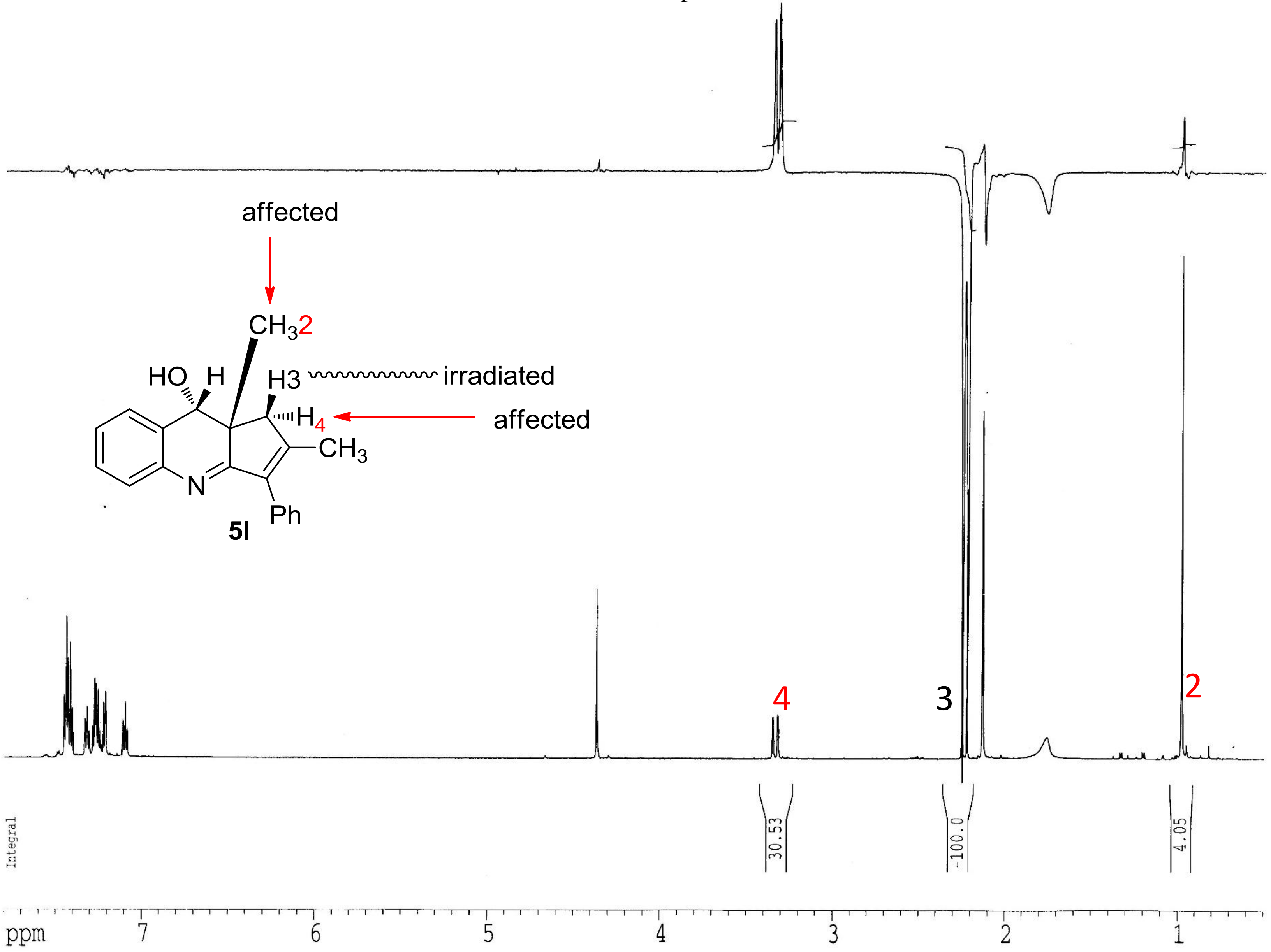

^{1}H NOE of compound **5l**

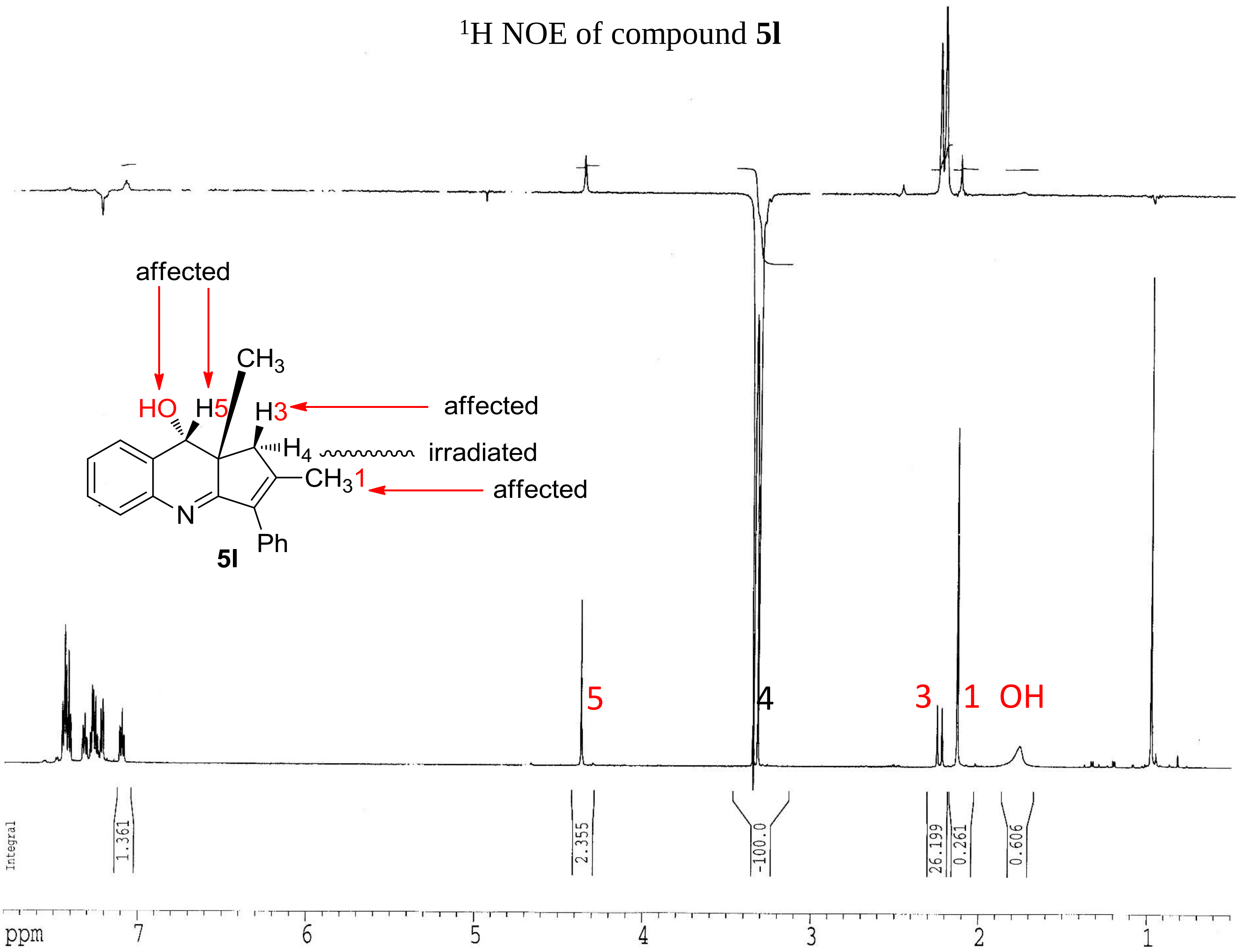

^{1}H NOE of compound **5l**

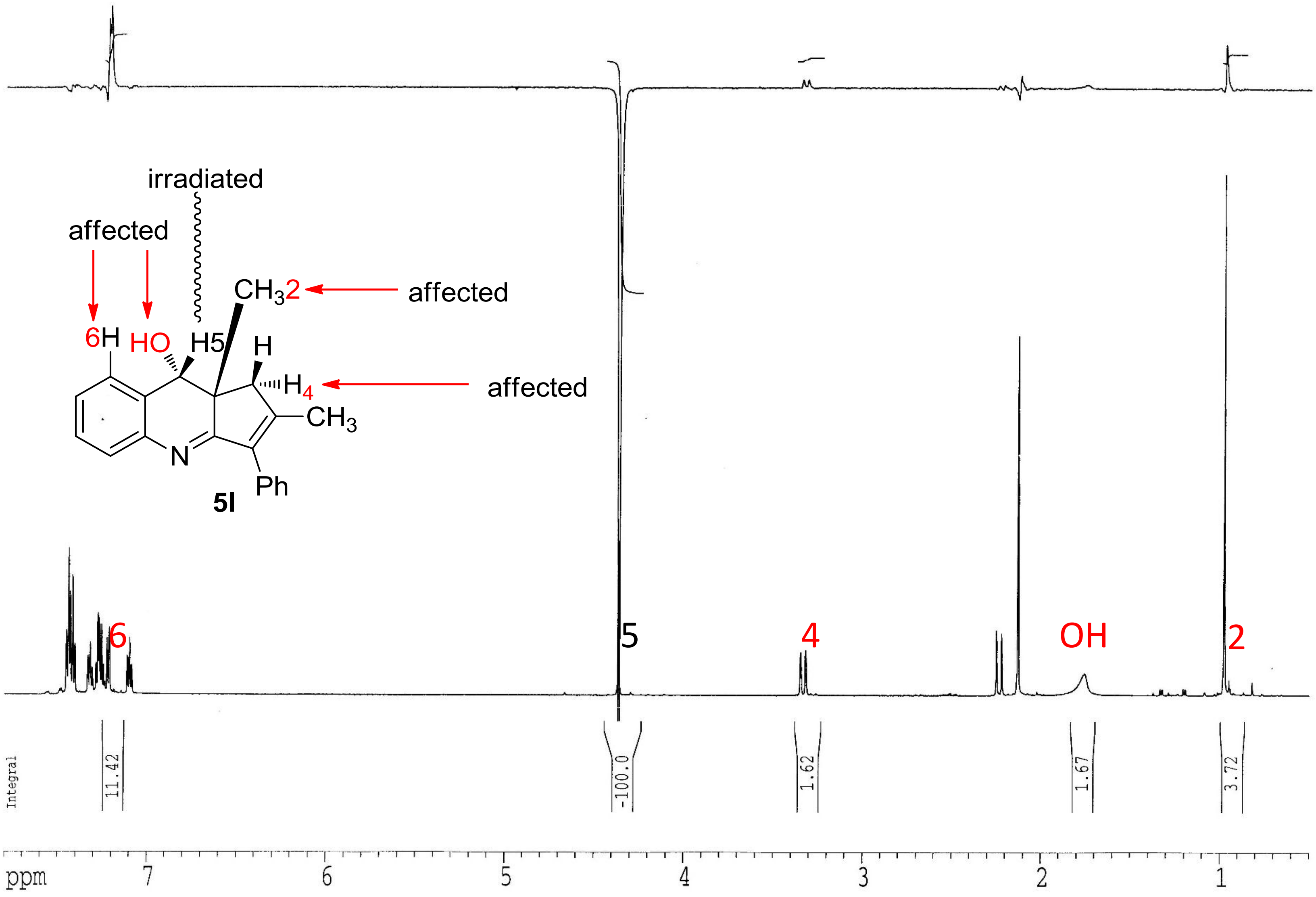

[1]H NOE of compound **8j**

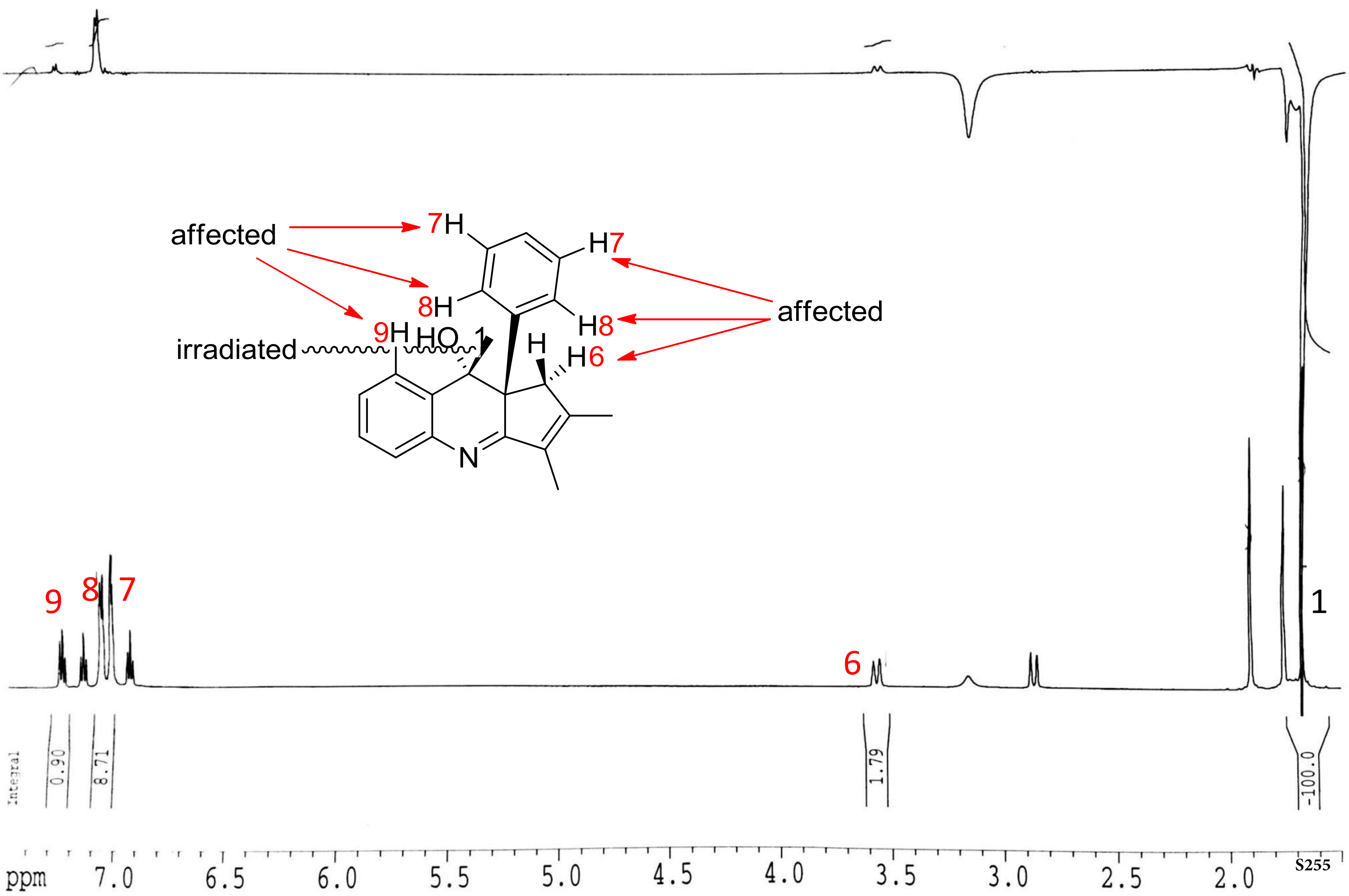

^{1}H NOE of compound **8j**

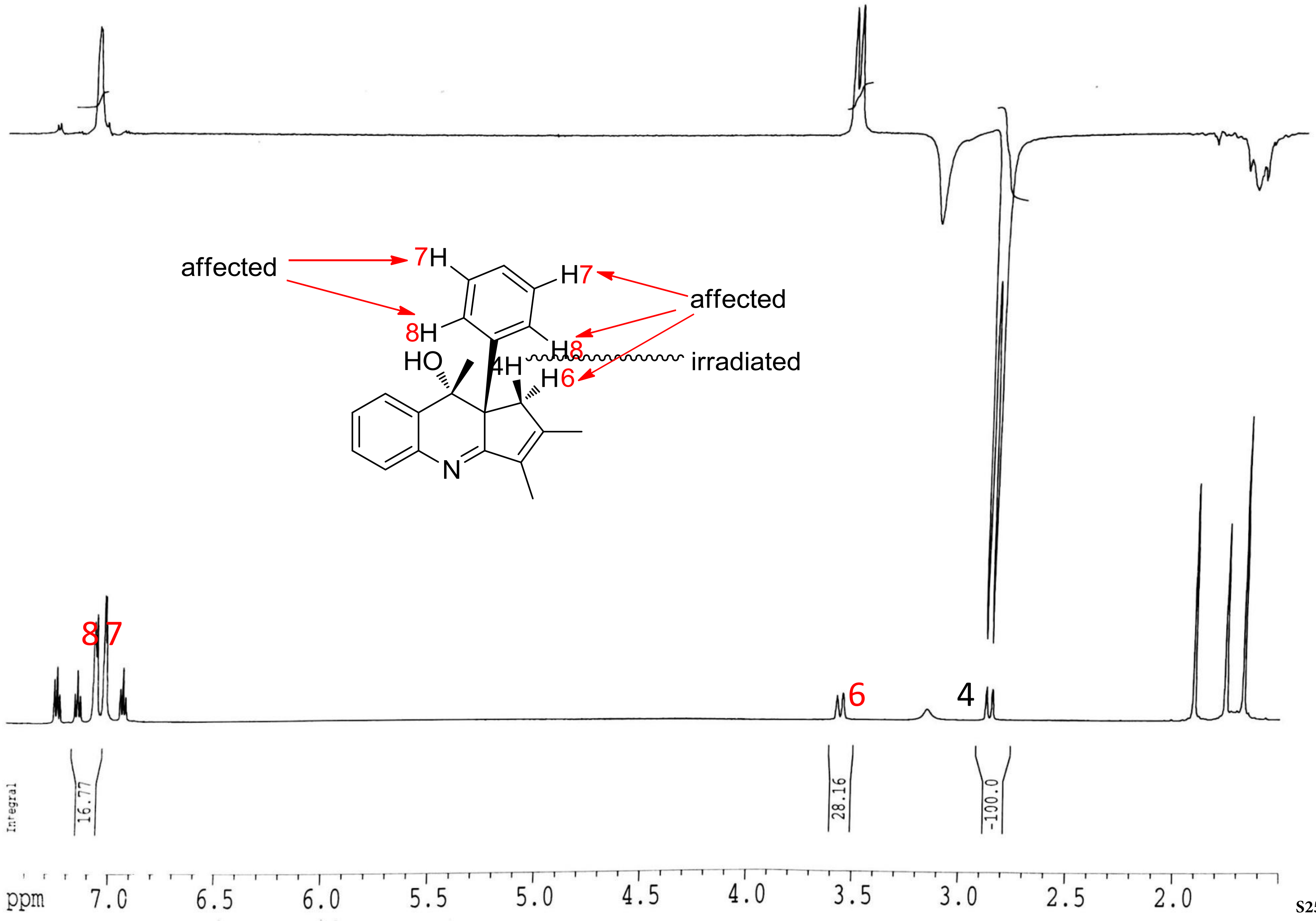

^{1}H NOE of compound **8j**

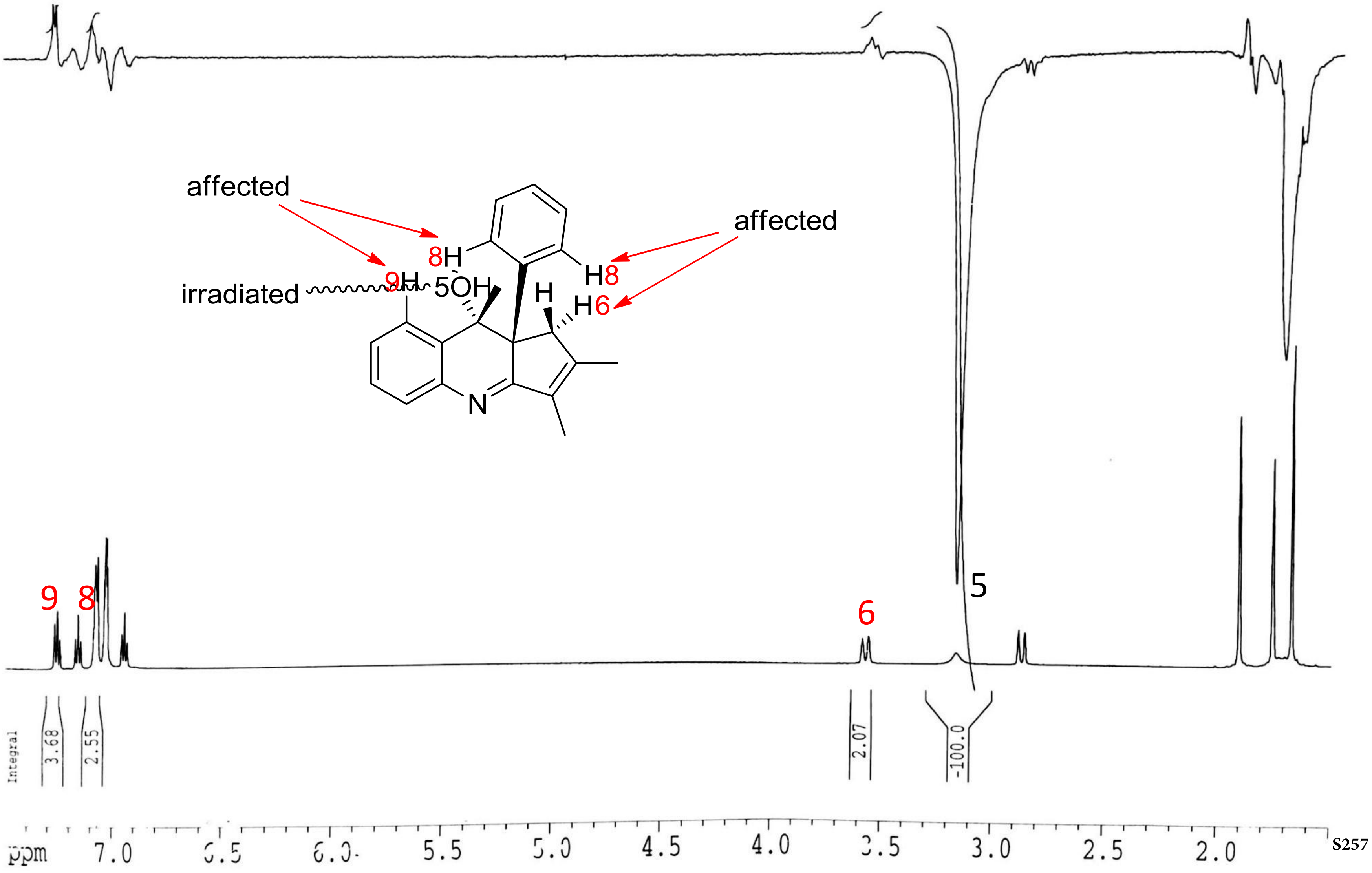

[1]H NOE of compound **8j**

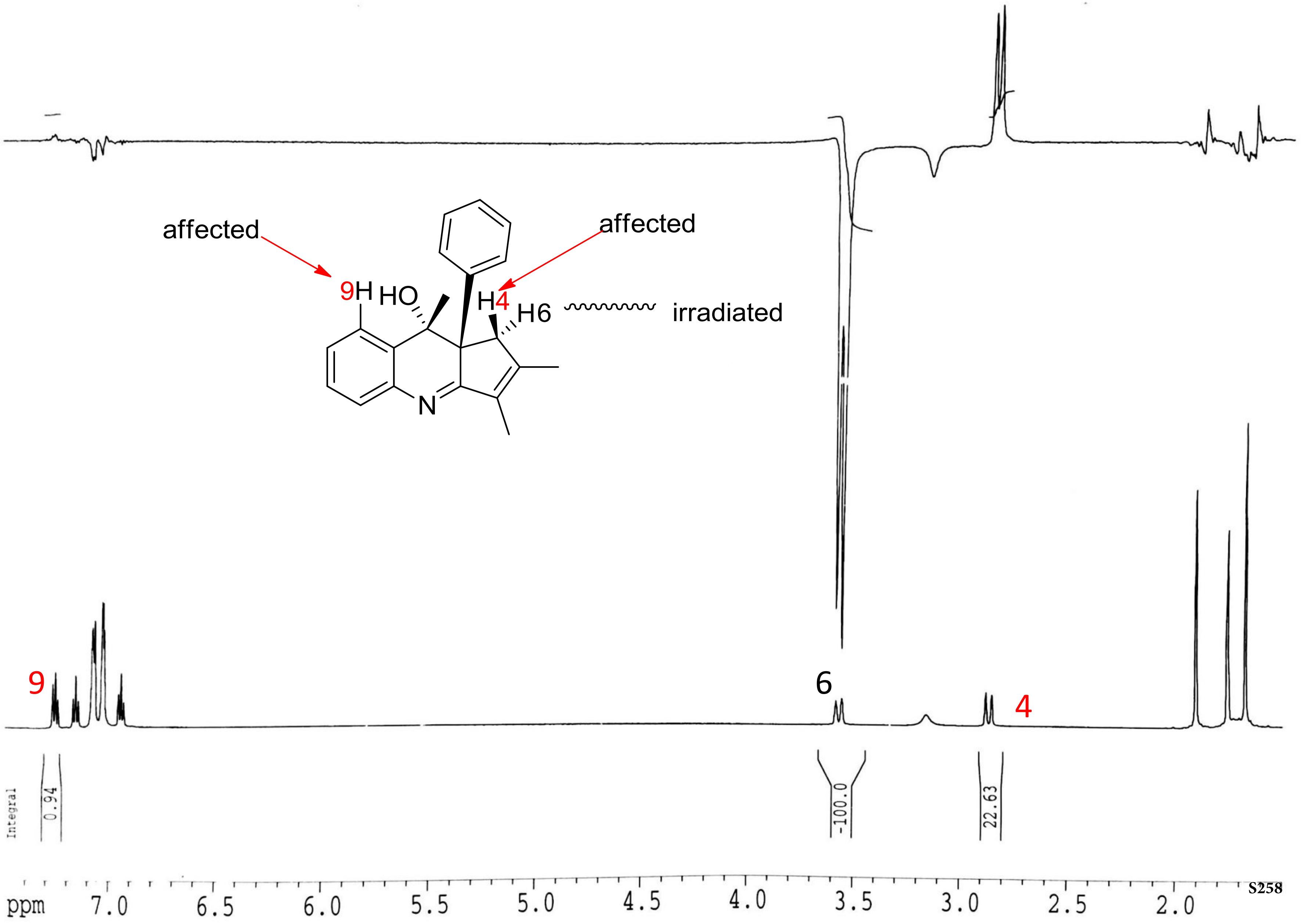

^{1}H NOE of compound **8j**

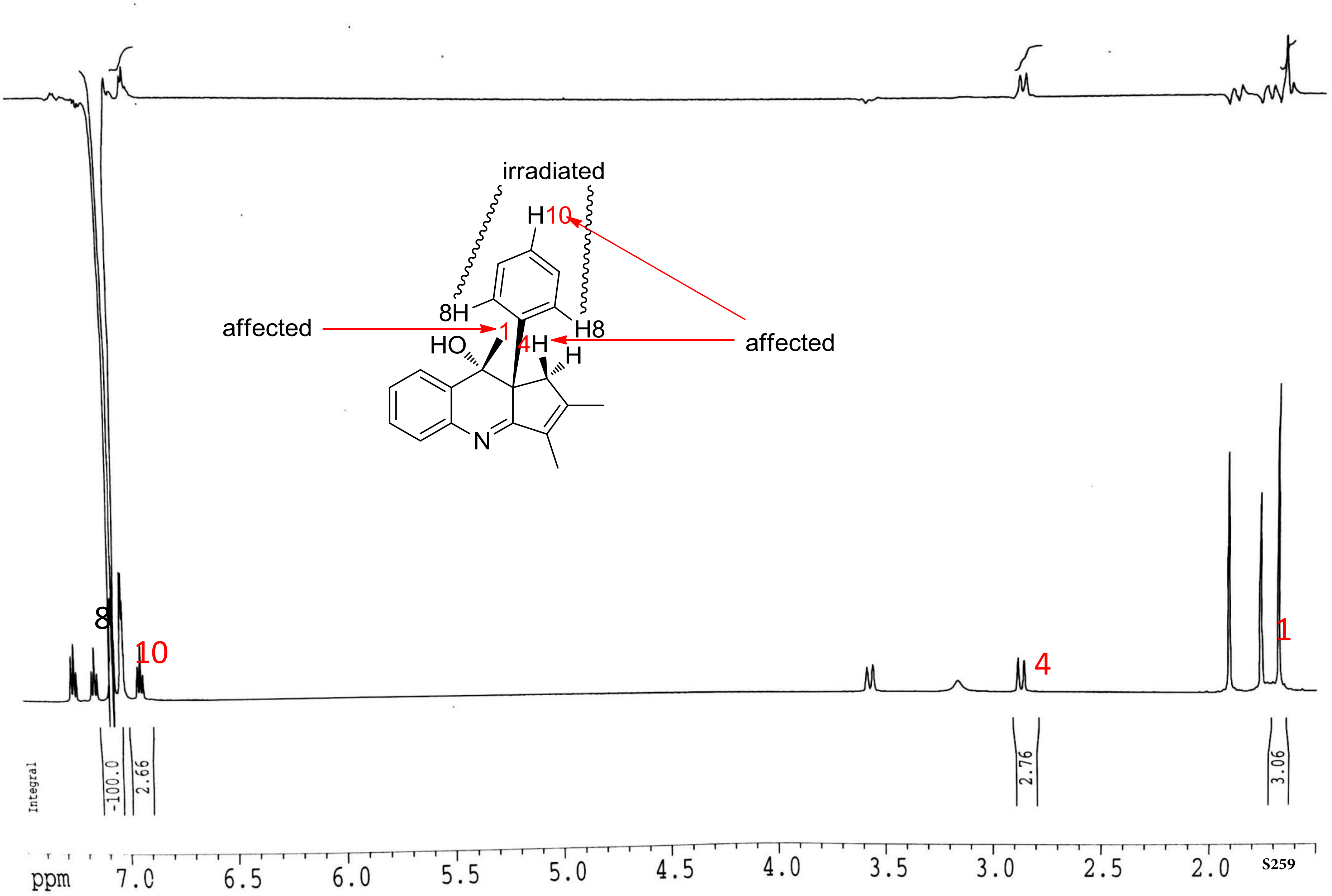

Supplement: Supplementary file 1 [file SC-009-C8SC00986D-s001.pdf]
